# Alterations in the Rumen Liquid-, Particle- and Epithelium-Associated Microbiota of Dairy Cows during the Transition from a Silage- and Concentrate-Based Ration to Pasture in Spring

**DOI:** 10.3389/fmicb.2017.00744

**Published:** 2017-05-02

**Authors:** Melanie Schären, Kerstin Kiri, Susanne Riede, Mark Gardener, Ulrich Meyer, Jürgen Hummel, Tim Urich, Gerhard Breves, Sven Dänicke

**Affiliations:** ^1^Institute of Animal Nutrition, Friedrich-Loeffler-Institute, Federal Research Institute for Animal HealthBrunswick, Germany; ^2^Department of Physiology, University of Veterinary Medicine HannoverHannover, Germany; ^3^Environment, Earth & Ecosystems, The Open UniversityMilton Keynes, UK; ^4^Department of Animal Sciences, University of GoettingenGoettingen, Germany; ^5^Institute of Microbiology, University of GreifswaldGreifswald, Germany

**Keywords:** dairy cow nutrition, rumen microbiota, pasture, ration change, PCR-SSCP, amplicon sequencing

## Abstract

In spring dairy cows are often gradually transitioned from a silage- and concentrate-based ration (total mixed ration, TMR) to pasture. Rumen microbiota adaptability is a key feature of ruminant survival strategy. However, only little is known on the temporal and spatial microbial alterations involved. This study aims to investigate how the rumen liquid (LAAB), particle (PAAB), and epithelium (EAAB) associated archaea and bacteria are influenced by this nutritional change. A 10-wk trial was performed, including 10 rumen-fistulated dairy cows, equally divided into a pasture- and a confinement- group (PG and CG). The CG stayed on a TMR-based ration, while the PG was gradually transitioned from TMR to pasture (wk 1: TMR-only, wk 2: 3 h/day on pasture, wk 3 & 4: 12 h/day on pasture, wk 5–10: pasture-only). In wk 1, wk 5, and wk 10 samples of solid and liquid rumen contents, and papillae biopsies were collected. The DNA was isolated, and PCR-SSCP and 16S rRNA gene amplicon sequencing analysis were performed. Cluster analysis revealed a higher similarity between LAAB and PAAB, compared to the EAAB, characterized by higher species diversity. At all three locations the microbiota was significantly influenced by the ration change, opposite the generally acknowledged hypothesis that the EAAB remain more consistent throughout dietary changes. Even though the animals in the PG were already on a full-grazing ration for 4–6 days in wk 5, the microbiota at all three locations was significantly different compared to wk 10, suggesting an adaptation period of several days to weeks. This is in line with observations made on animal level, showing a required time for adaptation of 2–3 weeks for production and metabolic variables. A large part of the rumen prokaryote species remained unaltered upon transition to pasture and exhibited a strong host influence, supporting the hypothesis that the rumen microbiota consists of a core and a variable microbiota. For the effect of the location as well as the ration change either very similar or opposite trends among member species of common taxa were observed, demonstrating that microbes that are phylogenetically close may still exhibit substantially different phenotypes and functions.

## Introduction

In temperate climate zones dairy cows often receive a silage and concentrate-based ration (total mixed ration, TMR) during winter time and are then gradually transitioned to a pasture-based ration in spring. Since the two systems (confinement and pasture) do not only differ substantially in ration composition, but also how feed is acquired, considerable metabolic as well as behavioral adaptations are required upon this nutritional change (Osuji, 1974; Kolver, 2003). The adaptability of rumen microbiota is a key feature of ruminant physiology and survival strategy (Russell and Rychlik, 2001; McCann et al., 2014a; Zanton, 2015). It has been shown that whenever cows undergo a ration change rumen microbiota needs between 1 day and sometimes even longer than 3 weeks to adapt and stabilize, depending on the group of bacteria, archaea, fungi, or protozoa, the extent of diet change and the behavioral adaptation required (Hackmann, 2015). de Menezes et al. (2011) have shown in a cross-over design, with 2 weeks for diet adaptation, that the liquid and solid rumen bacterial and archaeal community of TMR and pasture fed dairy cows differs significantly. Furthermore, Nakano et al. (2013) showed that rumen microbiota needs 3–4 weeks to adapt to a pasture-based ration when no gradual adaptation to the new nutritional situation is granted. In both studies Prevotellaceae were more prevalent on pasture and a possible key role of this bacterial family in reducing methane production and in transitioning cows to a pasture-based ration was suggested (de Menezes et al., 2011; Nakano et al., 2013; McCann et al., 2014a). However, further data on time required for adaptation of rumen microbiota during the gradual transition from a TMR to a pasture-based ration, and the prokaryotes playing a key role during this nutritional change are lacking.

Aside the particle- and liquid-associated, a third rumen bacterial community has been described. The epithelium-associated or “epimural” microbiota has been investigated in few studies and it has been suggested that it is associated with fermentation end-products, volatile fatty acid (VFA) absorption, maintaining an anaerobe environment, recycling of endogenous nitrogen, and tissue (Cheng et al., 1979; Wallace et al., 1979; McCann et al., 2014a). It has been further speculated whether this microbial community may remain more consistent through dietary changes compared with the particle- and liquid-associated bacterial community (Sadet et al., 2007; McCann et al., 2014a).

In previous publications, we described the alterations in production and rumen variables during a gradual transition from a TMR- to a pasture-based ration (Schären et al., 2016a,b). Primarily a decrease in rumen fermentation activity during the initial phase of transition was observed, most likely due to a decreased intake of fermentable organic matter. After 2–3 weeks on a full-grazing ration an increase in rumen fermentation activity occurred indicated by a decrease in mean daily pH and acetate/propionate ratio as well as an increase in daily pH variation and total VFA concentrations. This was also mirrored in the development of different other rumen (increase of VFA absorption capacity and rumen papillae surface area), performance (stabilization of milk yield and increase in BW) and metabolic (serum non-esterified fatty acid concentrations) variables. We suggested a behavioral and metabolic adaptation after 2–3 weeks on a full-grazing ration leading to an increased intake of fermentable organic matter and therefore rumen fermentation activity and stabilization of milk production. Since rumen microbiota plays a key role in adaptation to a new ration we hypothesized that the effects of a transition from a TMR- to a pasture-based ration observed in other performance, rumen and metabolic variables would also be mirrored in the different rumen archaea and bacteria communities. To investigate whether the archaea and bacteria communities are differently affected by this ration change, polymerase-chain-reaction-single-strand-conformation-polymorphism (PCR-SSCP)- and amplicon sequencing-analysis were performed on samples of the liquid and solid fraction, as well as rumen papillae.

## Materials and methods

Experimental work was conducted at the experimental station of the Institute of Animal Nutrition of the Friedrich-Loeffler-Institute (FLI) in Brunswick, Germany. The experiment was carried out in accordance with the German Animal Welfare Act approved by the LAVES (Lower Saxony State Office for Consumer Protection and Food Safety, Germany; approval number: 33.09-42502-04-11/0444).

### Experimental design and treatments

A 10-wk trial (wk 1–10) was performed from April 21st until June 27th 2014 including 10 rumen-fistulated German Holstein cows (182 ± 24 days in milk, 23.5 ± 3.5 kg milk/d; parity: 4.5 ± 2.2; mean ± SD; at the beginning of the trial). The full trial included 60 dairy cows (166 ± 23 days in milk and 23.5 ± 3.7 kg milk/day; parity: 1.9 ± 1.6; mean ± SD; at the beginning of the trial); the experimental design, treatments, rations, climate data, animal performance, urine variables, clinical chemistry, and total blood counts have been reported previously (Schären et al., 2016a). The rumen fermentation, VFA absorption characteristics and morphology variables assessed in the fistulated animals, as well as their performance data have been separately described (Schären et al., 2016b). The experimental work and data of the present paper have been exclusively conducted and collected in these 10 fistulated animals. At the beginning of the trial the animals were randomly assigned to either a pasture group (PG, *n* = 5) or a confinement group (CG, *n* = 5). The CG stayed in a confinement system and received a TMR throughout the whole trial (35% corn silage, 35% grass silage, 30% concentrate; DM basis), whereas the PG was transitioned from a TMR- to a pasture-based ration (wk 1: TMR-only, wk 2: TMR and 3 h/d on pasture, wk 3 and 4: TMR and 12 h/d on pasture, wk 5–10: pasture and 1.75 kg DM concentrate/d offered in 2 equal meals in troughs after morning and evening milking). A continuous grazing system was implemented on ryegrass dominated pasture. The cows were milked two times per day at 05:30 and 15:00 h and the TMR was fed daily at ~11:00 h. Individual TMR and water intake was continuously recorded in the confinement system using electronic weighing throughs (Insentec, B.V., Markenesse, The Netherlands). Dry matter intake (DMI) on pasture was estimated using the n-alkane method in wk 7 and wk 9 (described in detail in Schären et al., 2016a).

### Sample collection

Sample collection took place at three points in time: at the beginning of the trial (TMR-only, wk1), during the transitional period (PG being 4–6 days on a full-grazing ration, wk 5) and at the end of the trial (PG being 6 weeks on a full-grazing ration, wk 10). All animals were sampled within 3 days of the particular week, between 07:30 and 14:30 h. Firstly, a sample of approximately 200 g rumen solid content was collected at the height of the rumen fistula aperture (pool sample of grab-samples collected from cranial to caudal in the upper half of the rumen fiber mat). Thereafter a 250 mL rumen fluid sample was collected from the ventral site of the rumen (*saccus ventralis*) using a manual pump. Both samples were stored at −20°C within 30 min. Subsequently the total rumen content was evacuated, transferred into insulated barrels and the rumen was washed twice (2 × 10 L water, 39°C). The rumen papillae were then collected at the most ventral site of the ventral rumen sac (*saccus ventralis*; ~5 cm adjacent to the *pila coronaria ventralis*) using a biopsy forceps (Lloyd-Davis biopsy forceps 35 cm, Zepf Instruments, Tuttlingen, Germany). Papillae were immediately washed with a 0.9% NaCl solution, stored in 2 mL cryo tubes (Cryo-Pure Tubes, Sarstedt AG & Co, Nürmbrecht, Germany) and shock frozen using liquid nitrogen. Papillae samples were stored at −80°C until analysis. After the papillae collection a VFA absorption test was performed and the rumen content was reintroduced (detailed protocol in Schären et al., 2016b).

### DNA extraction

#### Rumen liquid content

The separation of the liquid-associated microbes from feed particles and the subsequent DNA extraction have been described by Meibaum et al. (2012) and Schären et al. (2017) (exact protocol). Briefly, several centrifugation steps were performed (once 5 min at 600 *g* (4°C) to remove feed particles and debris, and four times during 20 min at 27,000 *g* (4°C); between each centrifugation step the pellet was re-suspended in 40 mL 0.9% NaCl) and the concentrated samples were liquid shock frozen under the form of droplets for storage at −80°C. After a centrifugation step (13,000 *g*, 5 min, 4°C) the supernatant was discarded and the sample was re-suspended in 1 × tris(hydroxymethyl)-aminomethane-HCl, EDTA (both 10 mM, pH 8.0), and NaCl (150 mM), and a DNA extraction was performed including a mechanical lysis of the cells by bead beating method (Fast Prep, MP Biomedicals, Eschwege, Germany; in two sequences of acceleration, 6.0 and 4.5 m/s, 40 s each). This was followed by different incubation steps including lysozyme and RNaseA (30 min at 37°C), sodiumdodecylsulphate and proteinase K (1 h at 37°C), and 4 M NaCl and cetyltrimethylammoniumbromide (65°C during 10 min). To purify the mixture from proteins phenol-chloroform-isoamylalcohol was added, the mixture was centrifuged (7 min, 13,000 *g*, 4°C), the supernatants were discarded, chloroform-isoamylalcohol was added, centrifuged again (10 min, 13,000 *g*, 4°C) and the supernatant was then kept for further processing. As a final step the samples were further purified using the peqGold Tissue-Kit (peqlab, Erlangen, Germany) according to manufacturer's guidelines. The genomic DNA (gDNA) samples were then stored at 4°C until further processing.

#### Rumen solid content

To remove all liquid-associated bacteria from the sample several washing steps were performed per sample (10 g sample, 4–5 washing steps with each 1 L 0.9% NaCl, using a 4 mm sieve, until washing solution was clear). Thereafter the fiber particles were transferred into a 50 mL vessel, immersed in sterile 0.9% NaCl solution and sonicated in an ultrasonic-bath during 30 min to detach the particle associated bacteria. Thereafter the sample was sieved (4 mm sieve), centrifuged at 27,000 g during 20 min (4°C), the supernatant discarded and the pellet was resuspended in 1,000 μl 0.9% NaCl. For DNA extraction and purification 200 μl of the microbe-pellet and the peqGold Tissue-Kit was used (according to manufacturer's guidelines; 1. Incubation: 150 μL TE-Puffer, 50 μL lysozyme, 30 min at 30°C on thermoshaker; 2. Incubation: 400 μL DNA lysis puffer, 20 μL proteinase K, 15 μL RNaseA, 60 min at 50°C on thermoshaker). The gDNA samples were then stored at 4°C until further processing.

#### Rumen papillae

Rumen papillae samples were thawed on ice and 120 mg of each sample were washed twice with 1,000 μL sterile 0.9% NaCl. Thereafter DNA extraction (400 μL DNA lysis buffer, 20 μL proteinase K, 15 μL RNaseA, 50 min at 60°C on thermoshaker) and purification was performed using the pegGold Tissue-Kit according to manufacturer's guidelines and samples were then stored at 4°C until further processing.

### PCR-SSCP analysis

After DNA extraction a two-step amplification (initial and nested PCR) of the bacteria specific 16S rRNA gene regions and a single-strand digestion step were performed (protocol and primers described in detail in Meibaum et al., 2012). To compare the bacterial populations at the three different locations in the rumen, as well as the change over time in both groups, 12 different SSCP-gels were created: 6 gels comparing the liquid- (LAB), particle- (PAB), and epithelium- (EAB) associated bacteria at one particular point in time in the PG or CG, 6 gels comparing the LAB, PAB or EAB at the three points in time (wk 1, wk 5, and wk 10) in the PG or CG. Gel-electrophoresis was carried out at 300 V during 22.5 h at 20°C (described in detail in Dohrmann et al., 2004). The gels were digitalized and analyzed using ScanMaker (i800, Mikrotek, Willich, Germany) and GelComparII (Applied Maths, Sint-Martens-Latem, Belgium) as described in Meibaum et al. (2012). For graphical illustration two dimensional principal co-ordinates analysis (PCO) plots based on dissimilarities were created with the *cmdscale()* command in the R Guide 3.0.2 software package (R-Core-Team, 2013).

### Prokaryotic 16S rRNA gene amplification, illumina MiSeq sequencing and bioinformatics

For sequencing gDNA samples were sent to Microsynth AG (Balgach, Switzerland). A primer pair with 97.7/98.4% (forward primer) and 96.9/96.5% (reverse primer) coverage (one mismatch) for archaea and bacteria, respectively, was chosen for 16S sequencing library preparation: A519F (S-D-Arch-0519-a-S-15): CAGCMGCCGCGGTAA and 802R (S-D-Bact-0785-b-A-18): TACNVGGGTATCTAATCC (Klindworth et al., 2013). Due to the additional inclusion of the archaea in this approach (in comparison to the PCR-SSCP analysis), samples will be referred to as liquid- (LAAB), particle- (PAAB), and epithelium- (EAAB) associated bacteria and archaea. For 16S rDNA amplification the HiFi HotStart PCR Kit (Kapa Biosystems, Wilmington, MA, USA) was used with following PCR conditions: initial denaturation (95°C, 180 s), denaturation (98°C, 20 s), annealing (50.8°C, 30 s) and elongation (72°C, 30 s) with 30 cycles, and a final elongation step (72°C, 5 min). Further, the Illumina Nextera Libraries were prepared according to the manufacturers instruction (Illumina, San Diego, USA). Sequencing was performed on the Illumina MiSeq Sequencing System using the Illumina MiSeq reagent Kit v2 (2 × 250 bp). Sequence data were demultiplexed and trimmed using the Illumina MiSeq v2.5.1.3. reporter and cutadapt v1.8.1 software package (Martin, 2011). Read stitching was performed using FLASH v1.2.11 (Magoč and Salzberg, 2011) and only stitched reads with an average quality score (whole read) of 25 or higher were used for downstream analysis. Further, *de novo* Chimera detection, identification and removal was done using the Uchime v4.2 (Edgar et al., 2011) and Usearch v8.1.1861 (Edgar, 2010) software package. The taxonomic assignment and the OTU clustering (based on 97% sequence similarity) were performed using Uclust (Edgar, 2010) and QIIME v1.9.1 (Caporaso et al., 2010), respectively. Only matches with a minimum sequence similarity of ≥90% and a score 0.67 or 1.00 in the greengenes database were used. Singeltons were removed from the dataset to reduce bias introduced by sequencing errors. As a reference database for the taxonomic assignment the SILVA rRNA database v111 was chosen (Quast et al., 2013). For downstream analysis only OTUs with a relative abundance of at least 0.1% were considered. Alpha diversity analysis was performed and PCO plots were created using QIIME. Robustness of clusters displayed in PCO plots was ensured by jackknife resampling (10-fold).

### Statistical analysis

All statistical analyses were performed using the R 3.0.2 software package. In case of the SSCP-gels a PERMANOVA was performed using the *adonis()* function in the R software package vegan (Oksanen et al., 2015). To evaluate the alterations in similarity of samples on the SSCP gels over the course of the trial [comparing each sample in wk 5 and wk 10 to its reference sample of wk 1 (within the same cow)] a repeated measures ANOVA using the *aov()* function was performed. Alpha diversity variables (chao1 index, observed species, and Shannon index) were analyzed via a PERMANOVA using the *aovp()* function of the lmPerm software package (Wheeler, 2010). Beta-diversity was evaluated based on the weighted UniFrac distances via a PERMANOVA using the *adonis()* function in the software package vegan. For species level comparison a PERMANOVA model using the *aovp()* function in the software package lmPerm was performed. The model included Group, Time and Location and their interactions as well as the Cow and a Cow × Time interaction as fixed factor and the Cow as random factor. Results were considered significant at *P* ≤ 0.05 and a trend declared at 0.05 < *P* ≤ 0.10.

## Results

### SSCP analysis

**Figure 1:**
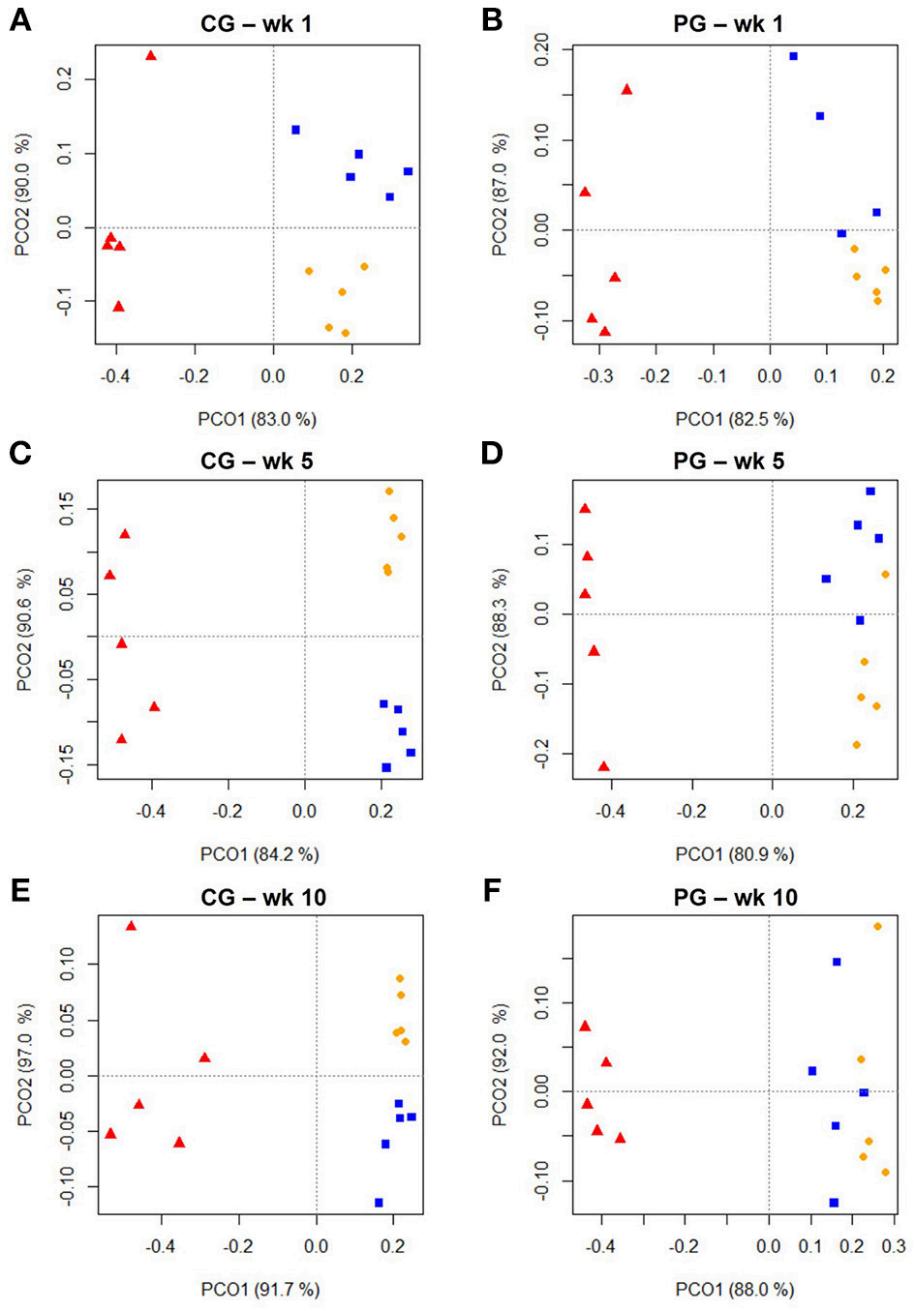
**Two dimensional PCO-plots from SSCP-gels of rumen liquid (LAB, orange dot), particle (PAB, blue square) and epithelium (EAB, red triangle) associated bacteria of the confinement group (CG) and pasture group (PG) in wk 1, wk 5, and wk 10 (explained variance indicated in % on x- and y-axis, *n* = 5)**. The CG stayed on a TMR based ration during the entire trial while the PG was slowly introduced to a pasture-based ration: wk 1: TMR, wk 2: TMR and 3 h pasture/d, wk 3 and 4: TMR and 12 h pasture/d, wk 5–10: pasture and 1.75 kg DM concentrate/d. Significance: **(A)** *P* = 0.001, **(B)** *P* = 0.001, **(C)** *P* = 0.001, **(D)** *P* = 0.001, **(E)** *P* = 0.001, **(F)** *P* = 0.002.

**Figure 2:**
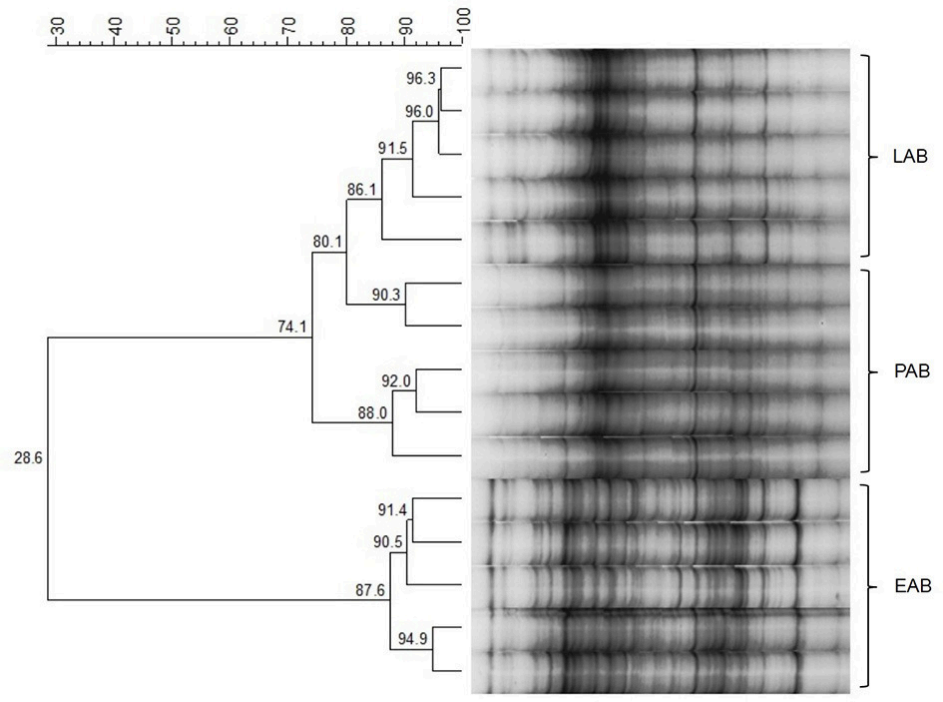
**Example of SSCP-Gel and dendrogram of rumen liquid (LAB), particle (PAB), and epithelium (EAB) associated bacteria at one point in time during the trial (wk 5, confinement group, *n* = 5)**. Numbers indicate similarity (in %) between samples/clusters.

**Figure 3:**
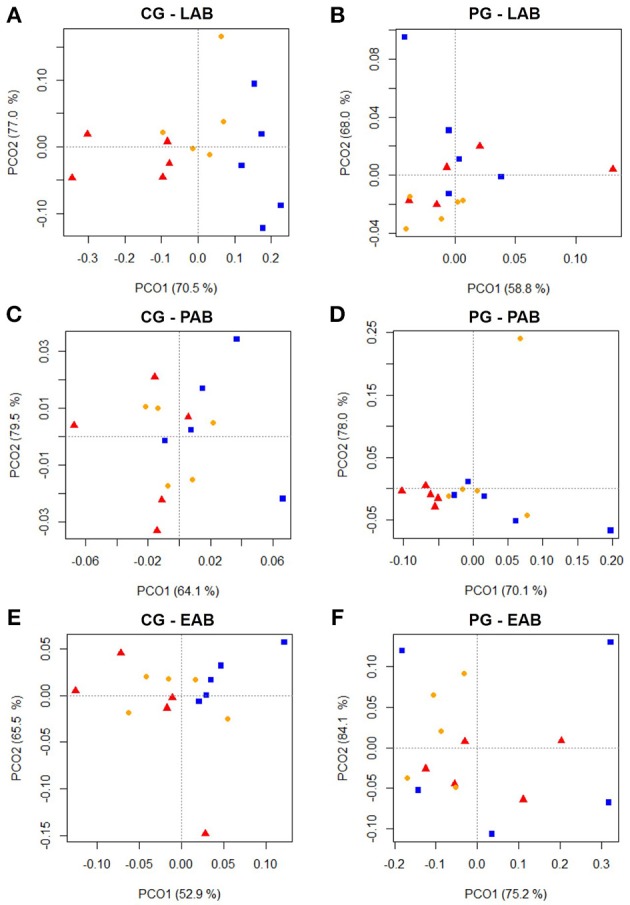
**Two dimensional PCO-plots from SSCP-gels of rumen liquid (LAB), particle (PAB), and epithelium (EAB) associated bacteria illustrating changes over the course of the trial (wk 1 = red triangle, wk 5 = orange dot, wk 10 = blue square) in the confinement group (CG) and pasture group (PG; *n* = 5; explained variance indicated in % on x- and y-axis)**. The CG stayed on a TMR based ration during the entire trial while the PG was slowly introduced to a pasture-based ration: wk 1: TMR, wk 2: TMR and 3 h pasture/d, wk 3 and 4: TMR and 12 h pasture/d, wk 5–10: pasture and 1.75 kg DM concentrate/d. Significance: **(A)** *P* = 0.141, **(B)** *P* = 0.001, **(C)** *P* = 0.080, **(D)** *P* = 0.328, **(E)** *P* = 0.013, **(F)** *P* = 0.115.

**Figure 4:**
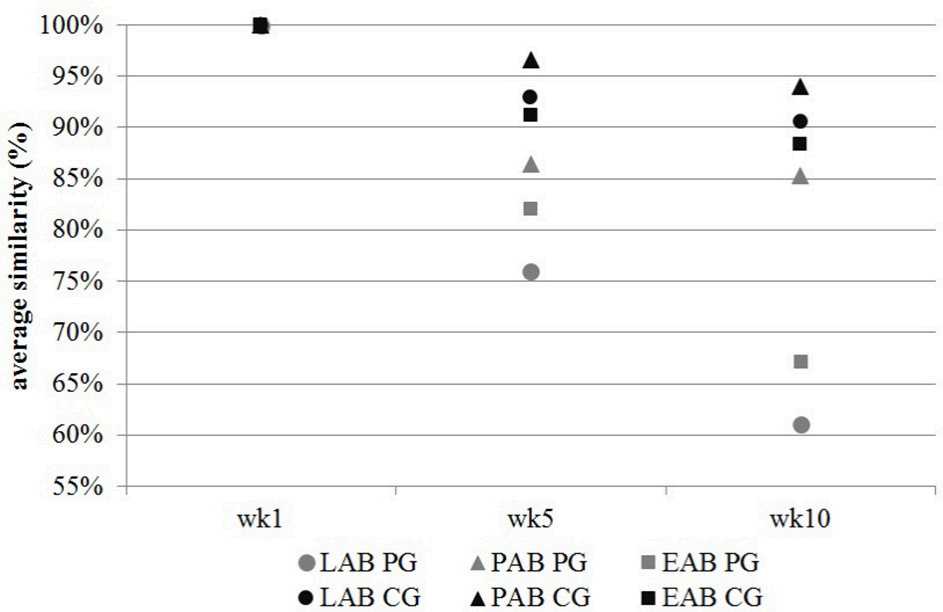
**Change in bacterial communities of the liquid- (LAB), particle- (PAB), and epithelium-associated bacteria (EAB) over time expressed in average similarity (in %) of samples compared to their reference sample in wk 1. CG, confinement group, PG, pasture group (*n* = 5)**. A significant greater decrease in similarity over time in all three bacteria populations was observed for the PG compared to the CG (*P*_EAB_ = 0.002, *P*_LAB_ = 0.008; *P*_PAB_ = 0.003; SD_EAB_ = 11%; SD_LAB_ = 8%, SD_PAB_ = 16%). The CG stayed on a TMR based ration during the entire trial while the PG was slowly introduced to a pasture-based ration: wk 1: TMR, wk 2: TMR and 3 h pasture/d, wk 3 and 4: TMR and 12 h pasture/d, wk 5–10: pasture and 1.75 kg DM concentrate/d.

Cluster analysis displayed a clustering of LAB, PAB, and EAB in both ration types and all three points in time, with a higher similarity among LAB and PAB, compared to EAB samples (Figure 1). LAB and PAB had an average similarity of 82 ± 8% and differ strongly from the EAB with a similarity of 39 ± 11% EAB compared to LAB, and 37 ± 10% EAB compared to PAB (mean ± SD). For illustrational purposes the dendrogram and SSCP-gel of the comparison of the samples collected in wk 5 in the CG are depicted in Figure 2. Within the different bacteria communities, a significant influence of time was only observed for the LAB in the PG and the EAB in CG (Figure 3). However, when comparing the samples of the different bacteria populations in wk 5 and wk 10 to their reference sample in wk 1, a significant greater decrease in similarity over time in all three bacteria populations was observed for the PG compared to the CG (Figure 4).

### 16S rRNA gene amplicon analysis

For the LAAB, PAAB and EAAB a total number of 2,151 ± 312, 2,615 ± 338, and 662 ± 161 different OTUs were detected, respectively. Filtering (exclusion of OTUs with a relative abundance < 0.1%) resulted in a total number of 177 different OTUs with an average of 167 ± 5 (LAAB), 162 ± 4 (PAAB), and 74 ± 8 (EAAB) different OTUs per sample (mean ± SD, Table 1), with an average of 12,882 ± 3,389, 11,853 ± 3,433, and 2,129 ± 815 reads per sample (after filtering, mean ± SD), respectively. Four samples from the EAAB were excluded due to an extremely low reads count (wk1 CG, 2x wk 1 PG and wk 5 PG with 942, 590, 859, and 801 reads per sample). Most OTUs could be taxonomically classified to the family level, while their genus or species level affiliation were “uncultured bacterium or archaeon” in many cases (Table 1). One OTU was assigned to an archaeal and 176 OTUs to bacterial taxa.

**Table 1:** **Summary of detected OTUs, location and treatment effects^a^**.

**Boxplot Nr.^b^**	**OTU Nr.^c^**	**Taxonomy^d^**					**Location Effect^e^**				**Treatment Effect^f^**				**Cow Effect^g^**
		**Phylum**	**Class**	**Order**	**Family**	**Genus**	***P*****−value**	**EAAB**	**LAAB**	**PAAB**	***P*****−value**	**EAAB**	**LAAB**	**PAAB**	
A.	EF112194	Euryarchaeota	Methanobacteria	Methanobacteriales	Methanobacteriaceae	Methanobrevibacter	***	+	+++	−	−				−
B1.	EU779121	Actinobacteria	Actinobacteria	Bifidobacteriales	Bifidobacteriaceae	u.b.	***	−	++	+	*				−
B2.	AB559503	Actinobacteria	Actinobacteria	Bifidobacteriales	Bifidobacteriaceae	Bifidobacterium	***	−	++	++	−				*
B3.	AM277978	Actinobacteria	Actinobacteria	Bifidobacteriales	Bifidobacteriaceae	Bifidobacterium	**	−	+	++	†		d	d	*
C1.	EU469015	Actinobacteria	Coriobacteriia	Coriobacteriales	Coriobacteriaceae	Atopobium	***	−	++	−	**		ii		*
C2.	AB270014	Actinobacteria	Coriobacteriia	Coriobacteriales	Coriobacteriaceae	Atopobium	***	−	++	−	**		ii		−
C3.	New.Ref.OTU	Actinobacteria	Coriobacteriia	Coriobacteriales	Coriobacteriaceae	Atopobium	***	−	++	−	**		ii		−
D.	EF445233	Bacteroidetes	Bacteroidia	Bacteroidales	u.b.		***	−	++	++	−				−
E1.	AB185544	Bacteroidetes	Bacteroidia	Bacteroidales	BS11 gut group	u.b.	***	++	++	+++	−				−
E2.	EF686531	Bacteroidetes	Bacteroidia	Bacteroidales	BS11 gut group	u.b.	***	++	++	++	−				−
E3.	EU773647	Bacteroidetes	Bacteroidia	Bacteroidales	BS11 gut group	u.b.	***	−	+++	++	−				−
E4.	AY244965	Bacteroidetes	Bacteroidia	Bacteroidales	BS11 gut group	u.b.	***	−	++	++	−				*
F1.	AB009235	Bacteroidetes	Bacteroidia	Bacteroidales	Prevotellaceae	Prevotella	***	−	++	++	*			d	−
F2.	EU259377	Bacteroidetes	Bacteroidia	Bacteroidales	Prevotellaceae	Prevotella	***	+	+++	++	−				−
F3.	EF445293	Bacteroidetes	Bacteroidia	Bacteroidales	Prevotellaceae	Prevotella	***	−	++	++	−				−
F4.	AB009192	Bacteroidetes	Bacteroidia	Bacteroidales	Prevotellaceae	Prevotella	***	+	+++	+++	−				−
F5.	New.Ref.OTU	Bacteroidetes	Bacteroidia	Bacteroidales	Prevotellaceae	Prevotella	***	−	++	++	−				−
F6.	AB269981	Bacteroidetes	Bacteroidia	Bacteroidales	Prevotellaceae	Prevotella	***	−	+++	++	−				−
F7.	EF445210	Bacteroidetes	Bacteroidia	Bacteroidales	Prevotellaceae	Prevotella	***	−	+	++	−				−
F8.	EU844726	Bacteroidetes	Bacteroidia	Bacteroidales	Prevotellaceae	Prevotella	***	+	−	++	−				−
F9.	GQ327024	Bacteroidetes	Bacteroidia	Bacteroidales	Prevotellaceae	Prevotella	***	−	++	++	−				†
F10.	EF436359	Bacteroidetes	Bacteroidia	Bacteroidales	Prevotellaceae	Prevotella	***	−	++	++	−				−
F11.	EU719305	Bacteroidetes	Bacteroidia	Bacteroidales	Prevotellaceae	Prevotella	−	−	−	−	−				−
F12.	AB185608	Bacteroidetes	Bacteroidia	Bacteroidales	Prevotellaceae	Prevotella	***	−	++	++	−				−
F13.	AY244946	Bacteroidetes	Bacteroidia	Bacteroidales	Prevotellaceae	Prevotella	***	+	+++	+++	−				−
F14.	AF018469	Bacteroidetes	Bacteroidia	Bacteroidales	Prevotellaceae	Prevotella	***	−	++	++	−				*
F15.	GQ327306	Bacteroidetes	Bacteroidia	Bacteroidales	Prevotellaceae	Prevotella	***	−	++	++	−				−
F16.	AB034102	Bacteroidetes	Bacteroidia	Bacteroidales	Prevotellaceae	Prevotella	***	−	+++	+++	*				−
F17.	AB270138	Bacteroidetes	Bacteroidia	Bacteroidales	Prevotellaceae	Prevotella	***	+	+++	+++	*		ii		−
F18.	AB270130	Bacteroidetes	Bacteroidia	Bacteroidales	Prevotellaceae	Prevotella	***	+	−	++	−				−
F19.	GQ327214	Bacteroidetes	Bacteroidia	Bacteroidales	Prevotellaceae	Prevotella	***	−	++	++	−				−
F20.	EU719226	Bacteroidetes	Bacteroidia	Bacteroidales	Prevotellaceae	Prevotella	***	−	++	++	−				−
F21.	AF001777	Bacteroidetes	Bacteroidia	Bacteroidales	Prevotellaceae	Prevotella	†	−	++	++	**	i	ii	i	−
F22.	AB269968	Bacteroidetes	Bacteroidia	Bacteroidales	Prevotellaceae	Prevotella	***	−	+++	+++	−				−
F23.	GU302536	Bacteroidetes	Bacteroidia	Bacteroidales	Prevotellaceae	Prevotella	***	−	++	++	*		ii		−
F24.	New.Ref.OTU	Bacteroidetes	Bacteroidia	Bacteroidales	Prevotellaceae	Prevotella	***	−	++	++	**				−
F25.	New.Ref.OTU	Bacteroidetes	Bacteroidia	Bacteroidales	Prevotellaceae	Prevotella	***	−	++	++	−				−
F26.	EU381920	Bacteroidetes	Bacteroidia	Bacteroidales	Prevotellaceae	u.b.	***	−	++	++	−				†
F27.	EU461494	Bacteroidetes	Bacteroidia	Bacteroidales	Prevotellaceae	u.b.	***	−	++	++	−				−
G1.	AB494890	Bacteroidetes	Bacteroidia	Bacteroidales	Rikenellaceae	RC9 gut group	***	+	+	+++	−				−
G2.	DQ394621	Bacteroidetes	Bacteroidia	Bacteroidales	Rikenellaceae	RC9 gut group	***	++	++	++	*	dd	dd		−
G3.	EU842535	Bacteroidetes	Bacteroidia	Bacteroidales	Rikenellaceae	RC9 gut group	*	++	++	++	−				−
G4.	GU304085	Bacteroidetes	Bacteroidia	Bacteroidales	Rikenellaceae	RC9 gut group	***	+++	++	−	−				−
G5.	AM183042	Bacteroidetes	Bacteroidia	Bacteroidales	Rikenellaceae	RC9 gut group	***	+	+	++	−				−
G6.	AB494915	Bacteroidetes	Bacteroidia	Bacteroidales	Rikenellaceae	RC9 gut group	***	−	+++	++	†		dd		−
G7.	GU302529	Bacteroidetes	Bacteroidia	Bacteroidales	Rikenellaceae	RC9 gut group	***	++	++	−	*	dd	d		−
G8.	New.Ref.OTU	Bacteroidetes	Bacteroidia	Bacteroidales	Rikenellaceae	RC9 gut group	***	−	++	++	−				−
H1.	EU470196	Bacteroidetes	Bacteroidia	Bacteroidales	S24−7	u.b.	***	+	++	++	*	dd		dd	−
H2.	EU843773	Bacteroidetes	Bacteroidia	Bacteroidales	S24−7	u.b.	***	−	−	++	−				−
I.	EU381782	Candidate division SR1	u.b.				***	++	−	+++	−				−
J1.	EU462203	Candidate division TM7	u.b.				***	−	+	++	−				−
J2.	GQ327541	Candidate division TM7	u.b.				***	−	−	++	−				−
J3.	EU474584	Candidate division TM7	u.b.				***	+++	++	++	−				−
J4.	EU381496	Candidate division TM7	u.b.				***	+	++	++	−				−
K.	GU303955	Cyanobacteria	SHA−109	u.b.			***	−	++	++	***			d	−
L1.	EF190826	Fibrobacteres	Fibrobacteria	Fibrobacterales	Fibrobacteraceae	Fibrobacter	***	−	+	++	−				−
L2.	EU381811	Fibrobacteres	Fibrobacteria	Fibrobacterales	Fibrobacteraceae	Fibrobacter	***	+	+	++	−				*
L3.	EU381936	Fibrobacteres	Fibrobacteria	Fibrobacterales	Fibrobacteraceae	Fibrobacter	***	−	+	++	−				−
M1.	EF436353	Firmicutes	Clostridia	Clostridiales	Christensenellaceae	u.b.	***	−	+++	−	−				−
M2.	AB270057	Firmicutes	Clostridia	Clostridiales	Christensenellaceae	u.b.	***	++	+++	−	−				−
M3.	AB185717	Firmicutes	Clostridia	Clostridiales	Christensenellaceae	u.b.	***	−	++	++	−				−
M4.	AB270004	Firmicutes	Clostridia	Clostridiales	Christensenellaceae	u.b.	***	+	+++	++	†				−
M5.	EU468616	Firmicutes	Clostridia	Clostridiales	Christensenellaceae	u.b.	***	−	++	−	−				−
M6.	AY854343	Firmicutes	Clostridia	Clostridiales	Christensenellaceae	u.b.	***	−	++	+	*				−
M7.	AB185553	Firmicutes	Clostridia	Clostridiales	Christensenellaceae	u.b.	***	++	+++	+++	**		d		−
M8.	AB494899	Firmicutes	Clostridia	Clostridiales	Christensenellaceae	u.b.	***	++	+++	++	−				−
M9.	AB185594	Firmicutes	Clostridia	Clostridiales	Christensenellaceae	u.b.	*	++	+	++	−				−
N1.	EU843488	Firmicutes	Clostridia	Clostridiales	Family XIII Incertae Sedis	Anaerovorax	***	+++	−	+	−				−
N2.	New.Ref.OTU	Firmicutes	Clostridia	Clostridiales	Family XIII Incertae Sedis	Incertae Sedis	***	+++	+	−	−				−
N3.	EU842492	Firmicutes	Clostridia	Clostridiales	Family XIII Incertae Sedis	Incertae Sedis	***	+++	−	−	−				−
N4.	EU842291	Firmicutes	Clostridia	Clostridiales	Family XIII Incertae Sedis	Mogibacterium	***	++	++	++	−				−
N5.	AY854273	Firmicutes	Clostridia	Clostridiales	Family XIII Incertae Sedis	Mogibacterium	***	−	++	++	−				*
N6.	FJ682205	Firmicutes	Clostridia	Clostridiales	Family XIII Incertae Sedis	Mogibacterium	***	+++	++	−	−				−
O1.	AB494822	Firmicutes	Clostridia	Clostridiales	Lachnospiraceae	Acetitomaculum	***	−	++	−	***		dd		−
O2.	AB185642	Firmicutes	Clostridia	Clostridiales	Lachnospiraceae	Acetitomaculum	***	−	+++	−	−				−
O3.	AM039826	Firmicutes	Clostridia	Clostridiales	Lachnospiraceae	Butyrivibrio	***	−	++	++	**		i	ii	−
O4.	New.Ref.OTU	Firmicutes	Clostridia	Clostridiales	Lachnospiraceae	Butyrivibrio	***	+++	−	−	***	dd			−
O5.	AB494805	Firmicutes	Clostridia	Clostridiales	Lachnospiraceae	Butyrivibrio	***	+	++	+++	−				−
O6.	AB494848	Firmicutes	Clostridia	Clostridiales	Lachnospiraceae	Butyrivibrio	***	−	++	++	−				−
O7.	EF445238	Firmicutes	Clostridia	Clostridiales	Lachnospiraceae	Butyrivibrio	***	−	+	++	−				†
O8.	AB034052	Firmicutes	Clostridia	Clostridiales	Lachnospiraceae	Butyrivibrio	***	−	+++	+++	−				−
O9.	GU303299	Firmicutes	Clostridia	Clostridiales	Lachnospiraceae	Butyrivibrio	***	+++	+	−	−				−
O10.	AB494833	Firmicutes	Clostridia	Clostridiales	Lachnospiraceae	Butyrivibrio	***	−	++	++	−				−
O11.	FJ032568	Firmicutes	Clostridia	Clostridiales	Lachnospiraceae	Butyrivibrio	***	−	+	++	**		d	d	−
O12.	EU843345	Firmicutes	Clostridia	Clostridiales	Lachnospiraceae	Butyrivibrio	***	−	++	++	−				−
O13.	GU124460	Firmicutes	Clostridia	Clostridiales	Lachnospiraceae	L. Incertae Sedis	***	−	++	++	−				*
O14.	AF001722	Firmicutes	Clostridia	Clostridiales	Lachnospiraceae	L. Incertae Sedis	***	−	++	+++	−				−
O15.	AB269976	Firmicutes	Clostridia	Clostridiales	Lachnospiraceae	L. Incertae Sedis	***	−	+	++	−				*
O16	EU381578	Firmicutes	Clostridia	Clostridiales	Lachnospiraceae	L. Incertae Sedis	***	−	+	++	*		i	ii	−
O17.	AB494761	Firmicutes	Clostridia	Clostridiales	Lachnospiraceae	L. Incertae Sedis	***	−	++	++	−				−
O18.	New.Ref.OTU	Firmicutes	Clostridia	Clostridiales	Lachnospiraceae	L. Incertae Sedis	***	−	++	++	−				−
O19.	New.Ref.OTU	Firmicutes	Clostridia	Clostridiales	Lachnospiraceae	L. Incertae Sedis	***	−	++	++	−				†
O20.	EF436345	Firmicutes	Clostridia	Clostridiales	Lachnospiraceae	L. Incertae Sedis	***	−	++	−	−				−
O21.	DQ237938	Firmicutes	Clostridia	Clostridiales	Lachnospiraceae	L. Incertae Sedis	***	−	+++	++	−				*
O22.	EF436445	Firmicutes	Clostridia	Clostridiales	Lachnospiraceae	L. Incertae Sedis	***	−	++	−	−				−
O23.	New.Ref.OTU	Firmicutes	Clostridia	Clostridiales	Lachnospiraceae	L. Incertae Sedis	***	−	++	++	−				−
O24.	GU303078	Firmicutes	Clostridia	Clostridiales	Lachnospiraceae	Oribacterium	***	−	−	++	*			ii	−
O25.	DQ085079	Firmicutes	Clostridia	Clostridiales	Lachnospiraceae	Pseudobutyrivibrio	***	−	++	++	**		ii	i	−
O26.	AB494919	Firmicutes	Clostridia	Clostridiales	Lachnospiraceae	Pseudobutyrivibrio	***	−	++	++	−				−
O27.	FJ032427	Firmicutes	Clostridia	Clostridiales	Lachnospiraceae	Roseburia	**	−	++	++	*		ii		−
O28.	EU842536	Firmicutes	Clostridia	Clostridiales	Lachnospiraceae	Roseburia	***	−	++	++	**		dd		−
O29.	AF371623	Firmicutes	Clostridia	Clostridiales	Lachnospiraceae	Roseburia	*	−	++	++	†		ii	dd	−
O30.	JF797351	Firmicutes	Clostridia	Clostridiales	Lachnospiraceae	Shuttleworthia	*	−	++	++	**		i	ii	−
O31.	AF001734	Firmicutes	Clostridia	Clostridiales	Lachnospiraceae	u.b.	***	−	−	++	−				*
O32.	EU845282	Firmicutes	Clostridia	Clostridiales	Lachnospiraceae	u.b.	***	+	++	++	−				−
O33.	EU773612	Firmicutes	Clostridia	Clostridiales	Lachnospiraceae	u.b.	***	+	++	++	−				−
O34.	EU843817	Firmicutes	Clostridia	Clostridiales	Lachnospiraceae	u.b.	***	−	++	++	−				−
O35.	EU381579	Firmicutes	Clostridia	Clostridiales	Lachnospiraceae	u.b.	***	+	++	++	**		i		***
O36.	AB270112	Firmicutes	Clostridia	Clostridiales	Lachnospiraceae	u.b.	***	−	++	++	−				−
O37.	AB494866	Firmicutes	Clostridia	Clostridiales	Lachnospiraceae	u.b.	***	−	+	++	−				−
O38.	FJ032551	Firmicutes	Clostridia	Clostridiales	Lachnospiraceae	u.b.	***	−	++	++	−				−
O39.	AY854272	Firmicutes	Clostridia	Clostridiales	Lachnospiraceae	u.b.	***	−	++	++	−				−
O40.	EU381488	Firmicutes	Clostridia	Clostridiales	Lachnospiraceae	u.b.	***	−	++	++	−				−
O41.	AF001717	Firmicutes	Clostridia	Clostridiales	Lachnospiraceae	u.b.	***	−	+	++	*			dd	**
O42.	EU719231	Firmicutes	Clostridia	Clostridiales	Lachnospiraceae	u.b.	***	−	++	++	*		d		−
O43.	AB494778	Firmicutes	Clostridia	Clostridiales	Lachnospiraceae	u.b.	***	−	++	+++	−				−
O44.	AB270116	Firmicutes	Clostridia	Clostridiales	Lachnospiraceae	u.b.	***	−	+	++	−				−
O45.	AB269996	Firmicutes	Clostridia	Clostridiales	Lachnospiraceae	u.b.	***	+++	+	−	−				−
O46.	GU304496	Firmicutes	Clostridia	Clostridiales	Lachnospiraceae	u.b.	***	+++	−	−	−				*
O47.	AB494806	Firmicutes	Clostridia	Clostridiales	Lachnospiraceae	u.b.	***	−	++	++	**		ii	ii	−
P1.	AB270001	Firmicutes	Clostridia	Clostridiales	Ruminococcaceae	Incertae Sedis	***	−	++	+	**		ii	i	**
P2.	EF686593	Firmicutes	Clostridia	Clostridiales	Ruminococcaceae	Ruminococcus	***	−	++	++	−		d		−
P3.	EF436321	Firmicutes	Clostridia	Clostridiales	Ruminococcaceae	Ruminococcus	***	−	++	+++	−				−
P4.	EU469842	Firmicutes	Clostridia	Clostridiales	Ruminococcaceae	Ruminococcus	***	−	++	++	*		ii	ii	−
P5.	AB494882	Firmicutes	Clostridia	Clostridiales	Ruminococcaceae	Ruminococcus	***	+	++	+++	−				−
P6.	EU381458	Firmicutes	Clostridia	Clostridiales	Ruminococcaceae	Ruminococcus	***	−	++	++	*		ii		−
P7.	AAQK01009861	Firmicutes	Clostridia	Clostridiales	Ruminococcaceae	Ruminococcus	***	−	+++	++	**		dd	dd	−
P8.	EU381848	Firmicutes	Clostridia	Clostridiales	Ruminococcaceae	Ruminococcus	***	+	++	++	−				†
P9.	GQ327231	Firmicutes	Clostridia	Clostridiales	Ruminococcaceae	Saccharofermentans	***	+	++	+++	†				−
P10.	EF686527	Firmicutes	Clostridia	Clostridiales	Ruminococcaceae	Saccharofermentans	***	−	++	++	−				−
P11.	AB494824	Firmicutes	Clostridia	Clostridiales	Ruminococcaceae	Saccharofermentans	***	++	++	+++	−				−
P12.	AY854346	Firmicutes	Clostridia	Clostridiales	Ruminococcaceae	Saccharofermentans	***	+	−	++	−				−
P13.	EU381703	Firmicutes	Clostridia	Clostridiales	Ruminococcaceae	Saccharofermentans	***	−	++	++	−				*
P14.	GQ327304	Firmicutes	Clostridia	Clostridiales	Ruminococcaceae	Saccharofermentans	***	−	++	++	−				−
P15.	AB034038	Firmicutes	Clostridia	Clostridiales	Ruminococcaceae	Saccharofermentans	***	−	++	++	*				−
P16.	EU468242	Firmicutes	Clostridia	Clostridiales	Ruminococcaceae	u.b.	***	−	++	++	−				−
P17.	AB494879	Firmicutes	Clostridia	Clostridiales	Ruminococcaceae	u.b.	***	+	+++	+++	−				−
P18.	EU344218	Firmicutes	Clostridia	Clostridiales	Ruminococcaceae	u.b.	***	−	++	+++	−				−
P19.	EU381706	Firmicutes	Clostridia	Clostridiales	Ruminococcaceae	u.b.	***	+	+	++	−				−
P20.	AB270149	Firmicutes	Clostridia	Clostridiales	Ruminococcaceae	u.b.	***	−	++	++	*			ii	−
P21.	EU381964	Firmicutes	Clostridia	Clostridiales	Ruminococcaceae	u.b.	***	−	+	++	−				*
P22.	AB494900	Firmicutes	Clostridia	Clostridiales	Ruminococcaceae	u.b.	***	−	−	++	*			dd	†
P23.	EU381950	Firmicutes	Clostridia	Clostridiales	Ruminococcaceae	u.b.	***	+++	++	+++	−				−
P24.	AF001762	Firmicutes	Clostridia	Clostridiales	Ruminococcaceae	u.b.	***	+++	++	++	*	dd			*
P25.	AF001761	Firmicutes	Clostridia	Clostridiales	Ruminococcaceae	u.b.	***	+	−	++	−				−
P26.	AB009186	Firmicutes	Clostridia	Clostridiales	Ruminococcaceae	u.b.	***	−	−	++	−				−
P27.	EU842742	Firmicutes	Clostridia	Clostridiales	Ruminococcaceae	u.b.	***	−	++	++	†		d	i	*
P28.	AB185556	Firmicutes	Clostridia	Clostridiales	Ruminococcaceae	u.b.	***	+	+++	+++	−				*
P29.	EU381629	Firmicutes	Clostridia	Clostridiales	Ruminococcaceae	u.b.	***	−	+	++	−				−
P30.	AY854363	Firmicutes	Clostridia	Clostridiales	Ruminococcaceae	u.b.	***	+	++	+++	−				−
P31.	AB185810	Firmicutes	Clostridia	Clostridiales	Ruminococcaceae	u.b.	*	−	++	++	−				†
P32.	DQ394677	Firmicutes	Clostridia	Clostridiales	Ruminococcaceae	u.b.	***	−	+	++	−				−
P33.	AB009189	Firmicutes	Clostridia	Clostridiales	Ruminococcaceae	u.b.	***	++	−	++	−				−
Q1.	AB009216	Firmicutes	Clostridia	Clostridiales	Veillonellaceae	Anaerovibrio	***	−	++	++	**		ii	i	−
Q2.	AB034139	Firmicutes	Clostridia	Clostridiales	Veillonellaceae	Selenomonas	***	−	++	++	**		ii	ii	−
Q3.	GQ327079	Firmicutes	Clostridia	Clostridiales	Veillonellaceae	Selenomonas	**	−	+	++	*		i	i	−
Q4.	AY244976	Firmicutes	Clostridia	Clostridiales	Veillonellaceae	Succiniclasticum	***	+++	+++	+++	*	d	dd	dd	−
Q5.	EU843672	Firmicutes	Clostridia	Clostridiales	Veillonellaceae	Succiniclasticum	***	+	−	++	−				−
R1.	AB210825	Firmicutes	Erysipelotrichia	Erysipelotrichales	Erysipelotrichaceae	Catenibacterium	*	+	++	++	**	i	ii	ii	−
R2.	FJ032444	Firmicutes	Erysipelotrichia	Erysipelotrichales	Erysipelotrichaceae	Sharpea	***	−	+	++	−				−
R3.	EU458717	Firmicutes	Erysipelotrichia	Erysipelotrichales	Erysipelotrichaceae	u.b.	***	−	+++	++	*		dd	dd	−
R4.	EU381583	Firmicutes	Erysipelotrichia	Erysipelotrichales	Erysipelotrichaceae	u.b.	***	−	++	++	−				−
R5.	EU381506	Firmicutes	Erysipelotrichia	Erysipelotrichales	Erysipelotrichaceae	u.b.	***	+++	++	++	−				−
S.	New.Ref.OTU	Proteobacteria	Betaproteobacteria	Burkholderiales	Comamonadaceae	Comamonas	***	+++	−	−	**	ii			−
T1.	EU844167	Proteobacteria	Deltaproteobacteria	Desulfobacterales	Desulfobulbaceae	Desulfobulbus	***	+++	−	−	−				†
T2.	New.Ref.OTU	Proteobacteria	Deltaproteobacteria	Desulfobacterales	Desulfobulbaceae	Desulfobulbus	***	+++	−	−	†	ii			−
T3.	GU303056	Proteobacteria	Deltaproteobacteria	Desulfobacterales	Desulfobulbaceae	Desulfobulbus	***	+++	−	−	−				−
U.	DQ174169	Proteobacteria	Epsilonproteobacteria	Campylobacterales	Campylobacteraceae	Campylobacter	***	+++	−	−	†				−
V1.	EF445274	Proteobacteria	Gammaproteobacteria	Aeromonadales	Succinivibrionaceae	u.b.	−	+	++	+	−				−
V2.	EU381934	Proteobacteria	Gammaproteobacteria	Aeromonadales	Succinivibrionaceae	u.b.	***	+	+++	+++	−				−
W.	New.Ref.OTU	Proteobacteria	Gammaproteobacteria	Cardiobacteriales	Cardiobacteriaceae	Suttonella	***	+++	−	−	**	dd			−
X1.	AB270123	Spirochaetes	Spirochaetes	Spirochaetales	Spirochaetaceae	Treponema	***	+	++	++	−				−
X2.	AF001693	Spirochaetes	Spirochaetes	Spirochaetales	Spirochaetaceae	Treponema	***	+	+++	+++	−				†
Y1.	EF445251	Tenericutes	Mollicutes	RF9	u.b.		***	+	++	++	*		dd		−
Y2.	EU381563	Tenericutes	Mollicutes	RF9	u.b.		***	+	++	++	−				−
Y3.	EU381558	Tenericutes	Mollicutes	RF9	u.b.		***	+	+	++	−				†
Y4.	AF001770	Tenericutes	Mollicutes	RF9	u.b.		***	+	−	++	*				−

a *Cows were divided into a pasture and confinement group (PG, CG, n = 5). The CG stayed on a TMR based ration during the entire trial while the PG was slowly introduced to a pasture-based ration: wk 1: TMR, wk 2: TMR and 3 h pasture/d, wk 3 and 4: TMR and 12 h pasture/d, wk 5–10: pasture and 1.75 kg DM concentrate/d. Samples of the rumen liquid- (LAAB), particle- (PAAB), and epithelium-(EAAB) associated archaea and bacteria were collected in wk 1, wk 5, and wk 10 and 16S rRNA gene amplification and sequencing was performed*.

b *Boxplots with statistics included in Data Sheet*.

c *OTU, Operational Taxonomic Unit, New.Ref. OTU, New Reference OTU*.

d *u.a., unculturable archeon, u.b., unculturable bacterium, all OTUs were classified as “unculturable bacterium or archeon” at species level, therefore only taxonomic classification up to the genus level is shown*.

e P-value: symbols indicate a significant difference in OTU abundance between locations (^***^P < 0.001, ^**^P < 0.01, ^*^P < 0.05, ^†^P < 0.10). Symbols describe the proportional abundance of the OTU at the three different locations (+++ > 1%, ++ > 0.1%, + < 0.1%, - not detected).

f Influence of the ration change from TMR to pasture on proportional abundance in the PG (comparison wk 1 and wk 10). P-value: symbols indicate a significant difference for a Group × Time or Group × Time × Location interaction (^***^P < 0.001, ^**^P < 0.01, ^*^P < 0.05, ^†^P < 0.10), dd/ ii = decrease/increase by > 2x or > 1% in proportional abundance when decreasing to/increasing from 0, d/i = a decrease/increase by < 2x or <1% in proportional abundance when decreasing to/increasing from 0.

g Symbols indicates a significant Cow or Cow × Time effect (^***^P < 0.001, ^**^P < 0.01, ^*^P < 0.05, ^†^P < 0.10).

#### Alpha diversity

**Figure 5:**
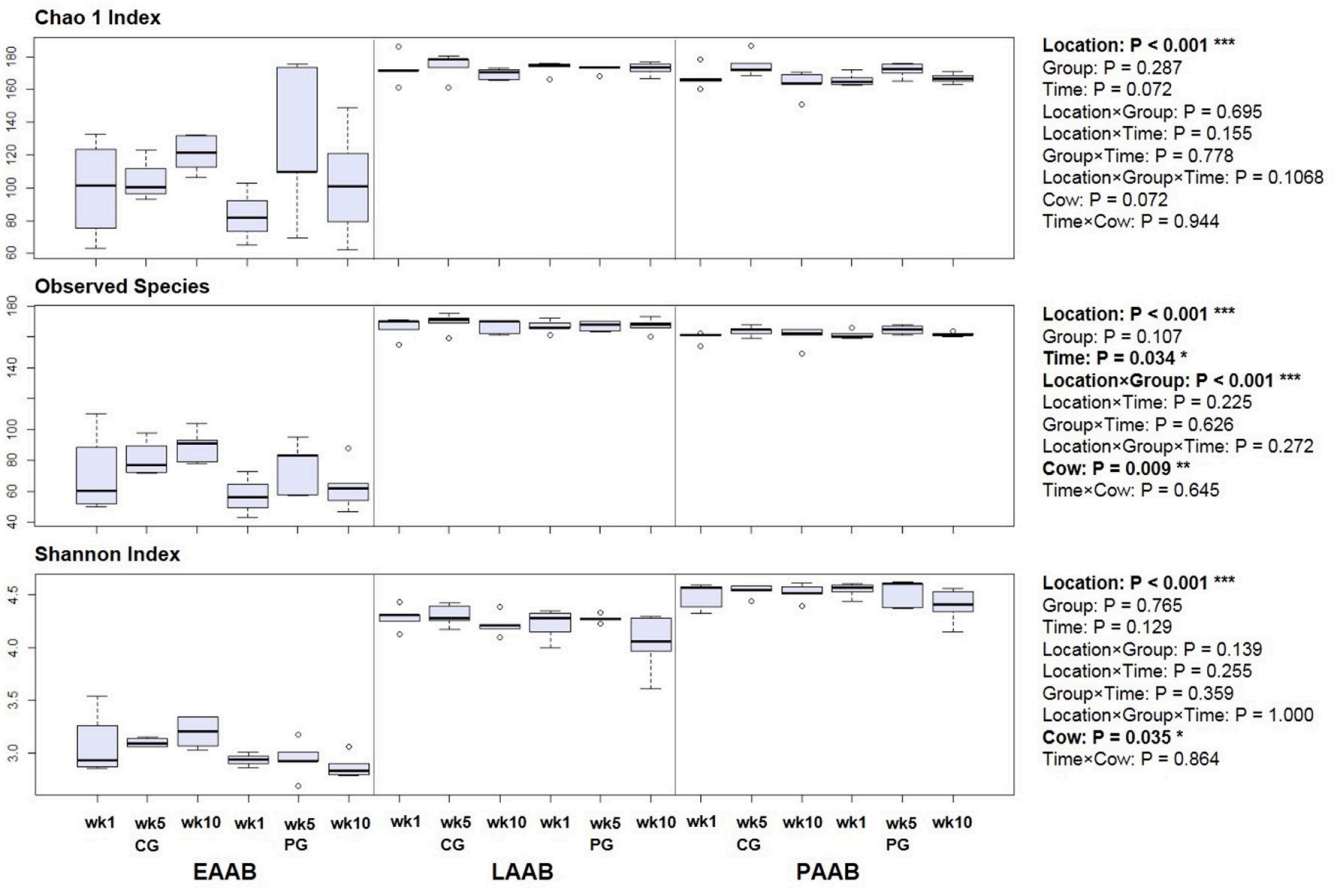
**Boxplots of diversity variables of rumen liquid (LAAB), particle (PAAB) and epithelium (EAAB) associated archaea and bacteria in wk 1, wk 5, and wk 10 of the pasture (PG) and confinement (CG, *n* = 5) group**. The CG stayed on a TMR based ration during the entire trial while the PG was slowly introduced to a pasture-based ration: wk 1: TMR, wk 2: TMR and 3 h pasture/d, wk 3 and 4: TMR and 12 h pasture/d, wk 5-10: pasture and 1.75 kg DM concentrate/d.

Alpha diversity analysis revealed a lower chao1 and Shannon index as well as lower observed species count in the EAAB compared to the LAAB and PAAB with an average of 73 [30; median (IQR)] compared to 169 (6) and 162 (4) observed species (*P* < 0.001), a chao1 index of 108 (33) compared to 173 (5) and 168 (7) (*P* < 0.001), and a Shannon index of 3.0 (0.2) compared to 4.3 (0.2) and 4.6 (0.2) (*P* < 0.001), respectively (Figure 5). The LAAB further exhibited a higher observed species count (*P* < 0.001) as well as chao1 index (*P* = 0.004) compared to the PAAB, whereas the PAAB had a higher Shannon index (*P* < 0.001). In the PG a significantly lower observed species count in the EAAB compared to the CG was observed (*P* = 0.035). Further, no significant treatment effects were observed. In two diversity variables (observed species and Shannon index) a significant Cow effect was observed.

#### Beta diversity

**Figure 6:**
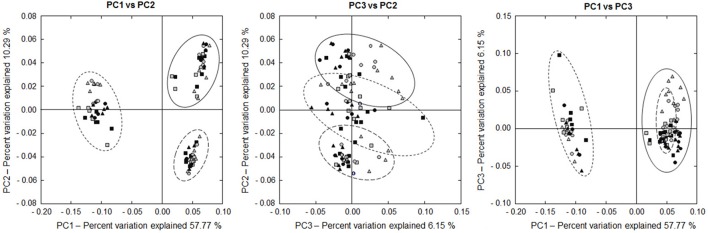
**Two dimensional PCO plots of 16S rRNA sequencing results (based on weighted UniFrac distances) of rumen liquid (LAAB, solid line), particle (PAAB, large dashed line), and epithelium (EAAB, small dashed line) associated archaea and bacteria in wk 1 (square), wk 5 (round), and wk 10 (triangle) of the pasture (PG, gray) and confinement (CG, black, *n* = 5) group**. The CG stayed on a TMR based ration during the entire trial while the PG was slowly introduced to a pasture-based ration: wk 1: TMR, wk 2: TMR and 3 h pasture/d, wk 3 and 4: TMR and 12 h pasture/d, wk 5–10: pasture and 1.75 kg DM concentrate/d.

**Figure 7:**
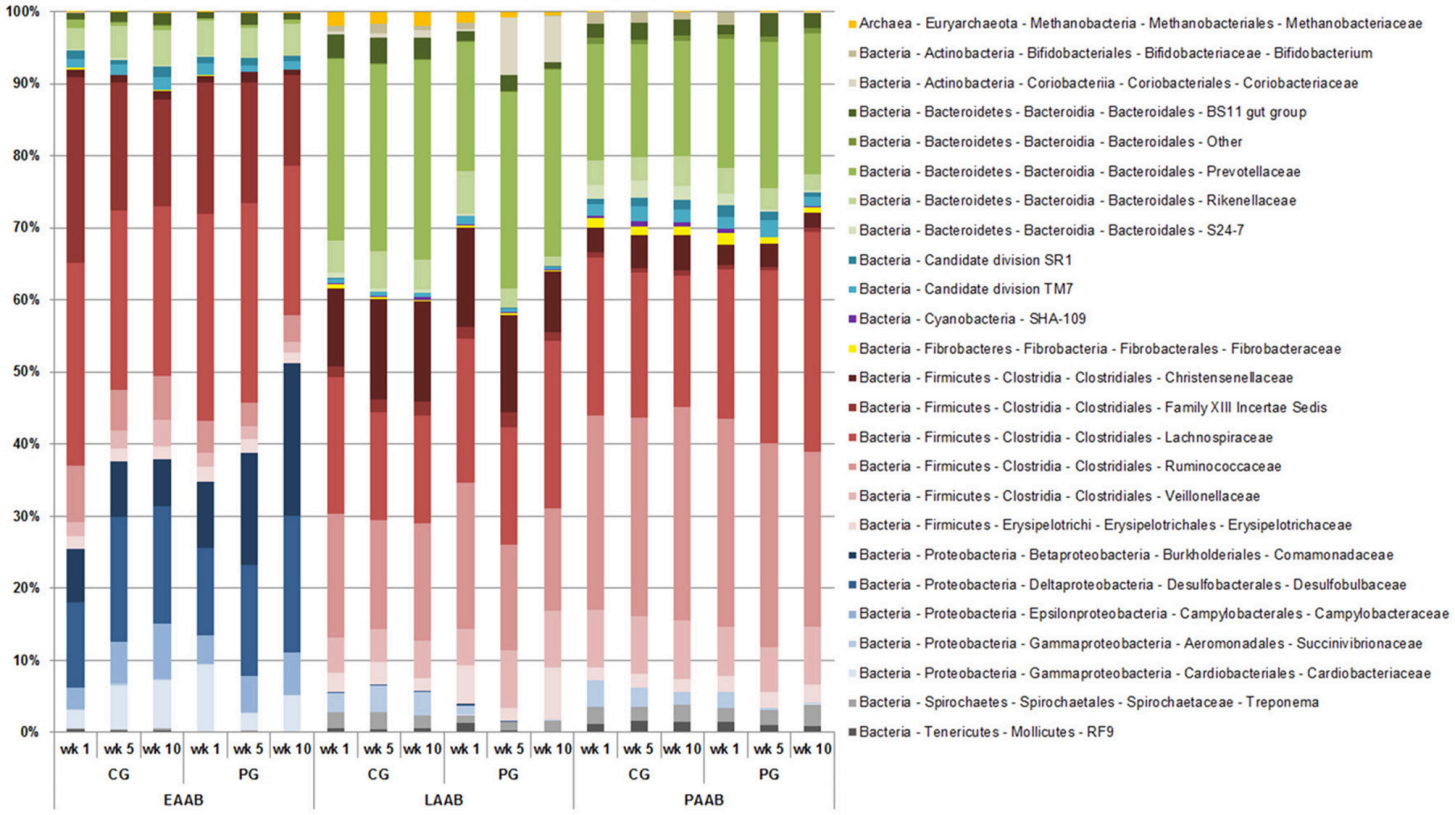
**Differences in relative abundances (expressed as percentages) of OTUs derived from 16S rRNA gene sequencing on family level of rumen liquid (LAAB), particle (PAAB), and epithelium (EAAB) associated archaea and bacteria families in wk 1, wk 5, and wk 10 of the pasture (PG) and confinement (CG, *n* = 5) group**. The CG stayed on a TMR based ration during the entire trial while the PG was slowly introduced to a pasture-based ration: wk 1: TMR, wk 2: TMR and 3 h pasture/d, wk 3 and 4: TMR and 12 h pasture/d, wk 5–10: pasture and 1.75 kg DM concentrate/d.

Beta diversity analysis revealed a significant Location (*P* < 0.001), Group (*P* < 0.001), Time (*P* = 0.035) and Cow (*P* < 0.001) effect, as well as a significant Location × Group (*P* = 0.011) and Group × Time (*P* = 0.036) interaction. PCO plots show a clustering of the LAAB, PAAB, and EAAB, with a higher similarity between LAAB and PAAB, compared to the EAAB samples (Figure 6). Taxonomic classification at family level showed broad differences in community composition between the LAAB, PAAB, and EAAB (Figure 7). In the LAAB members of the Prevotellaceae (25%), Lachnospiraceae (18%), Ruminococcaeceae (16%), Christensenellaceae (12%), Veillonellaceae (6%), Rikenellaceae (4%), Erysipelotrichaceae (4%), Coriobacteriaceae (3%), and the uncultured BS11 gut group (Bacteroidales, 2%) contributed to 90% of the relative abundance of 16S rRNA genes. In the PAAB a similar pattern was observed with members of the Ruminococcaceae (28%), Lachnospiraceae (23%), Prevotellaceae (18%), Veillonellaceae (8%), Christensenellaceae (4%), Rikenellaceae (3%), Spirochaetaceae (2%), uncultured BS11 gut group (2%), Erysipelotrichaceae (2%), and Succinivibrionaceae (2%) accounting for 90% of the relative abundance, whereas in the group of the EAAB members of the families Lachnospiraceae (26%), Family XIII Incertae Sedis (Clostridiales, 18%), Desulfobulbaceae (15%), Cardiobacteriaceae (5%), Comamonadaceae (11%), Campylobacteraceae (5%), Ruminococcaceae (5%), and Rikenellaceae (4%) were the dominant community members.

Statistical analysis on OTU level showed a significant location effect (i.e., LAAB, PAAB, and EAAB) for all 177 OTUs, except for two members of *Prevotella* genus and one member of Succinivibrionaceae family (Table 1 and Data Sheet 1). OTUs within particular families either exhibited a similar distribution pattern (Bifidobacteriaceae, Coriobacteriaceae, Desulfobulbaceae, Succinivibrionaceae, and Spirochaetaceae), a generally similar distribution pattern with few exceptions [*Prevotella*, Fibrobacteraceae, and members of the order RF9 (Mollicutes)] or a very diverse distribution pattern among the three locations [uncultured BS11 gut group (Bacteroidales), uncultured RC9 gut group (Rikenellaceae), uncultured S24-7 (Bacteroidales), members of the Candidate division TM7 phylum, Christensenellaceae, Family XIII Incertae Sedis (Clostridiales), Lachnospiraceae, Ruminococcaceae, Veillonellaceae, and Erysipelotrichales]. Analysis on OTU level also revealed that the predominant role of the family Lachnospiraceae in the EAAB can be attributed to mainly four OTUs (two of the genus *Butyrivibrio* and two further unclassified OTUs).

Similar to the location, also for the effect of the ration change from TMR to pasture in the PG variable trends within taxonomic groups were observed (Table 1). In some taxa either a decrease [Bifidobacteriaceae, uncultured RC9 gut group (Rikenellaceae), uncultured S24-7 (Bacteroidales), uncultured SHA-109 (Cyanobacteria), Christensenellaceae, Acetitomaculum (Lachnospiraceae), Succiniclasticum (Veillonellaceae), further unclassified members of the Erysipelotrichaceae, *Suttonella* (Cardiobacteriaceae), and uncultured RF9 (Mollicutes)], an increase of at least one member species [*Atopobium, Incertae Sedis (*Lachnospiraceae*), Oribacterium*, Pseudobutyrivibrio, Shuttleworthia, *Incertae Sedis* (Ruminococcaceae), *Anaerovibrio, Selenomonas, Catenibacterium, Comamonas*, and *Desulfobulbus*], or no alterations [*Methanobrevibacter*, uncultured BS11 gut group (Bacteroidales), uncultured Candidate division SR1, and TM7, *Fibrobacter*, uncultured Family XIII Incertae Sedis (Clostridiales), Saccharofermentans, *Sharpea, Campylobacter*, Succinivibrionaceae, and *Treponema*] in proportional abundances were observed, whereas others exhibited mixed effects (Prevotellaceae, *Butyrivibrio*, and *Roseburia, Ruminococcus* and other further unclassified Ruminococcaceae).

For most OTUs that showed an increase or decrease in proportional abundance over the course of the trial in the PG, a difference between wk 1, wk 5, and wk 10 was observed, mostly exhibiting a gradual increase or decrease over the three points in time (C1-3, F1, F17, F21, F23, G6, K, O1, O3, O16, O24, O25, O27, O28, O30, O35, O41, O42, O47, P1, P2, P4, P7, P20, P22, Q2, Q3, Q4, R1, S, and T2 in Table 1 and Data Sheet 1). A few OTUs, however, exhibited a V-shaped evolution over the course of wk 1, wk 5, and wk 10 (M7, O29, O42, P6, Q1, R3, W, and Y1). And for a few OTUs that differed in abundance between wk 1 and wk 10 in the PG, no alterations between wk 5 and wk 10 were observed (B3, G2, G7, H1, O4, O11, P4, P24, P27, Q1, Q2, Q4, and R3).

In the OTUs where a group effect was observed, the proportional abundance was altered at either all (B3, C1-C3, F21, G7, H1, K, O1, O3, O4, O11, O16, O24, O25, O27, O29, O30, O47, P1, P4, P7, P22, P27, Q1–4, R1, R3, S, T2, and W in Table 1 and Data Sheet 1) or only at a part of the locations (F1, F17, F23, G2, G6, M7, O28, O35, O41, O42, P2, P6, P20, P24, and Y1) where the OTUs exhibited a significant abundance (Table 1).

In case of 28 OTUs a significant or a trend for a Cow or Cow × Time effect was observed. To illustrate the alterations in proportional abundance of each species on cow level, plots including this aspect have been added to the appendix (Data Sheet 2).

## Discussion

### General differences between the LAAB, PAAB, and EAAB

-The DNA fingerprinting as well as amplicon sequencing approach revealed distinct differences between the three locations, with a higher similarity between the PAAB and LAAB, compared to the EAAB. This is in line with an earlier study using PCR-DGGE (Sadet et al., 2007) and can be explained by the close spatial relationship and constant interchange between the two communities due to the constant ongoing fiber colonization and degradation. As described in previous publications members of the Prevotellaceae constituted the most dominant family in the LAAB (Kong et al., 2010; Pitta et al., 2010; Singh et al., 2015), whereas in the PAAB a higher abundance of members of taxa were found that are associated with fiber digestion such as Ruminococcaceae and *Fibrobacter* (Koike and Kobayashi, 2009). This was to be expected, since a key role of the PAAB in the degradation of fiber can be assumed (Kong et al., 2010; McCann et al., 2014a). When analyzing the distribution across the three locations of the individual OTUs, no clear pattern at a higher level than species was observed for most taxa. Some species in a given taxa were detected in all or two communities, whereas others could exclusively be found in one. This finding illustrates that observed effects on phylum, class, order, family, or genus level do not necessarily account for all of its member species, demonstrating that microbes that are phylogenetically close may still exhibit substantially different phenotypes and functions (Morgavi et al., 2013). This discrepancy between taxonomic classification (or genomic commonality) and phenotype has been reported and criticized earlier and can most likely be attributed to differences in gene expression as a result of environmental influences (Achenbach and Coates, 2000; Kampfer and Glaeser, 2012). Weimer (2015) notes that the genetic capability of different degradative functions may reside within a single bacterial strain, but that it is dependent on the presence of potential competitors and symbionts if a particular degradative capability is carried out. Kampfer and Glaeser (2012) therefore suggest revising the polyphasic approach (integration of genotype and phenotype) in prokaryotic taxonomy. Regarding rumen microbiota research, different authors have noted that future studies should focus on the characterization of the functional properties of the rumen microbial ecosystem, aside the different microbial species (Morgavi et al., 2013).

When comparing our results with different studies comparing the LAAB and PAAB, we observed similar as well as different results concerning the abundance of different taxa (Cho et al., 2006; Brulc et al., 2009; Kong et al., 2010; Pei et al., 2010; Pitta et al., 2010; de Menezes et al., 2011; Kim and Yu, 2012; Singh et al., 2015). Further, similar to our results de Menezes et al. (2011) have shown a higher species diversity in the LAAB compared to the PAAB, whereas the results of Kong et al. (2010), Pitta et al. (2010), and Sadet et al. (2007) show the opposite. We suggest that these observed differences among studies can be ascribed to differences in ration composition (Henderson et al., 2015), time the animals received the ration prior to sampling (Hackmann, 2015), number of animals sampled (Weimer, 2015), sample collection (Li et al., 2009), microorganism and DNA isolation (Henderson et al., 2013), microbiota analysis method (DNA fingerprinting vs. amplicon sequencing, Sadet et al., 2007), sequencing platform and depth (Klindworth et al., 2013, also discussed in Schären et al., 2017).

In the EAAB we observed a much less diverse microbiota, with very different species compared to the PAAB and LAAB, which is in line with other studies (Cho et al., 2006; Sadet et al., 2007). Our results are fairly similar to the observations made by Petri et al. (2013) with the Lachnospiraceae, uncultured Family XIII Incertae Sedis (Clostridiales), Ruminococcaceae, Prevotellaceae, Desulfobulbaceae, Erysipelotrichaceae, and Rikenellaceae constituting the core microbiota of the EAAB in their trial. These findings illustrate a mixture of Gram-positive and Gram-negative bacteria, which is in contrast to the findings of older culture- (Wallace et al., 1979) and electron-microscopy-based studies (McCowan et al., 1978; Cheng et al., 1979), describing a mainly Gram-positive community. Earlier studies described the EAAB as being possibly associated with fermentation end-products, VFA absorption, oxygen consumption, urea digestion and initiated breakdown of dead epithelial tissue (Cheng et al., 1979; Wallace et al., 1979; McCann et al., 2014a). The hypothesis of an oxygen scavenging function of the EAAB is supported by the finding that some of the OTUs, that were detected in our trial as being mainly or only present in the EAAB, have been assigned to taxa that were earlier described as being aerobic (Erysipelotrichaceae, *Comamonas*, and *Suttonella*) or microaerophilic (*Campylobacter*) (Garrity et al., 2006; Vos et al., 2009). Further, a part of the OTUs have been assigned to taxa that have been described as being asaccharolytic (*Campylobacter* and *Mogibacterium*), nitrate reducing (*Comamonas* and *Campylobacter*), complex organic compound degrading (*Comamonas*), putrescine fermenting (*Anaerovorax*), and sulfur compounds reducing (*Desulfobulbus*) (Garrity et al., 2006; Vos et al., 2009). Further research involving cultivation-independent techniques is needed to elucidate the relevance of these functional properties in the rumen and the interrelations between the different microbial species and their host.

### Effects of the ration change from TMR to pasture

The DNA fingerprinting as well as the beta-diversity analysis of the amplicon sequencing approach showed that at all three locations the microbiota was significantly influenced by the ration change. However, the hypothesis that the EAAB remain more consistent throughout dietary changes (Sadet et al., 2007; McCann et al., 2014a) was not confirmed. This result is opposite to the findings of Sadet-Bourgeteau et al. (2010) illustrating only minor alterations in the EAAB using a DNA-fingerprinting (PCR-DGGE) method in a trial involving wethers that were consecutively fed forage and different mixed concentrate forage diets. The authors however admitted that this method may not be sensitive enough to detect subtler changes in the community (the PCR-DGGE technique has an relative abundance limit of 1%, it is therefore likely that alterations in less abundant taxa are underestimated Sadet et al., 2007). Contrary to this study, applying a more severe dietary influence, Petri et al. (2013) observed significant alterations in various taxa of the EAAB in a trial involving the transition from a forage to a high grain diet, an acidosis-challenge, and a recovery period, which is in line with our results. Additionally, an earlier study by McCowan et al. (1980) has shown that the distribution pattern of the epithelium adherent bacterial population is diet dependent. This aspect should be included in future studies.

Both analysis techniques (DNA fingerprinting and amplicon sequencing) showed that even though the animals in the PG were already on a full-grazing ration for 4–6 days in wk 5, the microbiota at all three locations was significantly different from that in wk 10. During this trial we also analyzed the ruminal protozoal counts on a weekly basis and observed a gradual increase in holotrich protozoa concentrations from wk 5 on Künzel et al. (2016). After wk 7 a plateau was observed, suggesting also an adaptation in this period. These findings further agree with the observations made on animal level. We observed alterations in different production, metabolic and rumen variables, all pointing toward a decreased rumen fermentation activity in the first weeks on a full-grazing ration due to a decreased DMI. Thereafter, most likely due to a behavioral and metabolic adaptation, DMI and rumen fermentation activity increased again, causing a decrease in energy deficit and stabilization of various variables in wk 8–10 of the trial (Schären et al., 2016a,b). Taken together this data illustrates that the adaption of the cow's rumen microbiota and metabolism to a pasture-based ration most likely required 2–3 weeks in our trial. This is in line with a study of Nakano et al. (2013) showing a stabilization of the rumen microbiota of steers 28 days after being switched onto a full-grazing ration. However, in future trials weekly or even daily sampling should be involved to monitor microbial changes in the rumen upon a ration change more closely and to investigate the delay with which metabolic and production variables follow alterations in the rumen microbiota.

Similar to de Menezes et al. (2011) and Nakano et al. (2013) we observed an increase in most OTUs assigned to *Prevotella* when cows were transitioned to a pasture-based ration. It has been suggested that members of *Prevotella* grow rapidly whenever readily fermentable carbohydrates are available (Tajima et al., 2001; Bekele et al., 2010; Pitta et al., 2010). Since fresh grass contains high amounts of water-soluble carbohydrates this could explain their increase in relative abundance. Further, de Menezes et al. (2011) hypothesized whether the increased propionate production on the pasture-based diet was related to the increased abundance of Prevotellaceae and Veillonellaceae. Also in our trial we observed a lower acetate proportion in wk 9 and 10 as well as lower acetate/propionate ratio in wk 9 in the PG (Schären et al., 2016b), along with an increase of these two taxa, supporting this hypothesis. However, we did not observe several alterations described by these other two studies, such as a higher relative abundance of the Fibrobacteraceae on a TMR-based ration, an increase in the abundance of the Erysipelotrichaceae in the LAAB and the Lachnospiraceae in the PAAB, no alterations in the rumen protozoal community (de Menezes et al., 2011) or an increase in OTUs assigned to the *Butyrivibrio* species (Nakano et al., 2013). Similar to the location effect we suggest that the differences between studies can be attributed to the different rations fed and the time the animals received the rations prior to sampling (14 and 28 d in study of de Menezes et al., 2011 and Nakano et al., 2013) as well as methodological aspects. Further, most studies so far summarized the effects on a higher taxonomic level than the species, possibly mingling effects in some cases. Our results have shown that the location as well as treatment effect can either be very similar throughout member species of a taxa, or exhibit opposite trends. This is in line with a study of Bekele et al. (2010) suggesting the existence of diet-specific members of *Prevotella*. Future studies should include this aspect by further characterizing the different member species and differentiating functional and taxonomic interrelations.

In the current study a filtering step was applied, in which all OTUs with a relative abundance of <0.1% were excluded. This was done to guarantee a solid differentiation between artifact and true organism. This however also implies that alterations in low abundant species (members of the so called “rare biosphere”) were not captured. It is generally acknowledged that the more dominant species most likely contribute to the key functions in rumen fermentation (Henderson et al., 2015). However, only little is known on the function and relevance of the low abundance members and future studies should involve their identification and functional characterization (Morgavi et al., 2013). Further, due to the different physical properties of the samples three different DNA extraction methods were used Henderson et al. (2013) have shown that depending on the method applied, the abundance of different taxa varies. The authors for example describe an increase in the abundance of the Bacteroidetes phylum and a concurrent decrease in the Firmicutes phylum, when a non-mechanical lysis procedure is used. It was suggested that this can be attributed to their cell wall constitution (Gram-negative vs. Gram-positive). In our data, however, no apparent divergence toward the phylum Bacteroidetes was observed in the samples treated with a non-mechanical procedure (PAAB and EAAB samples). Henderson et al. (2013) also describe an increase in the Fibrobacteres in non-mechanical DNA extraction methods. In our study, we observed a higher abundance of Fibrobacteres in the PAAB, compared to the LAAB, indicating a possible influence of the DNA extraction method in this context. However, as described above, these findings are also in line with the literature, describing the Fibrobacteres as fiber digesting bacteria. We therefore conclude that the possibility of a certain bias due to the different DNA extraction methods applied cannot fully be excluded, and that future studies should involve more uniform DNA extraction methods whenever possible, but its implications might be neglectable in this case. Further, the main focus of this manuscripts lies on the alterations in the three different communities over time. Since the comparisons are performed within sample types, these results are not affected by the different DNA extraction protocols.

### The effect of the individual cow

Different studies have shown that the cow itself as an individual has a significant influence on its rumen microbiota, most likely through behavioral and physiological processes, such as rumination, salivation, absorption, and passage of VFA in the rumen, thereby controlling the ruminal chemistry (Sadet-Bourgeteau et al., 2010; de Menezes et al., 2011; Petri et al., 2013; Weimer, 2015). Several of these effects were also confirmed in our trial. The alterations over time in the PG compared to the CG would only emerge properly in the SSCP gels, when samples were compared to their own reference sample collected in wk 1 from the same cow. In the alpha-diversity analysis of the amplicon sequencing data, a significant Cow effect was observed, illustrating that certain cows seem to possess a more diverse rumen microbiota than others. Further, the beta-diversity analysis revealed a significant Cow or Cow × Time effect in 16% of OTUs. Several recent studies illustrate that the rumen microbiota of dairy cows and steers can be linked to different phenotypic characteristics such as milk production and composition (Jami et al., 2014; Lima et al., 2015), feed efficiency (Guan et al., 2008; Zhou et al., 2009, 2010; Hernandez-Sanabria et al., 2010, 2012; Carberry et al., 2012; Rius et al., 2012; McCann et al., 2014b; Myer et al., 2015), and breed (Guan et al., 2008). These and our results suggest that acquired animal behavior through environmental conditions as well as genetics may play a role in the rumen microbiota composition (Henderson et al., 2015).

The significant Cow effect, as well as the finding that a large part of the detected OTUs at all three locations remained unaltered in their abundance upon the ration change, are in line with the generally acknowledged assumption that the rumen microbiota consists of a core and a variable microbiota, but that individual taxa abundances may vary greatly across diets and animals (Jami and Mizrahi, 2012; Wu et al., 2012; Henderson et al., 2015).

In summary, our data illustrated that the LAAB, PAAB, and EAAB are three distinct prokaryote communities, differing in species diversity and composition. The LAAB and PAAB exhibit a higher species diversity and similarity, compared to the EAAB. Where the latter can most likely be attributed to the constant interchange between the two communities due to the ongoing fiber colonization and degradation. Many bacteria species found in the EAAB have earlier been described as possessing functional properties in culture, of which their relevance in rumen fermentation and metabolism is yet to be elucidated. The ration change from TMR to pasture influenced the microbial composition in all three locations significantly, contrary to the earlier stated hypothesis that the EAAB remain more consistent throughout dietary changes. Our data further illustrates that the time for adaptation from TMR to pasture most likely requires several days to weeks. However, future studies should include more frequent sampling. Further, the hypothesis that the rumen microbiota consists of a core and a variable microbiota, exhibiting a strong host influence was confirmed, but future studies should include the description of rare prokaryote species as well. For the effect of location as well as the ration change either very similar or opposite trends among member species of common taxa were observed. This finding highlights the importance of functional aside genomic characterization, and supports earlier studies suggesting that the genotype as well as phenotype should be included in taxonomic classification (polyphasic approach).

## Author contributions

Project acquisition, UM, SD, GB; Trial and project design, MS, UM, SD, GB; Trial implementation and sample collection, MS; Sample Analysis (DNA extraction, PCR-SSCP Analysis), MS, KK, SR; Data Analysis (Statistics and Graphics), MS, MG, SR; Data Interpretation, MS, SR, MG, JH, TU, GB, UM, SD; Writing of manuscript, MS; Revision of manuscript, MS, KK, SR, MG, UM, JH, TU, GB, SD.

### Conflict of interest statement

The authors declare that the research was conducted in the absence of any commercial or financial relationships that could be construed as a potential conflict of interest.

## Supplementary material

The sequencing data has been submitted at the European Nucleotide Archive (ENA) under the following accession number: PRJEB19414 and can be accessed using following link: http://www.ebi.ac.uk/ena/data/view/PRJEB19414.

## Abbreviations

TMR: total mixed ration
VFA: volatile fatty acid
PG: pasture group
CG: confinement group
LAB: liquid associated bacteria
PAB: particle associated bacteria
EAB: epithelium associated bacteria
LAAB: liquid associated archaea and bacteria
PAAB: particle associated archaea and bacteria
EAAB: epithelium associated archaea and bacteria.
