# Supplementary material for: Alterations in the Rumen Liquid-, Particle- and Epithelium-Associated Microbiota of Dairy Cows during the Transition from a Silage- and Concentrate-Based Ration to Pasture in Spring
Source: Front Microbiol. 2017 May 2;8:744. doi: 10.3389/fmicb.2017.00744 (PMC5411454; doi:10.3389/fmicb.2017.00744)
Supplement: Supplementary file 1 [file DataSheet1.PDF]

A.

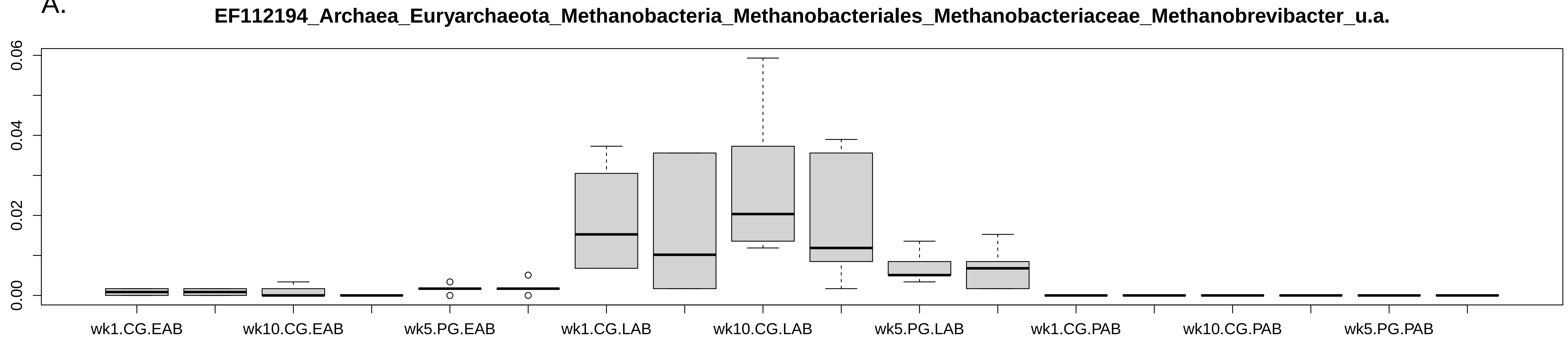

Statistics (p−values): Location: < 0.001; Group: 0.087; L×G: 0.031; Time: 0.560; L×T:0.562; G×T: 0.595; L×G×T: 0.230; Cow: 1.000; T×C: 1.000.

B1.

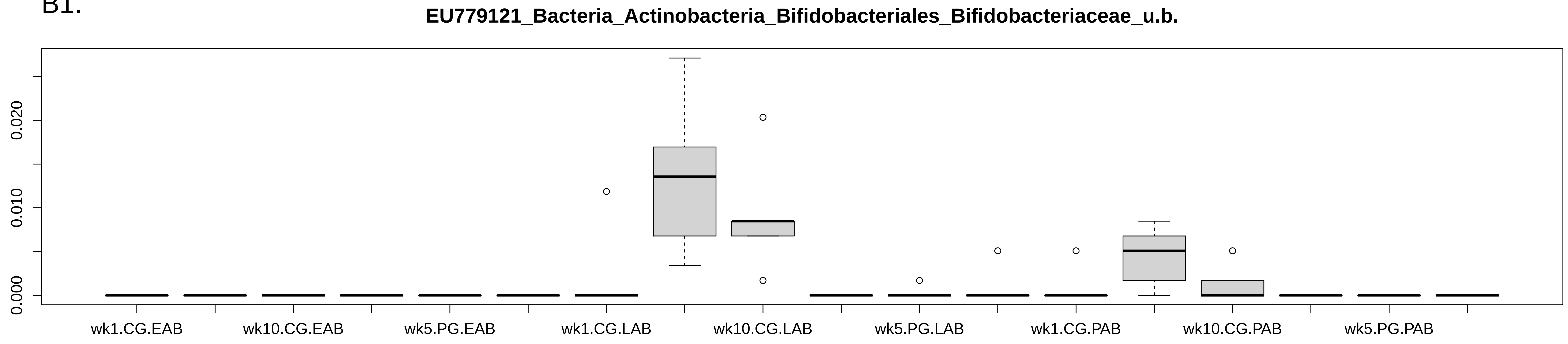

Statistics (p−values): Location: < 0.001; Group: < 0.001; L×G: < 0.001; Time: 0.016; L×T:0.058; G×T: 0.031; L×G×T: 0.162; Cow: 0.455; T×C: 0.154.

B2.

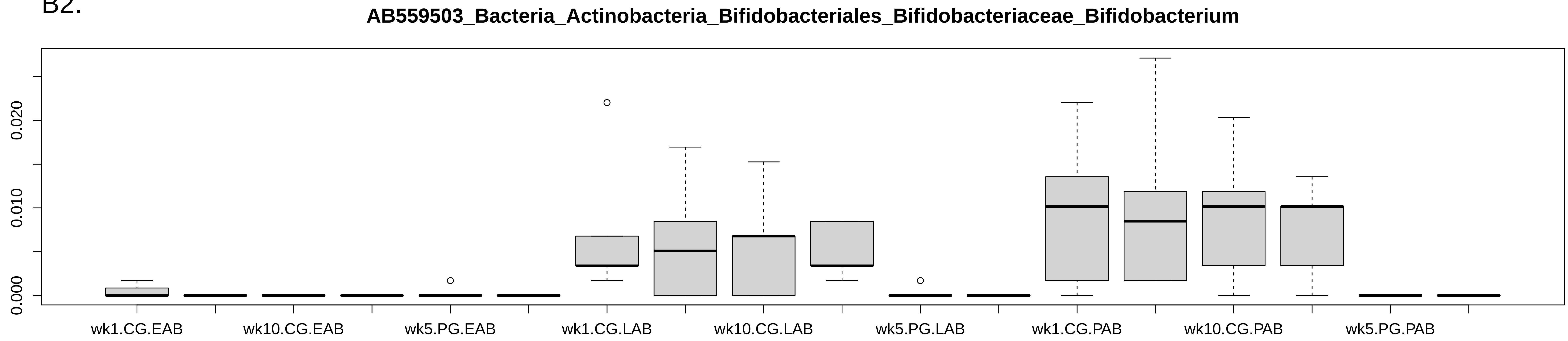

Statistics (p−values): Location: < 0.001; Group: < 0.001; L×G: 0.037; Time: 0.084; L×T:0.698; G×T: 0.270; L×G×T: 1.000; Cow: 0.032; T×C: 1.000.

B3.

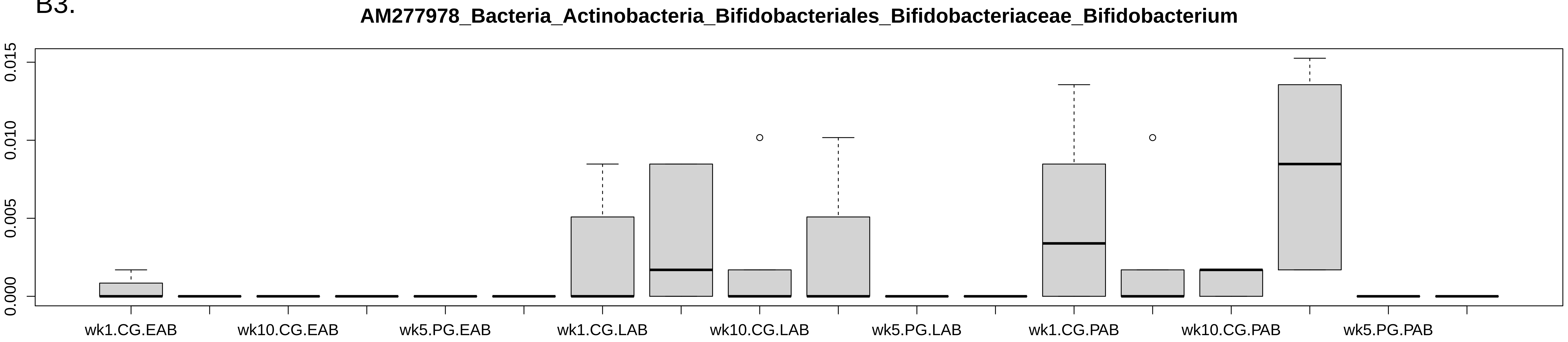

Statistics (p−values): Location: 0.008; Group: 0.286; L×G: 0.418; Time: 0.005; L×T:0.034; G×T: 0.098; L×G×T: 0.824; Cow: 0.041; T×C: 0.046.

C1.

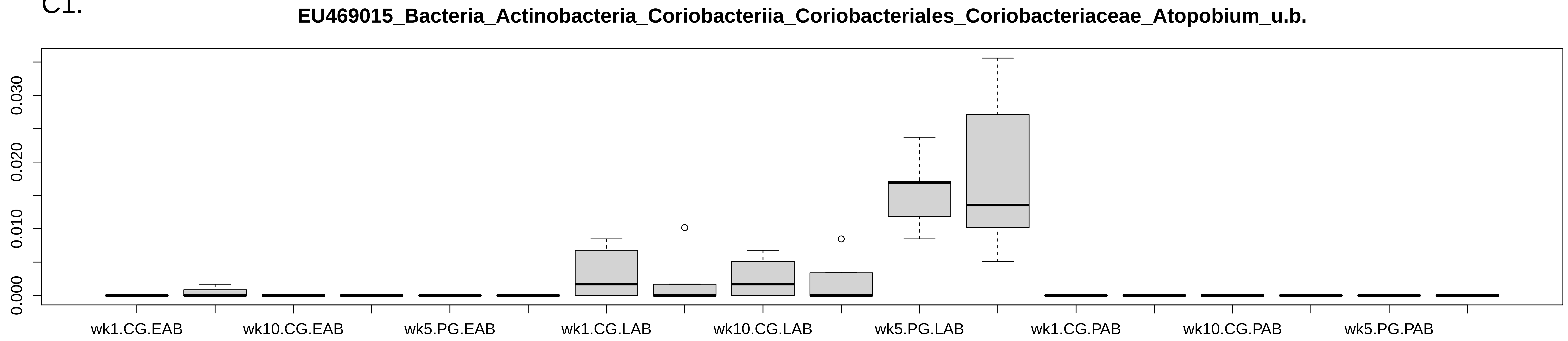

Statistics (p−values): Location: < 0.001; Group: < 0.001; L×G: < 0.001; Time: 0.030; L×T:0.001; G×T: 0.023; L×G×T: < 0.001; Cow: 0.765; T×C: 0.042.

C2.

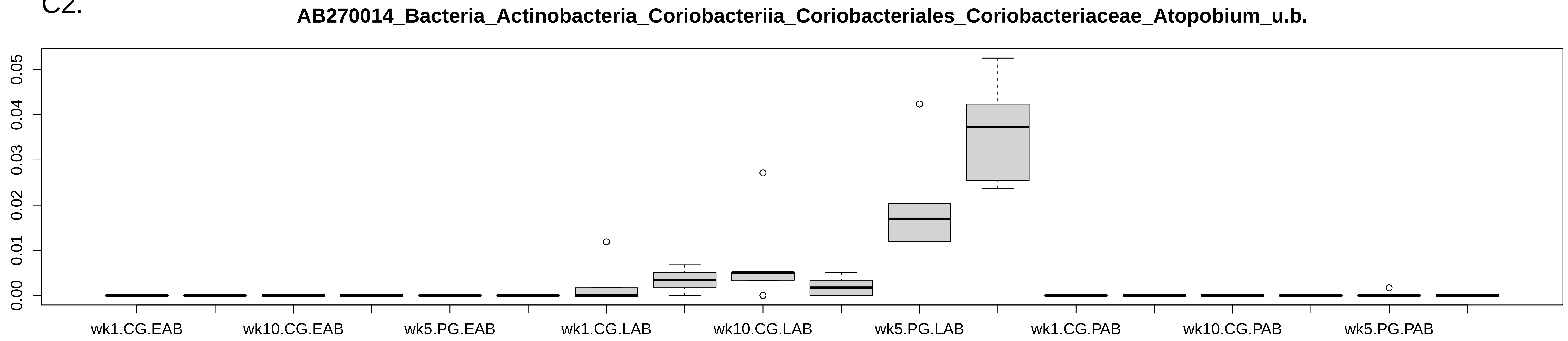

Statistics (p−values): Location: < 0.001; Group: < 0.001; L×G: < 0.001; Time: < 0.001; L×T:< 0.001; G×T: 0.008; L×G×T: < 0.001; Cow: 0.804; T×C: 0.113.

C3.

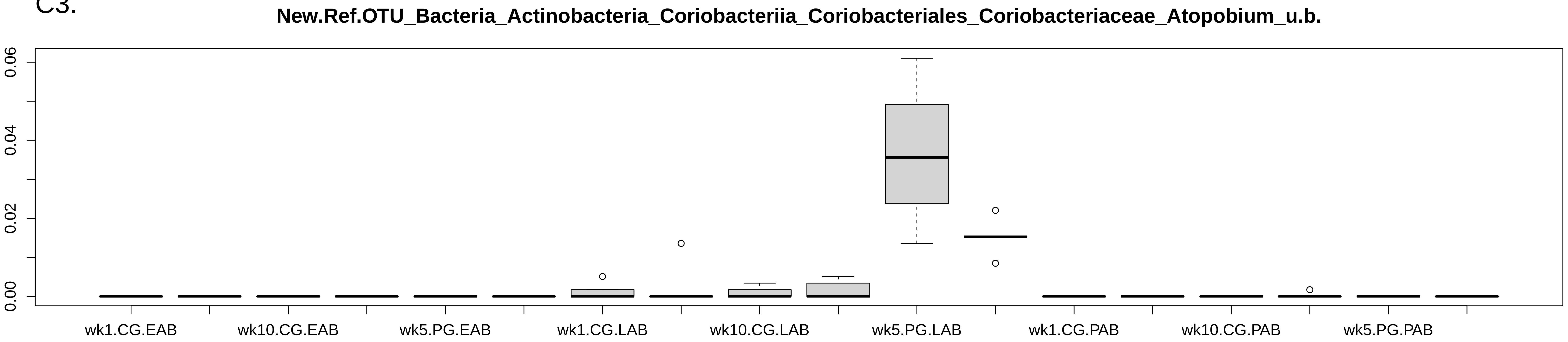

Statistics (p−values): Location: < 0.001; Group: < 0.001; L×G: < 0.001; Time: < 0.001; L×T:< 0.001; G×T: < 0.001; L×G×T: < 0.001; Cow: 0.351; T×C: 0.553.

D.

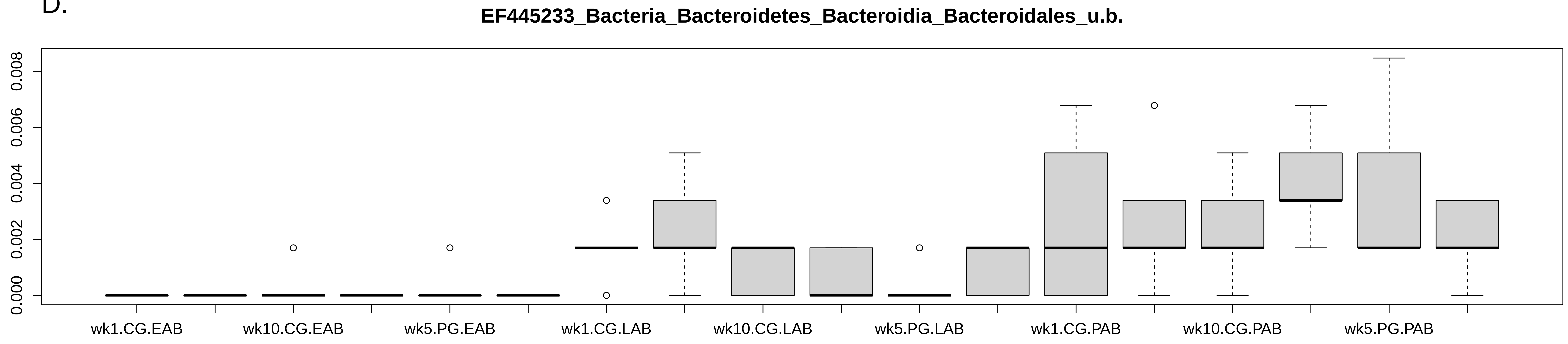

Statistics (p−values): Location: < 0.001; Group: 0.784; L×G: 0.094; Time: 0.502; L×T:0.780; G×T: 1.000; L×G×T: 0.480; Cow: 0.280; T×C: 0.444.

E1.

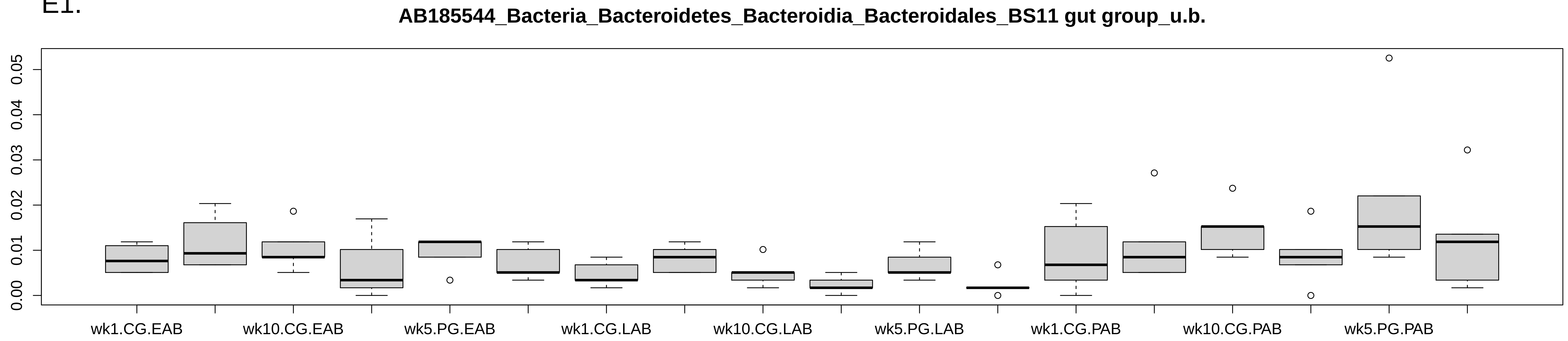

Statistics (p−values): Location: < 0.001; Group: 0.882; L×G: 0.535; Time: 0.049; L×T:0.864; G×T: 0.186; L×G×T: 0.674; Cow: 0.359; T×C: 0.135.

E2.

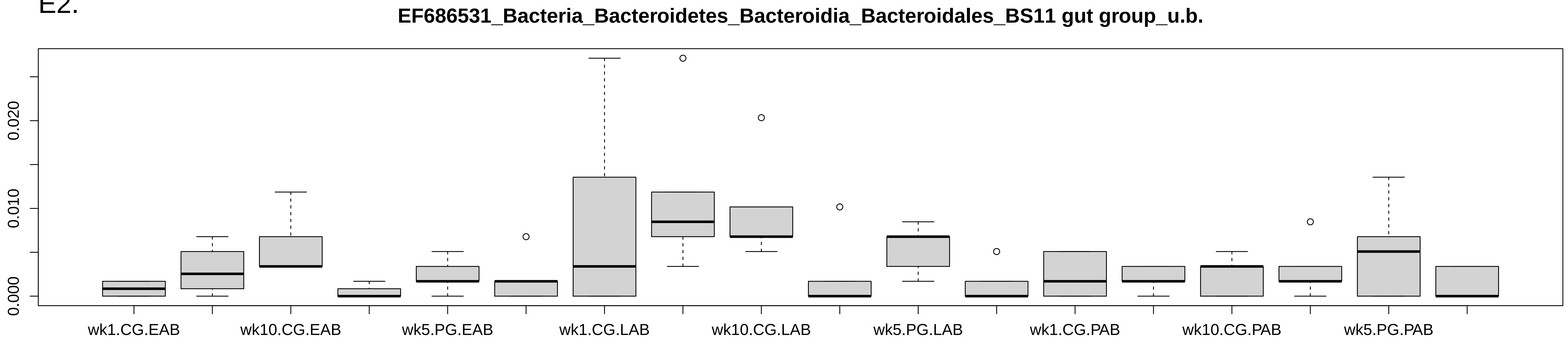

Statistics (p−values): Location: 0.001; Group: 0.008; L×G: 0.008; Time: 0.363; L×T:0.672; G×T: 0.483; L×G×T: 1.000; Cow: 0.241; T×C: 0.980.

E3.

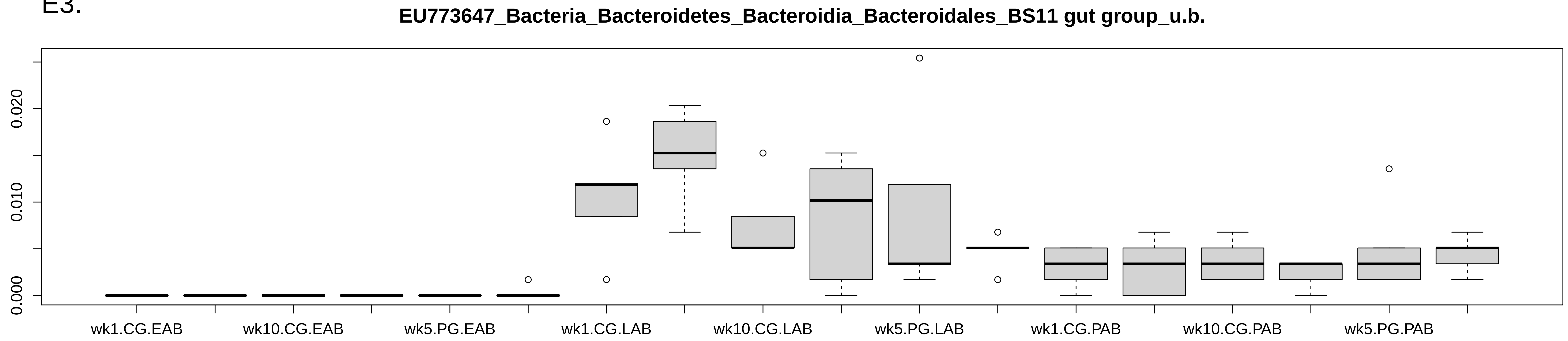

Statistics (p−values): Location: < 0.001; Group: 0.581; L×G: 0.141; Time: 0.122; L×T:0.122; G×T: 1.000; L×G×T: 0.842; Cow: 0.941; T×C: 0.109.

E4.

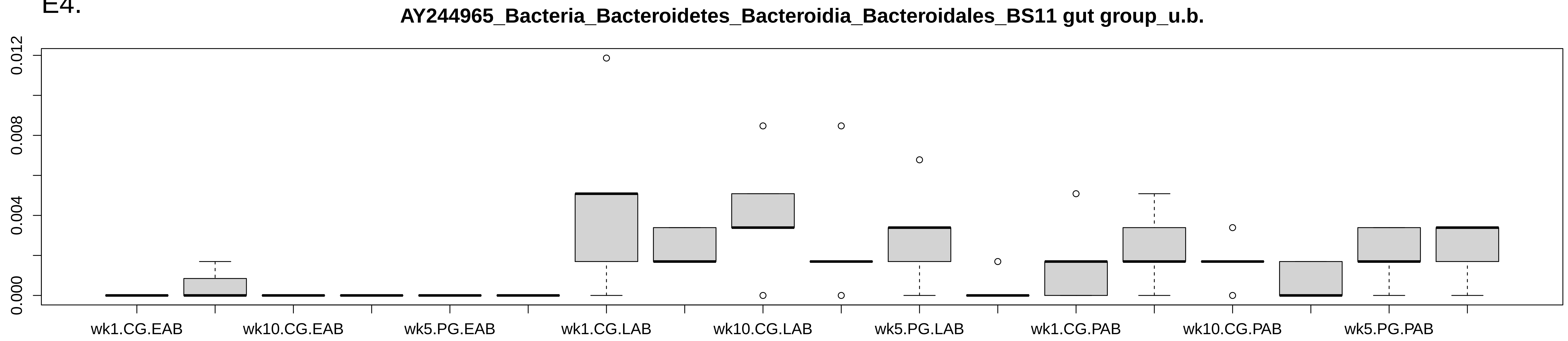

Statistics (p−values): Location: < 0.001; Group: 0.353; L×G: 0.194; Time: 0.682; L×T:0.373; G×T: 0.356; L×G×T: 0.194; Cow: 0.039; T×C: 0.780.

F1.

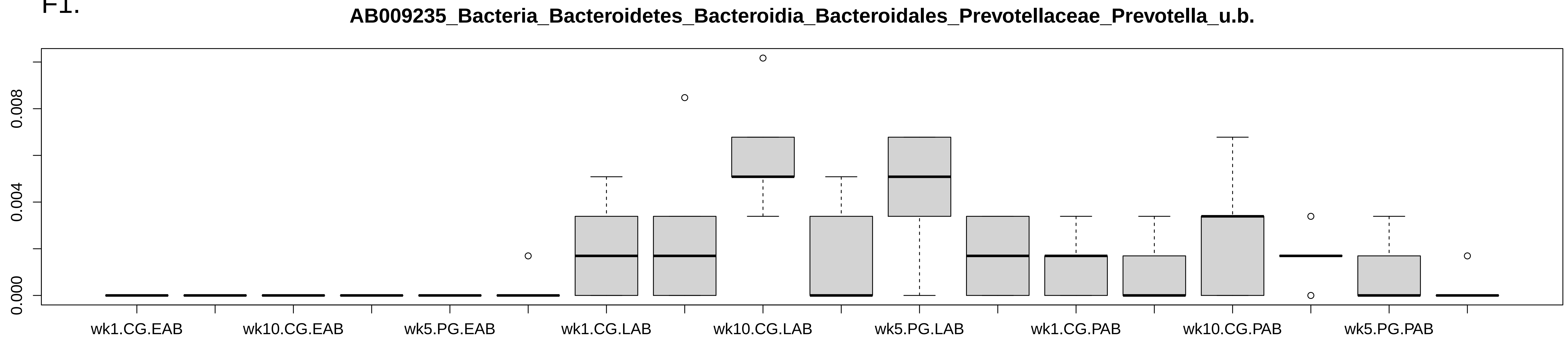

Statistics (p−values): Location: < 0.001; Group: 0.205; L×G: 0.652; Time: 0.190; L×T:0.265; G×T: 0.014; L×G×T: 0.085; Cow: 0.603; T×C: 0.363.

F2.

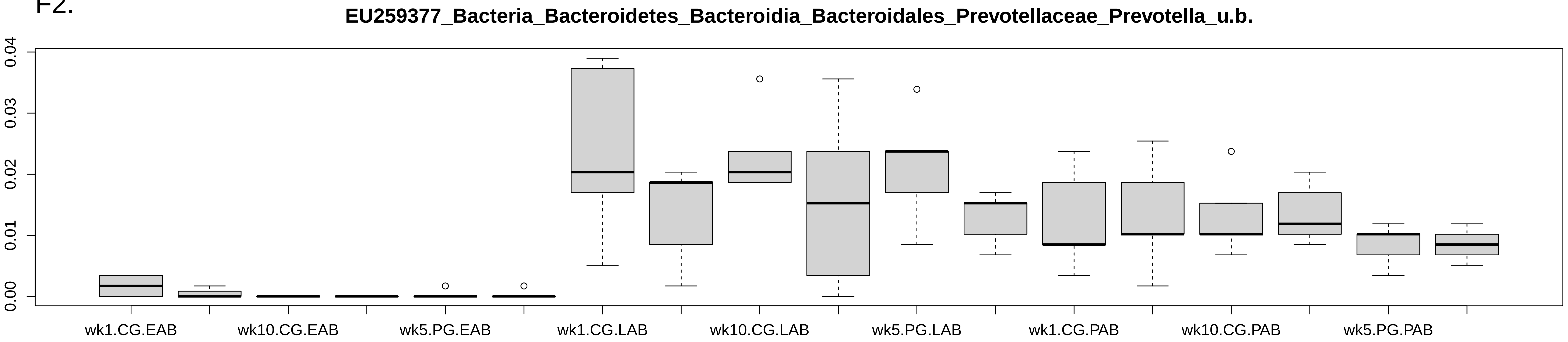

Statistics (p−values): Location: < 0.001; Group: 0.097; L×G: 0.730; Time: 0.980; L×T:1.000; G×T: 0.289; L×G×T: 0.156; Cow: 0.495; T×C: 1.000.

F3.

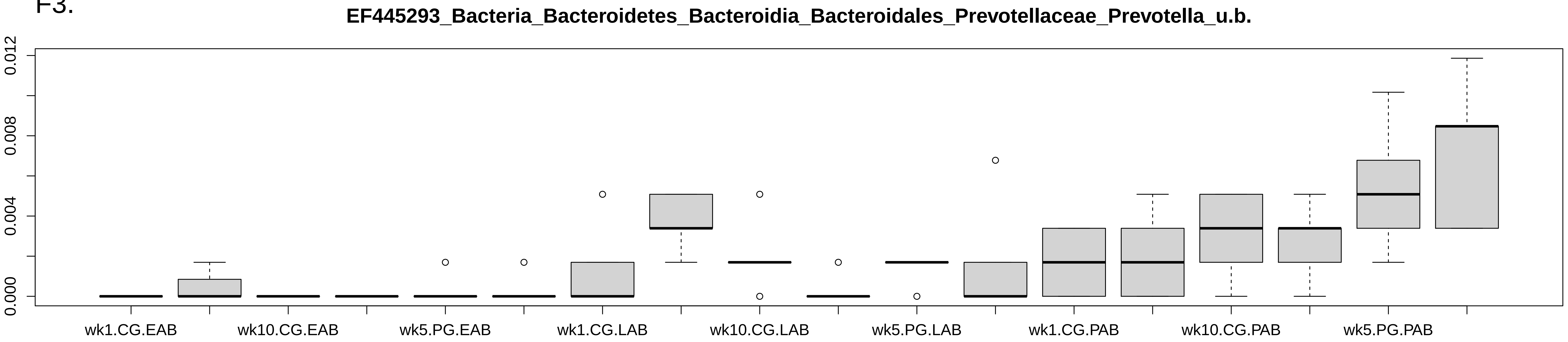

Statistics (p−values): Location: < 0.001; Group: 0.745; L×G: < 0.001; Time: 0.031; L×T:0.228; G×T: 0.332; L×G×T: 0.540; Cow: 1.000; T×C: 0.318.

F4.

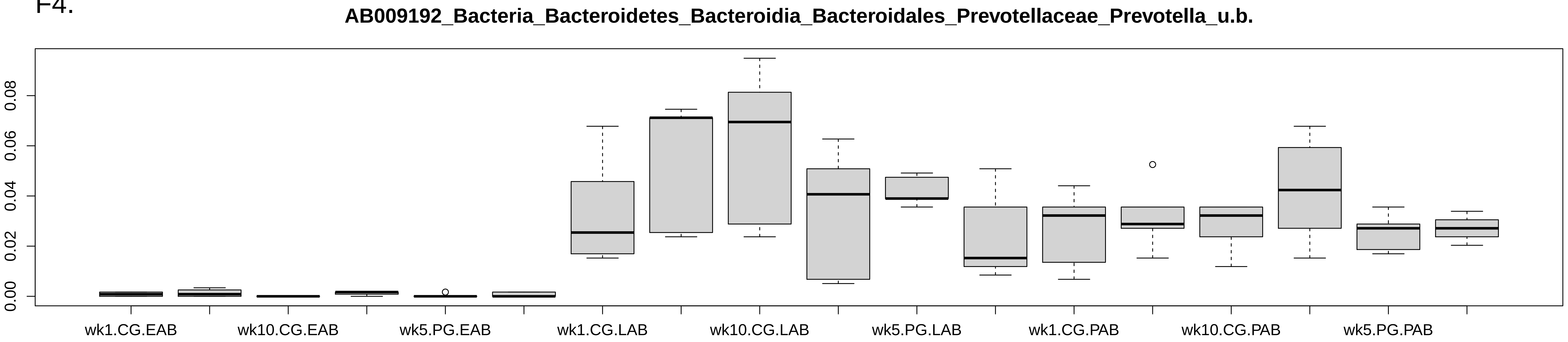

Statistics (p−values): Location: < 0.001; Group: 0.261; L×G: 0.068; Time: 0.693; L×T:0.461; G×T: 0.182; L×G×T: 0.490; Cow: 0.843; T×C: 0.961.

F5.

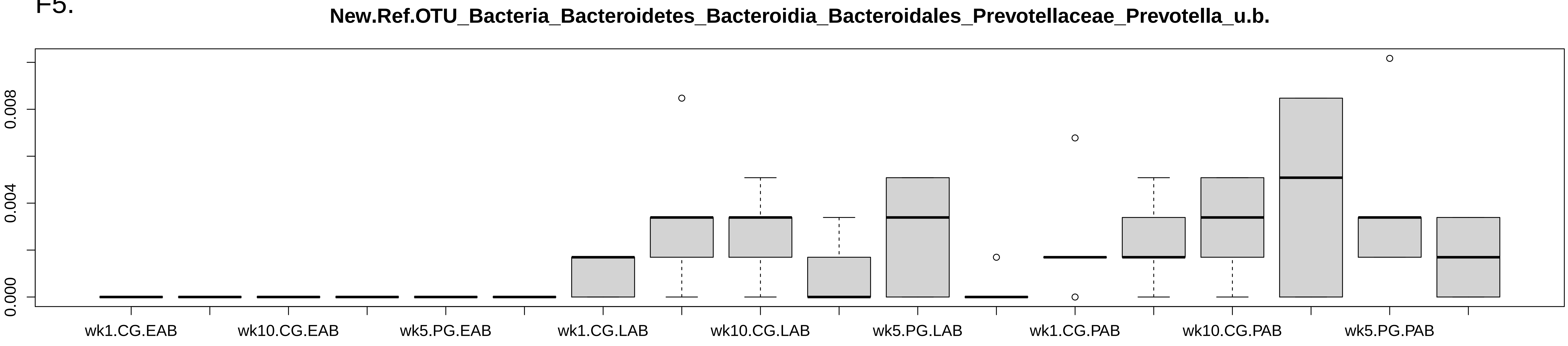

Statistics (p−values): Location: < 0.001; Group: 0.726; L×G: 0.413; Time: 0.440; L×T:0.502; G×T: 0.307; L×G×T: 0.640; Cow: 1.000; T×C: 0.462.

F6.

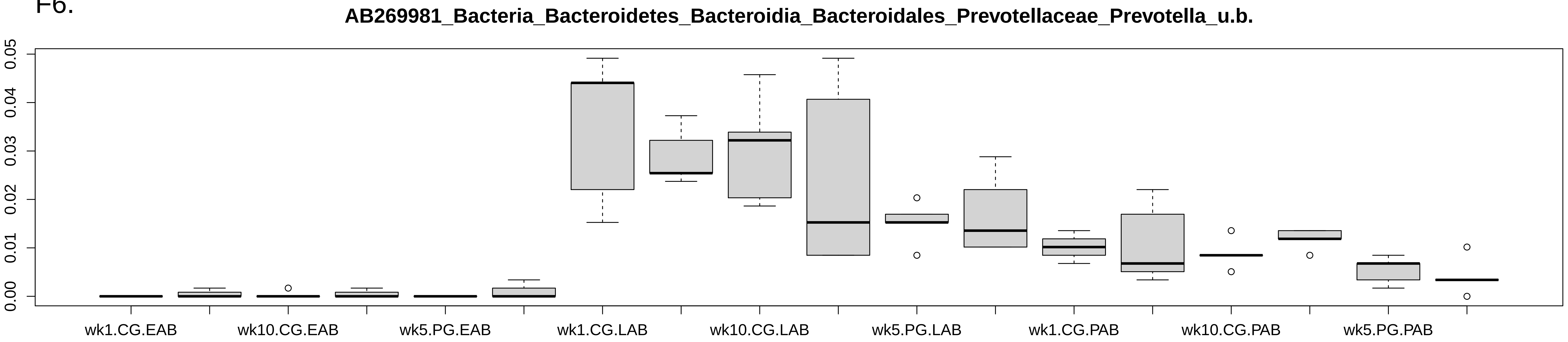

Statistics (p−values): Location: < 0.001; Group: 0.000; L×G: 0.008; Time: 0.379; L×T:0.639; G×T: 1.000; L×G×T: 0.938; Cow: 1.000; T×C: 0.311.

F7.

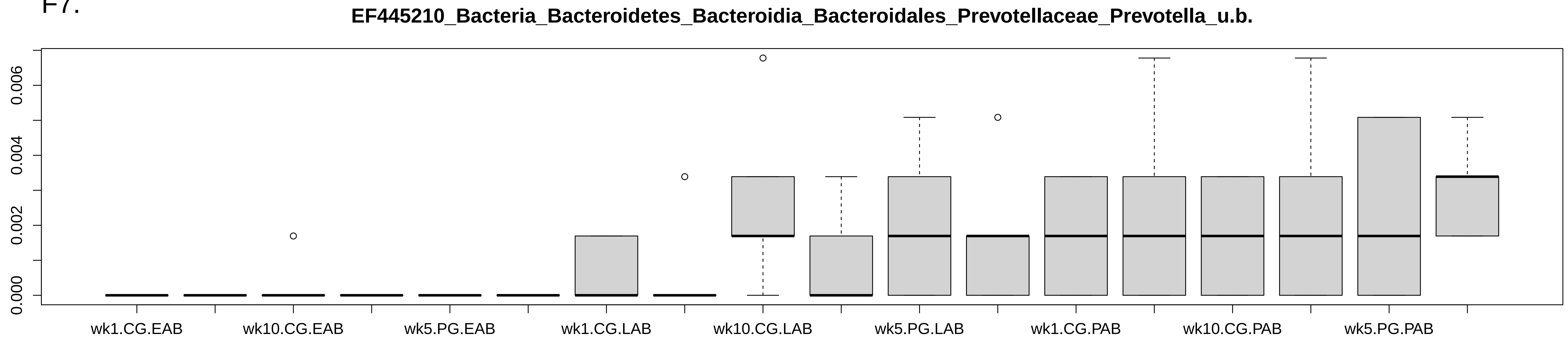

Statistics (p−values): Location: < 0.001; Group: 0.623; L×G: 0.655; Time: 0.795; L×T:0.862; G×T: 0.978; L×G×T: 0.663; Cow: 1.000; T×C: 0.498.

F8.

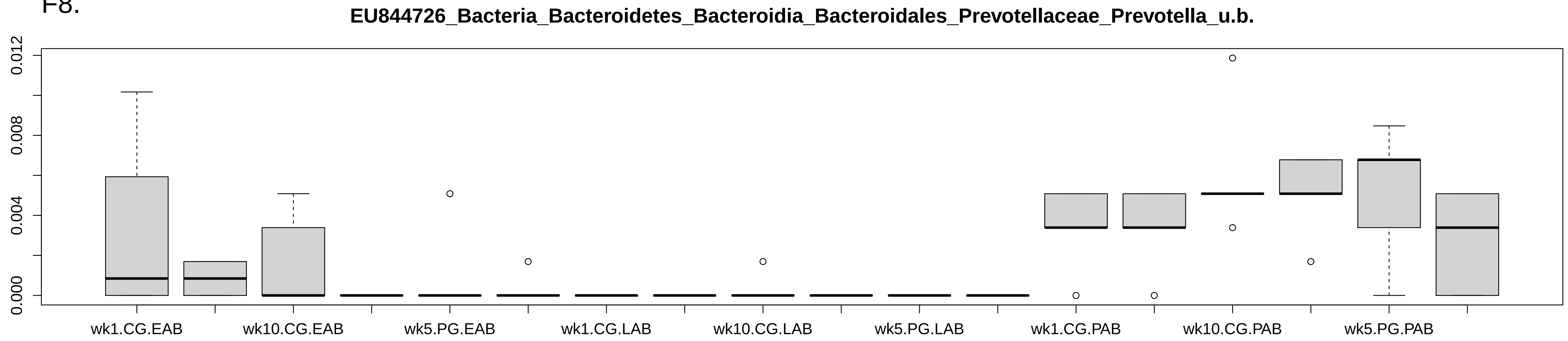

Statistics (p−values): Location: < 0.001; Group: 0.232; L×G: 0.287; Time: 1.000; L×T:1.000; G×T: 0.108; L×G×T: 0.176; Cow: 0.130; T×C: 1.000.

F9.

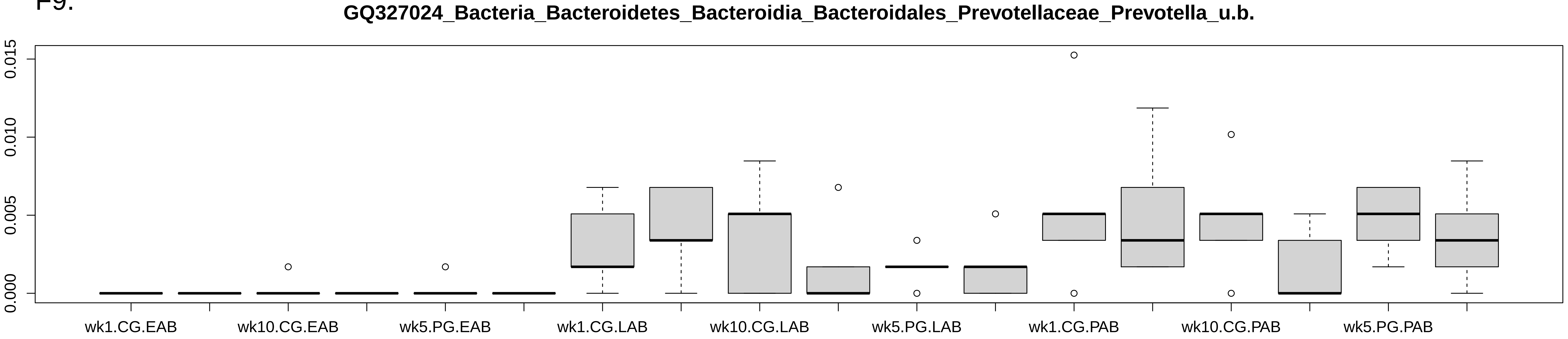

Statistics (p−values): Location: < 0.001; Group: 0.077; L×G: 0.705; Time: 0.668; L×T:1.000; G×T: 0.882; L×G×T: 0.441; Cow: 0.192; T×C: 0.088.

F10.

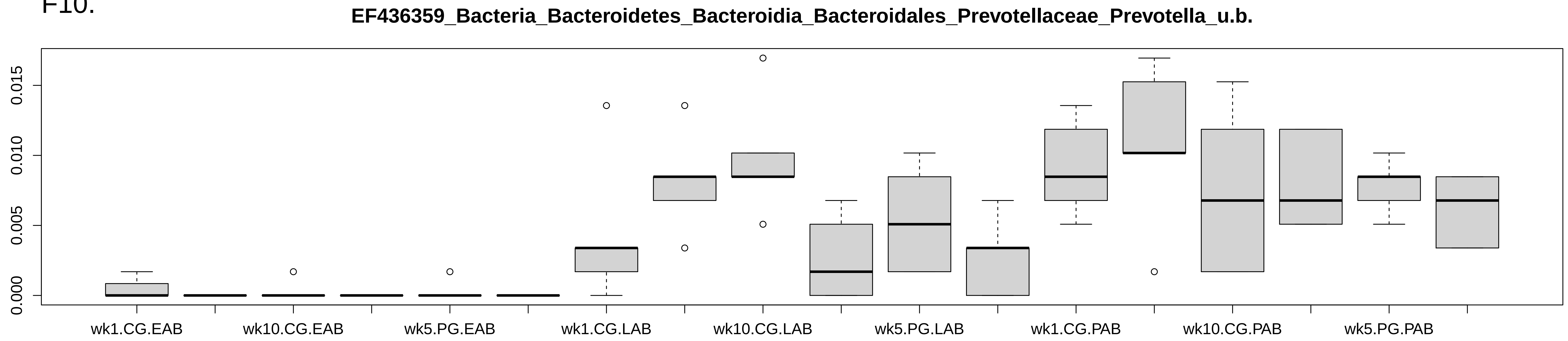

Statistics (p−values): Location: < 0.001; Group: 0.025; L×G: 0.080; Time: 0.395; L×T:0.314; G×T: 0.517; L×G×T: 0.656; Cow: 0.667; T×C: 0.941.

F11.

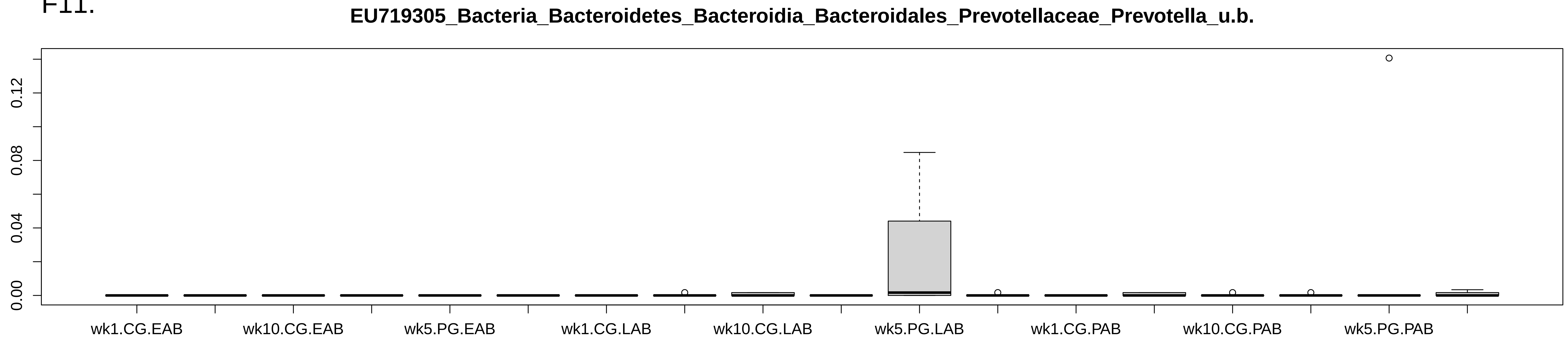

Statistics (p−values): Location: 0.580; Group: 0.116; L×G: 0.840; Time: 0.098; L×T:0.775; G×T: 0.109; L×G×T: 0.897; Cow: 0.745; T×C: 0.380.

F12.

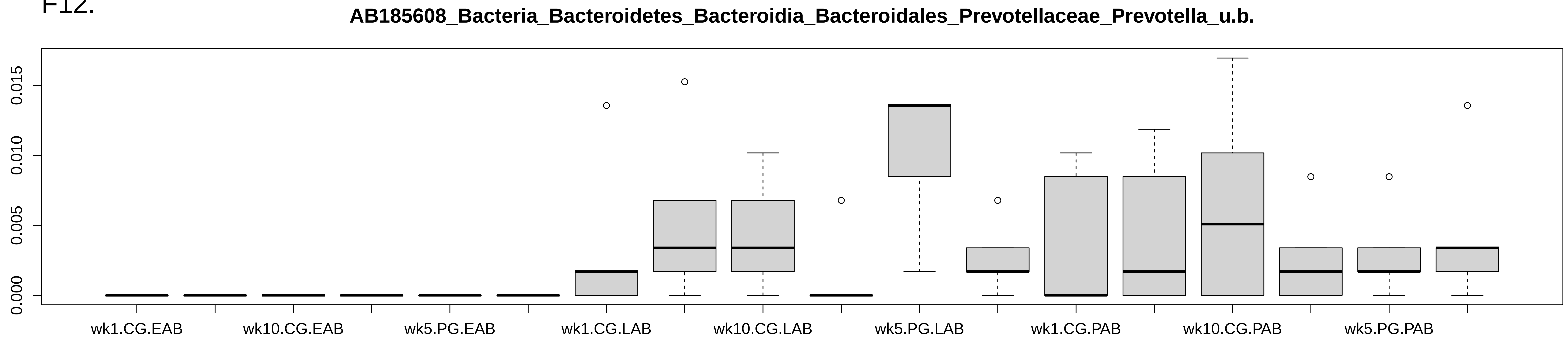

Statistics (p−values): Location: < 0.001; Group: 0.882; L×G: 0.794; Time: 0.173; L×T:0.146; G×T: 0.344; L×G×T: 0.560; Cow: 1.000; T×C: 0.981.

F13.

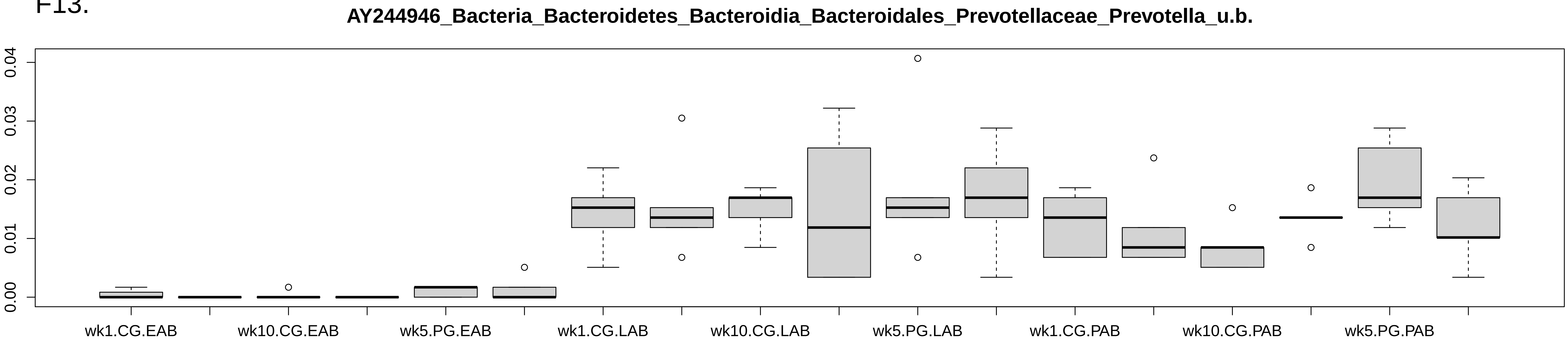

Statistics (p−values): Location: < 0.001; Group: 0.922; L×G: 0.961; Time: 0.379; L×T:0.944; G×T: 0.537; L×G×T: 1.000; Cow: 0.169; T×C: 0.142.

F14.

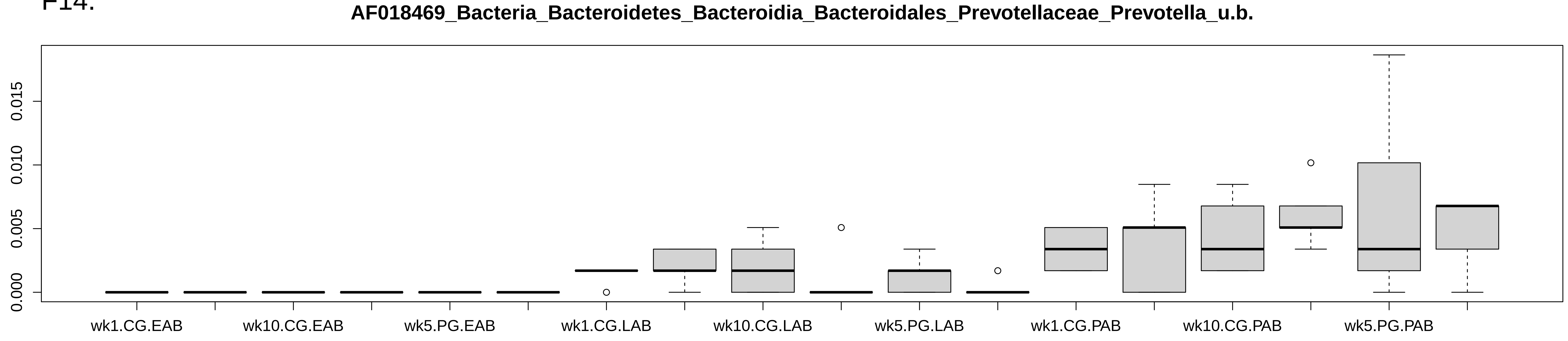

Statistics (p−values): Location: < 0.001; Group: 0.556; L×G: 0.074; Time: 0.601; L×T:1.000; G×T: 0.244; L×G×T: 0.991; Cow: 0.023; T×C: 0.524.

F15.

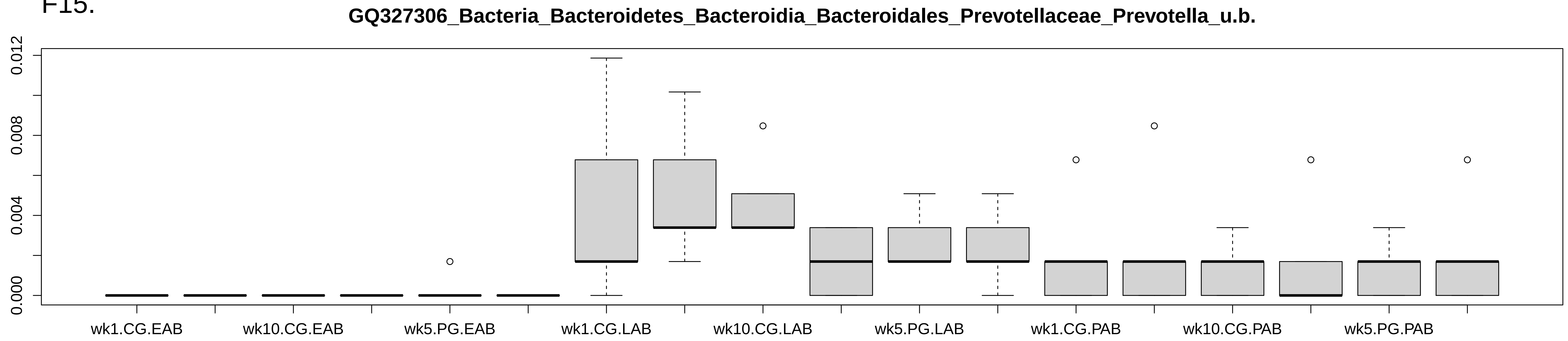

Statistics (p−values): Location: < 0.001; Group: 0.123; L×G: 0.054; Time: 0.588; L×T:0.855; G×T: 0.664; L×G×T: 1.000; Cow: 0.863; T×C: 0.647.

F16.

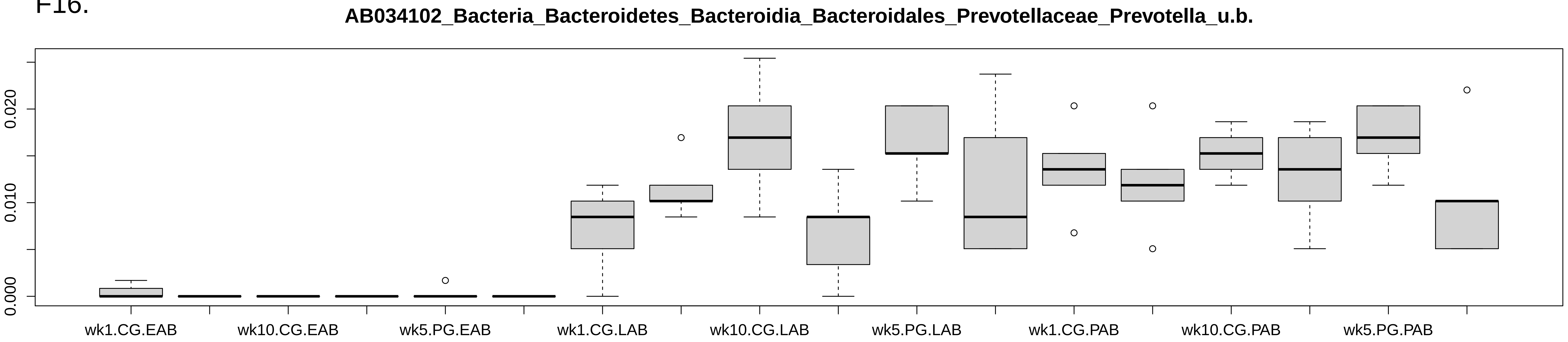

Statistics (p−values): Location: < 0.001; Group: 1.000; L×G: 1.000; Time: 0.042; L×T:0.075; G×T: 0.035; L×G×T: 0.485; Cow: 0.564; T×C: 0.970.

F17.

AB270138_Bacteria_Bacteroidetes_Bacteroidia_Bacteroidales_Prevotellaceae_Prevotella_u.b.

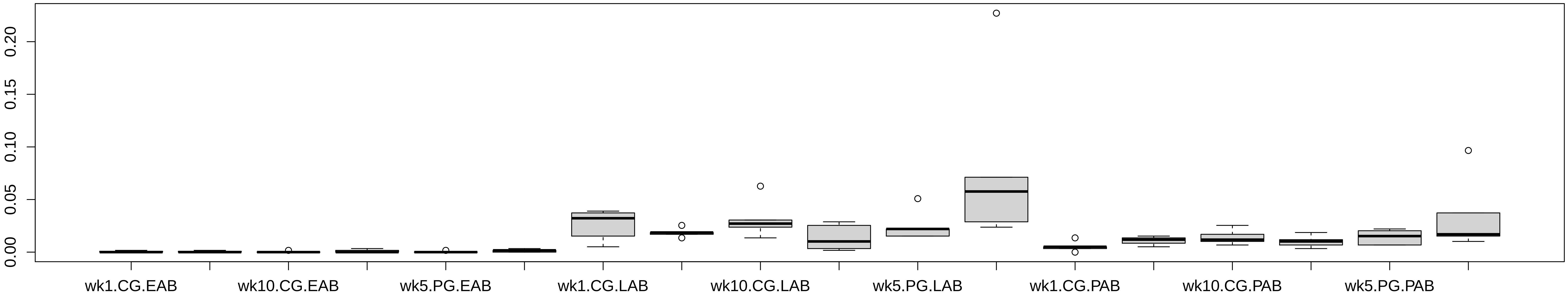

Statistics (p−values): Location: < 0.001; Group: 0.068; L×G: 0.510; Time: 0.003; L×T:0.125; G×T: 0.043; L×G×T: 0.380; Cow: 0.170; T×C: 0.215.

F18.

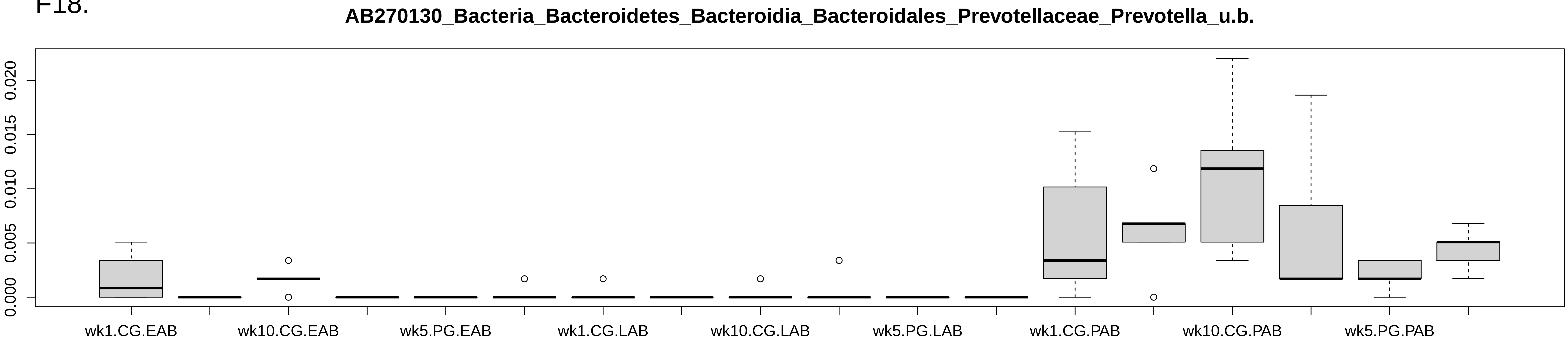

Statistics (p−values): Location: < 0.001; Group: 0.563; L×G: 0.128; Time: 0.251; L×T:0.351; G×T: 0.300; L×G×T: 0.372; Cow: 0.205; T×C: 0.849.

F19.

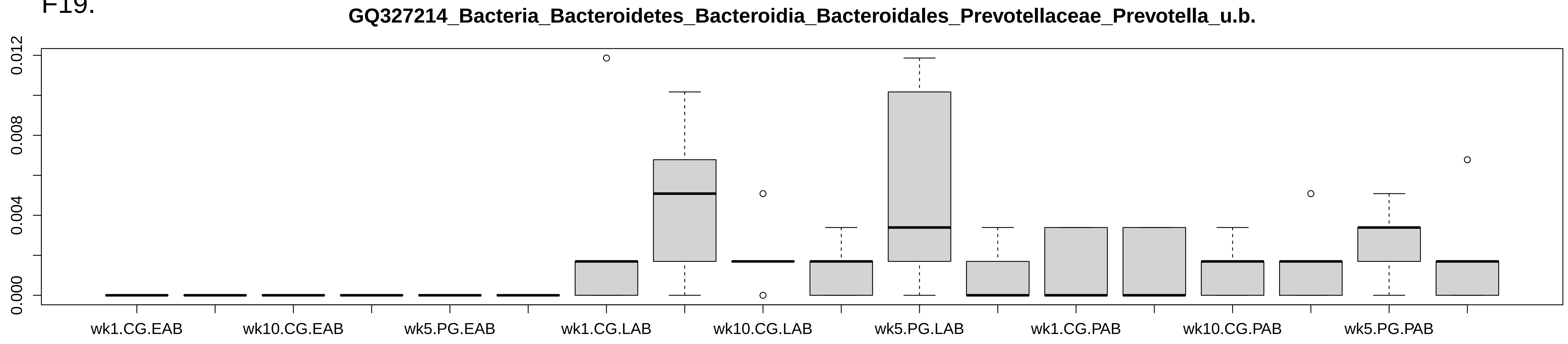

Statistics (p−values): Location: < 0.001; Group: 0.882; L×G: 0.788; Time: 0.075; L×T:0.140; G×T: 0.742; L×G×T: 0.981; Cow: 0.128; T×C: 0.549.

F20.

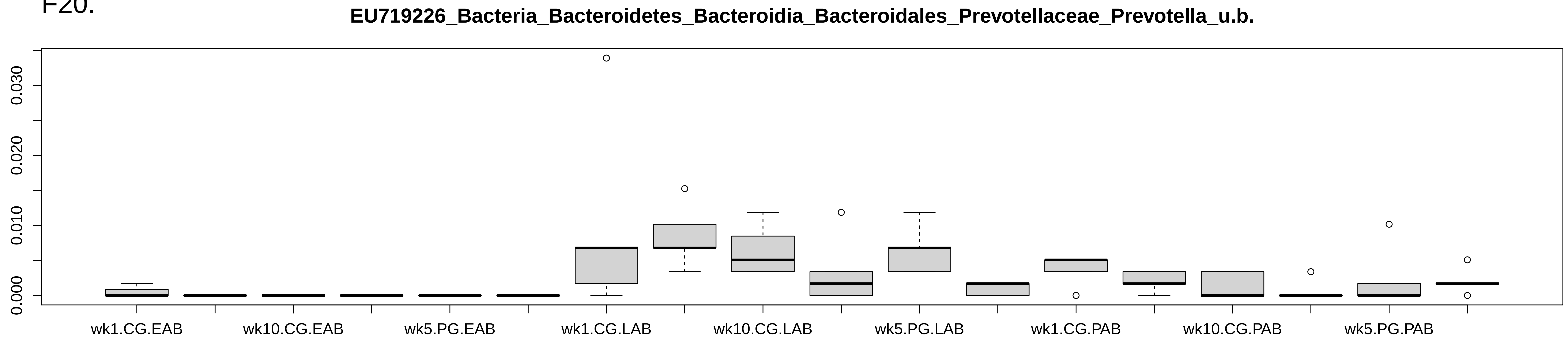

Statistics (p−values): Location: < 0.001; Group: 0.053; L×G: 0.046; Time: 0.470; L×T:0.776; G×T: 0.765; L×G×T: 1.000; Cow: 0.433; T×C: 0.863.

F21.

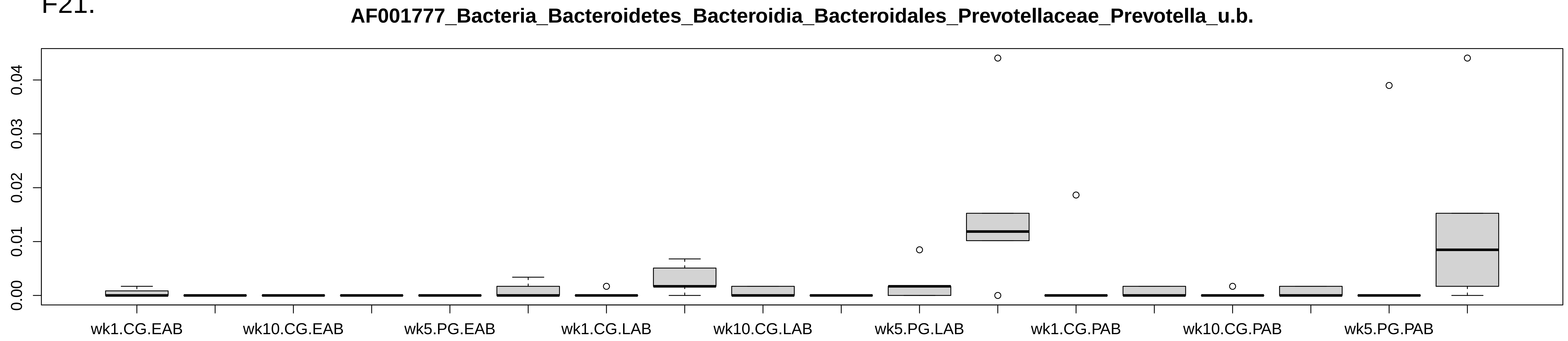

Statistics (p−values): Location: 0.062; Group: 0.050; L×G: 0.679; Time: 0.034; L×T:0.746; G×T: 0.009; L×G×T: 0.570; Cow: 0.338; T×C: 1.000.

F22.

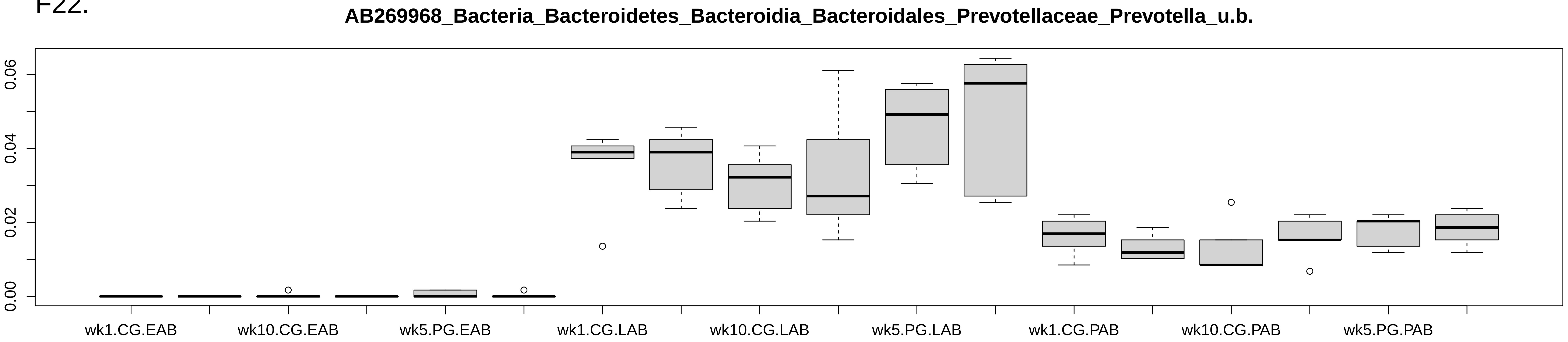

Statistics (p−values): Location: < 0.001; Group: 0.045; L×G: 0.321; Time: 1.000; L×T:0.771; G×T: 0.245; L×G×T: 0.638; Cow: 0.843; T×C: 0.453.

F23.

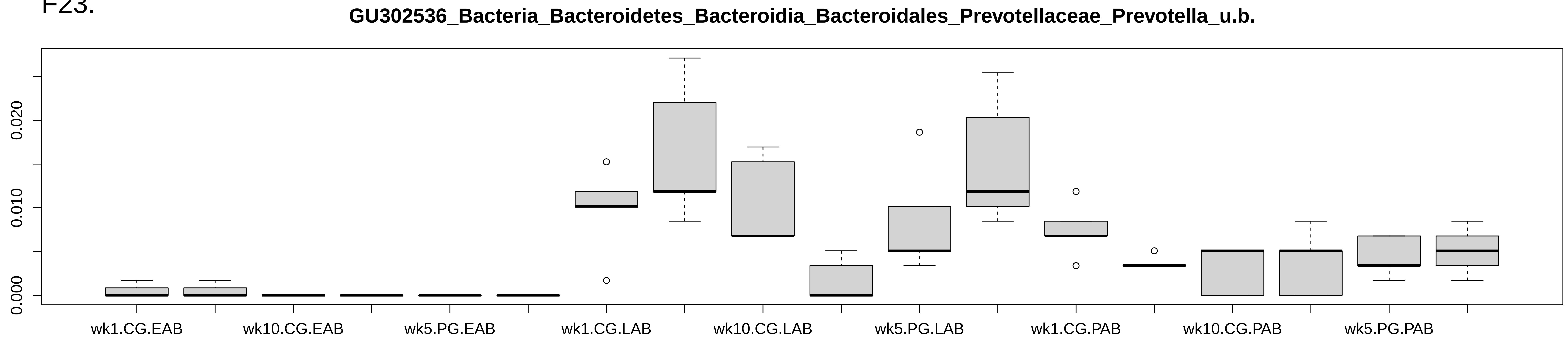

Statistics (p−values): Location: < 0.001; Group: 0.134; L×G: 0.135; Time: 0.136; L×T:0.003; G×T: 0.022; L×G×T: 0.067; Cow: 0.902; T×C: 0.539.

F24.

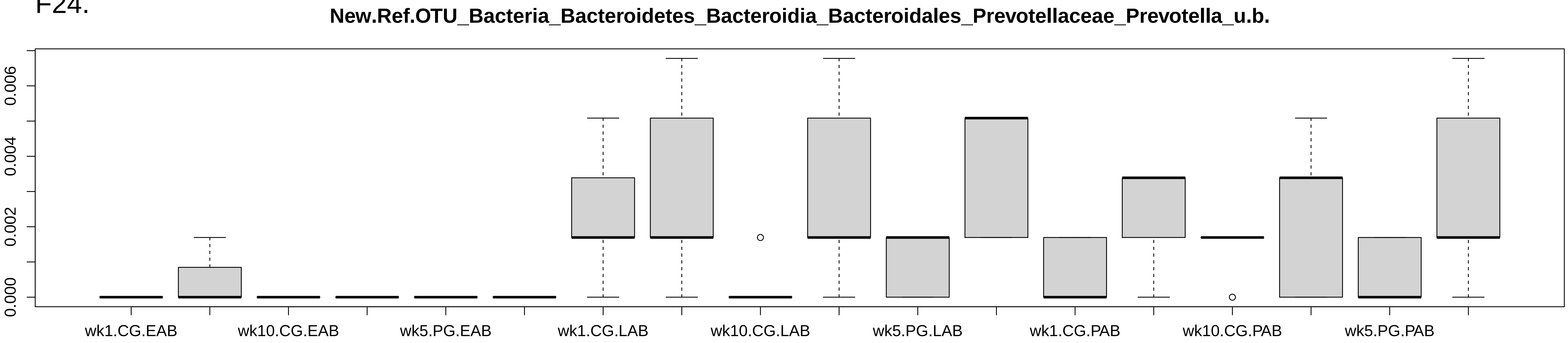

Statistics (p−values): Location: 0.001; Group: 0.655; L×G: 0.847; Time: 0.817; L×T:0.681; G×T: 0.003; L×G×T: 0.177; Cow: 0.491; T×C: 0.980.

F25.

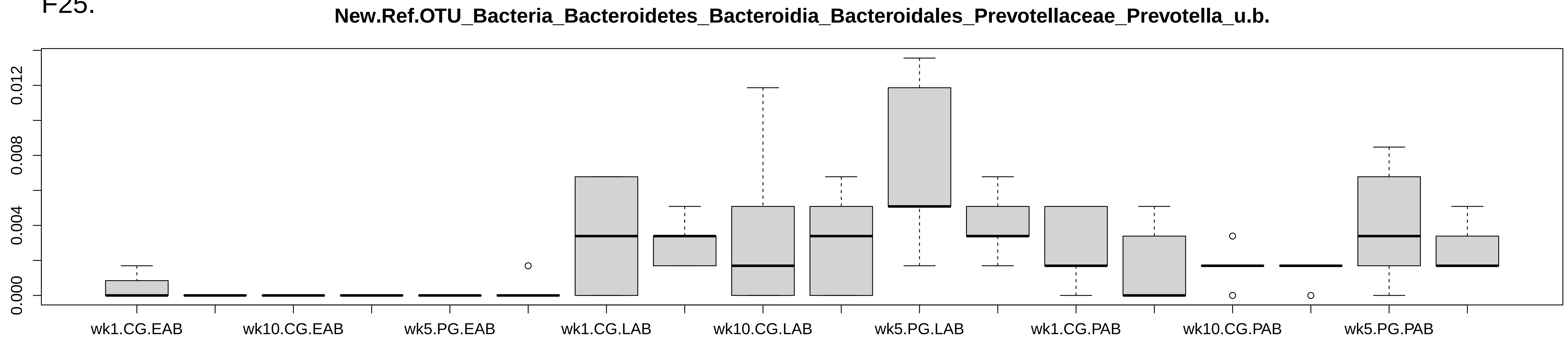

Statistics (p−values): Location: < 0.001; Group: 0.121; L×G: 0.477; Time: 0.555; L×T:0.887; G×T: 0.144; L×G×T: 0.813; Cow: 1.000; T×C: 0.909.

F26.

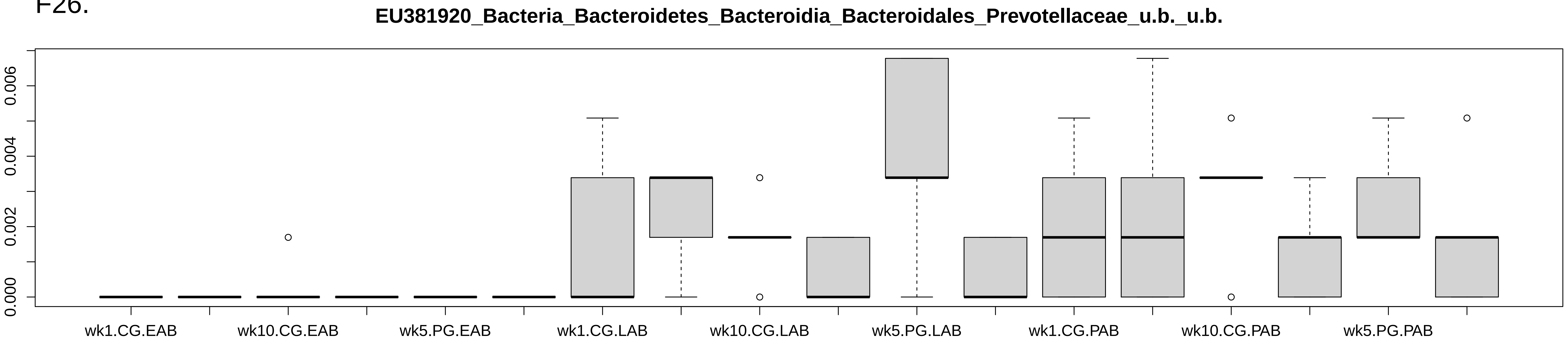

Statistics (p−values): Location: < 0.001; Group: 0.843; L×G: 0.839; Time: 0.047; L×T:0.169; G×T: 0.173; L×G×T: 0.580; Cow: 0.073; T×C: 0.567.

F27.

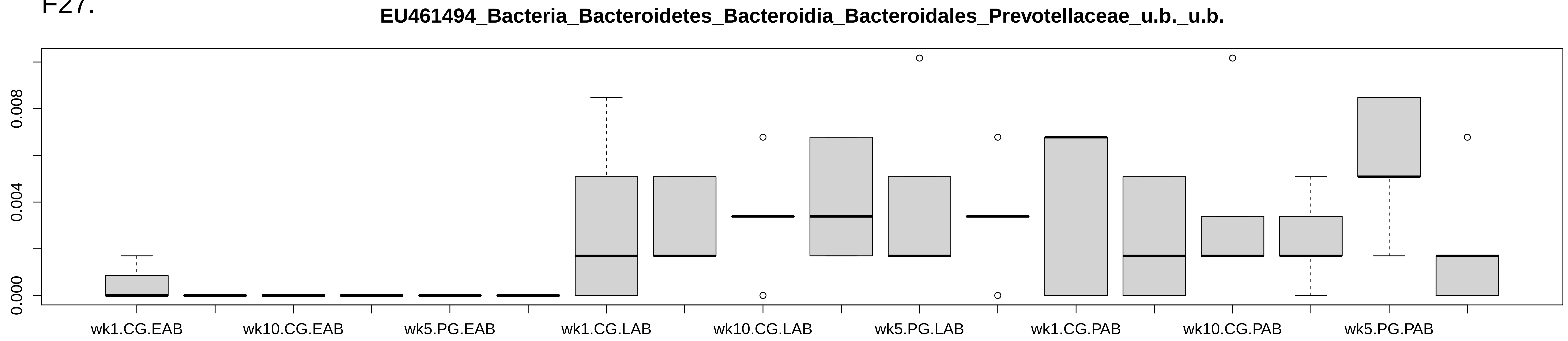

Statistics (p−values): Location: < 0.001; Group: 0.824; L×G: 0.711; Time: 1.000; L×T:1.000; G×T: 0.228; L×G×T: 0.599; Cow: 0.660; T×C: 1.000.

G1.

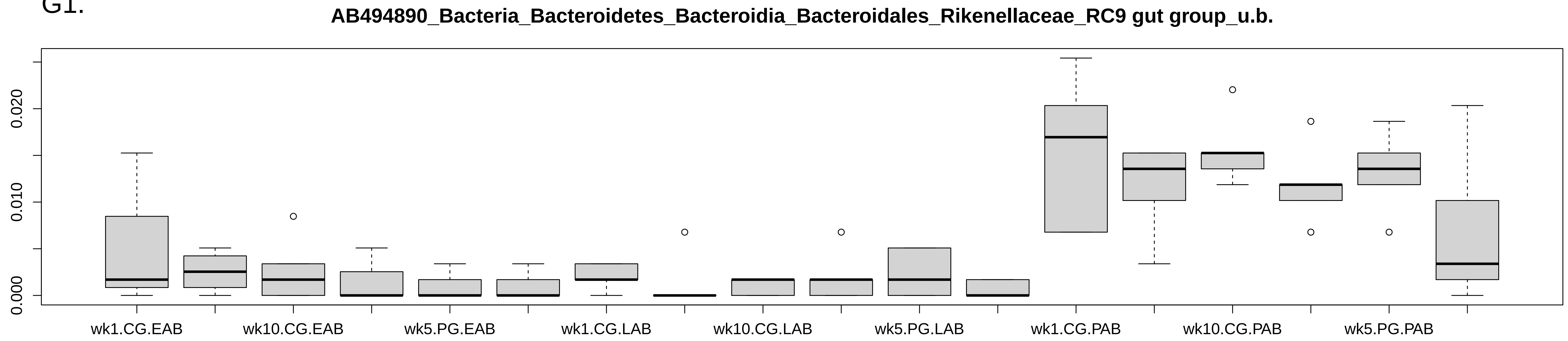

Statistics (p−values): Location: < 0.001; Group: 0.154; L×G: 0.236; Time: 0.379; L×T:1.000; G×T: 0.151; L×G×T: 0.392; Cow: 1.000; T×C: 0.442.

G2.

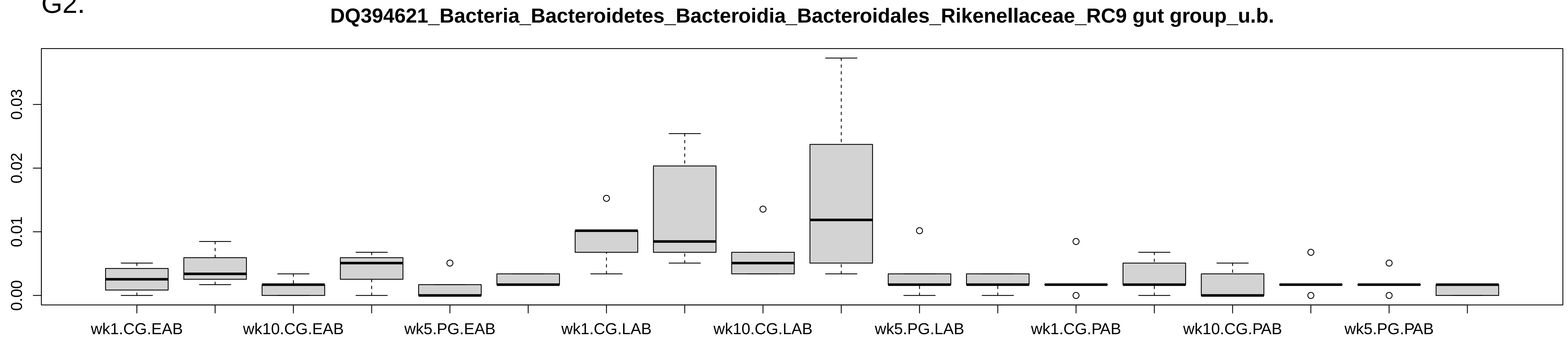

Statistics (p−values): Location: < 0.001; Group: 1.000; L×G: 0.961; Time: 0.011; L×T:0.187; G×T: 0.025; L×G×T: 0.103; Cow: 0.466; T×C: 1.000.

G3.

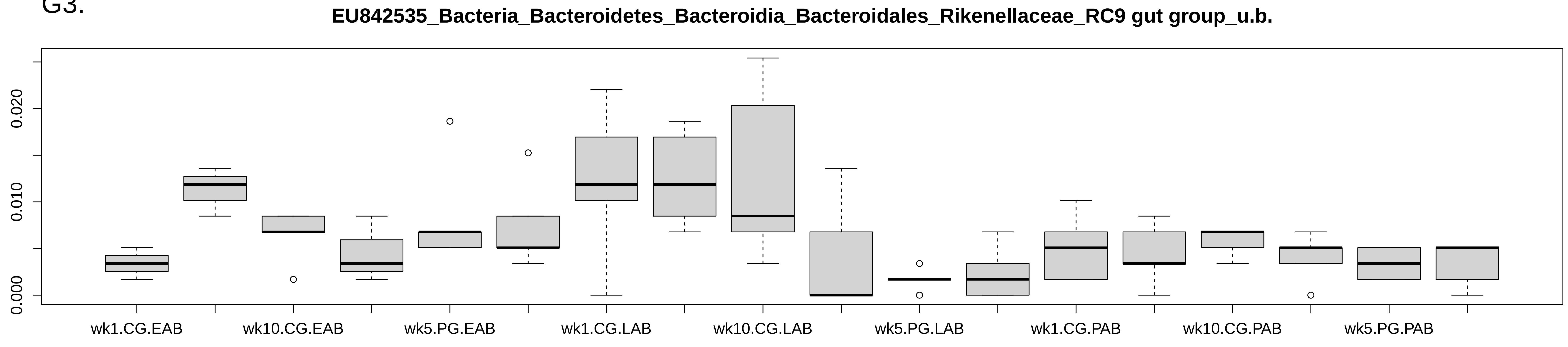

Statistics (p−values): Location: 0.017; Group: < 0.001; L×G: < 0.001; Time: 0.620; L×T:0.200; G×T: 0.659; L×G×T: 1.000; Cow: 0.231; T×C: 0.902.

G4.

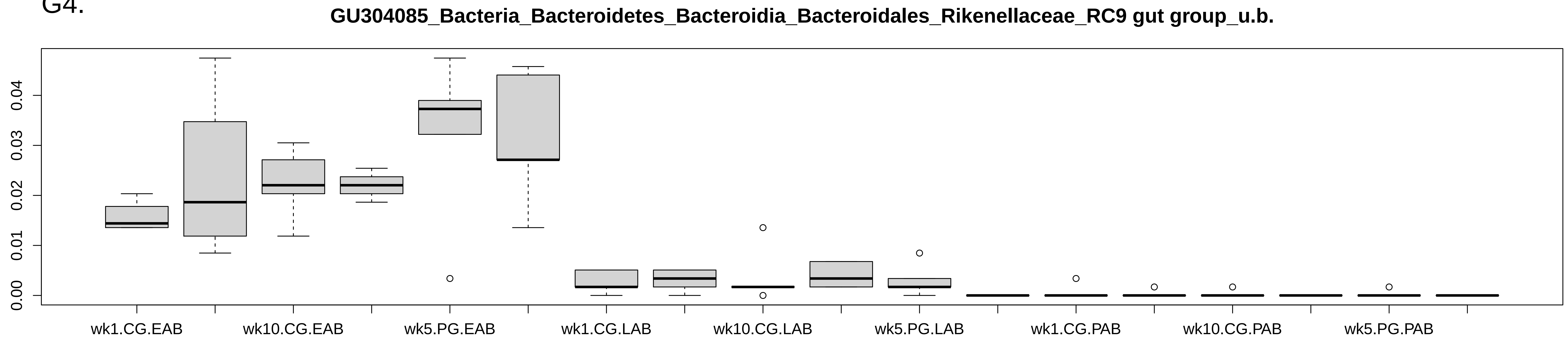

Statistics (p−values): Location: < 0.001; Group: 0.239; L×G: 0.114; Time: 0.478; L×T:0.205; G×T: 1.000; L×G×T: 0.941; Cow: 1.000; T×C: 0.753.

G5.

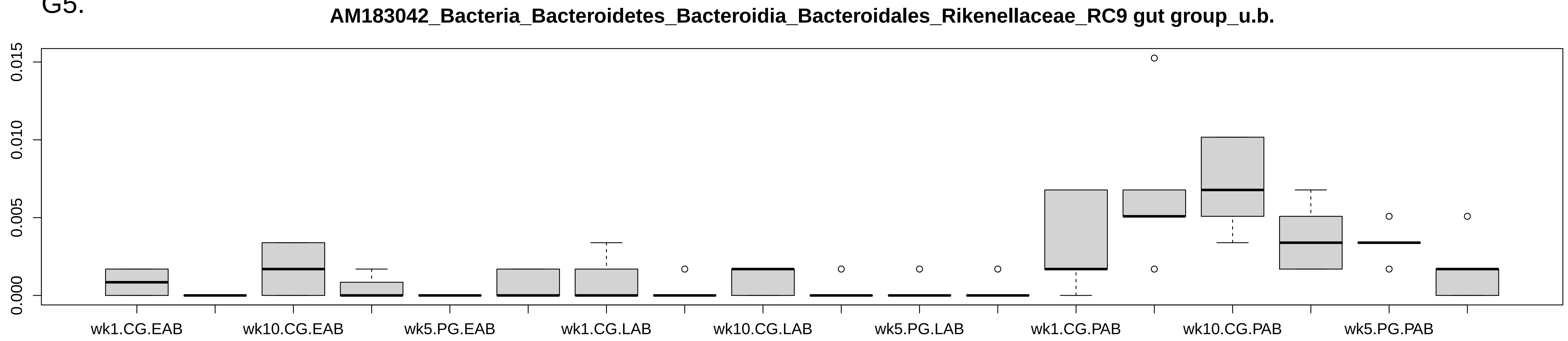

Statistics (p−values): Location: < 0.001; Group: 0.011; L×G: 0.036; Time: 0.815; L×T:0.420; G×T: 0.149; L×G×T: 0.238; Cow: 0.418; T×C: 1.000.

G6.

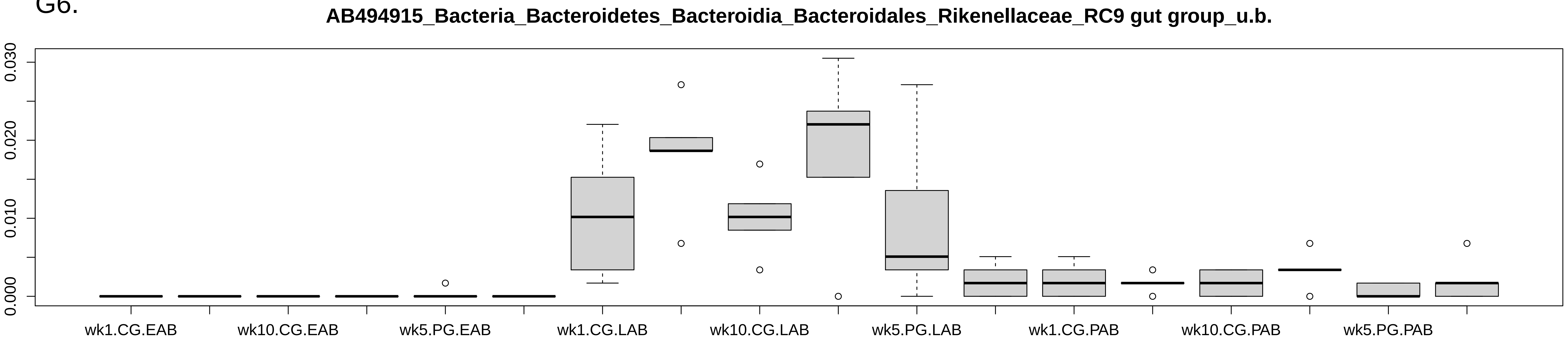

Statistics (p−values): Location: < 0.001; Group: 0.655; L×G: 1.000; Time: 0.079; L×T:0.026; G×T: 0.726; L×G×T: 0.062; Cow: 1.000; T×C: 0.271.

G7.

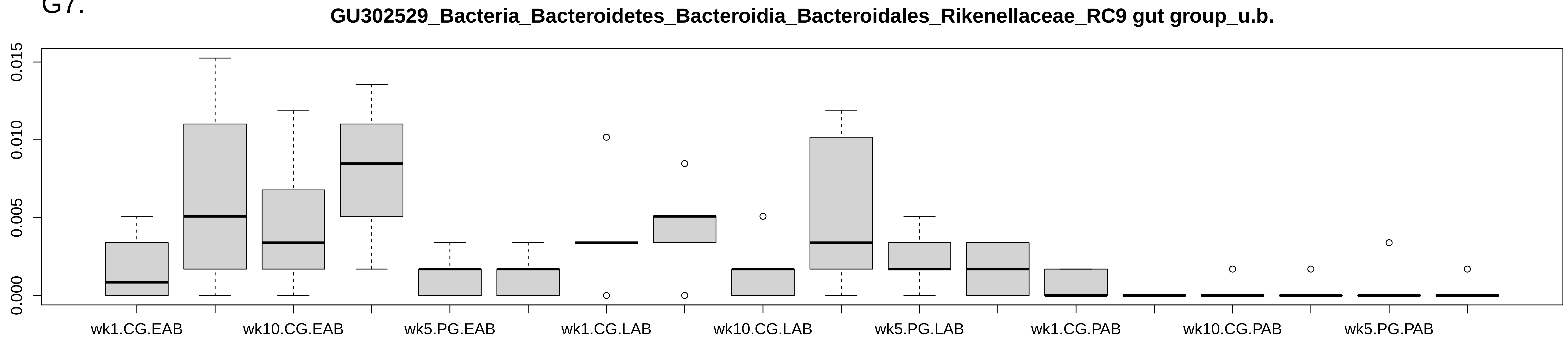

Statistics (p−values): Location: < 0.001; Group: 0.667; L×G: 0.902; Time: 0.070; L×T:0.744; G×T: 0.020; L×G×T: 0.034; Cow: 0.130; T×C: 0.145.

G8.

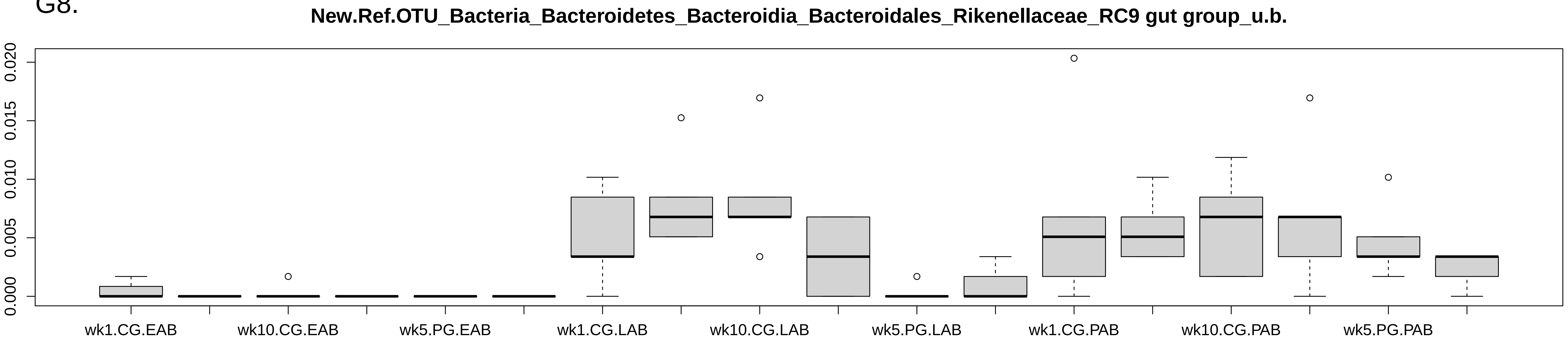

Statistics (p−values): Location: < 0.001; Group: 0.003; L×G: 0.018; Time: 0.593; L×T:0.927; G×T: 0.296; L×G×T: 0.797; Cow: 1.000; T×C: 0.424.

H1.

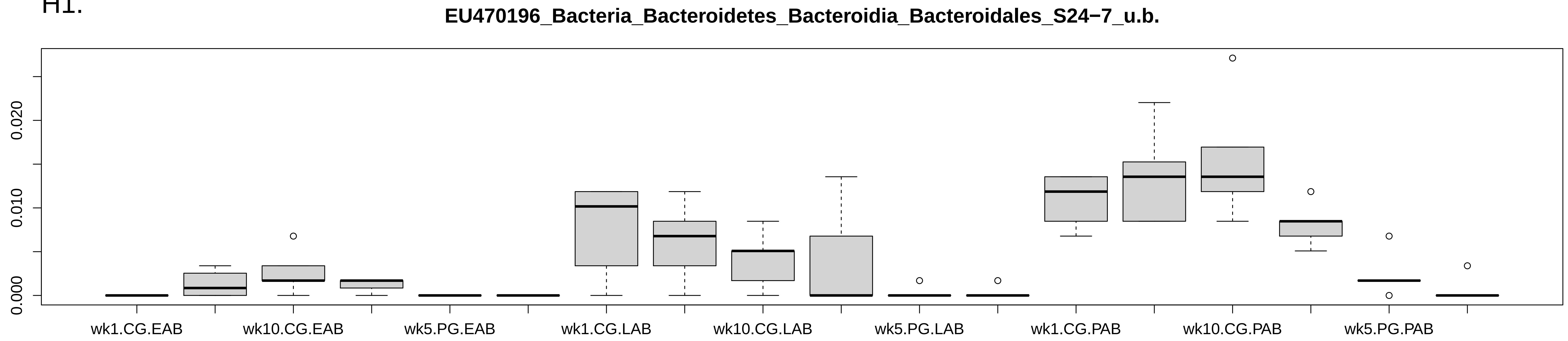

Statistics (p−values): Location: < 0.001; Group: < 0.001; L×G: < 0.001; Time: 0.621; L×T:0.407; G×T: 0.012; L×G×T: 0.127; Cow: 0.941; T×C: 1.000.

H2.

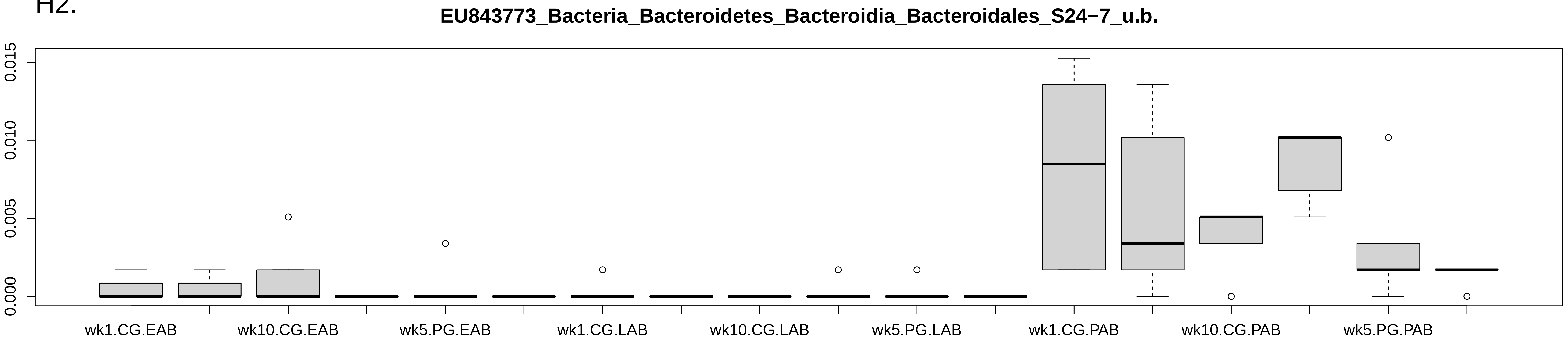

Statistics (p−values): Location: < 0.001; Group: 0.273; L×G: 1.000; Time: 0.018; L×T:0.004; G×T: 0.985; L×G×T: 1.000; Cow: 0.941; T×C: 0.691.

I.

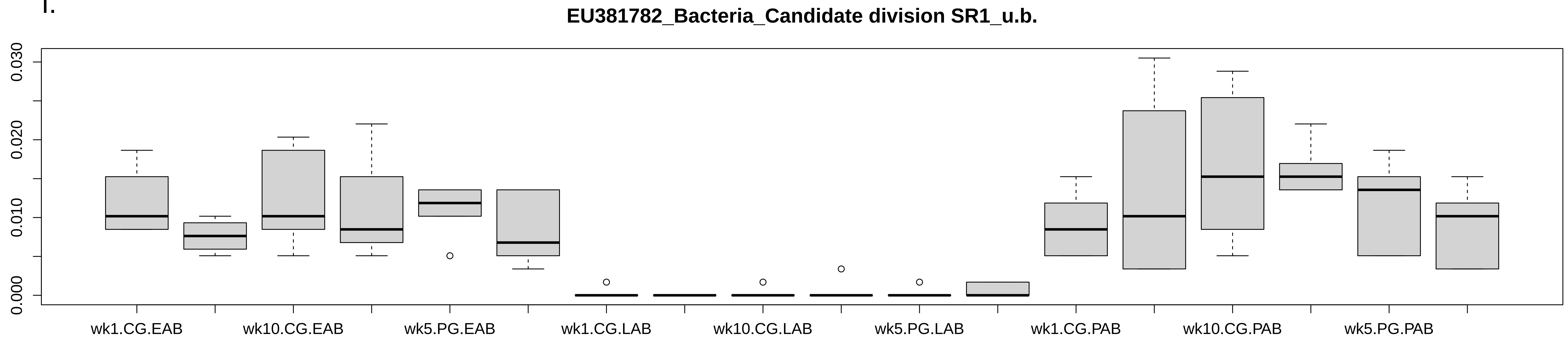

Statistics (p−values): Location: < 0.001; Group: 0.902; L×G: 0.726; Time: 0.603; L×T:0.843; G×T: 0.150; L×G×T: 0.237; Cow: 0.765; T×C: 0.656.

J1.

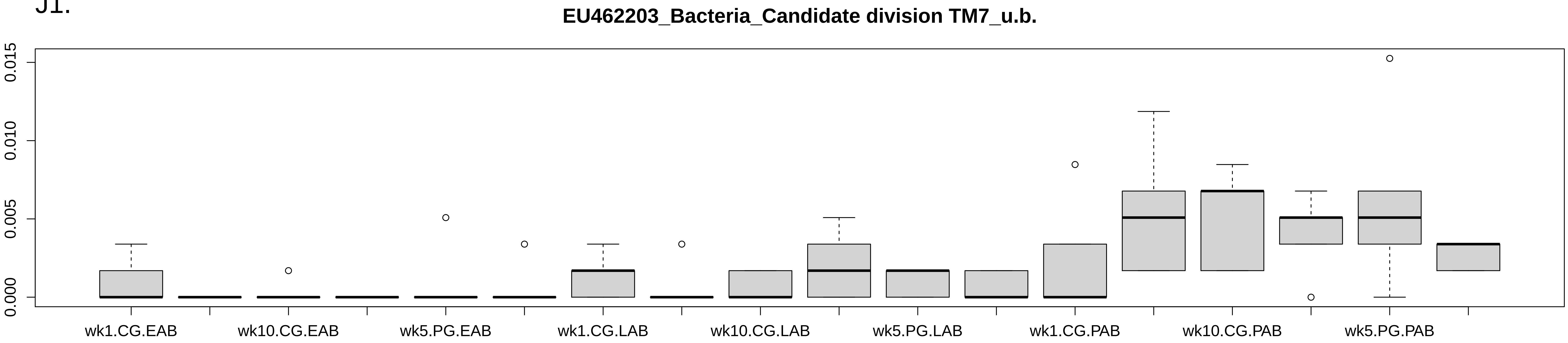

Statistics (p−values): Location: < 0.001; Group: 0.615; L×G: 1.000; Time: 0.321; L×T:0.270; G×T: 0.497; L×G×T: 0.876; Cow: 1.000; T×C: 0.363.

J2.

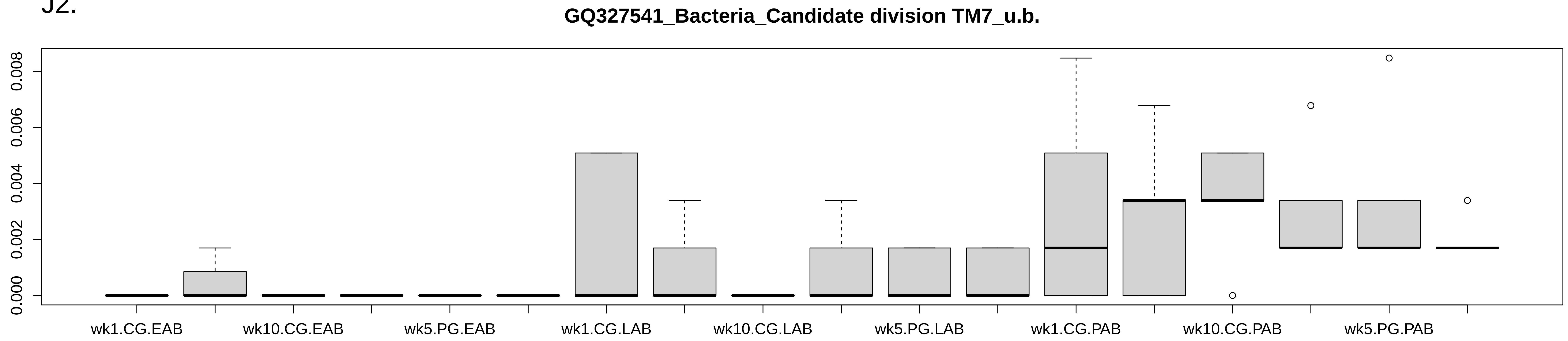

Statistics (p−values): Location: < 0.001; Group: 0.603; L×G: 1.000; Time: 1.000; L×T:0.938; G×T: 0.902; L×G×T: 0.669; Cow: 1.000; T×C: 0.484.

J3.

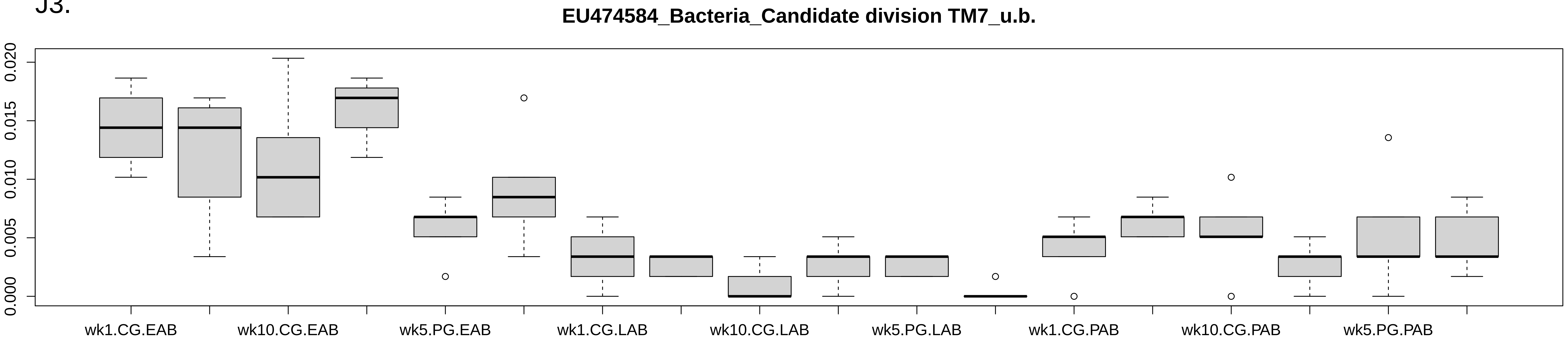

Statistics (p−values): Location: < 0.001; Group: 0.046; L×G: 0.438; Time: 0.113; L×T:0.007; G×T: 0.912; L×G×T: 0.378; Cow: 0.638; T×C: 0.630.

J4.

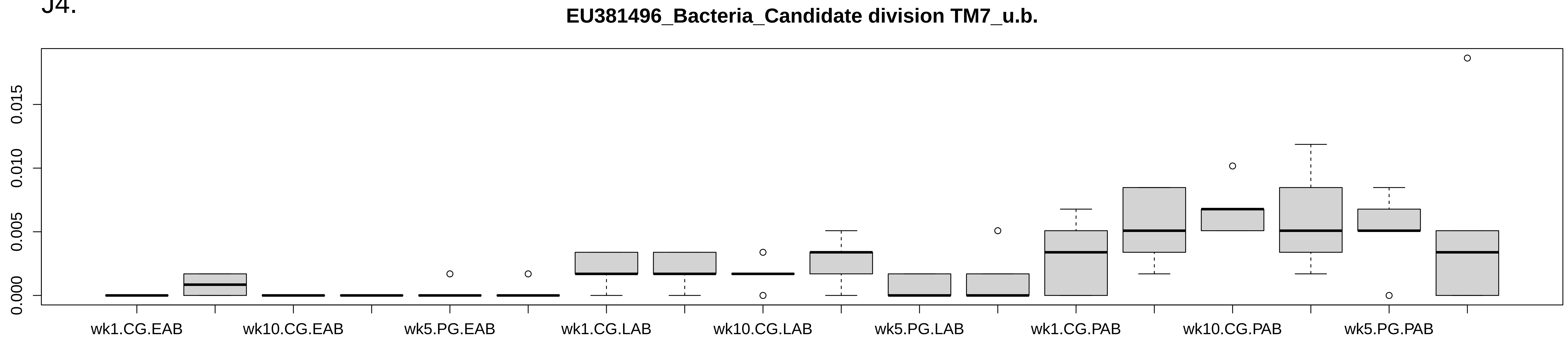

Statistics (p−values): Location: < 0.001; Group: 1.000; L×G: 0.880; Time: 0.919; L×T:0.563; G×T: 0.427; L×G×T: 0.665; Cow: 0.902; T×C: 0.516.

K.

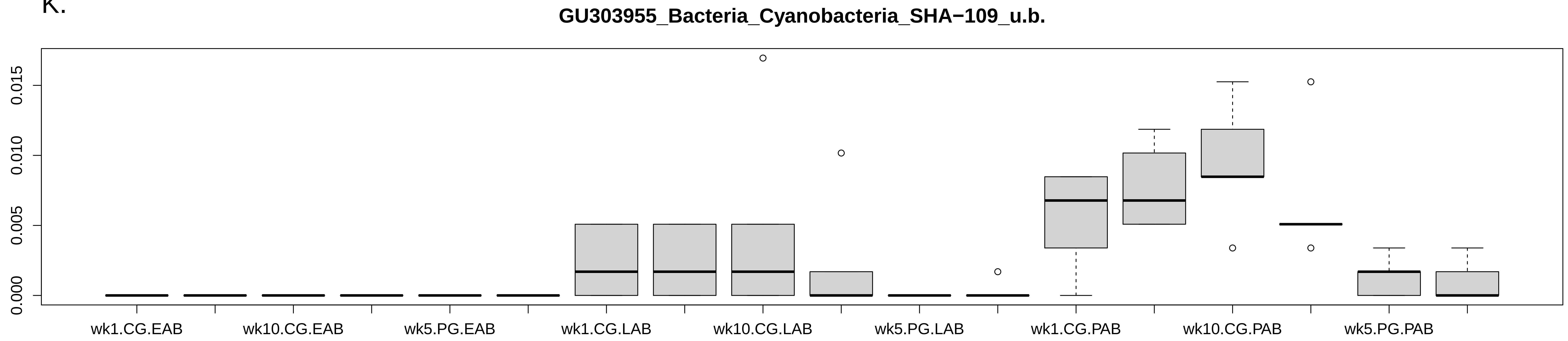

Statistics (p−values): Location: < 0.001; Group: 0.006; L×G: 0.001; Time: 0.523; L×T:1.000; G×T: 0.000; L×G×T: 0.257; Cow: 0.210; T×C: 0.255.

L1.

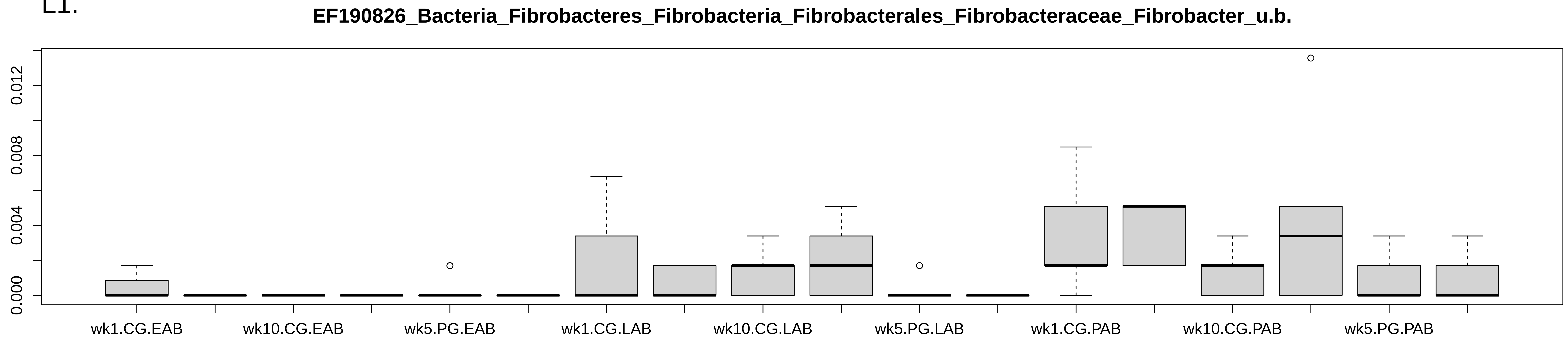

Statistics (p−values): Location: < 0.001; Group: 0.168; L×G: 0.891; Time: 0.021; L×T:0.270; G×T: 0.606; L×G×T: 0.775; Cow: 0.606; T×C: 0.787.

L2.

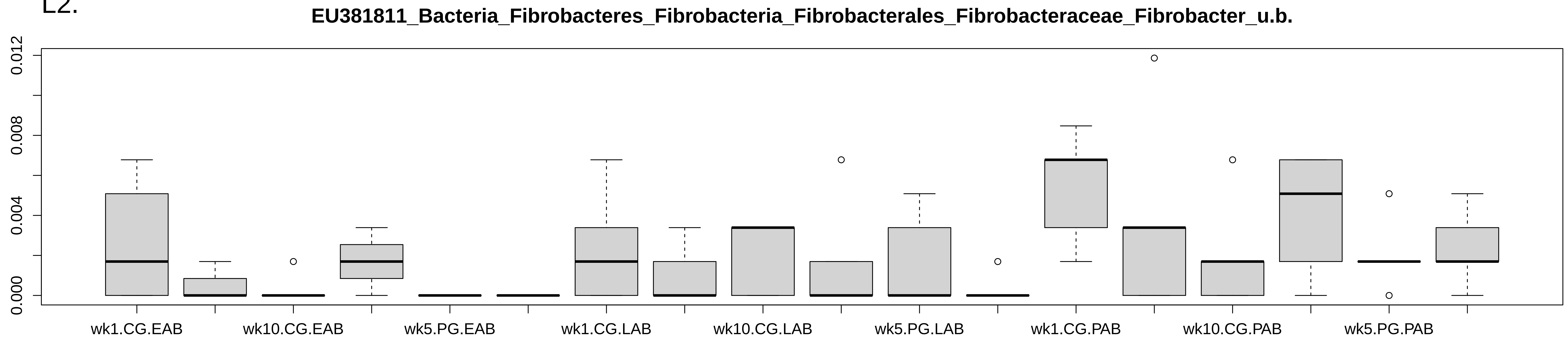

Statistics (p−values): Location: < 0.001; Group: 0.327; L×G: 0.961; Time: 0.009; L×T:0.977; G×T: 0.748; L×G×T: 0.601; Cow: 0.327; T×C: 0.039.

L3.

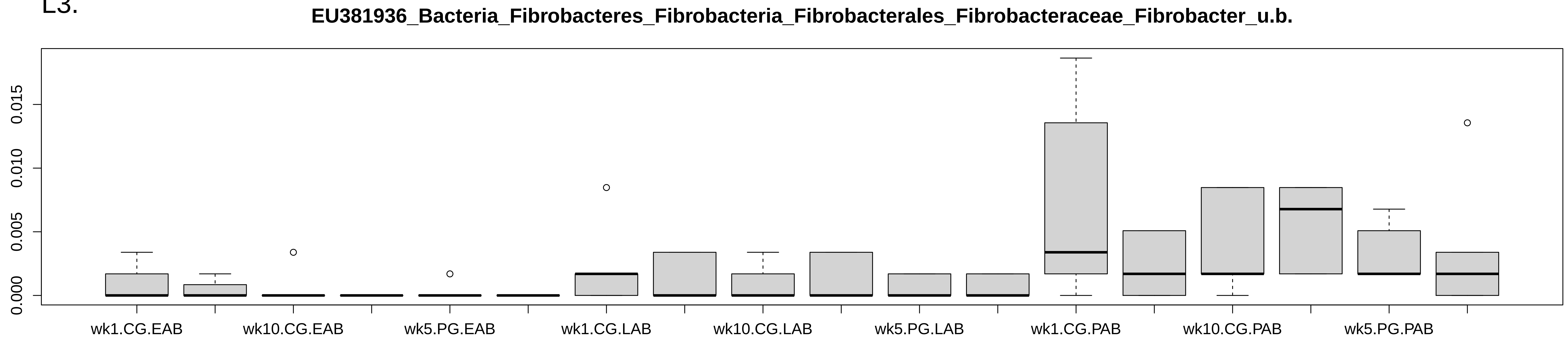

Statistics (p−values): Location: < 0.001; Group: 0.592; L×G: 1.000; Time: 0.200; L×T:0.601; G×T: 0.619; L×G×T: 0.990; Cow: 0.451; T×C: 0.882.

L4.

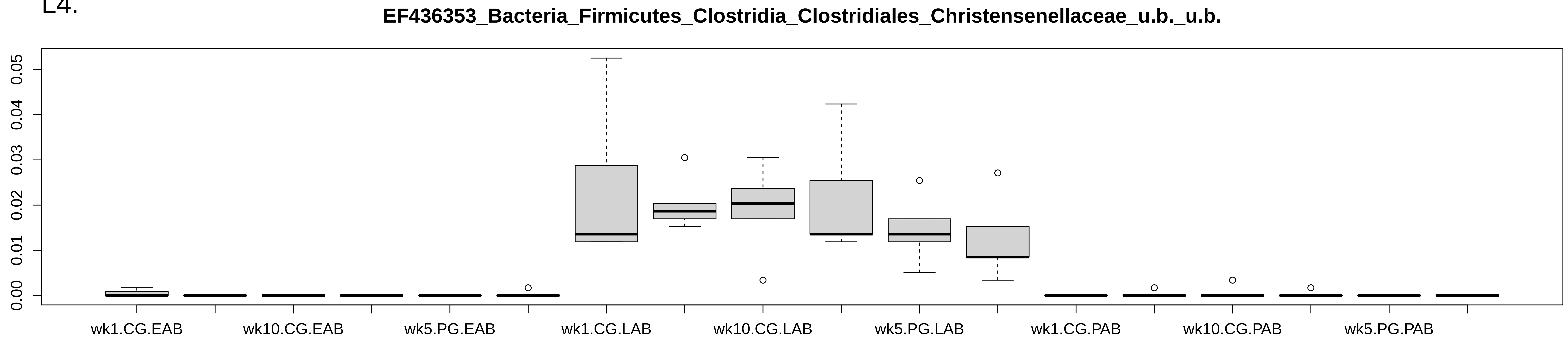

Statistics (p−values): Location: < 0.001; Group: 0.185; L×G: 0.783; Time: 0.225; L×T:0.393; G×T: 0.577; L×G×T: 1.000; Cow: 1.000; T×C: 0.656.

M1.

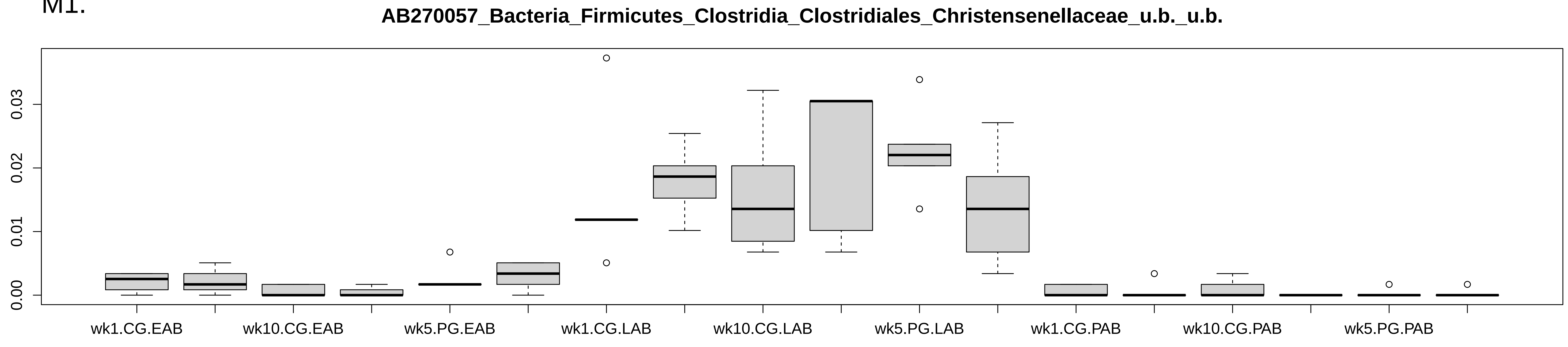

Statistics (p−values): Location: < 0.001; Group: 0.377; L×G: 0.413; Time: 0.478; L×T:0.582; G×T: 1.000; L×G×T: 0.426; Cow: 1.000; T×C: 0.843.

M2.

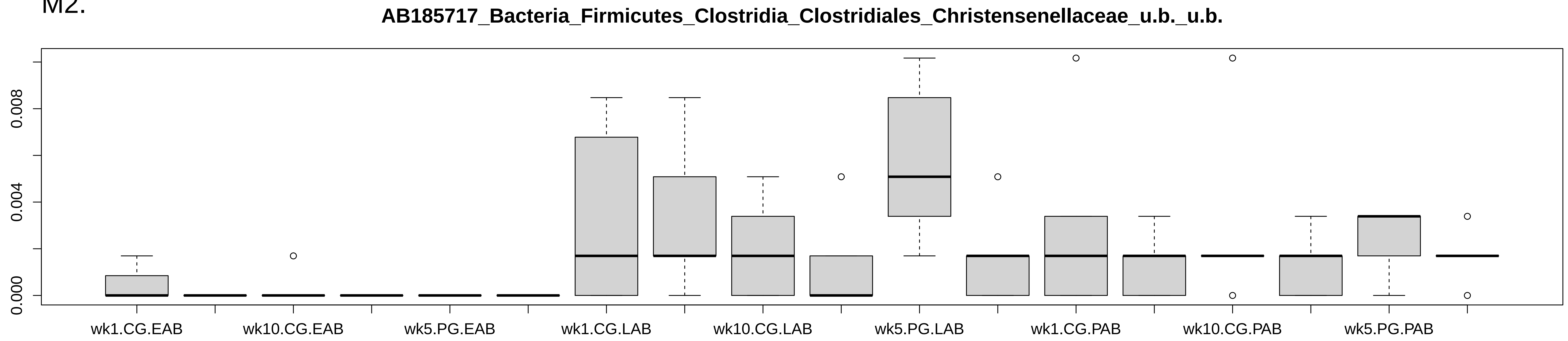

Statistics (p−values): Location: < 0.001; Group: 1.000; L×G: 1.000; Time: 1.000; L×T:0.245; G×T: 0.321; L×G×T: 0.829; Cow: 0.435; T×C: 0.190.

M3.

AB270004_Bacteria_Firmicutes_Clostridia_Clostridiales_Christensenellaceae_u.b._u.b.

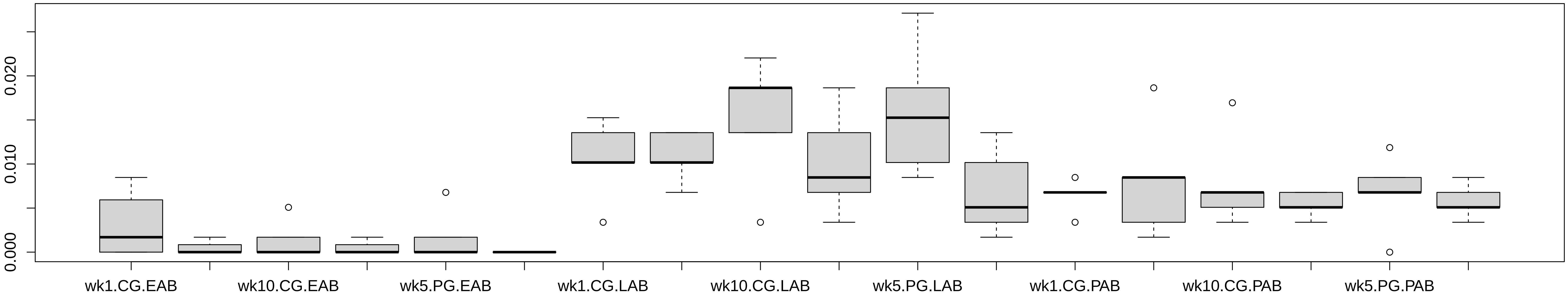

Statistics (p−values): Location: < 0.001; Group: 0.216; L×G: 1.000; Time: 0.571; L×T:0.988; G×T: 0.060; L×G×T: 0.176; Cow: 0.765; T×C: 0.838.

M4.

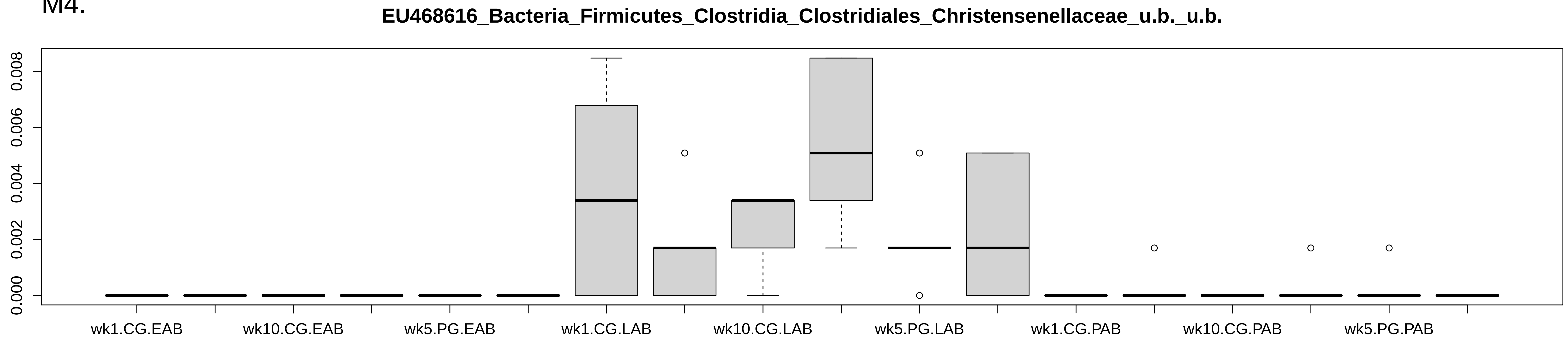

Statistics (p−values): Location: < 0.001; Group: 0.329; L×G: 0.876; Time: 0.162; L×T:0.029; G×T: 0.747; L×G×T: 0.983; Cow: 0.592; T×C: 1.000.

M5.

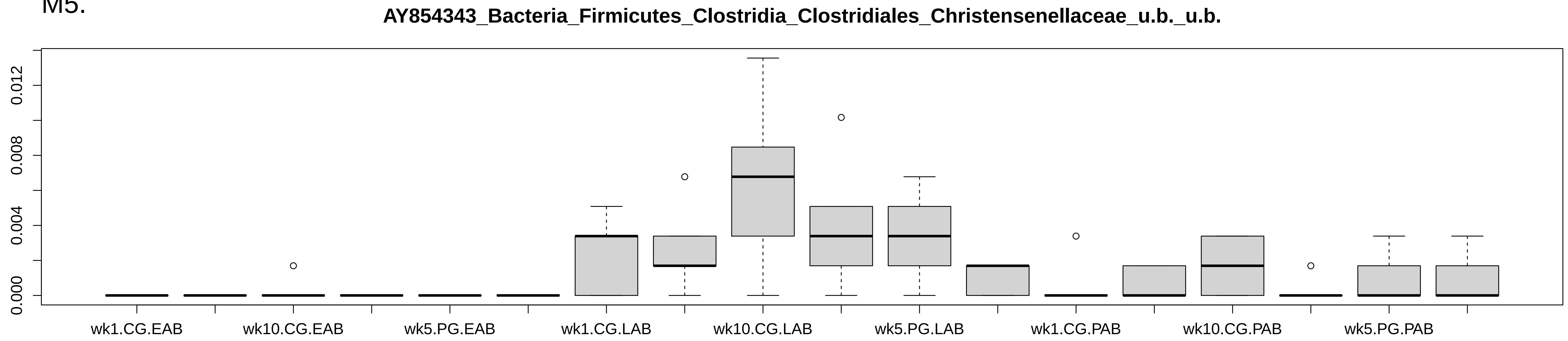

Statistics (p−values): Location: < 0.001; Group: 0.260; L×G: 1.000; Time: 0.619; L×T:1.000; G×T: 0.040; L×G×T: 0.075; Cow: 0.726; T×C: 0.811.

M6.

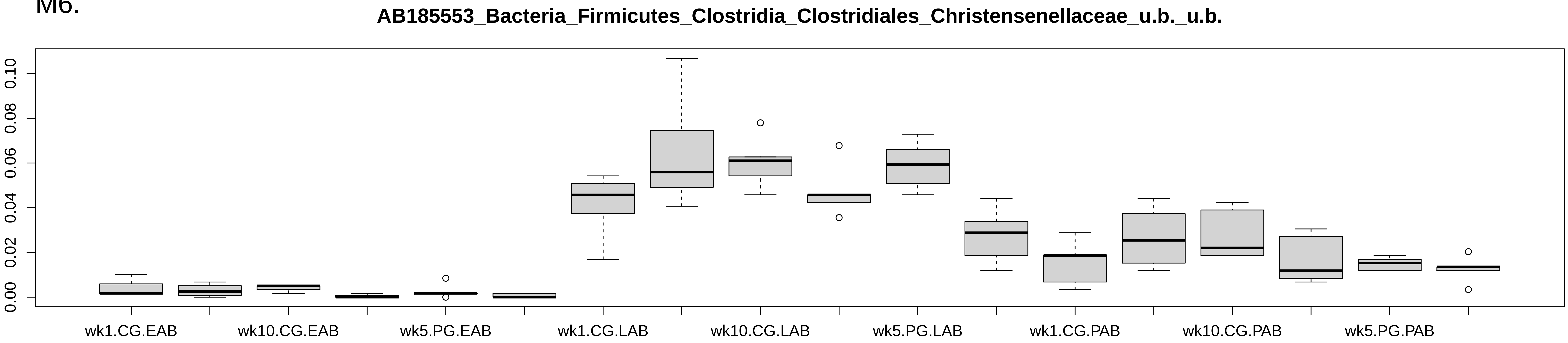

Statistics (p−values): Location: < 0.001; Group: 0.001; L×G: 0.370; Time: 0.024; L×T:0.087; G×T: 0.006; L×G×T: 0.189; Cow: 0.403; T×C: 0.344.

M7.

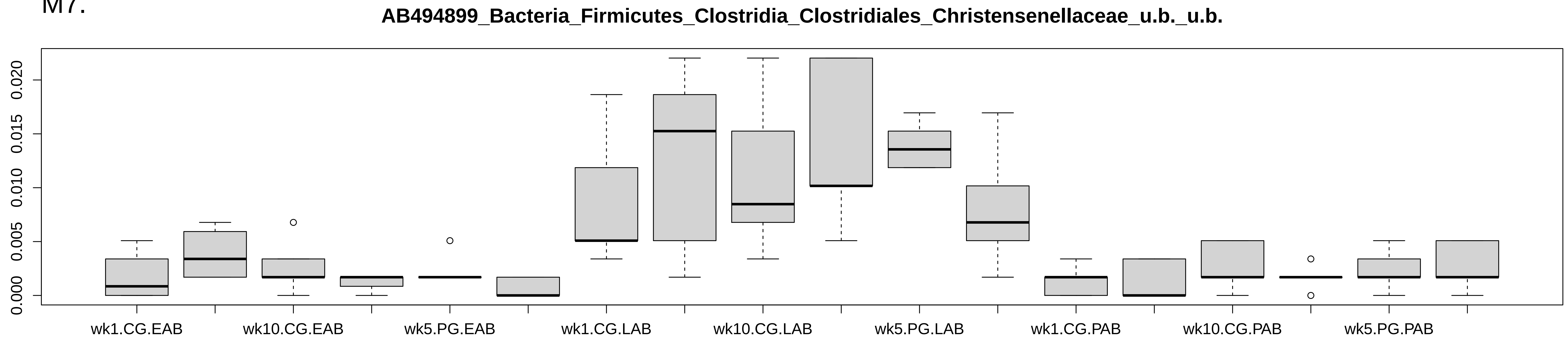

Statistics (p−values): Location: < 0.001; Group: 0.706; L×G: 0.500; Time: 0.398; L×T:0.620; G×T: 0.381; L×G×T: 0.736; Cow: 0.122; T×C: 1.000.

M8.

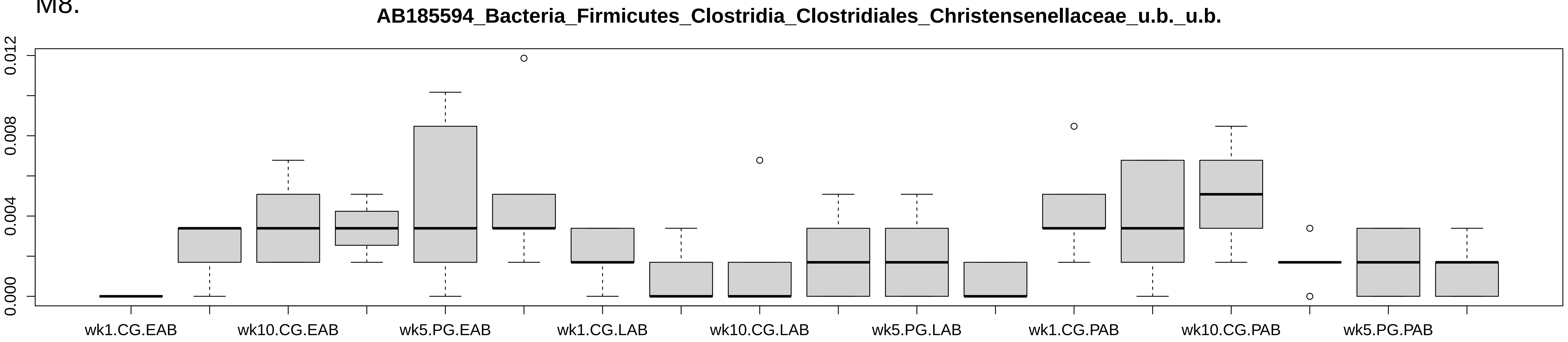

Statistics (p−values): Location: 0.039; Group: 0.615; L×G: 0.001; Time: 0.687; L×T:0.218; G×T: 0.409; L×G×T: 1.000; Cow: 0.643; T×C: 0.902.

N1.

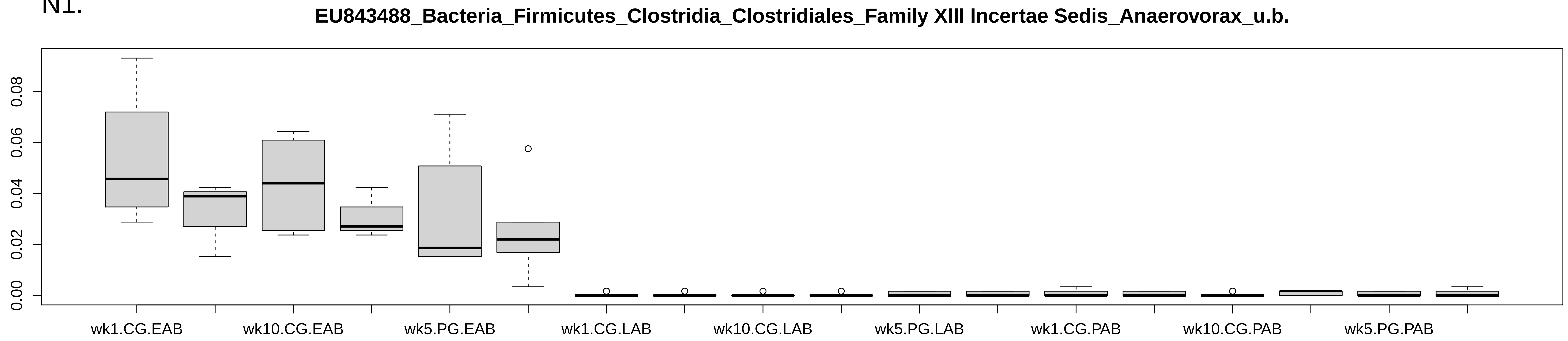

Statistics (p−values): Location: < 0.001; Group: 0.290; L×G: 0.044; Time: 0.824; L×T:0.749; G×T: 0.599; L×G×T: 0.664; Cow: 0.442; T×C: 0.863.

N2.

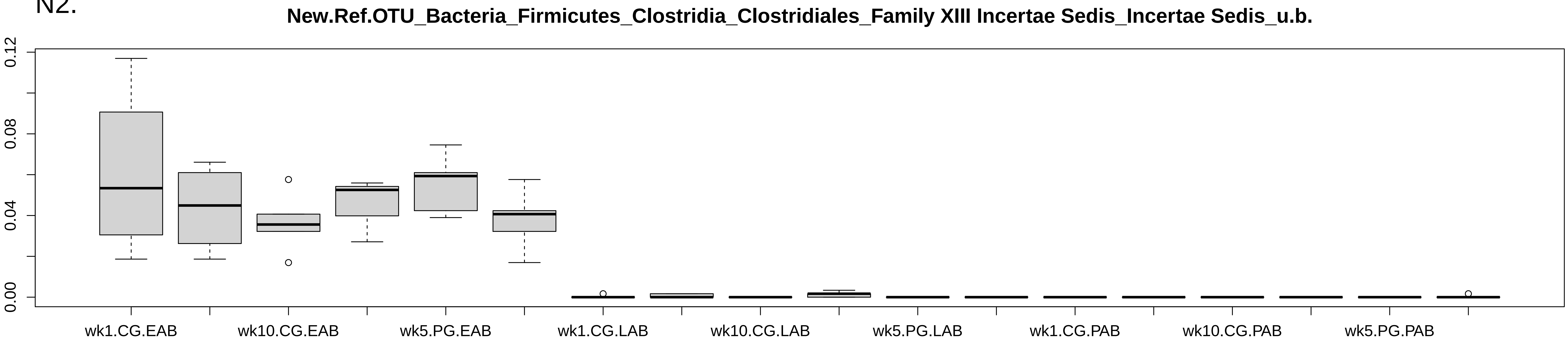

Statistics (p−values): Location: < 0.001; Group: 0.902; L×G: 1.000; Time: 0.207; L×T:0.232; G×T: 0.451; L×G×T: 0.487; Cow: 0.726; T×C: 0.466.

N3.

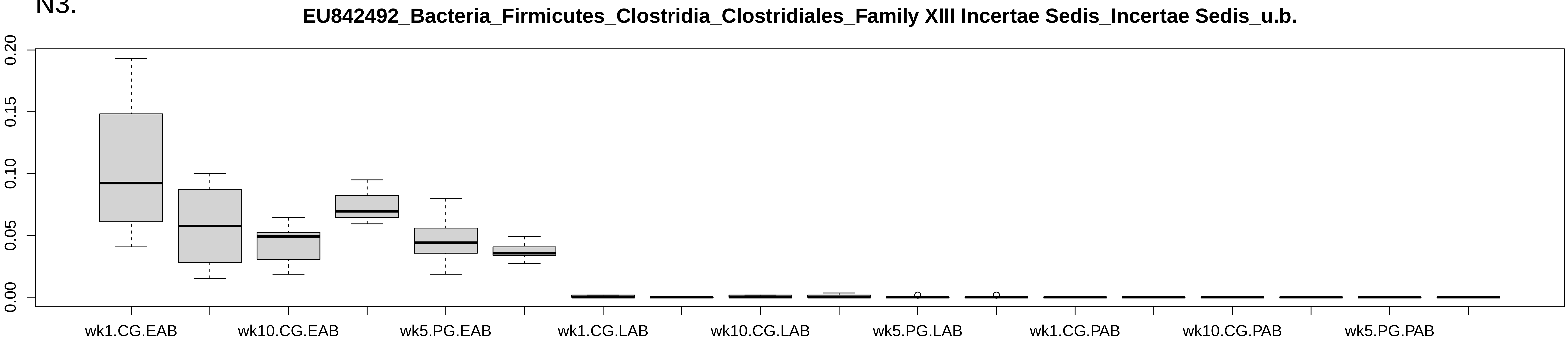

Statistics (p−values): Location: < 0.001; Group: 0.344; L×G: 0.230; Time: 0.002; L×T:0.003; G×T: 1.000; L×G×T: 0.866; Cow: 0.824; T×C: 0.391.

N4.

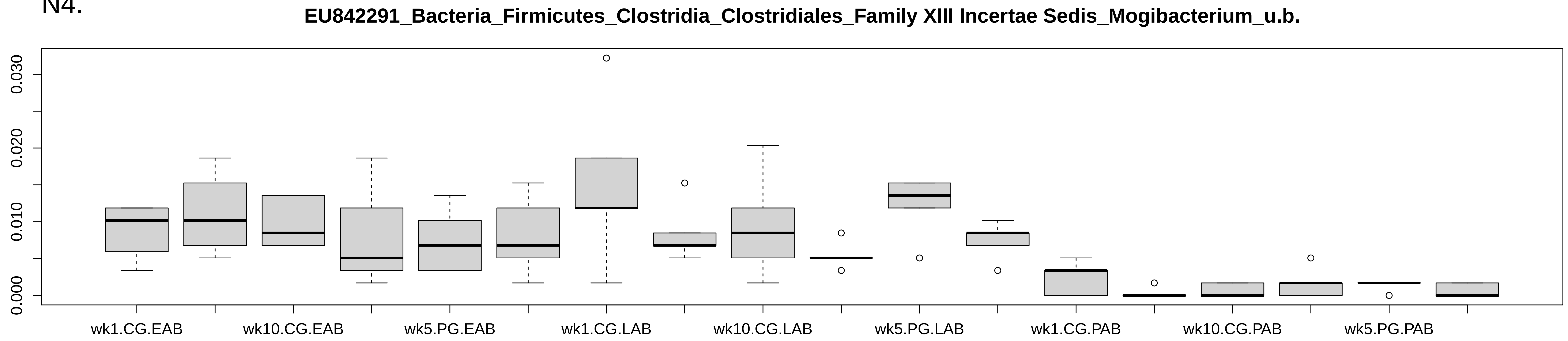

Statistics (p−values): Location: < 0.001; Group: 0.145; L×G: 0.617; Time: 0.626; L×T:0.926; G×T: 0.226; L×G×T: 0.132; Cow: 0.623; T×C: 0.653.

N5.

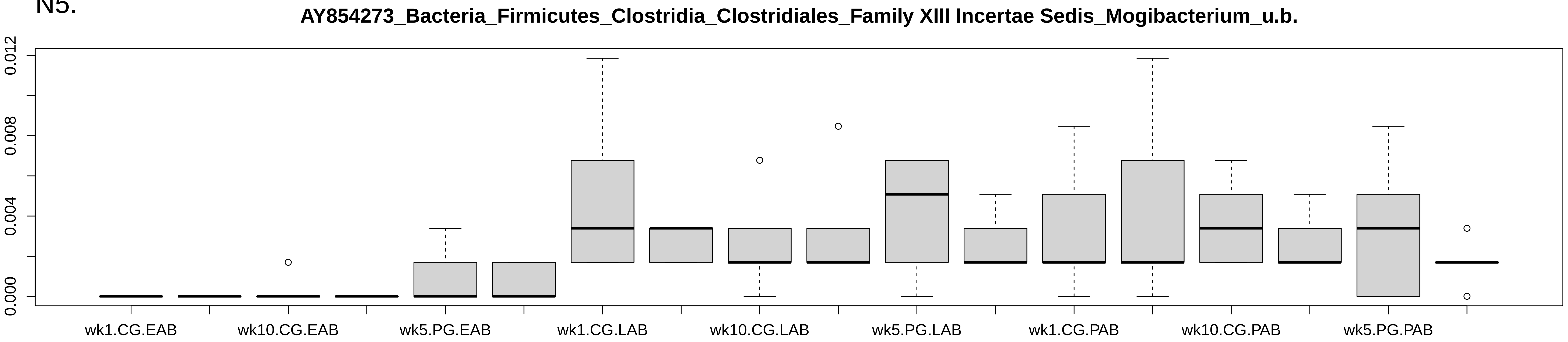

Statistics (p−values): Location: < 0.001; Group: 0.563; L×G: 0.381; Time: 0.610; L×T:0.576; G×T: 0.685; L×G×T: 0.801; Cow: 0.014; T×C: 0.675.

N6.

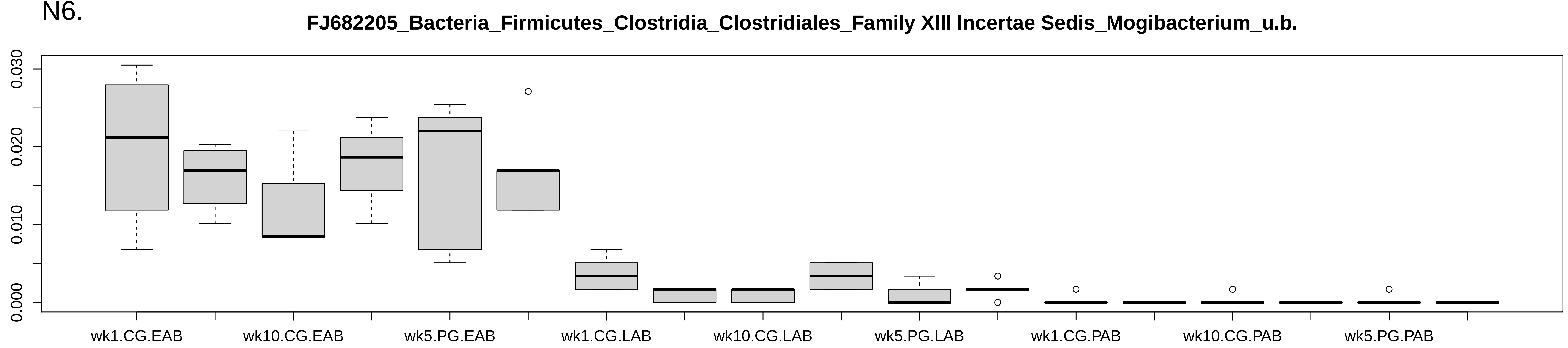

Statistics (p−values): Location: < 0.001; Group: 0.765; L×G: 1.000; Time: 0.227; L×T:0.537; G×T: 0.454; L×G×T: 0.766; Cow: 0.980; T×C: 0.868.

O1.

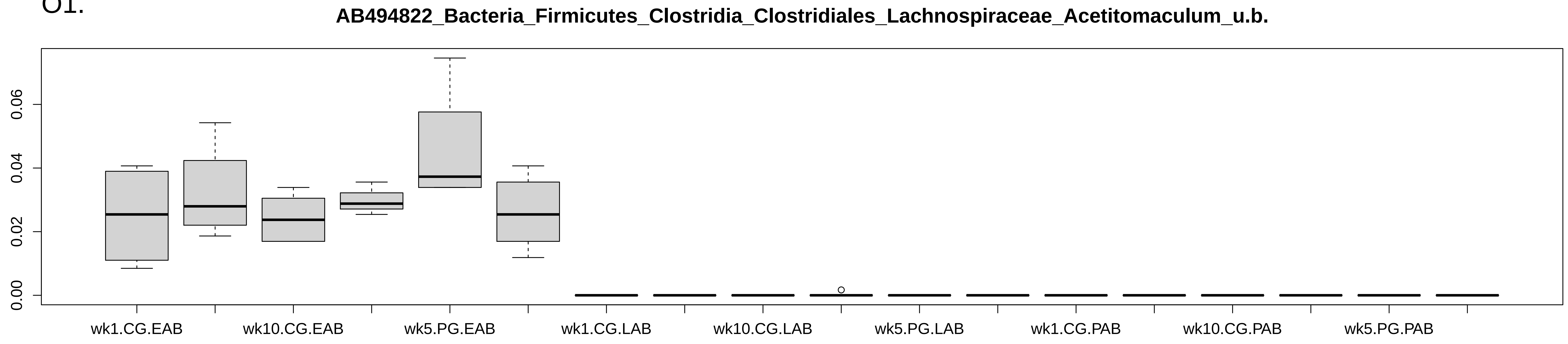

Statistics (p−values): Location: < 0.001; Group: 0.189; L×G: 0.257; Time: 0.017; L×T:0.006; G×T: 0.358; L×G×T: 0.406; Cow: 0.031; T×C: 0.120.

O2.

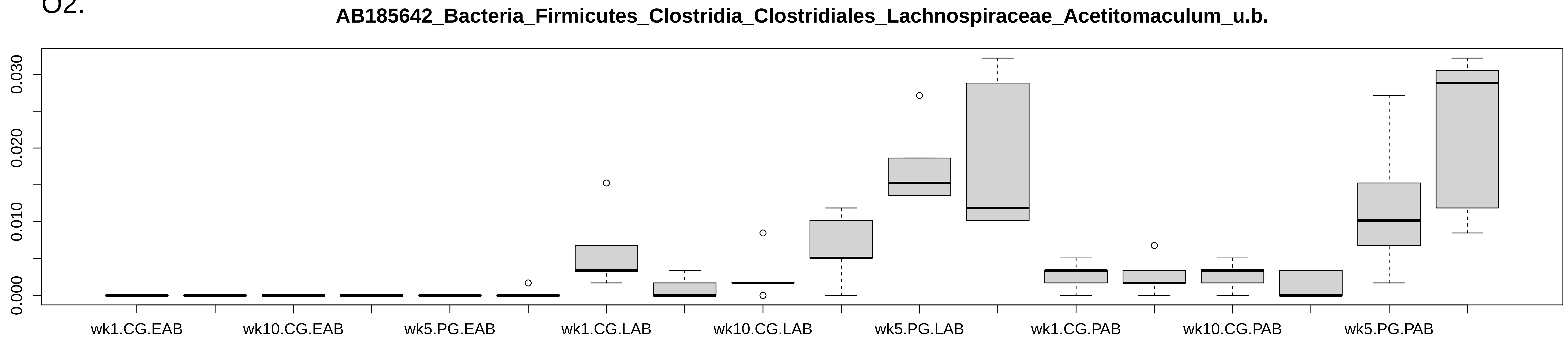

Statistics (p−values): Location: < 0.001; Group: < 0.001; L×G: < 0.001; Time: 0.002; L×T:0.050; G×T: < 0.001; L×G×T: 0.019; Cow: 0.745; T×C: 0.290.

O3.

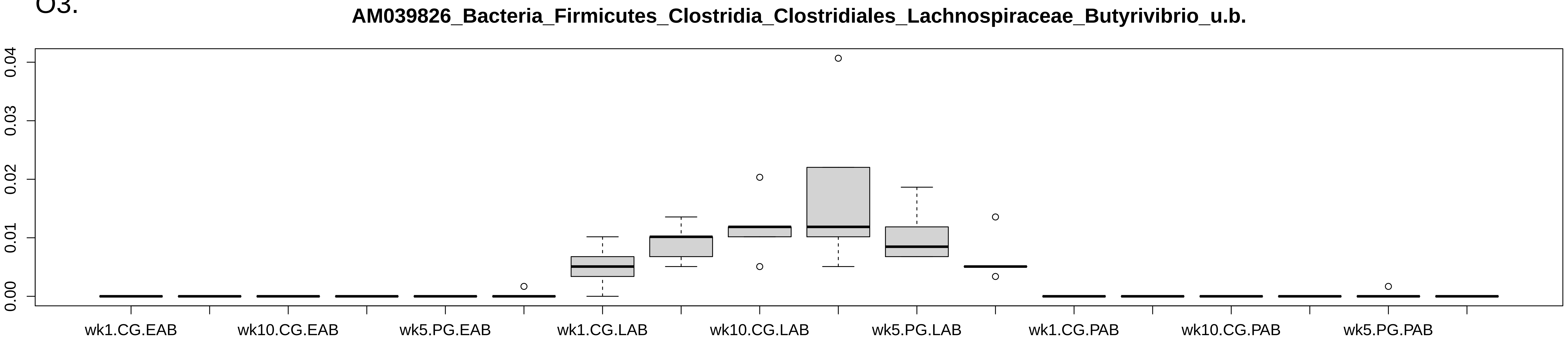

Statistics (p−values): Location: < 0.001; Group: 0.355; L×G: 0.577; Time: 1.000; L×T:1.000; G×T: 0.016; L×G×T: 0.001; Cow: 0.882; T×C: 1.000.

O4.

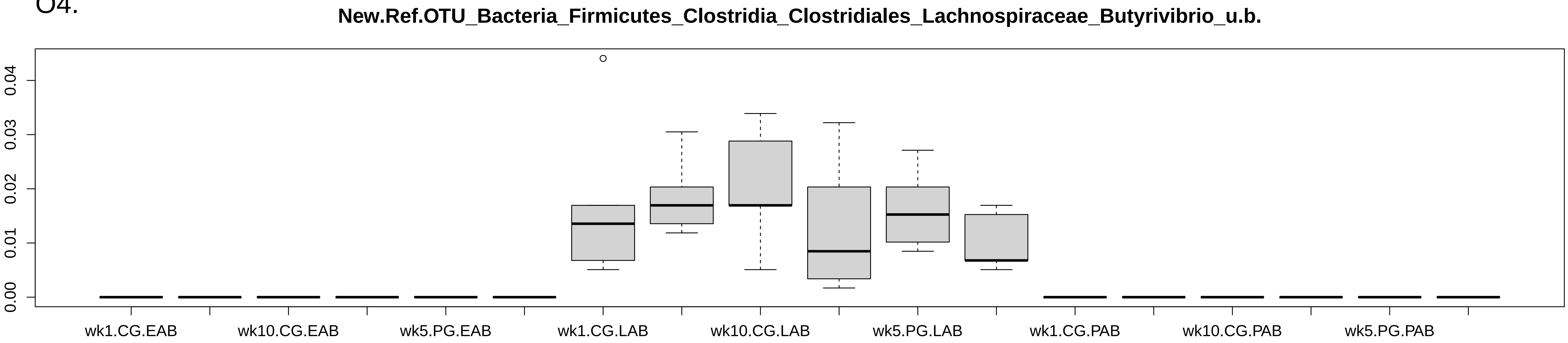

Statistics (p−values): Location: < 0.001; Group: 0.156; L×G: 0.243; Time: 1.000; L×T:1.000; G×T: 0.640; L×G×T: 0.669; Cow: 0.338; T×C: 1.000.

O5.

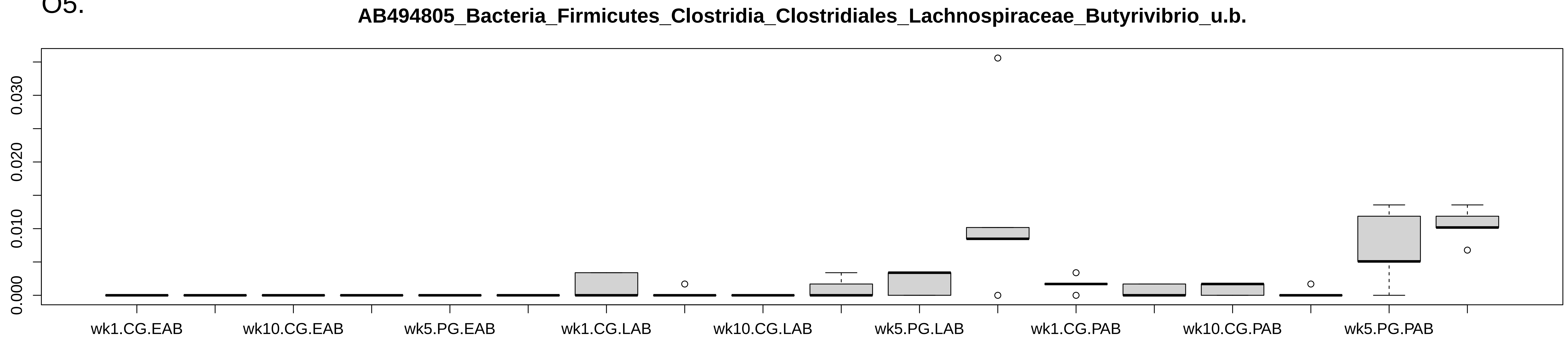

Statistics (p−values): Location: < 0.001; Group: < 0.001; L×G: 0.011; Time: 0.002; L×T:0.186; G×T: 0.002; L×G×T: 0.022; Cow: 0.368; T×C: 0.426.

O6.

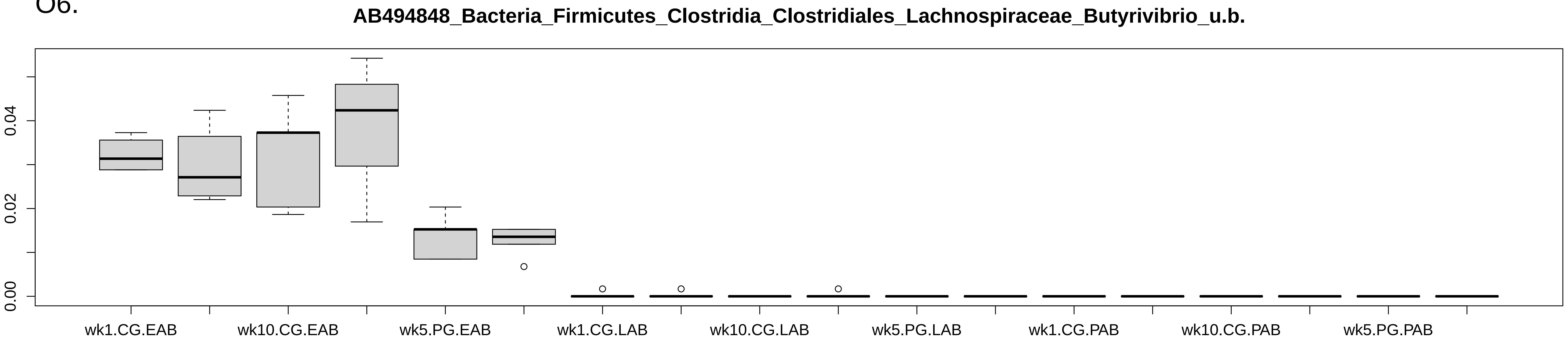

Statistics (p−values): Location: < 0.001; Group: < 0.001; L×G: 0.001; Time: 0.005; L×T:< 0.001; G×T: 0.006; L×G×T: 0.001; Cow: 0.240; T×C: 0.852.

O7.

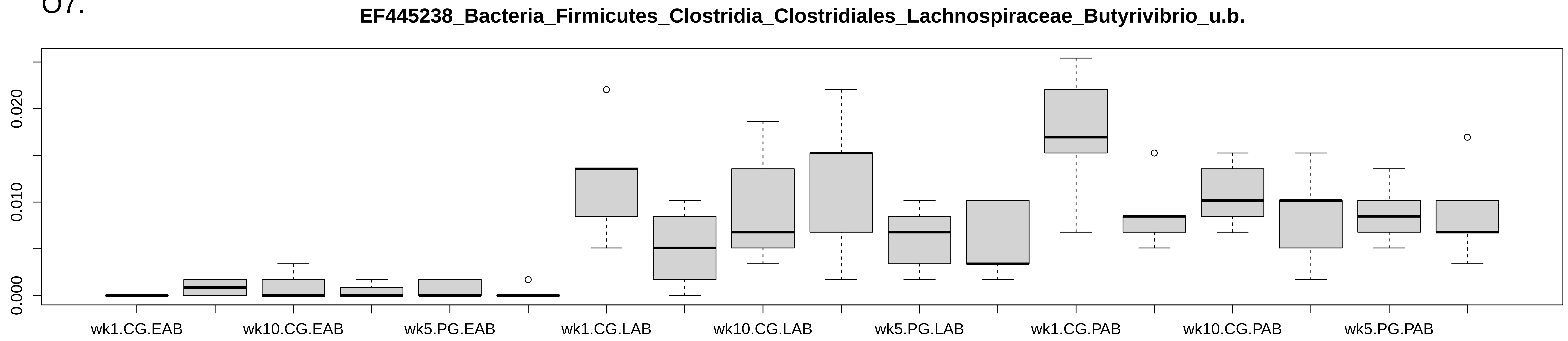

Statistics (p−values): Location: < 0.001; Group: 0.131; L×G: 0.408; Time: 0.008; L×T:0.240; G×T: 0.208; L×G×T: 0.396; Cow: 0.137; T×C: 0.271.

O8.

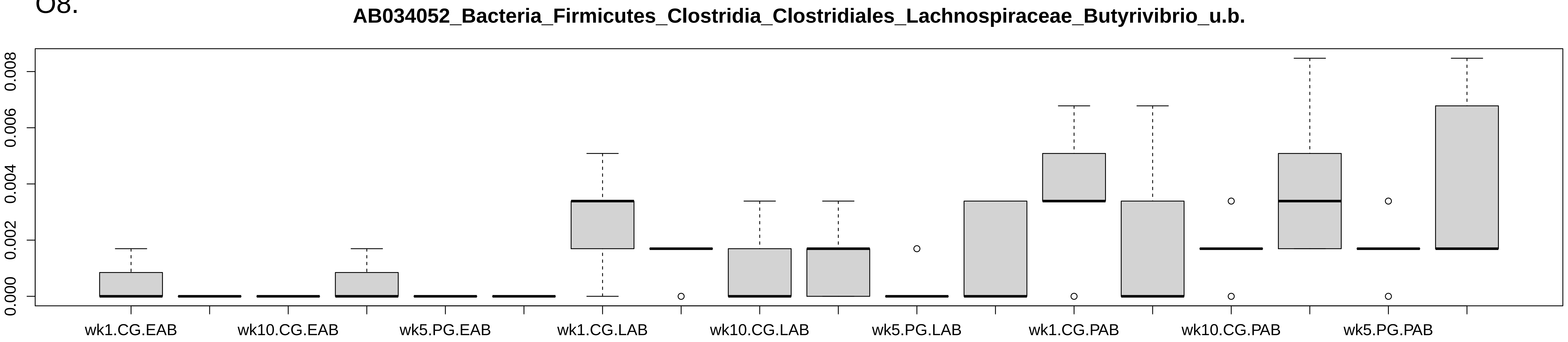

Statistics (p−values): Location: < 0.001; Group: 0.706; L×G: 0.183; Time: 0.029; L×T:0.760; G×T: 0.335; L×G×T: 0.739; Cow: 1.000; T×C: 0.510.

O9.

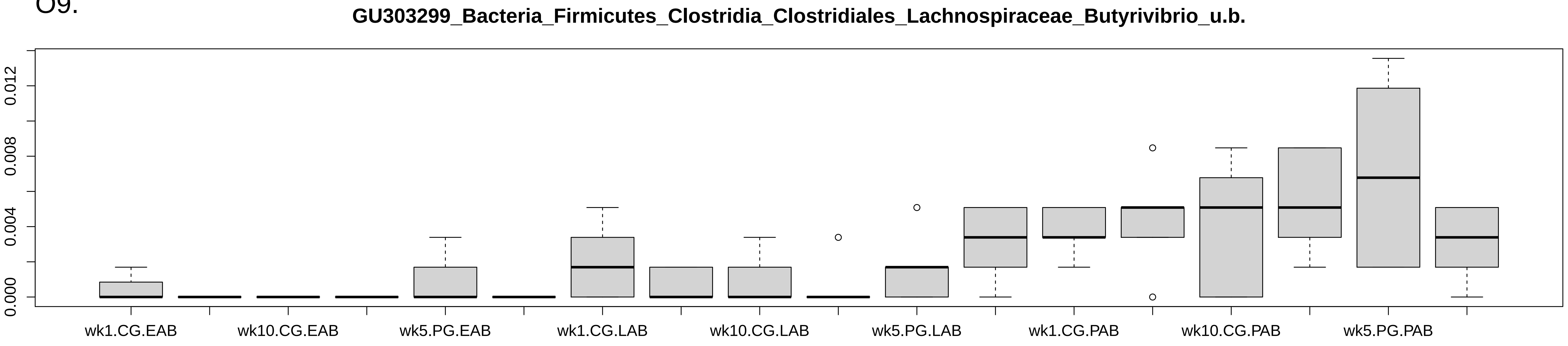

Statistics (p−values): Location: < 0.001; Group: 0.432; L×G: 0.936; Time: 0.698; L×T:0.407; G×T: 0.298; L×G×T: 0.296; Cow: 0.082; T×C: 0.335.

O10.

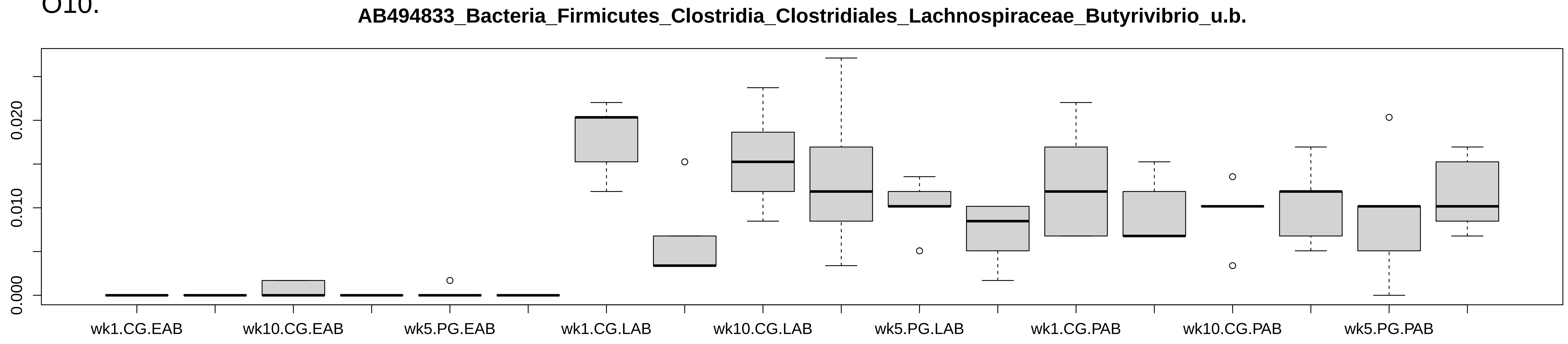

Statistics (p−values): Location: < 0.001; Group: 0.113; L×G: 0.401; Time: 0.024; L×T:0.154; G×T: 0.174; L×G×T: 0.124; Cow: 0.293; T×C: 0.156.

O11.

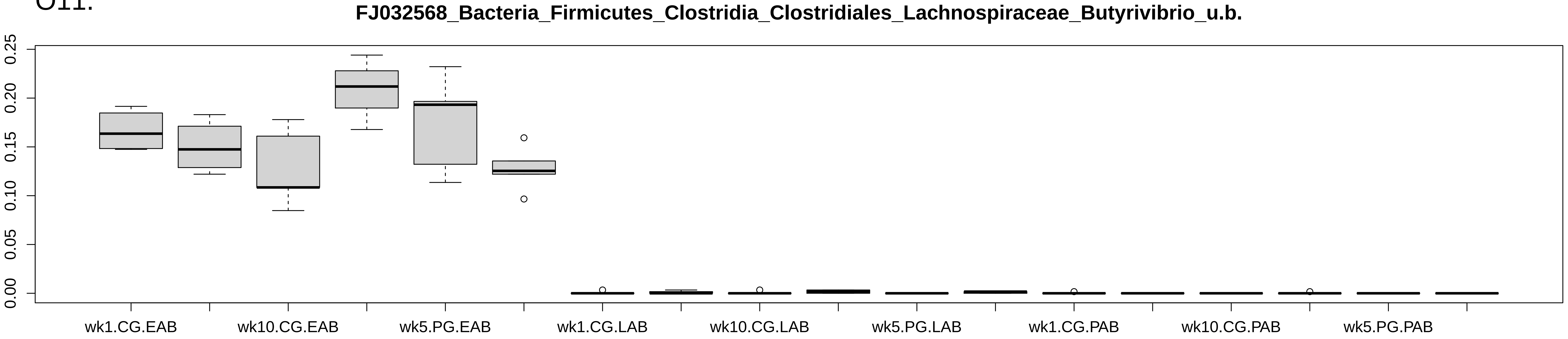

Statistics (p−values): Location: < 0.001; Group: 0.089; L×G: 0.082; Time: < 0.001; L×T:< 0.001; G×T: 0.346; L×G×T: 0.359; Cow: 0.882; T×C: 0.199.

O13.

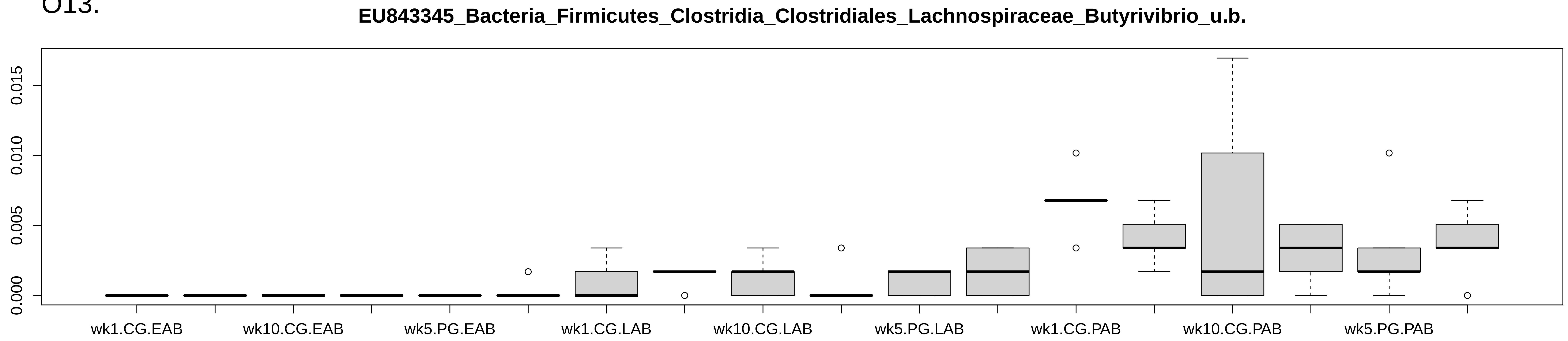

Statistics (p−values): Location: < 0.001; Group: 0.190; L×G: 0.262; Time: 0.928; L×T:0.922; G×T: 0.701; L×G×T: 0.989; Cow: 0.540; T×C: 0.468.

O14.

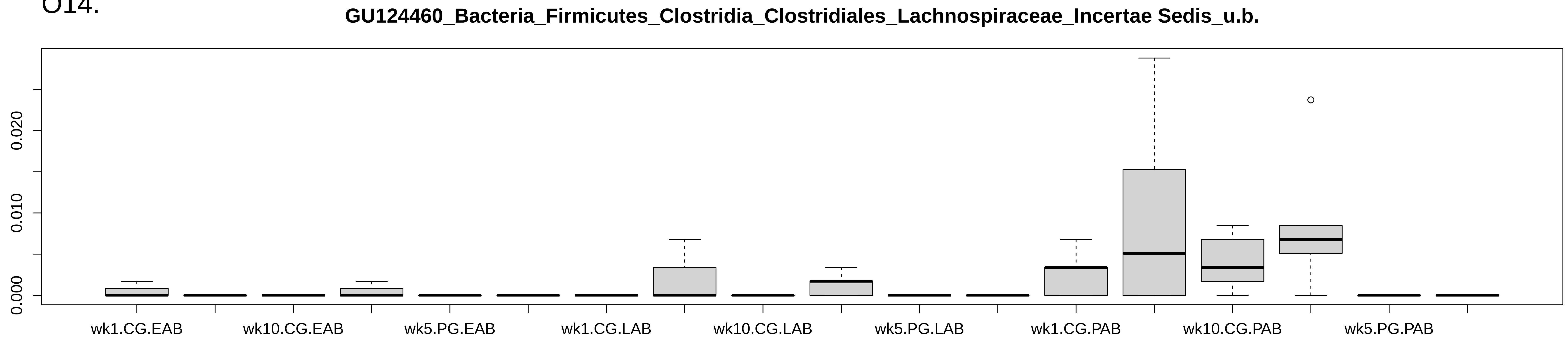

Statistics (p−values): Location: < 0.001; Group: 0.116; L×G: 0.408; Time: 0.161; L×T:0.764; G×T: < 0.001; L×G×T: 0.035; Cow: 0.922; T×C: 0.240.

O15.

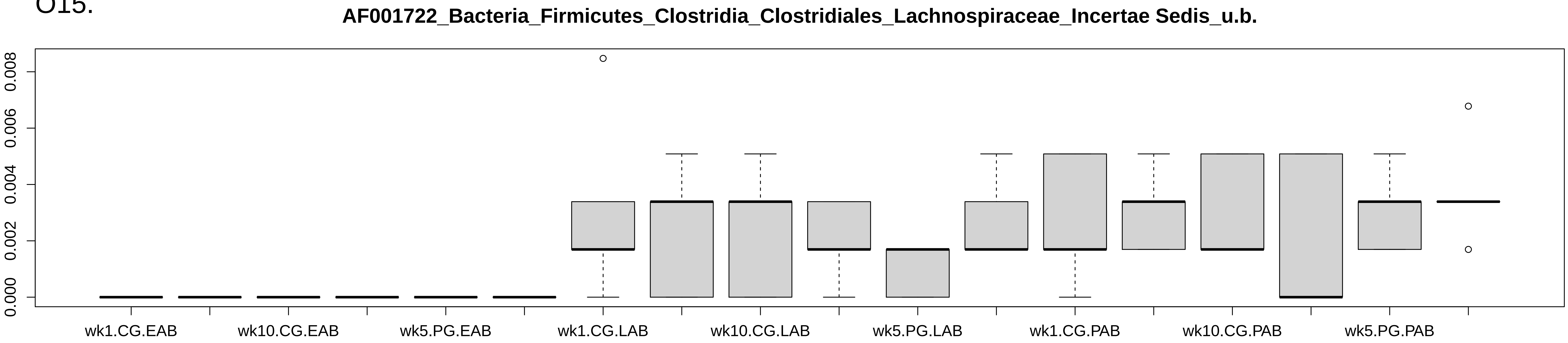

Statistics (p−values): Location: < 0.001; Group: 0.824; L×G: 0.830; Time: 0.642; L×T:0.574; G×T: 0.369; L×G×T: 0.993; Cow: 0.394; T×C: 0.585.

O16.

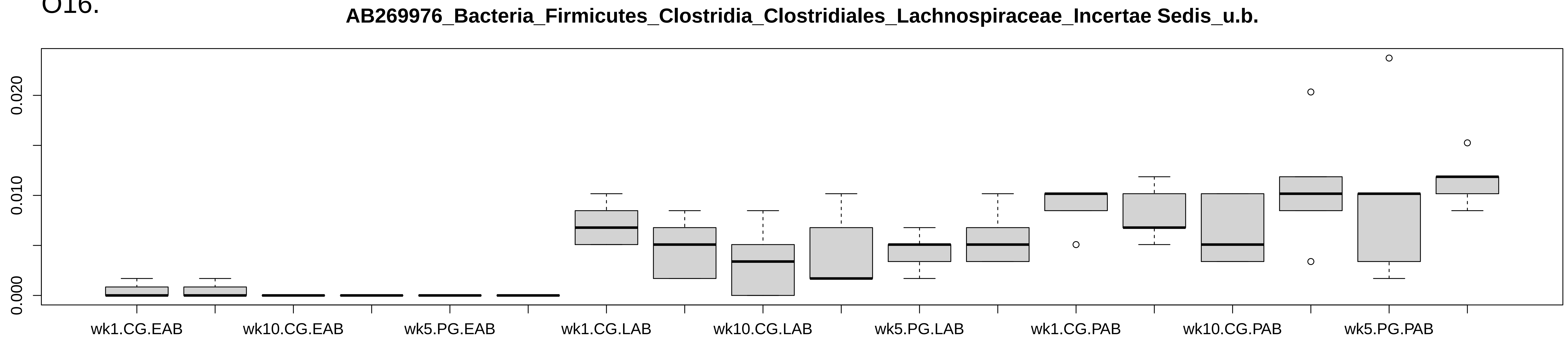

Statistics (p−values): Location: < 0.001; Group: 0.342; L×G: 0.121; Time: 0.686; L×T:1.000; G×T: 0.242; L×G×T: 0.842; Cow: 0.482; T×C: 0.029.

O17.

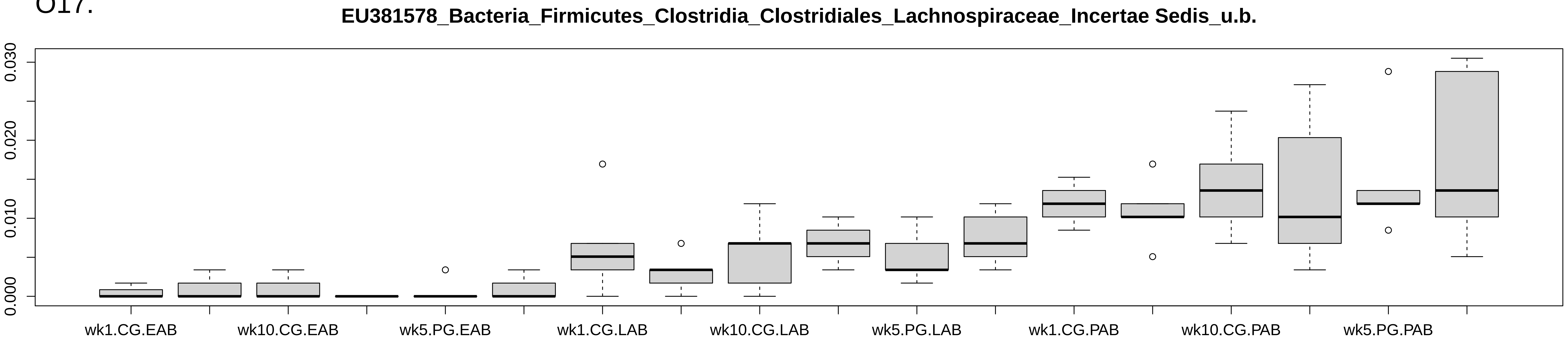

Statistics (p−values): Location: < 0.001; Group: 0.239; L×G: 0.648; Time: 0.198; L×T:0.563; G×T: 1.000; L×G×T: 1.000; Cow: 0.222; T×C: 0.193.

O18.

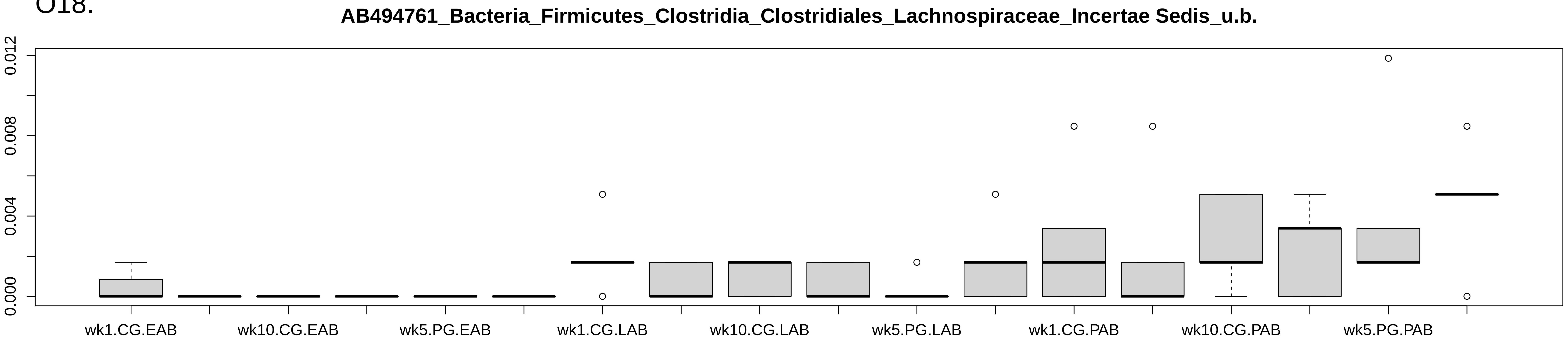

Statistics (p−values): Location: < 0.001; Group: 0.765; L×G: 0.700; Time: 0.641; L×T:0.965; G×T: 0.363; L×G×T: 1.000; Cow: 0.321; T×C: 0.045.

O19.

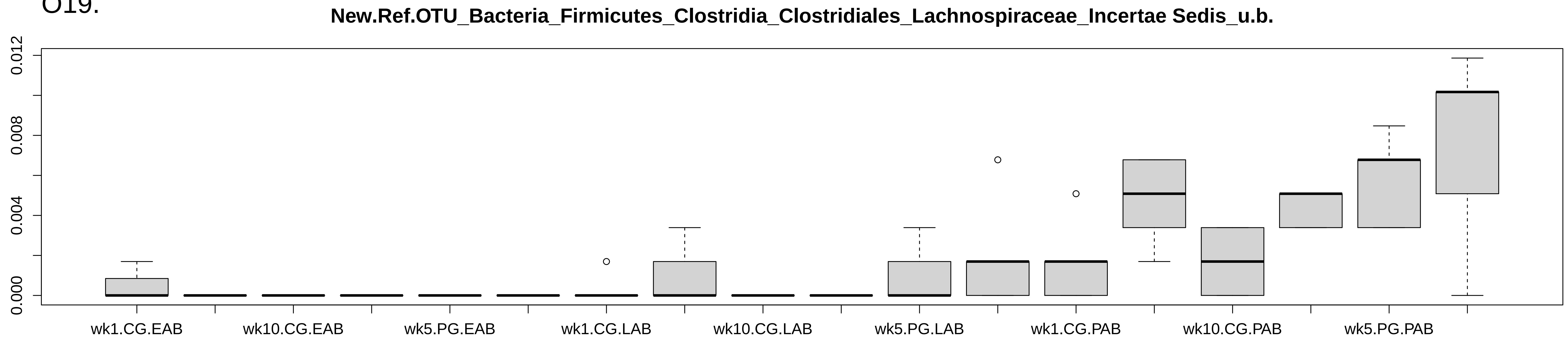

Statistics (p−values): Location: < 0.001; Group: 0.004; L×G: 0.000; Time: 0.149; L×T:0.393; G×T: 0.045; L×G×T: 0.328; Cow: 0.941; T×C: 0.340.

O20.

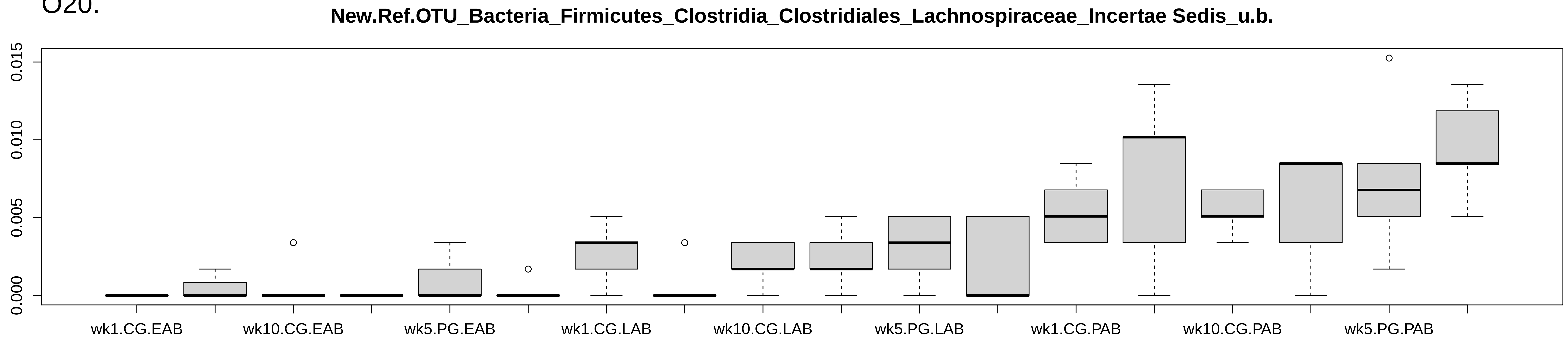

Statistics (p−values): Location: < 0.001; Group: 0.279; L×G: 0.488; Time: 0.526; L×T:0.525; G×T: 1.000; L×G×T: 0.365; Cow: 0.202; T×C: 0.285.

O21.

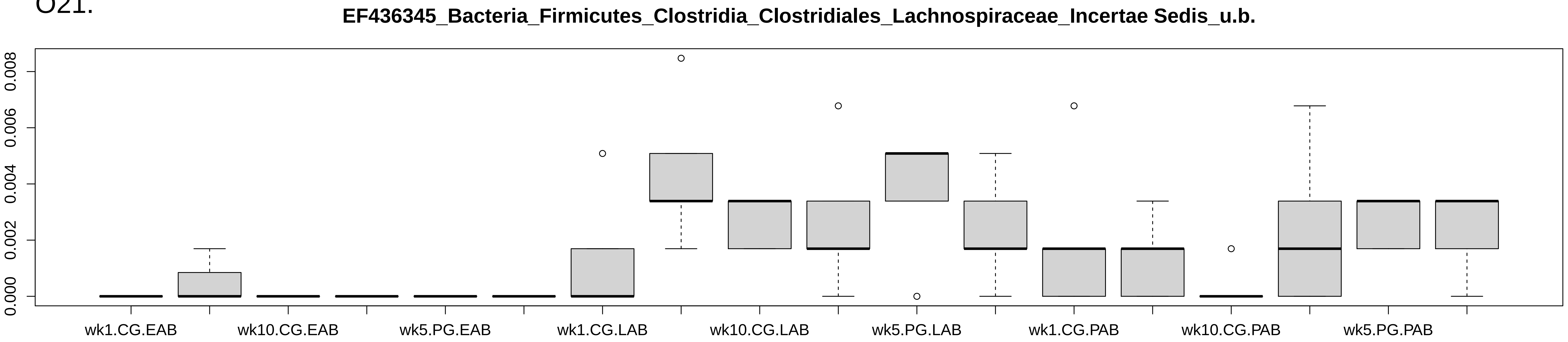

Statistics (p−values): Location: < 0.001; Group: 0.606; L×G: 0.189; Time: 0.241; L×T:0.226; G×T: 1.000; L×G×T: 0.684; Cow: 1.000; T×C: 1.000.

O22.

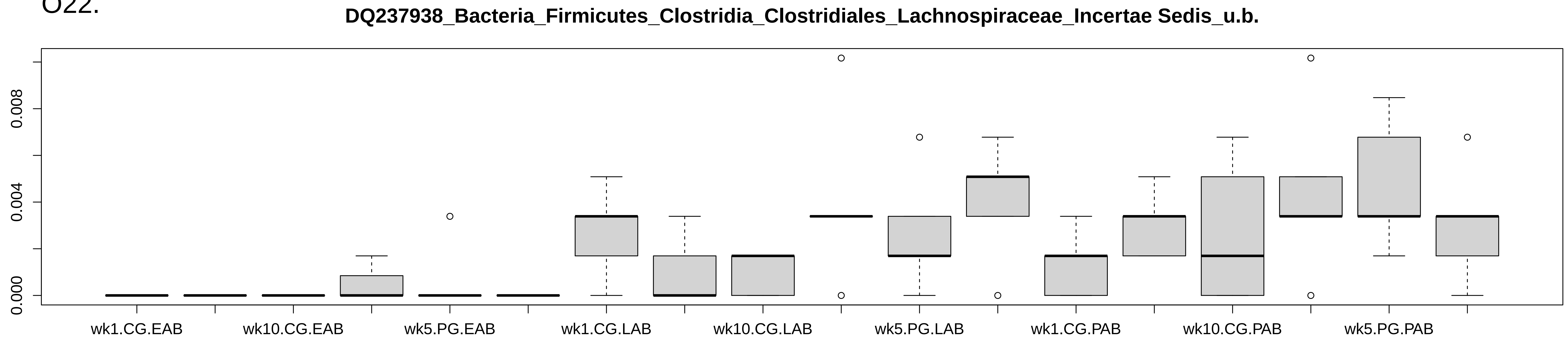

Statistics (p−values): Location: < 0.001; Group: 0.003; L×G: 0.299; Time: 1.000; L×T:0.477; G×T: 0.902; L×G×T: 0.454; Cow: 0.902; T×C: 0.062.

O23.

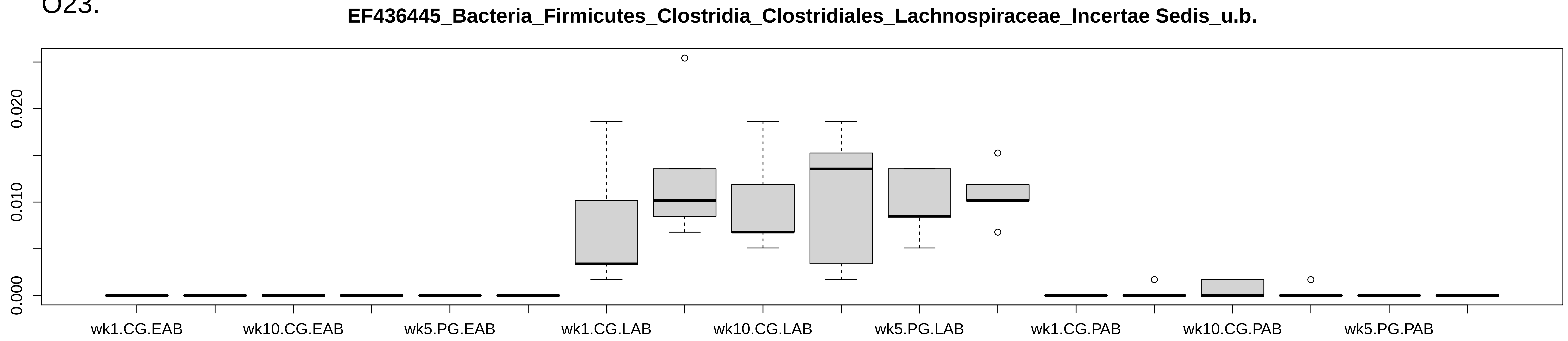

Statistics (p−values): Location: < 0.001; Group: 0.941; L×G: 0.882; Time: 0.843; L×T:0.887; G×T: 0.849; L×G×T: 0.484; Cow: 0.122; T×C: 0.968.

O24.

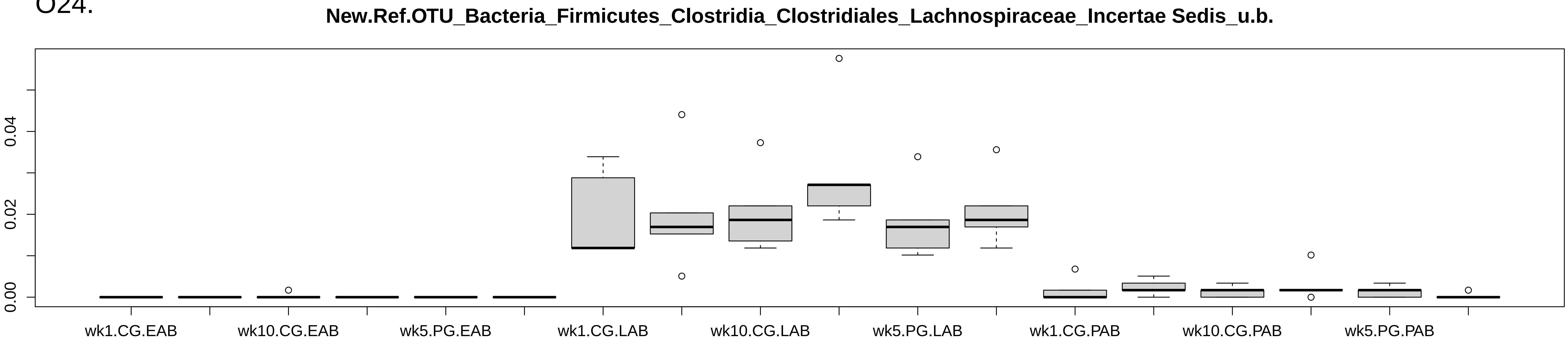

Statistics (p−values): Location: < 0.001; Group: 0.980; L×G: 0.434; Time: 0.804; L×T:0.629; G×T: 0.385; L×G×T: 0.592; Cow: 0.032; T×C: 0.500.

O25.

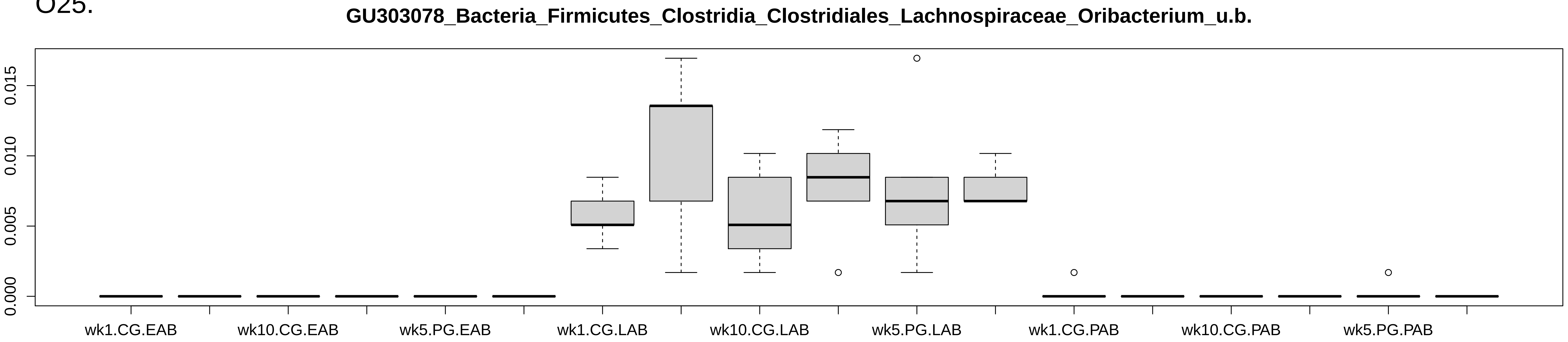

Statistics (p−values): Location: < 0.001; Group: 0.655; L×G: 0.902; Time: 0.458; L×T:0.315; G×T: 0.953; L×G×T: 0.508; Cow: 0.980; T×C: 0.393.

O26.

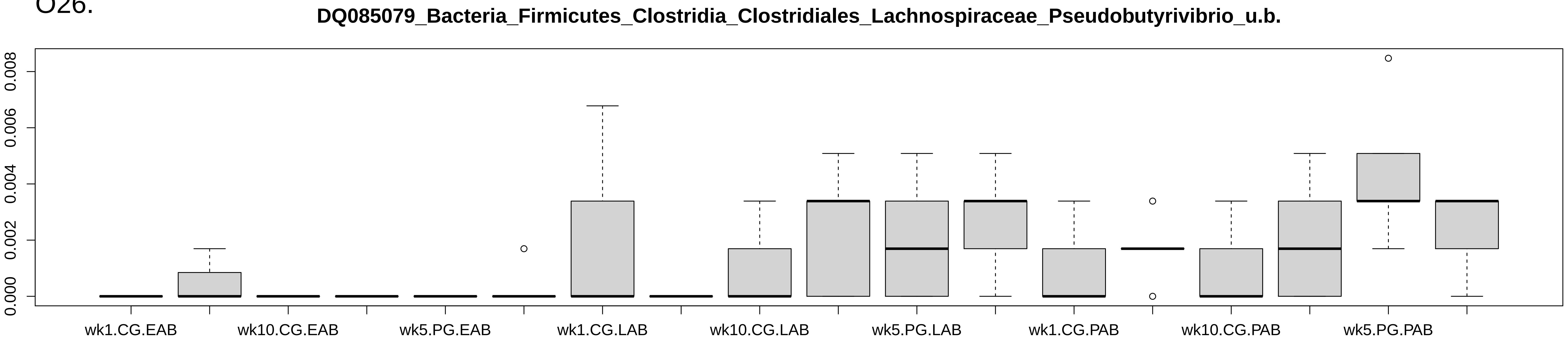

Statistics (p−values): Location: < 0.001; Group: 0.018; L×G: 0.116; Time: 0.981; L×T:0.075; G×T: 0.376; L×G×T: 1.000; Cow: 0.473; T×C: 0.601.

O27.

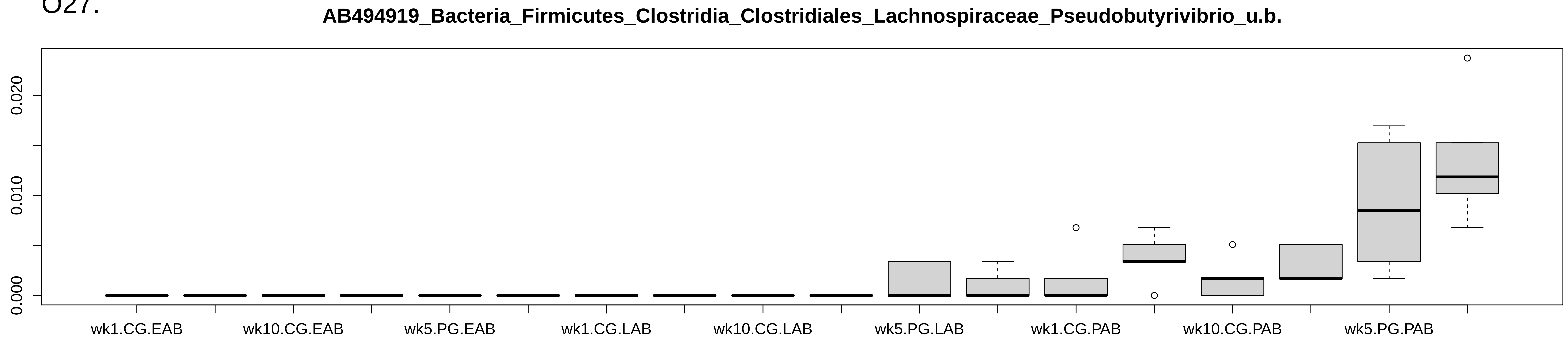

Statistics (p−values): Location: < 0.001; Group: < 0.001; L×G: < 0.001; Time: 0.021; L×T:0.011; G×T: 0.042; L×G×T: 0.026; Cow: 0.363; T×C: 0.980.

O28.

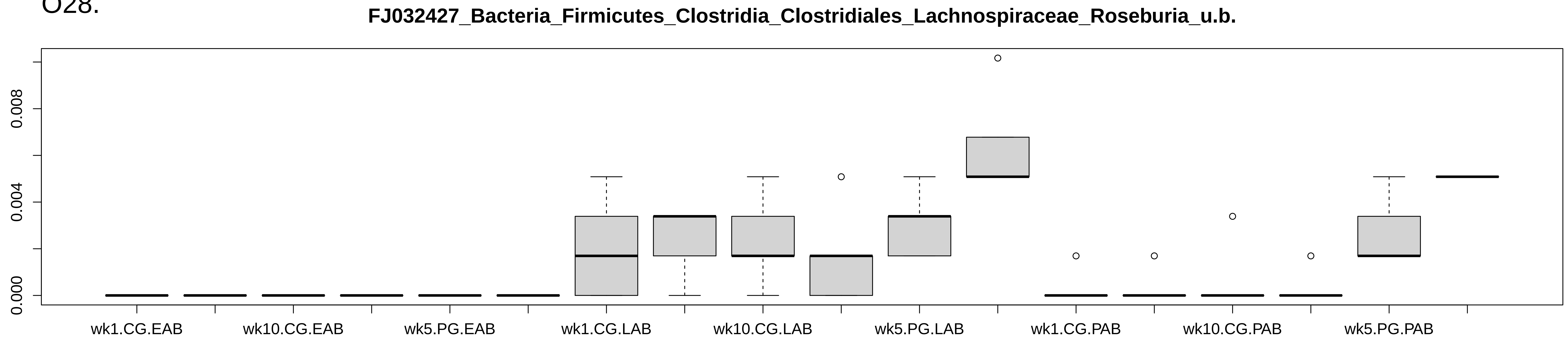

Statistics (p−values): Location: < 0.001; Group: < 0.001; L×G: 0.010; Time: < 0.001; L×T:0.051; G×T: < 0.001; L×G×T: 0.078; Cow: 0.322; T×C: 0.956.

O29.

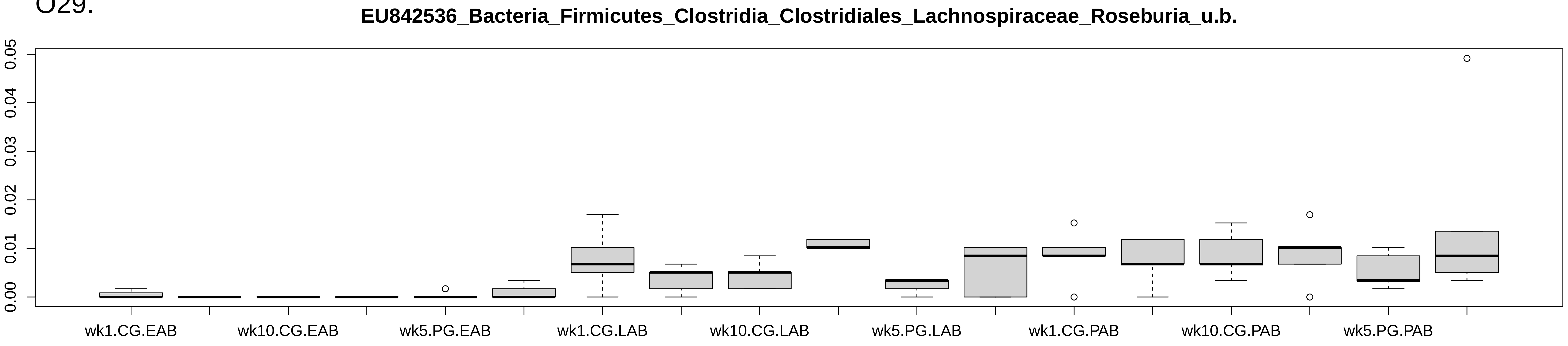

Statistics (p−values): Location: < 0.001; Group: 0.540; L×G: 0.660; Time: 0.080; L×T:0.086; G×T: 0.475; L×G×T: 0.850; Cow: 0.244; T×C: 1.000.

O30.

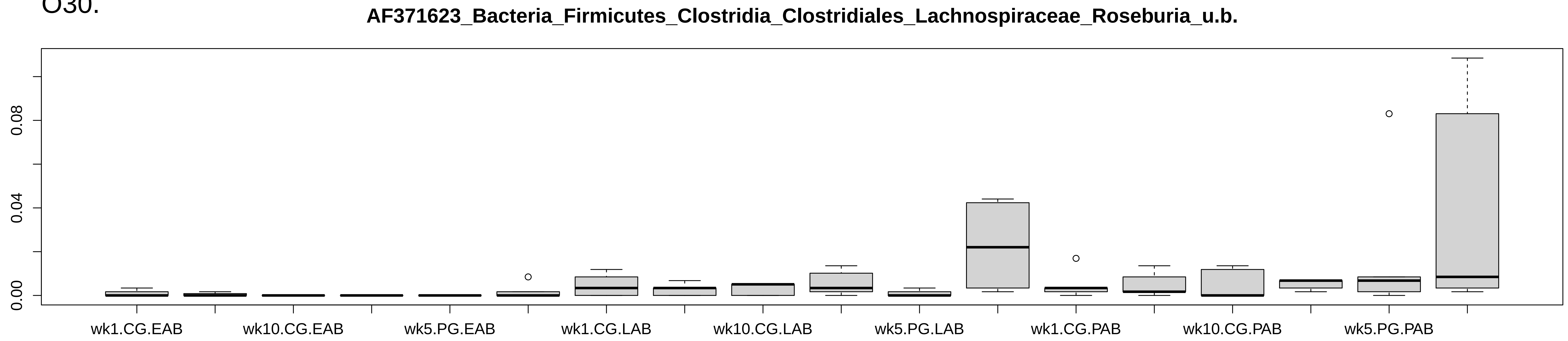

Statistics (p−values): Location: 0.007; Group: 0.045; L×G: 0.098; Time: 0.076; L×T:1.000; G×T: 0.043; L×G×T: 0.577; Cow: 0.100; T×C: 0.499.

O31.

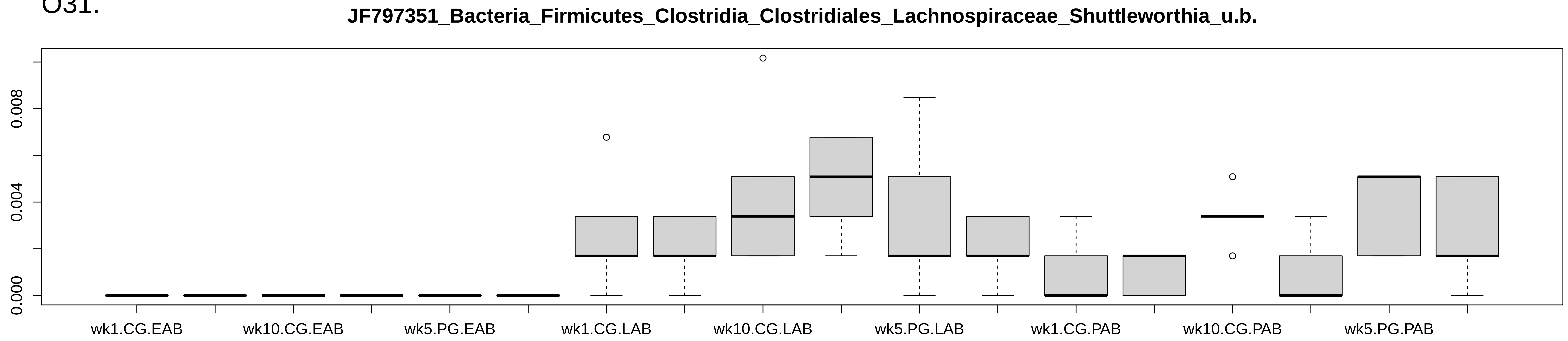

Statistics (p−values): Location: < 0.001; Group: 0.961; L×G: 0.520; Time: 0.412; L×T:0.211; G×T: 0.006; L×G×T: 0.199; Cow: 0.462; T×C: 0.980.

O32.

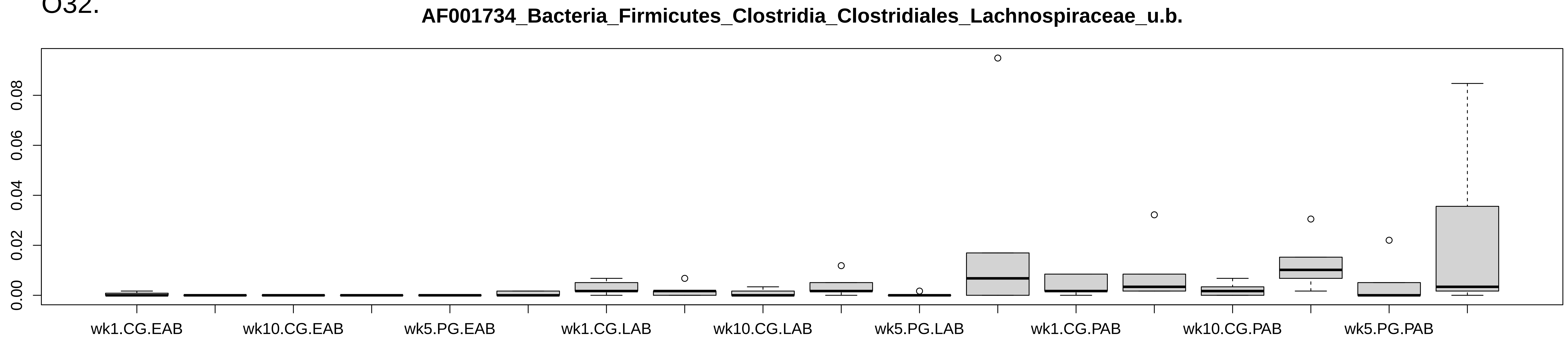

Statistics (p−values): Location: 0.032; Group: 0.045; L×G: 0.812; Time: 0.178; L×T:0.961; G×T: 0.060; L×G×T: 0.389; Cow: 0.505; T×C: 0.637.

O33.

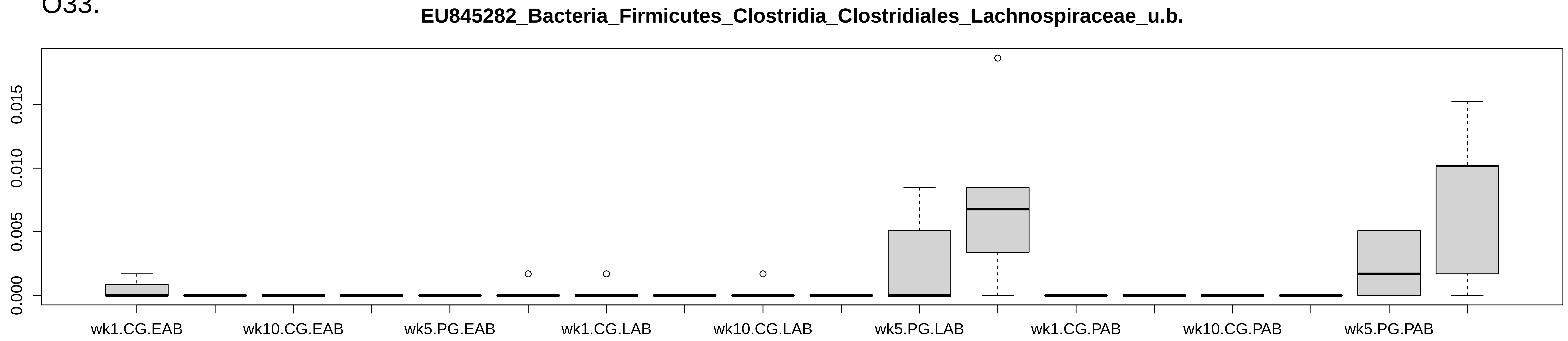

Statistics (p−values): Location: 0.033; Group: < 0.001; L×G: 0.052; Time: < 0.001; L×T:0.246; G×T: < 0.001; L×G×T: 0.324; Cow: 1.000; T×C: 1.000.

O34.

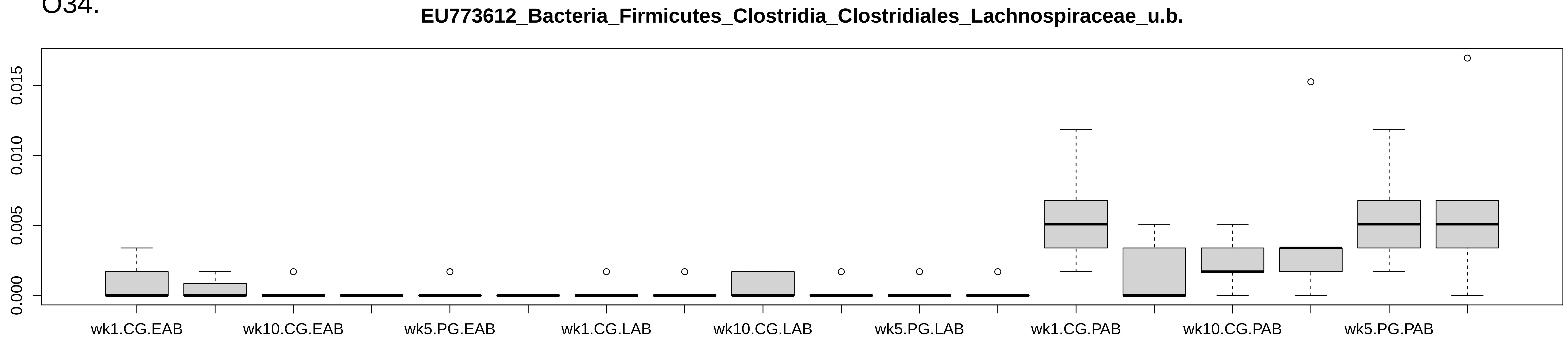

Statistics (p−values): Location: < 0.001; Group: 1.000; L×G: 0.068; Time: 0.634; L×T:0.962; G×T: 0.330; L×G×T: 0.900; Cow: 0.024; T×C: 0.667.

O35.

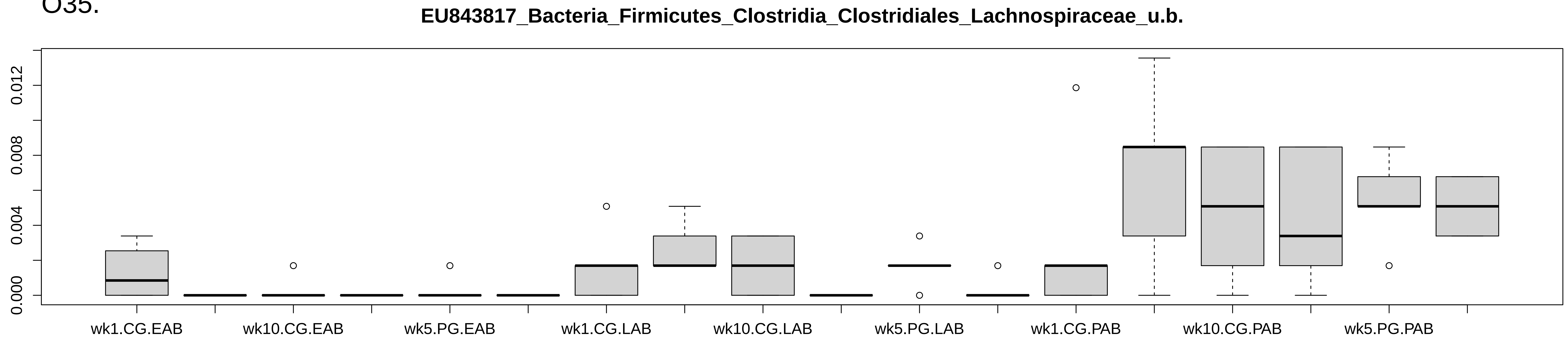

Statistics (p−values): Location: < 0.001; Group: 0.922; L×G: 0.572; Time: 0.312; L×T:0.621; G×T: 1.000; L×G×T: 0.917; Cow: 0.961; T×C: 0.788.

O36.

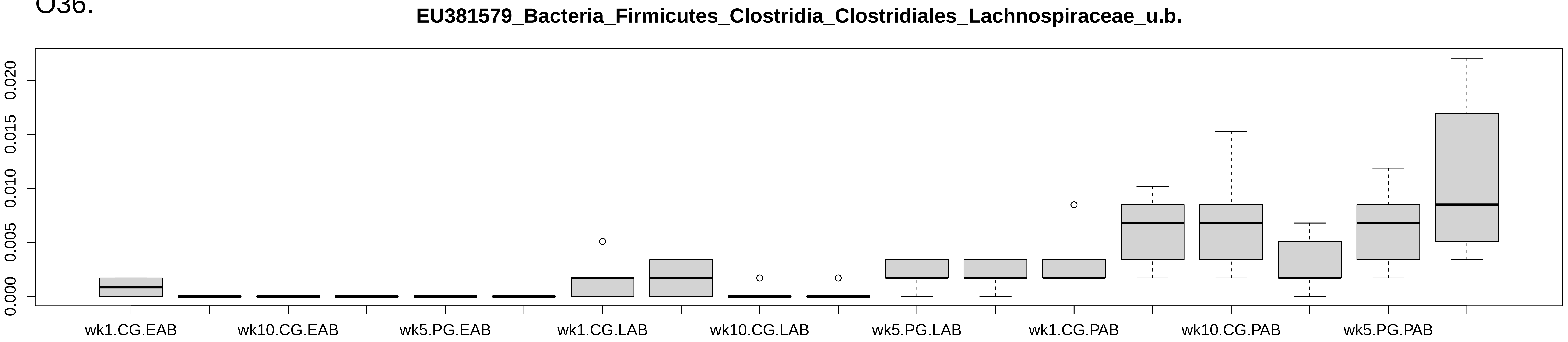

Statistics (p−values): Location: < 0.001; Group: 0.315; L×G: 0.848; Time: 0.080; L×T:0.012; G×T: 0.349; L×G×T: 0.842; Cow: 0.863; T×C: 0.465.

O37.

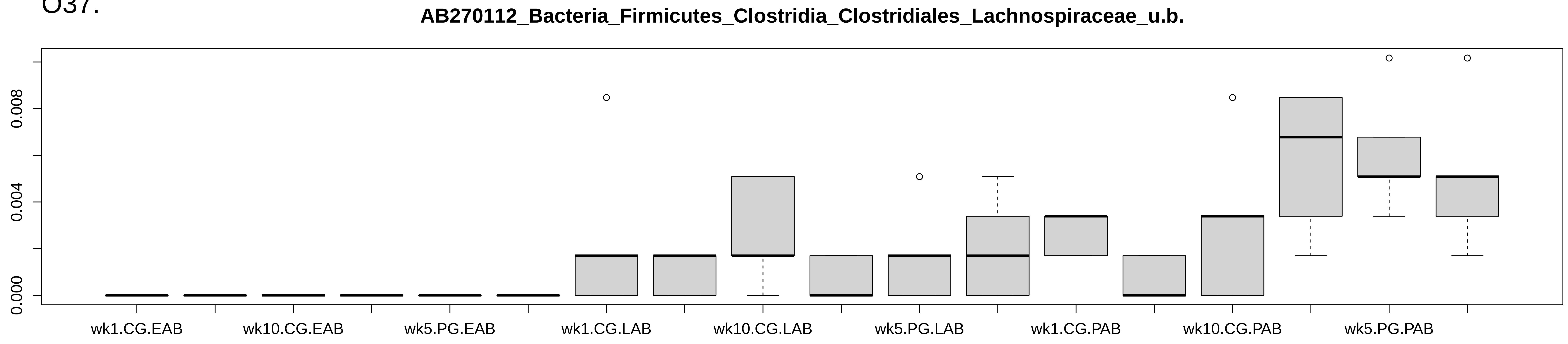

Statistics (p−values): Location: < 0.001; Group: 0.053; L×G: < 0.001; Time: 0.557; L×T:0.987; G×T: 0.196; L×G×T: 0.488; Cow: 0.473; T×C: 0.850.

O38.

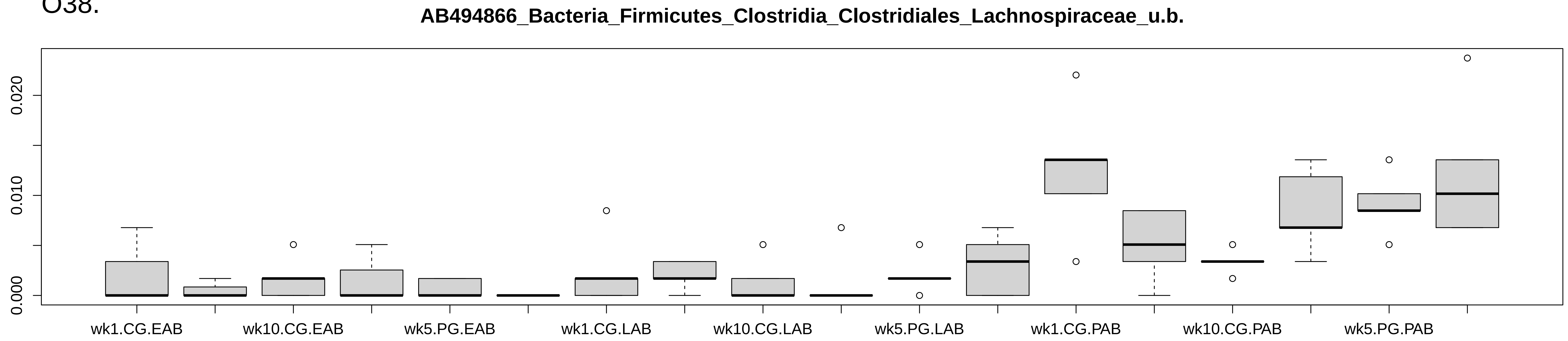

Statistics (p−values): Location: < 0.001; Group: 0.333; L×G: 0.041; Time: 0.333; L×T:0.527; G×T: 0.005; L×G×T: 0.021; Cow: 0.000; T×C: 0.170.

O39.

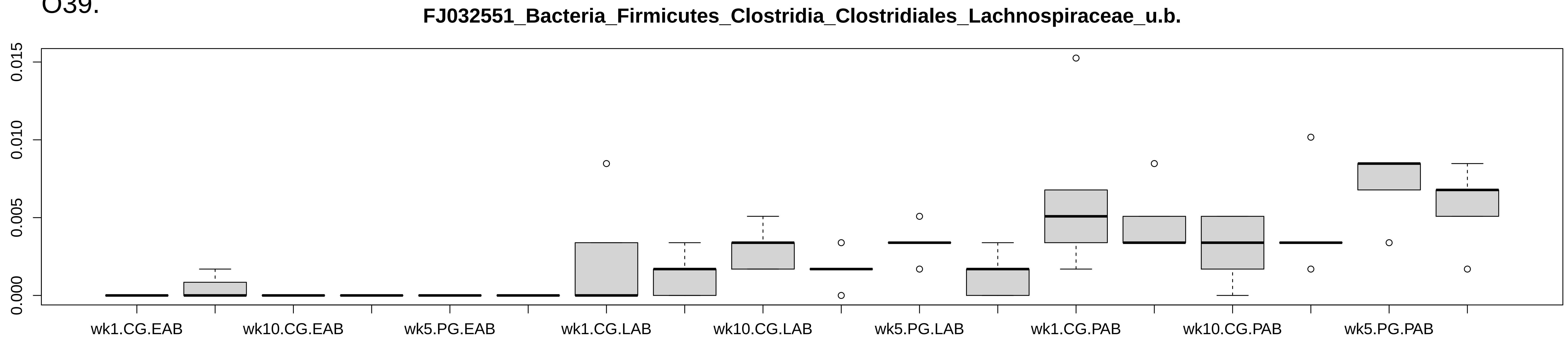

Statistics (p−values): Location: < 0.001; Group: 0.655; L×G: 0.323; Time: 0.702; L×T:0.752; G×T: 0.165; L×G×T: 0.177; Cow: 0.180; T×C: 0.493.

O40.

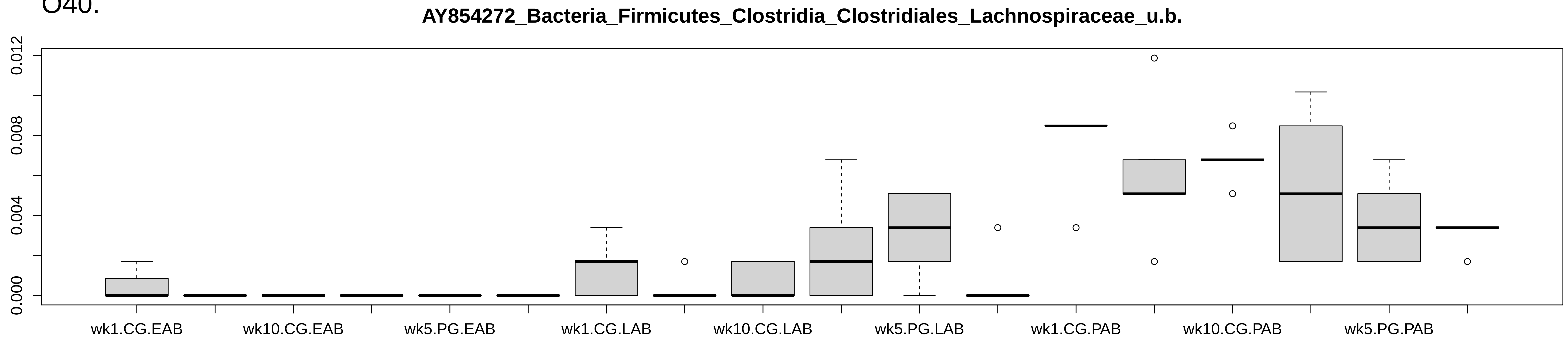

Statistics (p−values): Location: < 0.001; Group: 0.142; L×G: < 0.001; Time: 0.121; L×T:0.767; G×T: 0.207; L×G×T: 0.639; Cow: 1.000; T×C: 0.356.

O41.

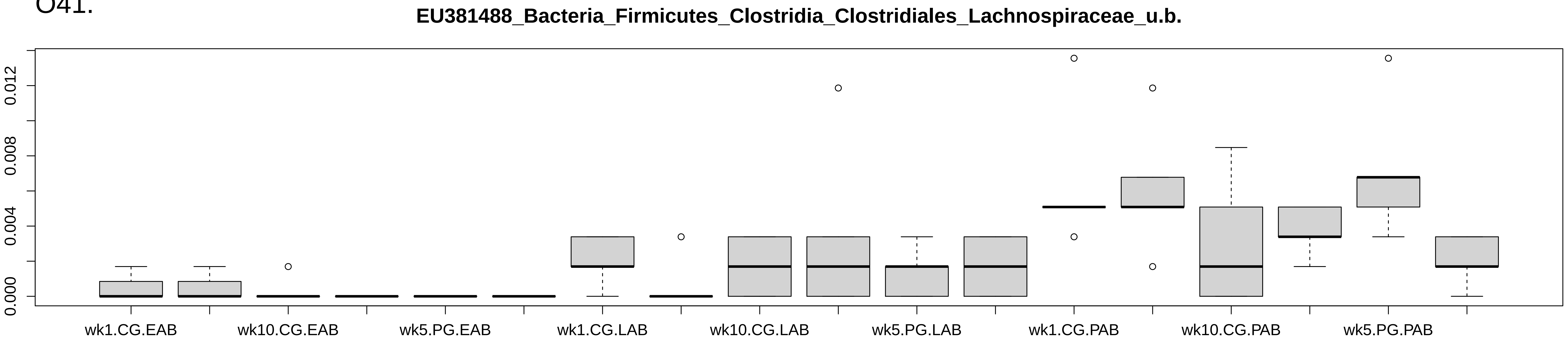

Statistics (p−values): Location: < 0.001; Group: 0.548; L×G: 0.502; Time: 0.092; L×T:0.017; G×T: 0.968; L×G×T: 0.731; Cow: 0.902; T×C: 0.766.

O42.

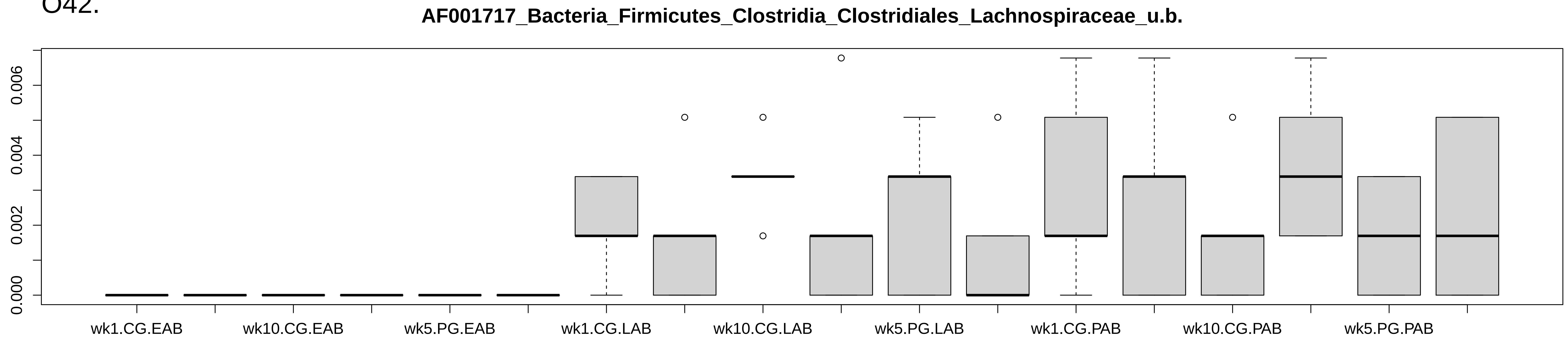

Statistics (p−values): Location: < 0.001; Group: 0.726; L×G: 0.897; Time: 1.000; L×T:0.556; G×T: 0.716; L×G×T: 0.542; Cow: 0.594; T×C: 0.746.

O43.

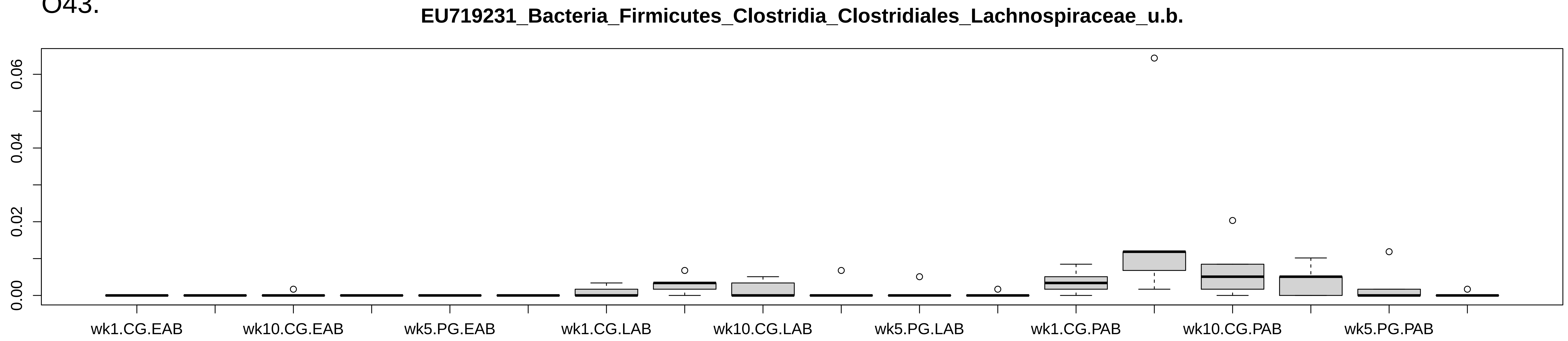

Statistics (p−values): Location: 0.001; Group: 0.016; L×G: 0.057; Time: 0.362; L×T:0.180; G×T: 0.207; L×G×T: 0.321; Cow: 0.706; T×C: 0.556.

O44.

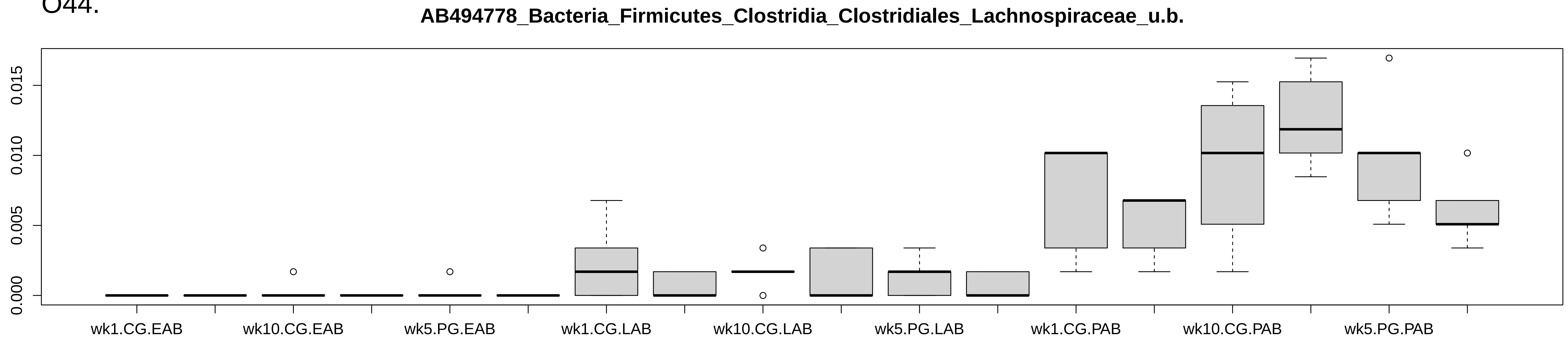

Statistics (p−values): Location: < 0.001; Group: 0.156; L×G: 0.320; Time: 0.376; L×T:0.680; G×T: 0.017; L×G×T: 0.089; Cow: 0.170; T×C: 0.009.

O45.

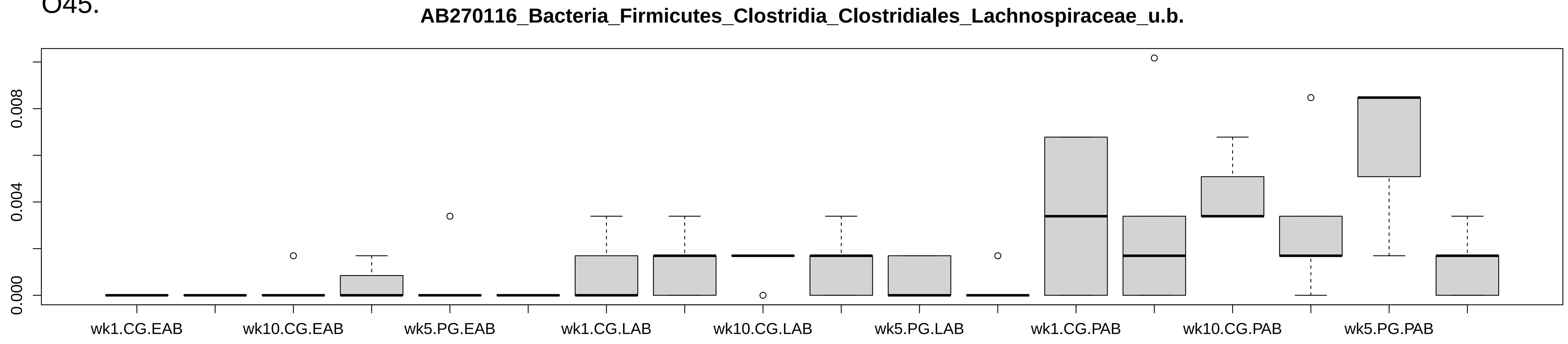

Statistics (p−values): Location: < 0.001; Group: 1.000; L×G: 0.741; Time: 0.482; L×T:0.383; G×T: 0.039; L×G×T: 0.050; Cow: 1.000; T×C: 1.000.

O46.

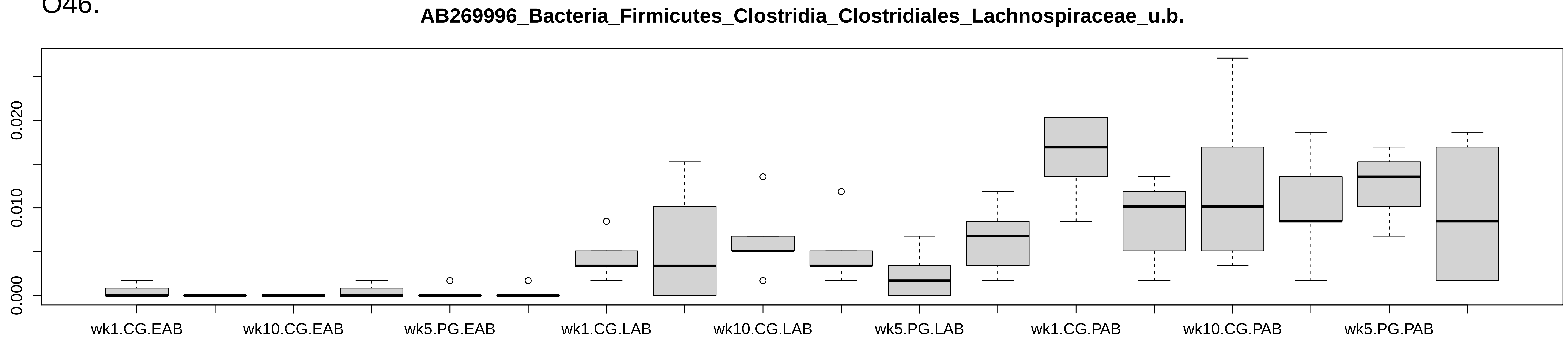

Statistics (p−values): Location: < 0.001; Group: 0.321; L×G: 0.756; Time: 1.000; L×T:0.701; G×T: 0.709; L×G×T: 0.213; Cow: 0.902; T×C: 0.478.

O47.

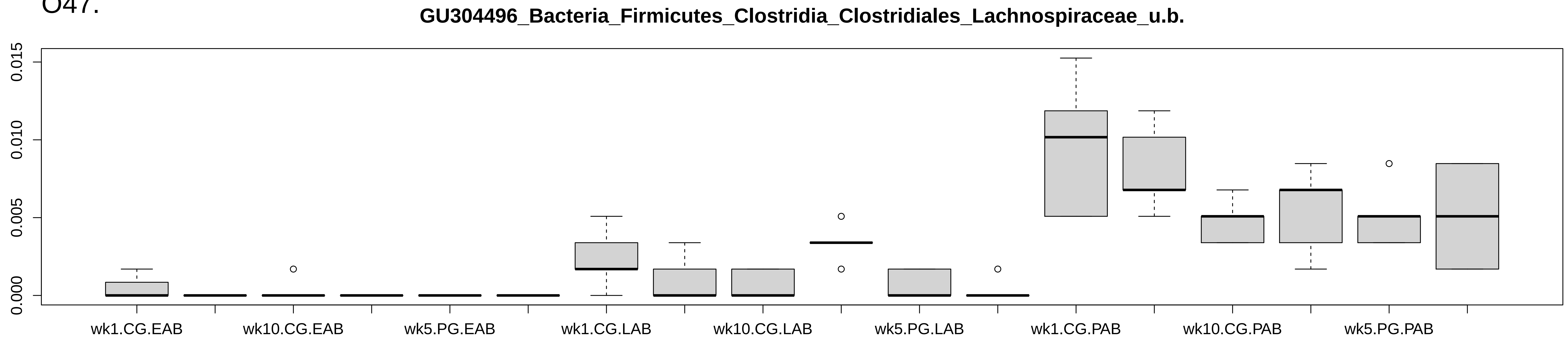

Statistics (p−values): Location: < 0.001; Group: 0.061; L×G: 0.050; Time: 0.005; L×T:0.178; G×T: 0.843; L×G×T: 0.121; Cow: 1.000; T×C: 0.420.

O48.

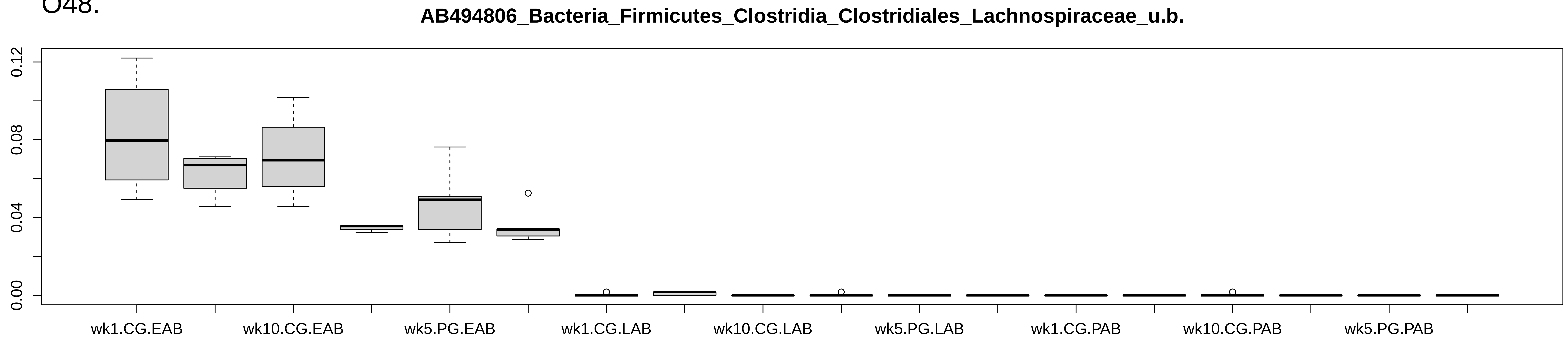

Statistics (p−values): Location: < 0.001; Group: < 0.001; L×G: < 0.001; Time: 0.762; L×T:0.955; G×T: 0.179; L×G×T: 0.123; Cow: 0.824; T×C: 0.337.

P1.

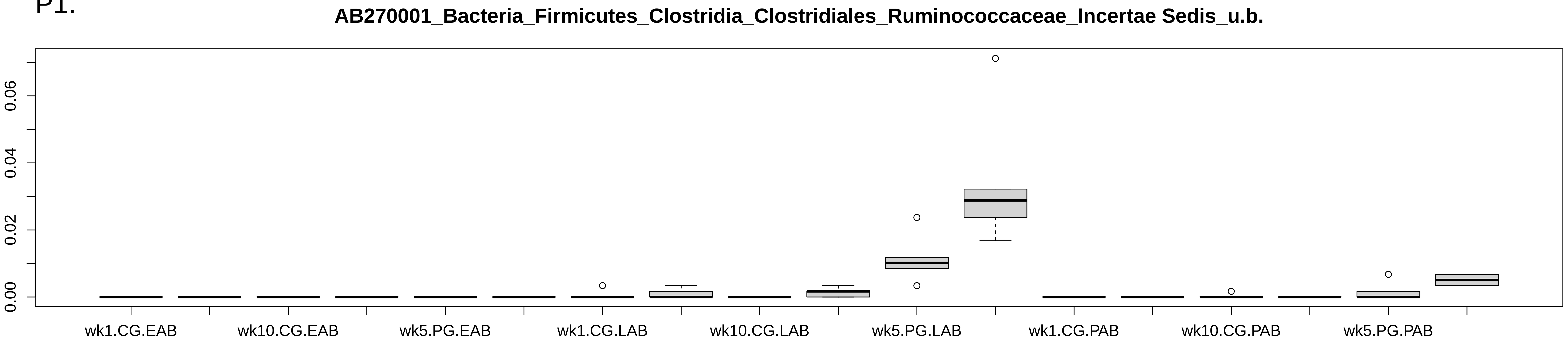

Statistics (p−values): Location: < 0.001; Group: < 0.001; L×G: < 0.001; Time: < 0.001; L×T:< 0.001; G×T: < 0.001; L×G×T: < 0.001; Cow: 0.980; T×C: 0.009.

P2.

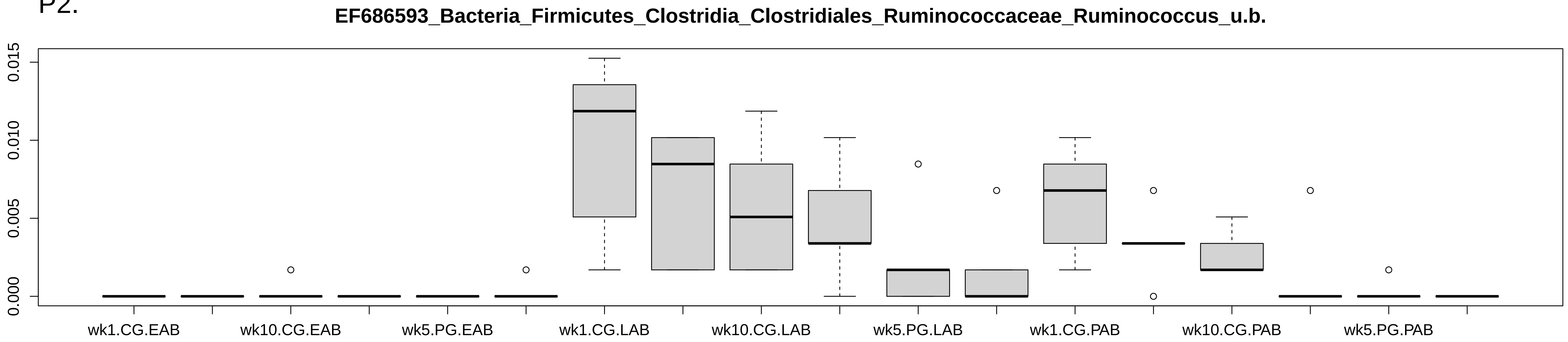

Statistics (p−values): Location: < 0.001; Group: < 0.001; L×G: 0.022; Time: 0.061; L×T:0.387; G×T: 0.902; L×G×T: 1.000; Cow: 0.448; T×C: 0.829.

P3.

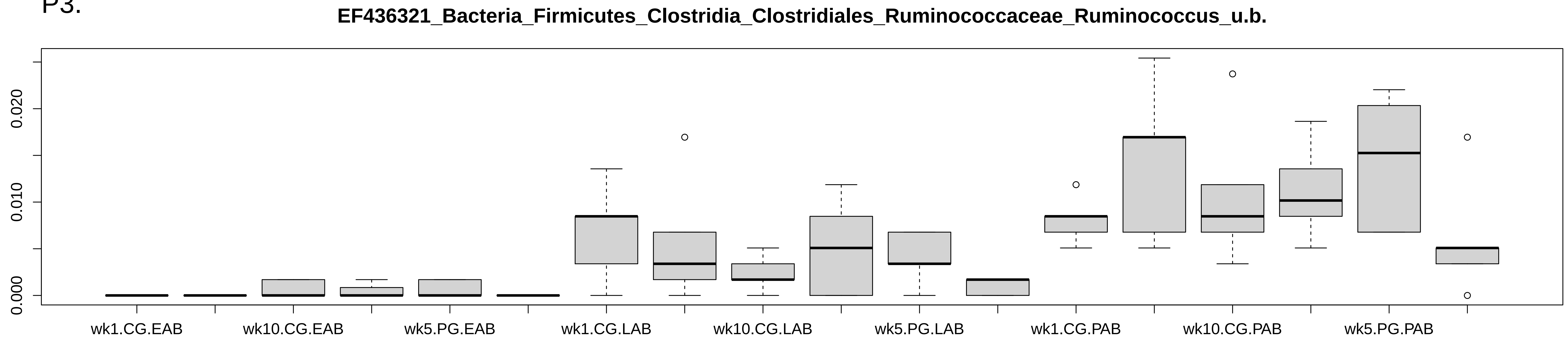

Statistics (p−values): Location: < 0.001; Group: 0.295; L×G: 0.843; Time: 0.055; L×T:0.124; G×T: 0.586; L×G×T: 0.818; Cow: 0.380; T×C: 0.853.

P4.

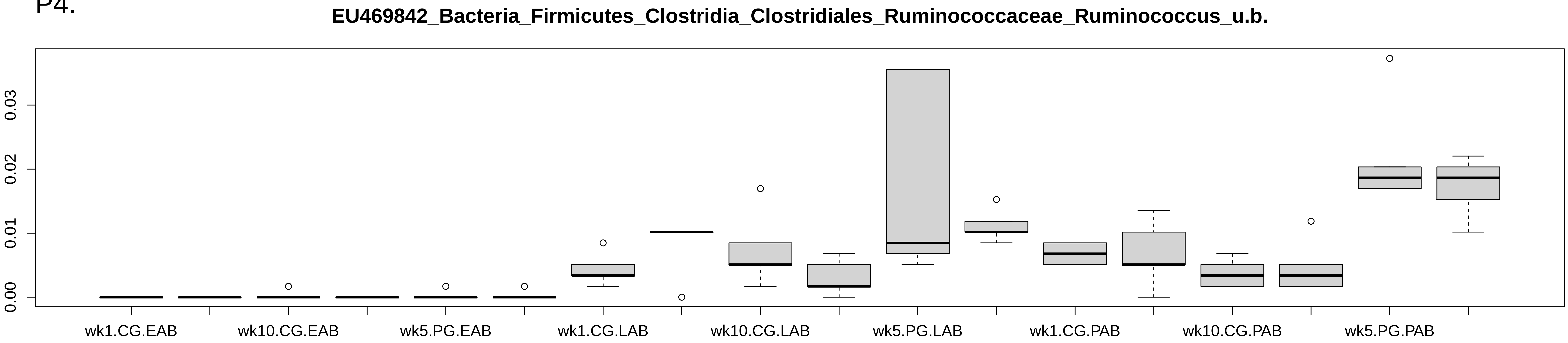

Statistics (p−values): Location: < 0.001; Group: < 0.001; L×G: 0.017; Time: < 0.001; L×T:0.080; G×T: 0.011; L×G×T: 0.098; Cow: 0.882; T×C: 0.141.

P5.

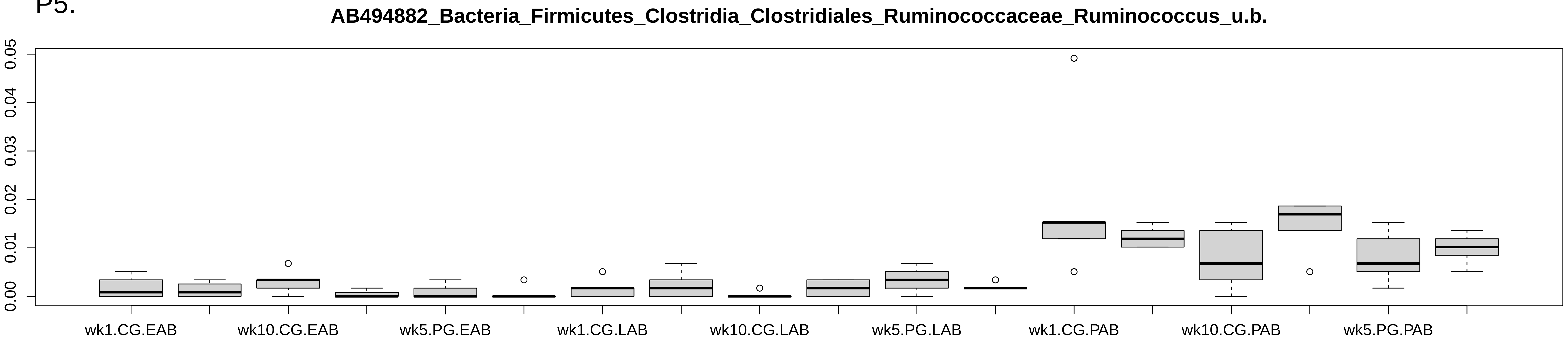

Statistics (p−values): Location: < 0.001; Group: 0.415; L×G: 0.600; Time: 0.169; L×T:0.061; G×T: 0.557; L×G×T: 0.628; Cow: 0.660; T×C: 0.833.

P6.

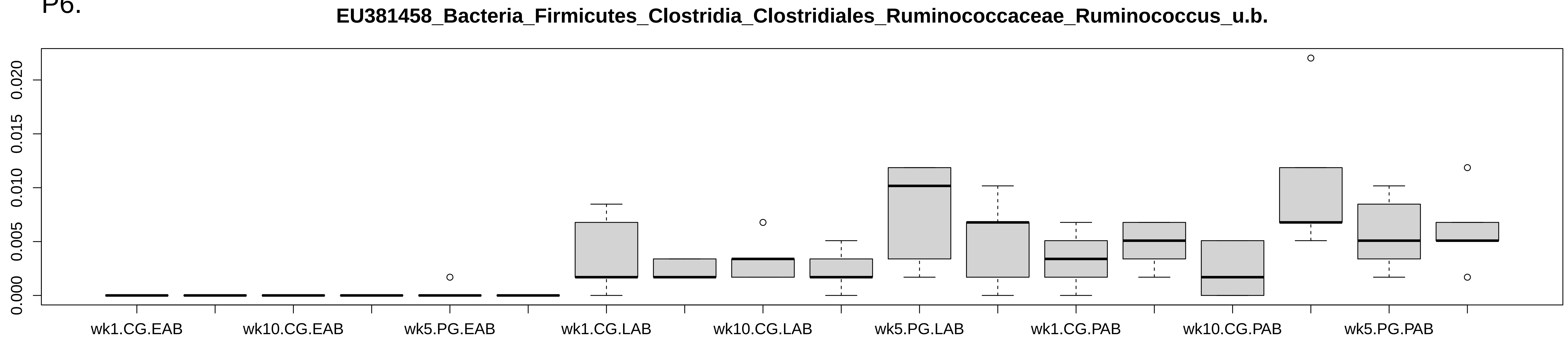

Statistics (p−values): Location: < 0.001; Group: 0.003; L×G: 0.060; Time: 0.436; L×T:0.231; G×T: 1.000; L×G×T: 0.012; Cow: 0.784; T×C: 0.635.

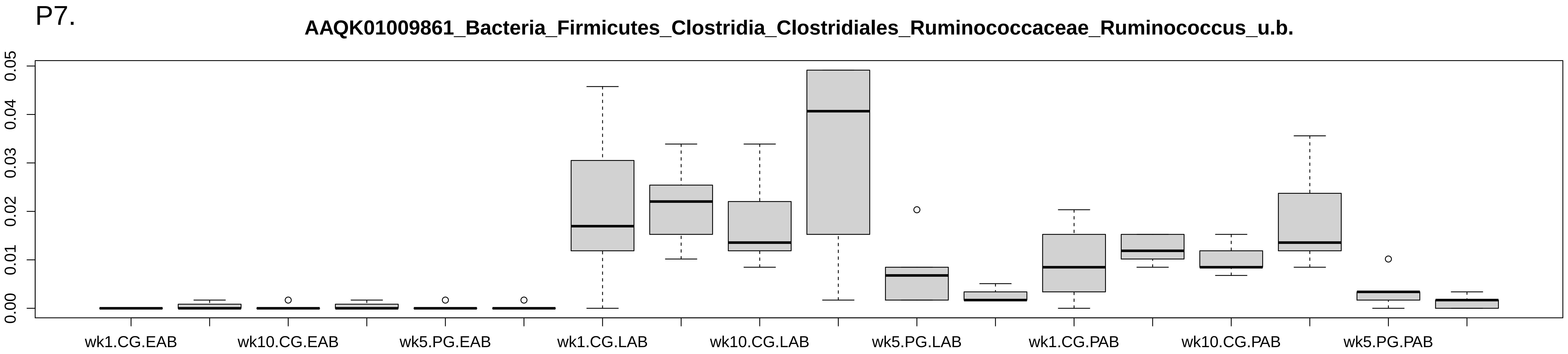

Statistics (p−values): Location: < 0.001; Group: 0.686; L×G: 0.534; Time: 0.001; L×T:0.091; G×T: < 0.001; L×G×T: 0.219; Cow: 0.706; T×C: 0.179.

P8.

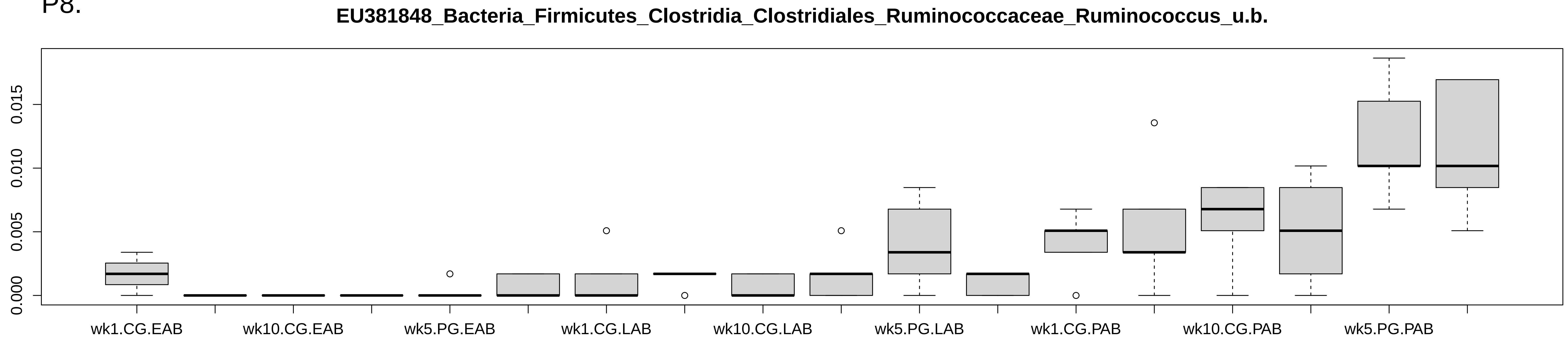

Statistics (p−values): Location: < 0.001; Group: 0.002; L×G: 0.009; Time: 0.199; L×T:0.016; G×T: 0.222; L×G×T: 0.419; Cow: 0.296; T×C: 0.093.

P9.

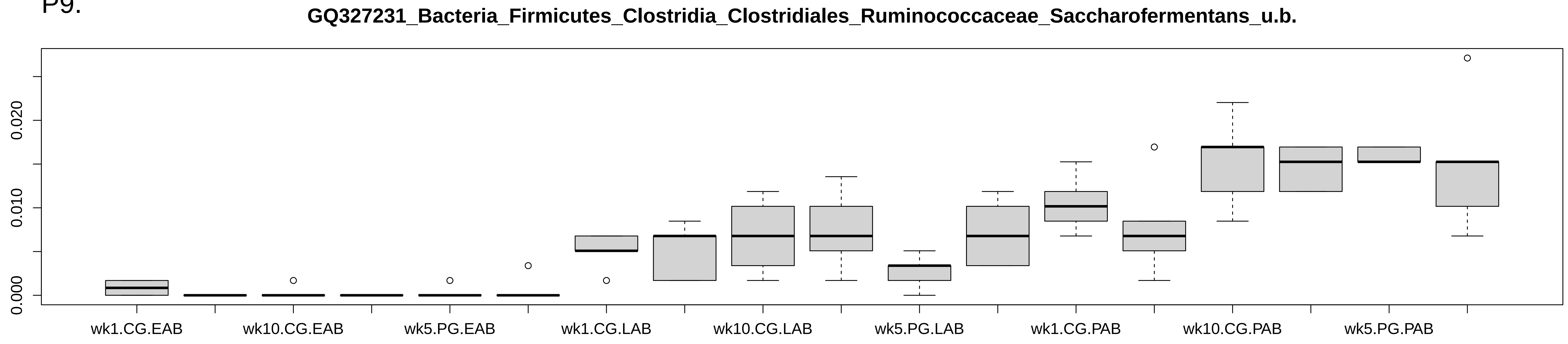

Statistics (p−values): Location: < 0.001; Group: 0.271; L×G: 0.261; Time: 0.035; L×T:0.610; G×T: 0.656; L×G×T: 0.097; Cow: 0.941; T×C: 1.000.

P10.

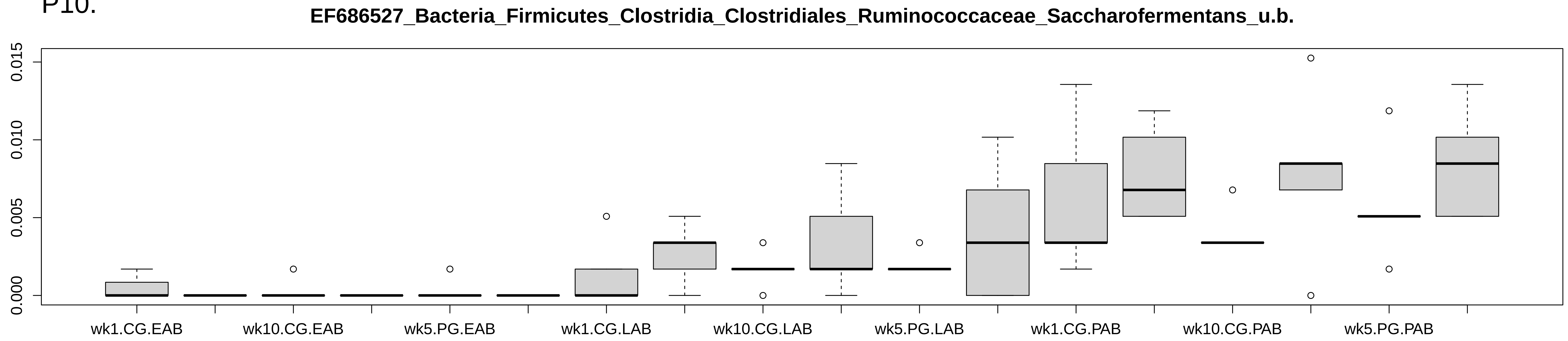

Statistics (p−values): Location: < 0.001; Group: 0.205; L×G: 0.510; Time: 0.980; L×T:0.977; G×T: 0.140; L×G×T: 0.398; Cow: 0.190; T×C: 0.478.

P11.

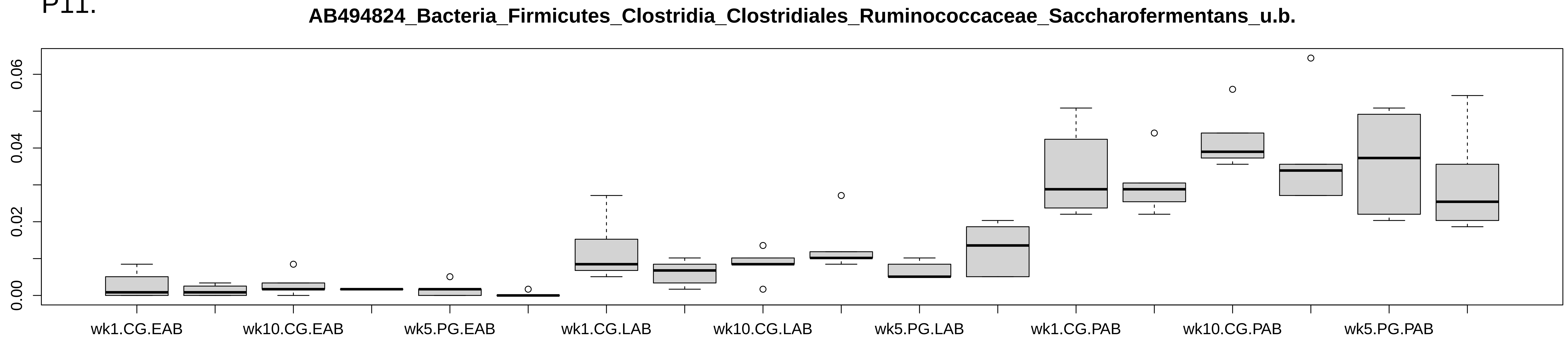

Statistics (p−values): Location: < 0.001; Group: 0.686; L×G: 0.598; Time: 0.247; L×T:0.707; G×T: 0.357; L×G×T: 0.297; Cow: 0.281; T×C: 0.229.

P12.

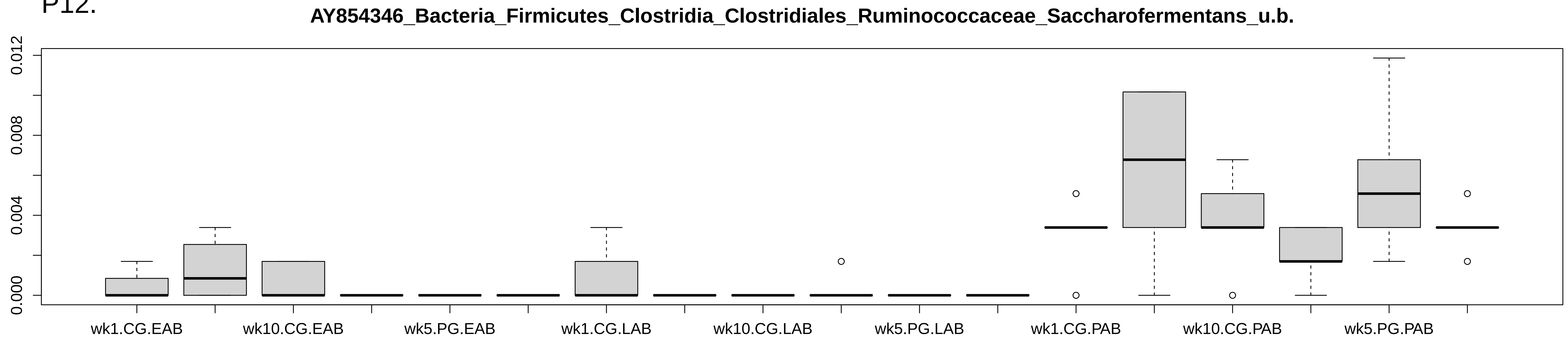

Statistics (p−values): Location: < 0.001; Group: 0.063; L×G: 0.869; Time: 0.042; L×T:0.009; G×T: 0.980; L×G×T: 1.000; Cow: 0.655; T×C: 0.410.

P13.

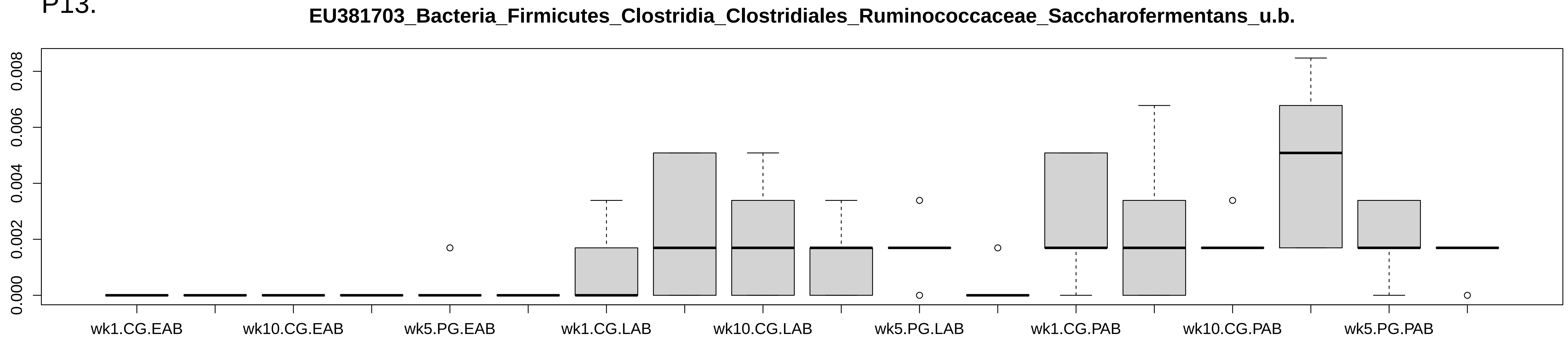

Statistics (p−values): Location: < 0.001; Group: 0.784; L×G: 0.396; Time: 0.199; L×T:0.097; G×T: 0.385; L×G×T: 0.689; Cow: 0.031; T×C: 0.289.

P14.

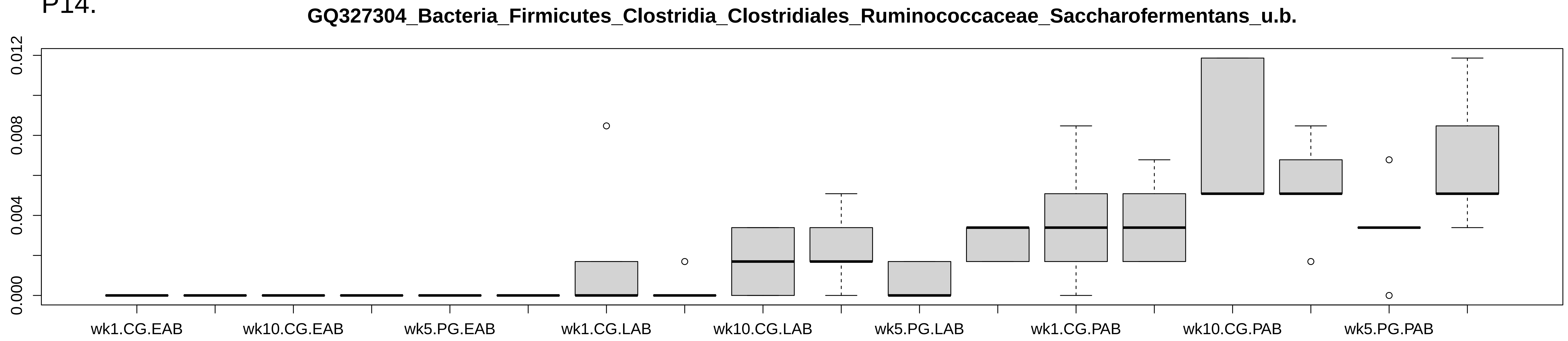

Statistics (p−values): Location: < 0.001; Group: 0.638; L×G: 0.804; Time: 0.017; L×T:0.061; G×T: 0.824; L×G×T: 0.841; Cow: 1.000; T×C: 0.589.

P15.

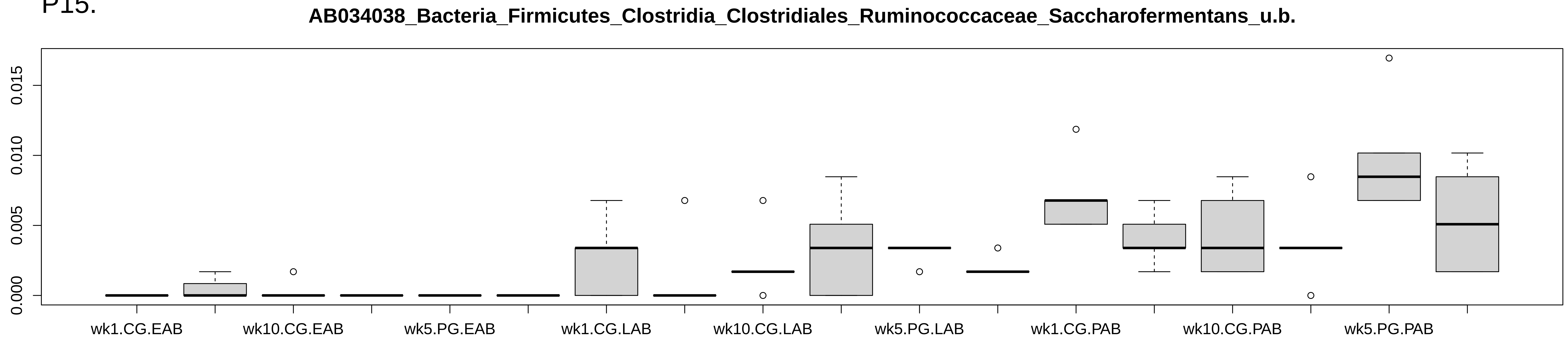

Statistics (p−values): Location: < 0.001; Group: 0.240; L×G: 0.690; Time: 0.594; L×T:0.537; G×T: 0.049; L×G×T: 0.049; Cow: 0.370; T×C: 0.725.

P16.

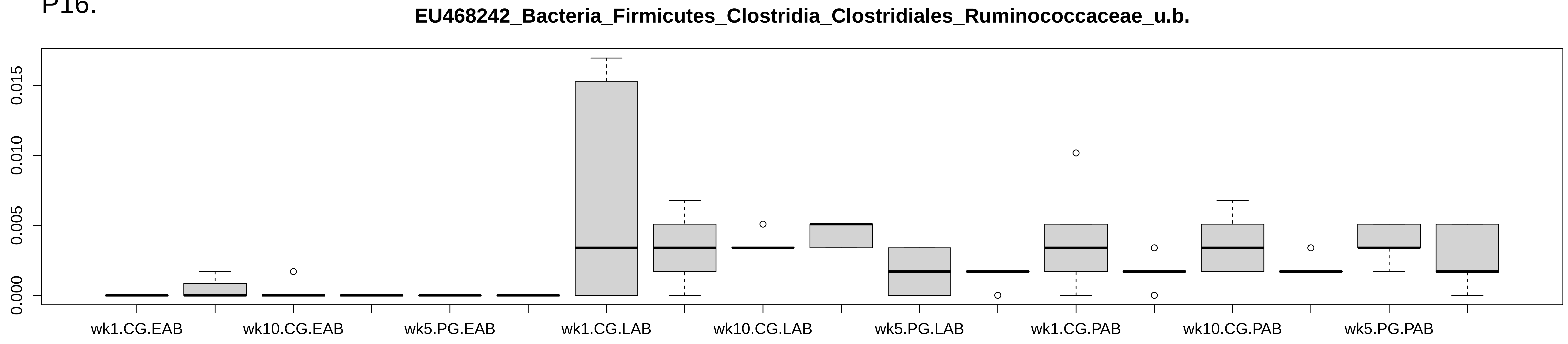

Statistics (p−values): Location: < 0.001; Group: 0.382; L×G: 0.370; Time: 0.422; L×T:0.160; G×T: 0.405; L×G×T: 0.900; Cow: 0.143; T×C: 0.306.

P17.

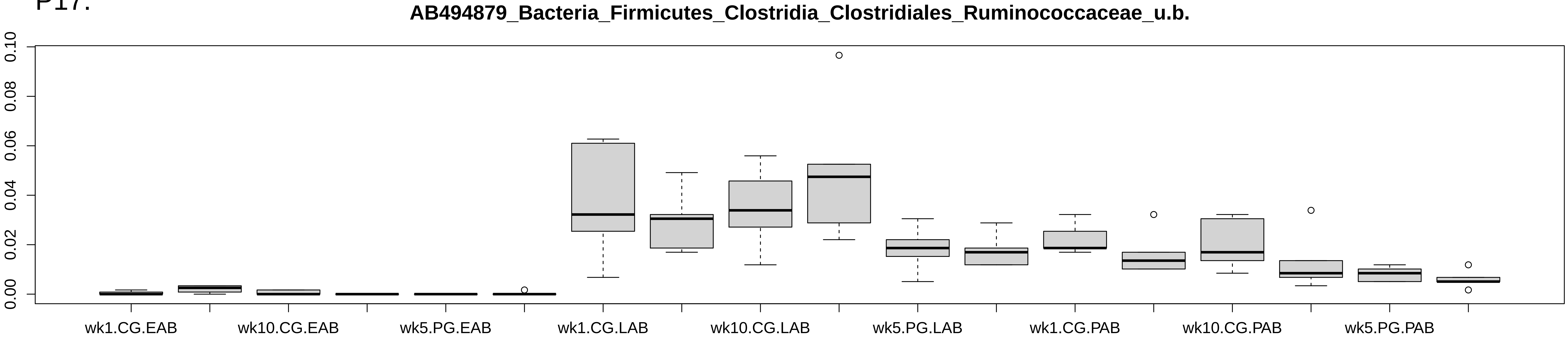

Statistics (p−values): Location: < 0.001; Group: 0.051; L×G: 0.273; Time: 0.024; L×T:0.077; G×T: 0.234; L×G×T: 0.456; Cow: 0.843; T×C: 0.701.

P18.

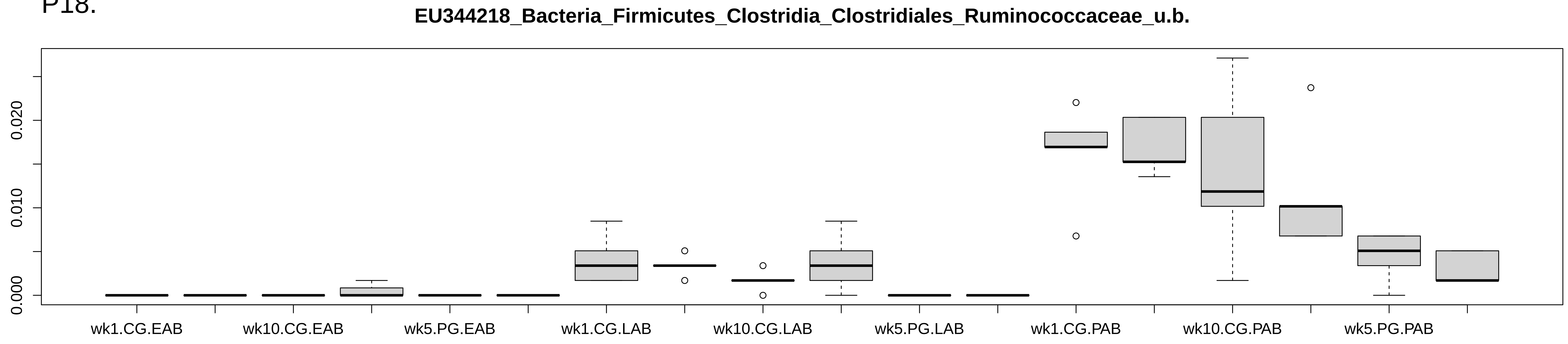

Statistics (p−values): Location: < 0.001; Group: < 0.001; L×G: < 0.001; Time: 0.022; L×T:0.297; G×T: 0.141; L×G×T: 0.536; Cow: 0.765; T×C: 1.000.

P19.

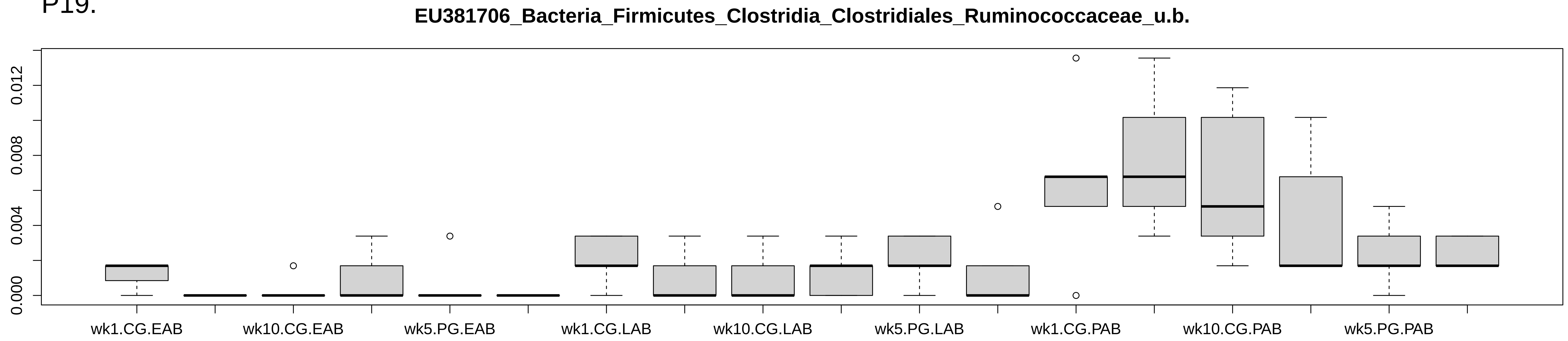

Statistics (p−values): Location: < 0.001; Group: 0.144; L×G: 0.001; Time: 0.420; L×T:1.000; G×T: 1.000; L×G×T: 0.536; Cow: 0.458; T×C: 0.774.

P20.

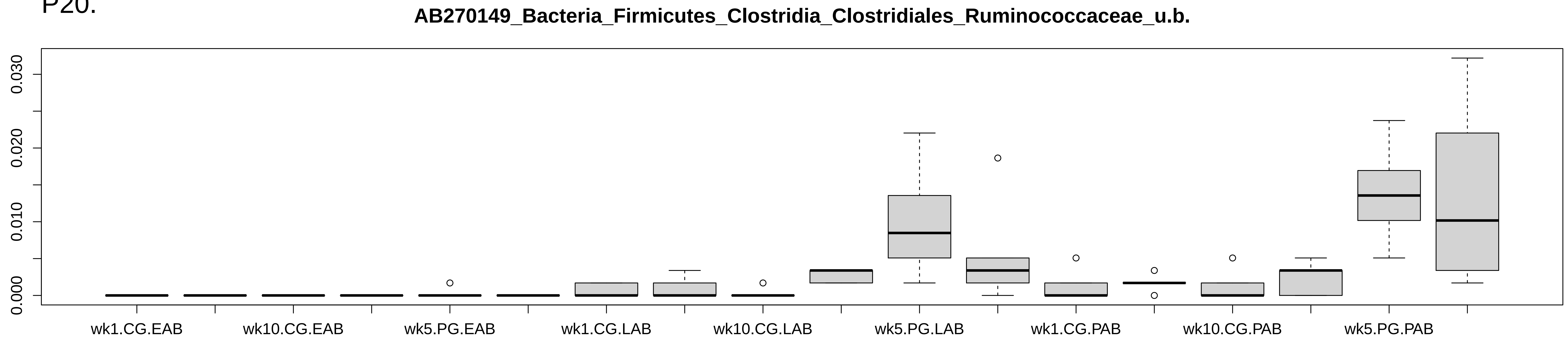

Statistics (p−values): Location: < 0.001; Group: < 0.001; L×G: < 0.001; Time: 0.022; L×T:0.186; G×T: 0.034; L×G×T: 0.317; Cow: 1.000; T×C: 0.346.

P21.

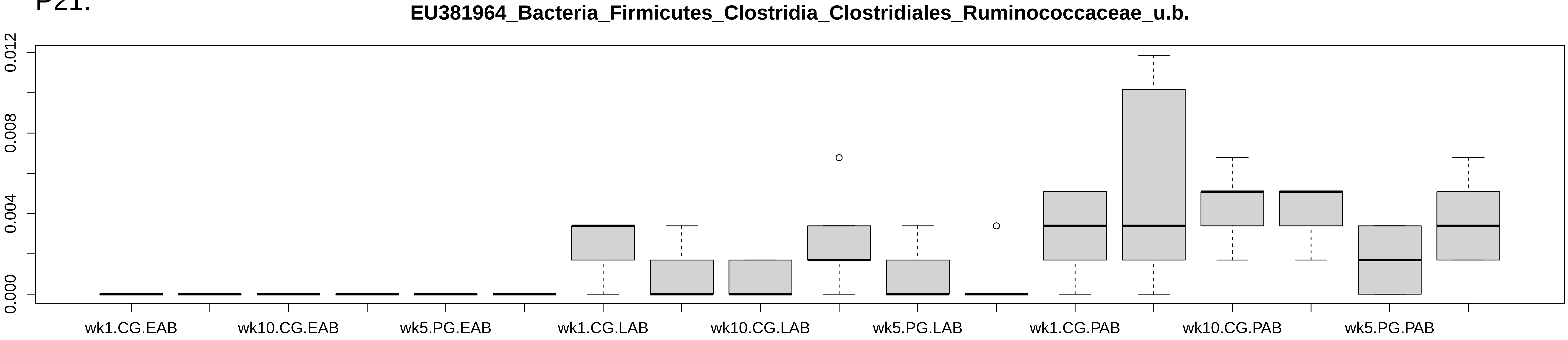

Statistics (p−values): Location: < 0.001; Group: 0.344; L×G: 0.134; Time: 0.464; L×T:0.276; G×T: 0.143; L×G×T: 0.266; Cow: 0.540; T×C: 0.045.

P22.

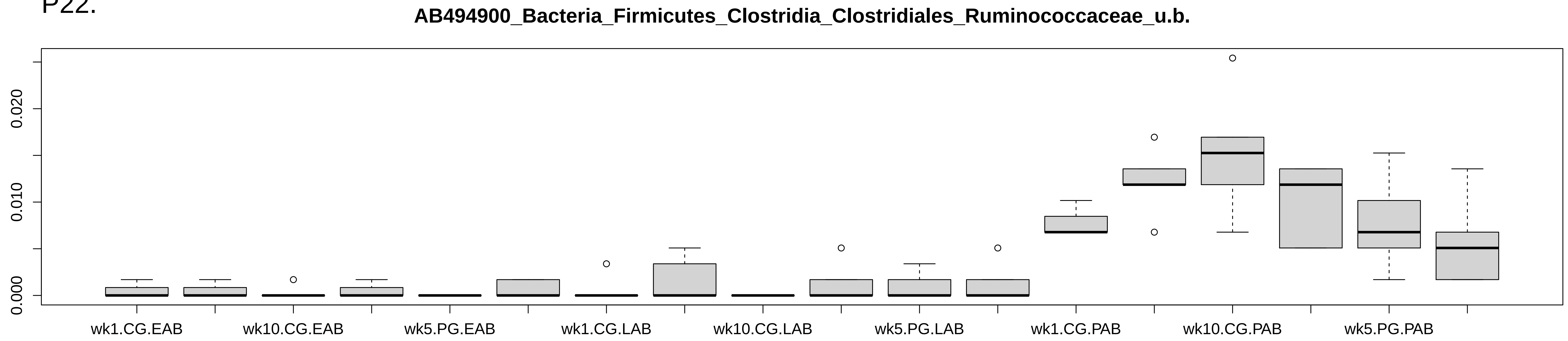

Statistics (p−values): Location: < 0.001; Group: 0.143; L×G: 0.011; Time: 1.000; L×T:1.000; G×T: 0.025; L×G×T: 0.020; Cow: 0.183; T×C: 0.083.

P23.

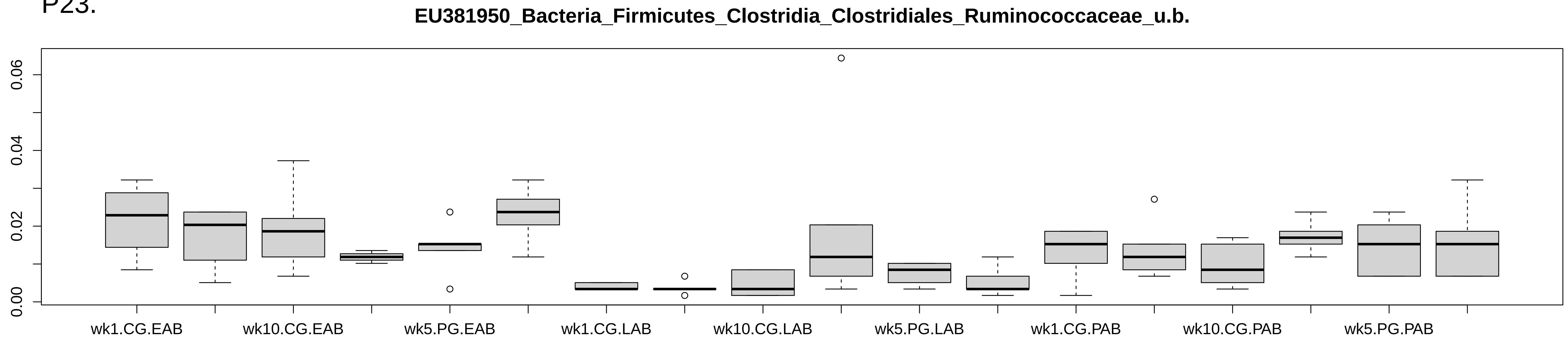

Statistics (p−values): Location: < 0.001; Group: 0.171; L×G: 0.090; Time: 0.568; L×T:0.362; G×T: 0.962; L×G×T: 0.264; Cow: 0.418; T×C: 0.447.

P23..1

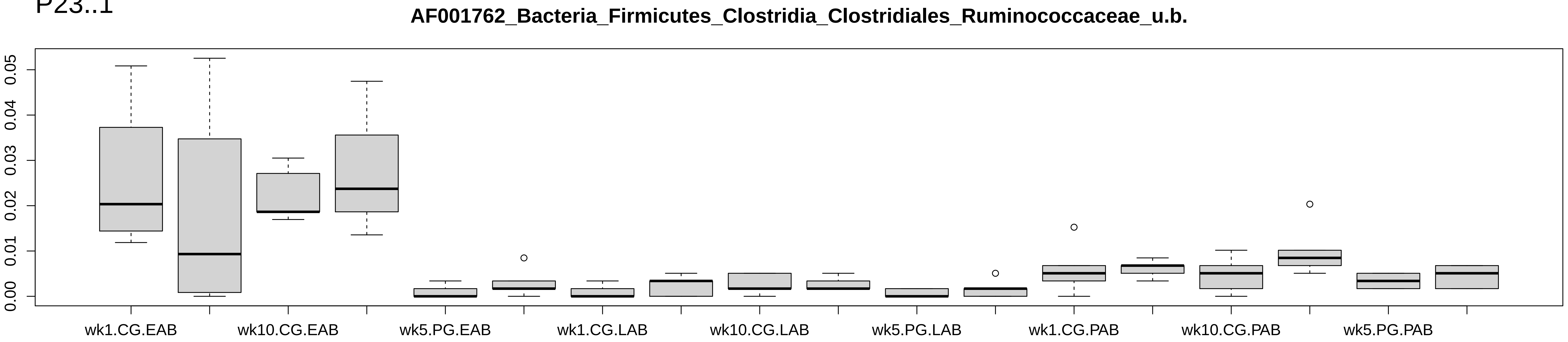

Statistics (p−values): Location: < 0.001; Group: 0.016; L×G: 0.010; Time: 0.002; L×T:0.010; G×T: 0.041; L×G×T: 0.315; Cow: 0.032; T×C: 0.980.

P24.

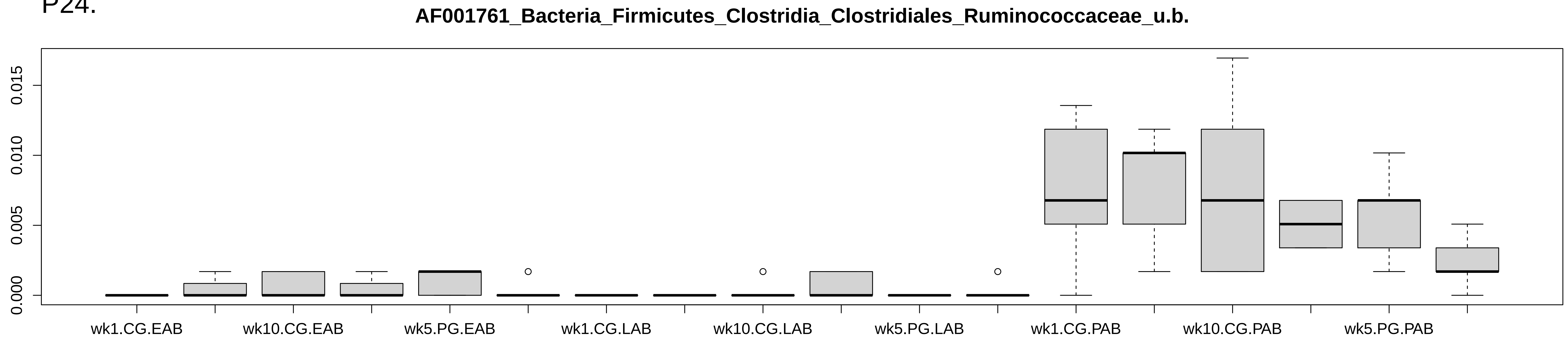

Statistics (p−values): Location: < 0.001; Group: 0.088; L×G: 0.013; Time: 0.750; L×T:0.764; G×T: 0.392; L×G×T: 1.000; Cow: 0.686; T×C: 0.904.

P25.

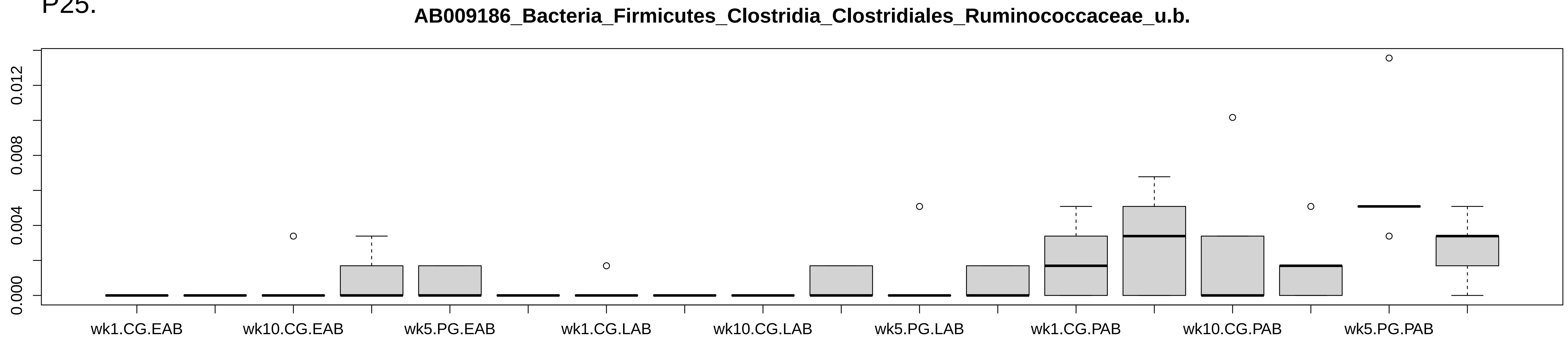

Statistics (p−values): Location: < 0.001; Group: 0.380; L×G: 0.861; Time: 0.197; L×T:0.093; G×T: 0.406; L×G×T: 0.400; Cow: 0.521; T×C: 0.902.

P26.

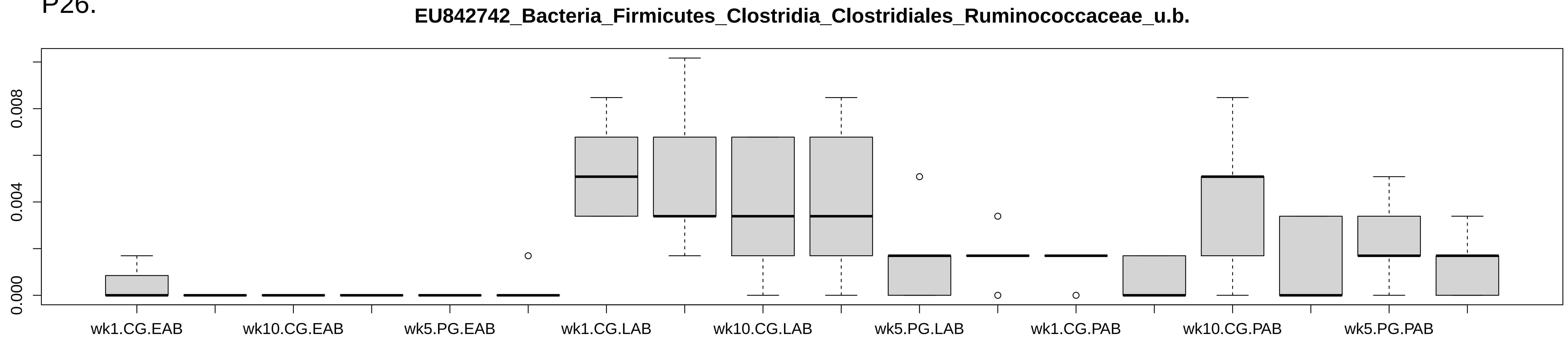

Statistics (p−values): Location: < 0.001; Group: 0.094; L×G: 0.071; Time: 1.000; L×T:0.090; G×T: 0.251; L×G×T: 0.094; Cow: 0.050; T×C: 0.650.

P27.

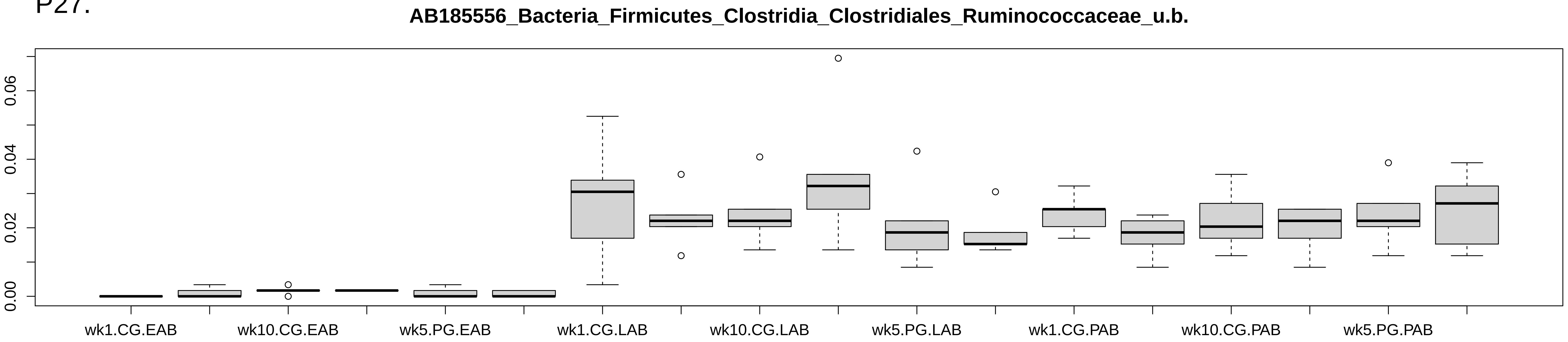

Statistics (p−values): Location: < 0.001; Group: 0.784; L×G: 1.000; Time: 0.407; L×T:0.795; G×T: 0.784; L×G×T: 0.448; Cow: 0.034; T×C: 0.275.

P28.

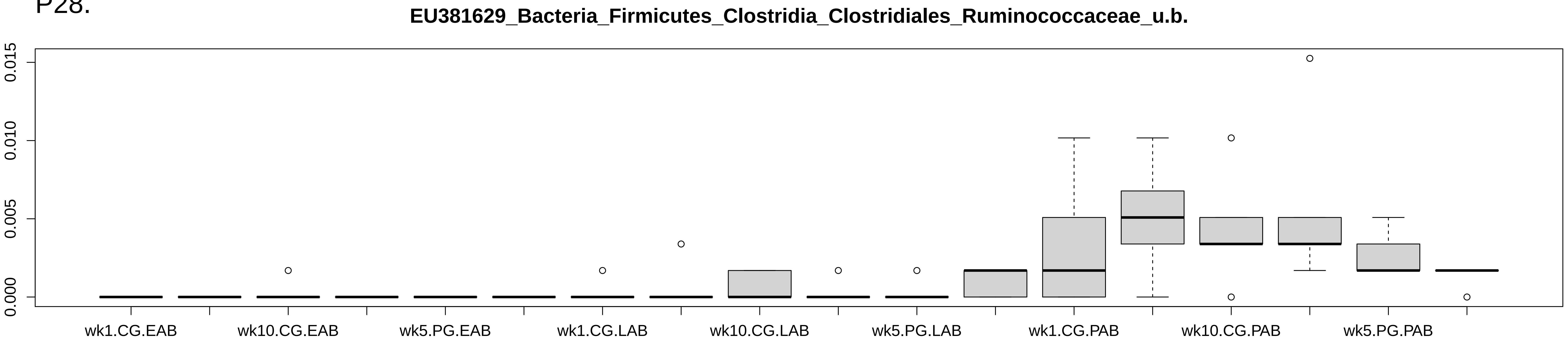

Statistics (p−values): Location: < 0.001; Group: 0.804; L×G: 0.574; Time: 0.786; L×T:0.349; G×T: 0.296; L×G×T: 0.371; Cow: 1.000; T×C: 0.582.

P29.

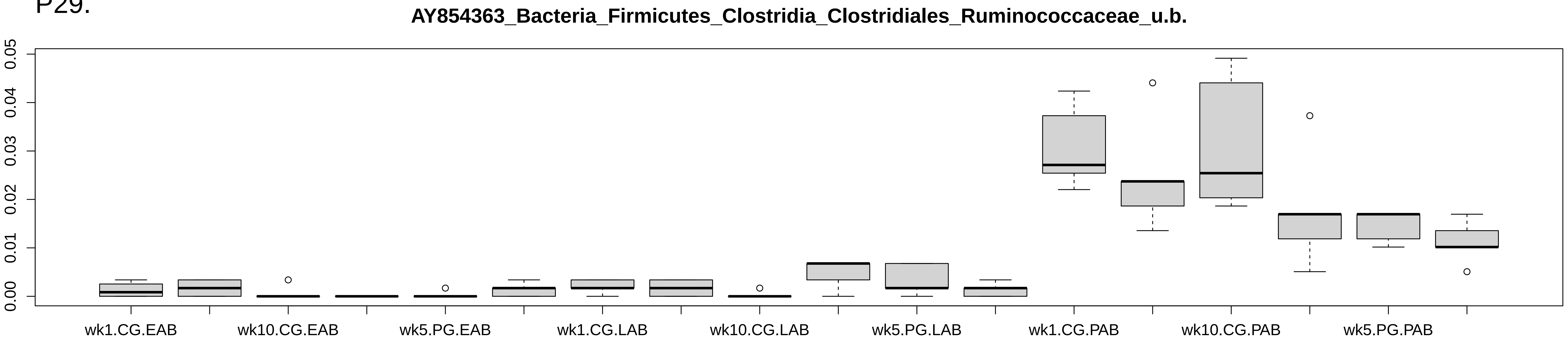

Statistics (p−values): Location: < 0.001; Group: 0.006; L×G: < 0.001; Time: 0.732; L×T:0.831; G×T: 0.601; L×G×T: 0.438; Cow: 0.784; T×C: 0.240.

P30.

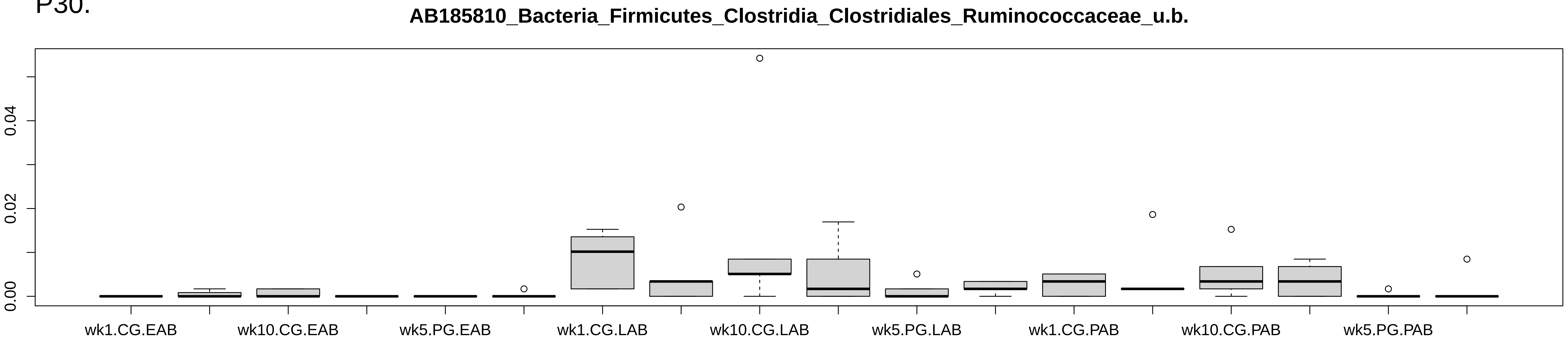

Statistics (p−values): Location: 0.018; Group: 0.011; L×G: 0.198; Time: 0.802; L×T:0.910; G×T: 1.000; L×G×T: 0.578; Cow: 0.060; T×C: 0.715.

P31.

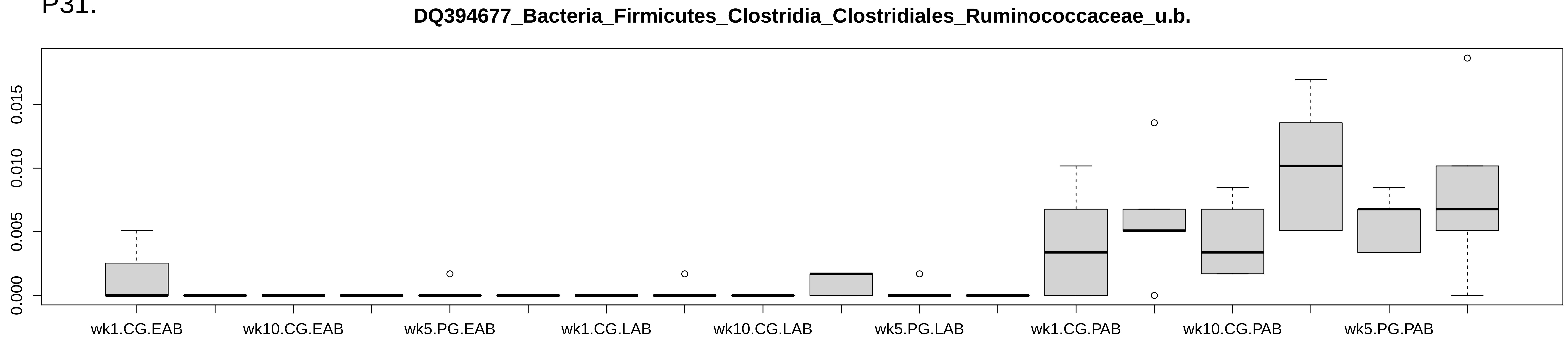

Statistics (p−values): Location: < 0.001; Group: 0.065; L×G: 0.088; Time: 0.801; L×T:0.987; G×T: 0.551; L×G×T: 0.394; Cow: 1.000; T×C: 0.773.

P32.

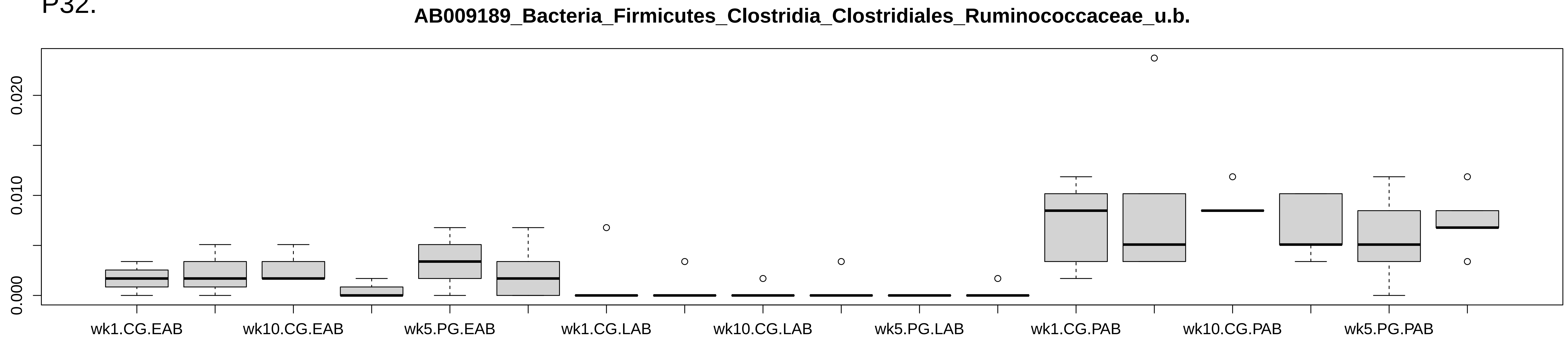

Statistics (p−values): Location: < 0.001; Group: 0.282; L×G: 0.867; Time: 0.641; L×T:0.740; G×T: 1.000; L×G×T: 0.774; Cow: 0.564; T×C: 0.355.

Q1.

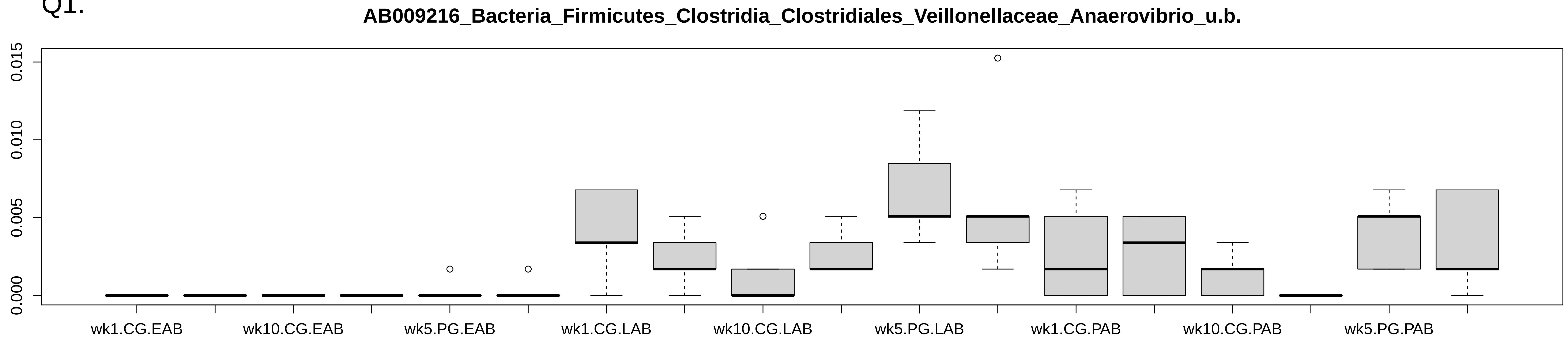

Statistics (p−values): Location: < 0.001; Group: 0.062; L×G: 0.140; Time: 0.223; L×T:0.806; G×T: 0.002; L×G×T: 0.384; Cow: 0.922; T×C: 0.643.

Q2.

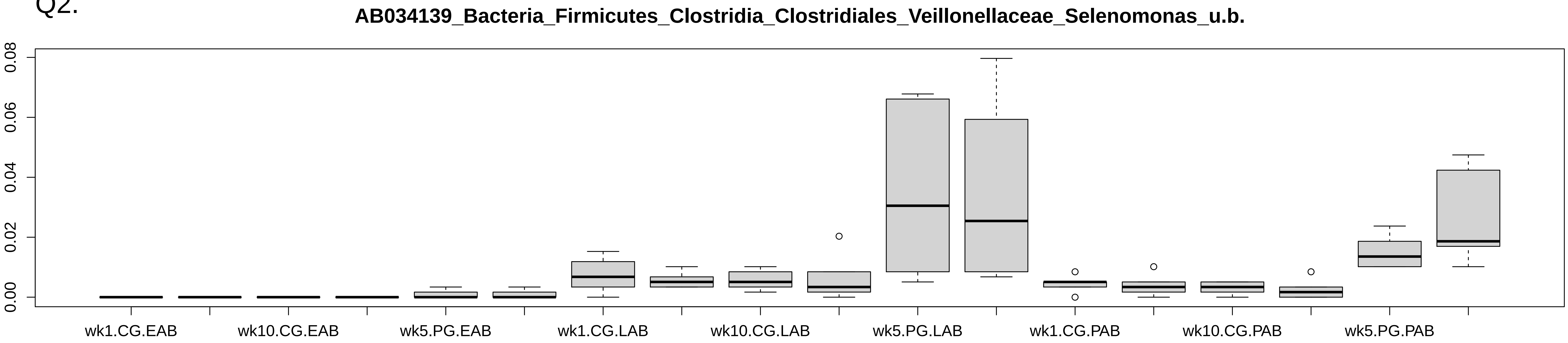

Statistics (p−values): Location: < 0.001; Group: < 0.001; L×G: 0.003; Time: 0.019; L×T:0.234; G×T: 0.001; L×G×T: 0.273; Cow: 0.500; T×C: 0.250.

Q3.

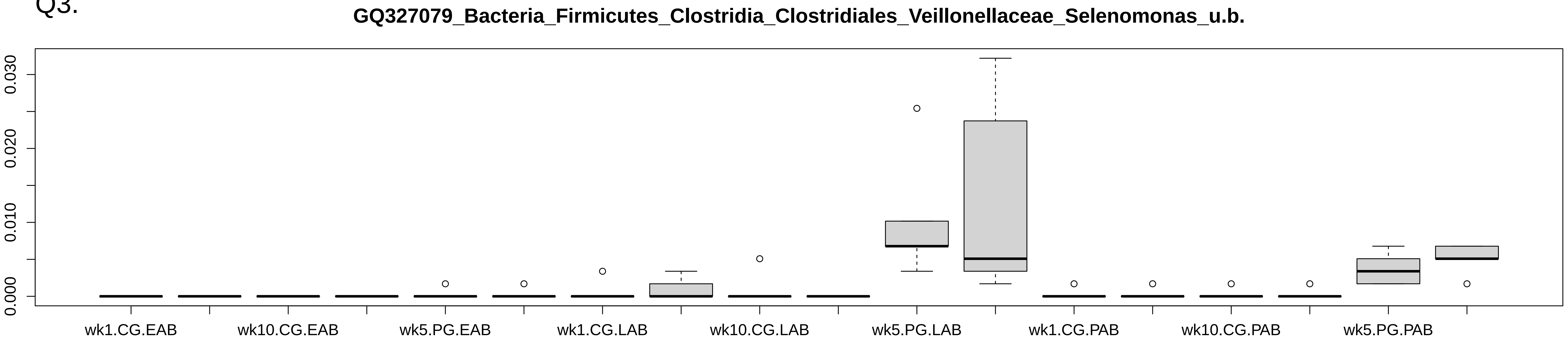

Statistics (p−values): Location: 0.008; Group: < 0.001; L×G: 0.016; Time: 0.029; L×T:0.178; G×T: 0.033; L×G×T: 0.321; Cow: 0.843; T×C: 0.679.

Q4.

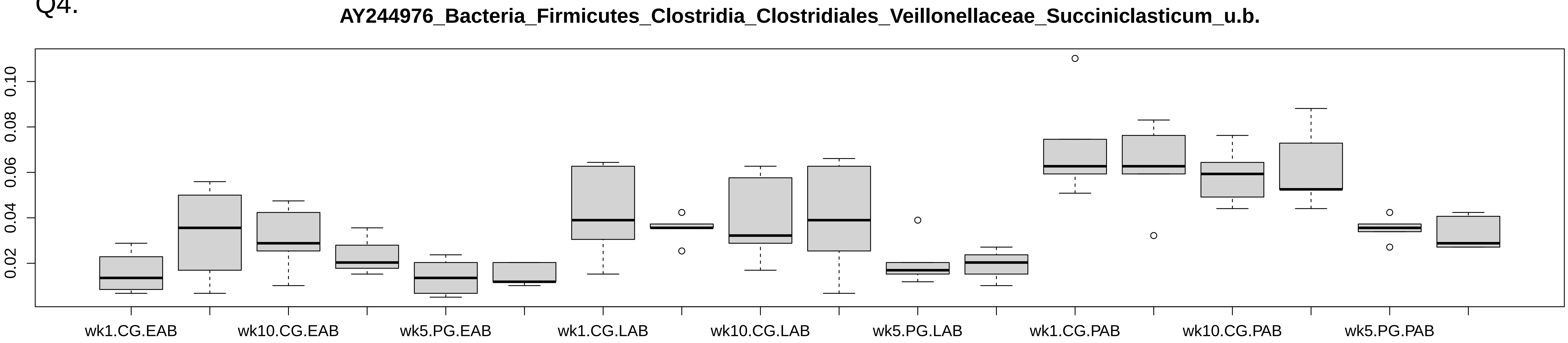

Statistics (p−values): Location: < 0.001; Group: < 0.001; L×G: 0.476; Time: 0.036; L×T:0.163; G×T: 0.042; L×G×T: 1.000; Cow: 0.373; T×C: 0.765.

Q5.

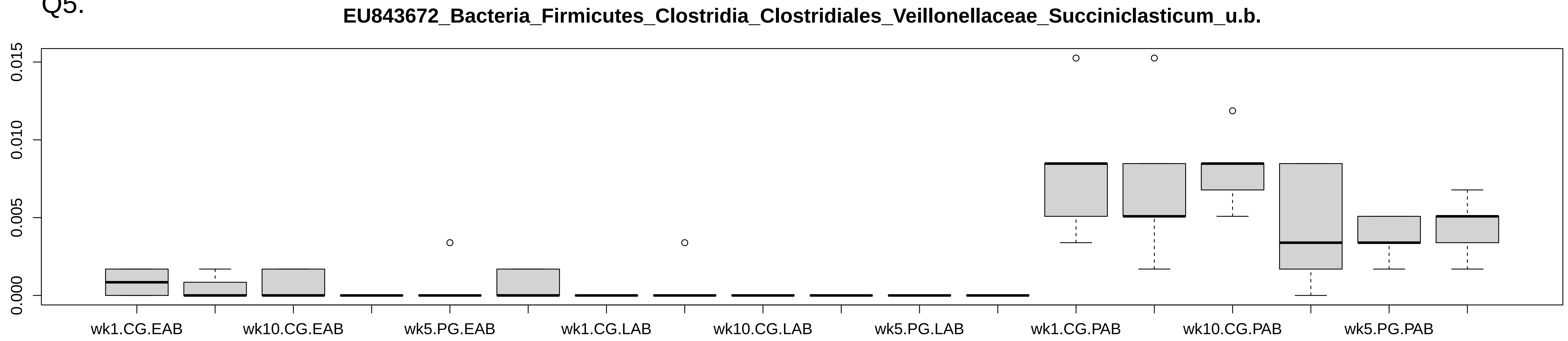

Statistics (p−values): Location: < 0.001; Group: 0.014; L×G: 0.001; Time: 1.000; L×T:0.752; G×T: 0.922; L×G×T: 0.988; Cow: 0.327; T×C: 0.815.

R1.

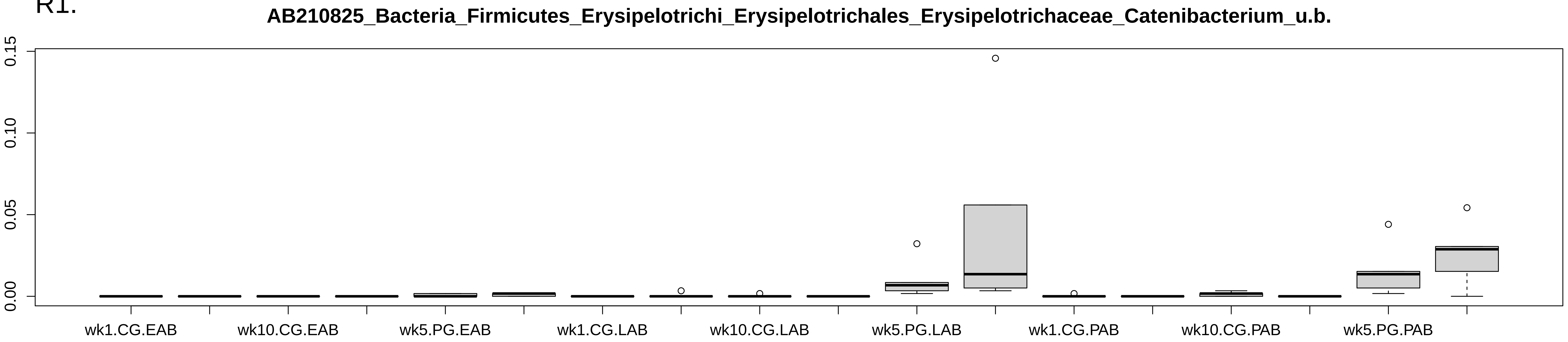

Statistics (p−values): Location: 0.039; Group: 0.001; L×G: 0.104; Time: 0.013; L×T:0.342; G×T: 0.003; L×G×T: 0.432; Cow: 0.686; T×C: 0.220.

R2.

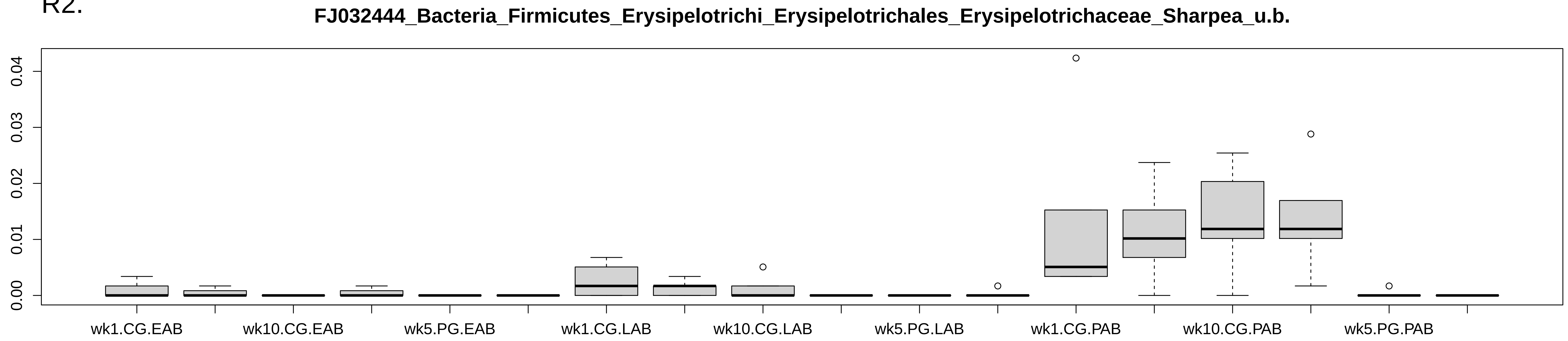

Statistics (p−values): Location: < 0.001; Group: 0.008; L×G: 0.106; Time: 0.208; L×T:0.274; G×T: 0.505; L×G×T: 0.305; Cow: 0.655; T×C: 0.961.

R3.

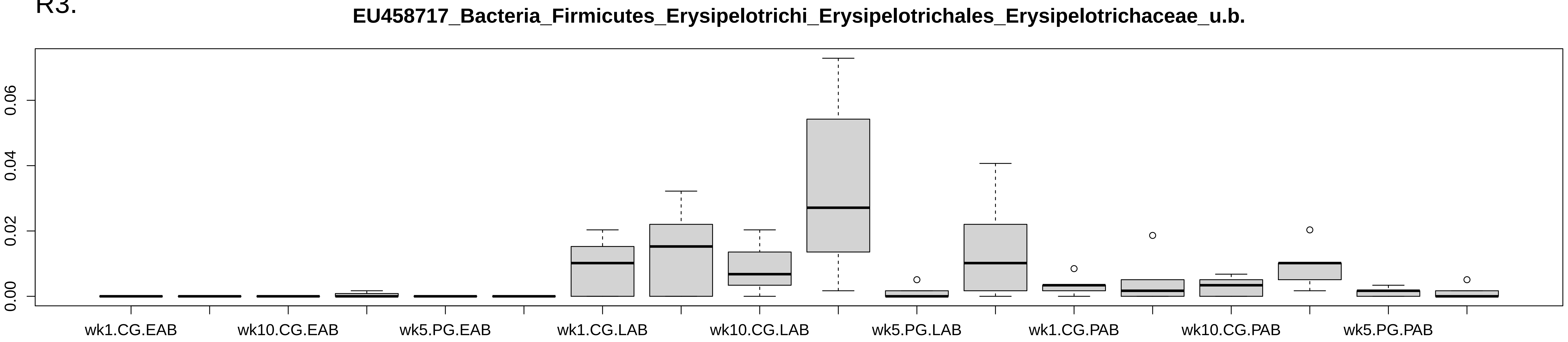

Statistics (p−values): Location: < 0.001; Group: 0.706; L×G: 0.322; Time: 0.058; L×T:0.304; G×T: 0.020; L×G×T: 0.065; Cow: 1.000; T×C: 0.788.

R4.

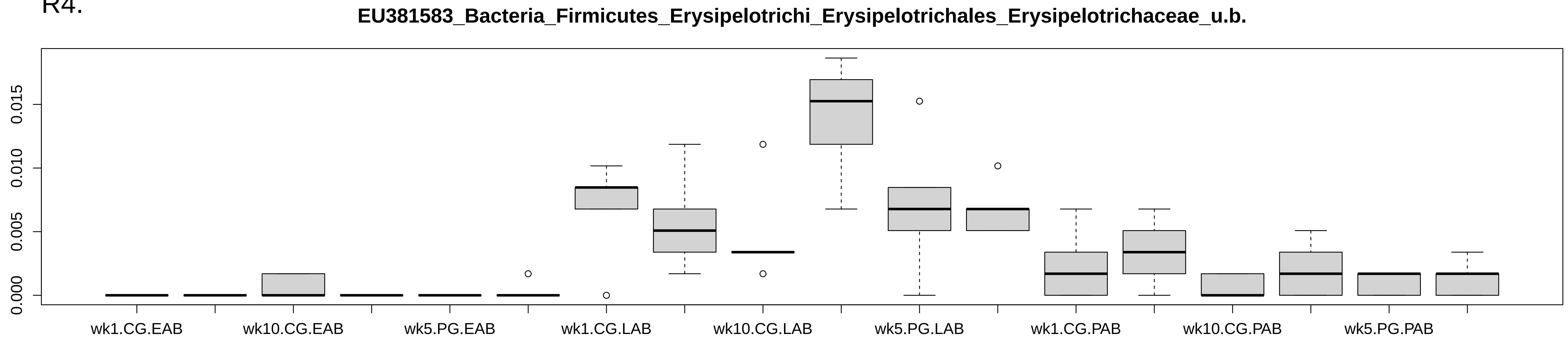

Statistics (p−values): Location: < 0.001; Group: 0.148; L×G: 0.027; Time: 0.045; L×T:0.052; G×T: 0.169; L×G×T: 0.184; Cow: 0.373; T×C: 0.686.

R5.

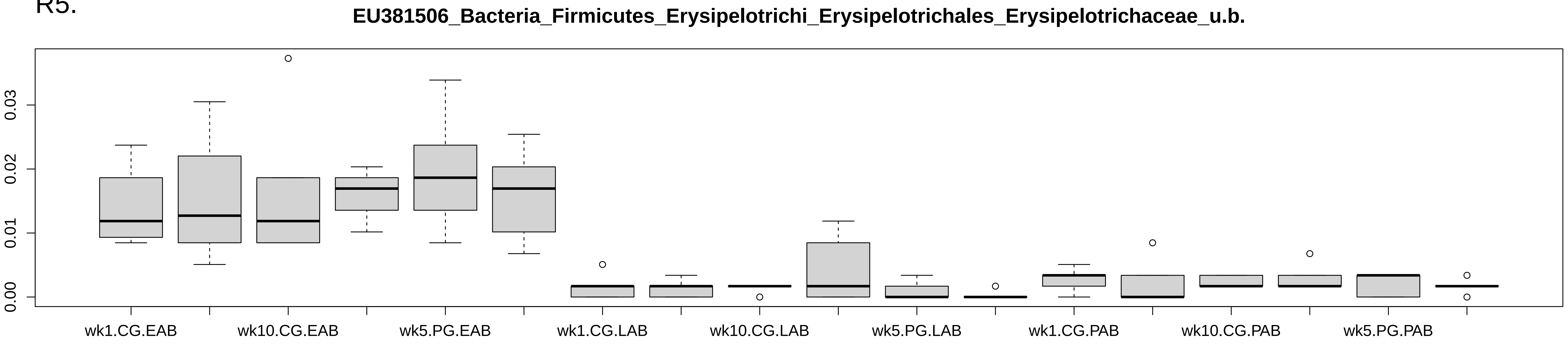

Statistics (p−values): Location: < 0.001; Group: 0.413; L×G: 0.975; Time: 1.000; L×T:0.940; G×T: 0.510; L×G×T: 0.720; Cow: 0.177; T×C: 0.662.

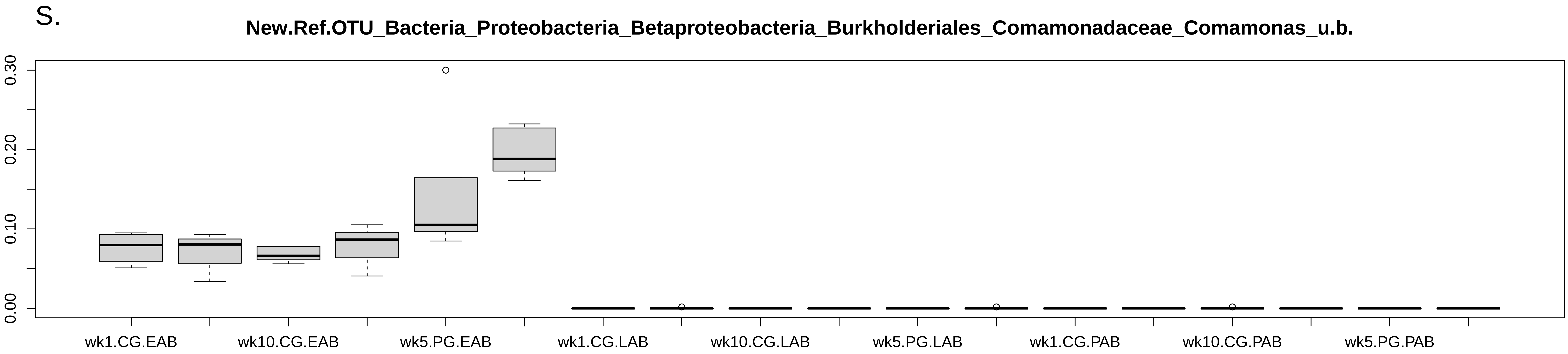

Statistics (p−values): Location: < 0.001; Group: < 0.001; L×G: < 0.001; Time: 0.010; L×T:0.006; G×T: 0.006; L×G×T: 0.001; Cow: 0.583; T×C: 0.209.

T1.

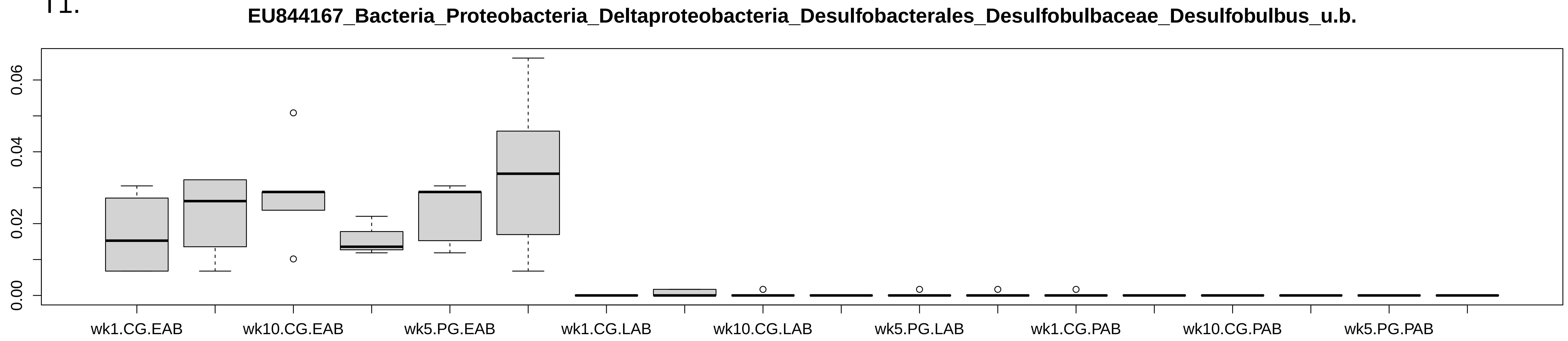

Statistics (p−values): Location: < 0.001; Group: 1.000; L×G: 1.000; Time: 0.068; L×T:0.032; G×T: 0.922; L×G×T: 1.000; Cow: 0.096; T×C: 0.630.

T2.

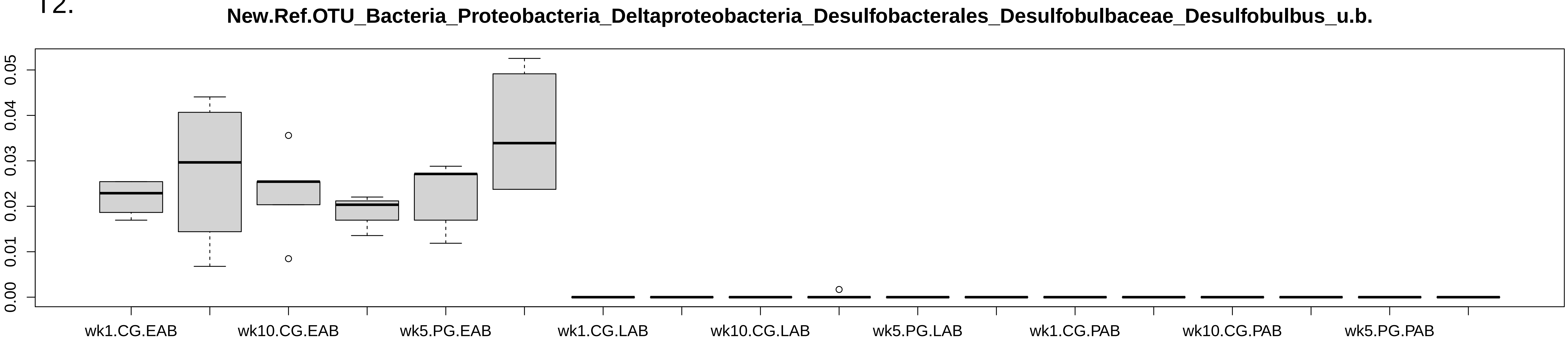

Statistics (p−values): Location: < 0.001; Group: 1.000; L×G: 1.000; Time: 0.103; L×T:0.193; G×T: 0.154; L×G×T: 0.077; Cow: 0.804; T×C: 0.421.

T3.

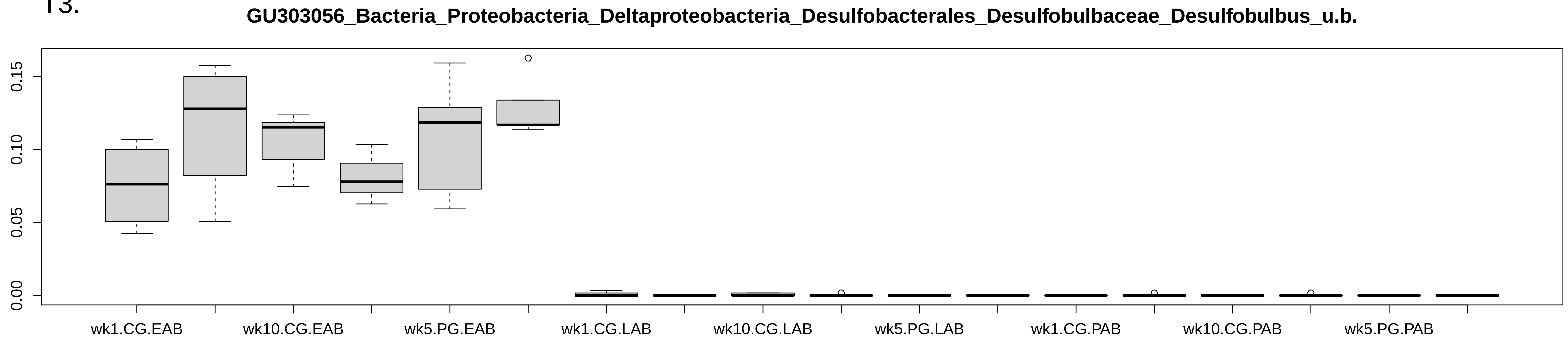

Statistics (p−values): Location: < 0.001; Group: 0.804; L×G: 0.472; Time: 0.015; L×T:0.001; G×T: 0.444; L×G×T: 0.614; Cow: 0.726; T×C: 0.461.

U.

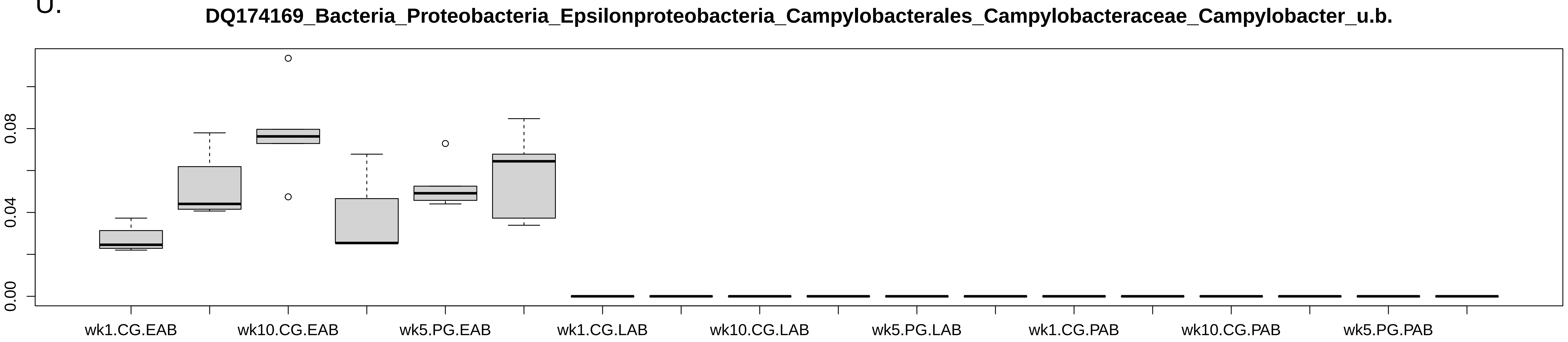

Statistics (p−values): Location: < 0.001; Group: 0.765; L×G: 0.853; Time: < 0.001; L×T:< 0.001; G×T: 0.182; L×G×T: 0.095; Cow: 0.902; T×C: 0.989.

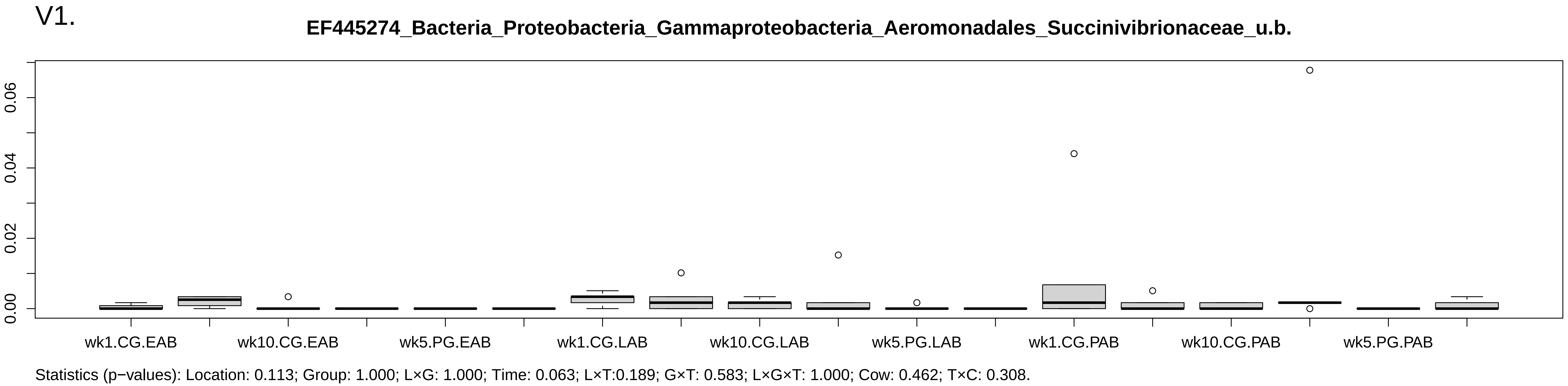
V1.
EF445274_Bacteria_Proteobacteria_Gammaproteobacteria_Aeromonadales_Succinivibrionaceae_u.b.
0.00
0.02
0.04
0.06
wk1.CG.EAB
wk10.CG.EAB
wk5.PG.EAB
wk1.CG.LAB
wk10.CG.LAB
wk5.PG.LAB
wk1.CG.PAB
wk10.CG.PAB
wk5.PG.PAB
Statistics (p−values): Location: 0.113; Group: 1.000; L×G: 1.000; Time: 0.063; L×T:0.189; G×T: 0.583; L×G×T: 1.000; Cow: 0.462; T×C: 0.308.

V2.

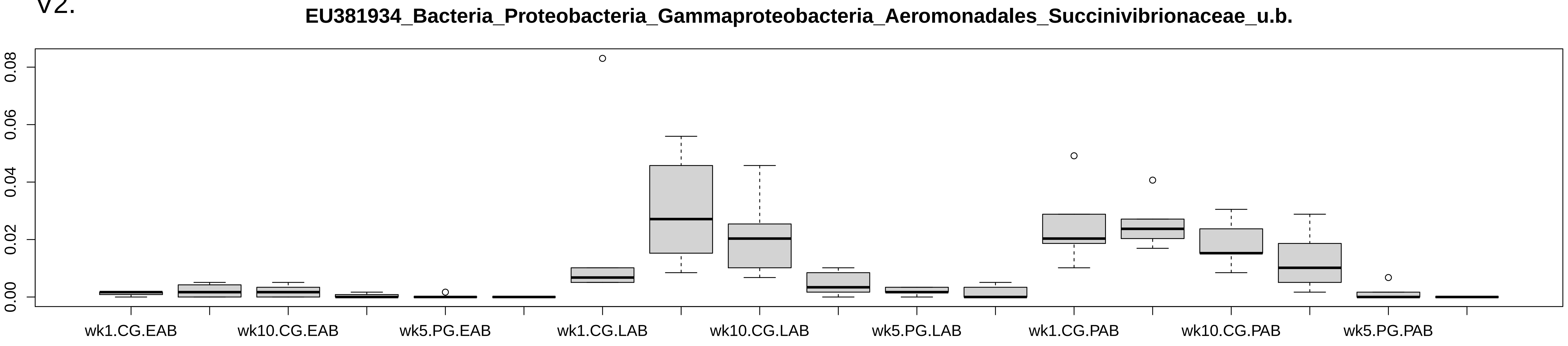

Statistics (p−values): Location: < 0.001; Group: < 0.001; L×G: 0.007; Time: 0.324; L×T:0.444; G×T: 0.543; L×G×T: 1.000; Cow: 0.980; T×C: 1.000.

W1.

New.Ref.OTU_Bacteria_Proteobacteria_Gammaproteobacteria_Cardiobacteriales_Cardiobacteriaceae_Suttonella_u.b.

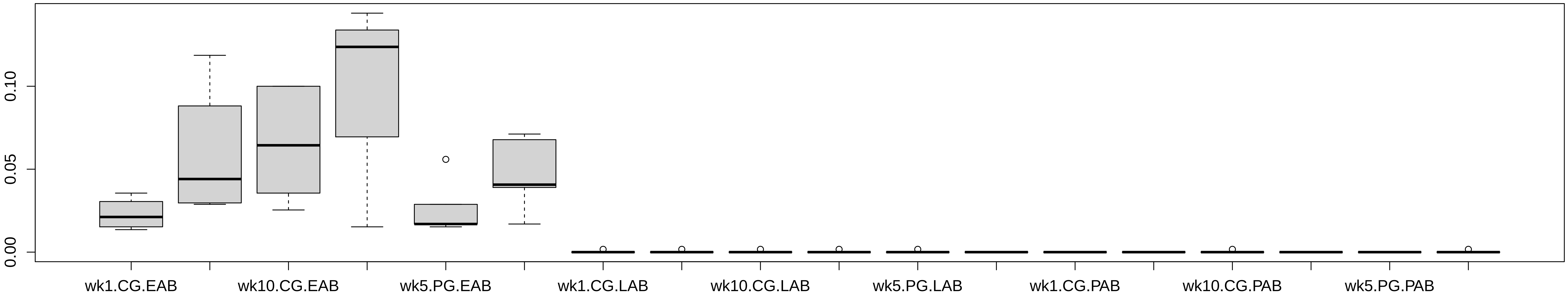

Statistics (p−values): Location: < 0.001; Group: 0.941; L×G: 0.647; Time: 0.987; L×T:0.443; G×T: < 0.001; L×G×T: < 0.001; Cow: 0.478; T×C: 0.253.

X1.

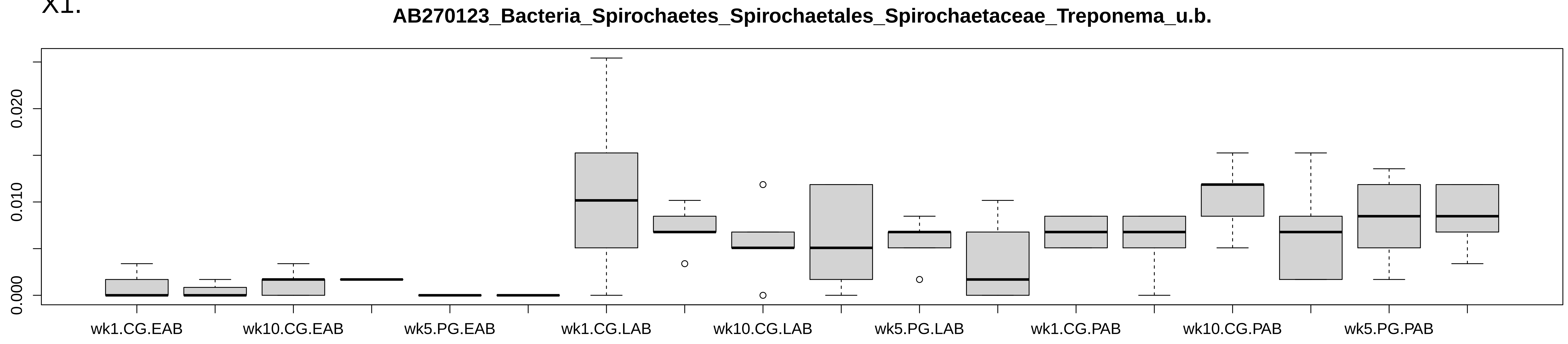

Statistics (p−values): Location: < 0.001; Group: 0.229; L×G: 0.406; Time: 0.770; L×T:0.100; G×T: 0.494; L×G×T: 0.946; Cow: 0.540; T×C: 0.187.

X2.

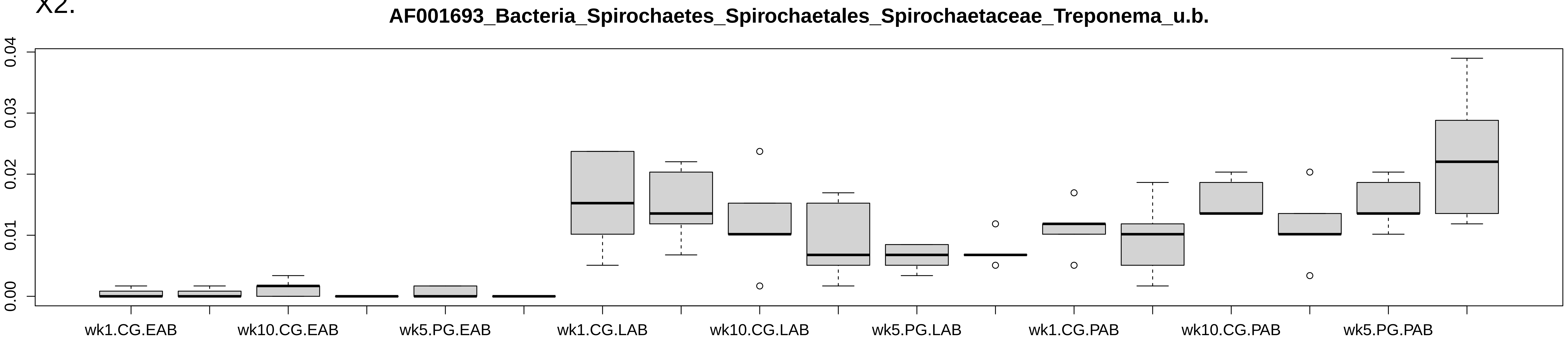

Statistics (p−values): Location: < 0.001; Group: 0.313; L×G: < 0.001; Time: 0.217; L×T:0.029; G×T: 0.549; L×G×T: 0.691; Cow: 0.466; T×C: 0.052.

Y1.

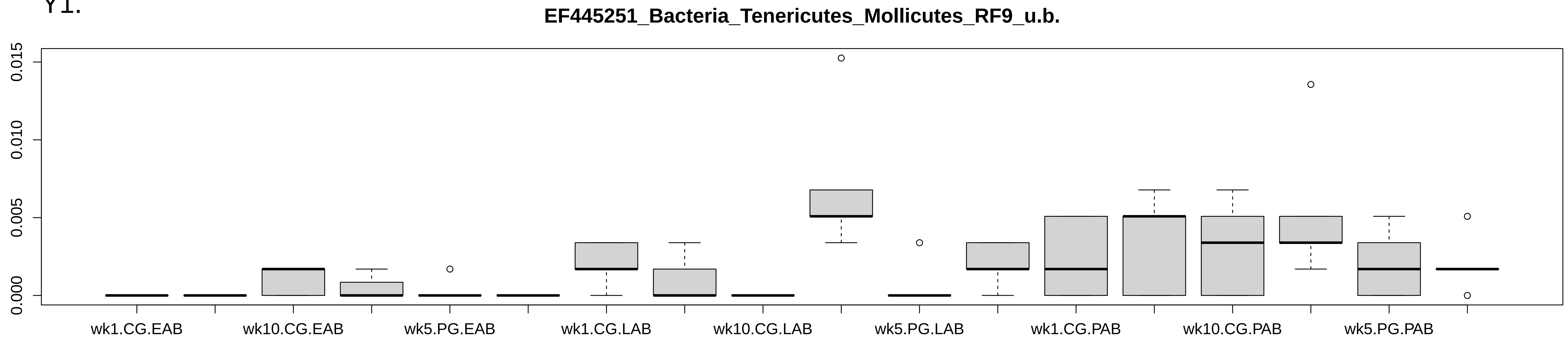

Statistics (p−values): Location: 0.001; Group: 0.277; L×G: 0.095; Time: 0.012; L×T:0.088; G×T: 0.013; L×G×T: 0.626; Cow: 1.000; T×C: 0.697.

Y2.

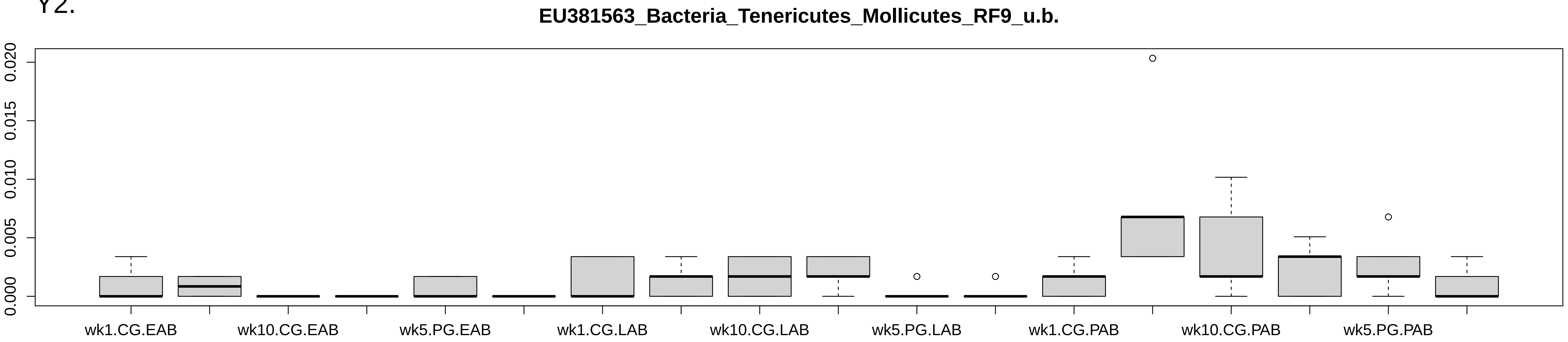

Statistics (p−values): Location: < 0.001; Group: 0.035; L×G: 0.627; Time: 0.101; L×T:0.035; G×T: 0.158; L×G×T: 0.355; Cow: 0.902; T×C: 0.863.

Y3.

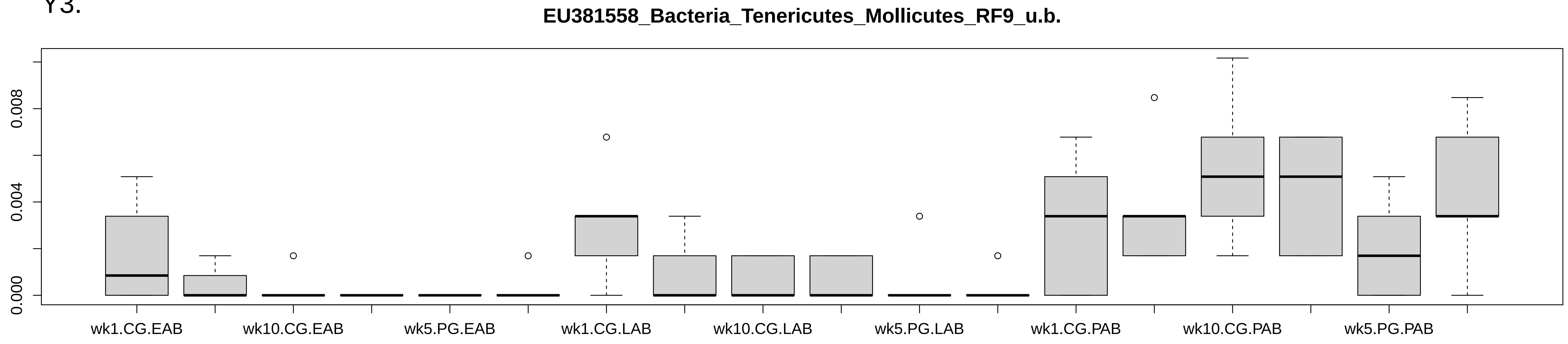

Statistics (p−values): Location: < 0.001; Group: 0.329; L×G: 1.000; Time: 0.355; L×T:0.255; G×T: 1.000; L×G×T: 0.275; Cow: 0.077; T×C: 0.605.

Y4.

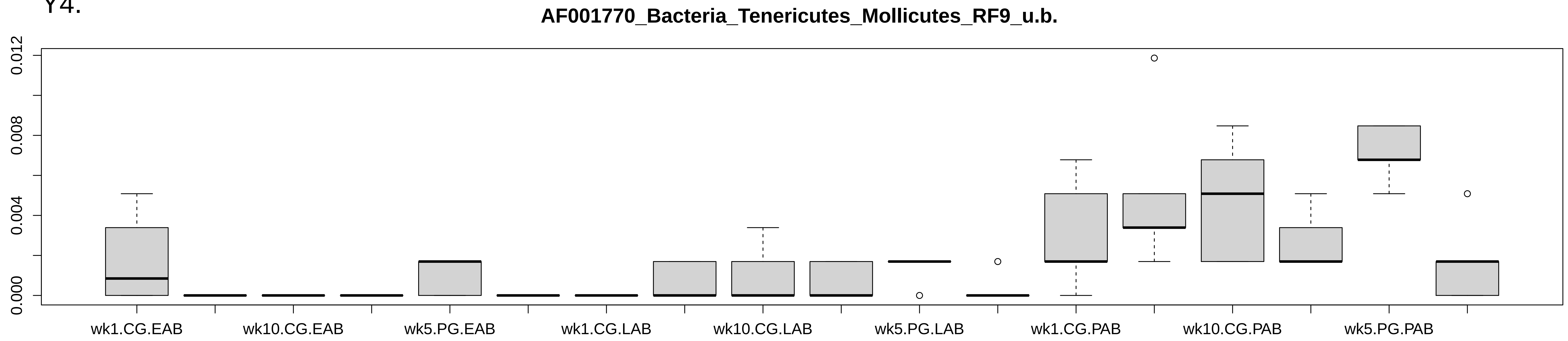

Statistics (p−values): Location: < 0.001; Group: 0.843; L×G: 0.709; Time: 0.003; L×T:0.024; G×T: 0.031; L×G×T: 0.158; Cow: 0.843; T×C: 0.939.
