# Supplementary material for: Alterations in the Rumen Liquid-, Particle- and Epithelium-Associated Microbiota of Dairy Cows during the Transition from a Silage- and Concentrate-Based Ration to Pasture in Spring
Source: Front Microbiol. 2017 May 2;8:744. doi: 10.3389/fmicb.2017.00744 (PMC5411454; doi:10.3389/fmicb.2017.00744)
Supplement: Supplementary file 2 [file DataSheet2.PDF]

EF112194_Archaea_Euryarchaeota_Methanobacteria_Methanobacteriales_Methanobacteriaceae_Methanobrevibacter_u.a.

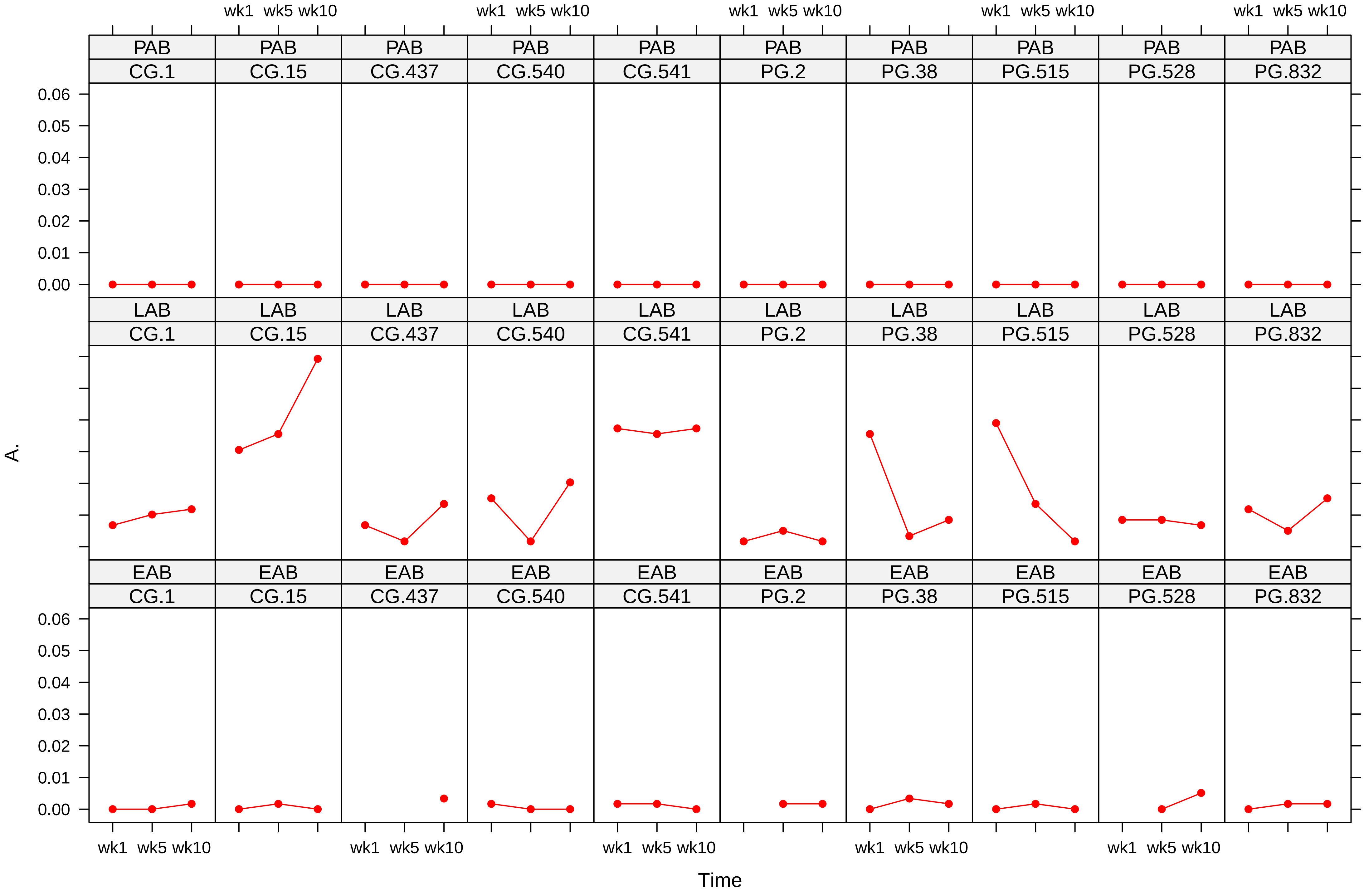

EU779121_Bacteria_Actinobacteria_Bifidobacteriales_Bifidobacteriaceae_u.b.

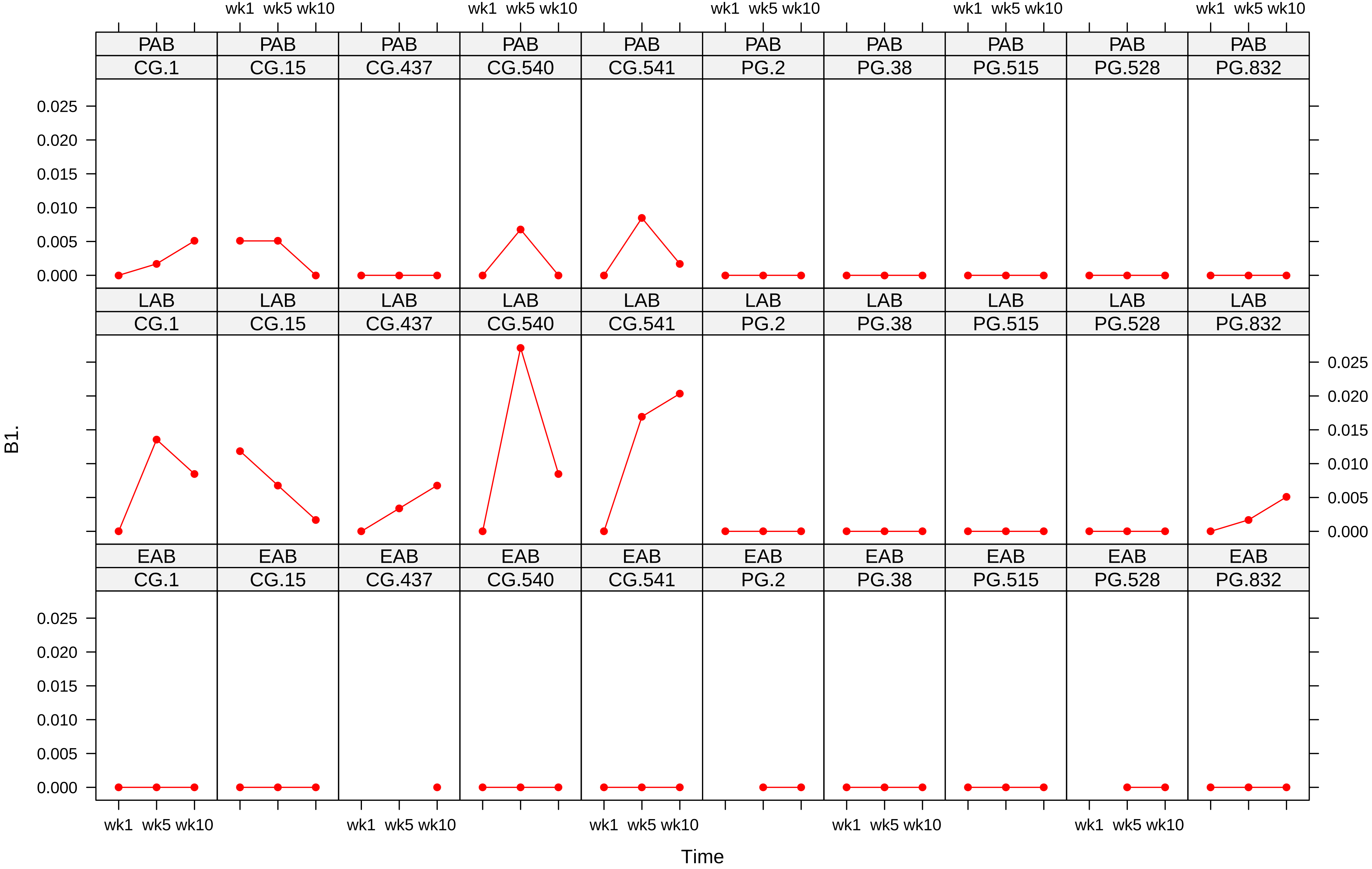

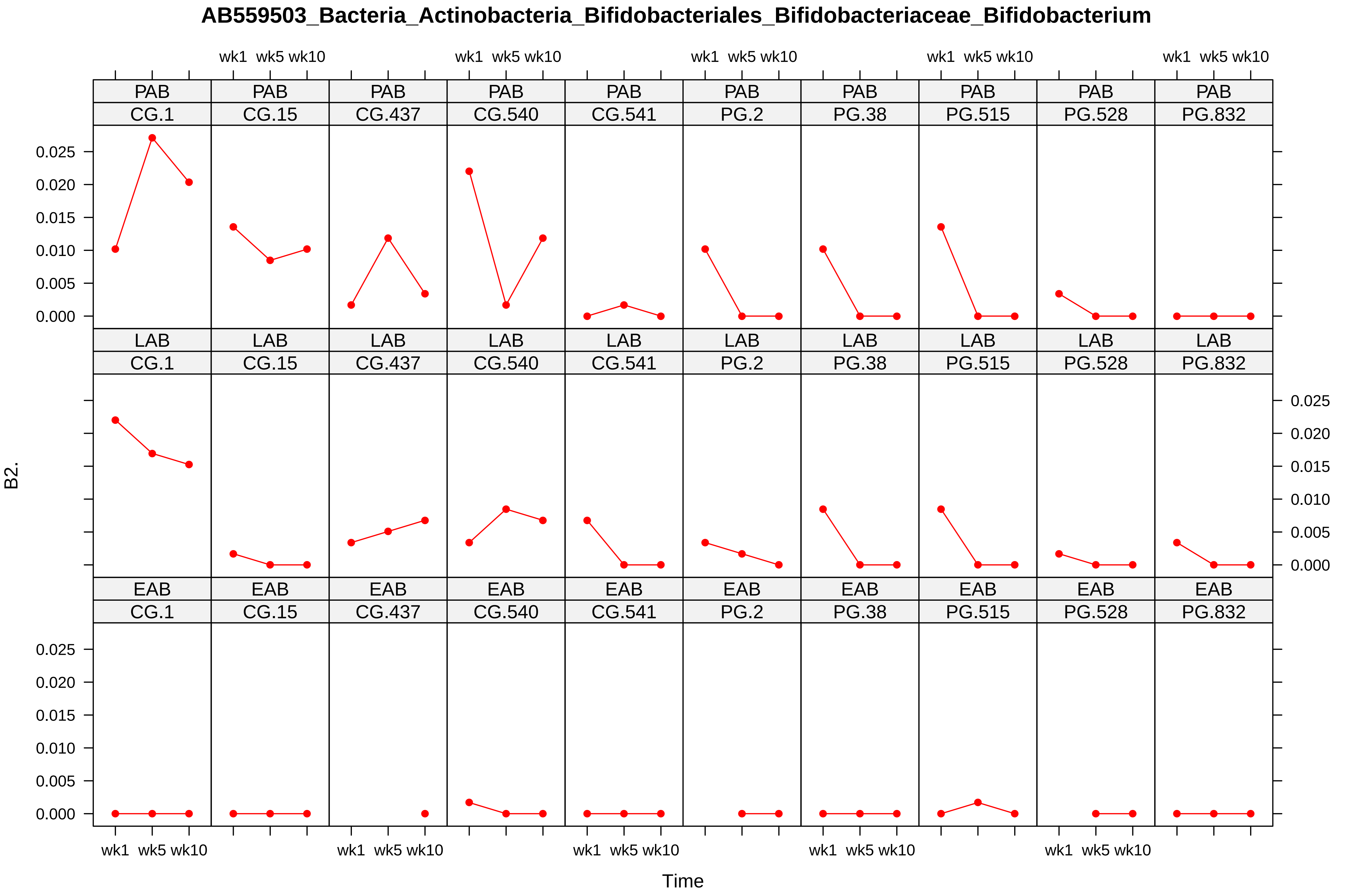
AB559503_Bacteria_Actinobacteria_Bifidobacteriales_Bifidobacteriaceae_Bifidobacterium
PAB
LAB
EAB
CG.1
CG.15
CG.437
CG.540
CG.541
PG.2
PG.38
PG.515
PG.528
PG.832
0.025
0.020
0.015
0.010
0.005
0.000
wk1 wk5 wk10
B2.
Time

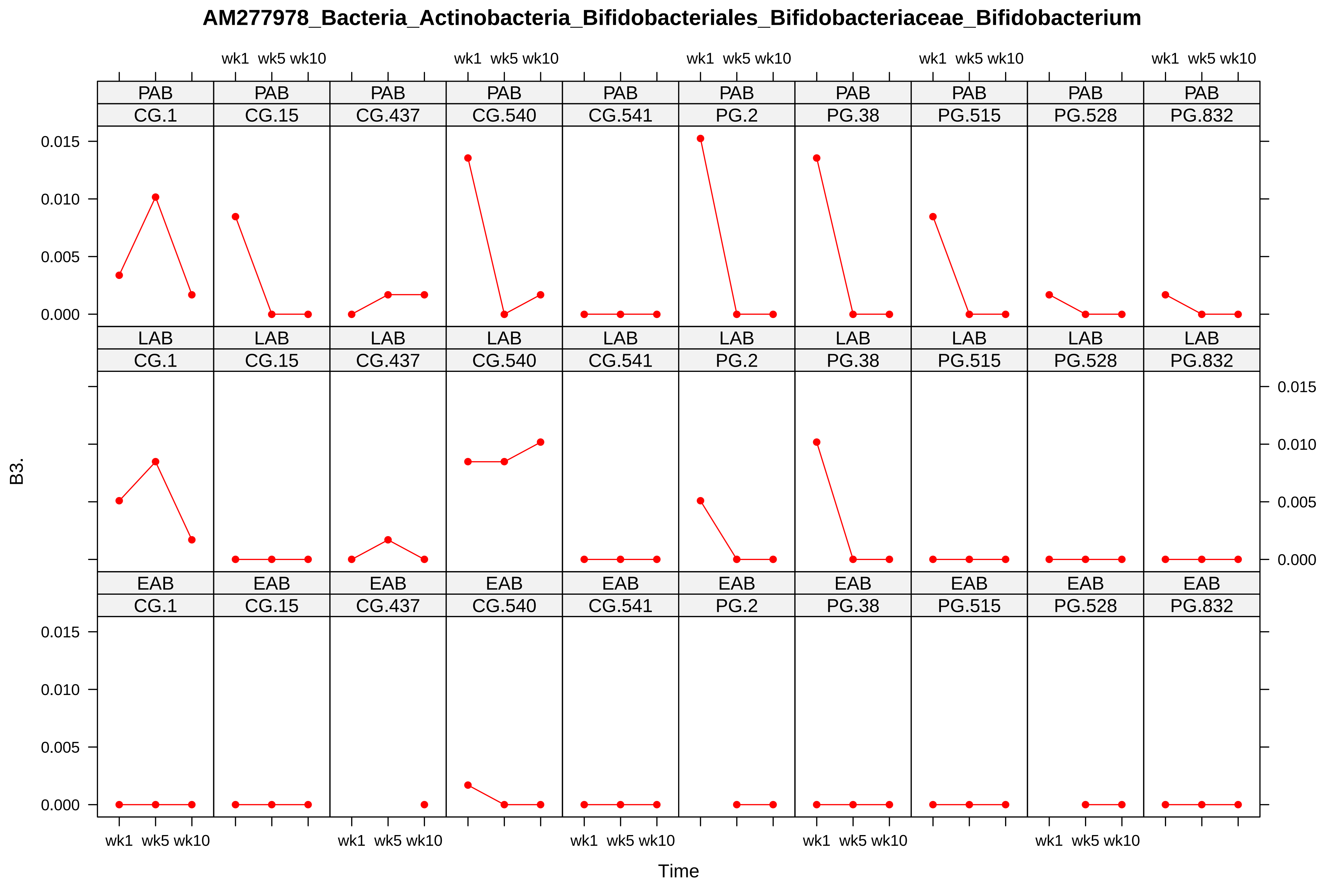
AM277978_Bacteria_Actinobacteria_Bifidobacteriales_Bifidobacteriaceae_Bifidobacterium
PAB
LAB
EAB
CG.1
CG.15
CG.437
CG.540
CG.541
PG.2
PG.38
PG.515
PG.528
PG.832
0.000
0.005
0.010
0.015
wk1
wk5
wk10
B3.
Time

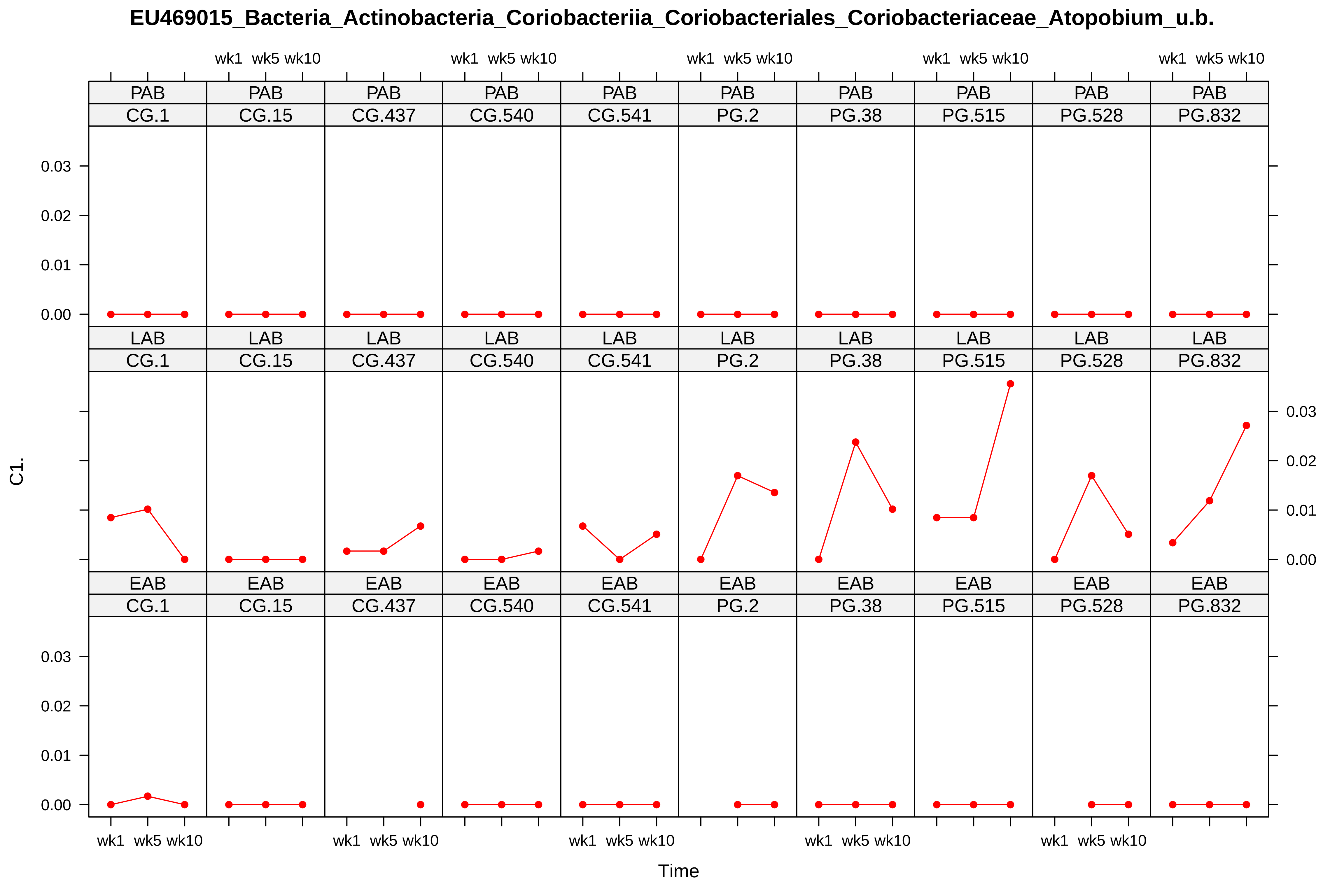
EU469015_Bacteria_Actinobacteria_Coriobacteriia_Coriobacteriales_Coriobacteriaceae_Atopobium_u.b.
PAB
LAB
EAB
CG.1
CG.15
CG.437
CG.540
CG.541
PG.2
PG.38
PG.515
PG.528
PG.832
0.00
0.01
0.02
0.03
wk1
wk5
wk10
C1.
Time

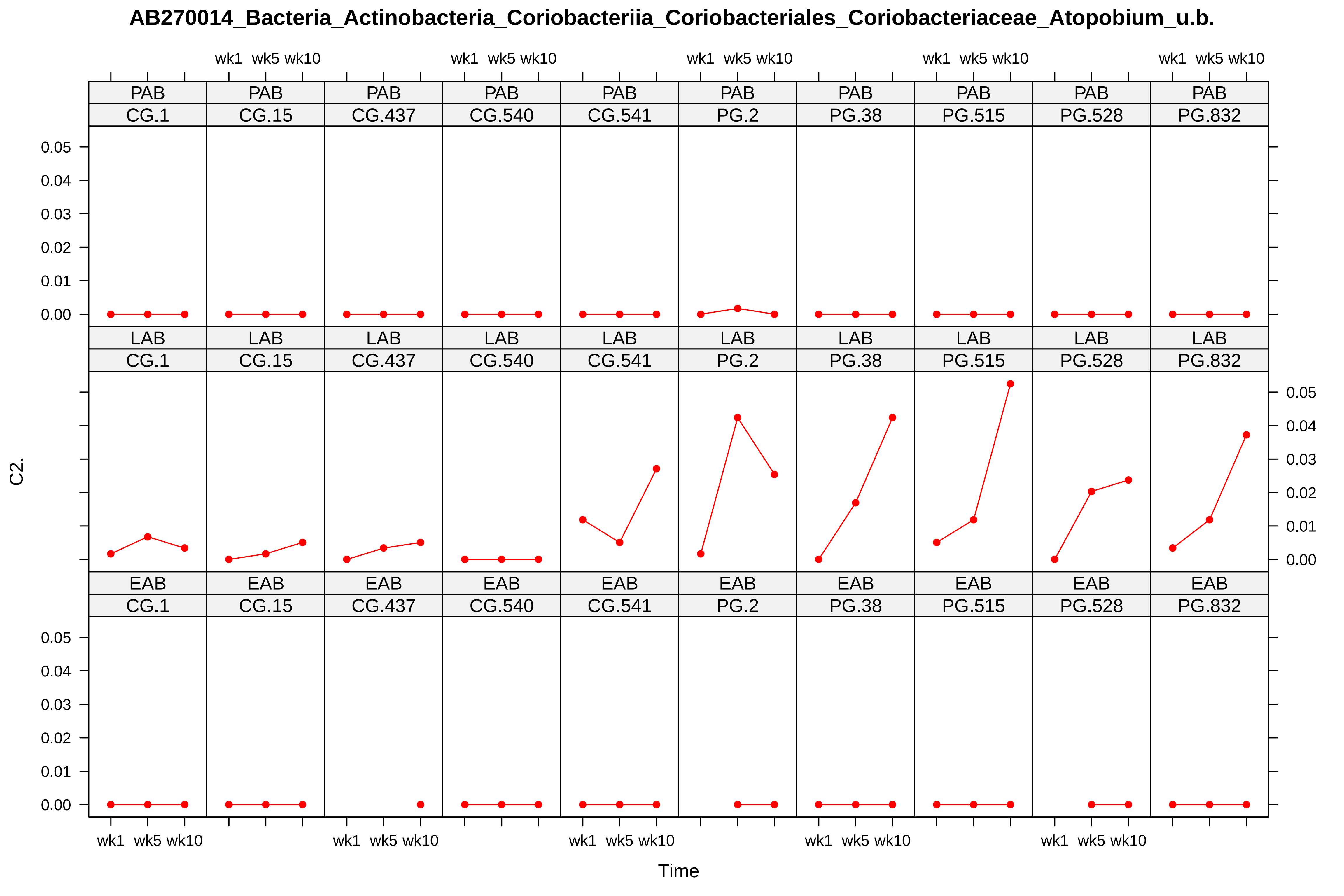
AB270014_Bacteria_Actinobacteria_Coriobacteriia_Coriobacteriales_Coriobacteriaceae_Atopobium_u.b.
PAB
LAB
EAB
CG.1
CG.15
CG.437
CG.540
CG.541
PG.2
PG.38
PG.515
PG.528
PG.832
wk1 wk5 wk10
0.00
0.01
0.02
0.03
0.04
0.05
C2.
Time

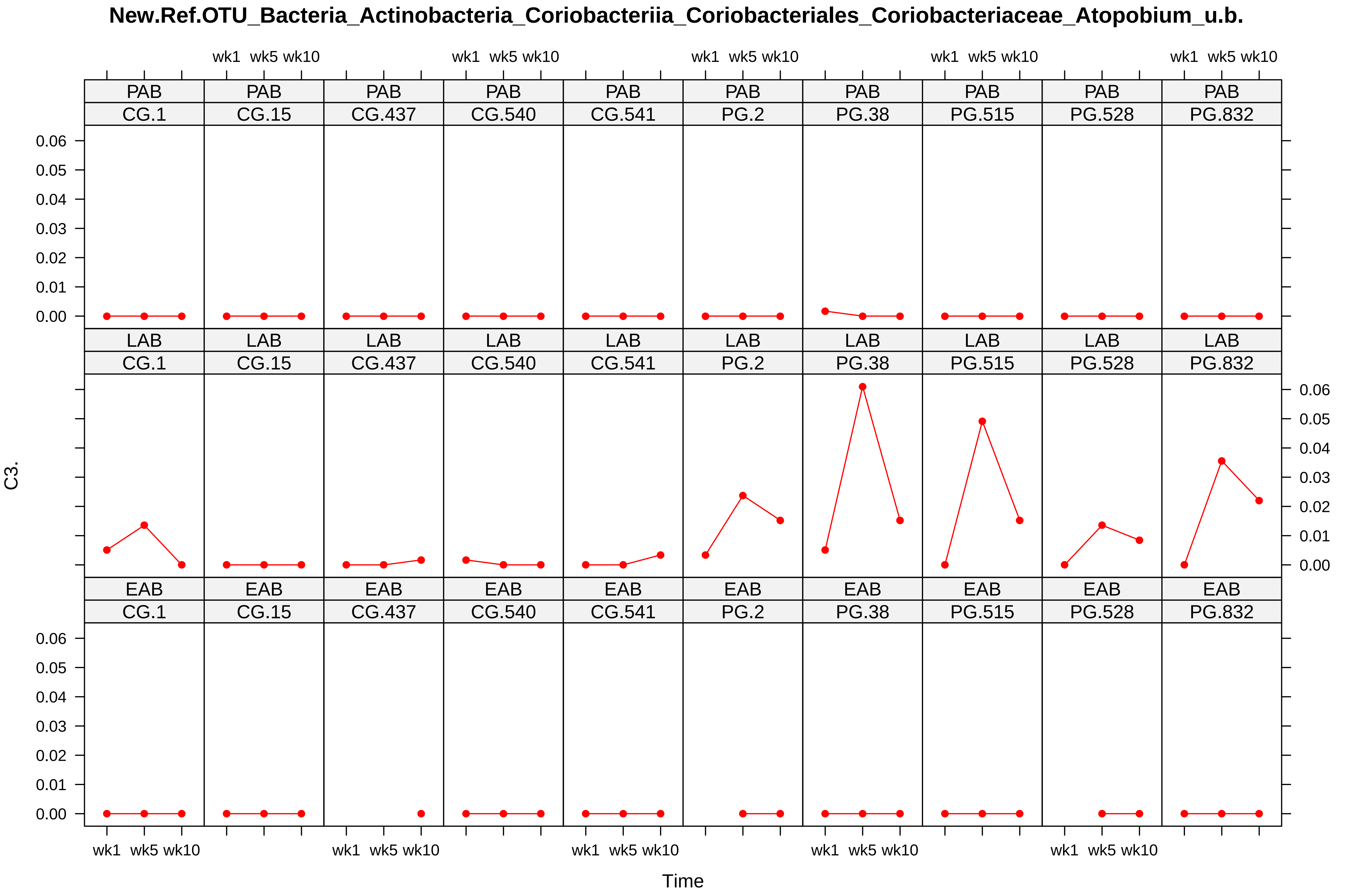

New.Ref.OTU_Bacteria_Actinobacteria_Coriobacteriia_Coriobacteriales_Coriobacteriaceae_Atopobium_u.b.
PAB
LAB
EAB
CG.1
CG.15
CG.437
CG.540
CG.541
PG.2
PG.38
PG.515
PG.528
PG.832
wk1 wk5 wk10
0.00
0.01
0.02
0.03
0.04
0.05
0.06
C3.
Time

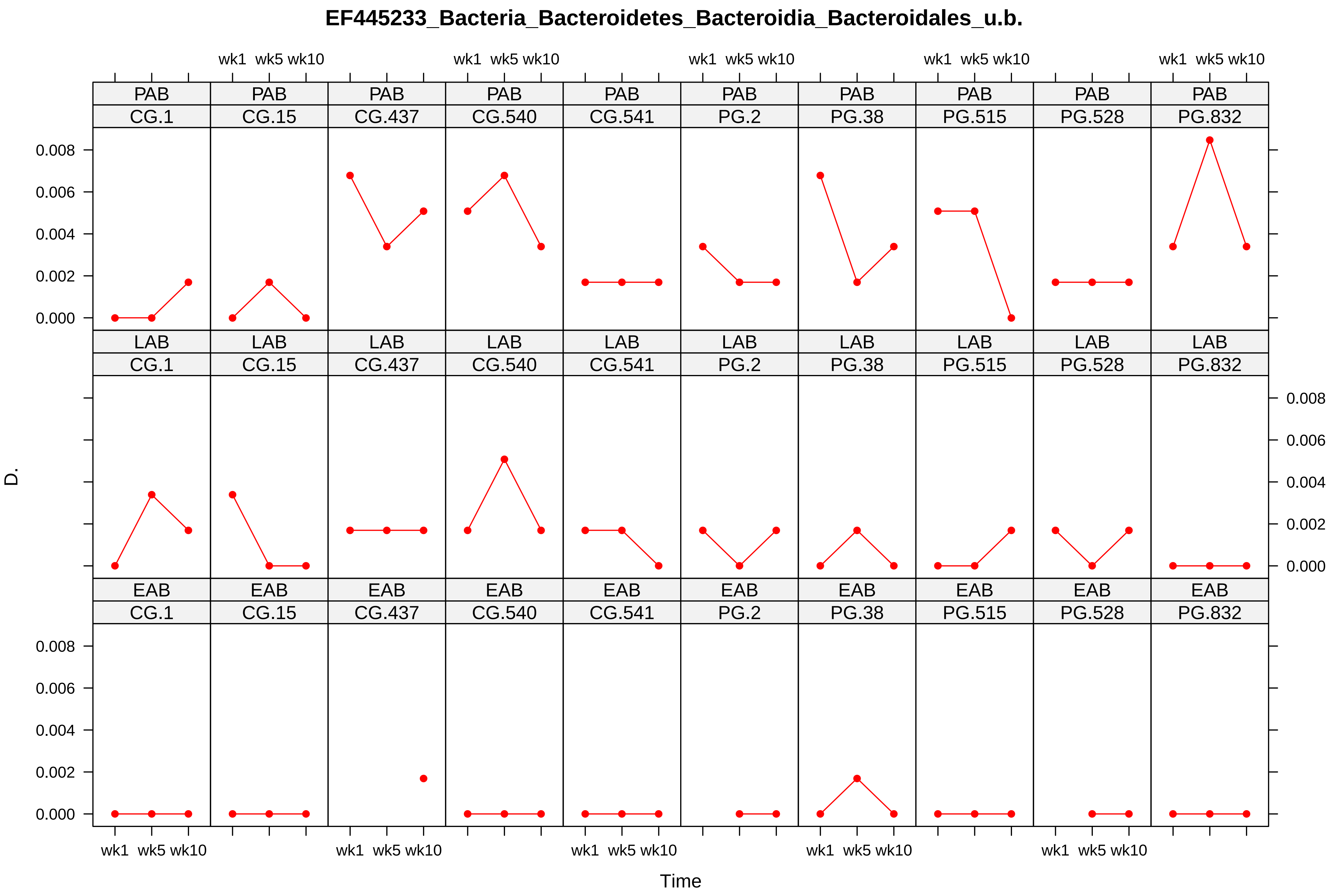
EF445233_Bacteria_Bacteroidetes_Bacteroidia_Bacteroidales_u.b.
PAB
LAB
EAB
CG.1
CG.15
CG.437
CG.540
CG.541
PG.2
PG.38
PG.515
PG.528
PG.832
0.000
0.002
0.004
0.006
0.008
wk1 wk5 wk10
D.
Time

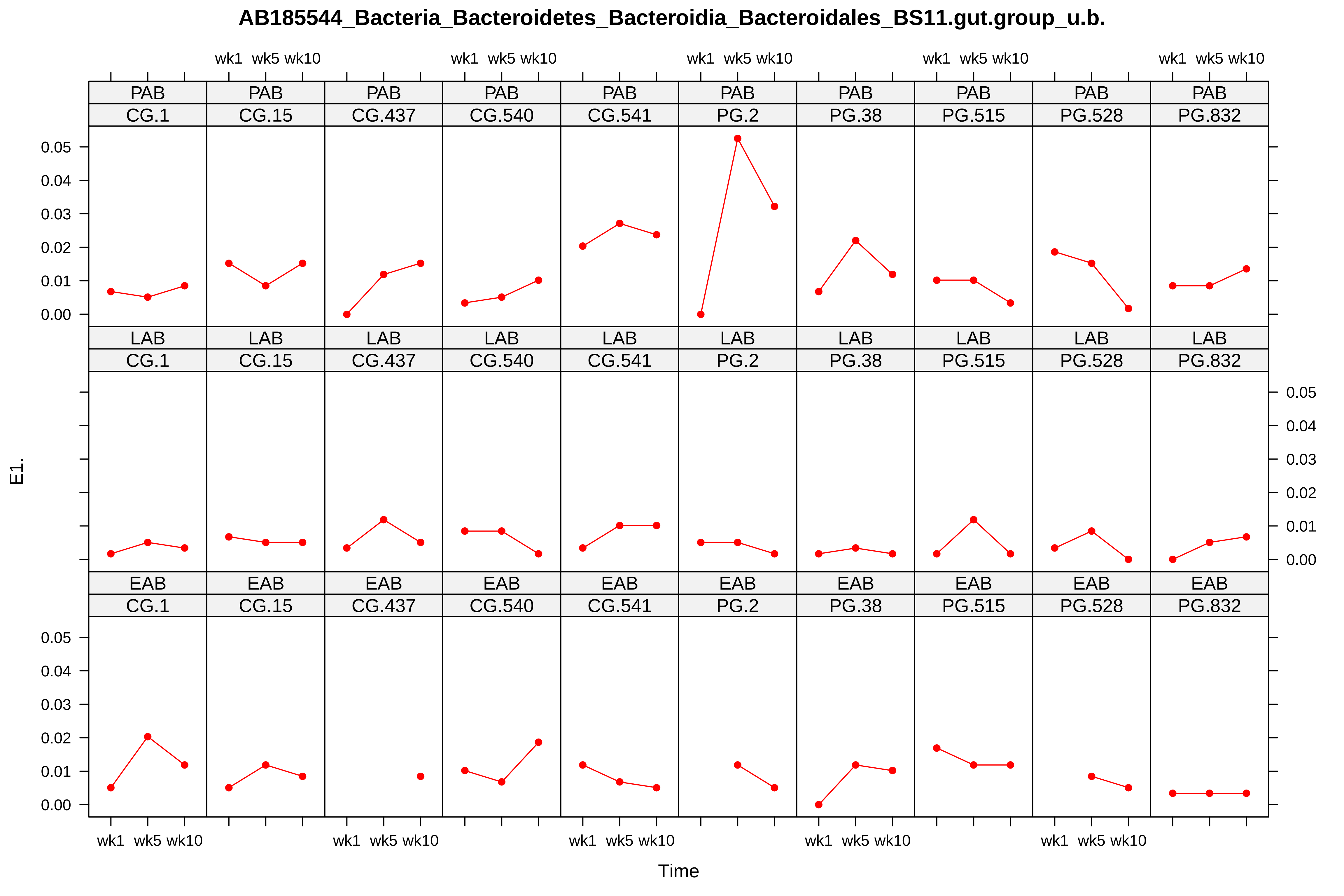
AB185544_Bacteria_Bacteroidetes_Bacteroidia_Bacteroidales_BS11.gut.group_u.b.
PAB CG.1
PAB CG.15
PAB CG.437
PAB CG.540
PAB CG.541
PAB PG.2
PAB PG.38
PAB PG.515
PAB PG.528
PAB PG.832
LAB CG.1
LAB CG.15
LAB CG.437
LAB CG.540
LAB CG.541
LAB PG.2
LAB PG.38
LAB PG.515
LAB PG.528
LAB PG.832
EAB CG.1
EAB CG.15
EAB CG.437
EAB CG.540
EAB CG.541
EAB PG.2
EAB PG.38
EAB PG.515
EAB PG.528
EAB PG.832
0.00
0.01
0.02
0.03
0.04
0.05
wk1 wk5 wk10
E1.
Time

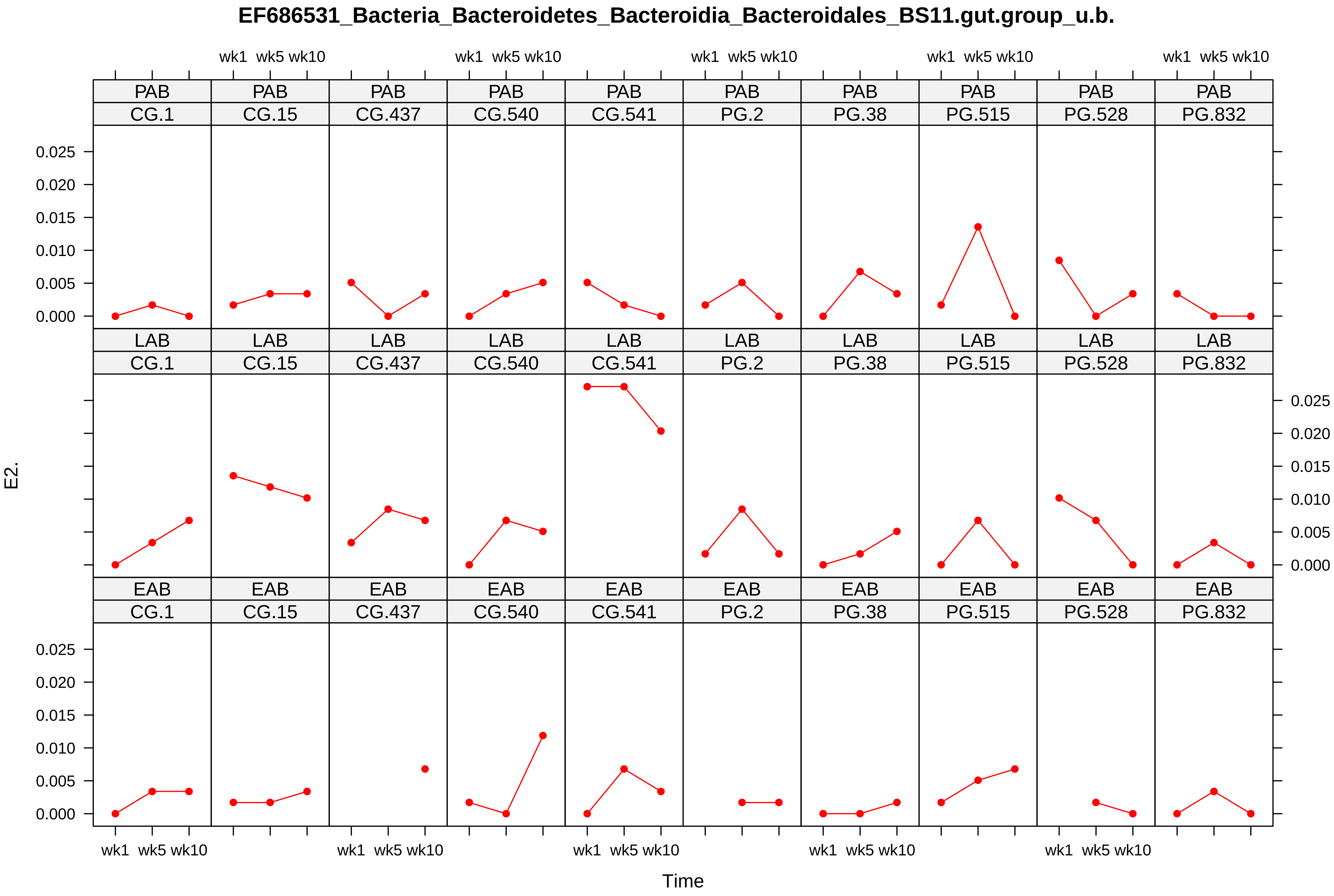

EF686531_Bacteria_Bacteroidetes_Bacteroidia_Bacteroidales_BS11.gut.group_u.b.
PAB
LAB
EAB
CG.1
CG.15
CG.437
CG.540
CG.541
PG.2
PG.38
PG.515
PG.528
PG.832
0.000
0.005
0.010
0.015
0.020
0.025
wk1 wk5 wk10
E2.
Time

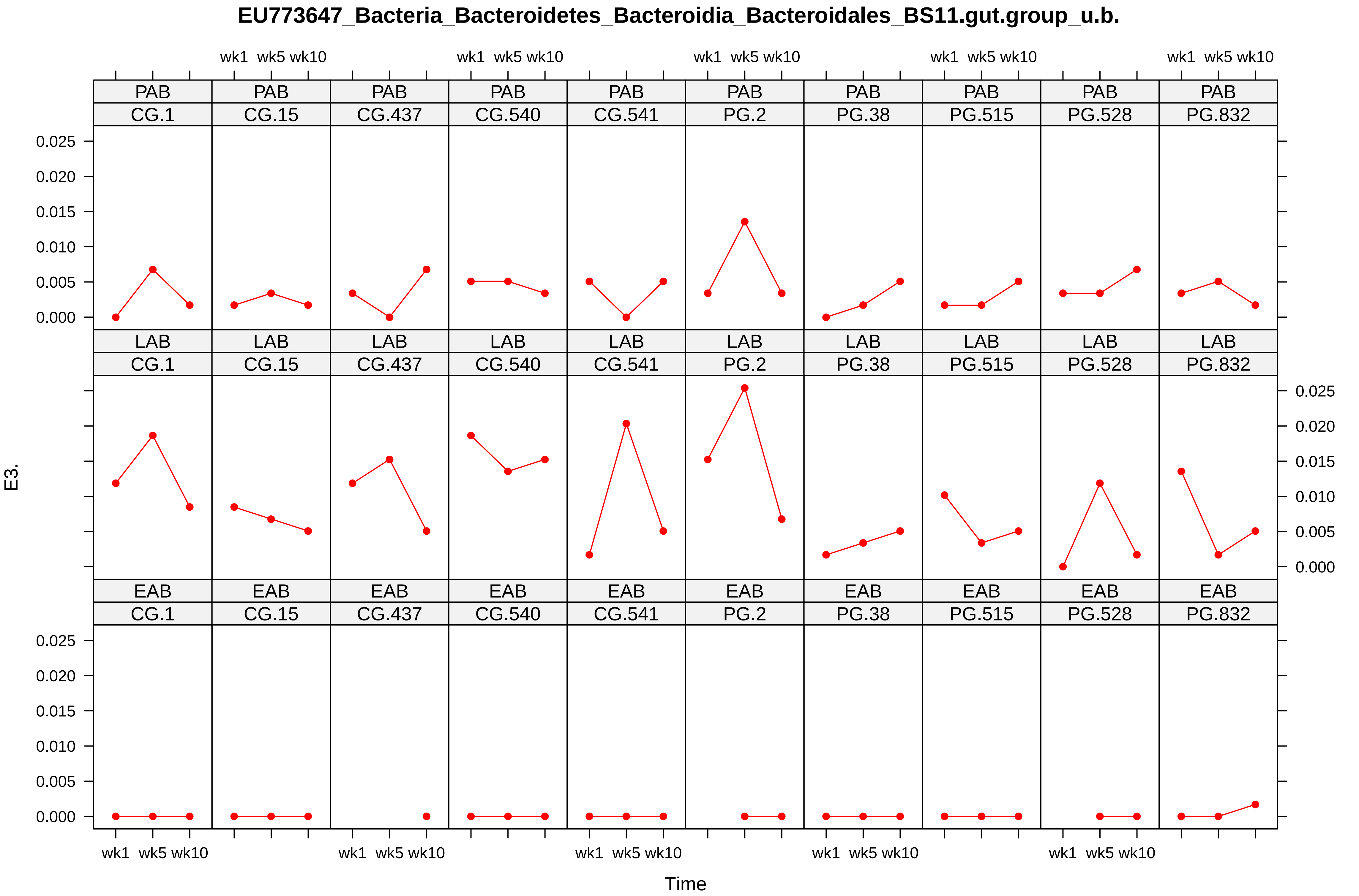
EU773647_Bacteria_Bacteroidetes_Bacteroidia_Bacteroidales_BS11.gut.group_u.b.
PAB
LAB
EAB
CG.1
CG.15
CG.437
CG.540
CG.541
PG.2
PG.38
PG.515
PG.528
PG.832
wk1 wk5 wk10
0.000
0.005
0.010
0.015
0.020
0.025
E3.
Time

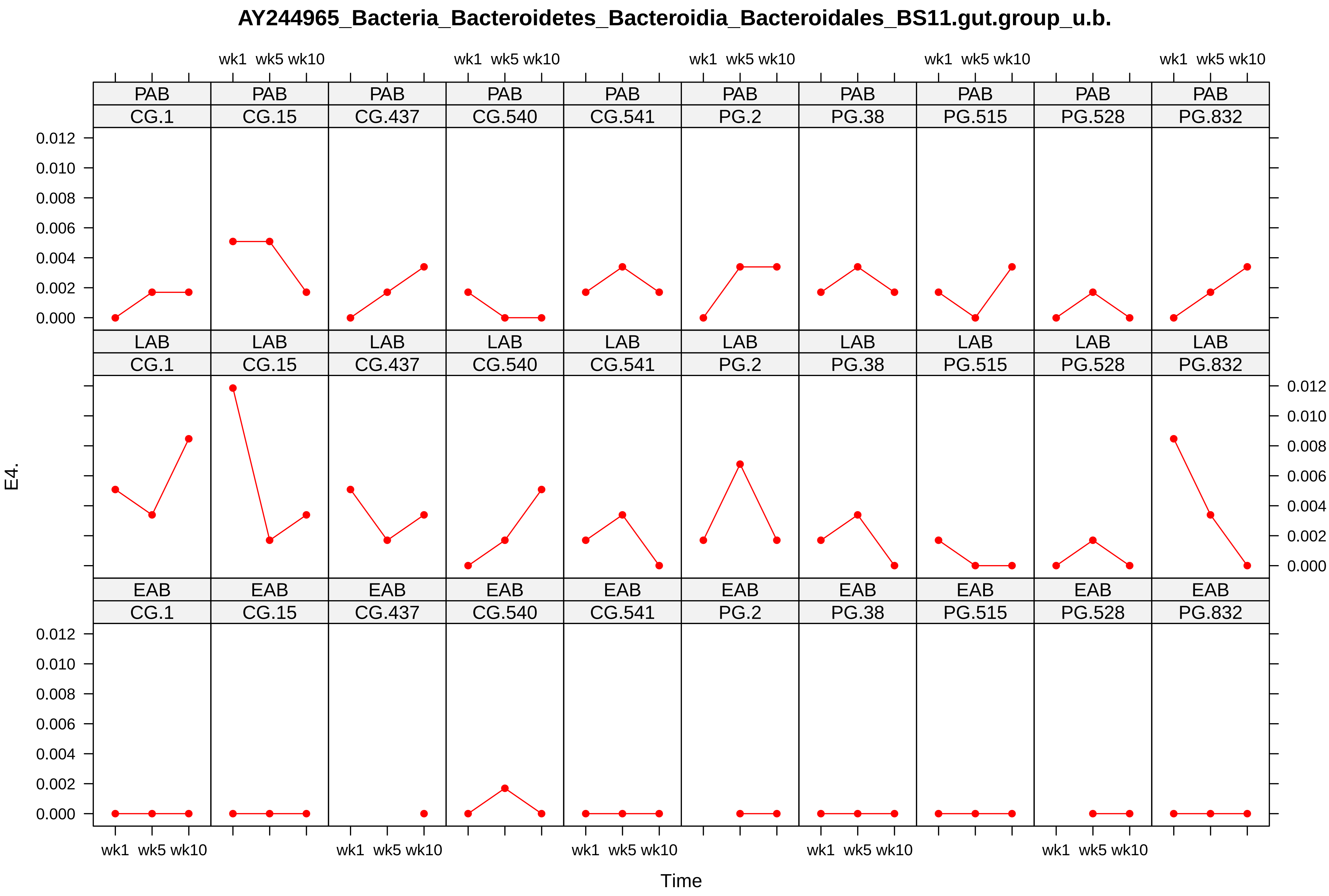
AY244965_Bacteria_Bacteroidetes_Bacteroidia_Bacteroidales_BS11.gut.group_u.b.
PAB
LAB
EAB
CG.1
CG.15
CG.437
CG.540
CG.541
PG.2
PG.38
PG.515
PG.528
PG.832
0.000
0.002
0.004
0.006
0.008
0.010
0.012
wk1 wk5 wk10
E4.
Time

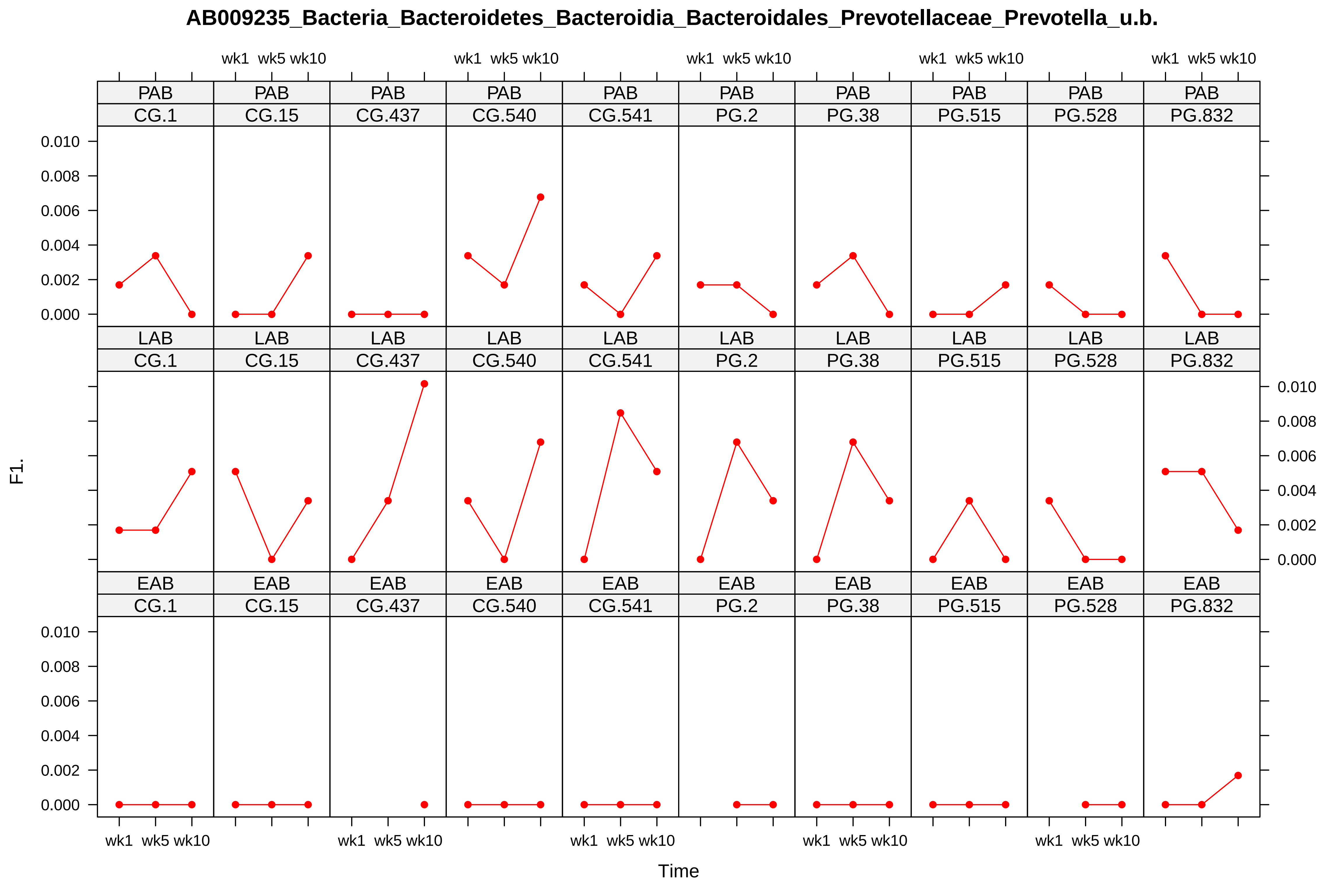
AB009235_Bacteria_Bacteroidetes_Bacteroidia_Bacteroidales_Prevotellaceae_Prevotella_u.b.
PAB
LAB
EAB
CG.1
CG.15
CG.437
CG.540
CG.541
PG.2
PG.38
PG.515
PG.528
PG.832
0.000
0.002
0.004
0.006
0.008
0.010
wk1 wk5 wk10
F1.
Time

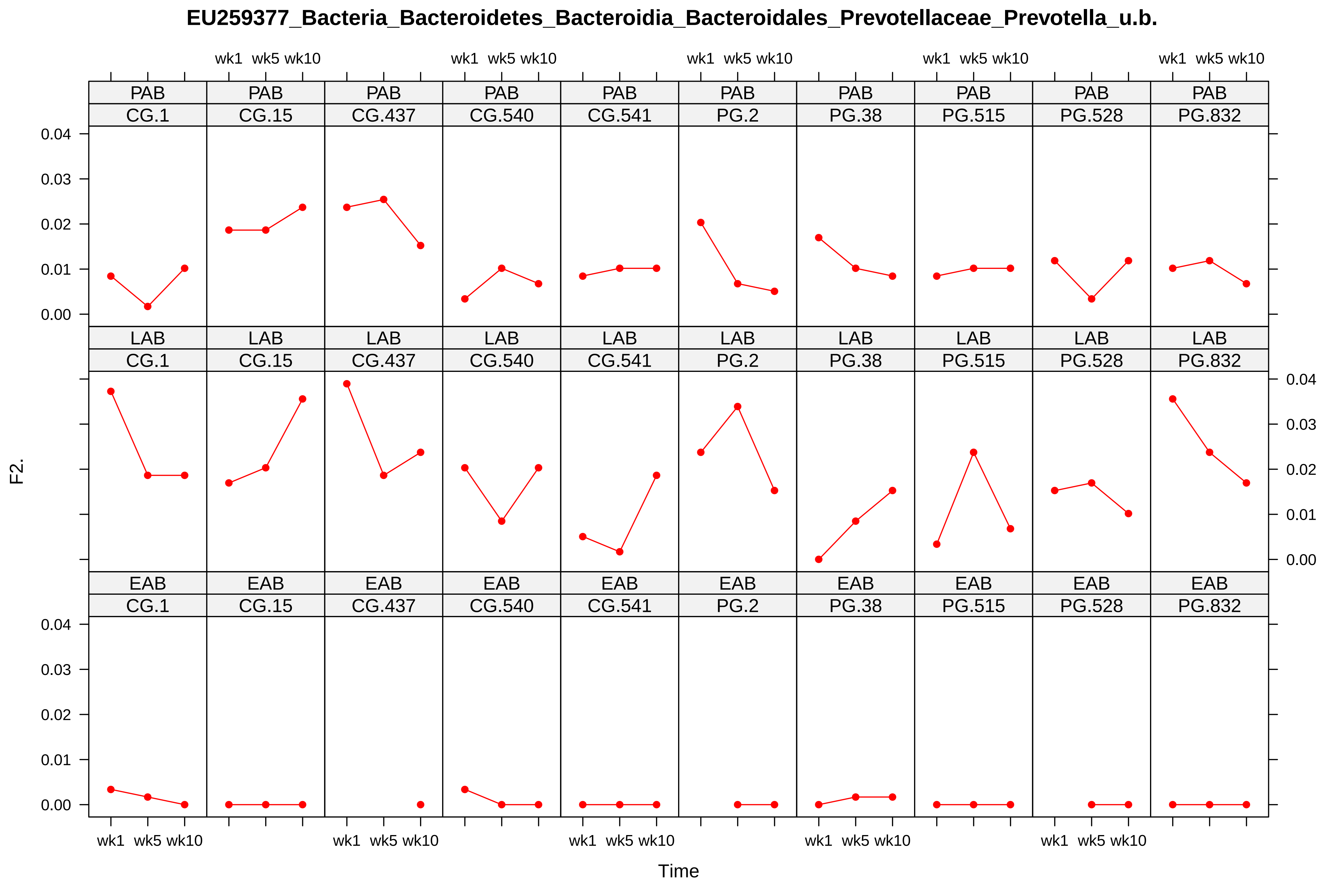
EU259377_Bacteria_Bacteroidetes_Bacteroidia_Bacteroidales_Prevotellaceae_Prevotella_u.b.
PAB
LAB
EAB
CG.1
CG.15
CG.437
CG.540
CG.541
PG.2
PG.38
PG.515
PG.528
PG.832
wk1
wk5
wk10
0.00
0.01
0.02
0.03
0.04
F2.
Time

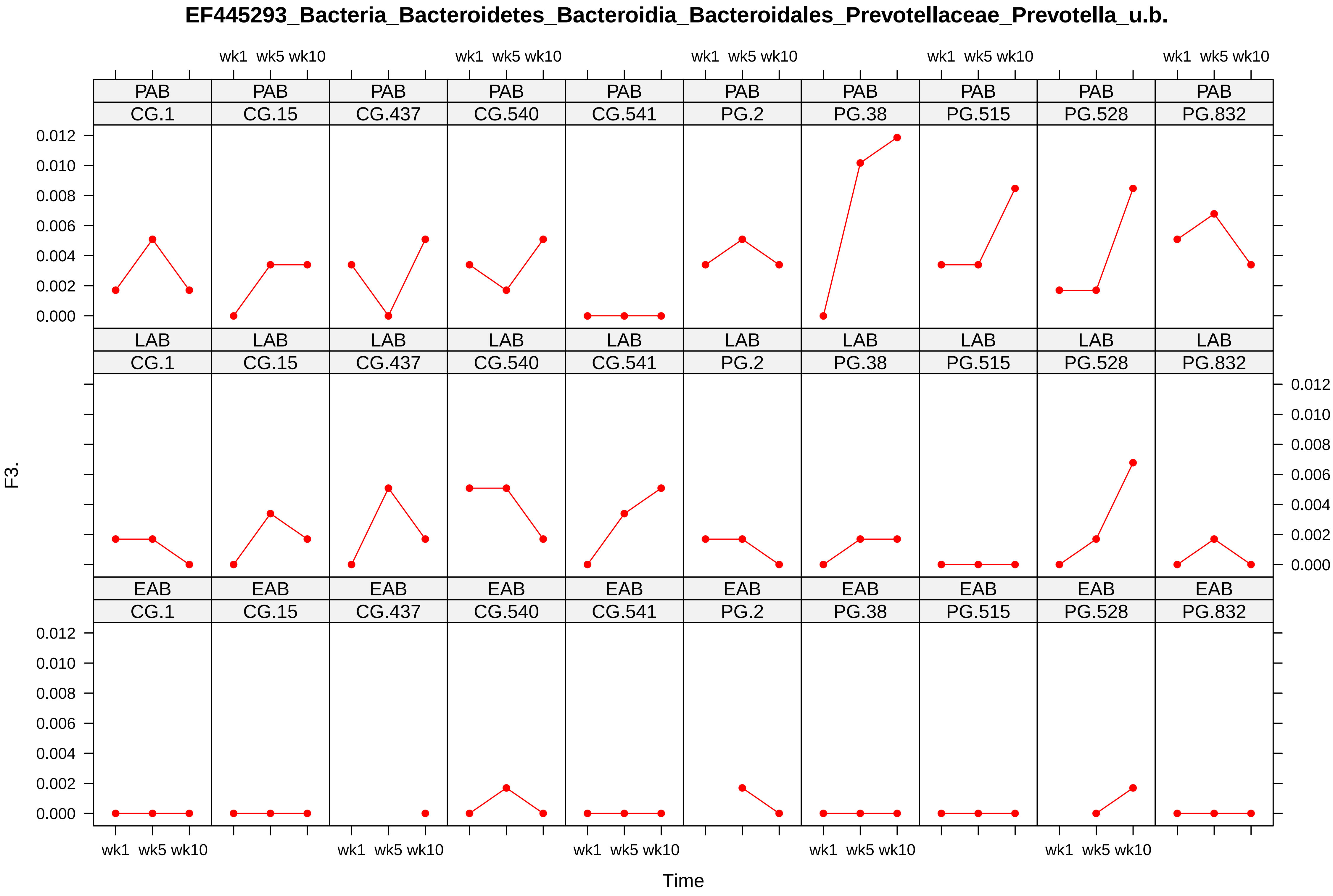
EF445293_Bacteria_Bacteroidetes_Bacteroidia_Bacteroidales_Prevotellaceae_Prevotella_u.b.
PAB
LAB
EAB
CG.1
CG.15
CG.437
CG.540
CG.541
PG.2
PG.38
PG.515
PG.528
PG.832
wk1 wk5 wk10
0.000
0.002
0.004
0.006
0.008
0.010
0.012
F3.
Time

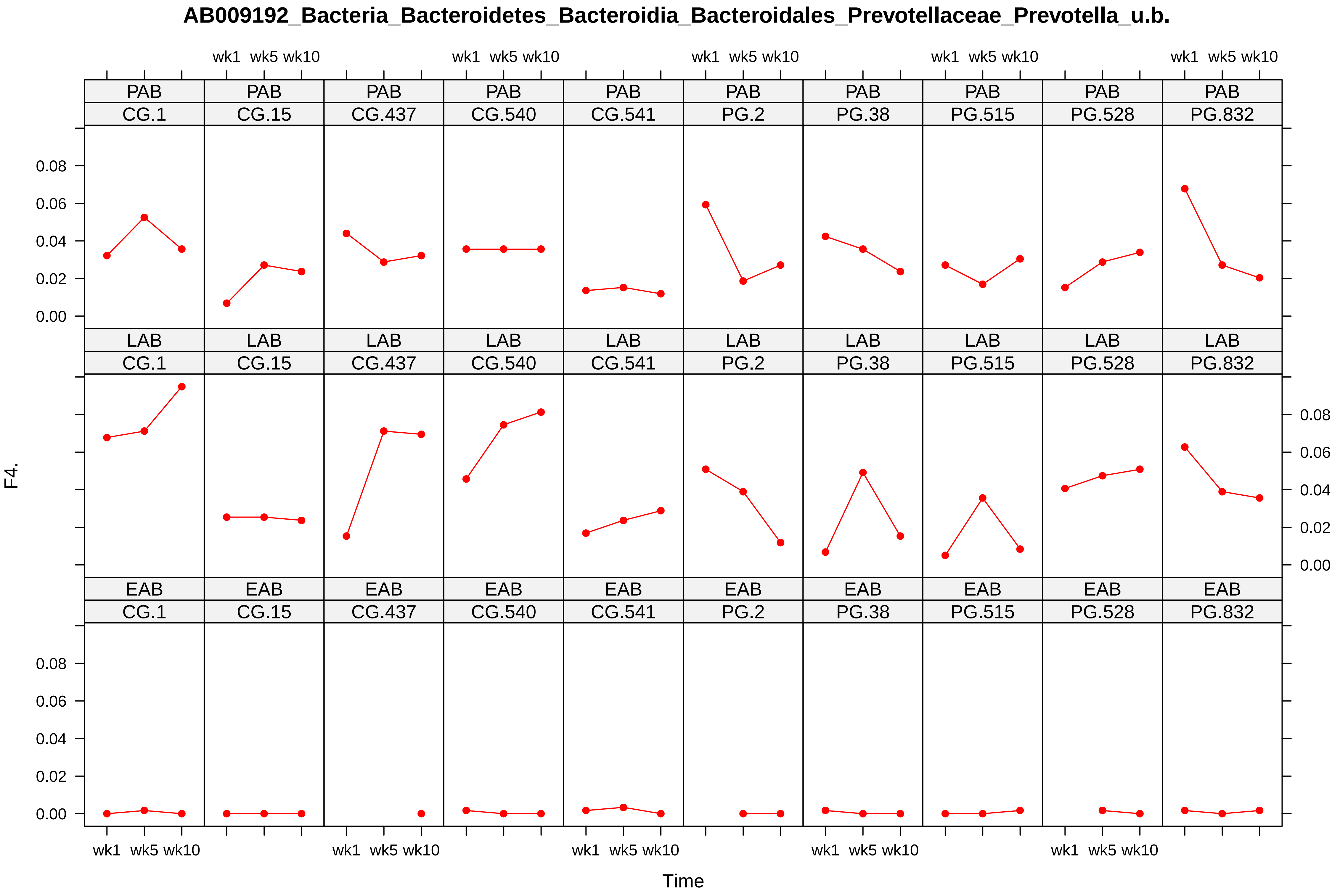

AB009192_Bacteria_Bacteroidetes_Bacteroidia_Bacteroidales_Prevotellaceae_Prevotella_u.b.
PAB
LAB
EAB
CG.1
CG.15
CG.437
CG.540
CG.541
PG.2
PG.38
PG.515
PG.528
PG.832
0.00
0.02
0.04
0.06
0.08
wk1
wk5
wk10
F4.
Time

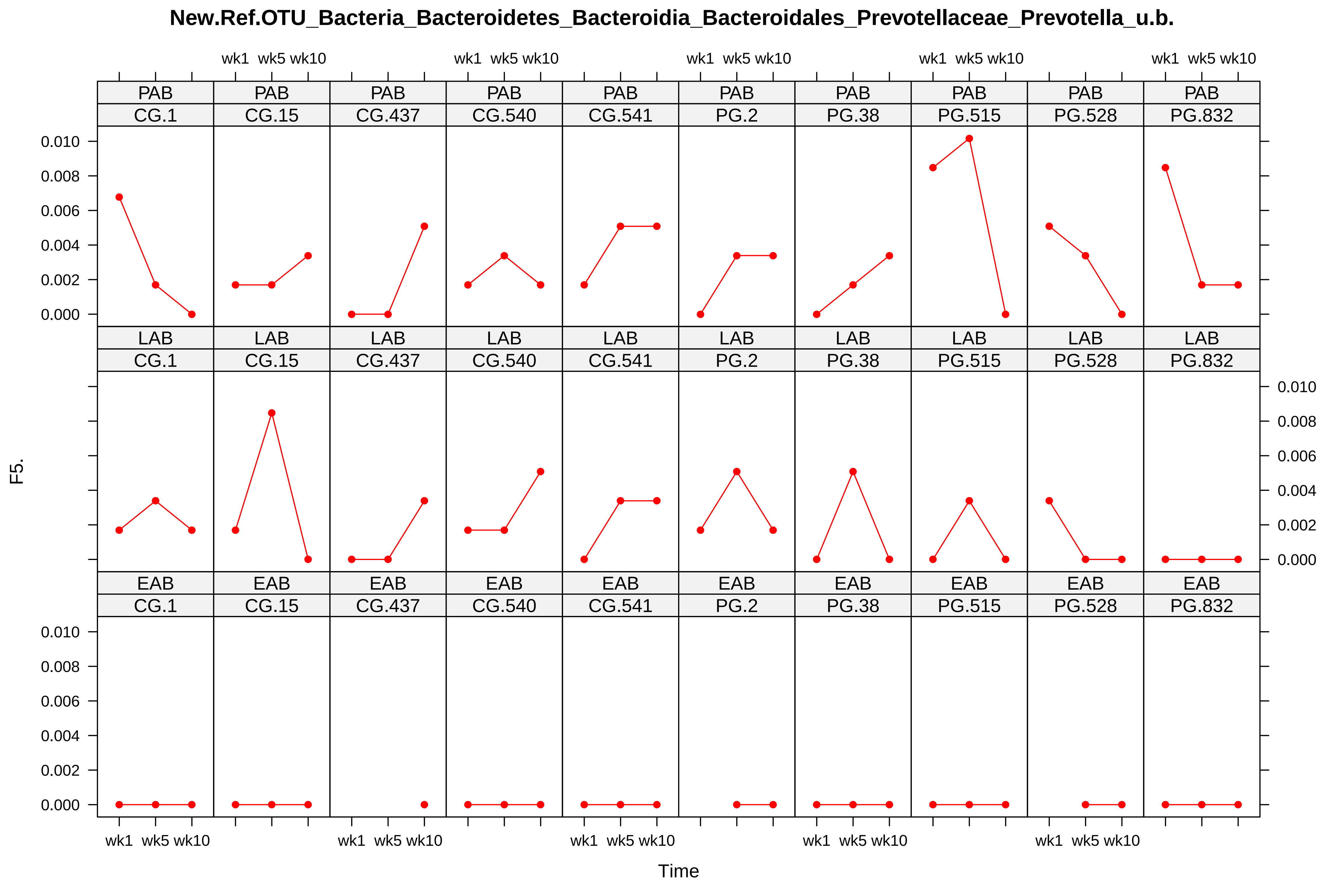
New.Ref.OTU_Bacteria_Bacteroidetes_Bacteroidia_Bacteroidales_Prevotellaceae_Prevotella_u.b.
PAB
LAB
EAB
CG.1
CG.15
CG.437
CG.540
CG.541
PG.2
PG.38
PG.515
PG.528
PG.832
0.000
0.002
0.004
0.006
0.008
0.010
wk1 wk5 wk10
F5.
Time

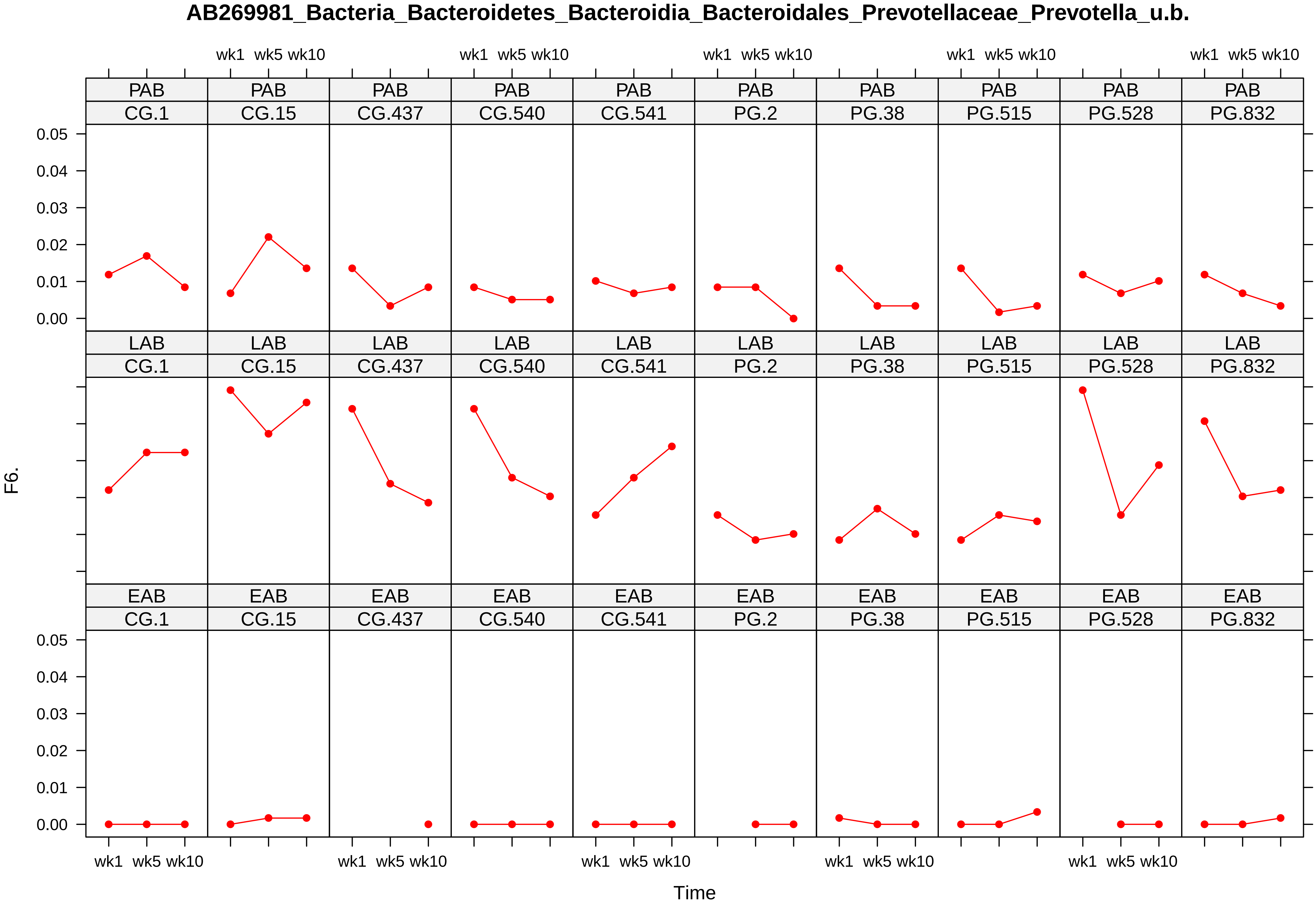
AB269981_Bacteria_Bacteroidetes_Bacteroidia_Bacteroidales_Prevotellaceae_Prevotella_u.b.
PAB
LAB
EAB
CG.1
CG.15
CG.437
CG.540
CG.541
PG.2
PG.38
PG.515
PG.528
PG.832
wk1 wk5 wk10
0.00
0.01
0.02
0.03
0.04
0.05
F6.
Time

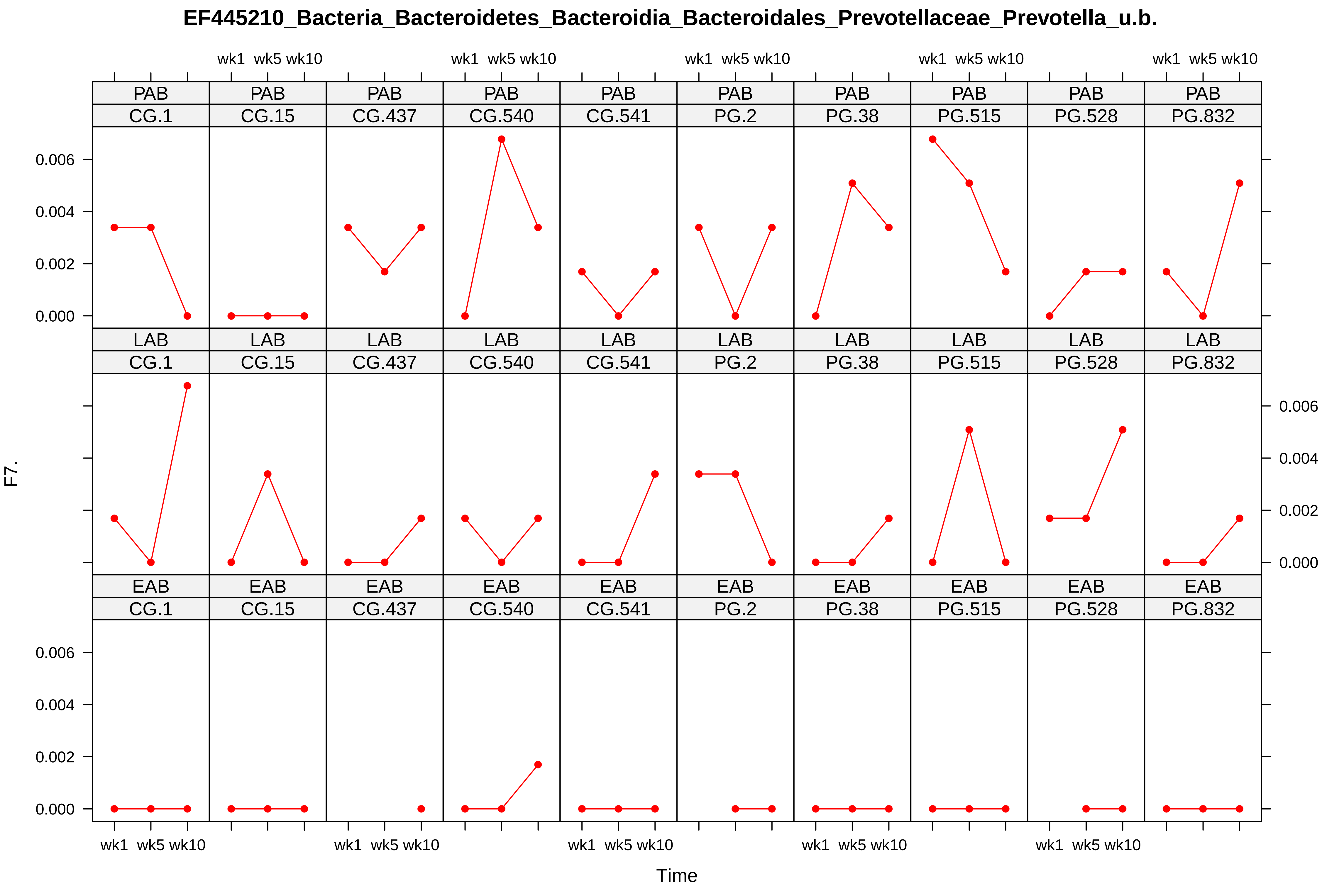

EF445210_Bacteria_Bacteroidetes_Bacteroidia_Bacteroidales_Prevotellaceae_Prevotella_u.b.
PAB
LAB
EAB
CG.1
CG.15
CG.437
CG.540
CG.541
PG.2
PG.38
PG.515
PG.528
PG.832
0.000
0.002
0.004
0.006
wk1 wk5 wk10
F7.
Time

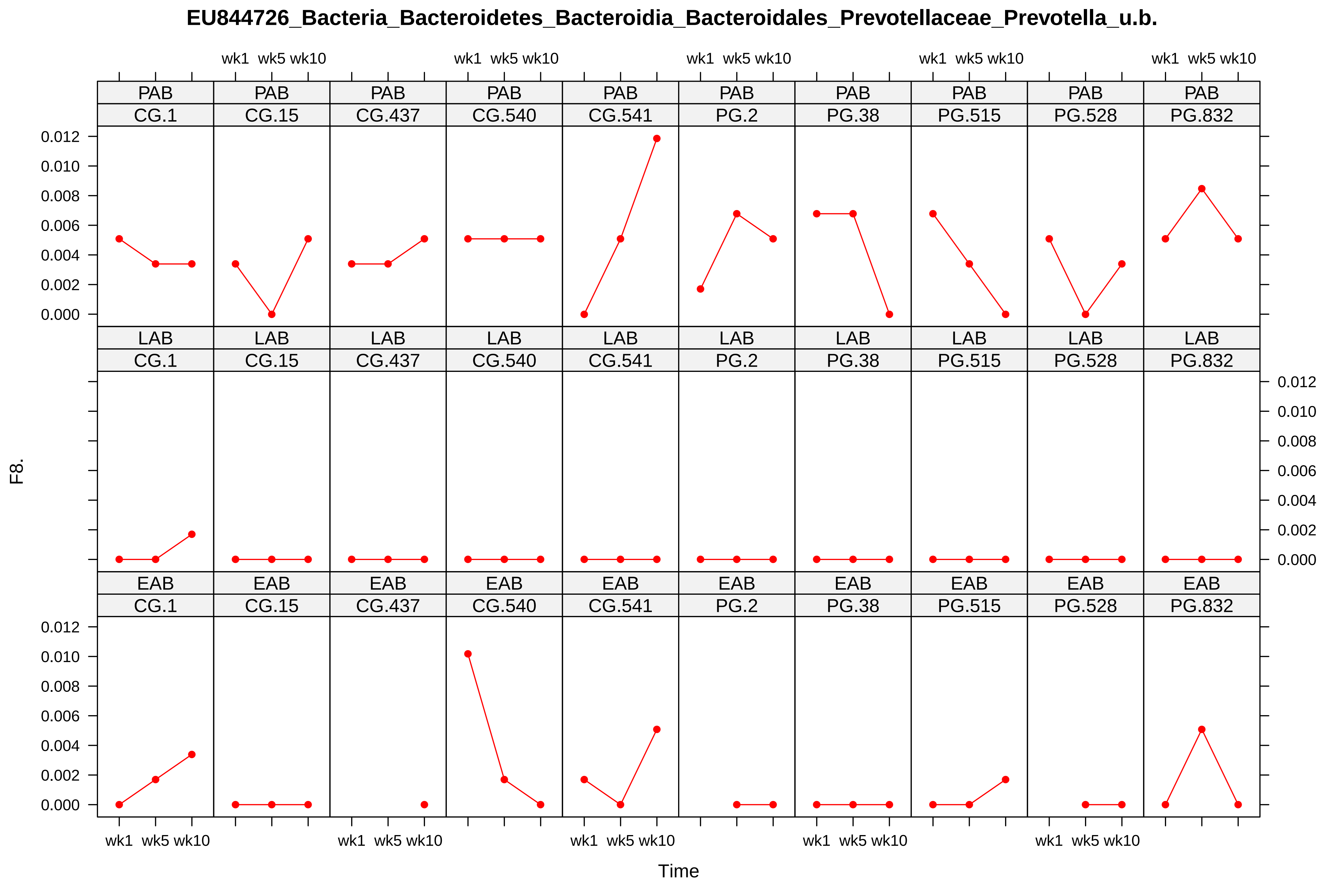
EU844726_Bacteria_Bacteroidetes_Bacteroidia_Bacteroidales_Prevotellaceae_Prevotella_u.b.
PAB
LAB
EAB
CG.1
CG.15
CG.437
CG.540
CG.541
PG.2
PG.38
PG.515
PG.528
PG.832
wk1 wk5 wk10
0.000
0.002
0.004
0.006
0.008
0.010
0.012
F8.
Time

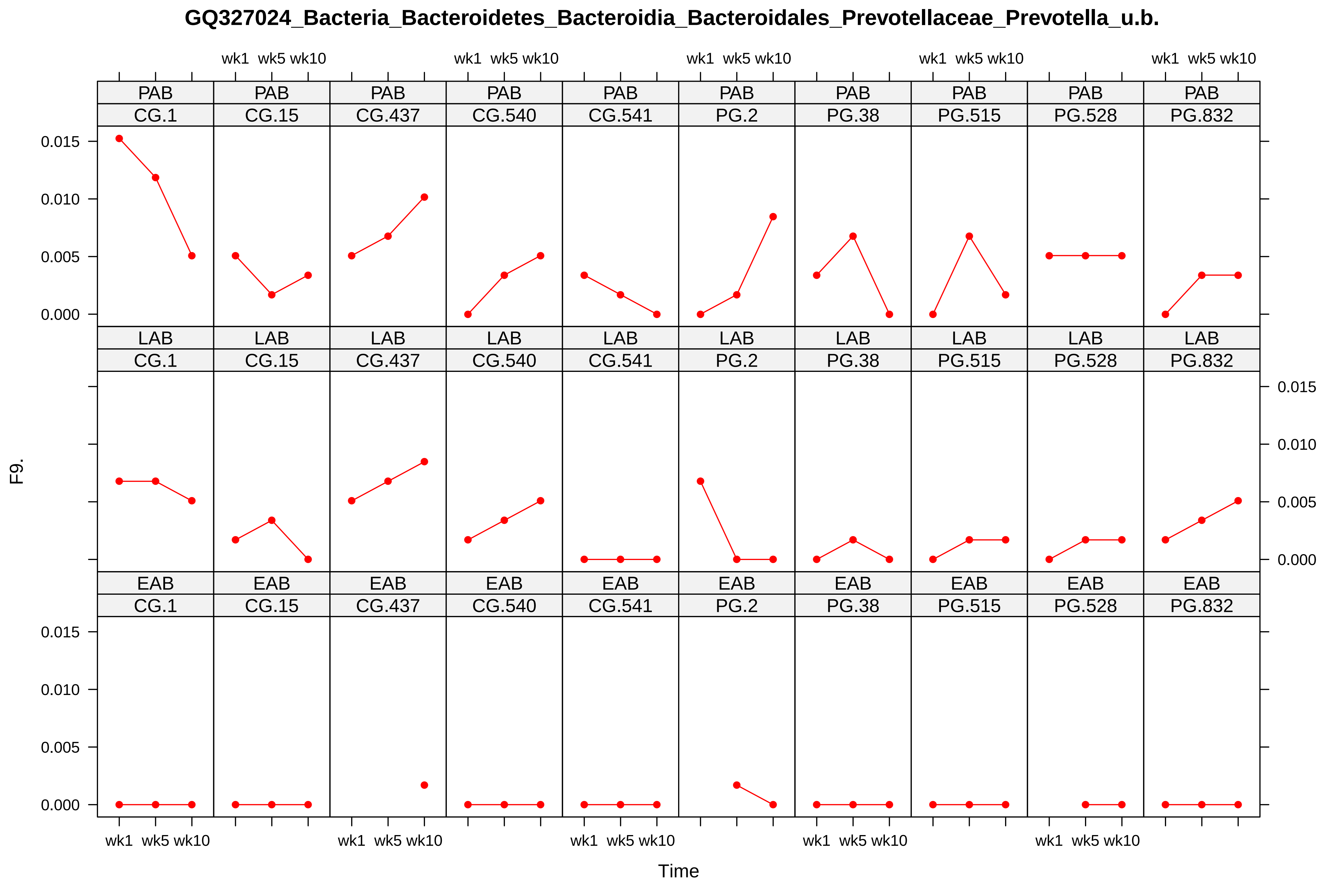

GQ327024_Bacteria_Bacteroidetes_Bacteroidia_Bacteroidales_Prevotellaceae_Prevotella_u.b.
PAB
LAB
EAB
CG.1
CG.15
CG.437
CG.540
CG.541
PG.2
PG.38
PG.515
PG.528
PG.832
0.000
0.005
0.010
0.015
wk1 wk5 wk10
F9.
Time

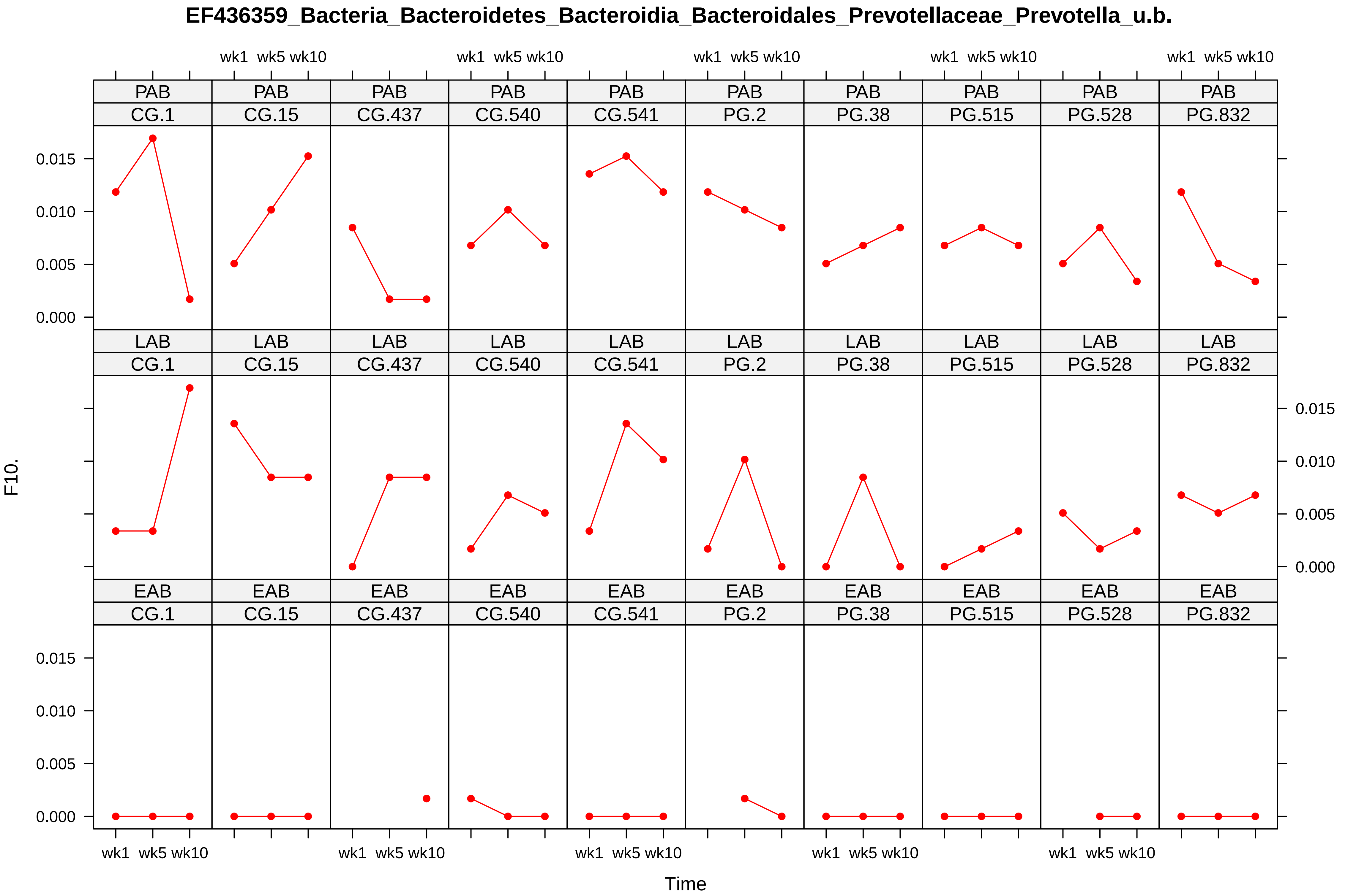

EF436359_Bacteria_Bacteroidetes_Bacteroidia_Bacteroidales_Prevotellaceae_Prevotella_u.b.
PAB
LAB
EAB
CG.1
CG.15
CG.437
CG.540
CG.541
PG.2
PG.38
PG.515
PG.528
PG.832
0.000
0.005
0.010
0.015
wk1
wk5
wk10
F10.
Time

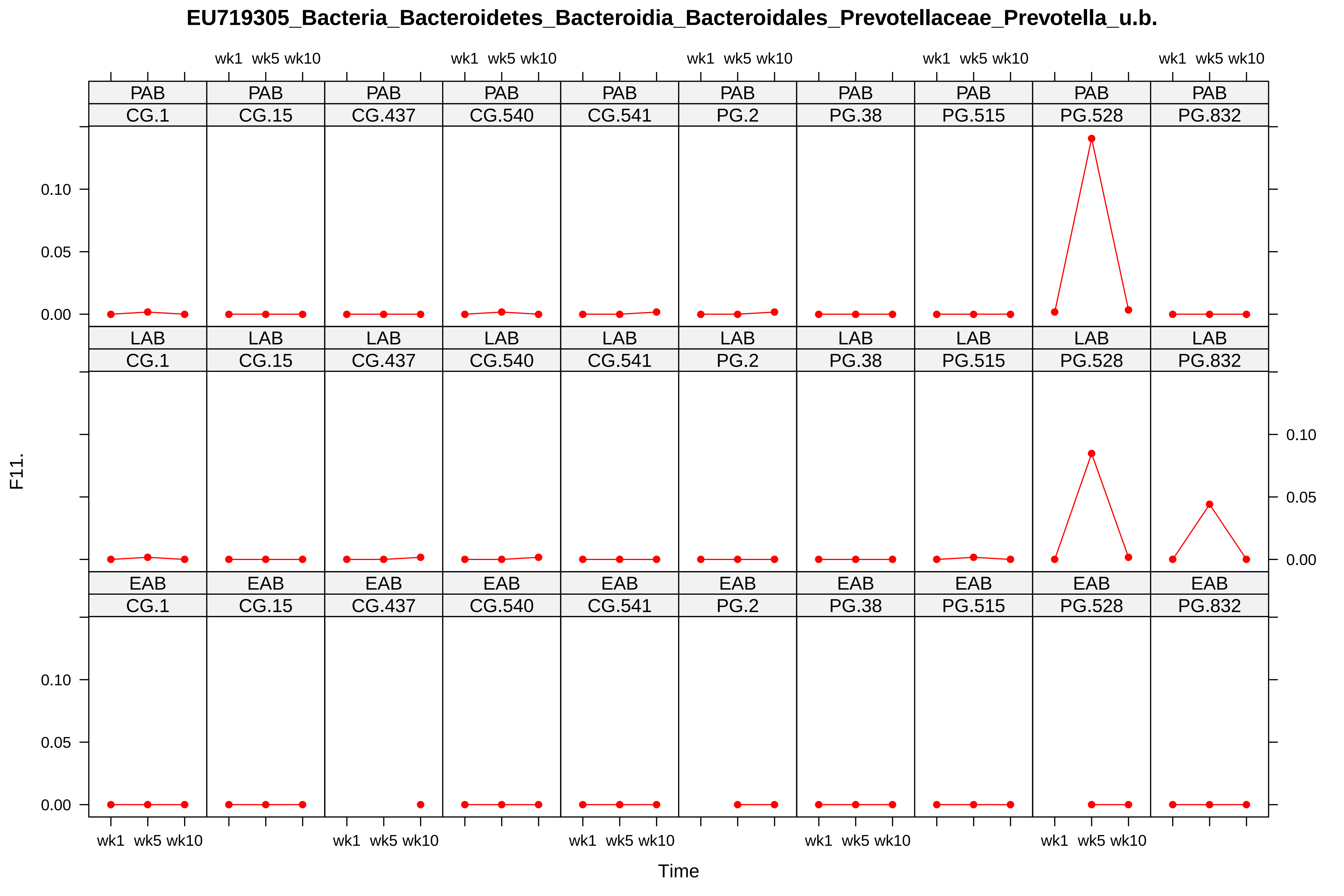

EU719305_Bacteria_Bacteroidetes_Bacteroidia_Bacteroidales_Prevotellaceae_Prevotella_u.b.
PAB
LAB
EAB
CG.1
CG.15
CG.437
CG.540
CG.541
PG.2
PG.38
PG.515
PG.528
PG.832
0.00
0.05
0.10
wk1
wk5
wk10
F11.
Time

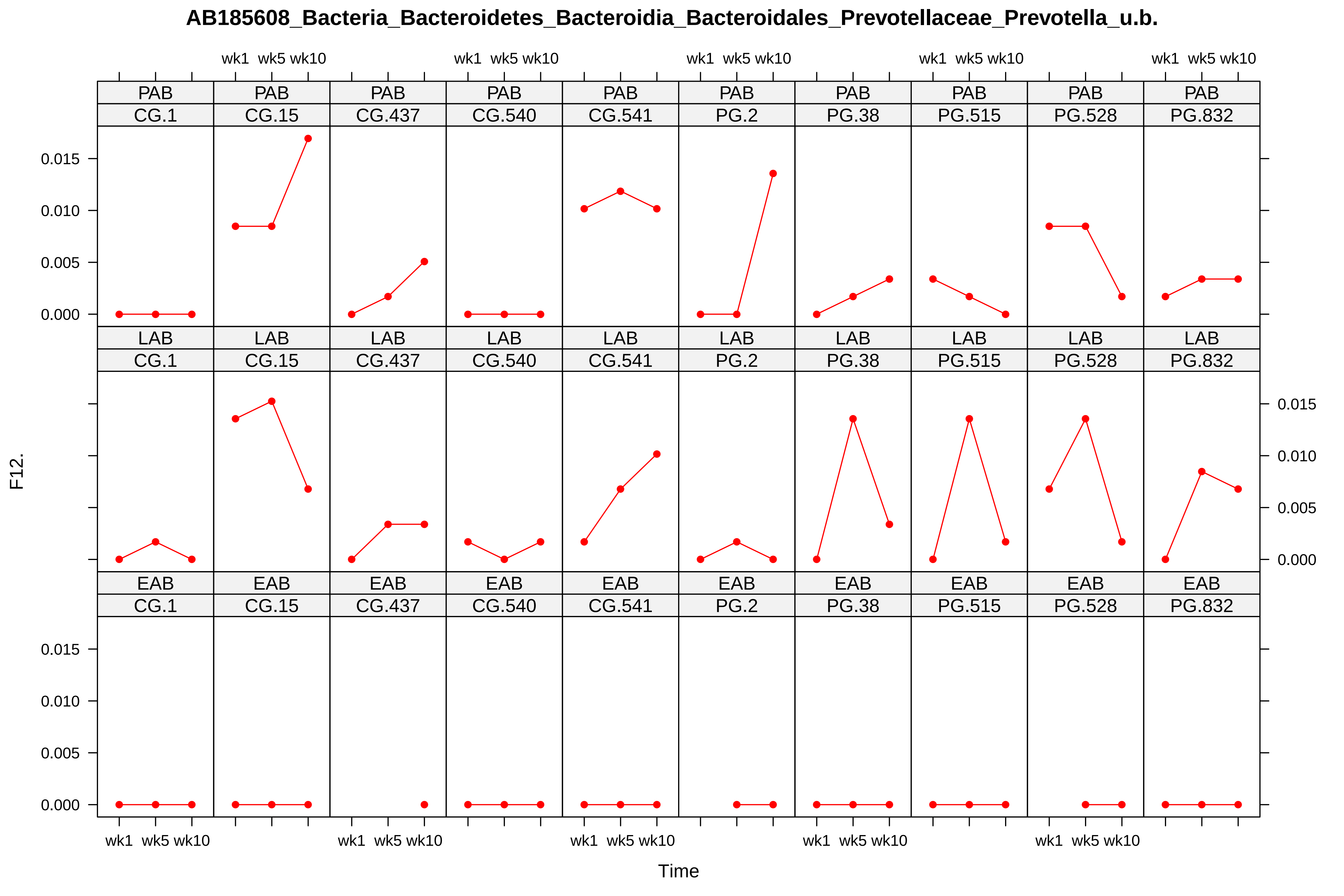
AB185608_Bacteria_Bacteroidetes_Bacteroidia_Bacteroidales_Prevotellaceae_Prevotella_u.b.
PAB
LAB
EAB
CG.1
CG.15
CG.437
CG.540
CG.541
PG.2
PG.38
PG.515
PG.528
PG.832
0.000
0.005
0.010
0.015
wk1
wk5
wk10
F12.
Time

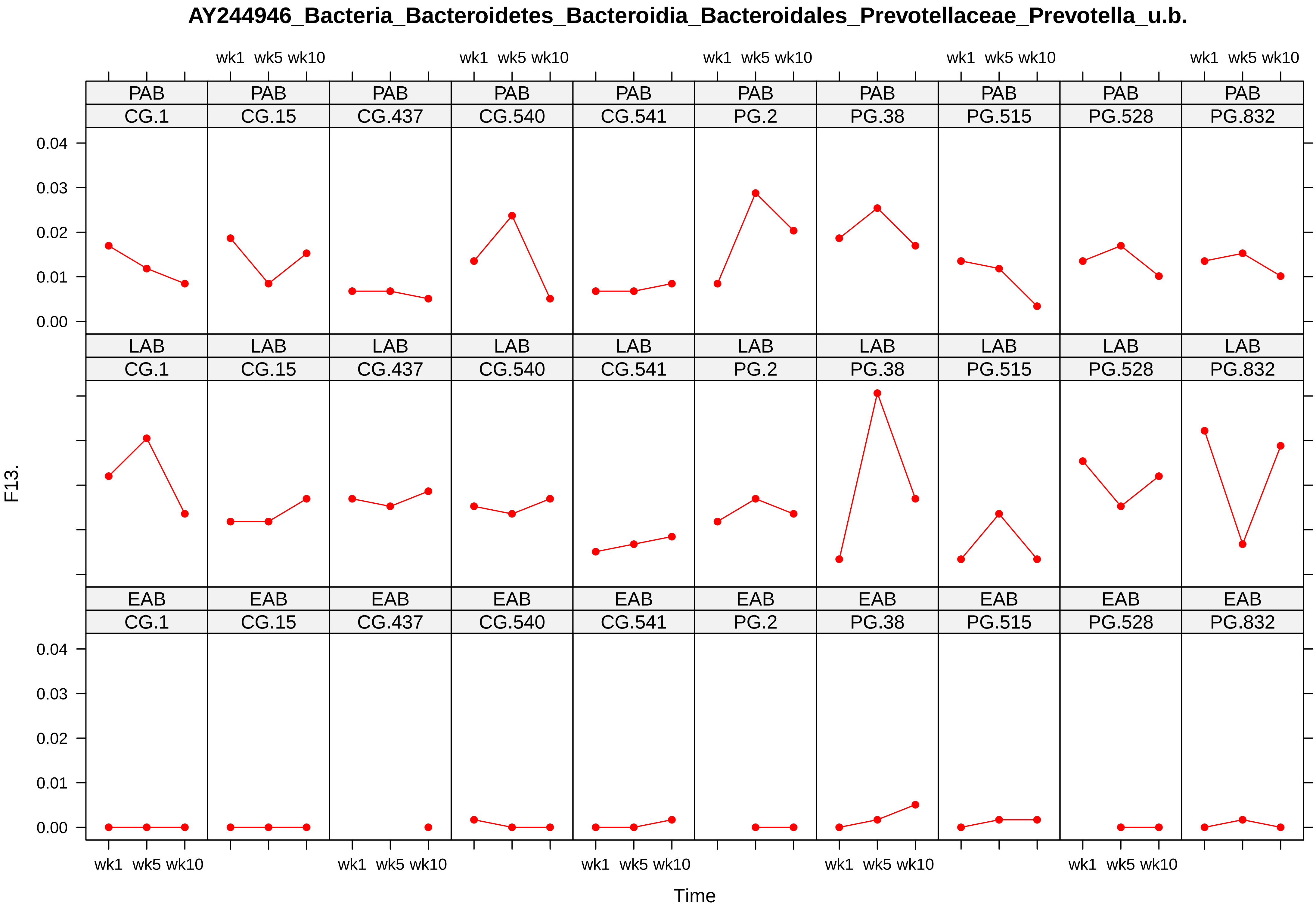
AY244946_Bacteria_Bacteroidetes_Bacteroidia_Bacteroidales_Prevotellaceae_Prevotella_u.b.
PAB
LAB
EAB
CG.1
CG.15
CG.437
CG.540
CG.541
PG.2
PG.38
PG.515
PG.528
PG.832
wk1 wk5 wk10
0.00
0.01
0.02
0.03
0.04
F13.
Time

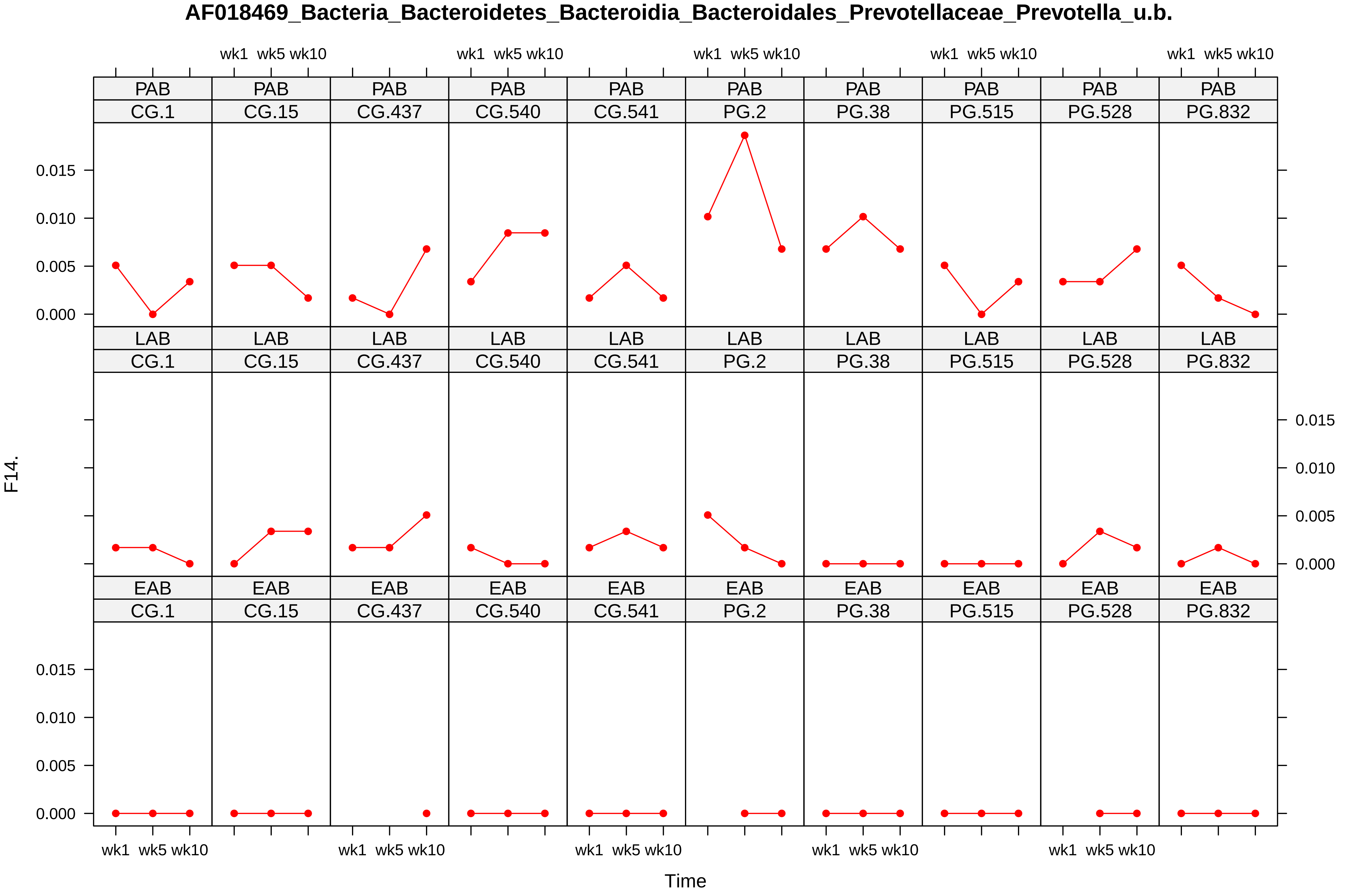
AF018469_Bacteria_Bacteroidetes_Bacteroidia_Bacteroidales_Prevotellaceae_Prevotella_u.b.
PAB
LAB
EAB
CG.1
CG.15
CG.437
CG.540
CG.541
PG.2
PG.38
PG.515
PG.528
PG.832
0.000
0.005
0.010
0.015
wk1 wk5 wk10
F14.
Time

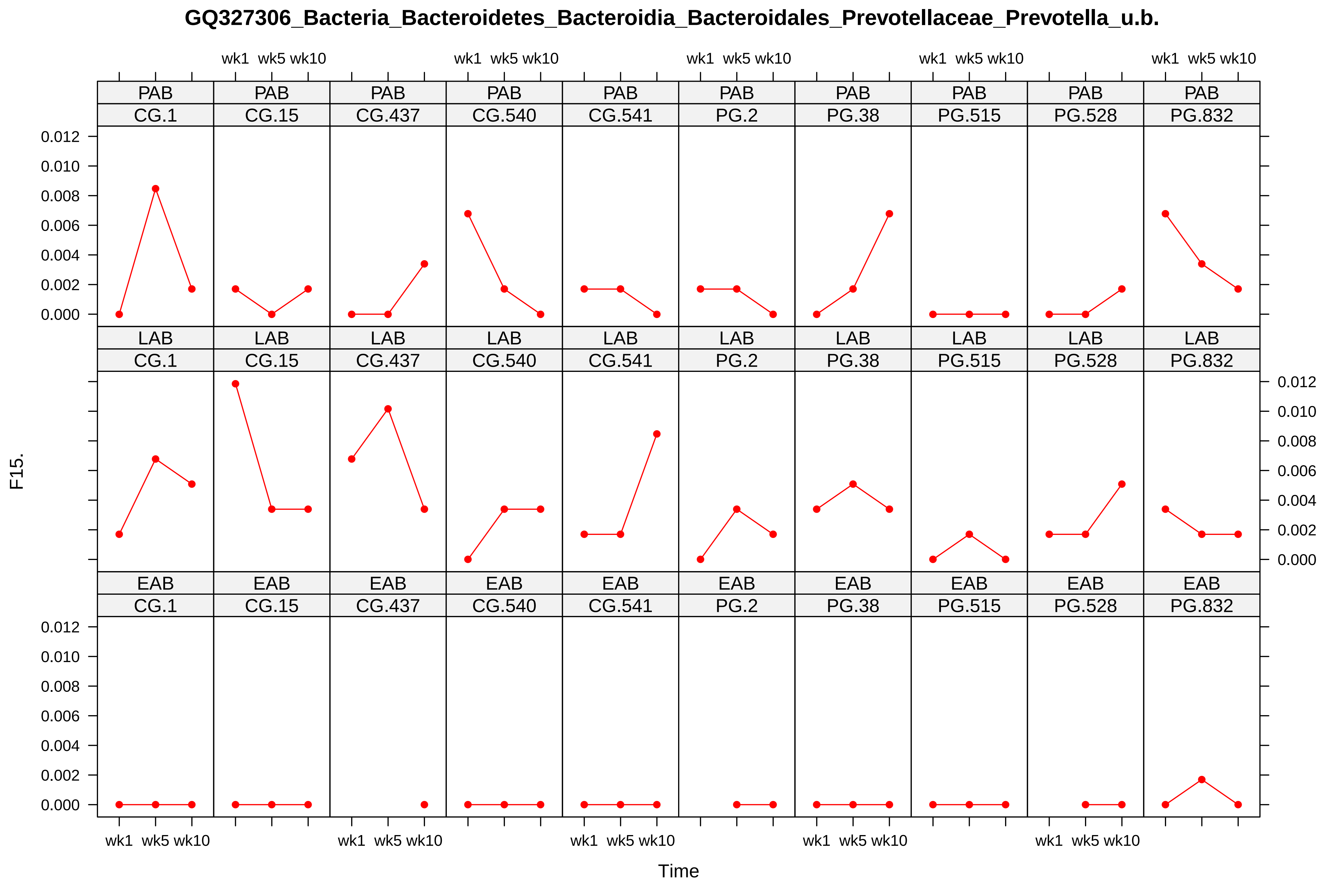

GQ327306_Bacteria_Bacteroidetes_Bacteroidia_Bacteroidales_Prevotellaceae_Prevotella_u.b.
PAB
LAB
EAB
CG.1
CG.15
CG.437
CG.540
CG.541
PG.2
PG.38
PG.515
PG.528
PG.832
wk1 wk5 wk10
0.000
0.002
0.004
0.006
0.008
0.010
0.012
F15.
Time

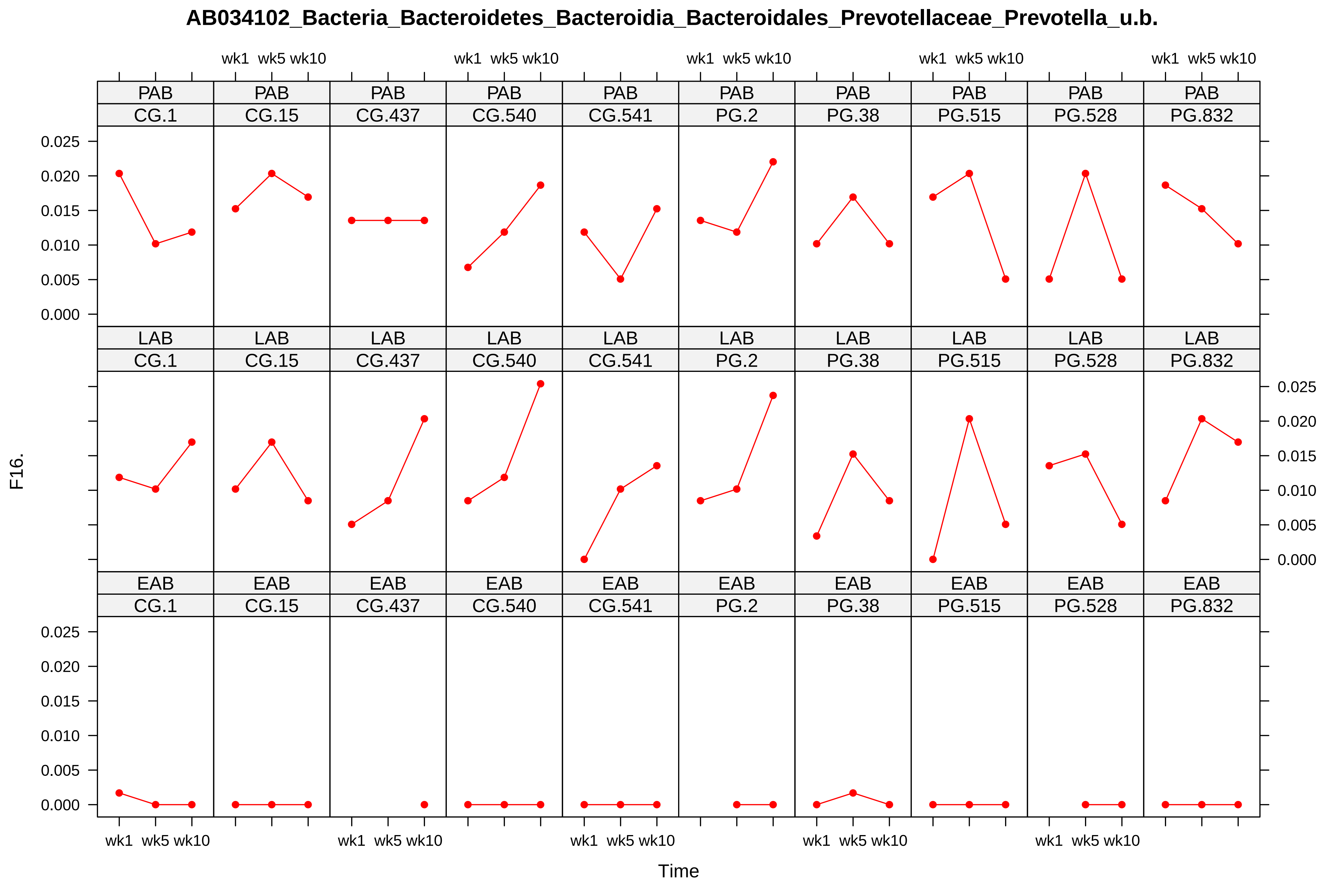
AB034102_Bacteria_Bacteroidetes_Bacteroidia_Bacteroidales_Prevotellaceae_Prevotella_u.b.
PAB
LAB
EAB
CG.1
CG.15
CG.437
CG.540
CG.541
PG.2
PG.38
PG.515
PG.528
PG.832
F16.
Time
wk1
wk5
wk10
0.000
0.005
0.010
0.015
0.020
0.025

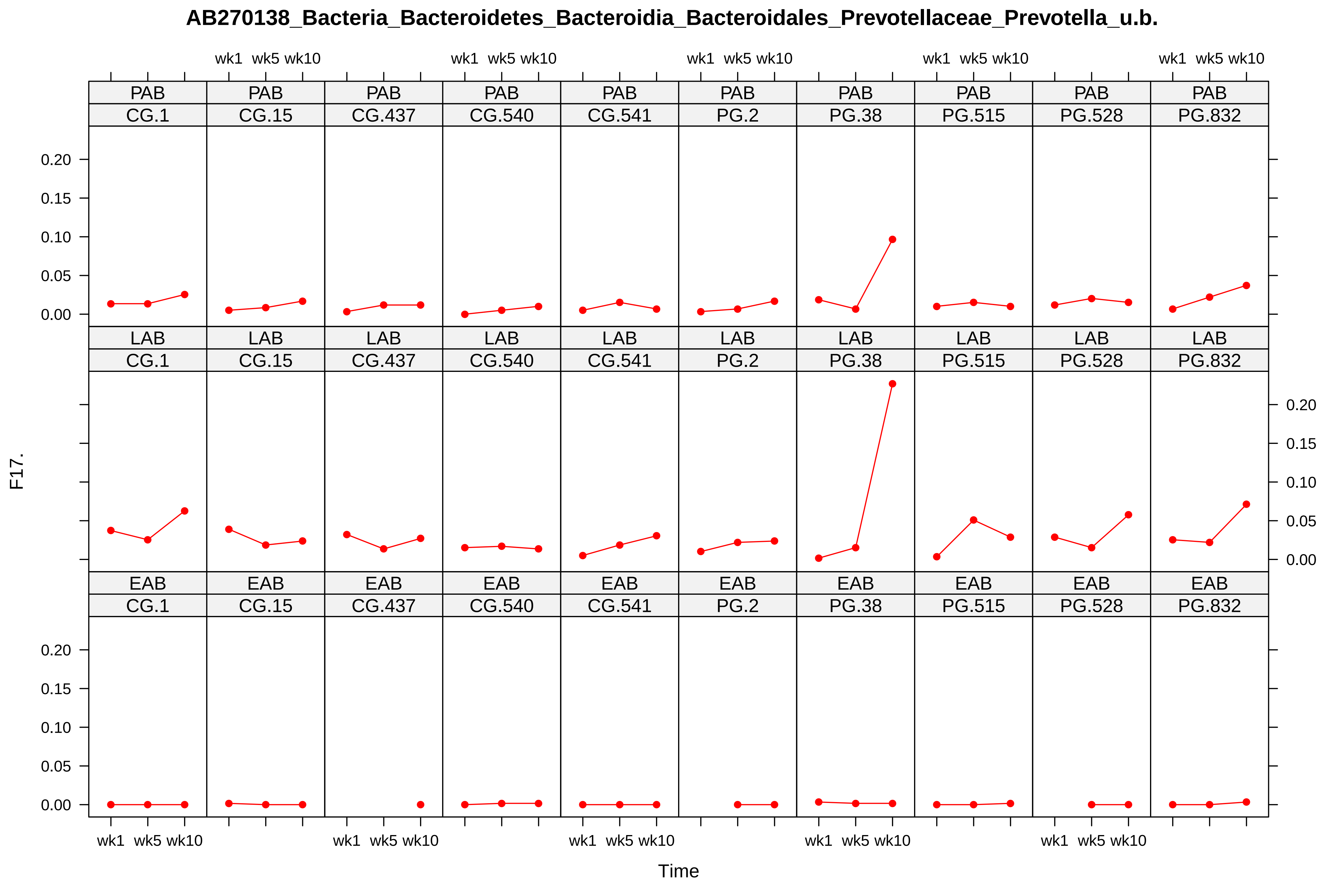
AB270138_Bacteria_Bacteroidetes_Bacteroidia_Bacteroidales_Prevotellaceae_Prevotella_u.b.
PAB
LAB
EAB
CG.1
CG.15
CG.437
CG.540
CG.541
PG.2
PG.38
PG.515
PG.528
PG.832
wk1 wk5 wk10
0.00
0.05
0.10
0.15
0.20
F17.
Time

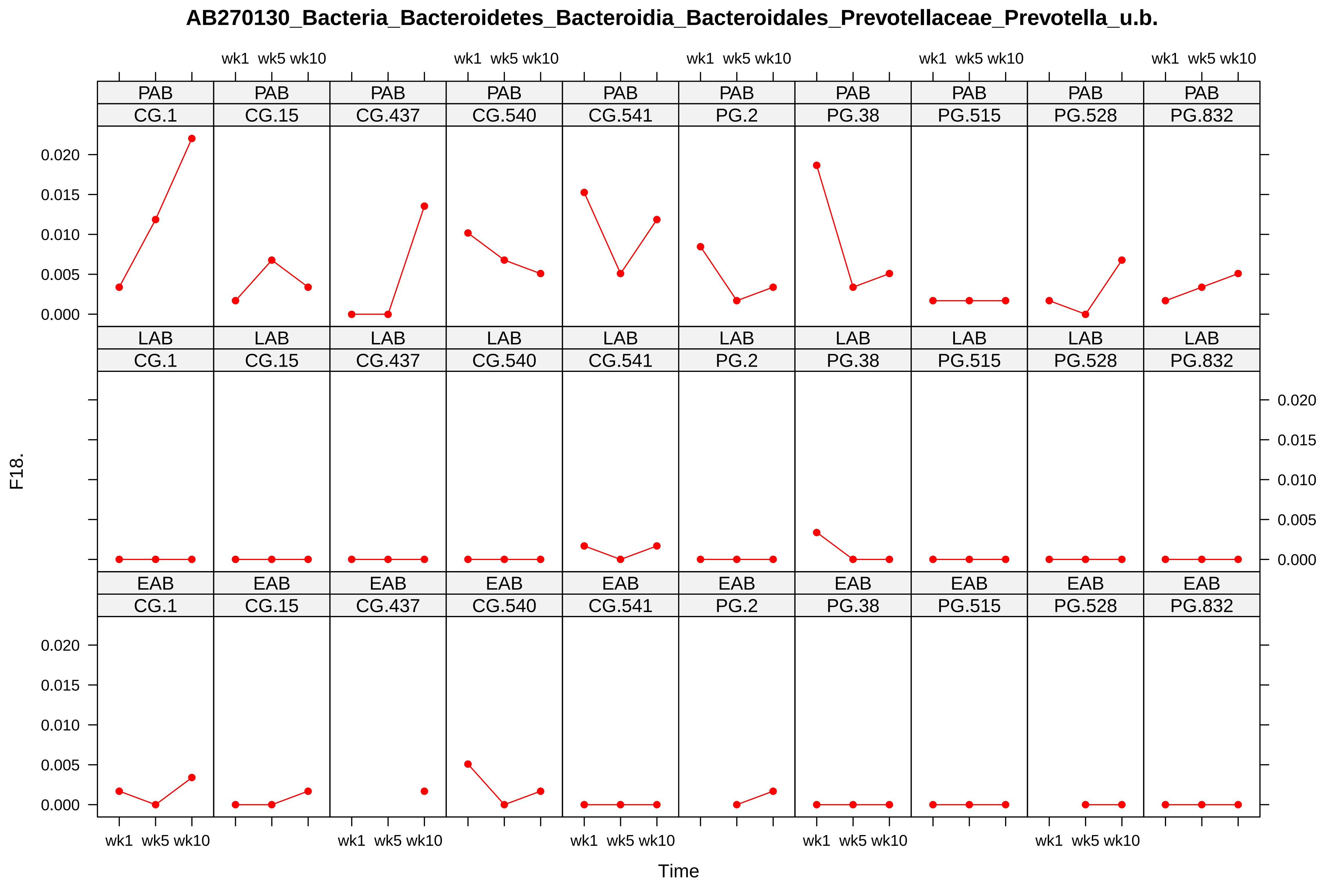

AB270130_Bacteria_Bacteroidetes_Bacteroidia_Bacteroidales_Prevotellaceae_Prevotella_u.b.
PAB
LAB
EAB
CG.1
CG.15
CG.437
CG.540
CG.541
PG.2
PG.38
PG.515
PG.528
PG.832
wk1
wk5
wk10
0.000
0.005
0.010
0.015
0.020
F18.
Time

GQ327214_Bacteria_Bacteroidetes_Bacteroidia_Bacteroidales_Prevotellaceae_Prevotella_u.b.

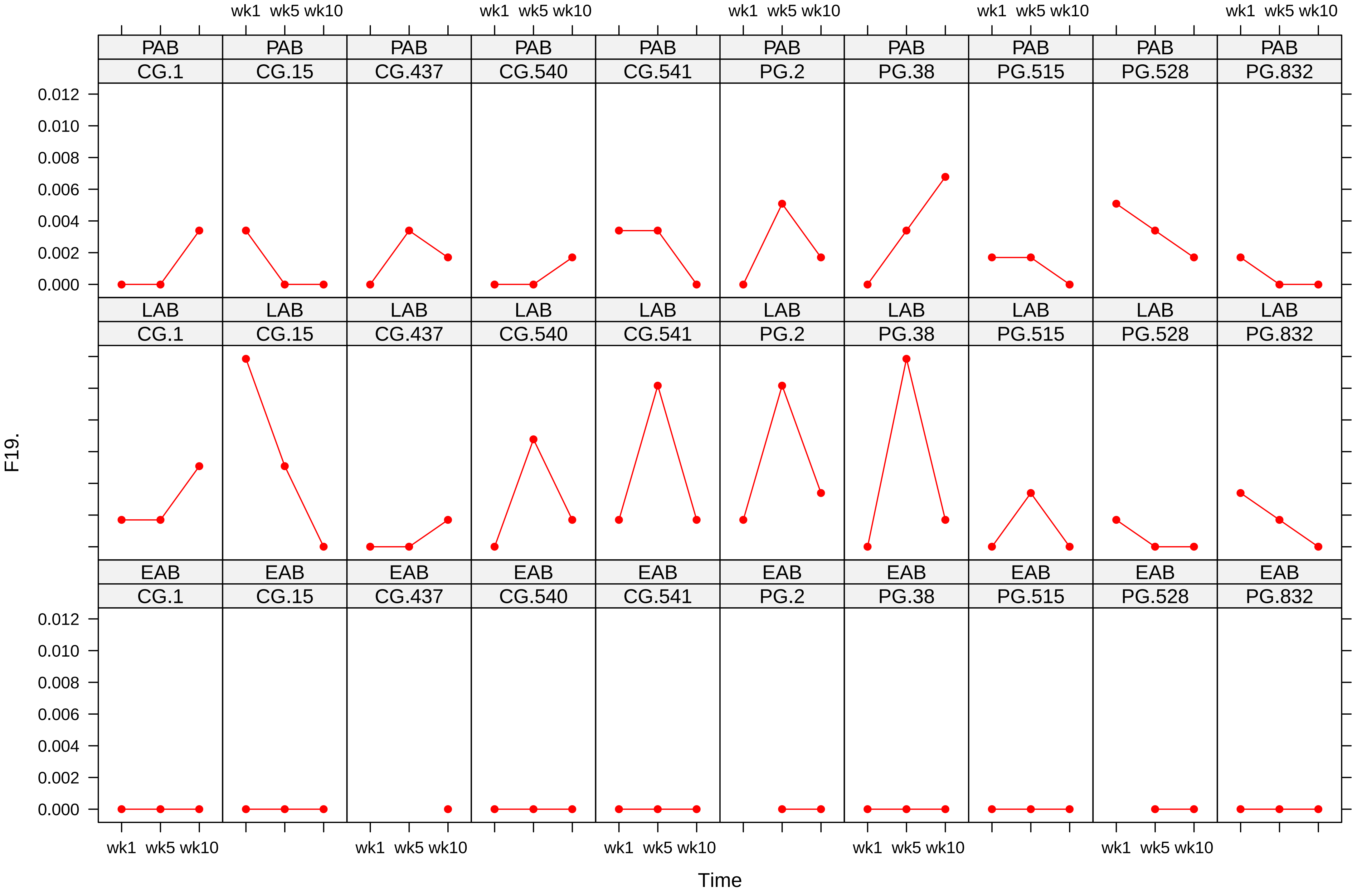

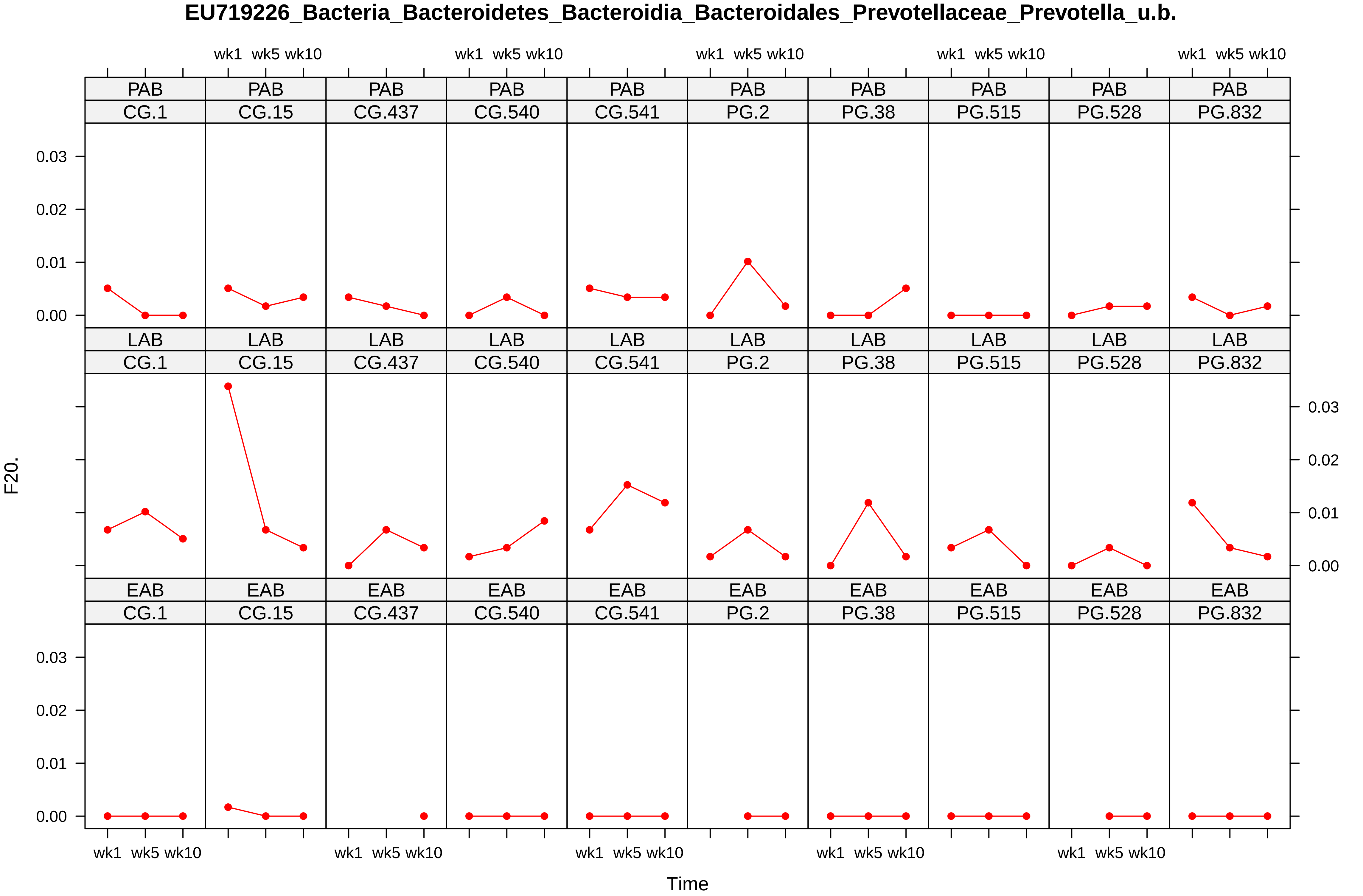

EU719226_Bacteria_Bacteroidetes_Bacteroidia_Bacteroidales_Prevotellaceae_Prevotella_u.b.
PAB
LAB
EAB
CG.1
CG.15
CG.437
CG.540
CG.541
PG.2
PG.38
PG.515
PG.528
PG.832
wk1
wk5
wk10
0.00
0.01
0.02
0.03
F20.
Time

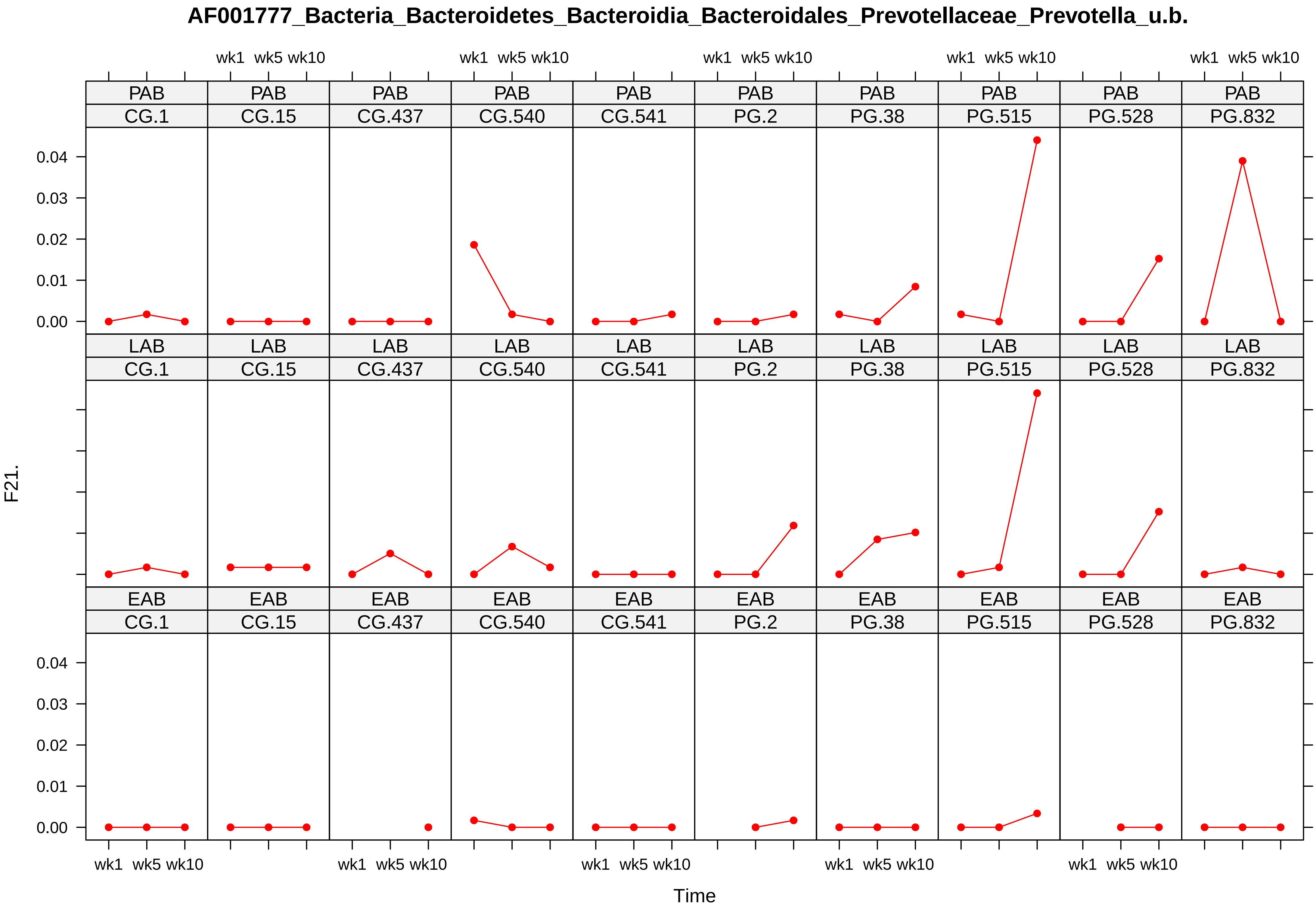
AF001777_Bacteria_Bacteroidetes_Bacteroidia_Bacteroidales_Prevotellaceae_Prevotella_u.b.
PAB
LAB
EAB
CG.1
CG.15
CG.437
CG.540
CG.541
PG.2
PG.38
PG.515
PG.528
PG.832
0.00
0.01
0.02
0.03
0.04
wk1 wk5 wk10
F21.
Time

AB269968_Bacteria_Bacteroidetes_Bacteroidia_Bacteroidales_Prevotellaceae_Prevotella_u.b.

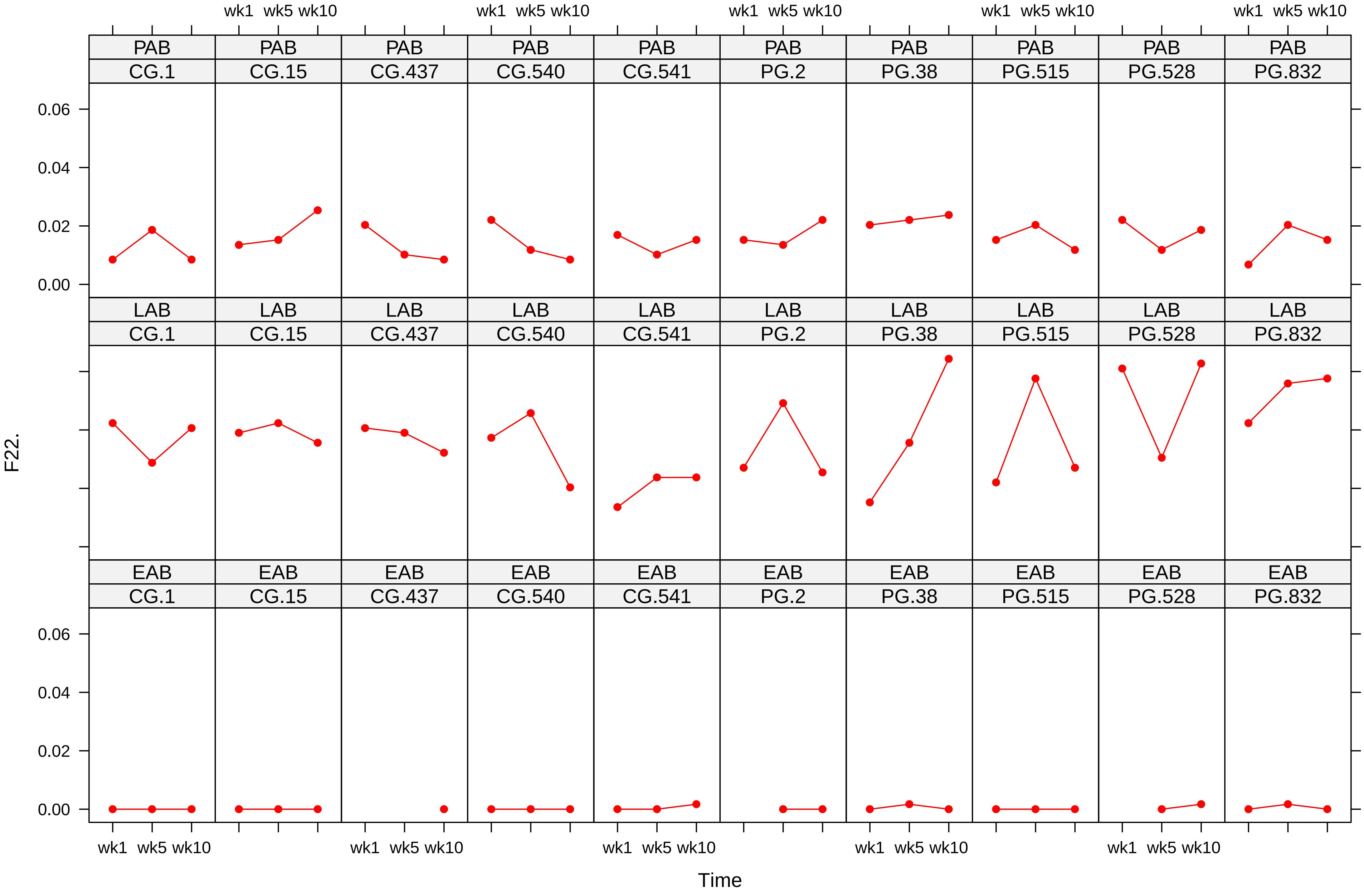

GU302536_Bacteria_Bacteroidetes_Bacteroidia_Bacteroidales_Prevotellaceae_Prevotella_u.b.

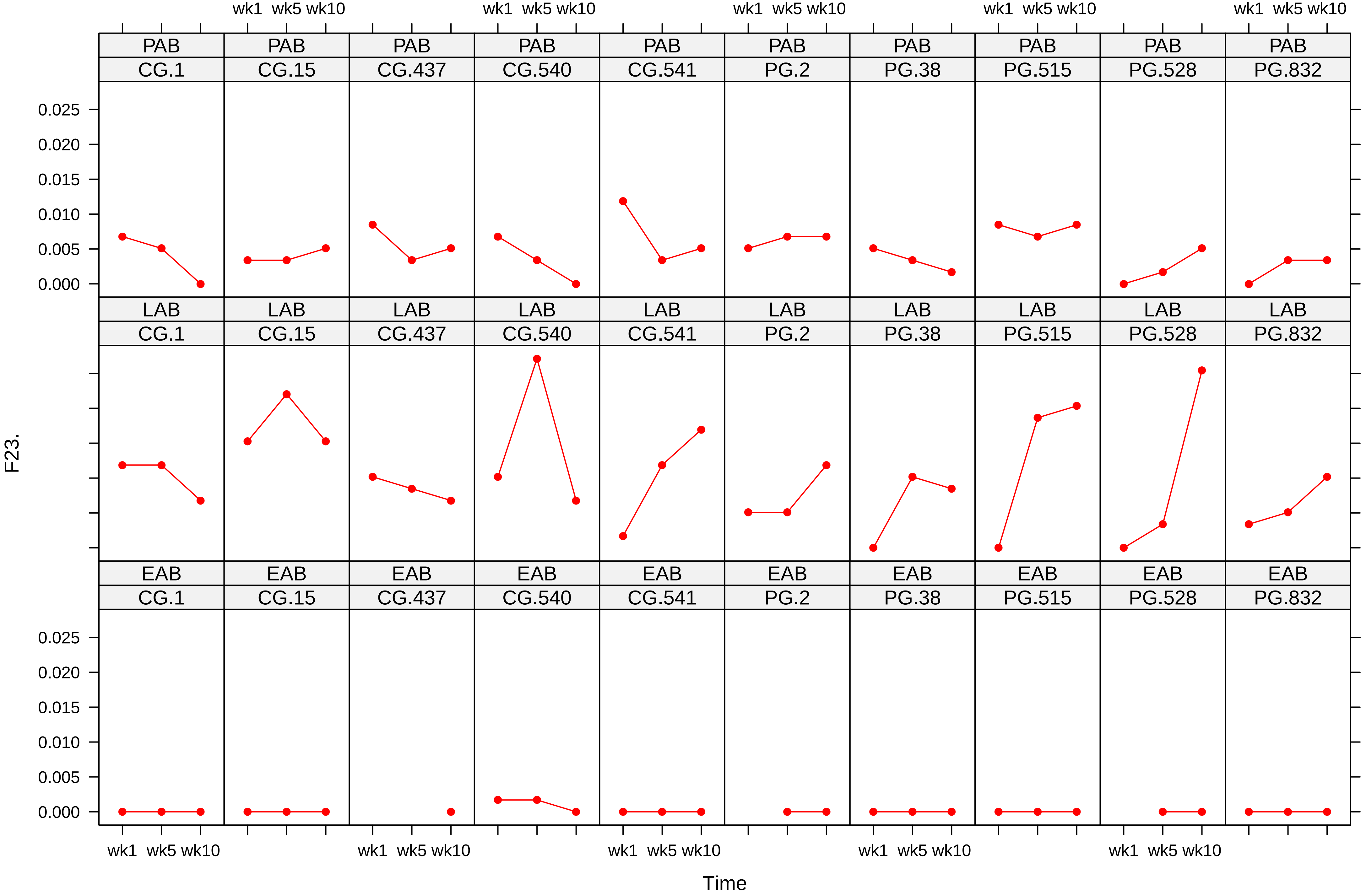

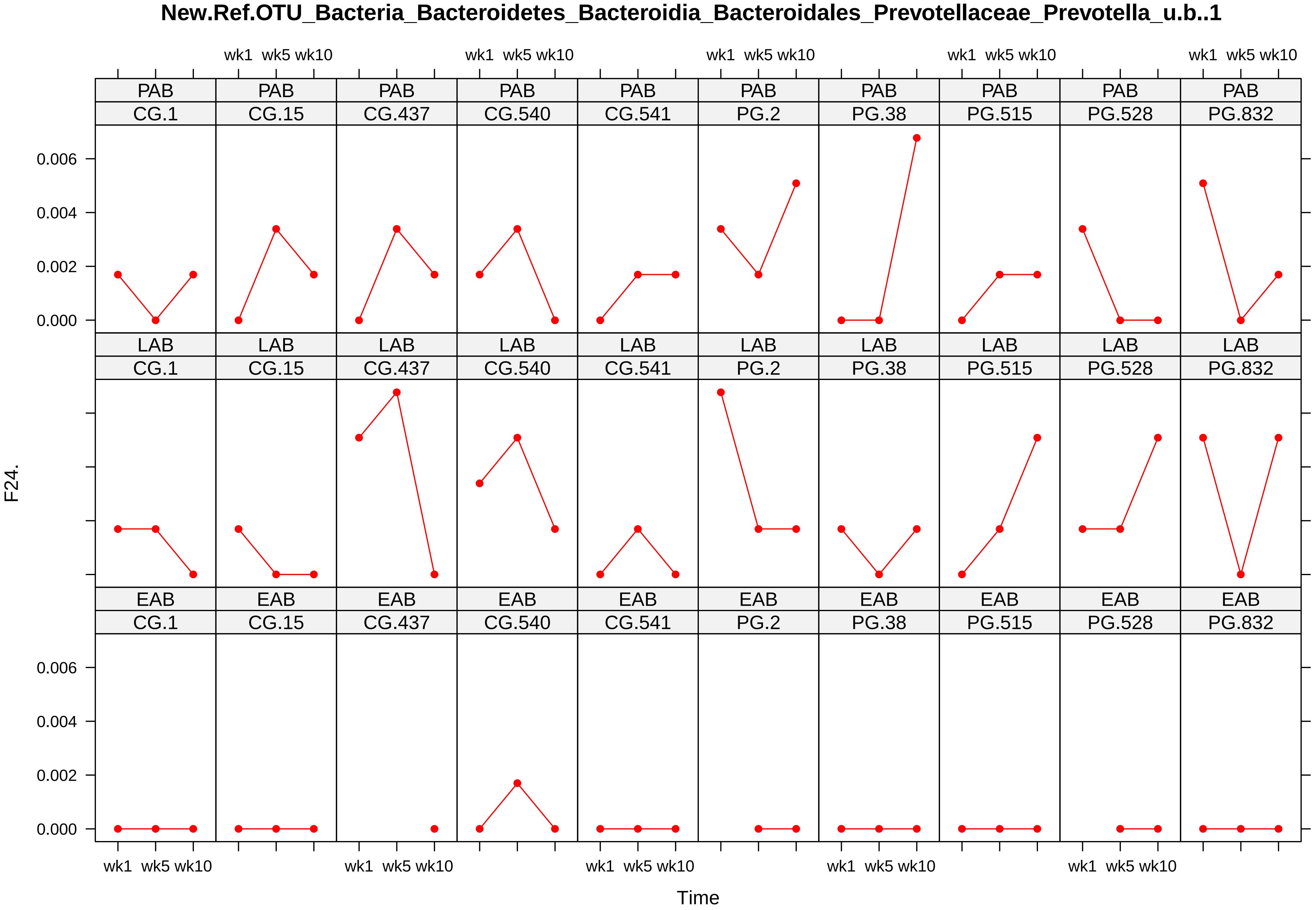
New.Ref.OTU_Bacteria_Bacteroidetes_Bacteroidia_Bacteroidales_Prevotellaceae_Prevotella_u.b..1
PAB
LAB
EAB
CG.1
CG.15
CG.437
CG.540
CG.541
PG.2
PG.38
PG.515
PG.528
PG.832
0.000
0.002
0.004
0.006
wk1 wk5 wk10
F24.
Time

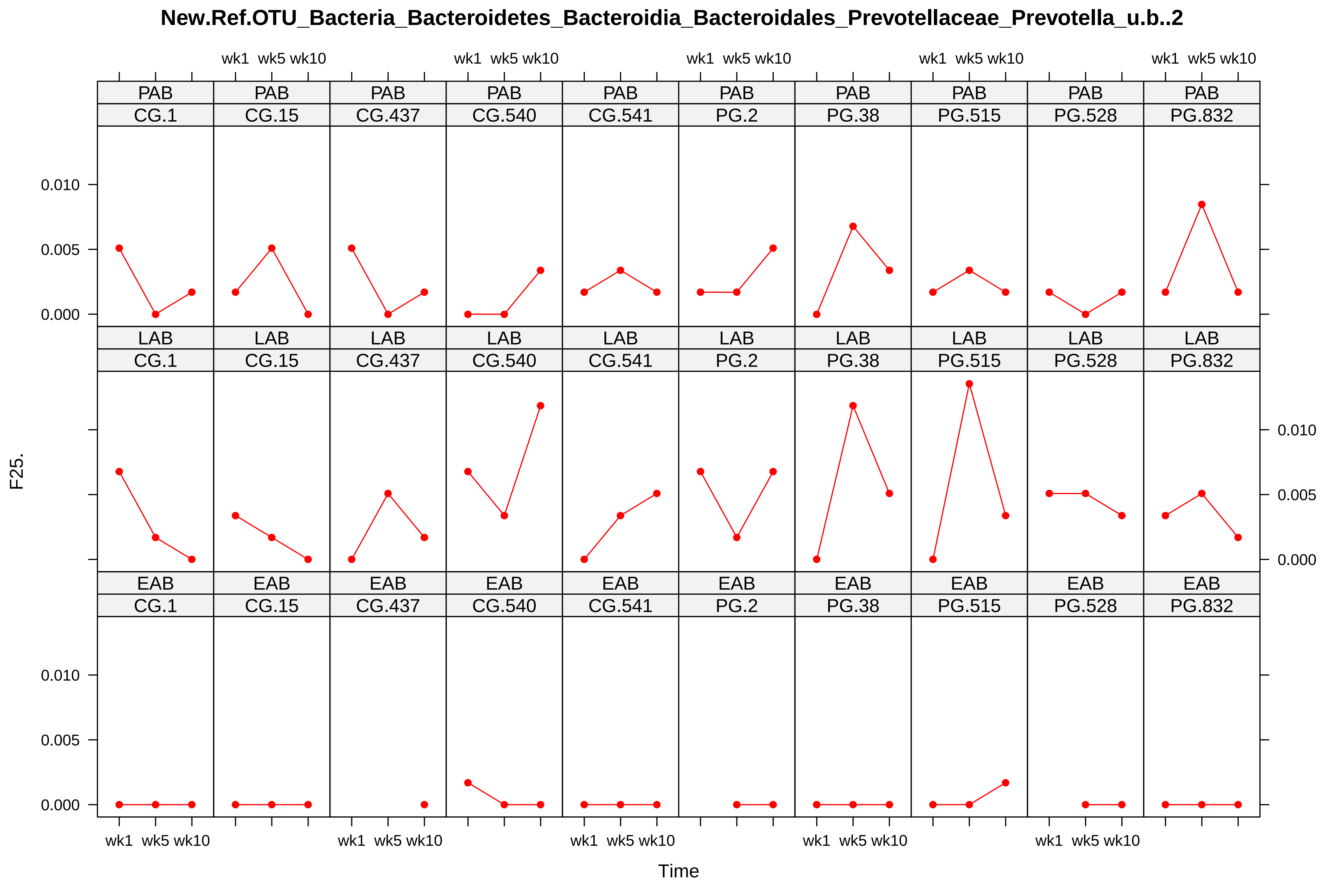
New.Ref.OTU_Bacteria_Bacteroidetes_Bacteroidia_Bacteroidales_Prevotellaceae_Prevotella_u.b..2
PAB
LAB
EAB
CG.1
CG.15
CG.437
CG.540
CG.541
PG.2
PG.38
PG.515
PG.528
PG.832
0.000
0.005
0.010
wk1 wk5 wk10
F25.
Time

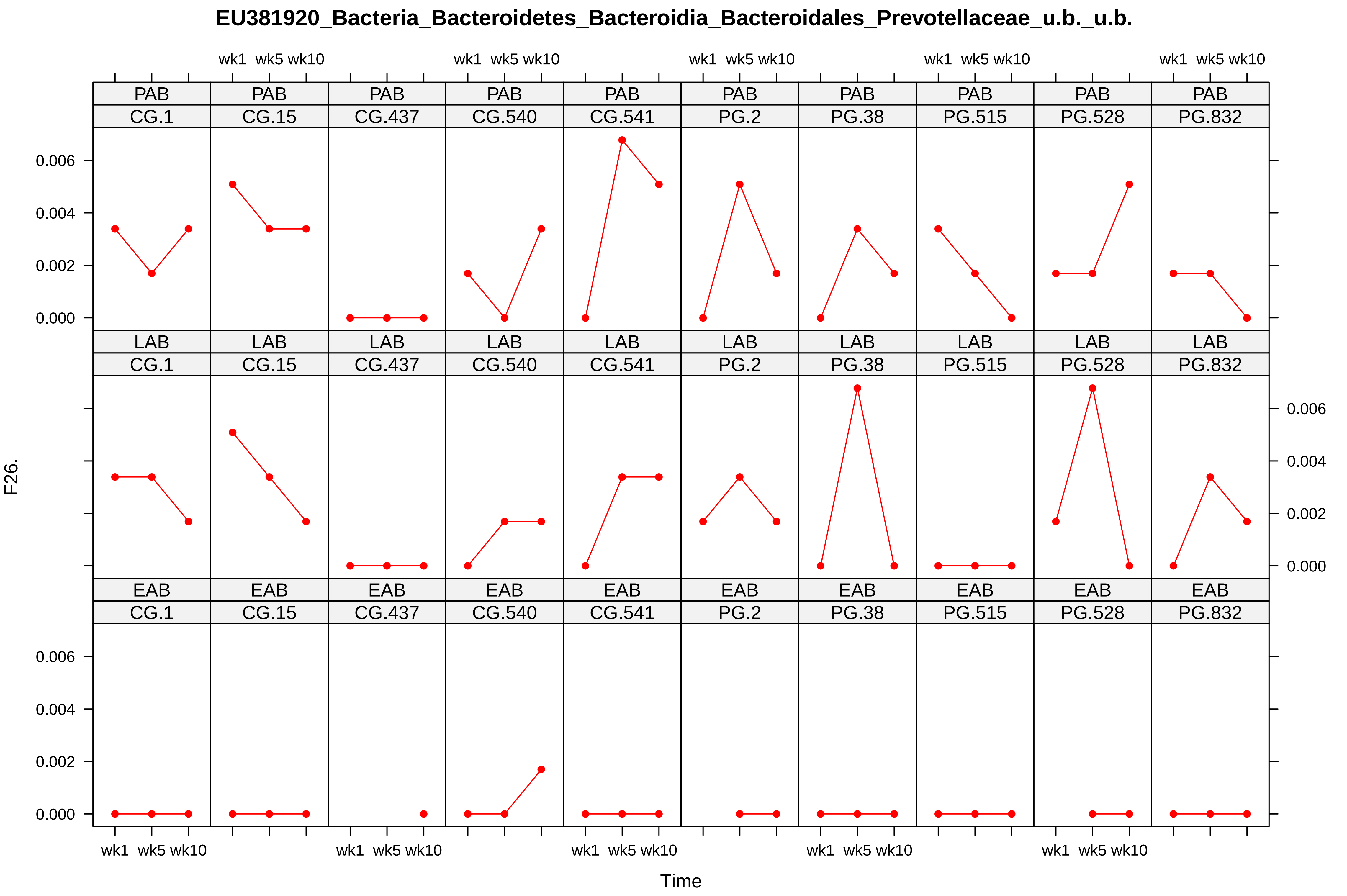
EU381920_Bacteria_Bacteroidetes_Bacteroidia_Bacteroidales_Prevotellaceae_u.b._u.b.
PAB
LAB
EAB
CG.1
CG.15
CG.437
CG.540
CG.541
PG.2
PG.38
PG.515
PG.528
PG.832
0.000
0.002
0.004
0.006
wk1 wk5 wk10
F26.
Time

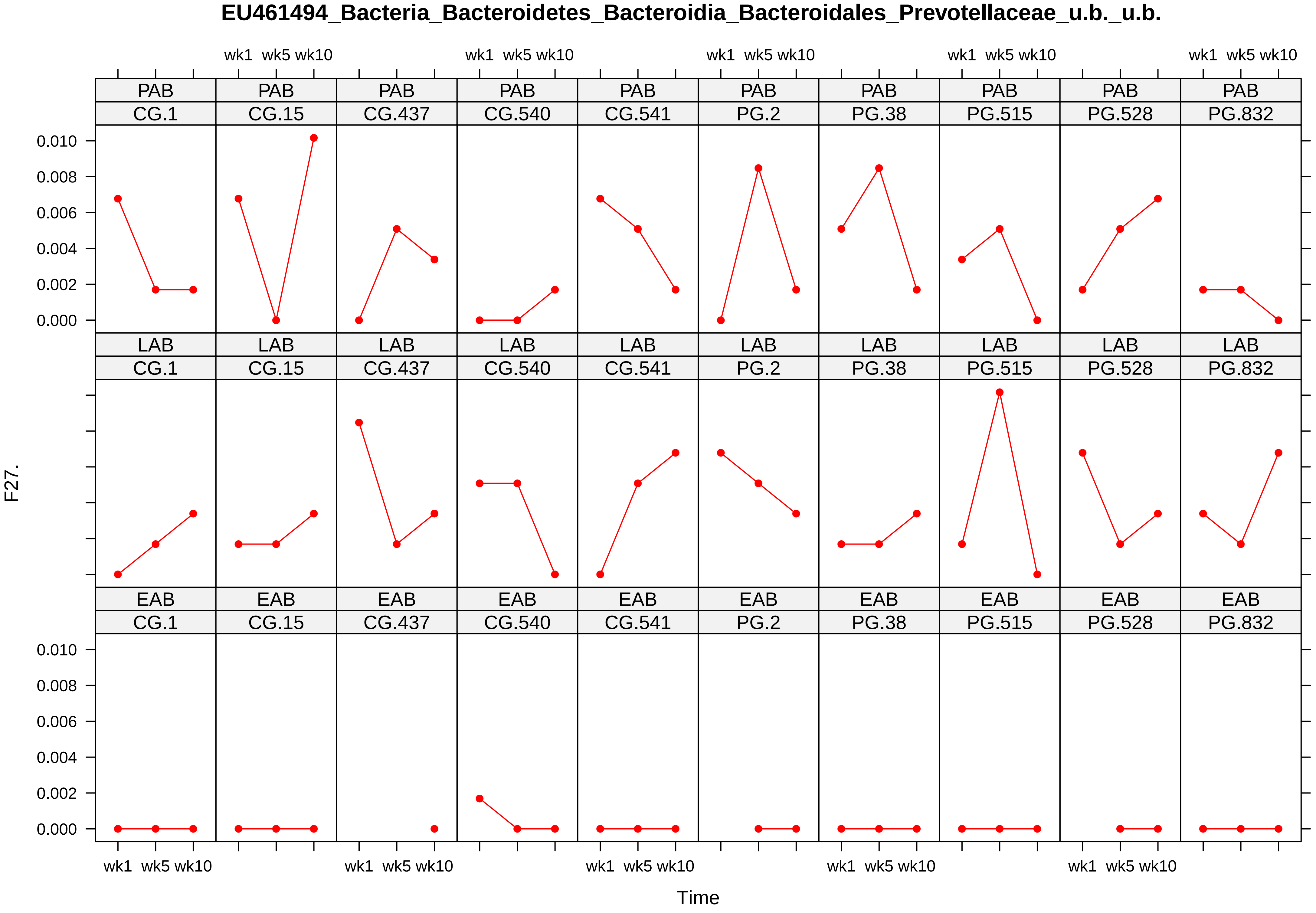
EU461494_Bacteria_Bacteroidetes_Bacteroidia_Bacteroidales_Prevotellaceae_u.b._u.b.
PAB
LAB
EAB
CG.1
CG.15
CG.437
CG.540
CG.541
PG.2
PG.38
PG.515
PG.528
PG.832
0.000
0.002
0.004
0.006
0.008
0.010
wk1 wk5 wk10
F27.
Time

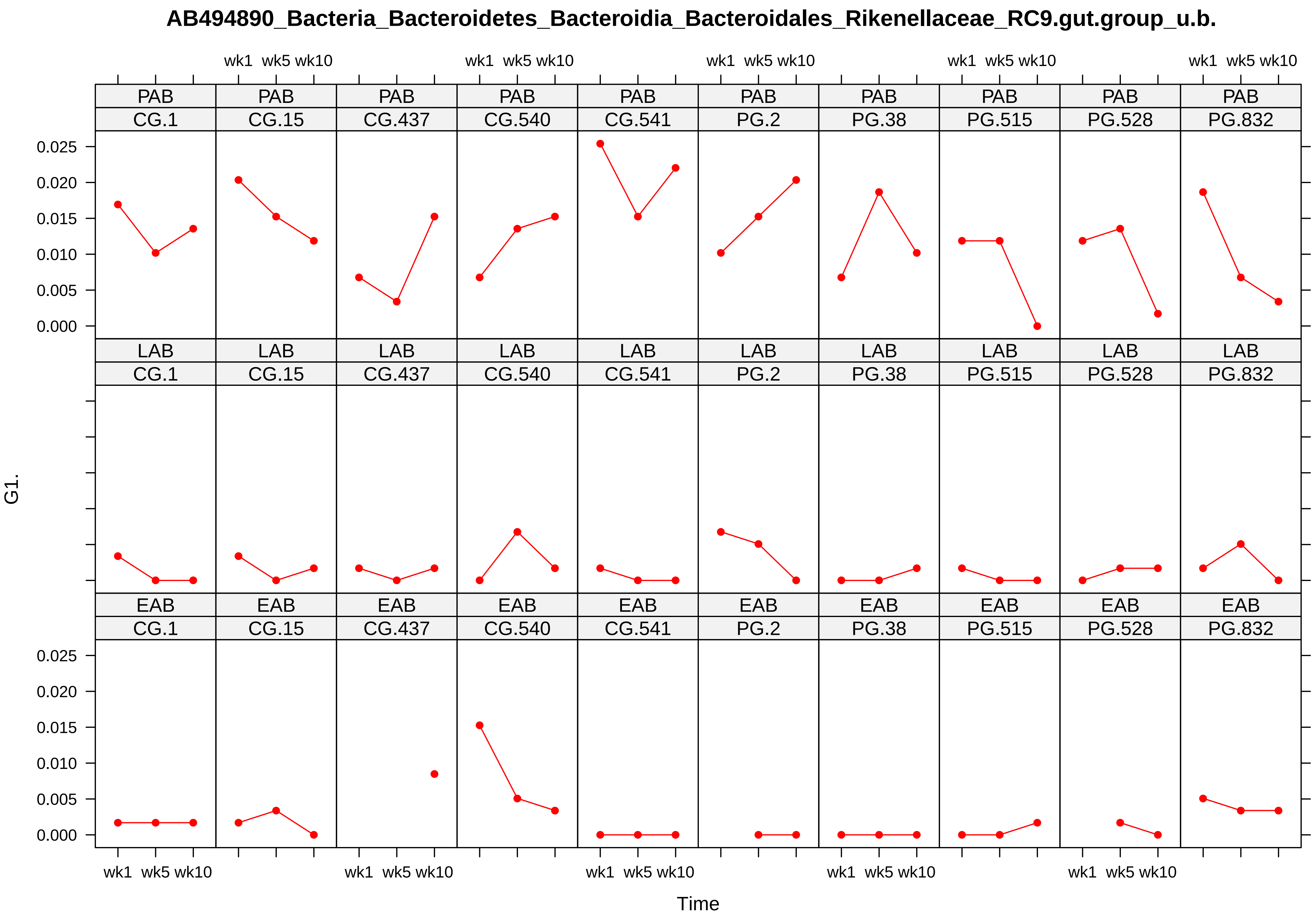
AB494890_Bacteria_Bacteroidetes_Bacteroidia_Bacteroidales_Rikenellaceae_RC9.gut.group_u.b.
PAB
LAB
EAB
CG.1
CG.15
CG.437
CG.540
CG.541
PG.2
PG.38
PG.515
PG.528
PG.832
wk1 wk5 wk10
0.000
0.005
0.010
0.015
0.020
0.025
G1.
Time

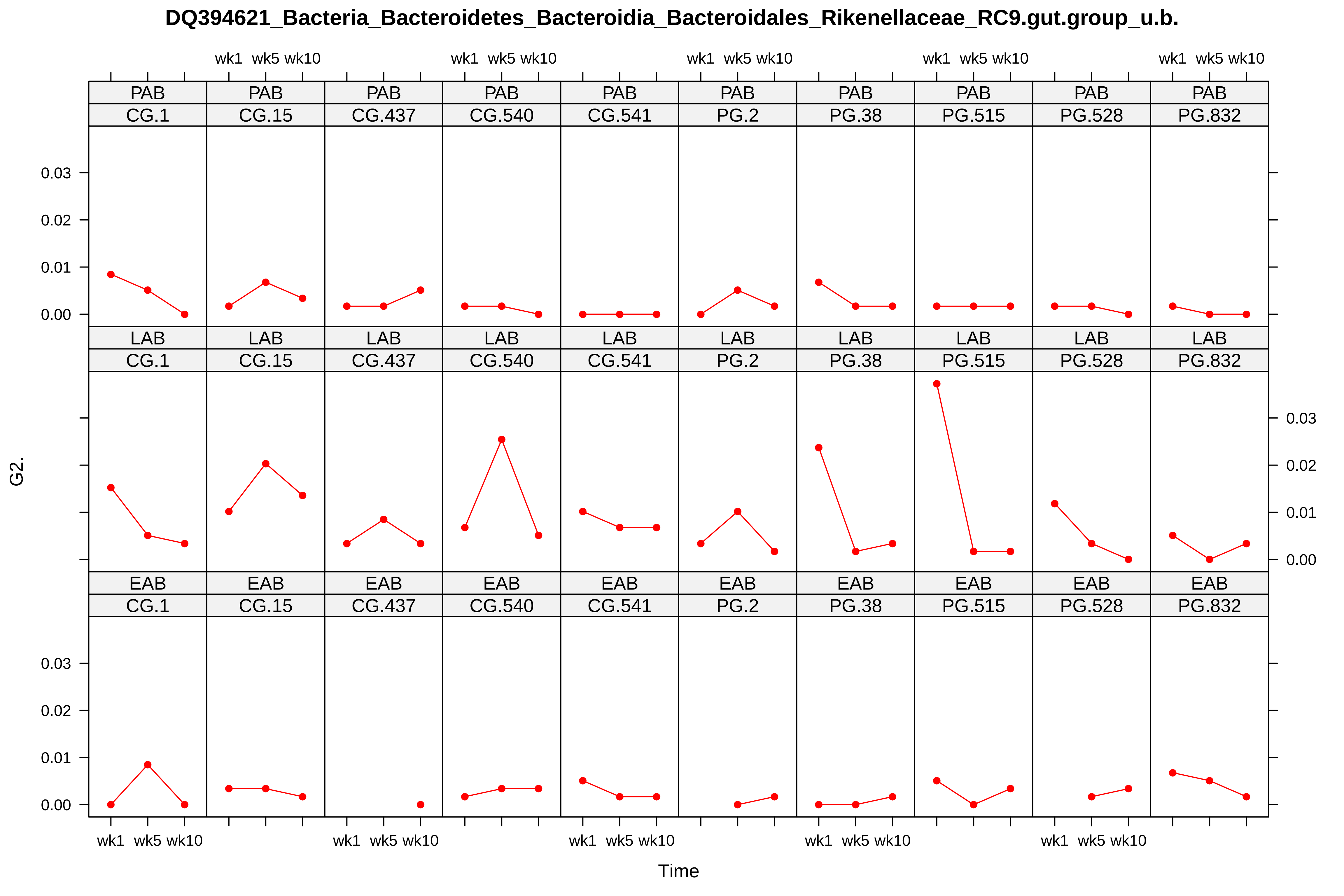

DQ394621_Bacteria_Bacteroidetes_Bacteroidia_Bacteroidales_Rikenellaceae_RC9.gut.group_u.b.
PAB
LAB
EAB
CG.1
CG.15
CG.437
CG.540
CG.541
PG.2
PG.38
PG.515
PG.528
PG.832
wk1 wk5 wk10
0.00
0.01
0.02
0.03
G2.
Time

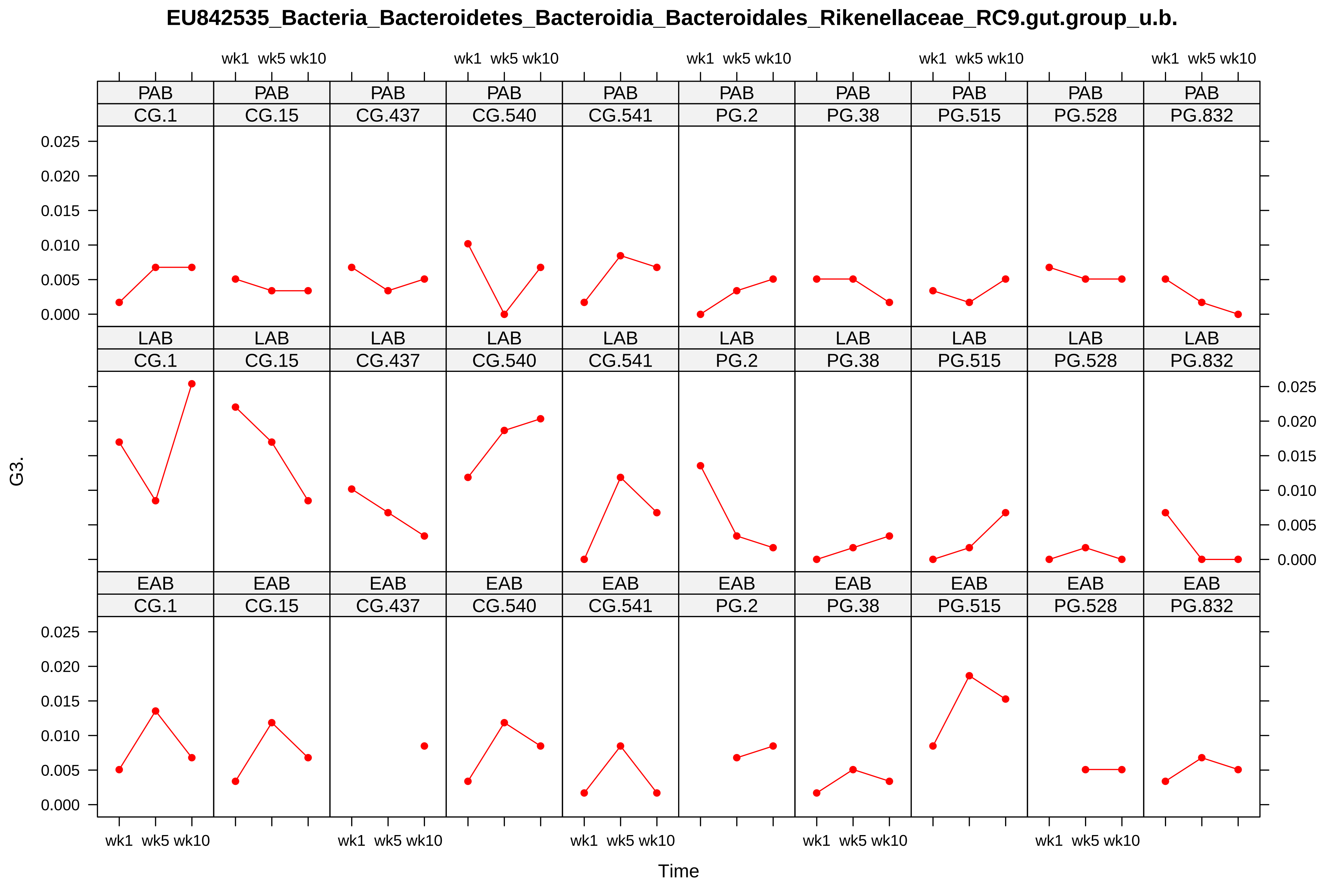
EU842535_Bacteria_Bacteroidetes_Bacteroidia_Bacteroidales_Rikenellaceae_RC9.gut.group_u.b.
PAB
LAB
EAB
CG.1
CG.15
CG.437
CG.540
CG.541
PG.2
PG.38
PG.515
PG.528
PG.832
0.000
0.005
0.010
0.015
0.020
0.025
wk1 wk5 wk10
G3.
Time

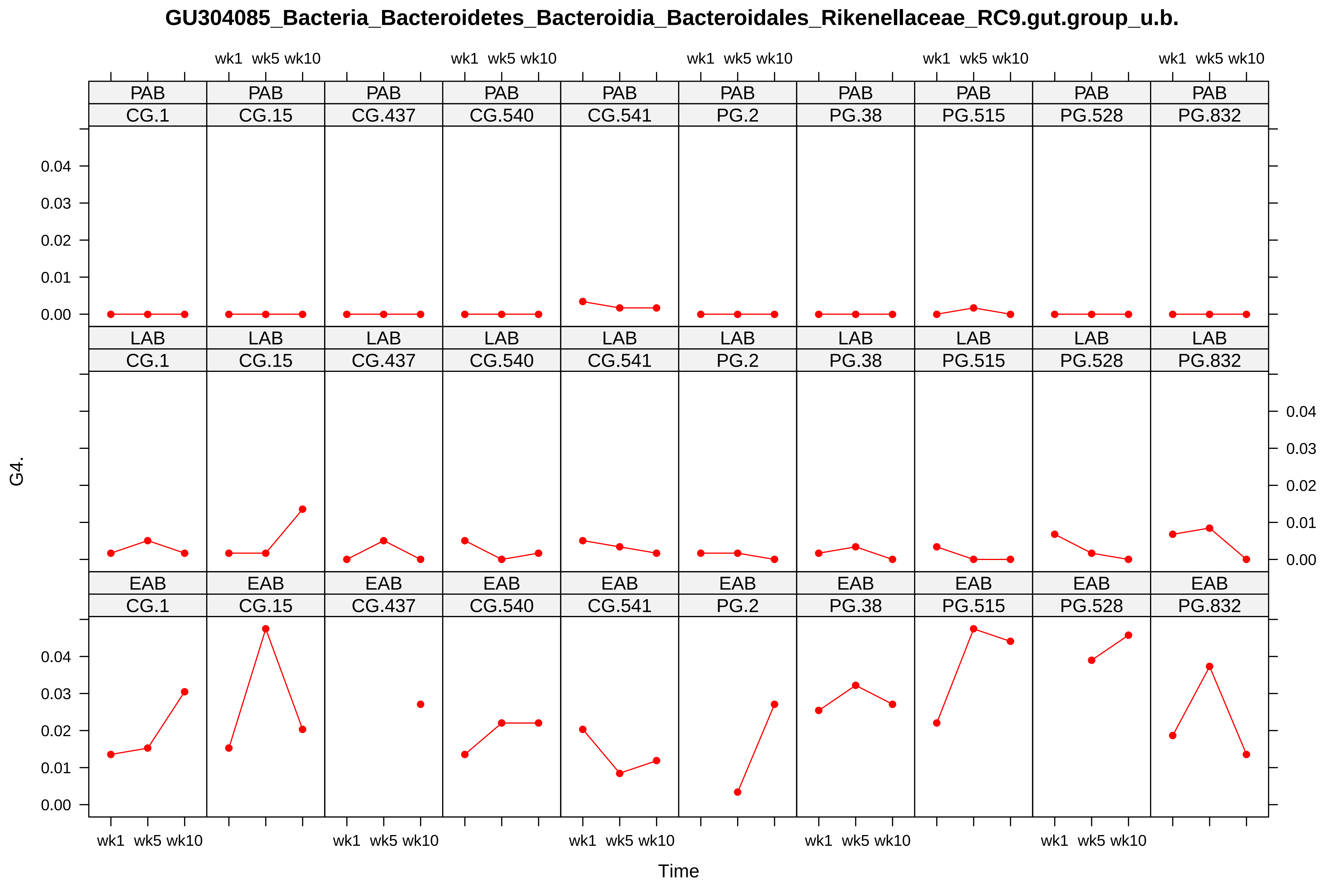

GU304085_Bacteria_Bacteroidetes_Bacteroidia_Bacteroidales_Rikenellaceae_RC9.gut.group_u.b.
PAB
CG.1
CG.15
CG.437
CG.540
CG.541
PG.2
PG.38
PG.515
PG.528
PG.832
LAB
EAB
wk1 wk5 wk10
0.00
0.01
0.02
0.03
0.04
G4.
Time

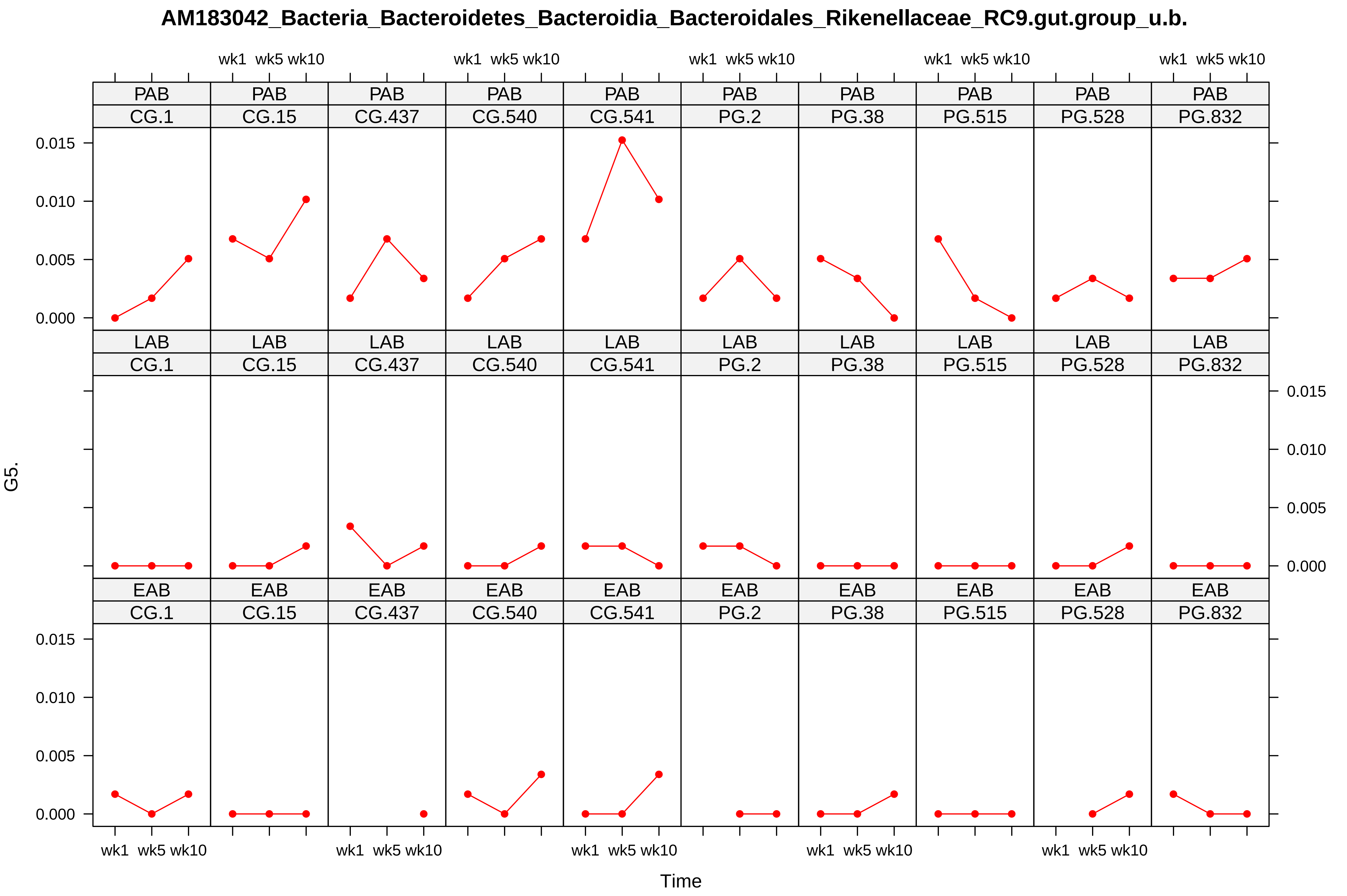
AM183042_Bacteria_Bacteroidetes_Bacteroidia_Bacteroidales_Rikenellaceae_RC9.gut.group_u.b.
PAB
LAB
EAB
CG.1
CG.15
CG.437
CG.540
CG.541
PG.2
PG.38
PG.515
PG.528
PG.832
0.000
0.005
0.010
0.015
G5.
wk1
wk5
wk10
Time

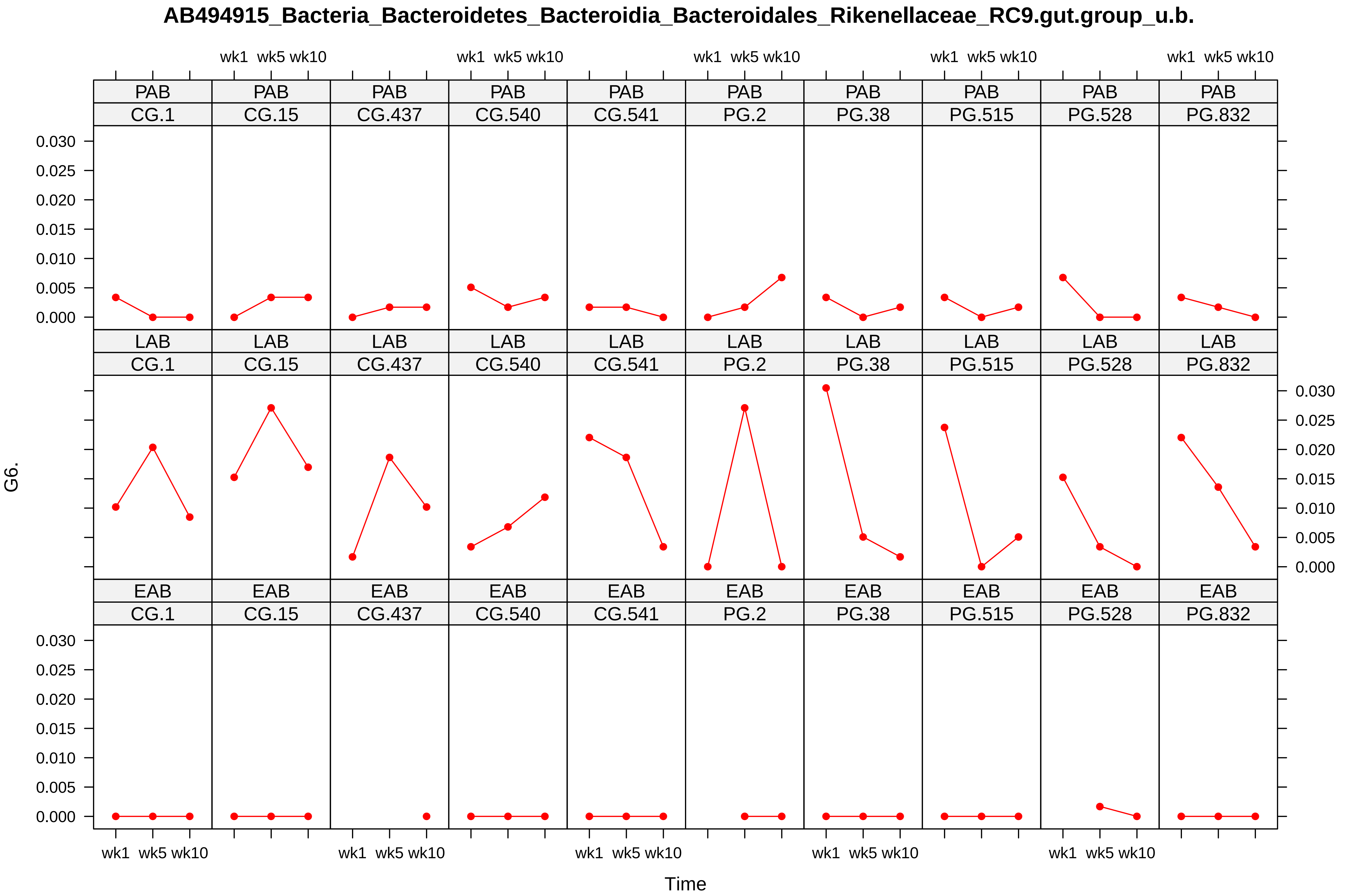

AB494915_Bacteria_Bacteroidetes_Bacteroidia_Bacteroidales_Rikenellaceae_RC9.gut.group_u.b.
PAB
LAB
EAB
CG.1
CG.15
CG.437
CG.540
CG.541
PG.2
PG.38
PG.515
PG.528
PG.832
0.000
0.005
0.010
0.015
0.020
0.025
0.030
wk1
wk5
wk10
G6.
Time

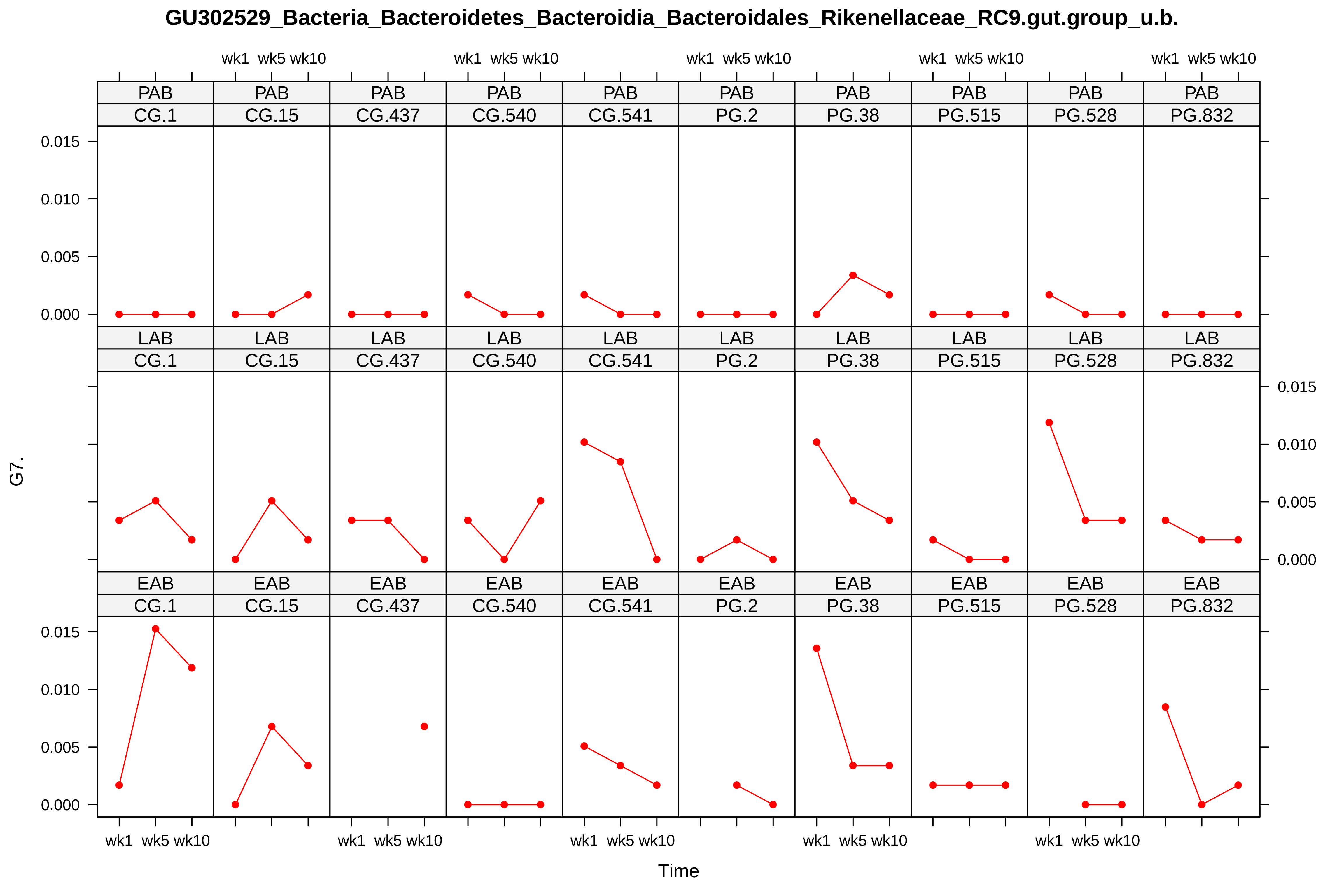

GU302529_Bacteria_Bacteroidetes_Bacteroidia_Bacteroidales_Rikenellaceae_RC9.gut.group_u.b.
PAB
LAB
EAB
CG.1
CG.15
CG.437
CG.540
CG.541
PG.2
PG.38
PG.515
PG.528
PG.832
0.000
0.005
0.010
0.015
wk1 wk5 wk10
G7.
Time

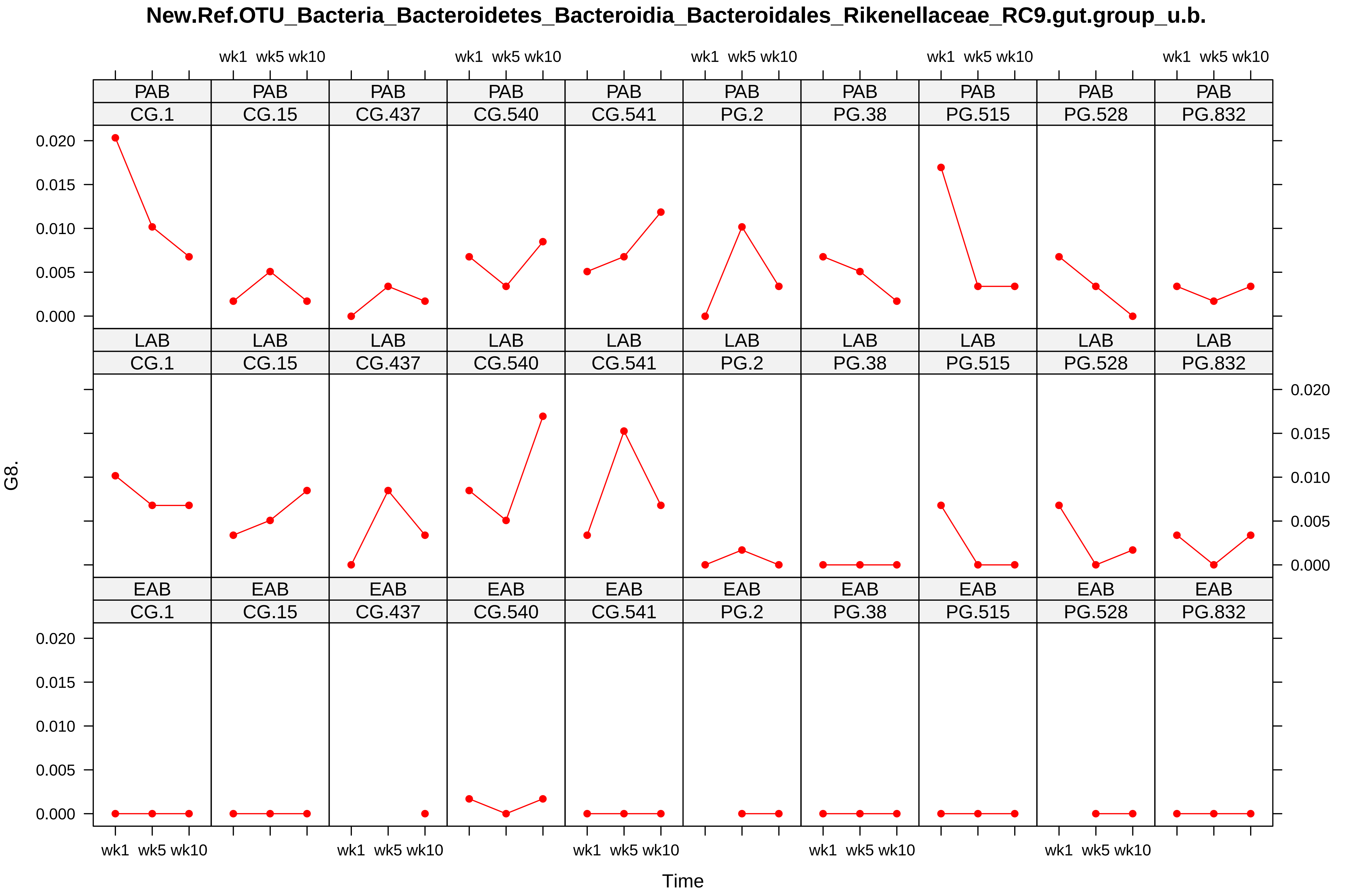
New.Ref.OTU_Bacteria_Bacteroidetes_Bacteroidia_Bacteroidales_Rikenellaceae_RC9.gut.group_u.b.
PAB
LAB
EAB
CG.1
CG.15
CG.437
CG.540
CG.541
PG.2
PG.38
PG.515
PG.528
PG.832
0.000
0.005
0.010
0.015
0.020
wk1
wk5
wk10
G8.
Time

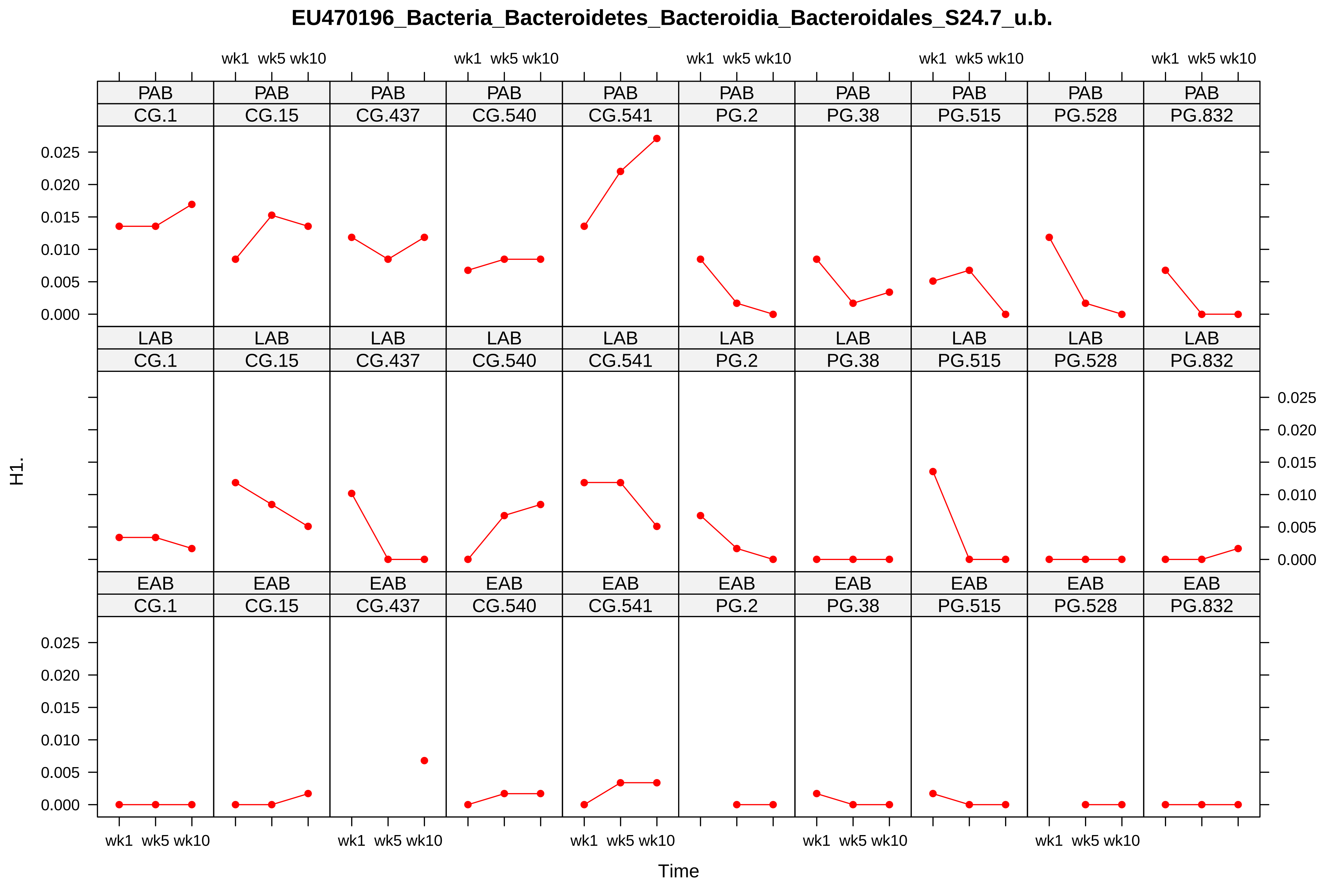
EU470196_Bacteria_Bacteroidetes_Bacteroidia_Bacteroidales_S24.7_u.b.
PAB
LAB
EAB
CG.1
CG.15
CG.437
CG.540
CG.541
PG.2
PG.38
PG.515
PG.528
PG.832
0.000
0.005
0.010
0.015
0.020
0.025
wk1 wk5 wk10
H1.
Time

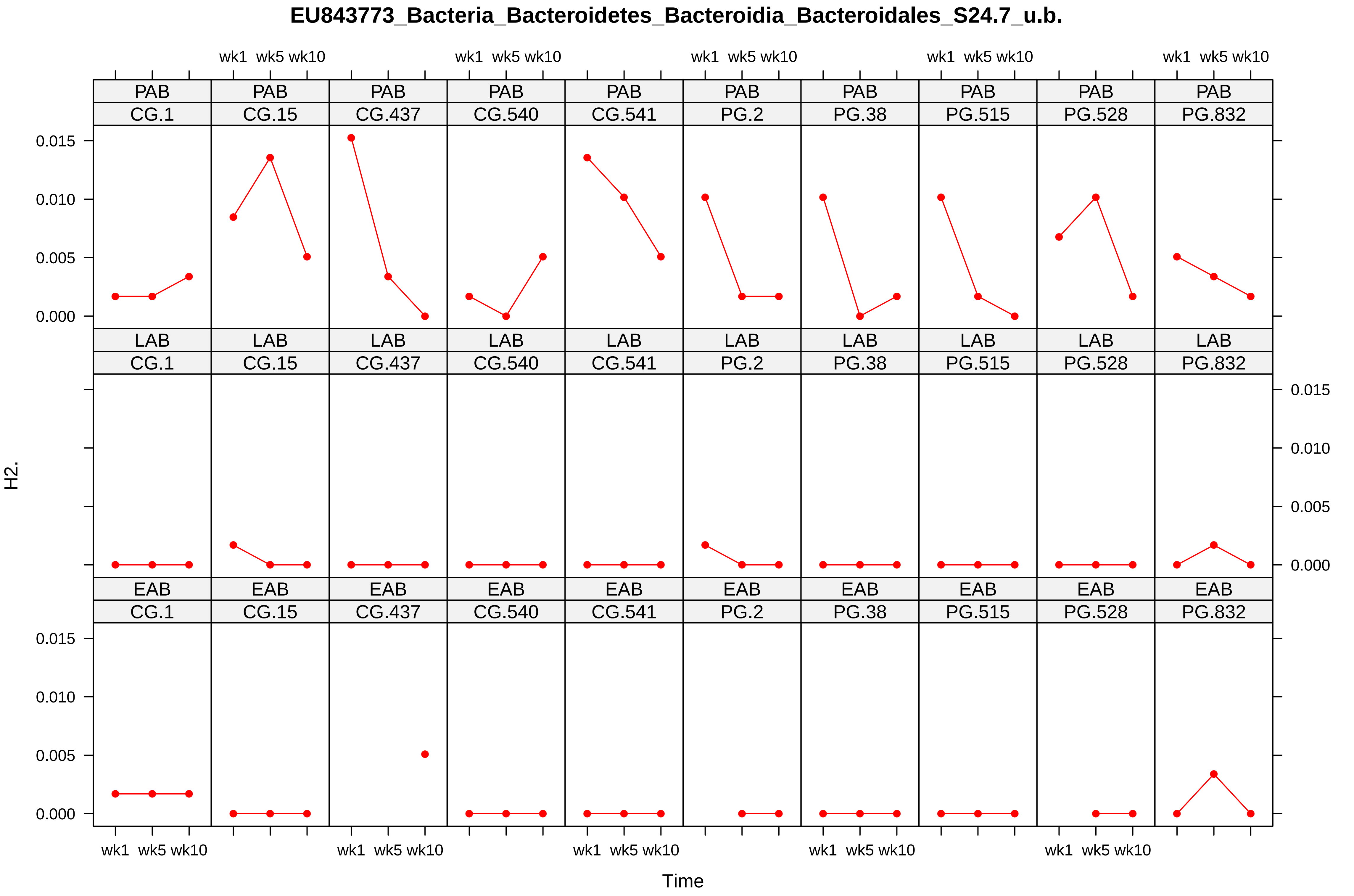
EU843773_Bacteria_Bacteroidetes_Bacteroidia_Bacteroidales_S24.7_u.b.
PAB
LAB
EAB
CG.1
CG.15
CG.437
CG.540
CG.541
PG.2
PG.38
PG.515
PG.528
PG.832
0.000
0.005
0.010
0.015
wk1
wk5
wk10
H2.
Time

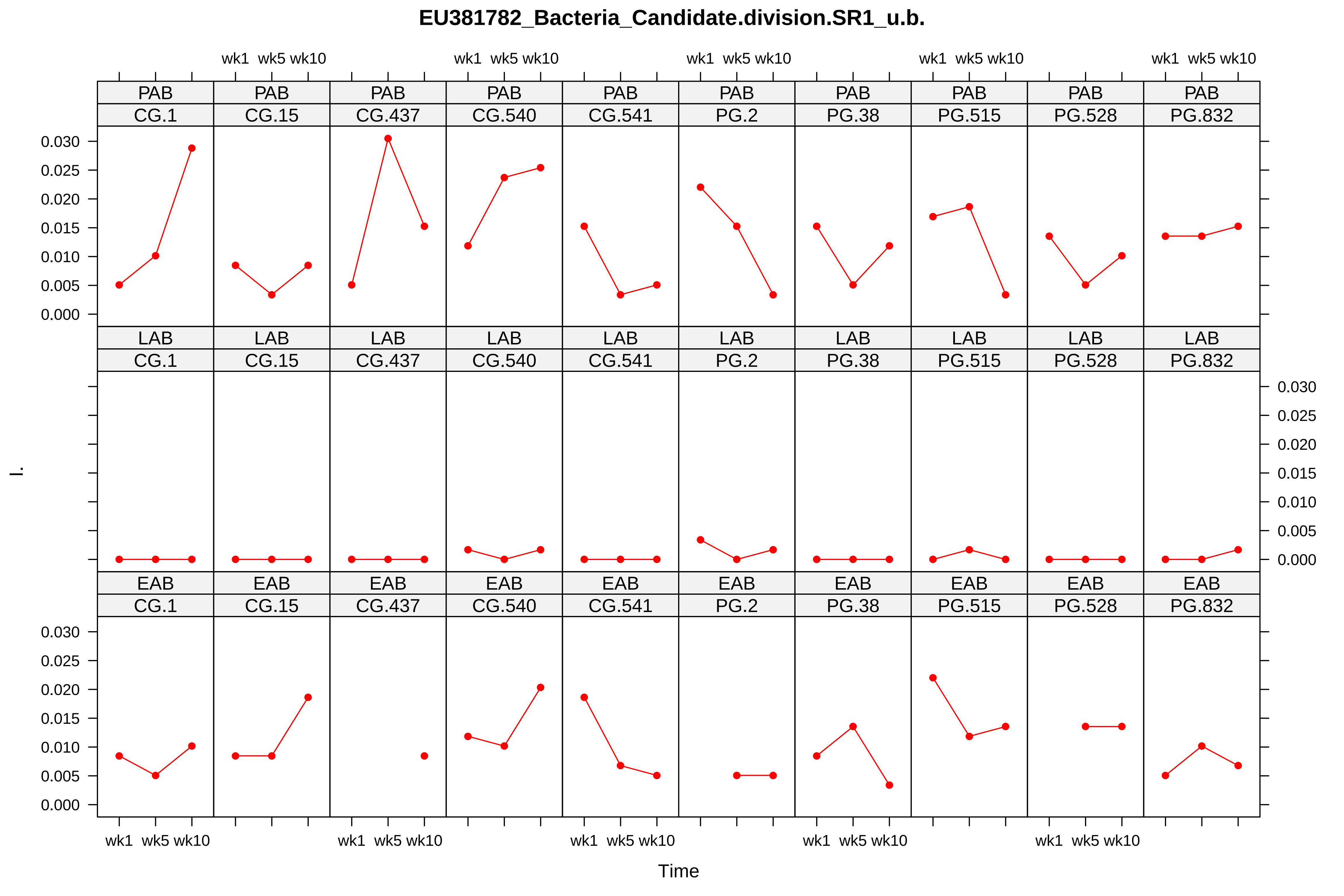

EU381782_Bacteria_Candidate.division.SR1_u.b.
PAB
LAB
EAB
CG.1
CG.15
CG.437
CG.540
CG.541
PG.2
PG.38
PG.515
PG.528
PG.832
0.000
0.005
0.010
0.015
0.020
0.025
0.030
wk1 wk5 wk10
I.
Time

EU462203_Bacteria_Candidate.division.TM7_u.b.

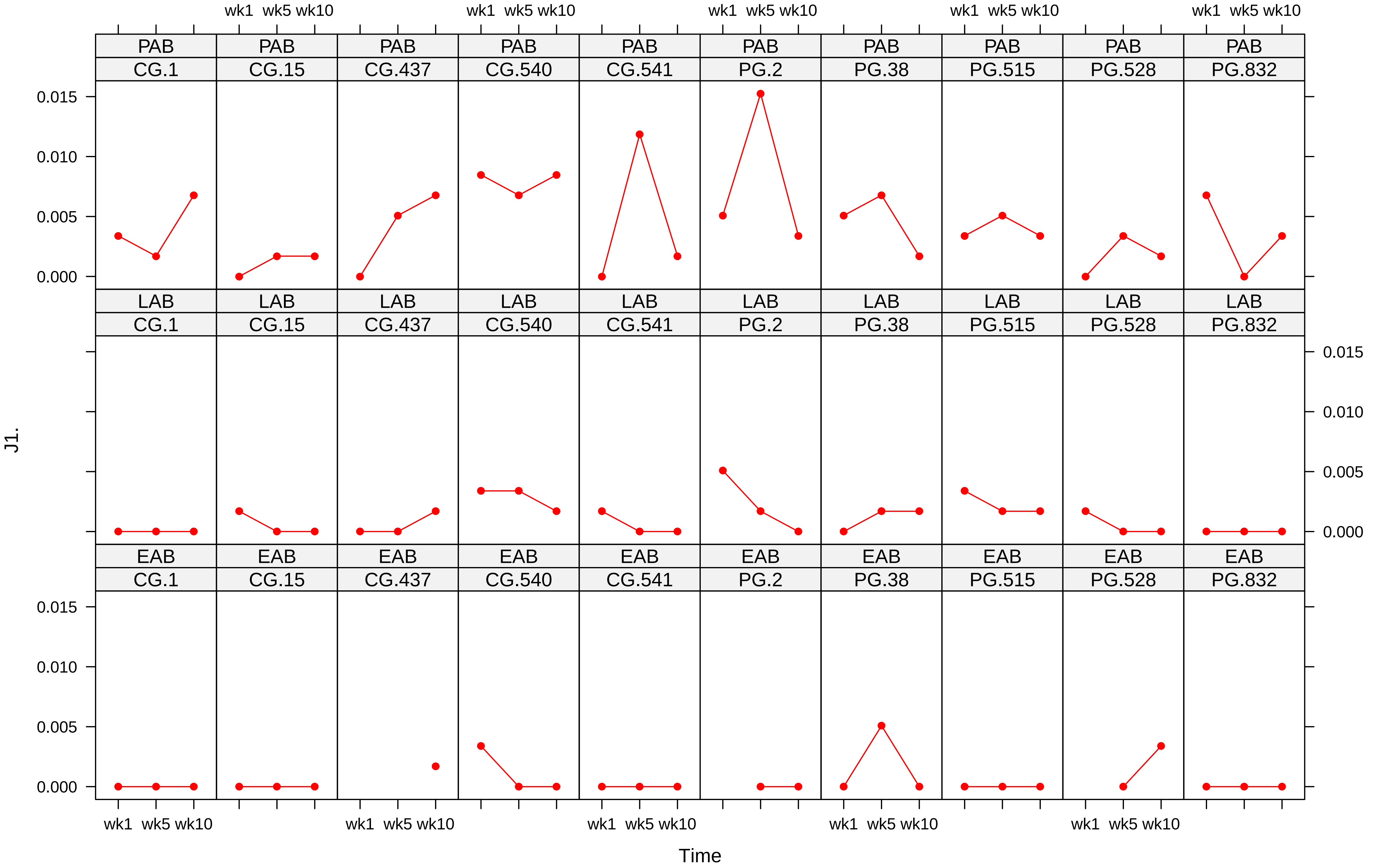

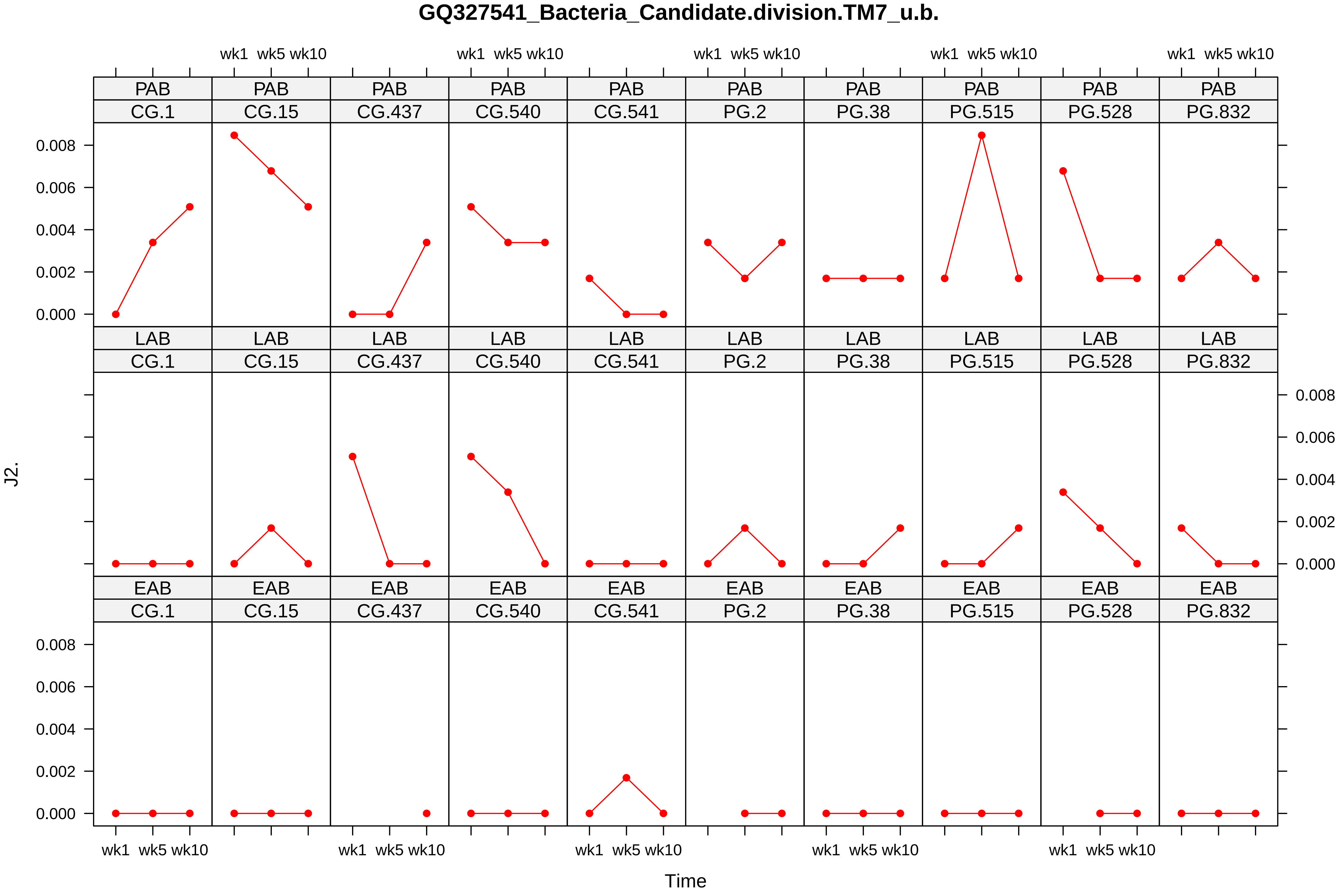

GQ327541_Bacteria_Candidate.division.TM7_u.b.
PAB
LAB
EAB
CG.1
CG.15
CG.437
CG.540
CG.541
PG.2
PG.38
PG.515
PG.528
PG.832
0.000
0.002
0.004
0.006
0.008
wk1 wk5 wk10
J2.
Time

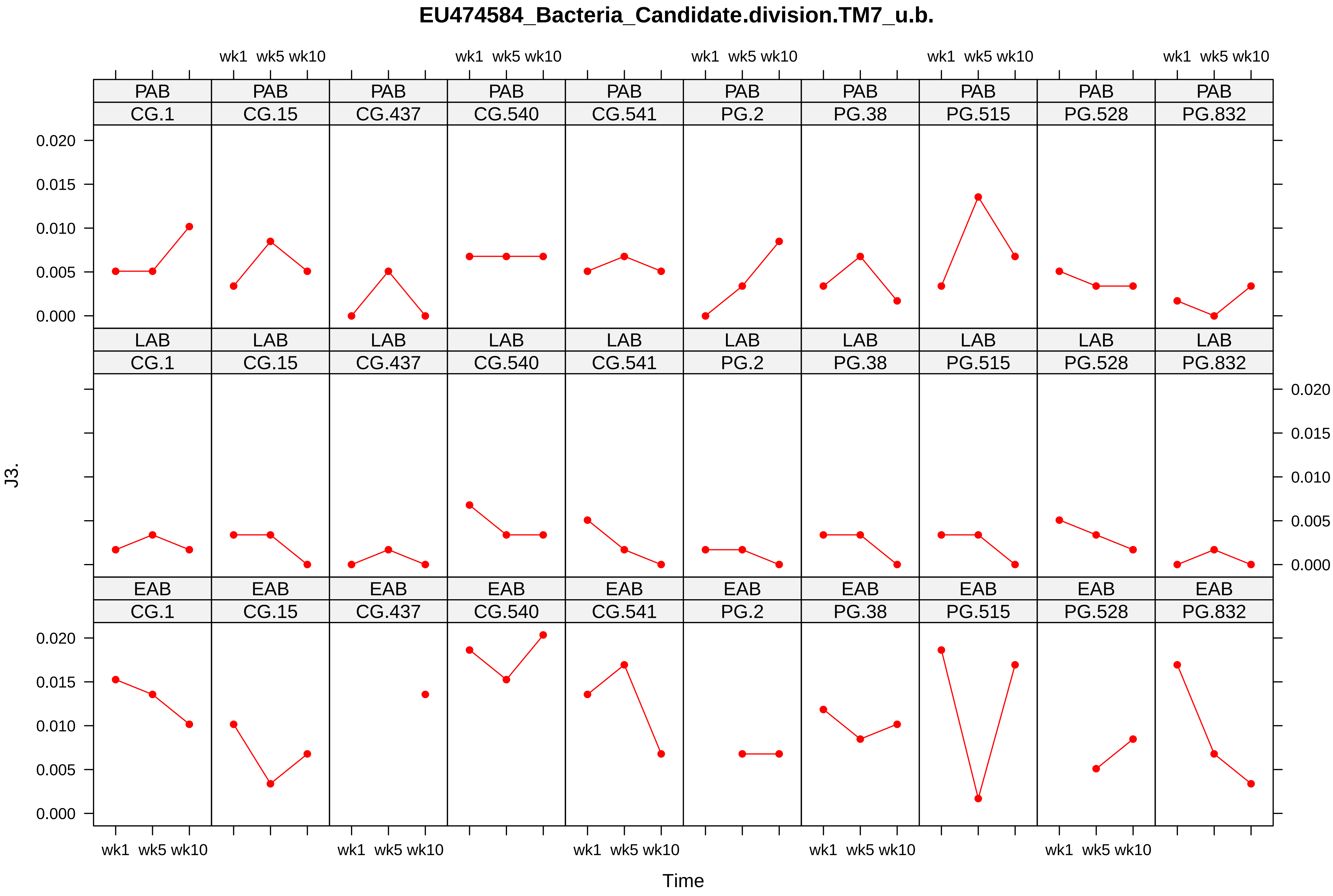
EU474584_Bacteria_Candidate.division.TM7_u.b.
PAB
LAB
EAB
CG.1
CG.15
CG.437
CG.540
CG.541
PG.2
PG.38
PG.515
PG.528
PG.832
J3.
Time
wk1 wk5 wk10
0.000
0.005
0.010
0.015
0.020

EU381496_Bacteria_Candidate.division.TM7_u.b.

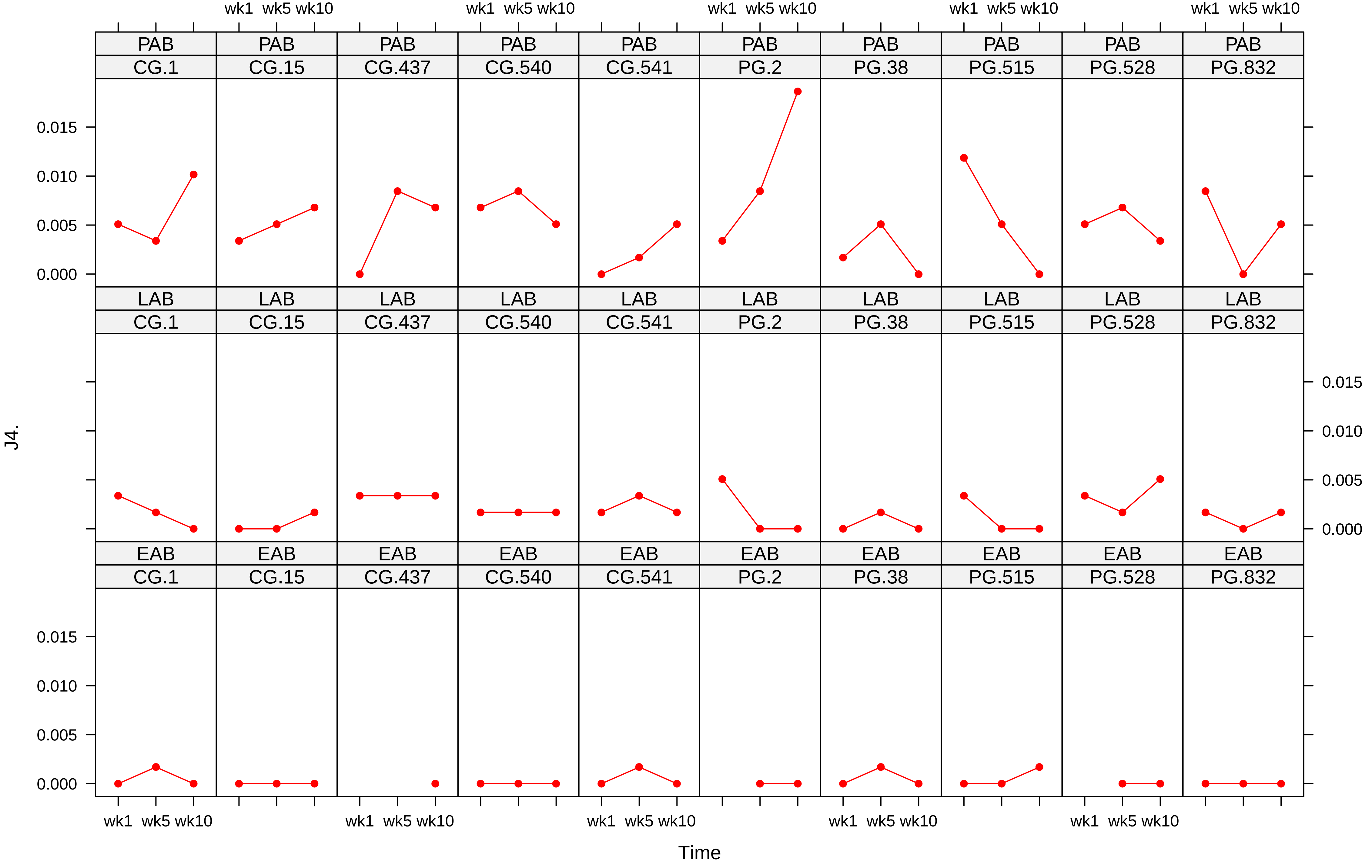

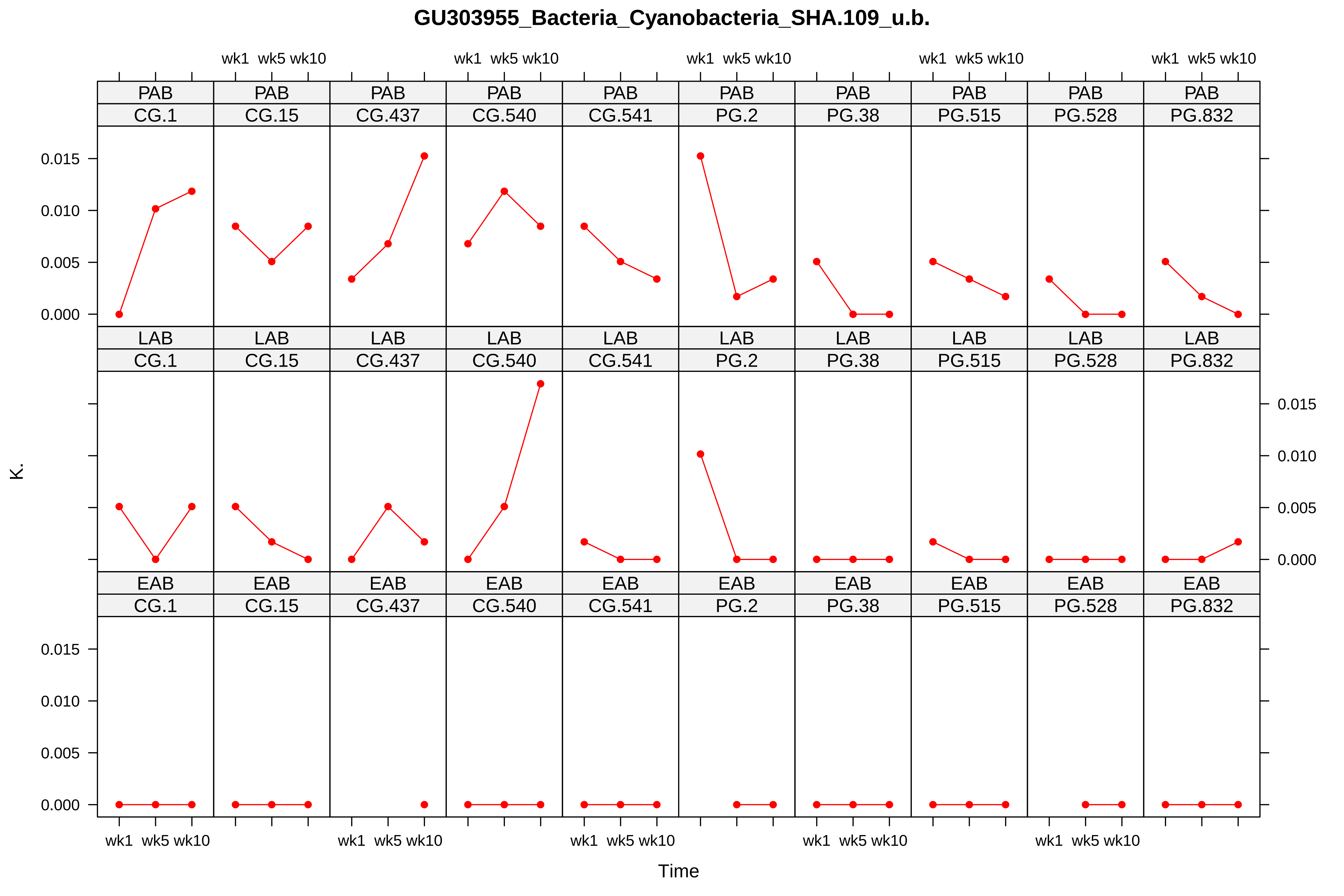
GU303955_Bacteria_Cyanobacteria_SHA.109_u.b.
PAB
LAB
EAB
CG.1
CG.15
CG.437
CG.540
CG.541
PG.2
PG.38
PG.515
PG.528
PG.832
0.000
0.005
0.010
0.015
wk1
wk5
wk10
K.
Time

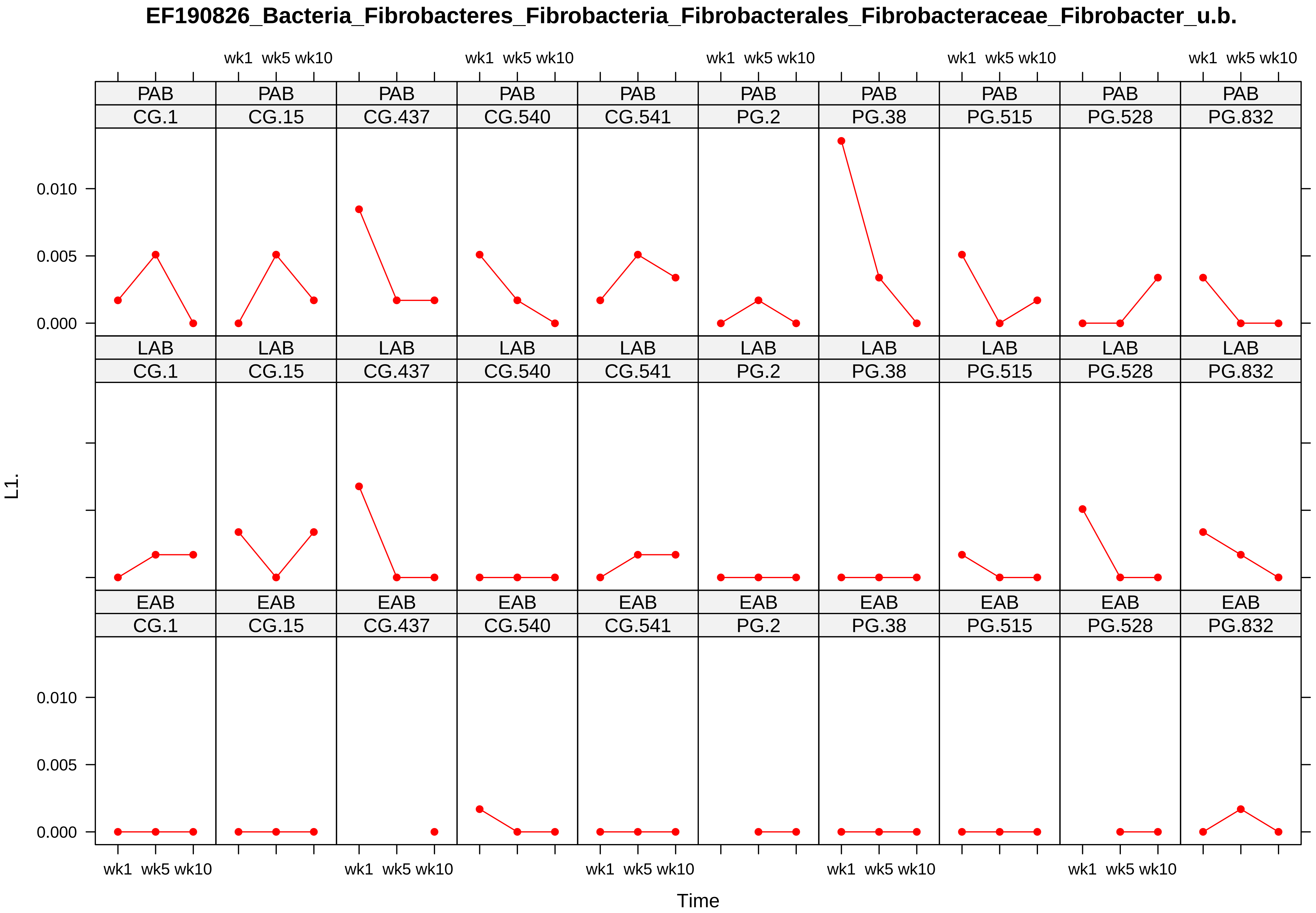

EF190826_Bacteria_Fibrobacteres_Fibrobacteria_Fibrobacterales_Fibrobacteraceae_Fibrobacter_u.b.
PAB
LAB
EAB
CG.1
CG.15
CG.437
CG.540
CG.541
PG.2
PG.38
PG.515
PG.528
PG.832
0.000
0.005
0.010
wk1 wk5 wk10
L1.
Time

EU381811_Bacteria_Fibrobacteres_Fibrobacteria_Fibrobacterales_Fibrobacteraceae_Fibrobacter_u.b.

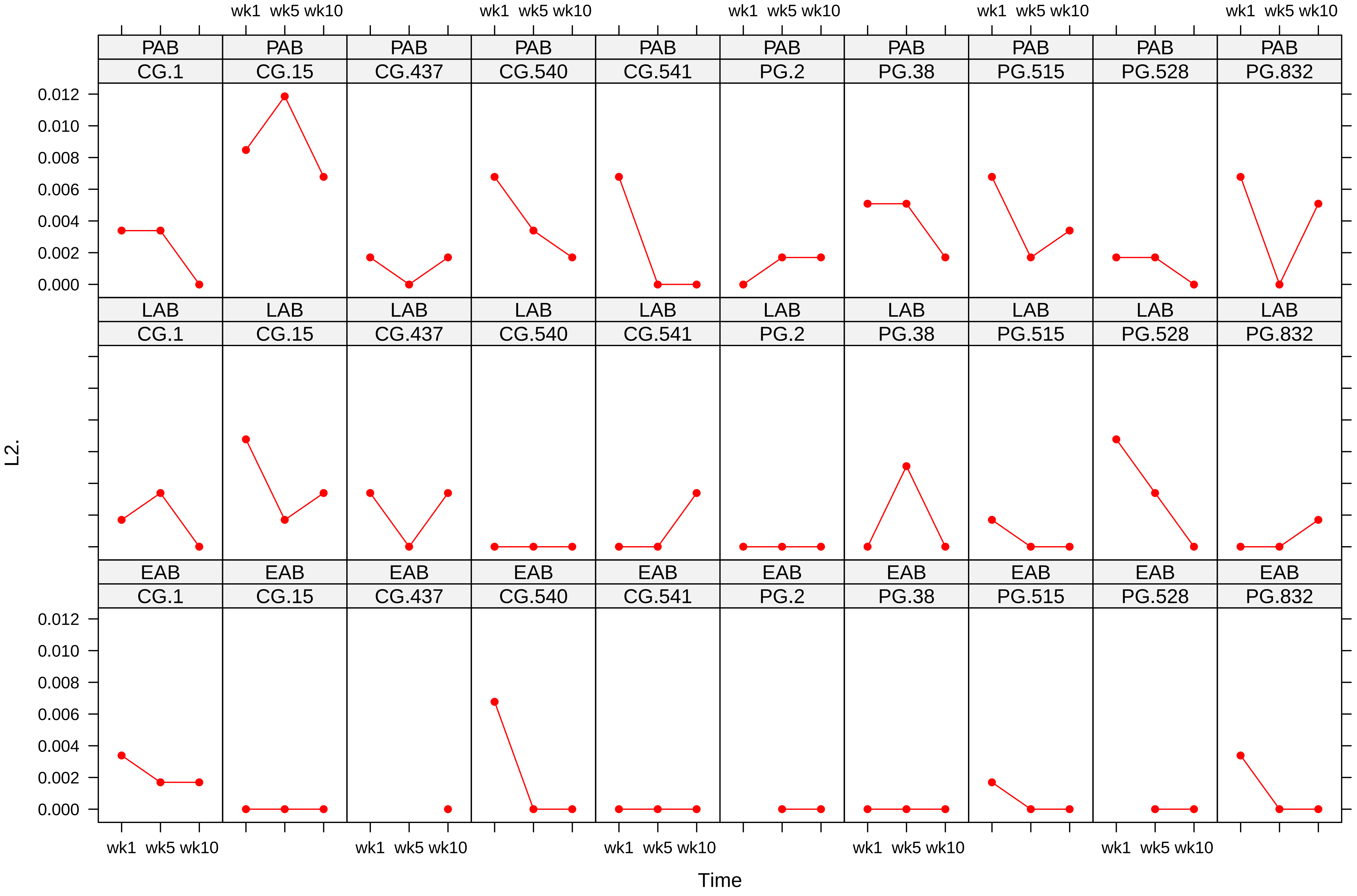

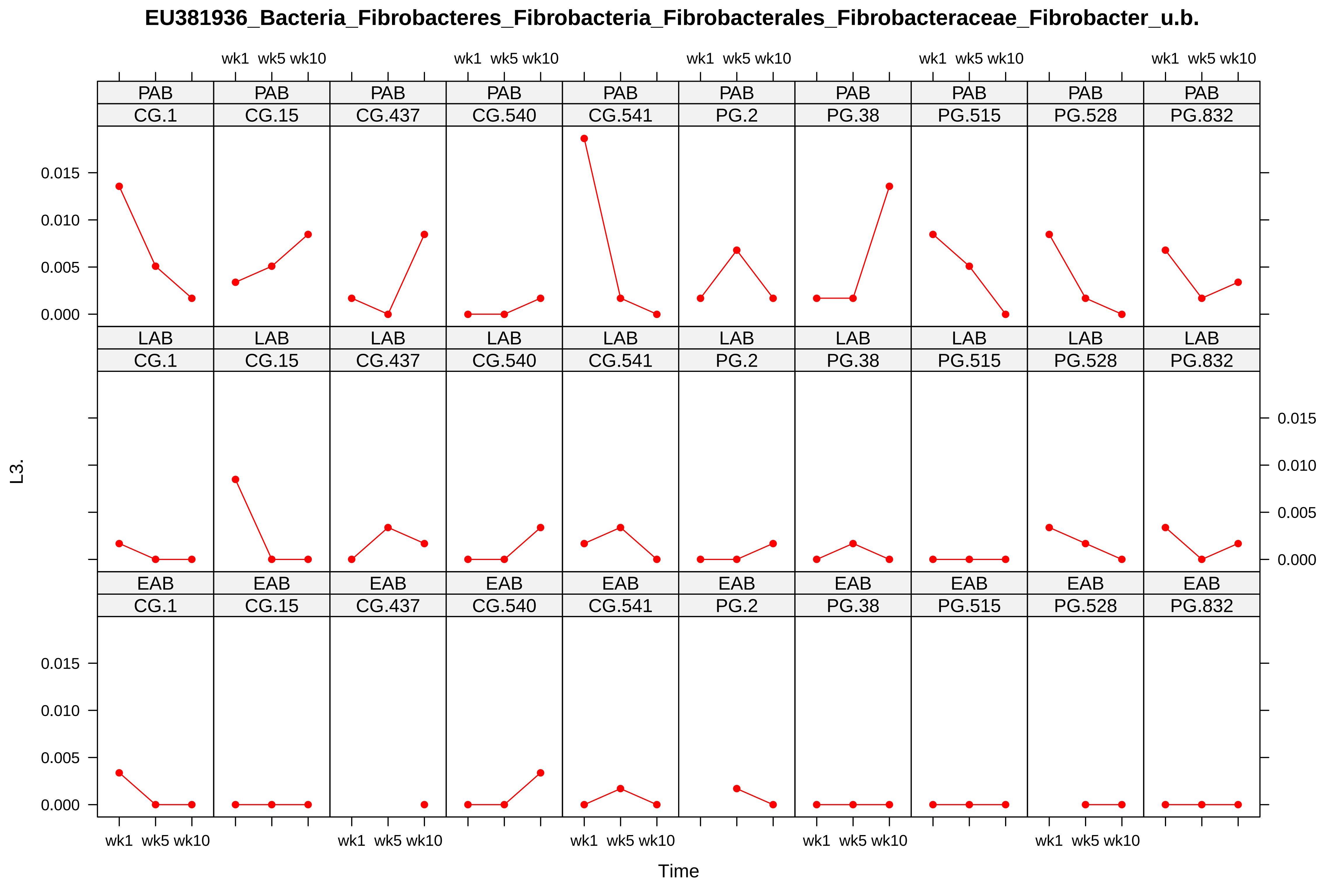

EU381936_Bacteria_Fibrobacteres_Fibrobacteria_Fibrobacterales_Fibrobacteraceae_Fibrobacter_u.b.
PAB
LAB
EAB
CG.1
CG.15
CG.437
CG.540
CG.541
PG.2
PG.38
PG.515
PG.528
PG.832
0.000
0.005
0.010
0.015
wk1
wk5
wk10
L3.
Time

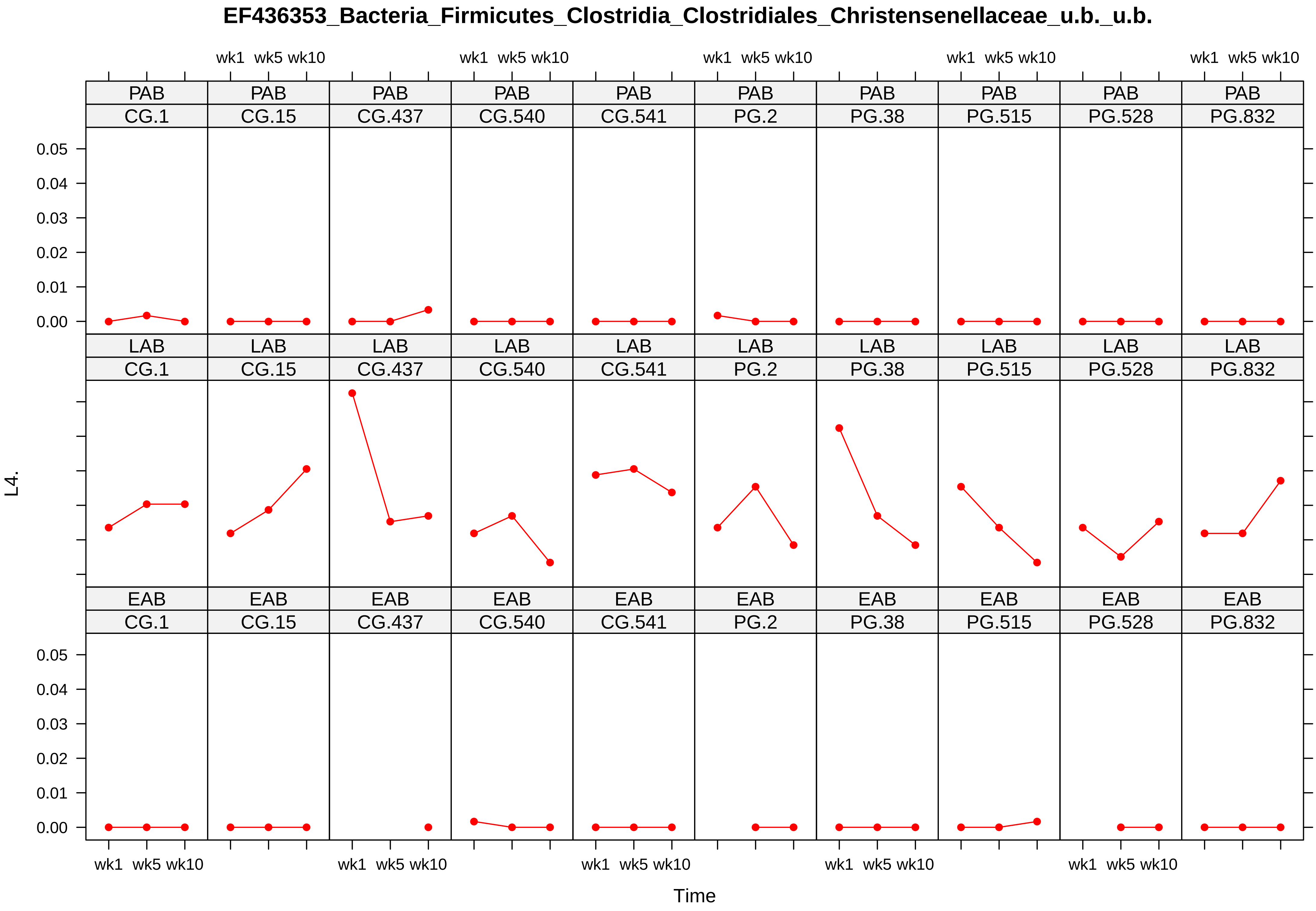
EF436353_Bacteria_Firmicutes_Clostridia_Clostridiales_Christensenellaceae_u.b._u.b.
PAB
LAB
EAB
CG.1
CG.15
CG.437
CG.540
CG.541
PG.2
PG.38
PG.515
PG.528
PG.832
0.00
0.01
0.02
0.03
0.04
0.05
wk1
wk5
wk10
L4.
Time

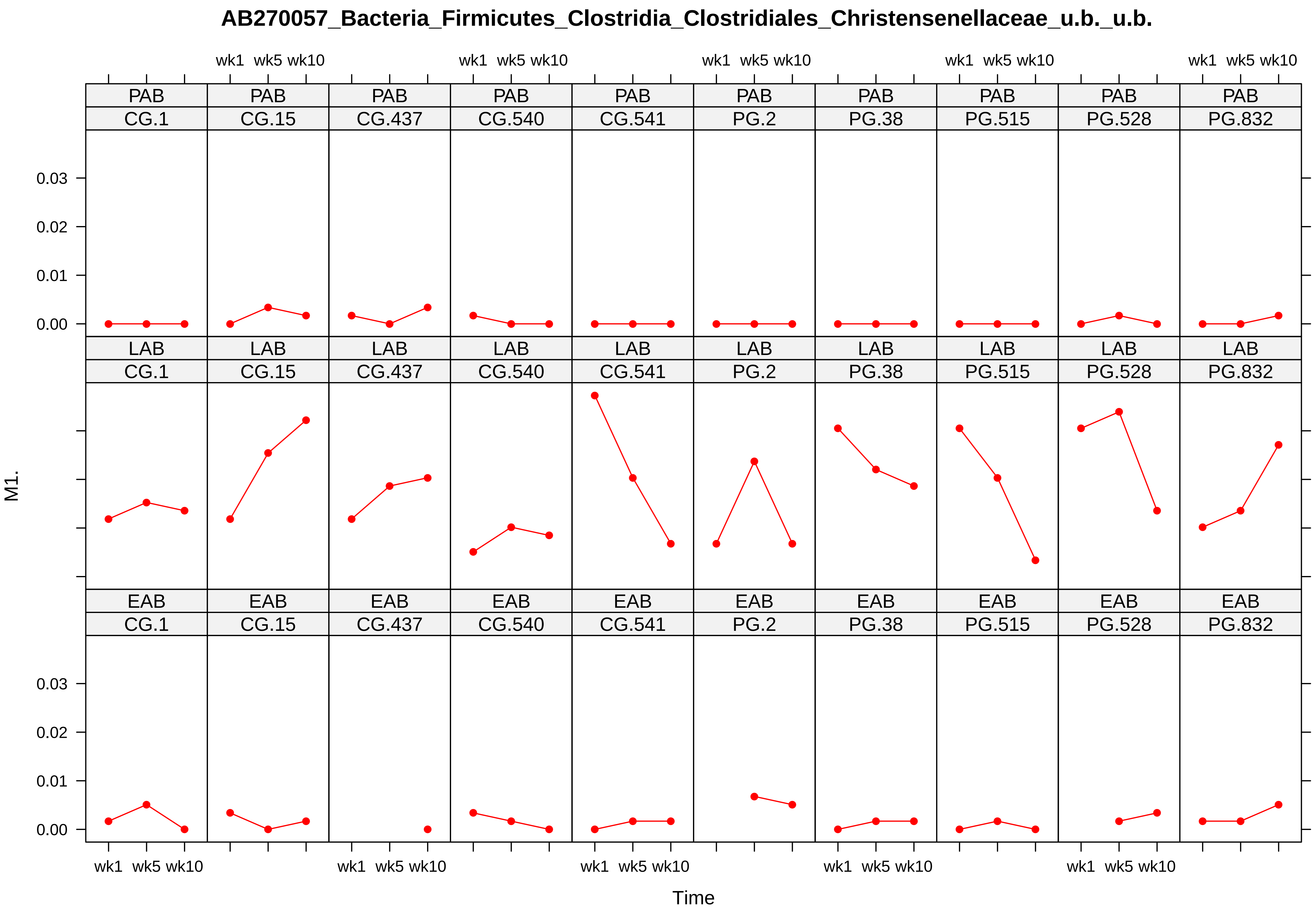
AB270057_Bacteria_Firmicutes_Clostridia_Clostridiales_Christensenellaceae_u.b._u.b.
PAB
LAB
EAB
CG.1
CG.15
CG.437
CG.540
CG.541
PG.2
PG.38
PG.515
PG.528
PG.832
0.00
0.01
0.02
0.03
wk1
wk5
wk10
M1.
Time

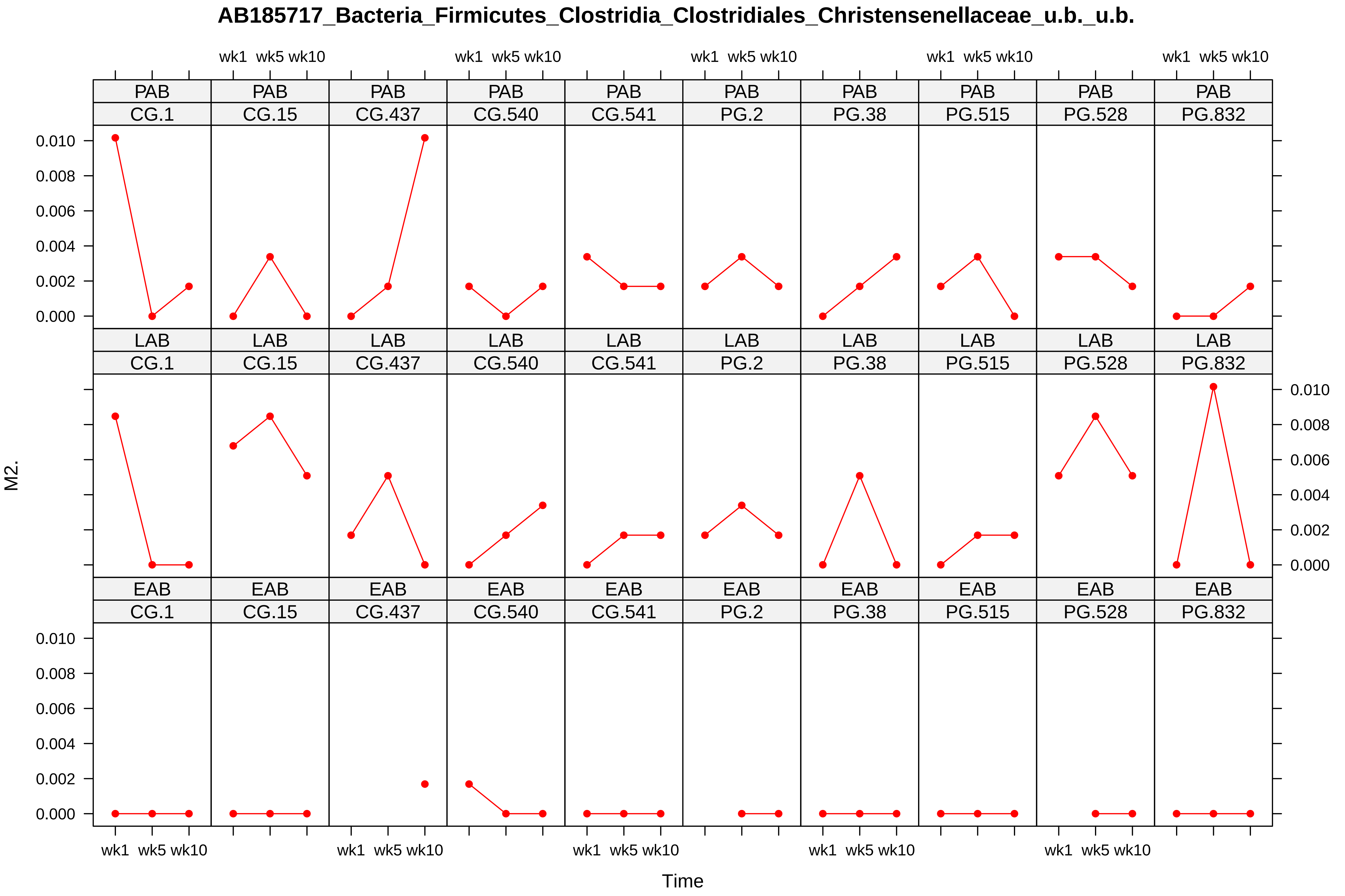
AB185717_Bacteria_Firmicutes_Clostridia_Clostridiales_Christensenellaceae_u.b._u.b.
PAB
LAB
EAB
CG.1
CG.15
CG.437
CG.540
CG.541
PG.2
PG.38
PG.515
PG.528
PG.832
wk1 wk5 wk10
0.000
0.002
0.004
0.006
0.008
0.010
M2.
Time

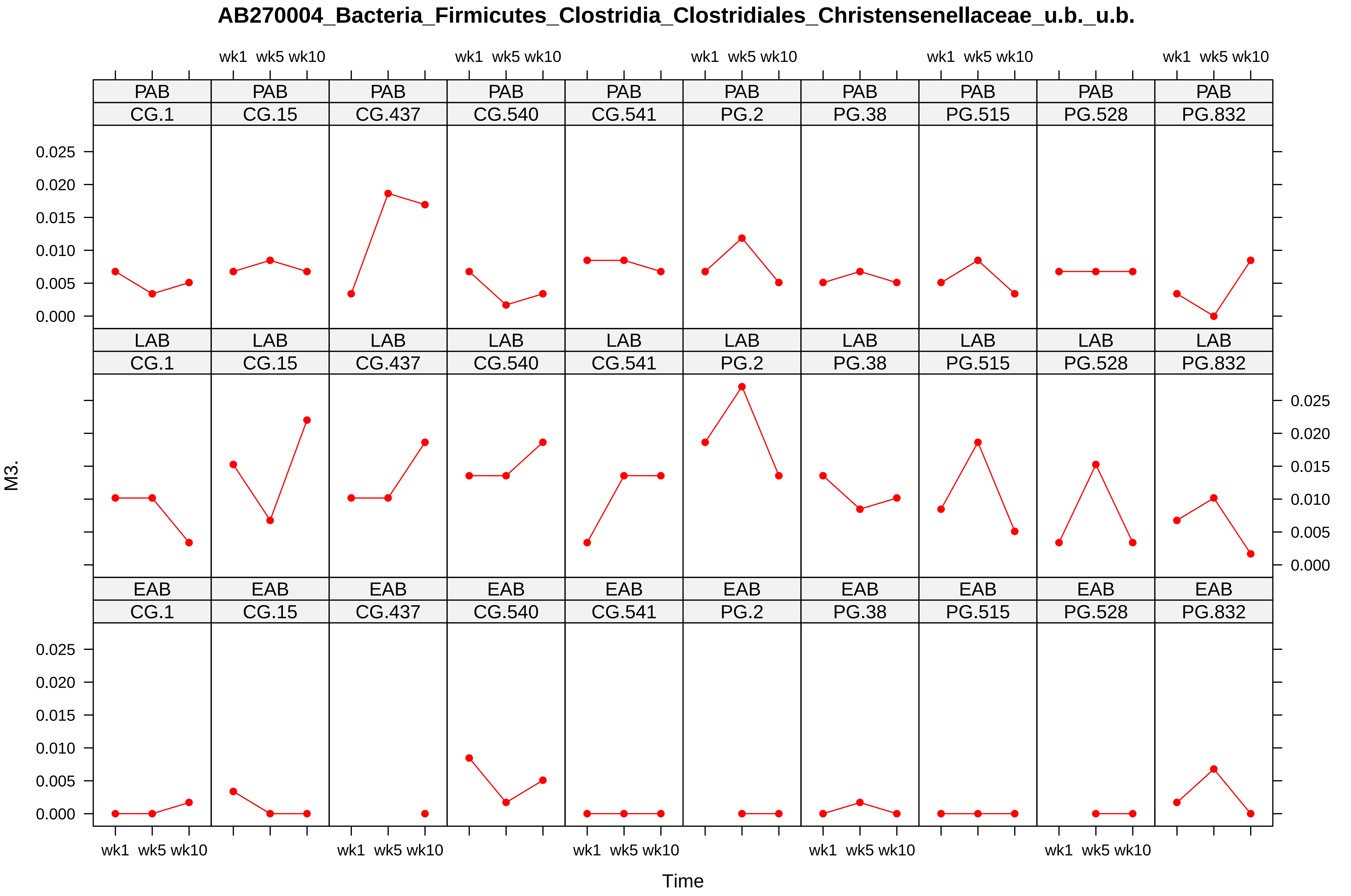

AB270004_Bacteria_Firmicutes_Clostridia_Clostridiales_Christensenellaceae_u.b._u.b.
PAB
LAB
EAB
CG.1
CG.15
CG.437
CG.540
CG.541
PG.2
PG.38
PG.515
PG.528
PG.832
0.000
0.005
0.010
0.015
0.020
0.025
wk1 wk5 wk10
M3.
Time

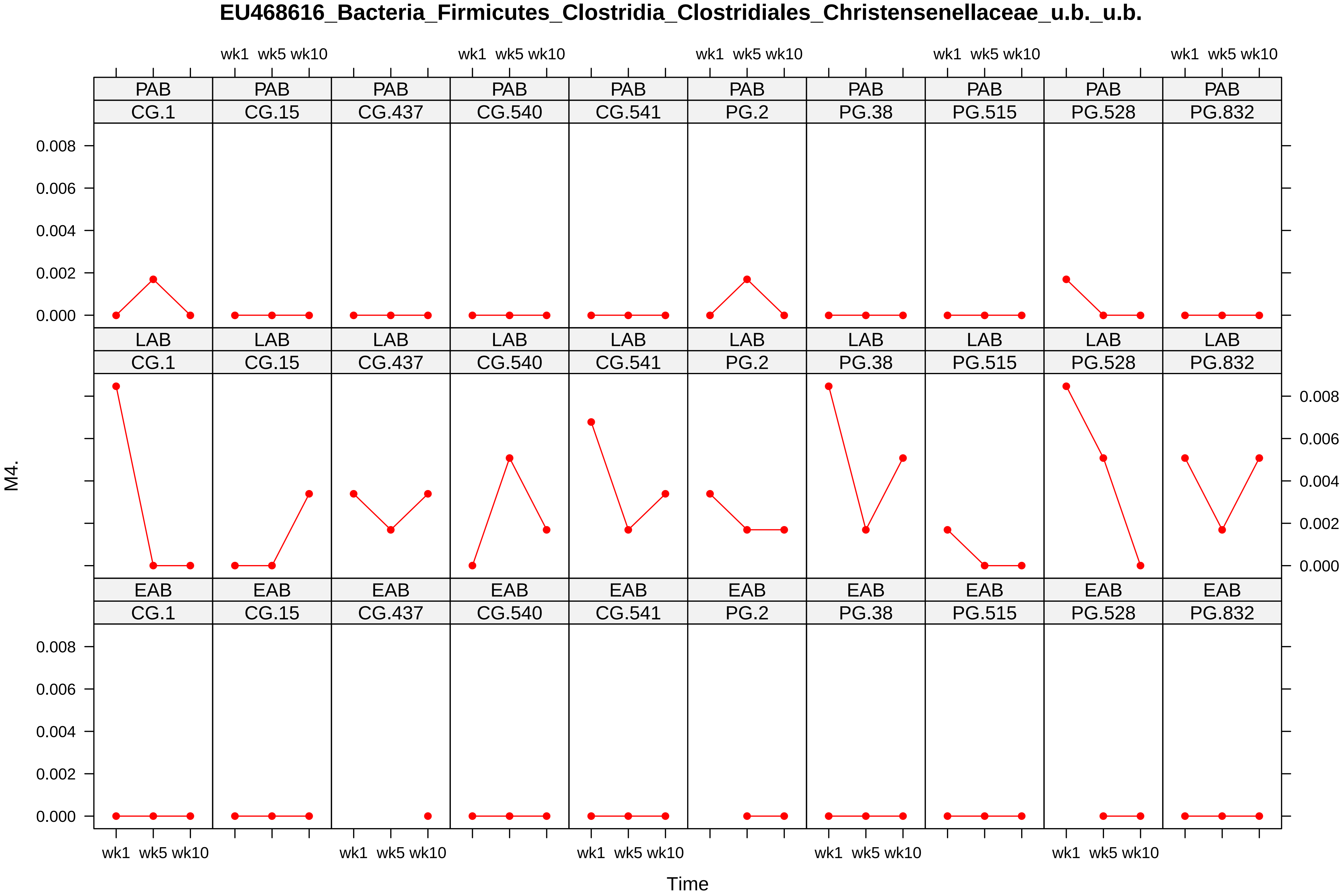

EU468616_Bacteria_Firmicutes_Clostridia_Clostridiales_Christensenellaceae_u.b._u.b.
PAB
LAB
EAB
CG.1
CG.15
CG.437
CG.540
CG.541
PG.2
PG.38
PG.515
PG.528
PG.832
0.000
0.002
0.004
0.006
0.008
wk1 wk5 wk10
M4.
Time

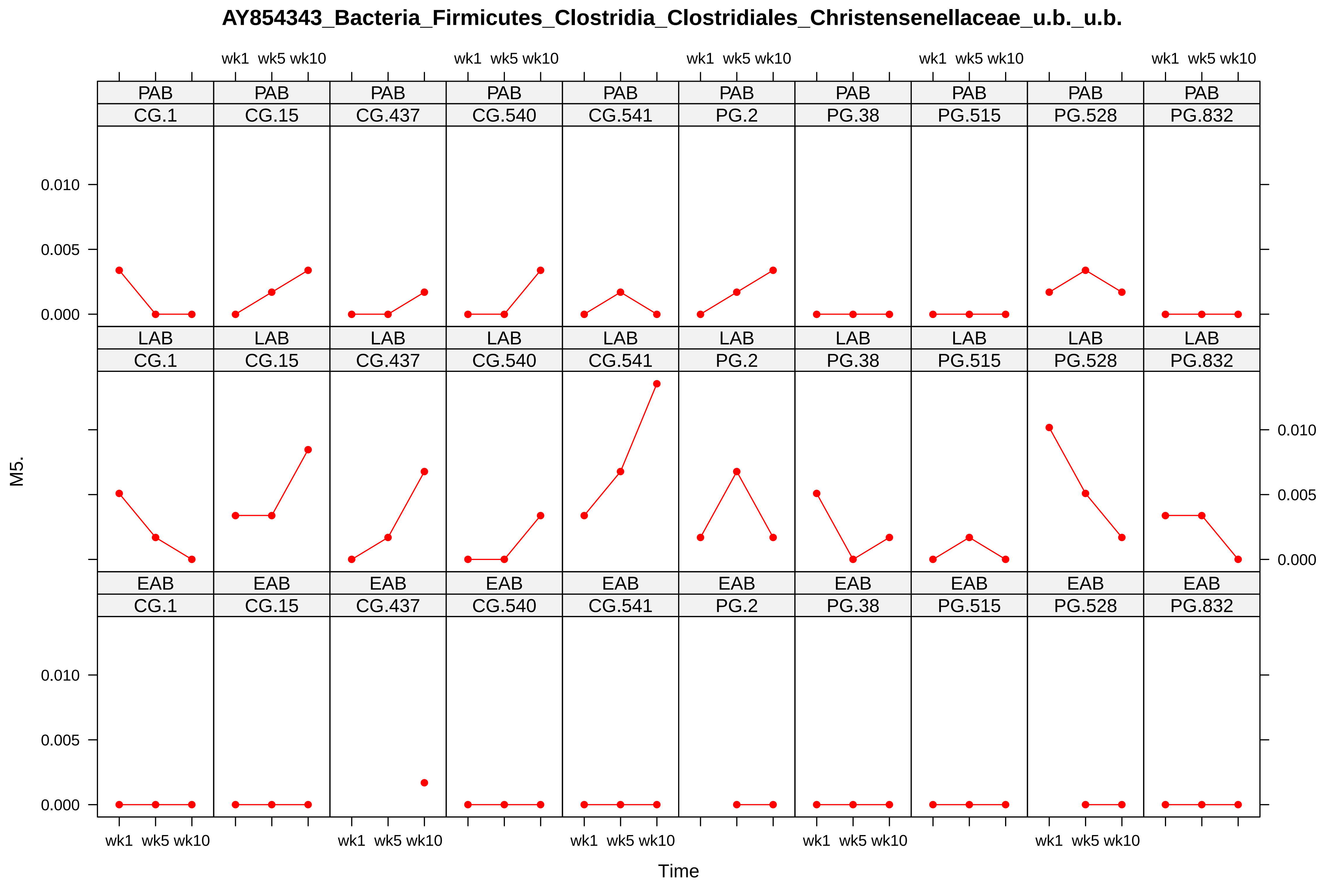
AY854343_Bacteria_Firmicutes_Clostridia_Clostridiales_Christensenellaceae_u.b._u.b.
PAB
LAB
EAB
CG.1
CG.15
CG.437
CG.540
CG.541
PG.2
PG.38
PG.515
PG.528
PG.832
0.000
0.005
0.010
wk1 wk5 wk10
M5.
Time

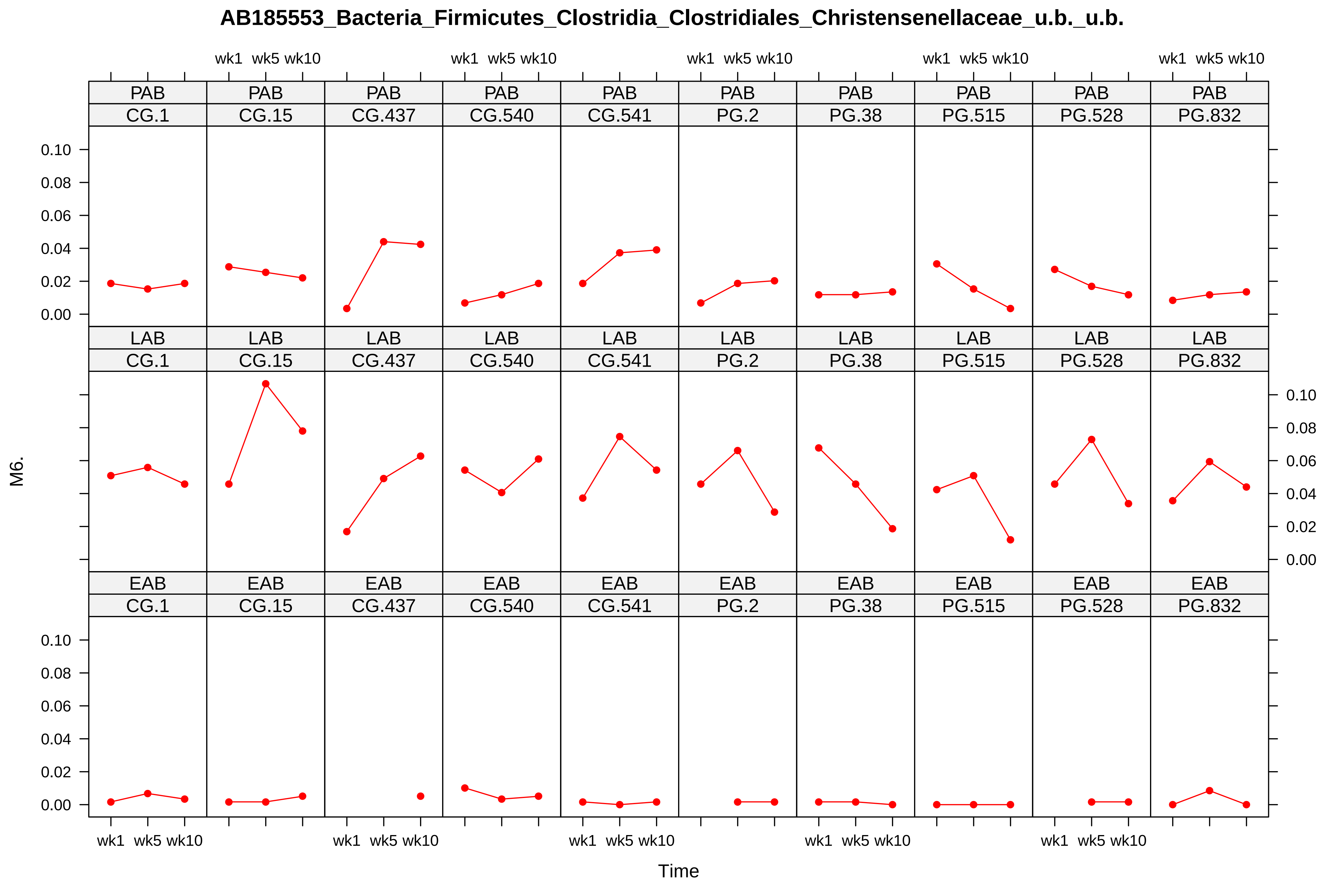

AB185553_Bacteria_Firmicutes_Clostridia_Clostridiales_Christensenellaceae_u.b._u.b.
PAB
LAB
EAB
CG.1
CG.15
CG.437
CG.540
CG.541
PG.2
PG.38
PG.515
PG.528
PG.832
0.00
0.02
0.04
0.06
0.08
0.10
wk1
wk5
wk10
M6.
Time

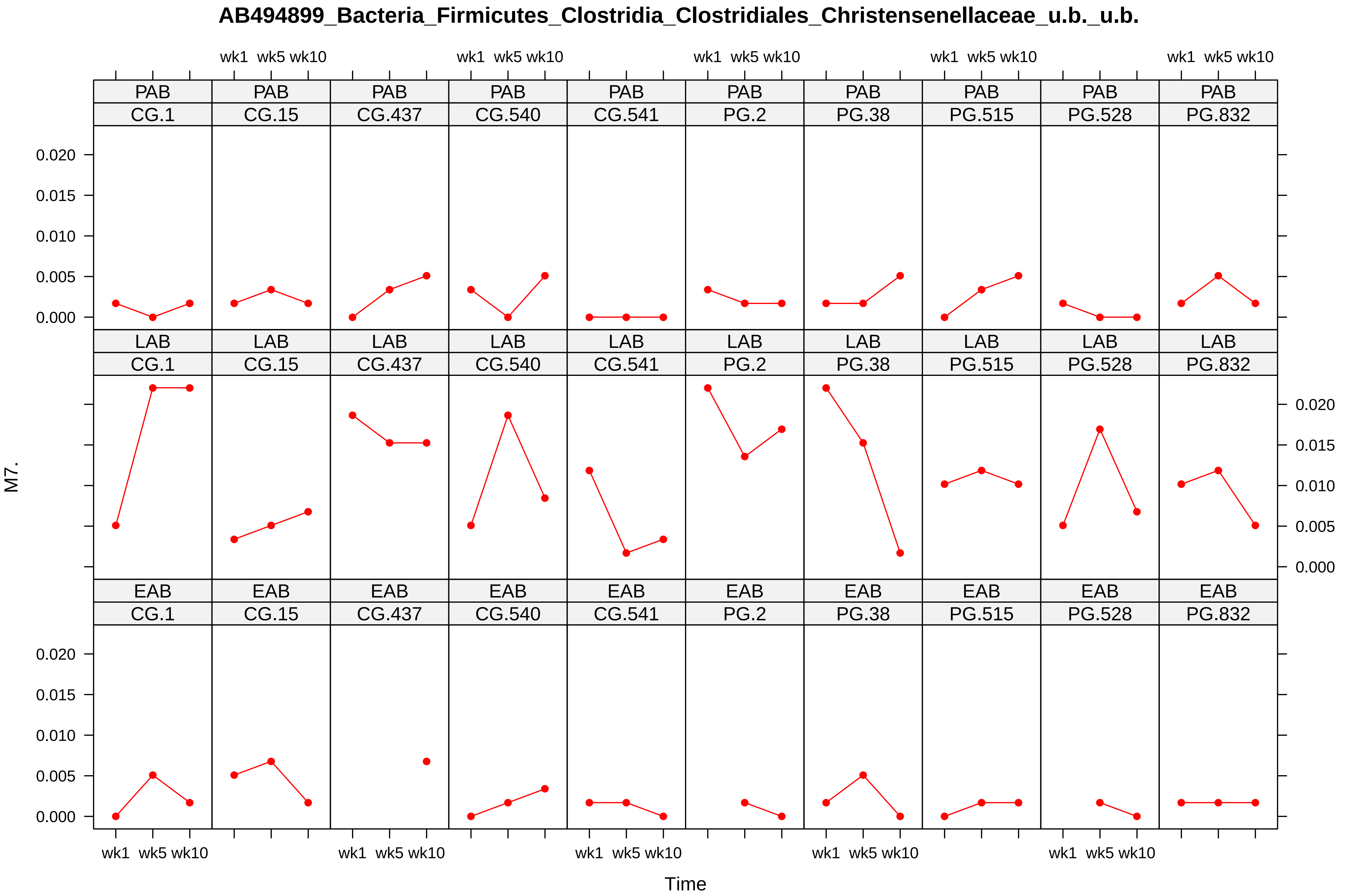
AB494899_Bacteria_Firmicutes_Clostridia_Clostridiales_Christensenellaceae_u.b._u.b.
PAB
LAB
EAB
CG.1
CG.15
CG.437
CG.540
CG.541
PG.2
PG.38
PG.515
PG.528
PG.832
0.000
0.005
0.010
0.015
0.020
wk1 wk5 wk10
M7.
Time

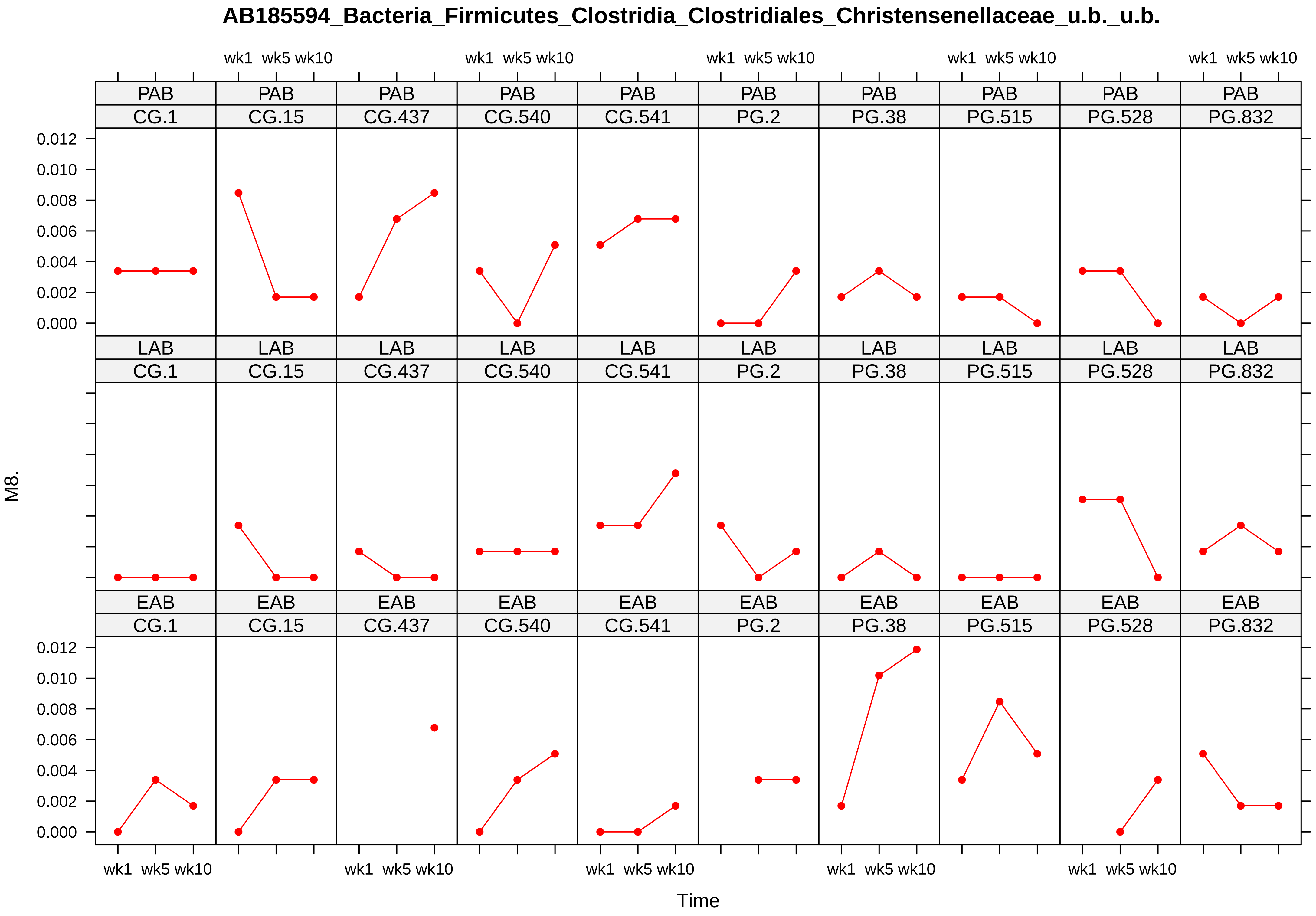
AB185594_Bacteria_Firmicutes_Clostridia_Clostridiales_Christensenellaceae_u.b._u.b.
PAB
LAB
EAB
CG.1
CG.15
CG.437
CG.540
CG.541
PG.2
PG.38
PG.515
PG.528
PG.832
wk1 wk5 wk10
0.000
0.002
0.004
0.006
0.008
0.010
0.012
M8.
Time

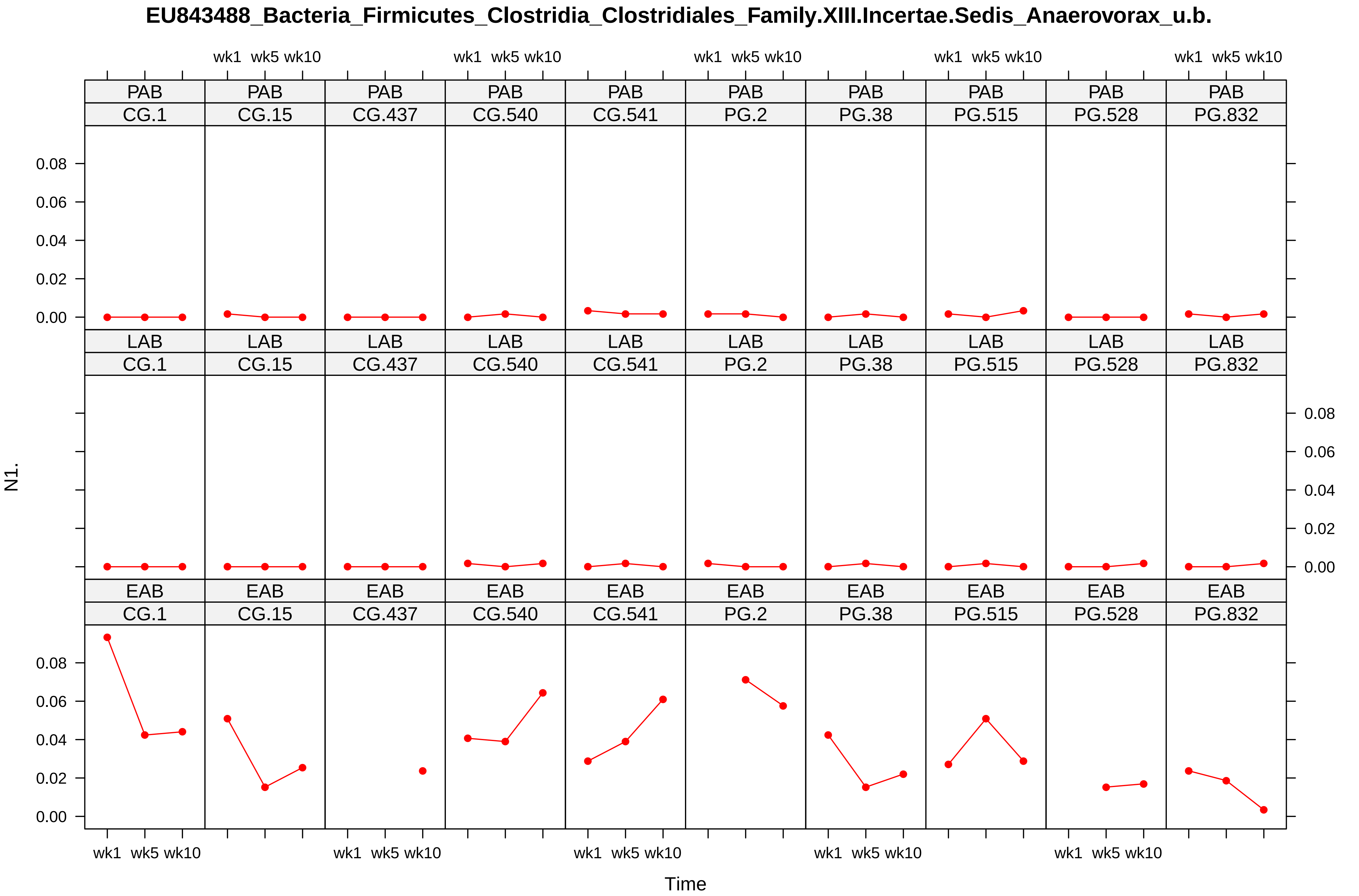
EU843488_Bacteria_Firmicutes_Clostridia_Clostridiales_Family.XIII.Incertae.Sedis_Anaerovorax_u.b.
PAB
LAB
EAB
CG.1
CG.15
CG.437
CG.540
CG.541
PG.2
PG.38
PG.515
PG.528
PG.832
wk1
wk5
wk10
0.00
0.02
0.04
0.06
0.08
N1.
Time

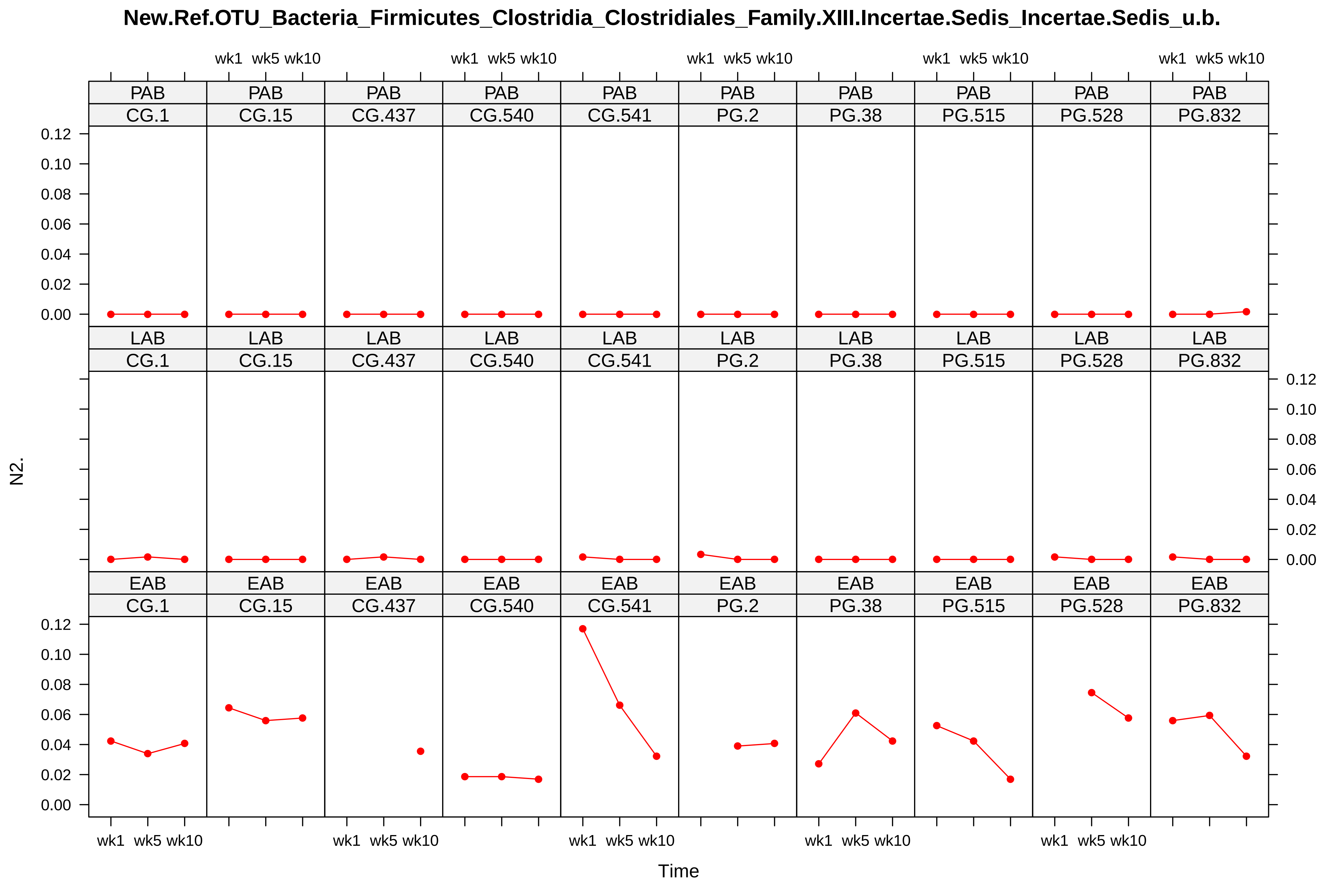
New.Ref.OTU_Bacteria_Firmicutes_Clostridia_Clostridiales_Family.XIII.Incertae.Sedis_Incertae.Sedis_u.b.
PAB
LAB
EAB
CG.1
CG.15
CG.437
CG.540
CG.541
PG.2
PG.38
PG.515
PG.528
PG.832
wk1 wk5 wk10
0.00
0.02
0.04
0.06
0.08
0.10
0.12
N2.
Time

EU842492_Bacteria_Firmicutes_Clostridia_Clostridiales_Family.XIII.Incertae.Sedis_Incertae.Sedis_u.b.

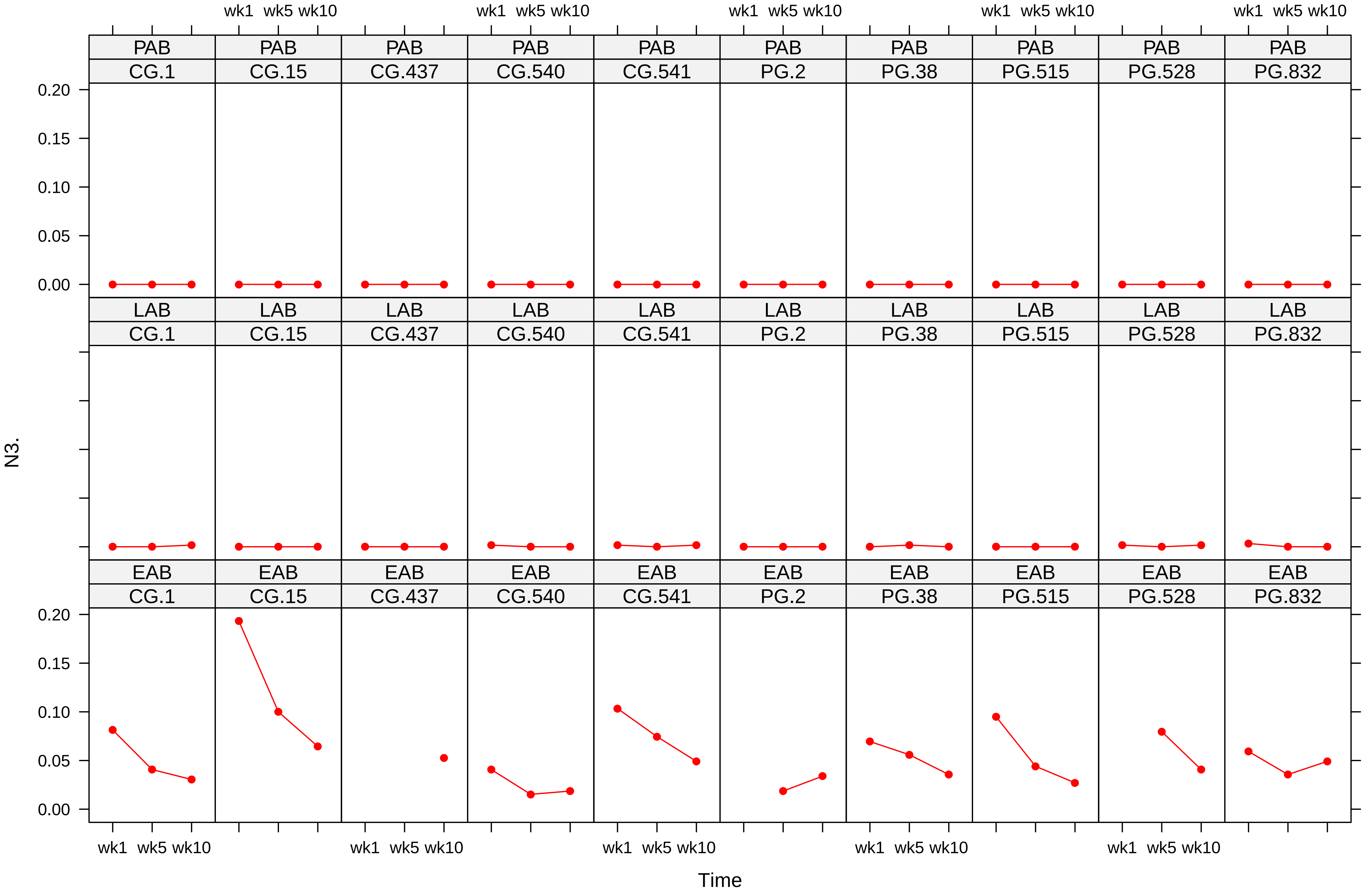

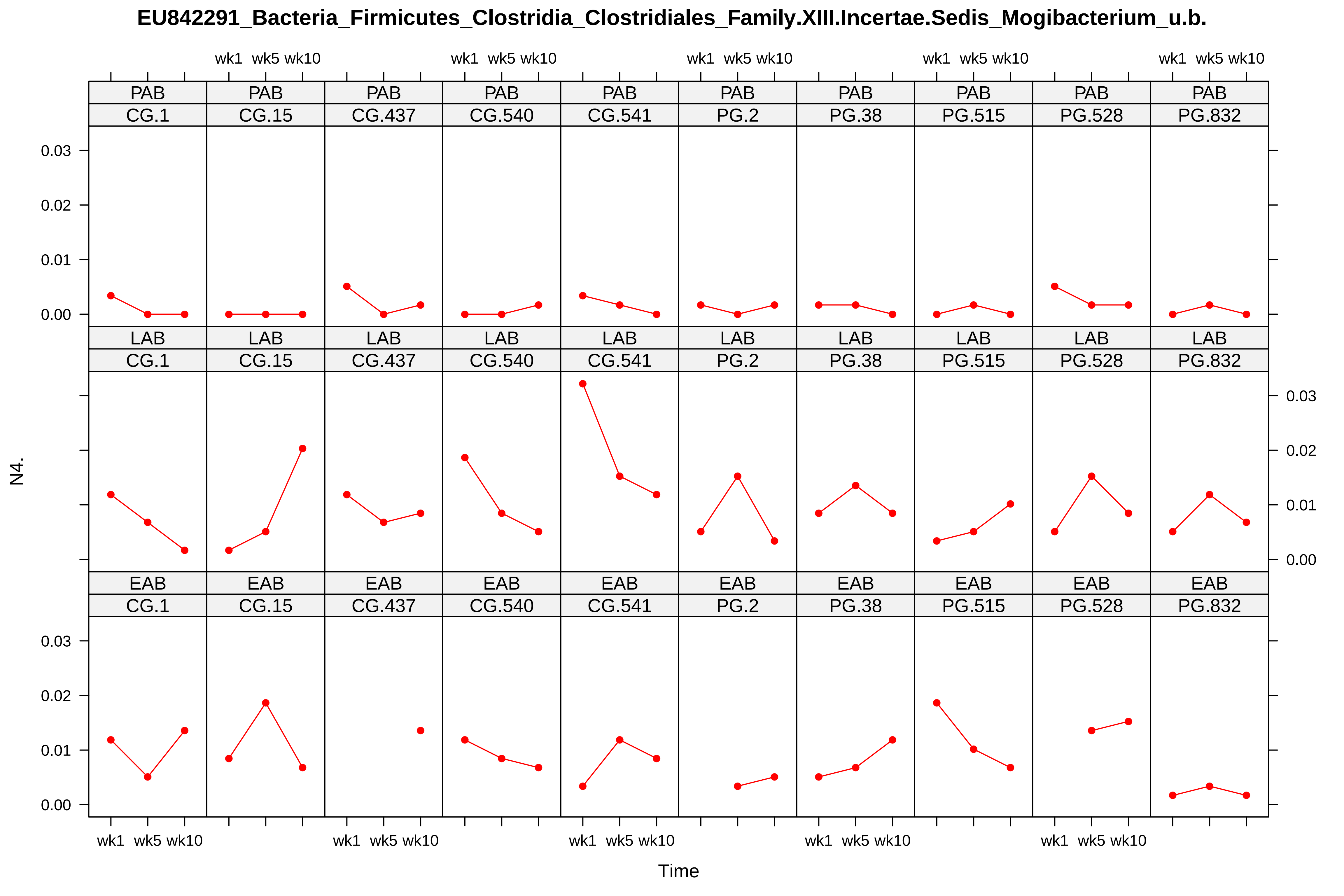
EU842291_Bacteria_Firmicutes_Clostridia_Clostridiales_Family.XIII.Incertae.Sedis_Mogibacterium_u.b.
PAB
LAB
EAB
CG.1
CG.15
CG.437
CG.540
CG.541
PG.2
PG.38
PG.515
PG.528
PG.832
wk1 wk5 wk10
0.00
0.01
0.02
0.03
N4.
Time

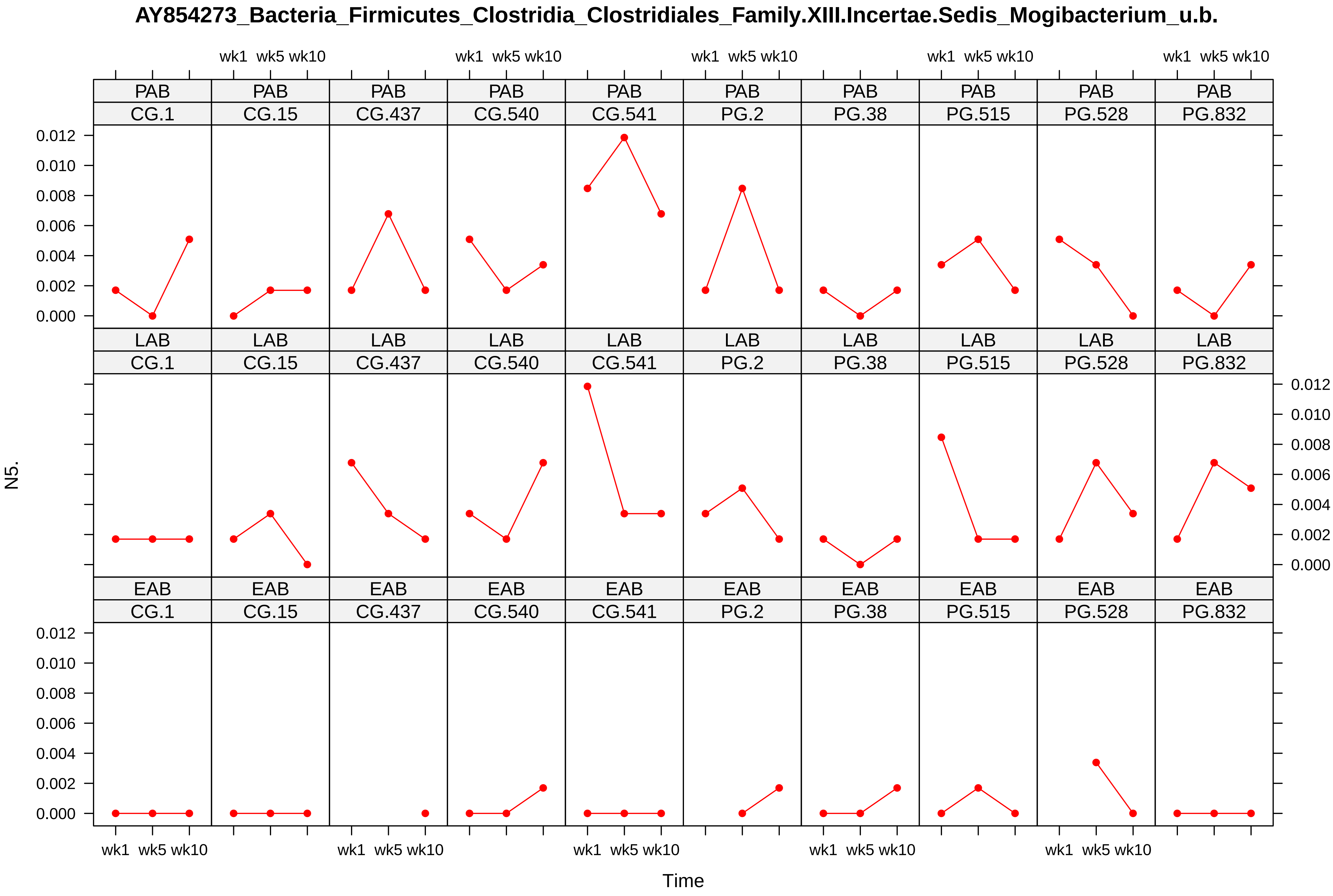
AY854273_Bacteria_Firmicutes_Clostridia_Clostridiales_Family.XIII.Incertae.Sedis_Mogibacterium_u.b.
PAB
LAB
EAB
CG.1
CG.15
CG.437
CG.540
CG.541
PG.2
PG.38
PG.515
PG.528
PG.832
wk1 wk5 wk10
0.000
0.002
0.004
0.006
0.008
0.010
0.012
N5.
Time

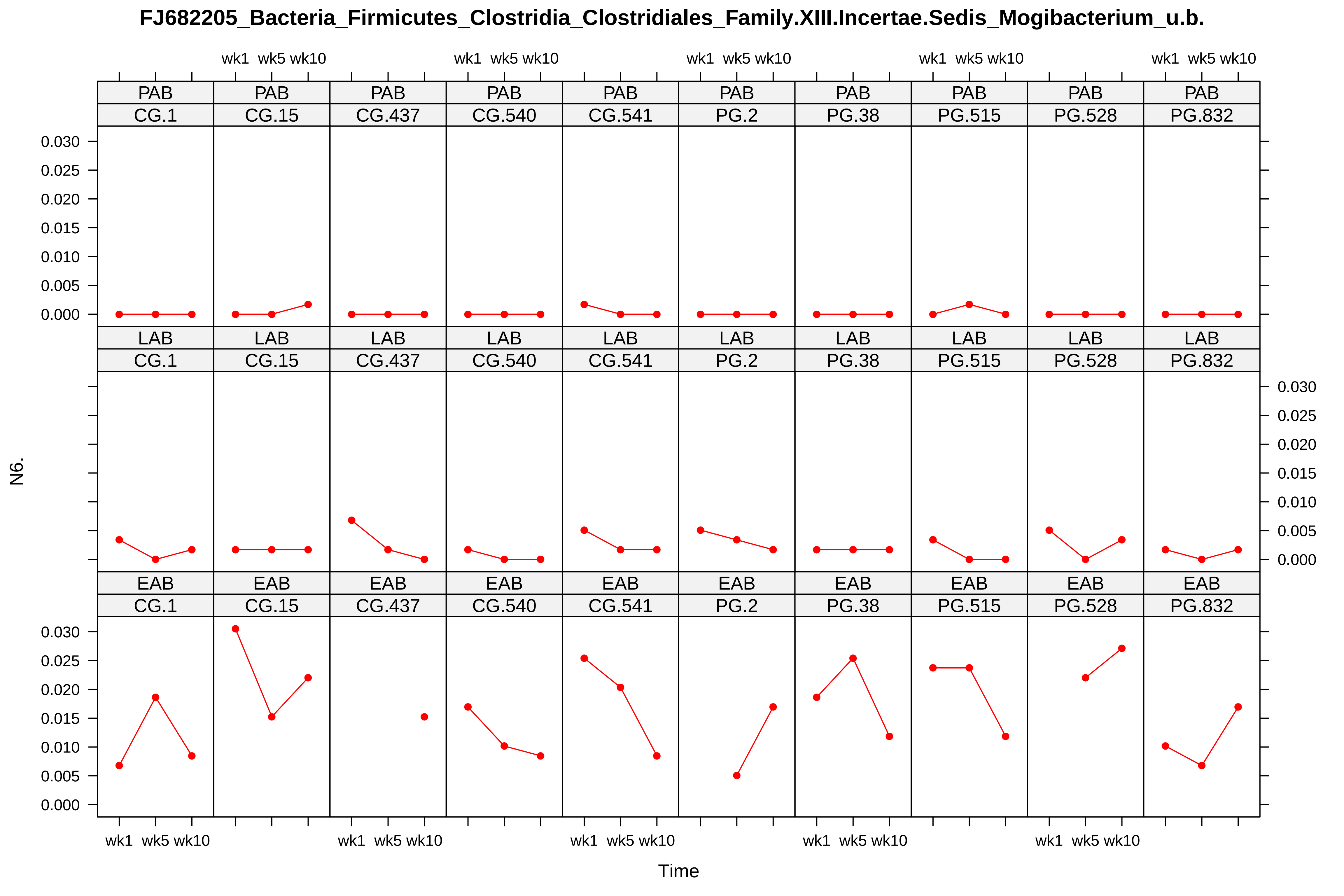
FJ682205_Bacteria_Firmicutes_Clostridia_Clostridiales_Family.XIII.Incertae.Sedis_Mogibacterium_u.b.
PAB
LAB
EAB
CG.1
CG.15
CG.437
CG.540
CG.541
PG.2
PG.38
PG.515
PG.528
PG.832
0.000
0.005
0.010
0.015
0.020
0.025
0.030
wk1 wk5 wk10
N6.
Time

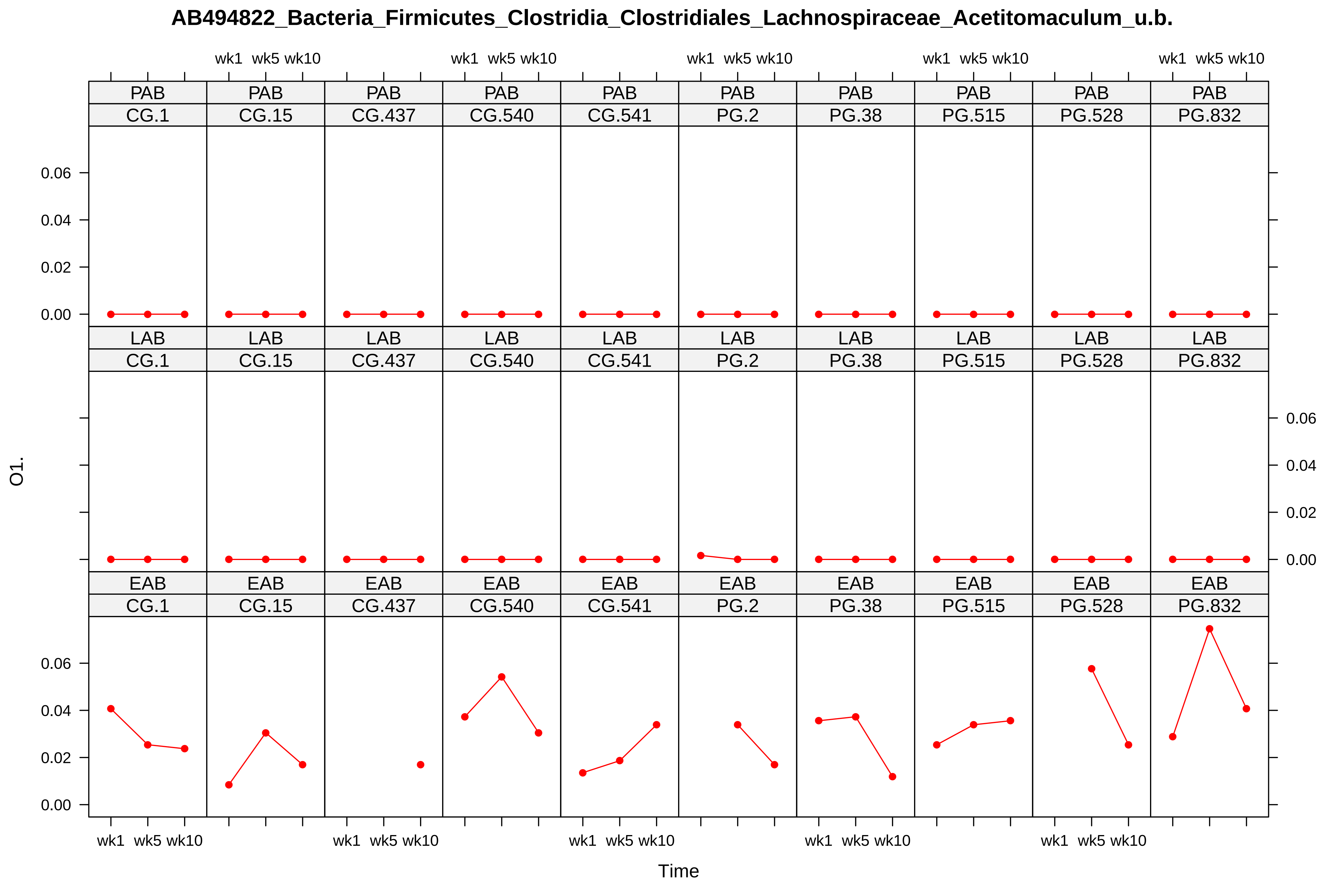
AB494822_Bacteria_Firmicutes_Clostridia_Clostridiales_Lachnospiraceae_Acetitomaculum_u.b.
PAB
LAB
EAB
CG.1
CG.15
CG.437
CG.540
CG.541
PG.2
PG.38
PG.515
PG.528
PG.832
0.00
0.02
0.04
0.06
wk1
wk5
wk10
O1.
Time

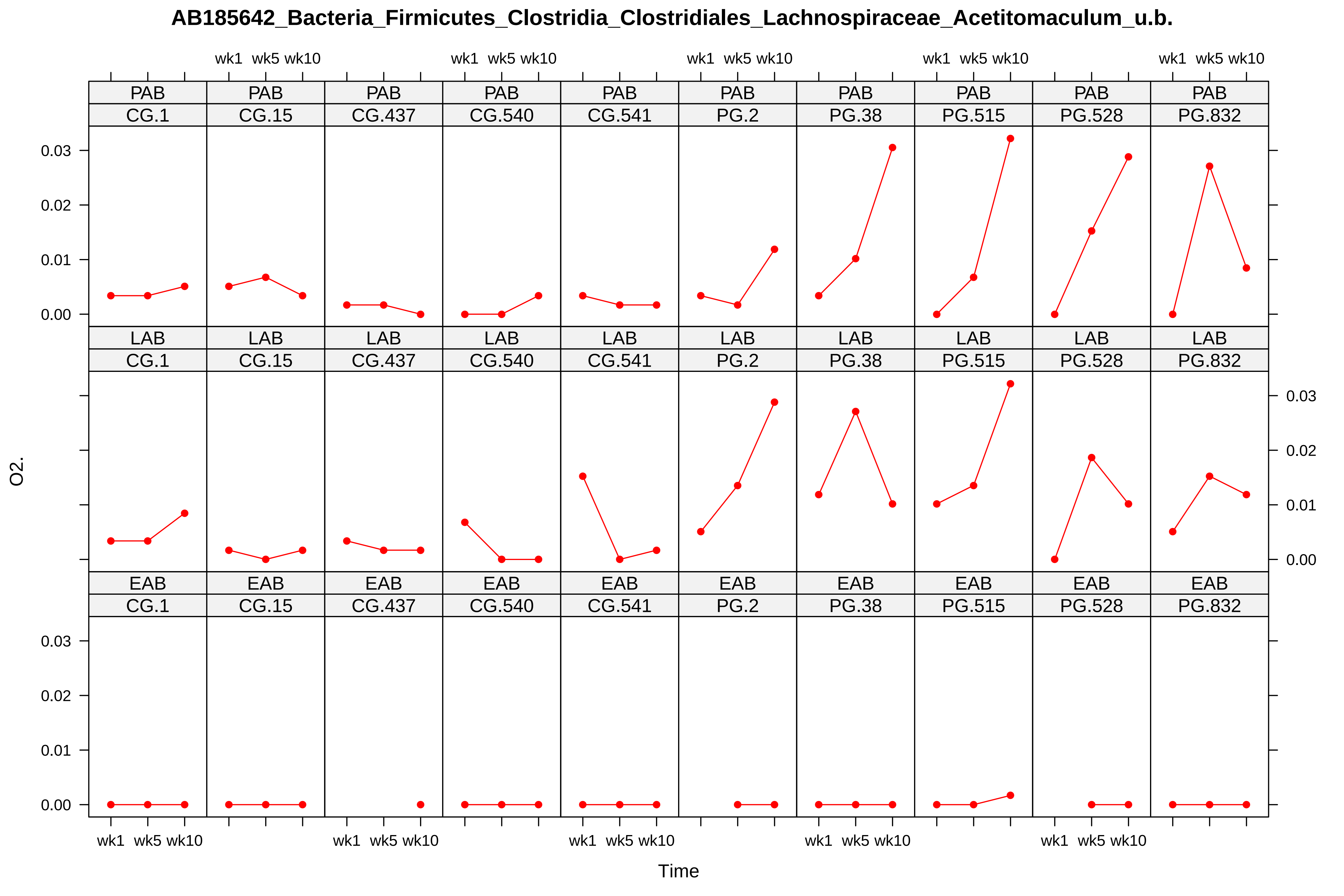
AB185642_Bacteria_Firmicutes_Clostridia_Clostridiales_Lachnospiraceae_Acetitomaculum_u.b.
PAB
LAB
EAB
CG.1
CG.15
CG.437
CG.540
CG.541
PG.2
PG.38
PG.515
PG.528
PG.832
0.00
0.01
0.02
0.03
wk1
wk5
wk10
O2.
Time

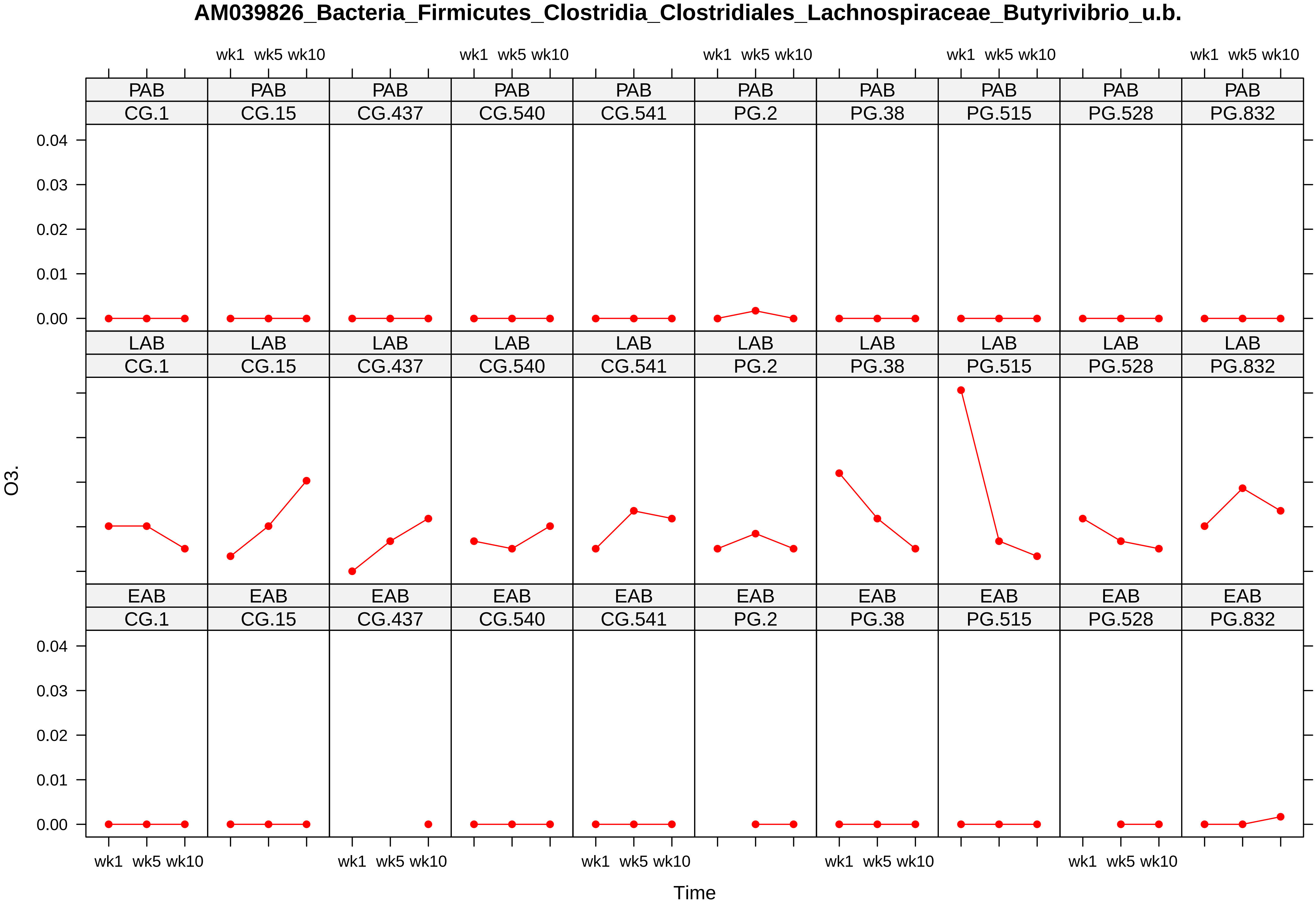
AM039826_Bacteria_Firmicutes_Clostridia_Clostridiales_Lachnospiraceae_Butyrivibrio_u.b.
PAB
LAB
EAB
CG.1
CG.15
CG.437
CG.540
CG.541
PG.2
PG.38
PG.515
PG.528
PG.832
wk1 wk5 wk10
0.00
0.01
0.02
0.03
0.04
O3.
Time

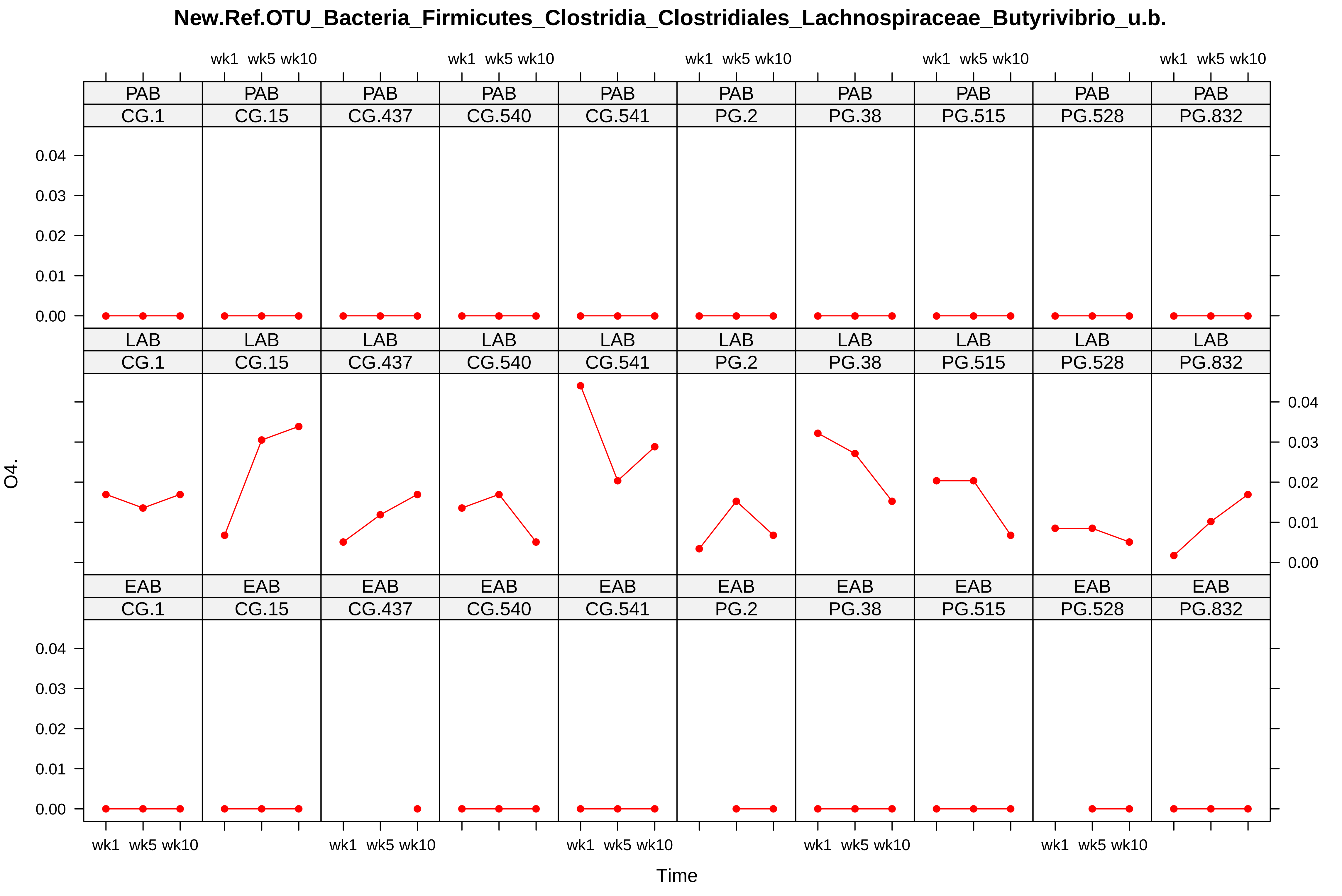

New.Ref.OTU_Bacteria_Firmicutes_Clostridia_Clostridiales_Lachnospiraceae_Butyrivibrio_u.b.
PAB
LAB
EAB
CG.1
CG.15
CG.437
CG.540
CG.541
PG.2
PG.38
PG.515
PG.528
PG.832
wk1 wk5 wk10
0.00
0.01
0.02
0.03
0.04
O4.
Time

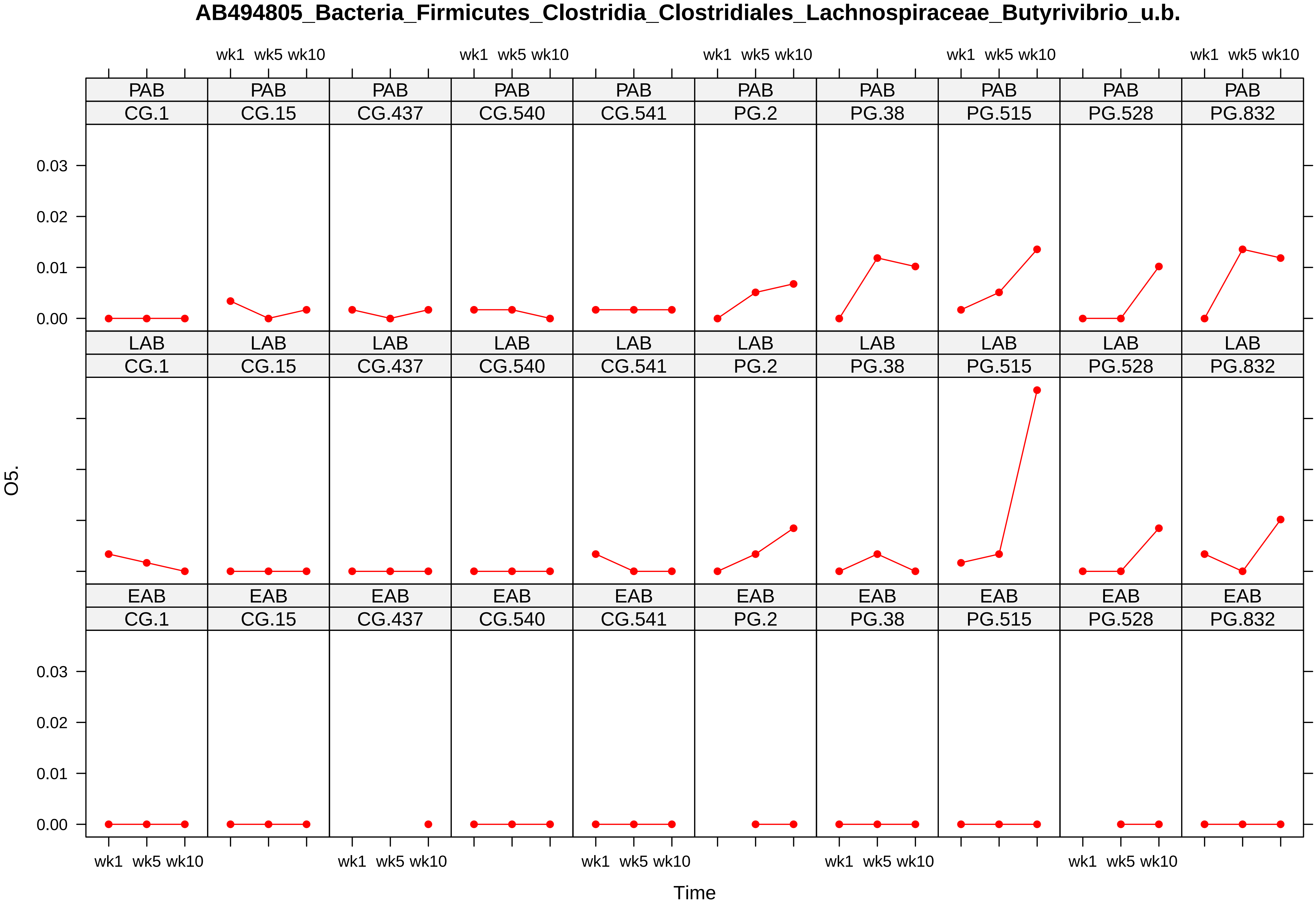

AB494805_Bacteria_Firmicutes_Clostridia_Clostridiales_Lachnospiraceae_Butyrivibrio_u.b.
PAB
LAB
EAB
CG.1
CG.15
CG.437
CG.540
CG.541
PG.2
PG.38
PG.515
PG.528
PG.832
0.00
0.01
0.02
0.03
wk1
wk5
wk10
O5.
Time

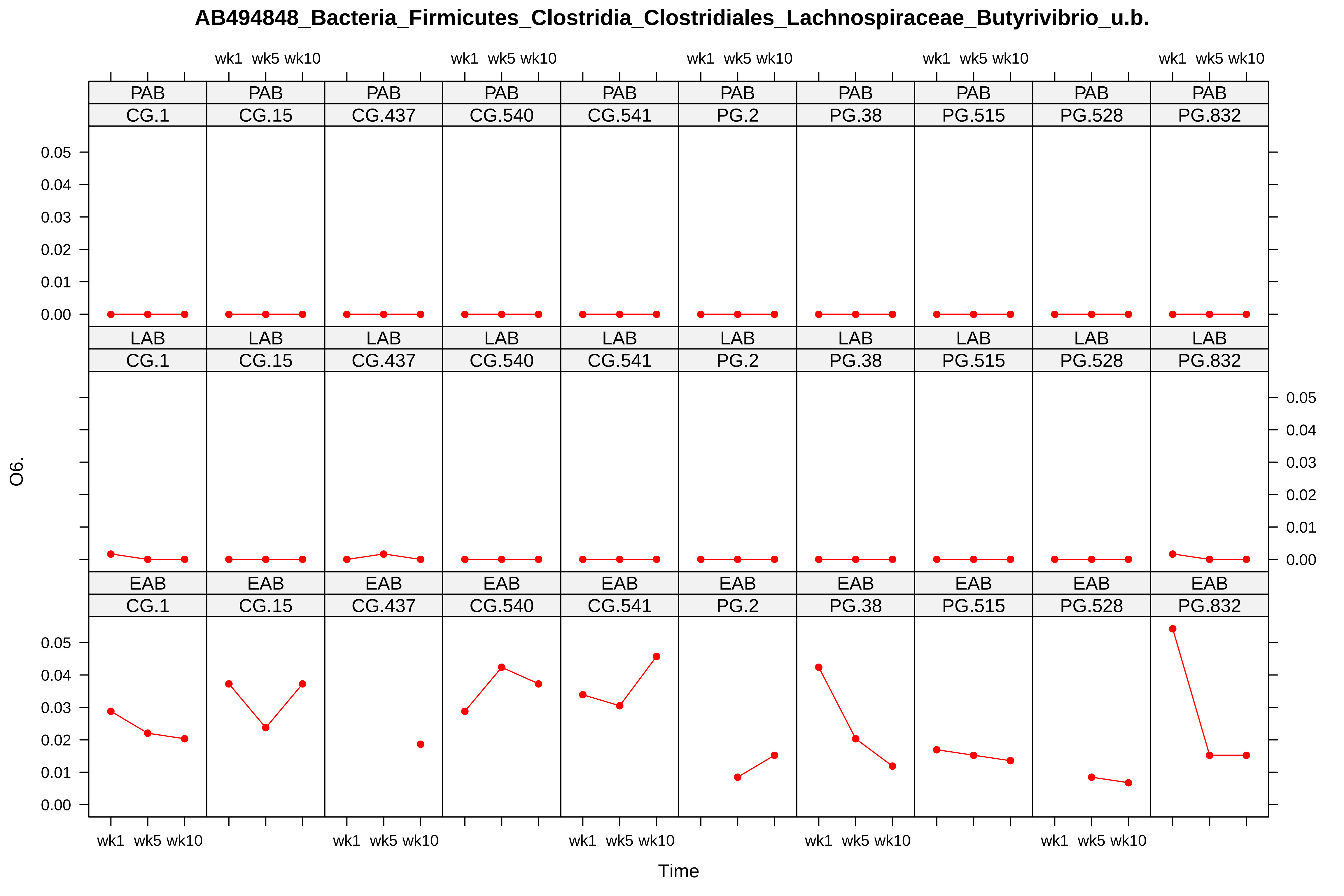

AB494848_Bacteria_Firmicutes_Clostridia_Clostridiales_Lachnospiraceae_Butyrivibrio_u.b.
PAB
LAB
EAB
CG.1
CG.15
CG.437
CG.540
CG.541
PG.2
PG.38
PG.515
PG.528
PG.832
wk1
wk5
wk10
0.00
0.01
0.02
0.03
0.04
0.05
O6.
Time

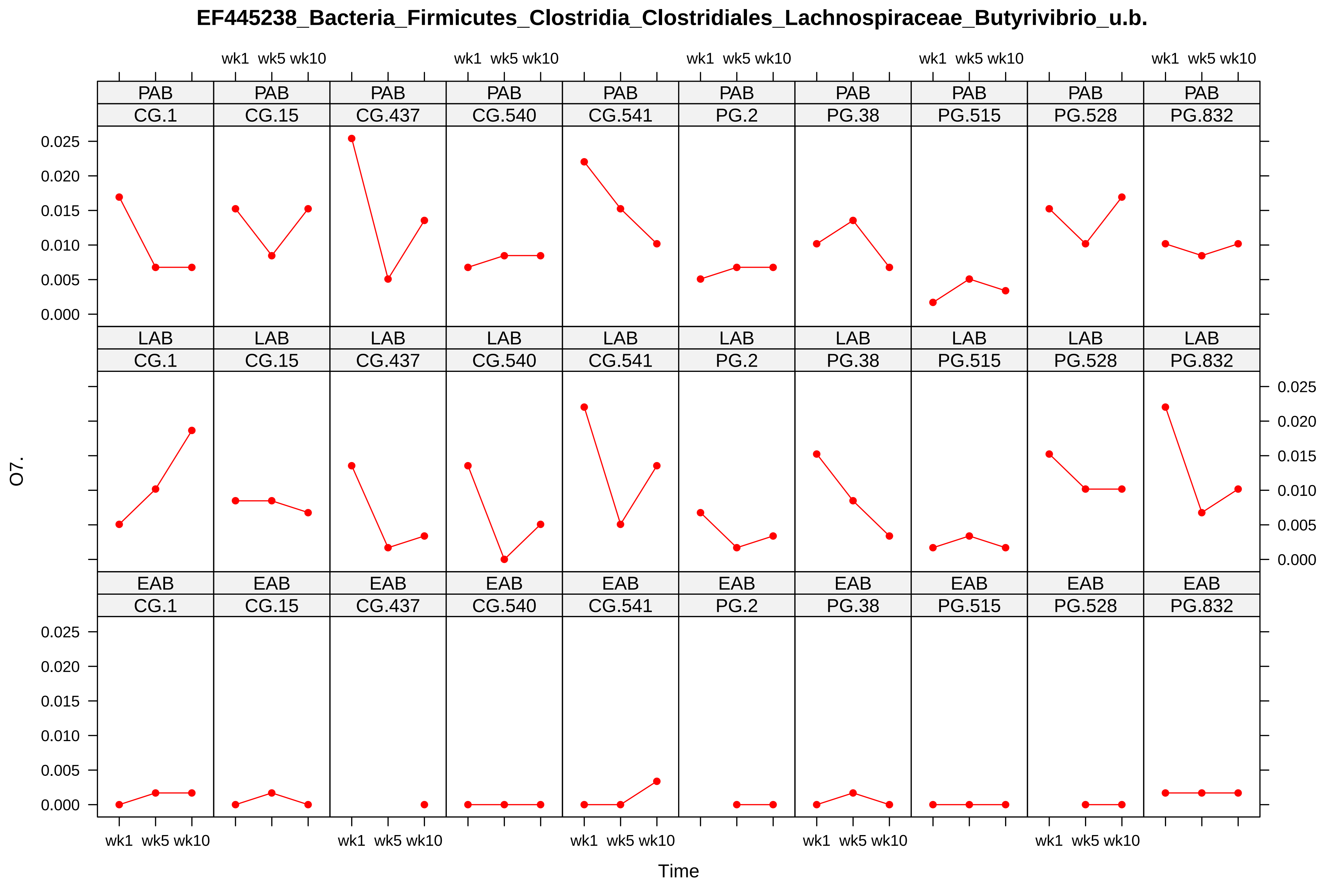

EF445238_Bacteria_Firmicutes_Clostridia_Clostridiales_Lachnospiraceae_Butyrivibrio_u.b.
PAB
LAB
EAB
CG.1
CG.15
CG.437
CG.540
CG.541
PG.2
PG.38
PG.515
PG.528
PG.832
wk1 wk5 wk10
0.000
0.005
0.010
0.015
0.020
0.025
O7.
Time

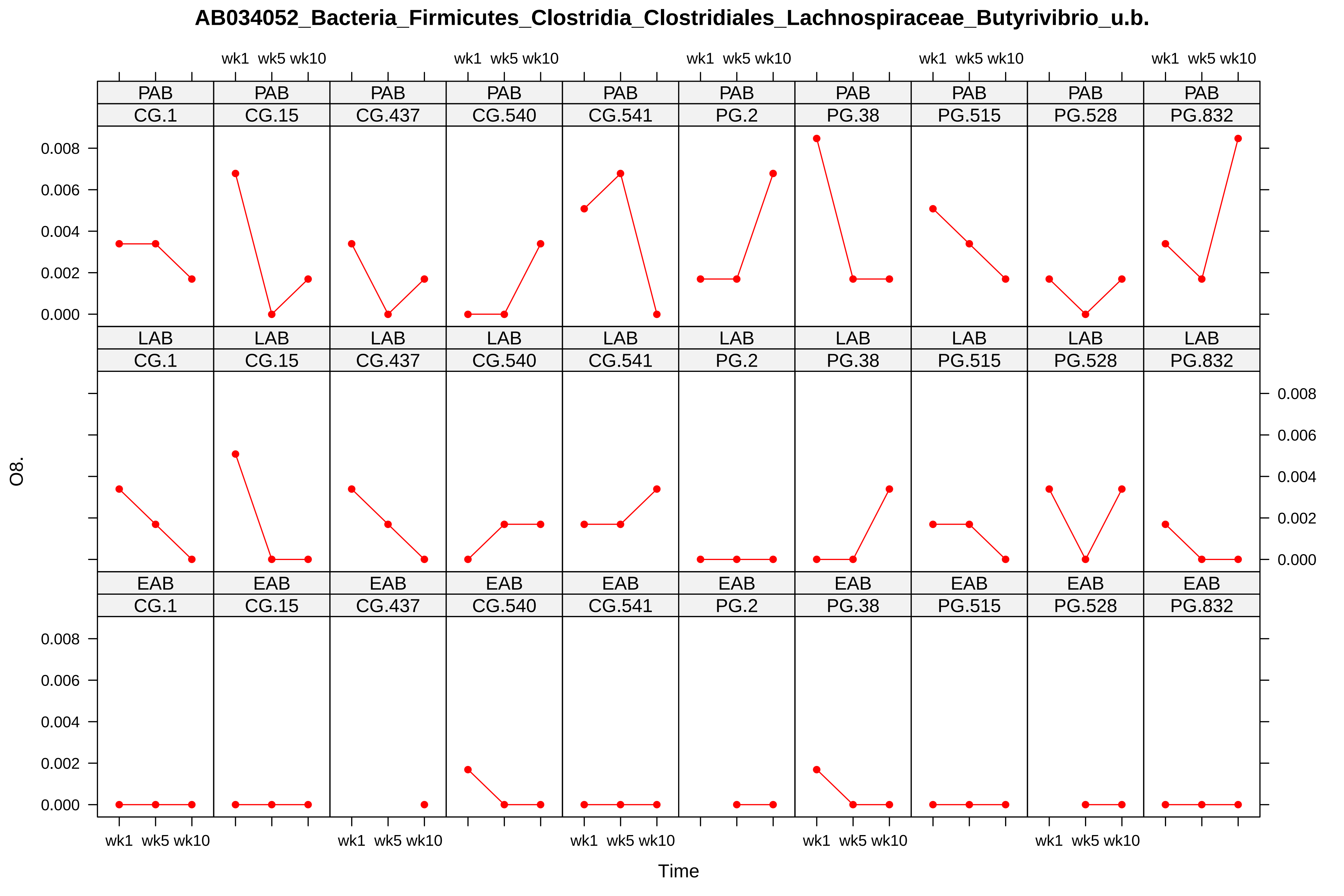
AB034052_Bacteria_Firmicutes_Clostridia_Clostridiales_Lachnospiraceae_Butyrivibrio_u.b.
PAB
LAB
EAB
CG.1
CG.15
CG.437
CG.540
CG.541
PG.2
PG.38
PG.515
PG.528
PG.832
wk1 wk5 wk10
0.000
0.002
0.004
0.006
0.008
O8.
Time

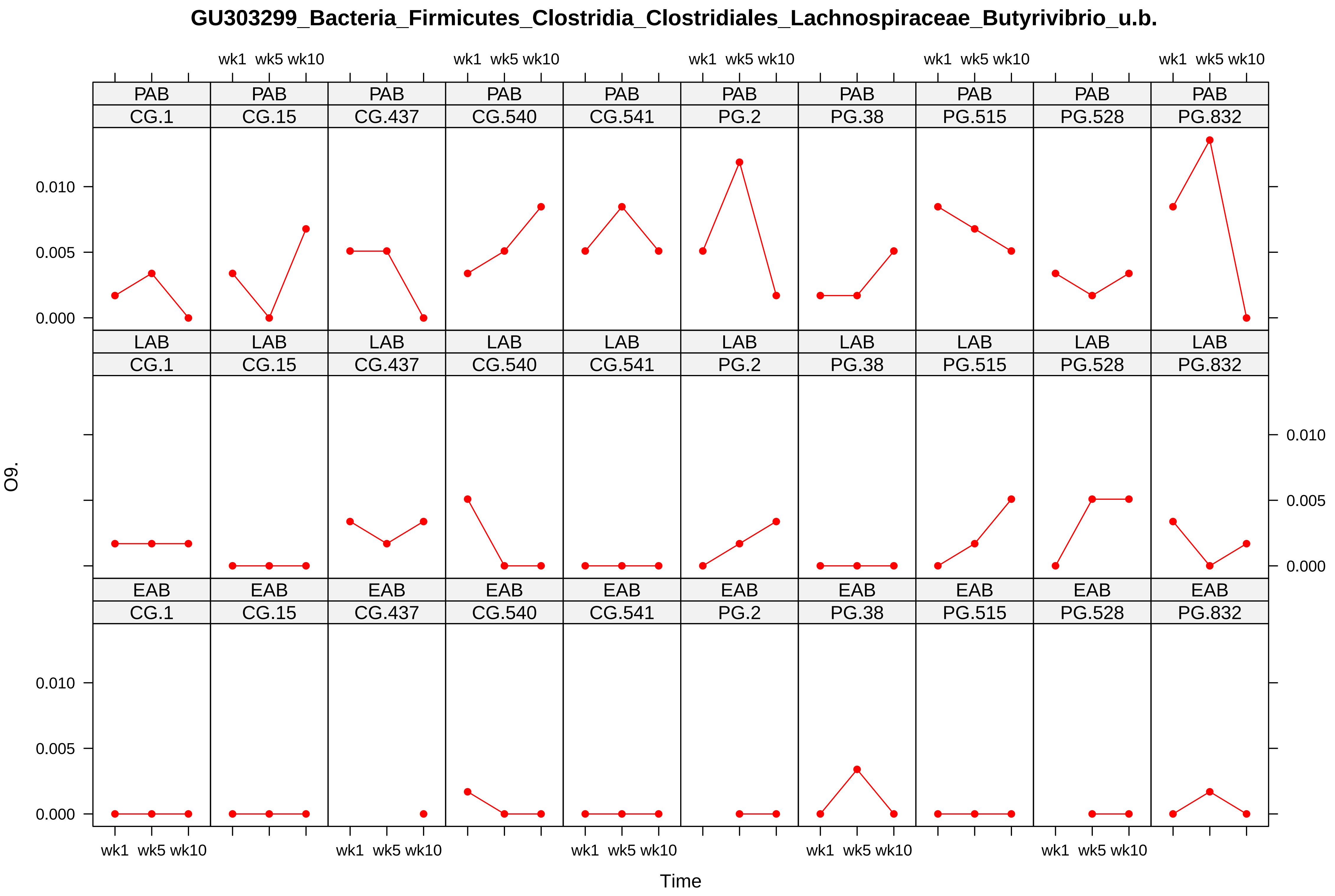
GU303299_Bacteria_Firmicutes_Clostridia_Clostridiales_Lachnospiraceae_Butyrivibrio_u.b.
PAB
LAB
EAB
CG.1
CG.15
CG.437
CG.540
CG.541
PG.2
PG.38
PG.515
PG.528
PG.832
0.000
0.005
0.010
wk1
wk5
wk10
Time
O9.

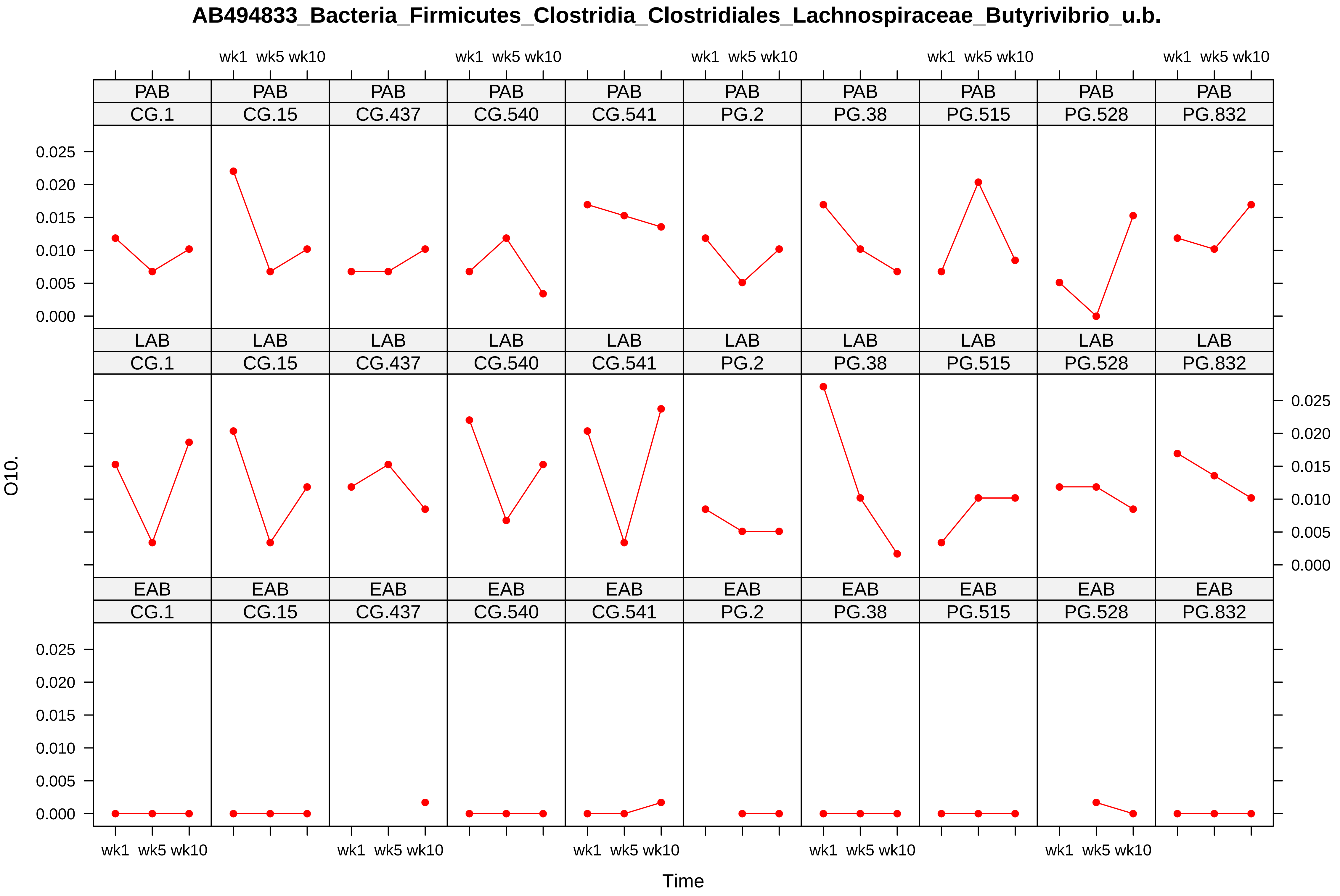

AB494833_Bacteria_Firmicutes_Clostridia_Clostridiales_Lachnospiraceae_Butyrivibrio_u.b.
PAB
LAB
EAB
CG.1
CG.15
CG.437
CG.540
CG.541
PG.2
PG.38
PG.515
PG.528
PG.832
0.000
0.005
0.010
0.015
0.020
0.025
wk1
wk5
wk10
O10.
Time

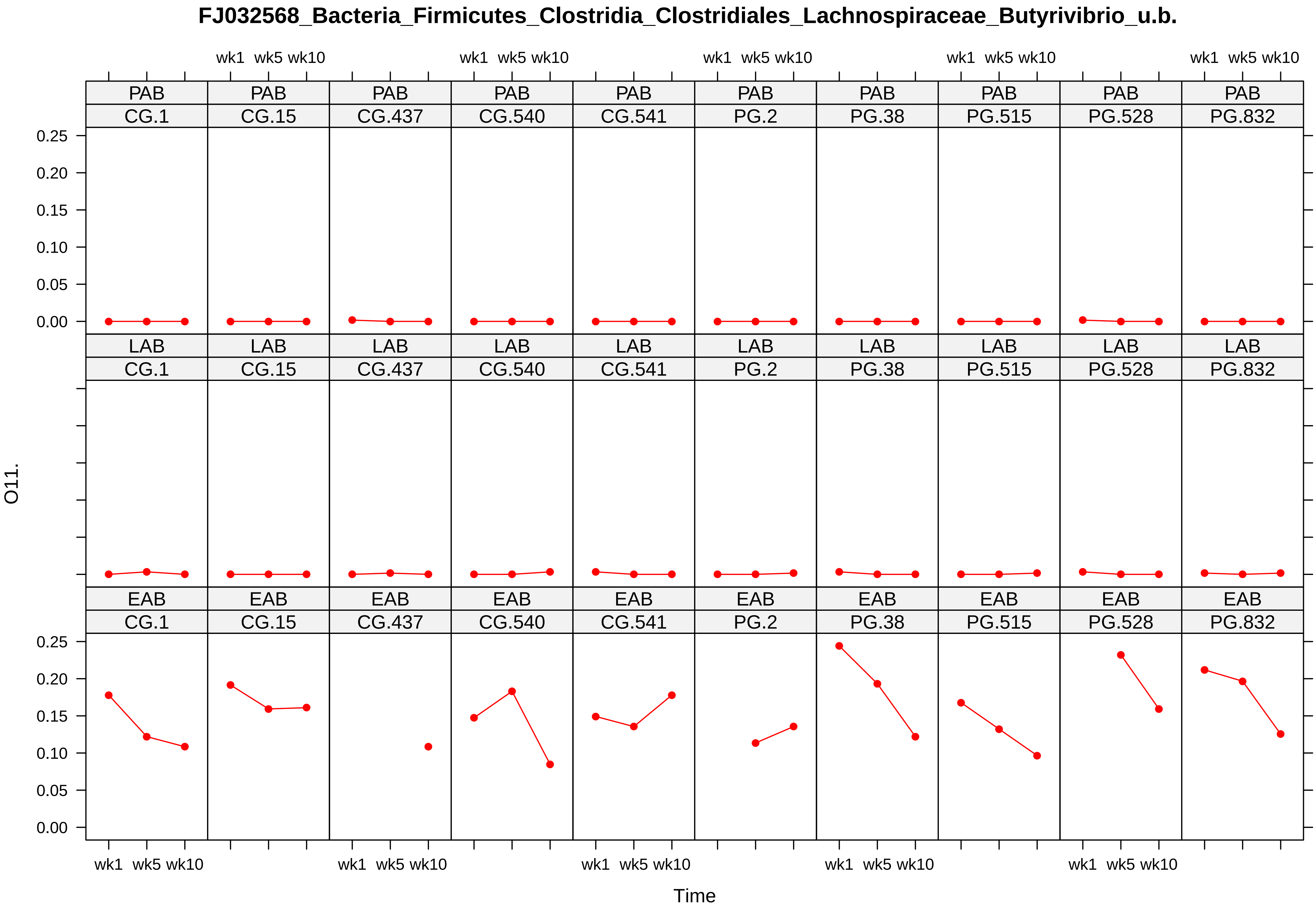
FJ032568_Bacteria_Firmicutes_Clostridia_Clostridiales_Lachnospiraceae_Butyrivibrio_u.b.
PAB
LAB
EAB
CG.1
CG.15
CG.437
CG.540
CG.541
PG.2
PG.38
PG.515
PG.528
PG.832
0.00
0.05
0.10
0.15
0.20
0.25
wk1
wk5
wk10
O11.
Time

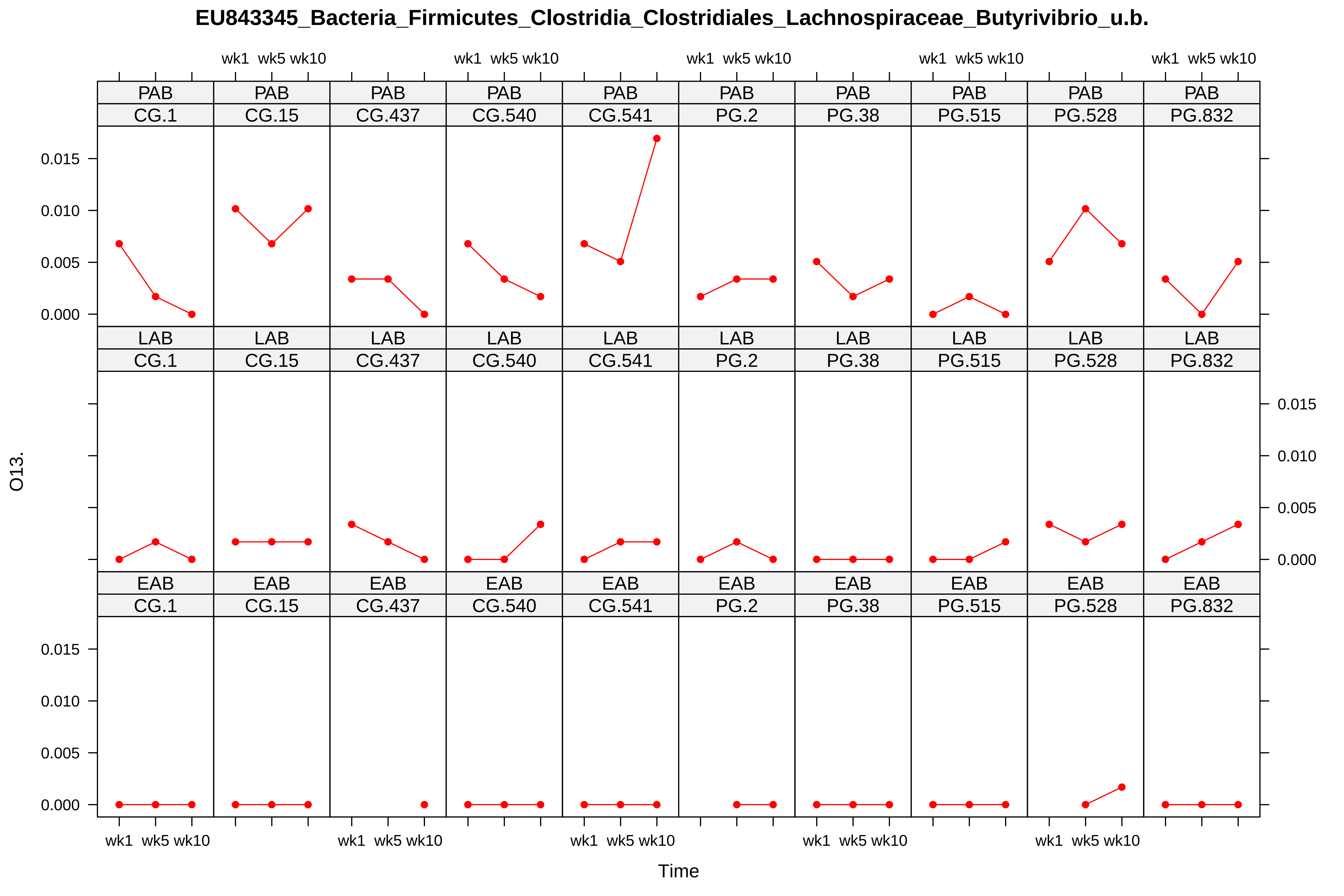
EU843345_Bacteria_Firmicutes_Clostridia_Clostridiales_Lachnospiraceae_Butyrivibrio_u.b.
PAB
LAB
EAB
CG.1
CG.15
CG.437
CG.540
CG.541
PG.2
PG.38
PG.515
PG.528
PG.832
0.000
0.005
0.010
0.015
wk1 wk5 wk10
O13.
Time

GU124460_Bacteria_Firmicutes_Clostridia_Clostridiales_Lachnospiraceae_Incertae.Sedis_u.b.

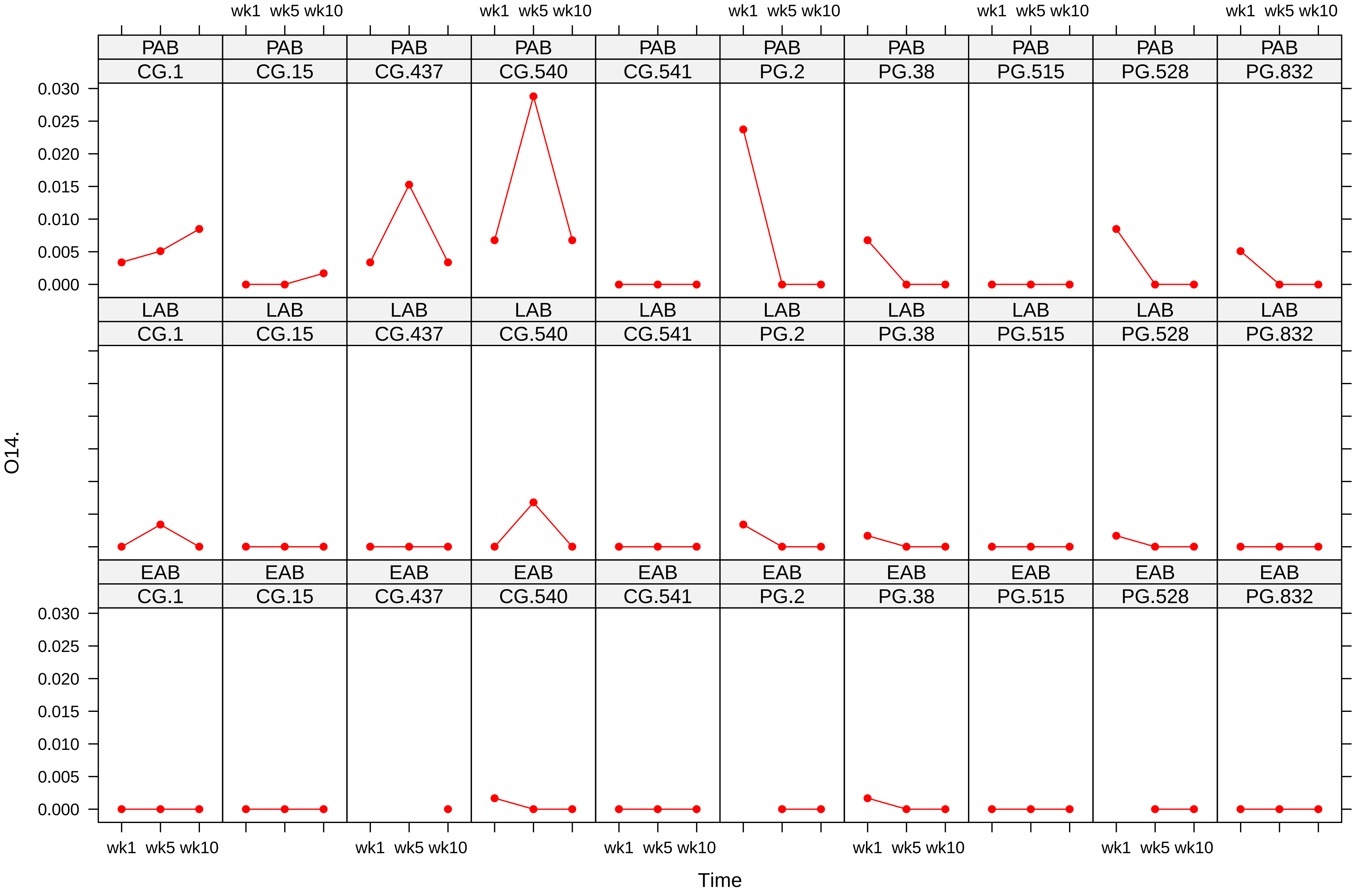

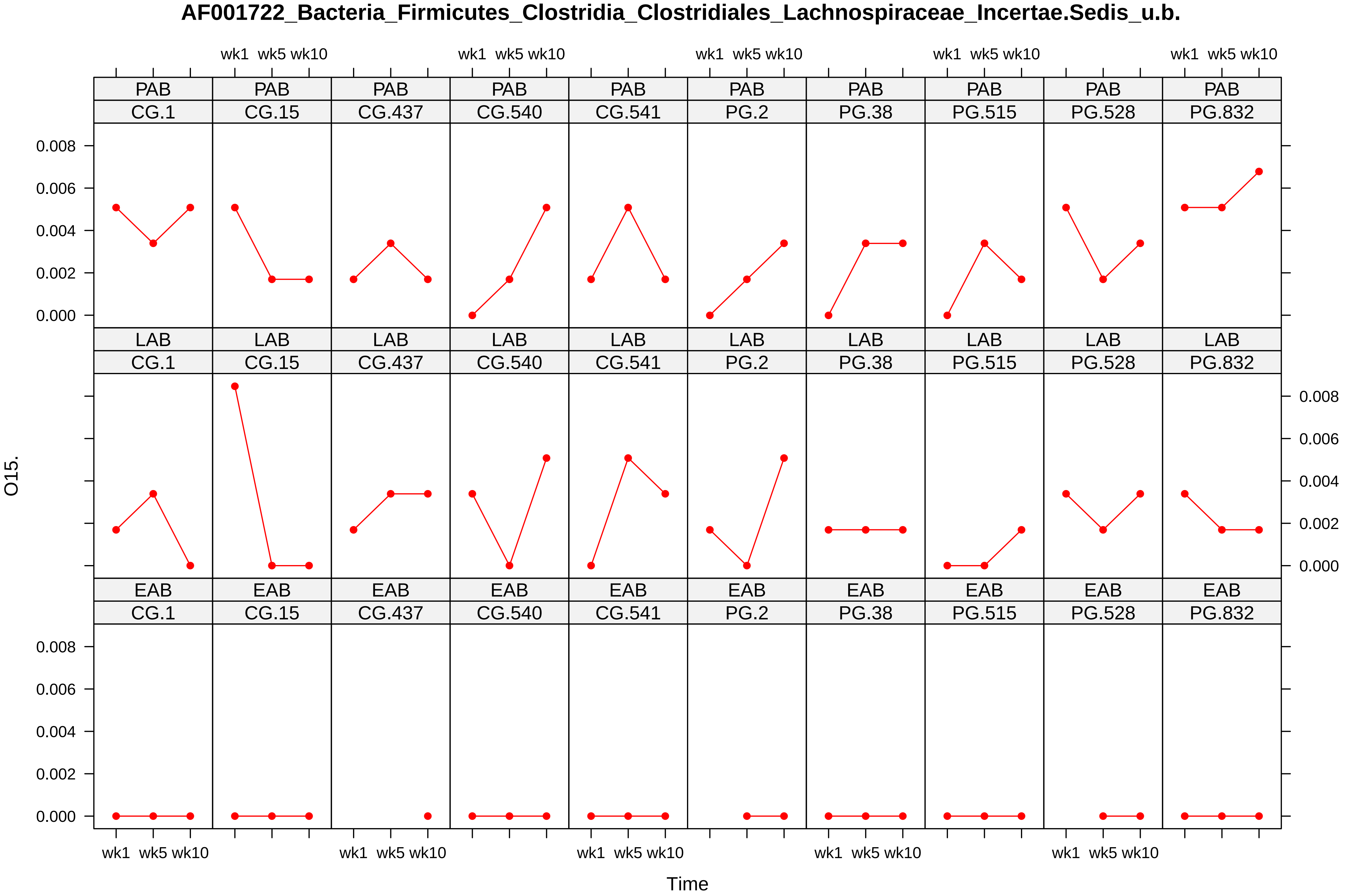

AF001722_Bacteria_Firmicutes_Clostridia_Clostridiales_Lachnospiraceae_Incertae.Sedis_u.b.
PAB
LAB
EAB
CG.1
CG.15
CG.437
CG.540
CG.541
PG.2
PG.38
PG.515
PG.528
PG.832
0.000
0.002
0.004
0.006
0.008
wk1 wk5 wk10
O15.
Time

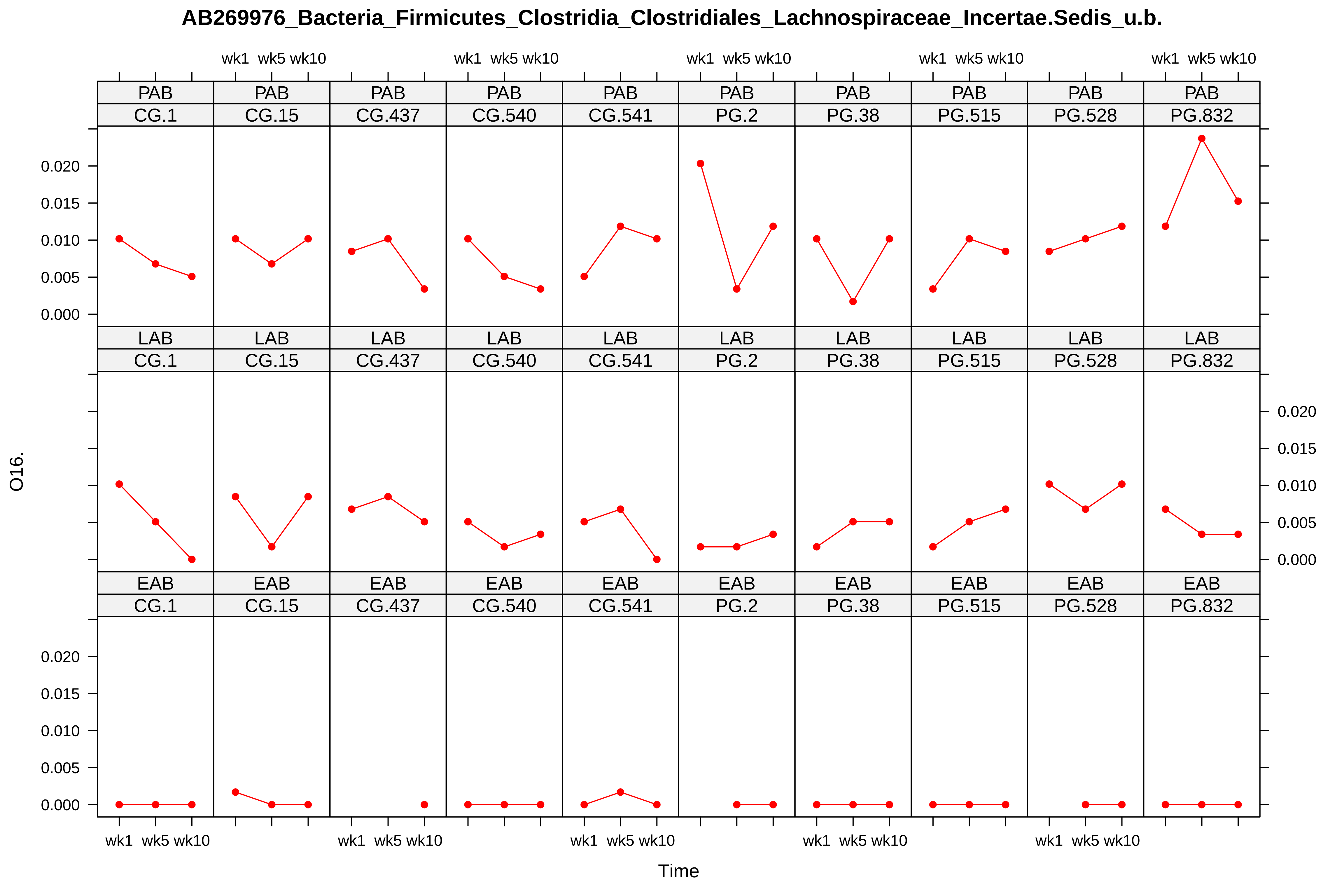
AB269976_Bacteria_Firmicutes_Clostridia_Clostridiales_Lachnospiraceae_Incertae.Sedis_u.b.
PAB
LAB
EAB
CG.1
CG.15
CG.437
CG.540
CG.541
PG.2
PG.38
PG.515
PG.528
PG.832
wk1 wk5 wk10
0.000
0.005
0.010
0.015
0.020
O16.
Time

EU381578_Bacteria_Firmicutes_Clostridia_Clostridiales_Lachnospiraceae_Incertae.Sedis_u.b.

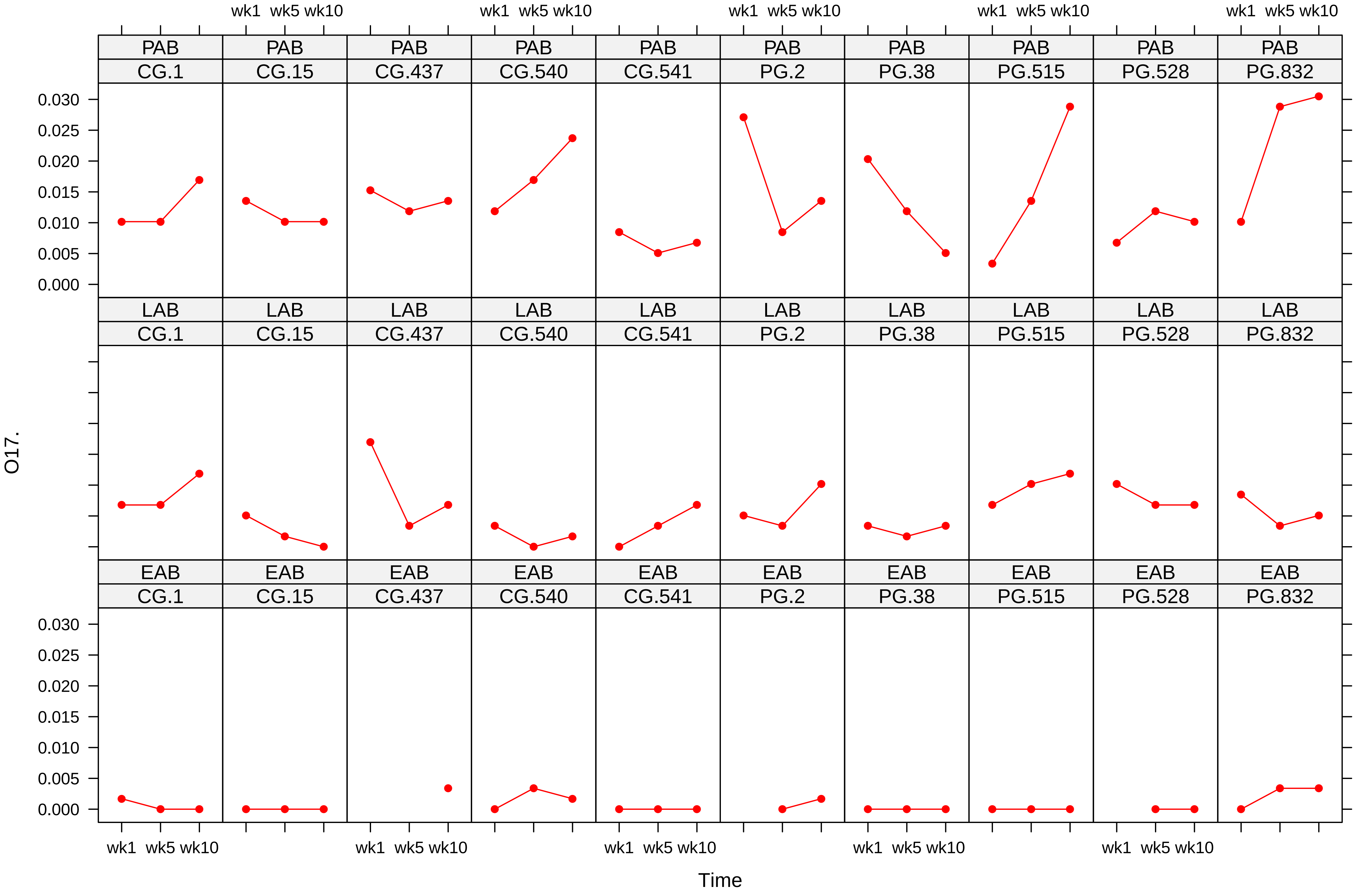

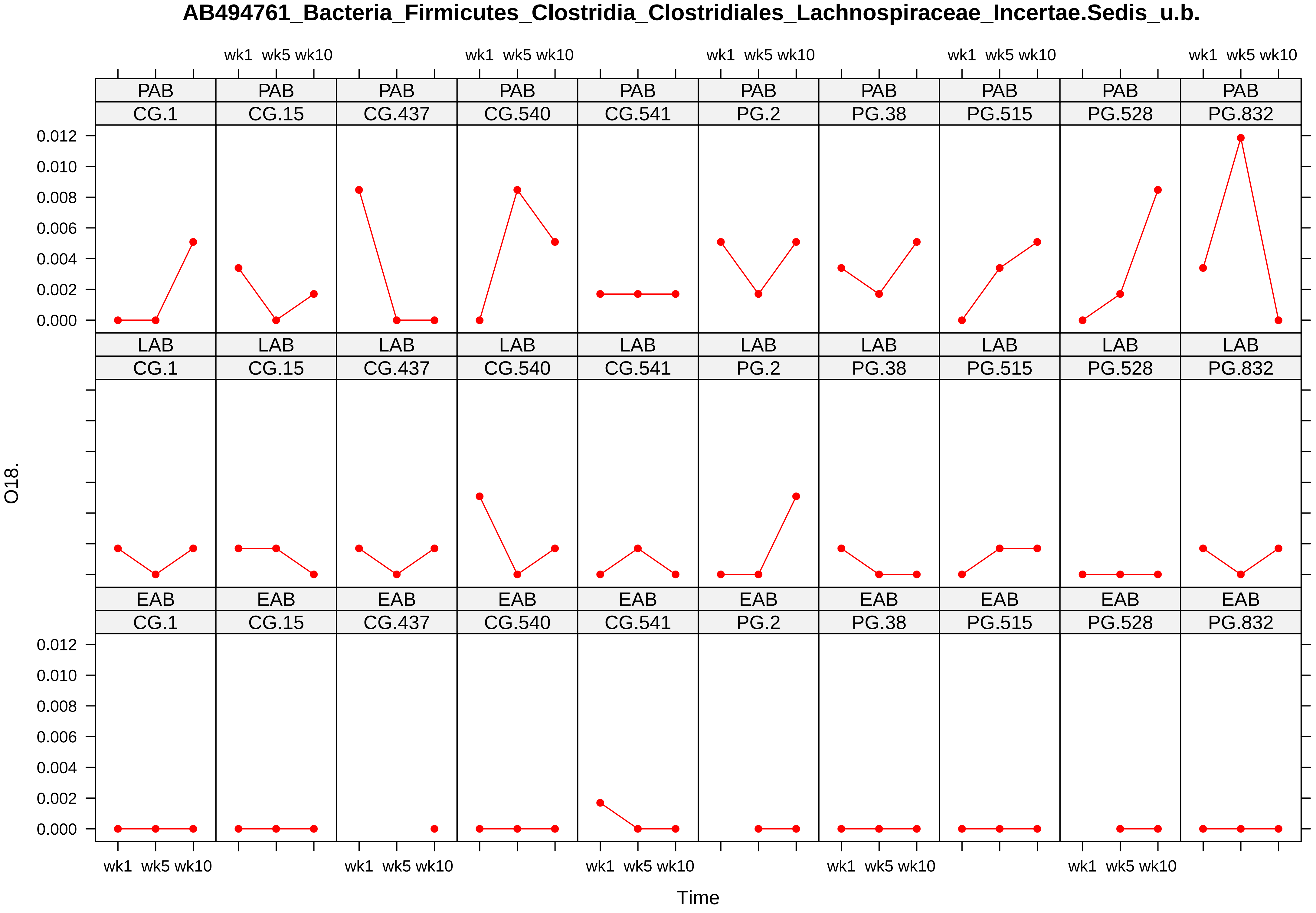
AB494761_Bacteria_Firmicutes_Clostridia_Clostridiales_Lachnospiraceae_Incertae.Sedis_u.b.
PAB
LAB
EAB
CG.1
CG.15
CG.437
CG.540
CG.541
PG.2
PG.38
PG.515
PG.528
PG.832
wk1
wk5
wk10
0.000
0.002
0.004
0.006
0.008
0.010
0.012
O18.
Time

New.Ref.OTU_Bacteria_Firmicutes_Clostridia_Clostridiales_Lachnospiraceae_Incertae.Sedis_u.b.

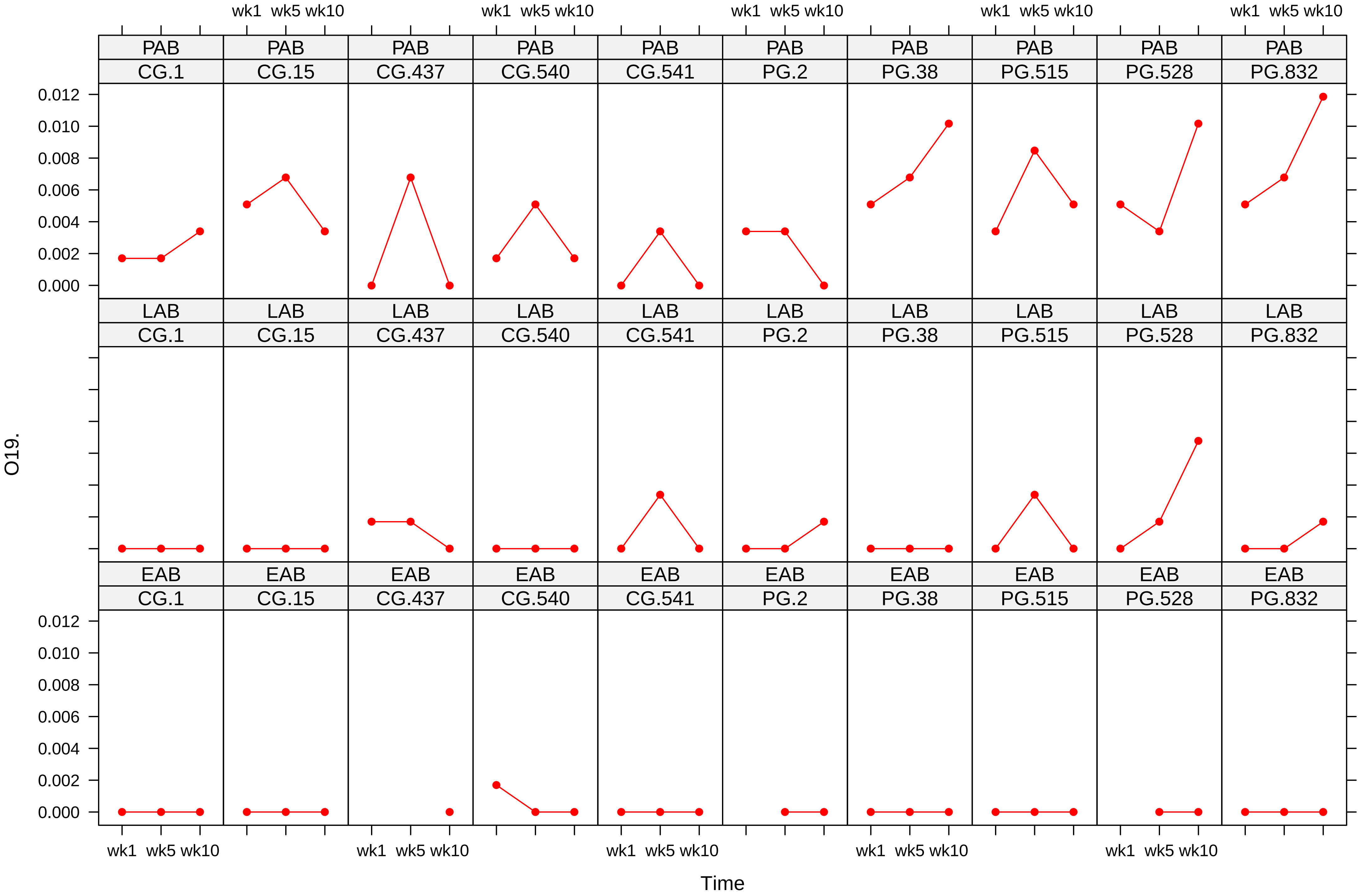

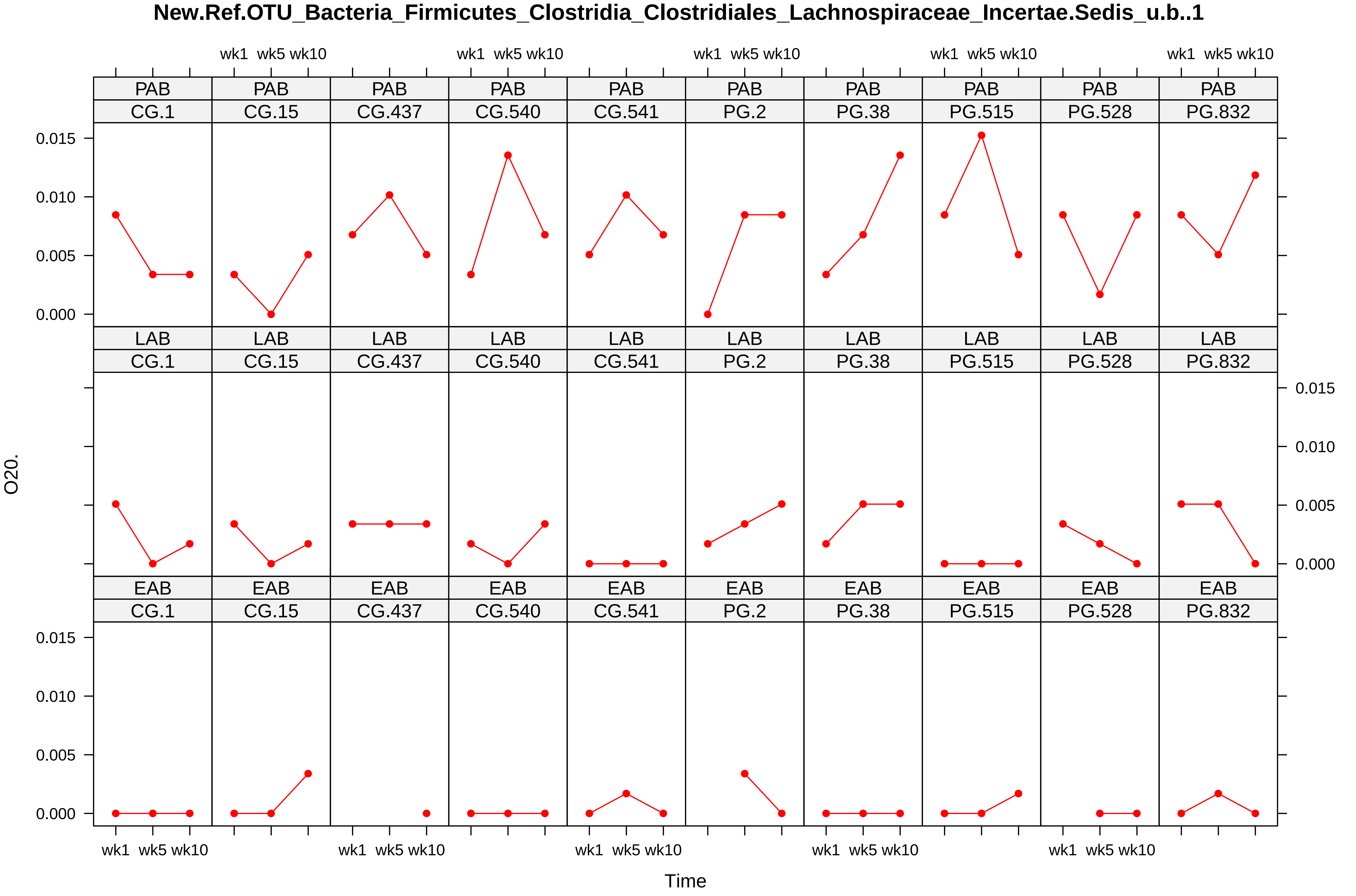

New.Ref.OTU_Bacteria_Firmicutes_Clostridia_Clostridiales_Lachnospiraceae_Incertae.Sedis_u.b..1
PAB
LAB
EAB
CG.1
CG.15
CG.437
CG.540
CG.541
PG.2
PG.38
PG.515
PG.528
PG.832
0.000
0.005
0.010
0.015
wk1 wk5 wk10
O20.
Time

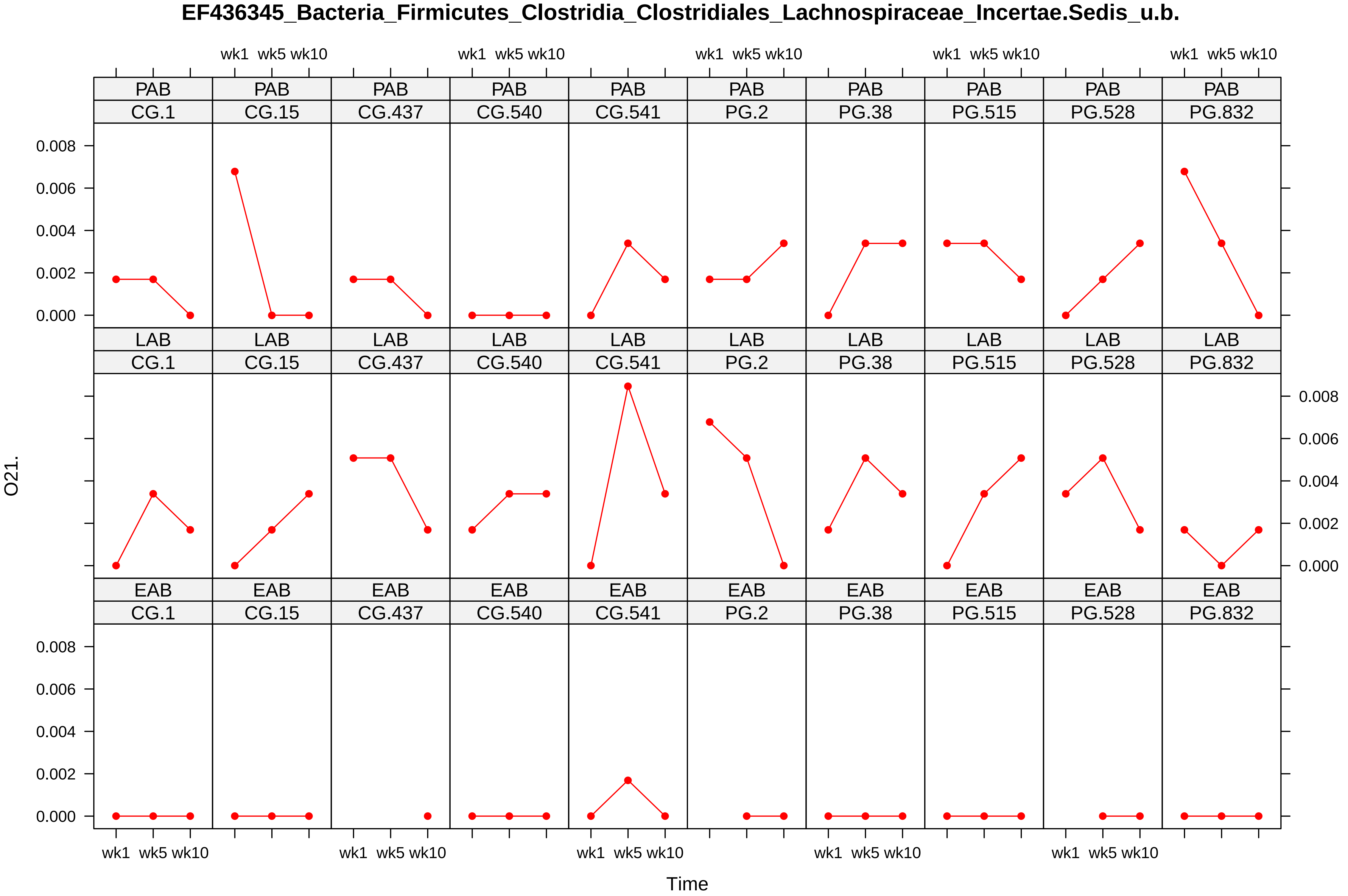
EF436345_Bacteria_Firmicutes_Clostridia_Clostridiales_Lachnospiraceae_Incertae.Sedis_u.b.
PAB
LAB
EAB
CG.1
CG.15
CG.437
CG.540
CG.541
PG.2
PG.38
PG.515
PG.528
PG.832
0.000
0.002
0.004
0.006
0.008
wk1 wk5 wk10
O21.
Time

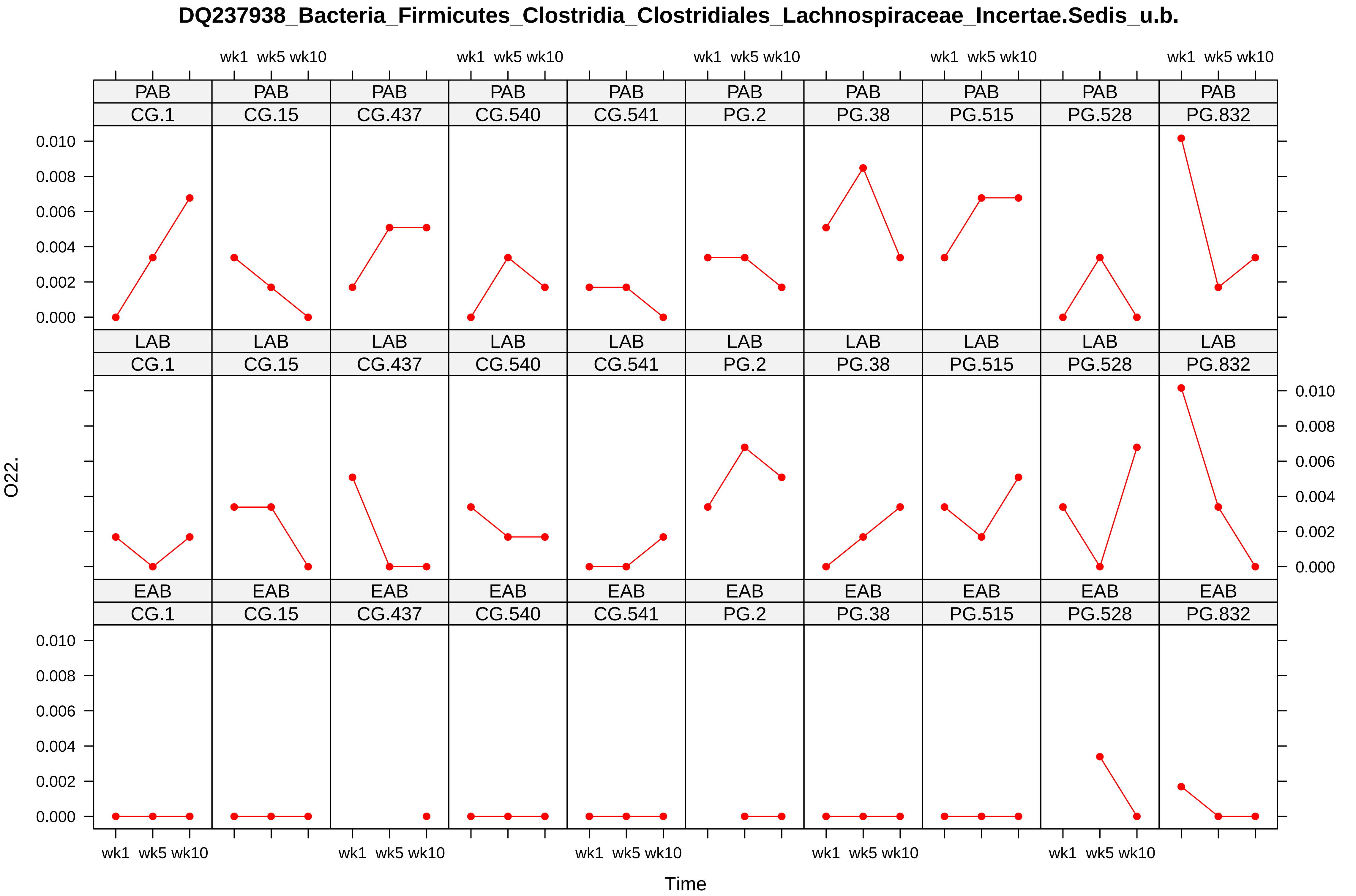

DQ237938_Bacteria_Firmicutes_Clostridia_Clostridiales_Lachnospiraceae_Incertae.Sedis_u.b.
PAB
LAB
EAB
CG.1
CG.15
CG.437
CG.540
CG.541
PG.2
PG.38
PG.515
PG.528
PG.832
wk1 wk5 wk10
0.000
0.002
0.004
0.006
0.008
0.010
O22.
Time

EF436445_Bacteria_Firmicutes_Clostridia_Clostridiales_Lachnospiraceae_Incertae.Sedis_u.b.

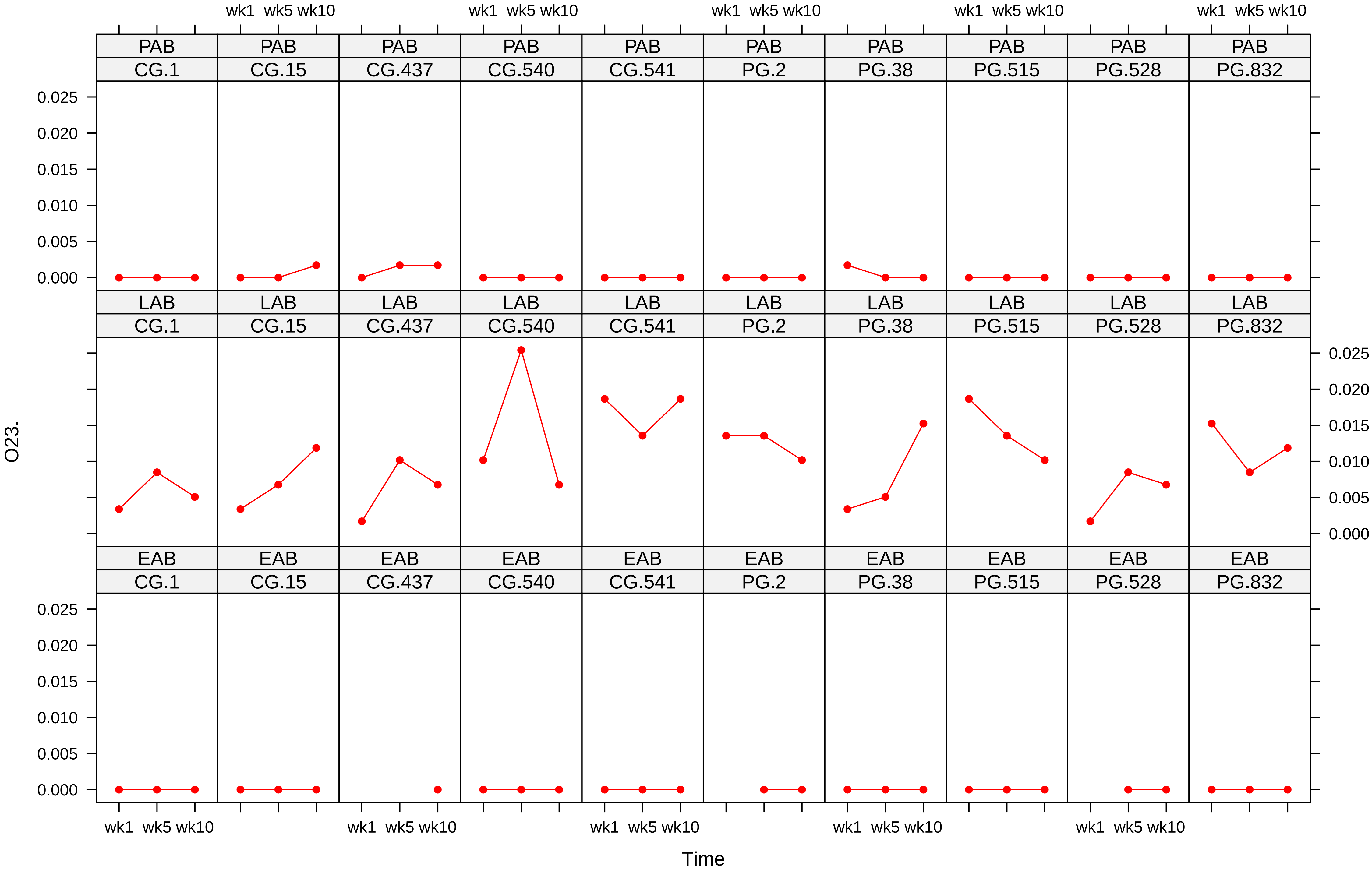

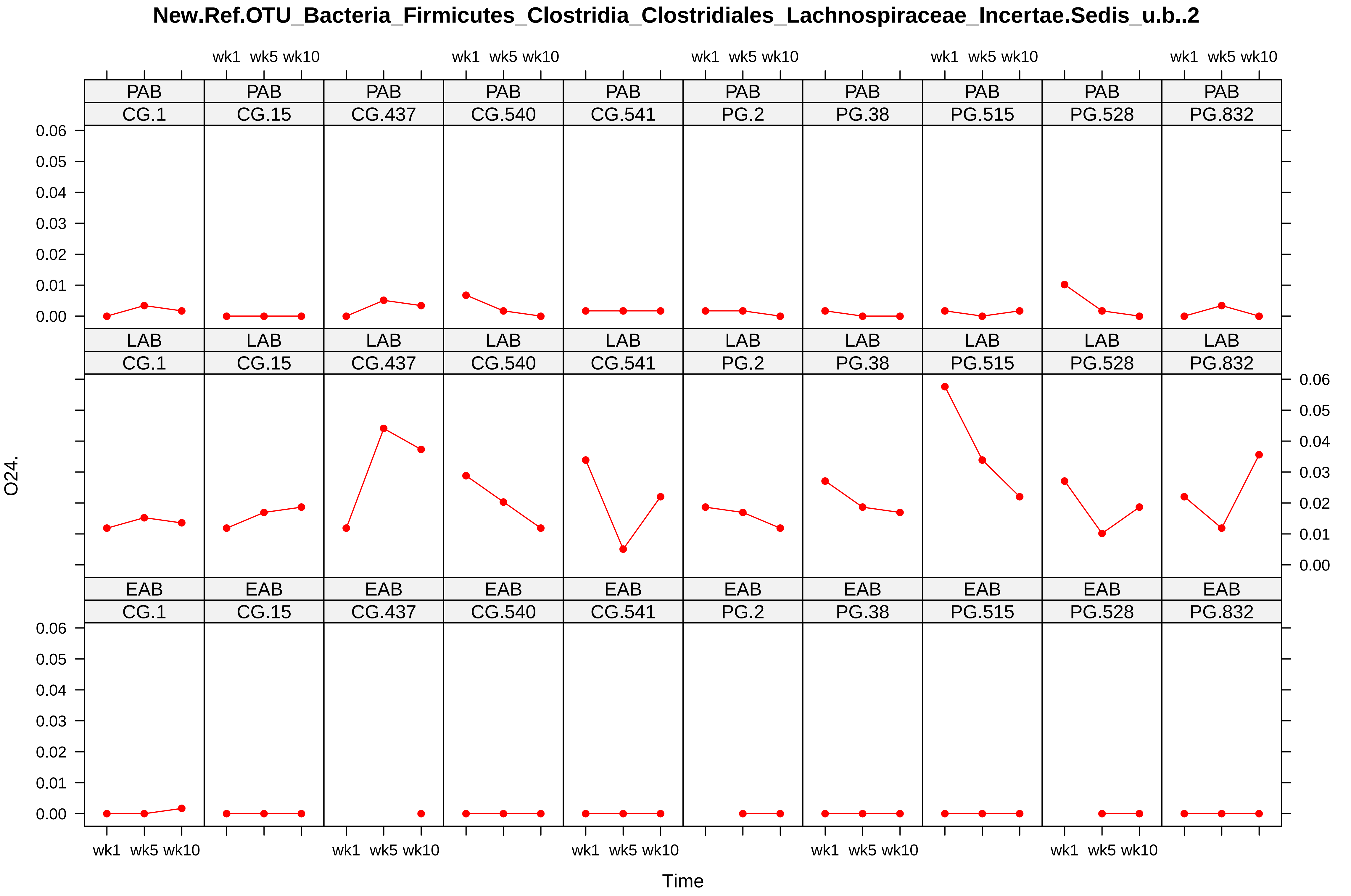
New.Ref.OTU_Bacteria_Firmicutes_Clostridia_Clostridiales_Lachnospiraceae_Incertae.Sedis_u.b..2
PAB
LAB
EAB
CG.1
CG.15
CG.437
CG.540
CG.541
PG.2
PG.38
PG.515
PG.528
PG.832
0.00
0.01
0.02
0.03
0.04
0.05
0.06
wk1
wk5
wk10
O24.
Time

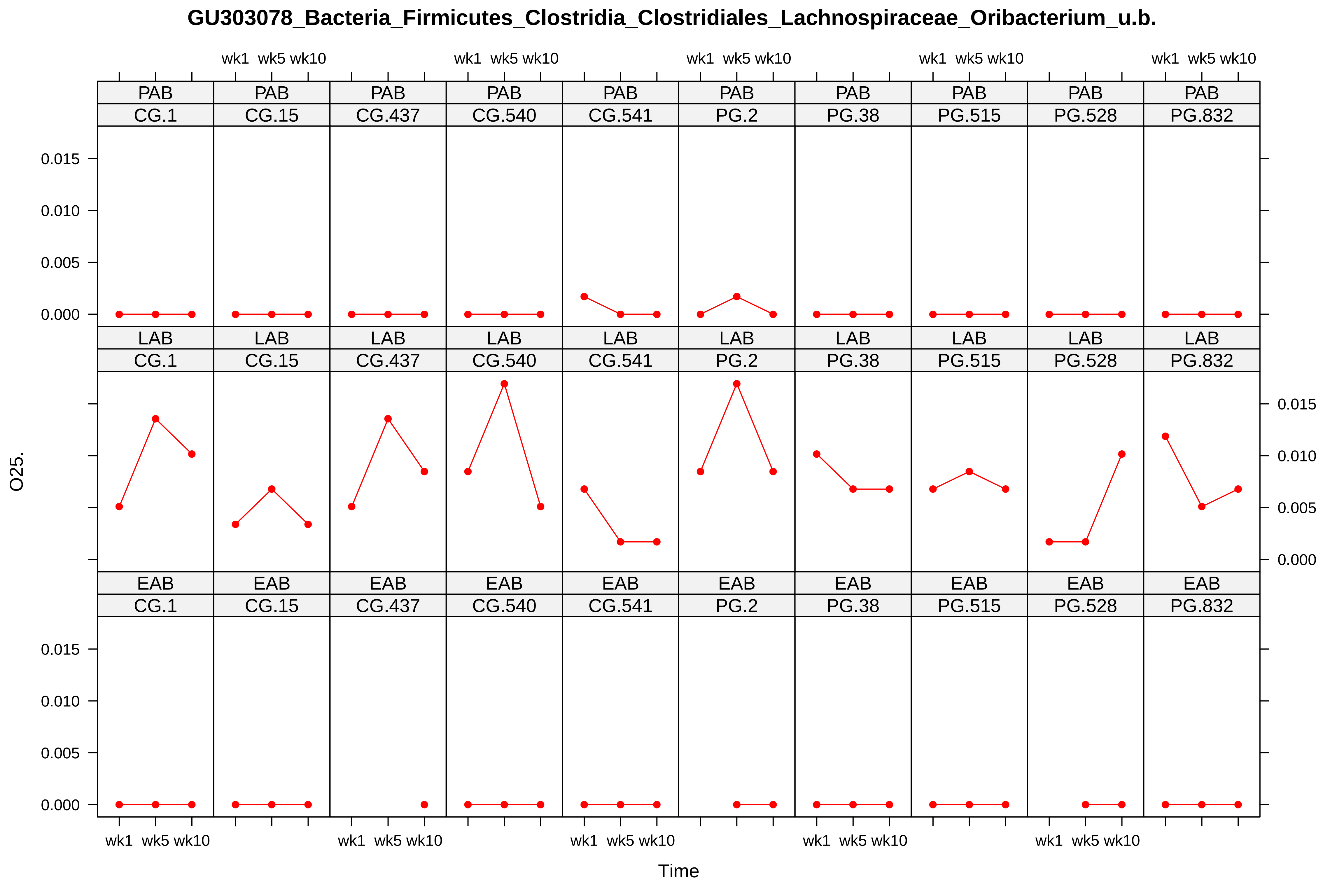
GU303078_Bacteria_Firmicutes_Clostridia_Clostridiales_Lachnospiraceae_Oribacterium_u.b.
PAB
LAB
EAB
CG.1
CG.15
CG.437
CG.540
CG.541
PG.2
PG.38
PG.515
PG.528
PG.832
O25.
Time
wk1 wk5 wk10
0.000
0.005
0.010
0.015

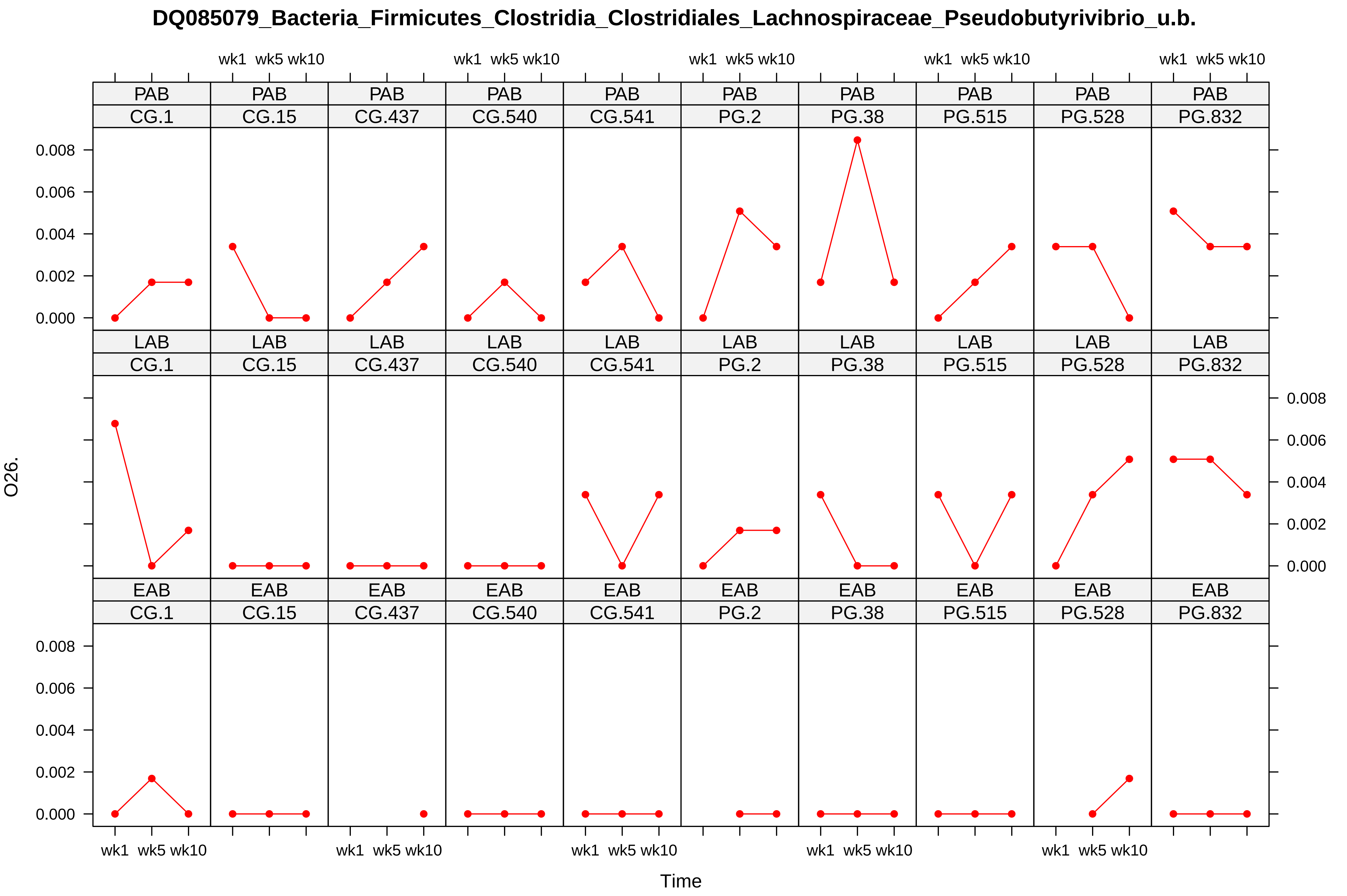
DQ085079_Bacteria_Firmicutes_Clostridia_Clostridiales_Lachnospiraceae_Pseudobutyrivibrio_u.b.
PAB
LAB
EAB
CG.1
CG.15
CG.437
CG.540
CG.541
PG.2
PG.38
PG.515
PG.528
PG.832
0.000
0.002
0.004
0.006
0.008
wk1
wk5
wk10
O26.
Time

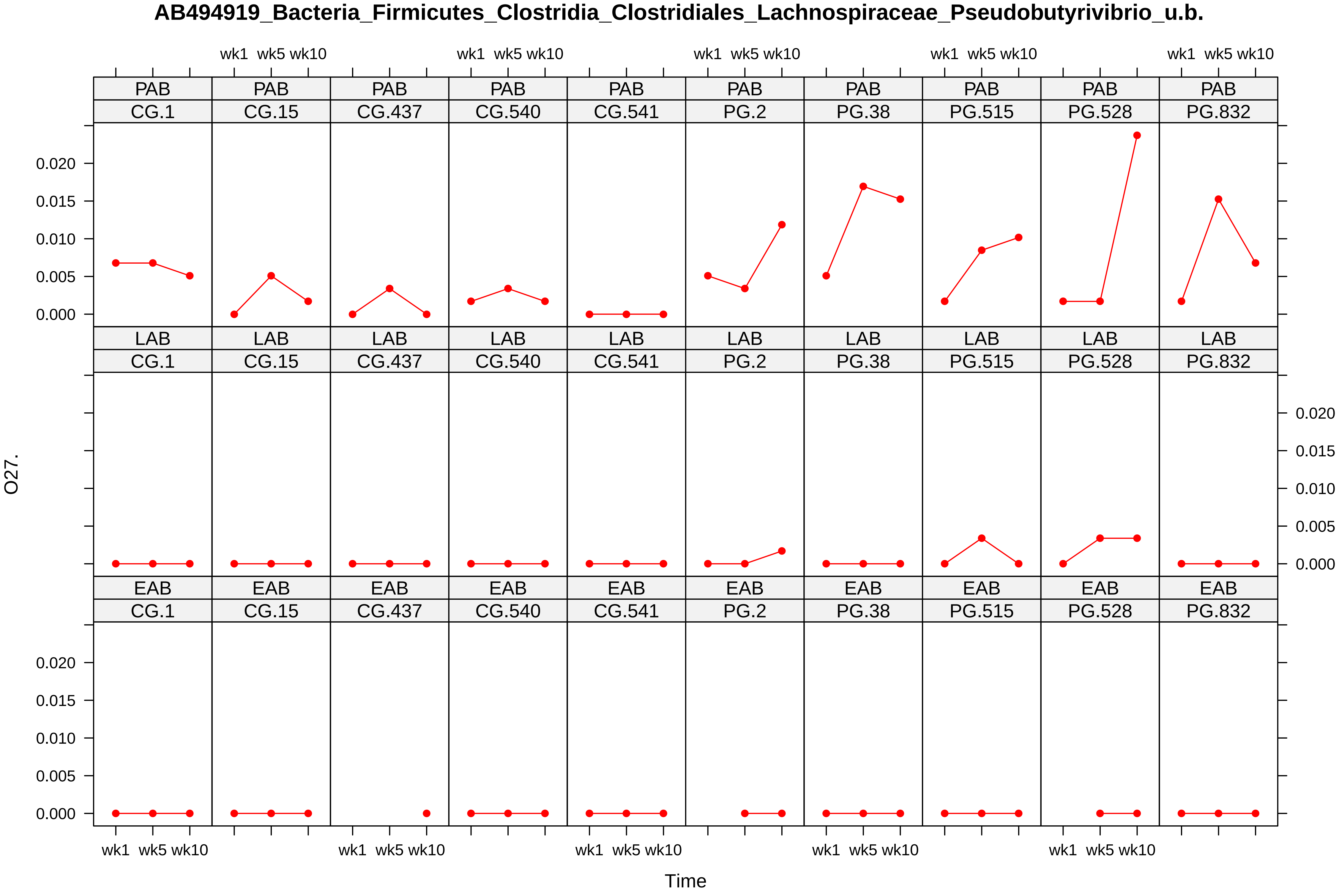
AB494919_Bacteria_Firmicutes_Clostridia_Clostridiales_Lachnospiraceae_Pseudobutyrivibrio_u.b.
PAB
LAB
EAB
CG.1
CG.15
CG.437
CG.540
CG.541
PG.2
PG.38
PG.515
PG.528
PG.832
0.000
0.005
0.010
0.015
0.020
wk1 wk5 wk10
O27.
Time

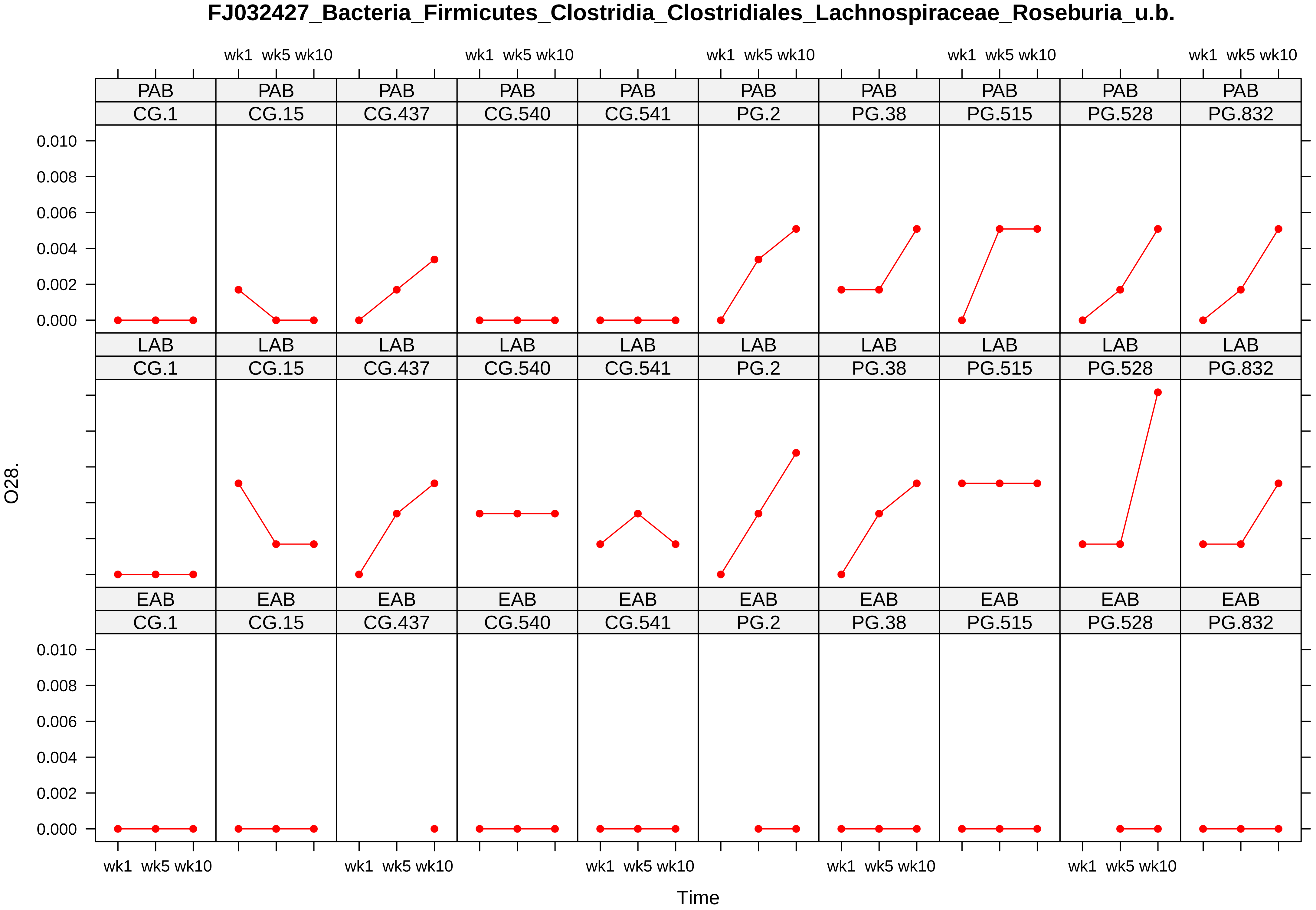
FJ032427_Bacteria_Firmicutes_Clostridia_Clostridiales_Lachnospiraceae_Roseburia_u.b.
PAB
LAB
EAB
CG.1
CG.15
CG.437
CG.540
CG.541
PG.2
PG.38
PG.515
PG.528
PG.832
0.000
0.002
0.004
0.006
0.008
0.010
wk1 wk5 wk10
O28.
Time

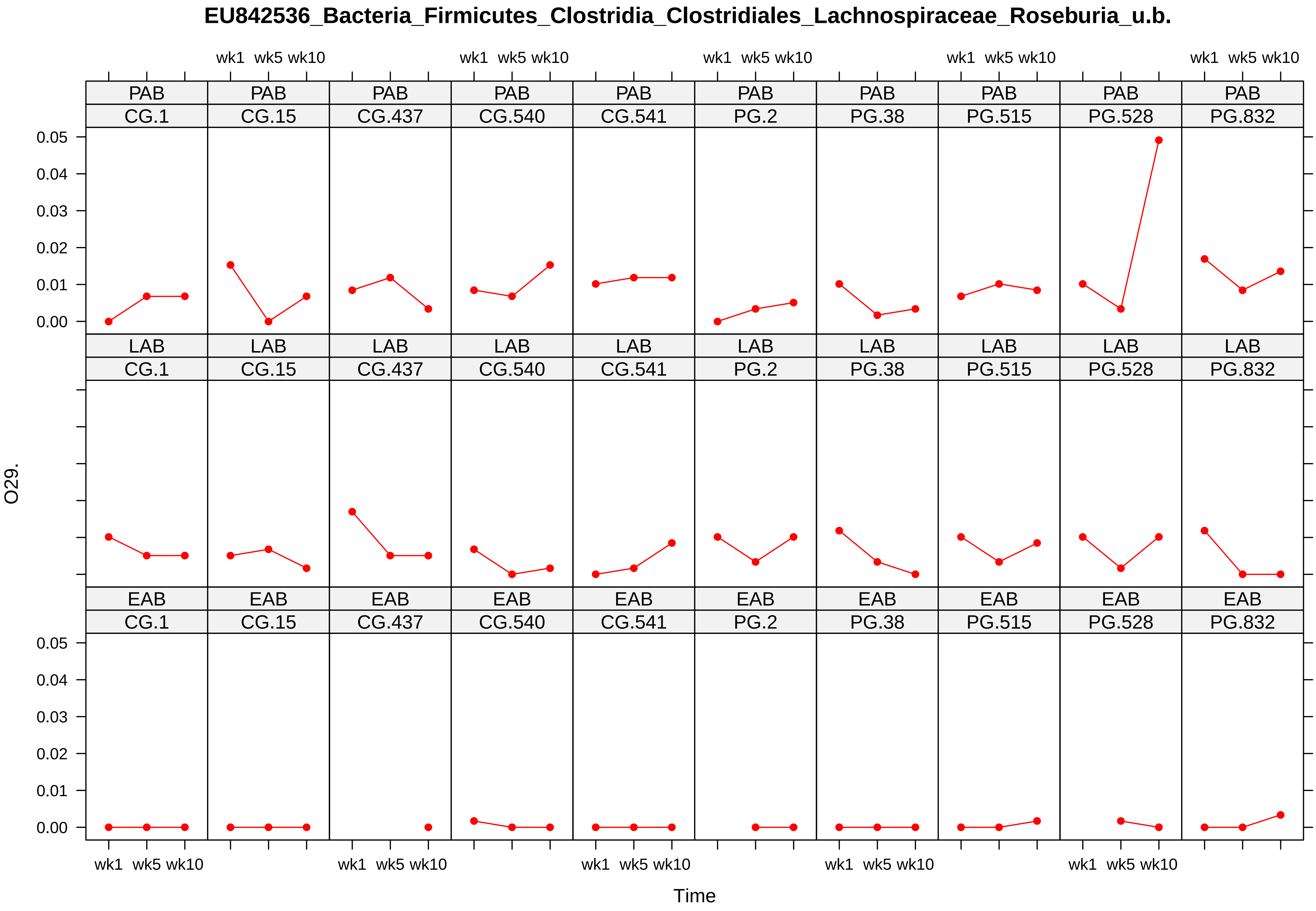
EU842536_Bacteria_Firmicutes_Clostridia_Clostridiales_Lachnospiraceae_Roseburia_u.b.
PAB
LAB
EAB
CG.1
CG.15
CG.437
CG.540
CG.541
PG.2
PG.38
PG.515
PG.528
PG.832
0.00
0.01
0.02
0.03
0.04
0.05
wk1
wk5
wk10
O29.
Time

AF371623_Bacteria_Firmicutes_Clostridia_Clostridiales_Lachnospiraceae_Roseburia_u.b.

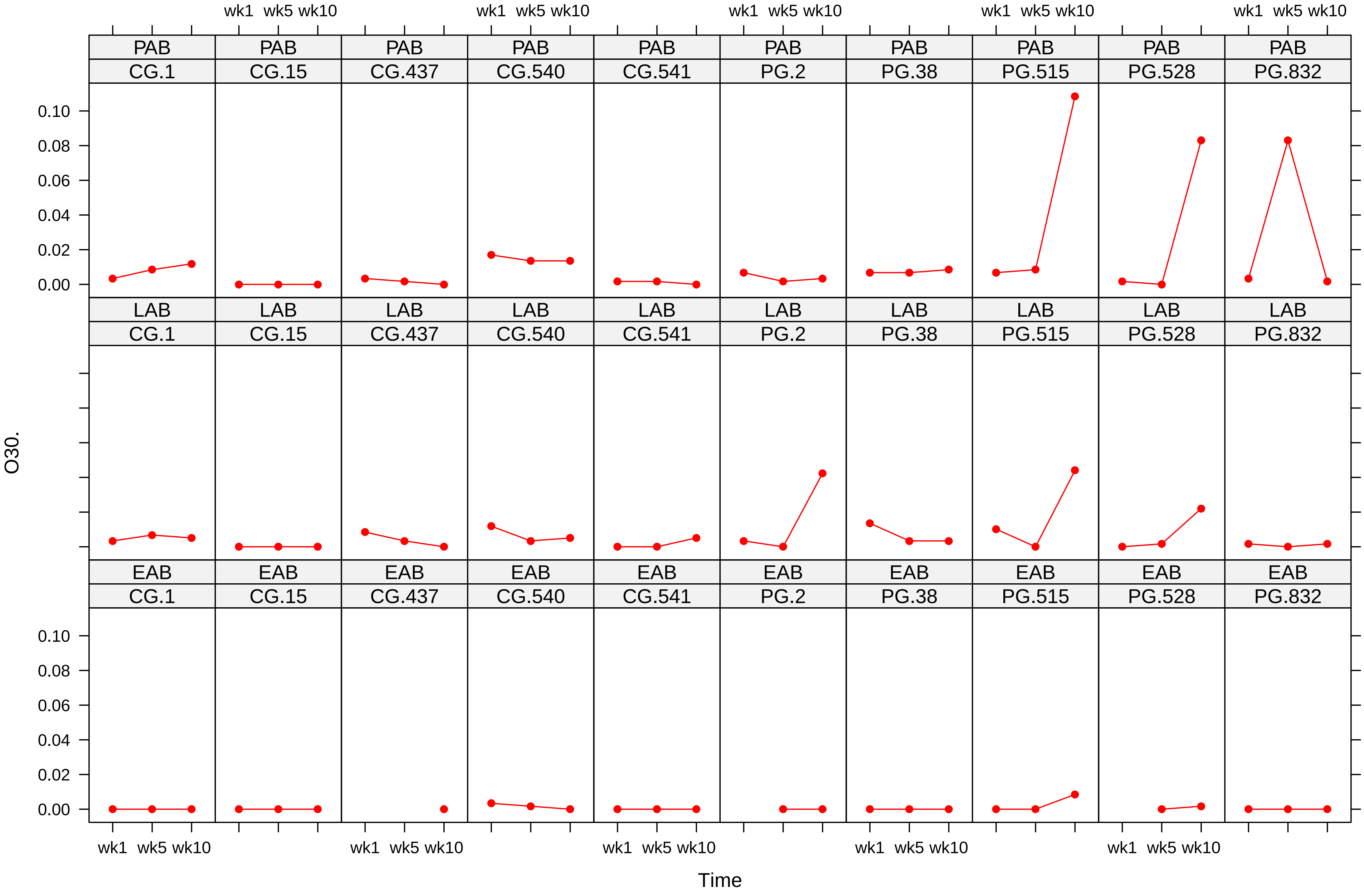

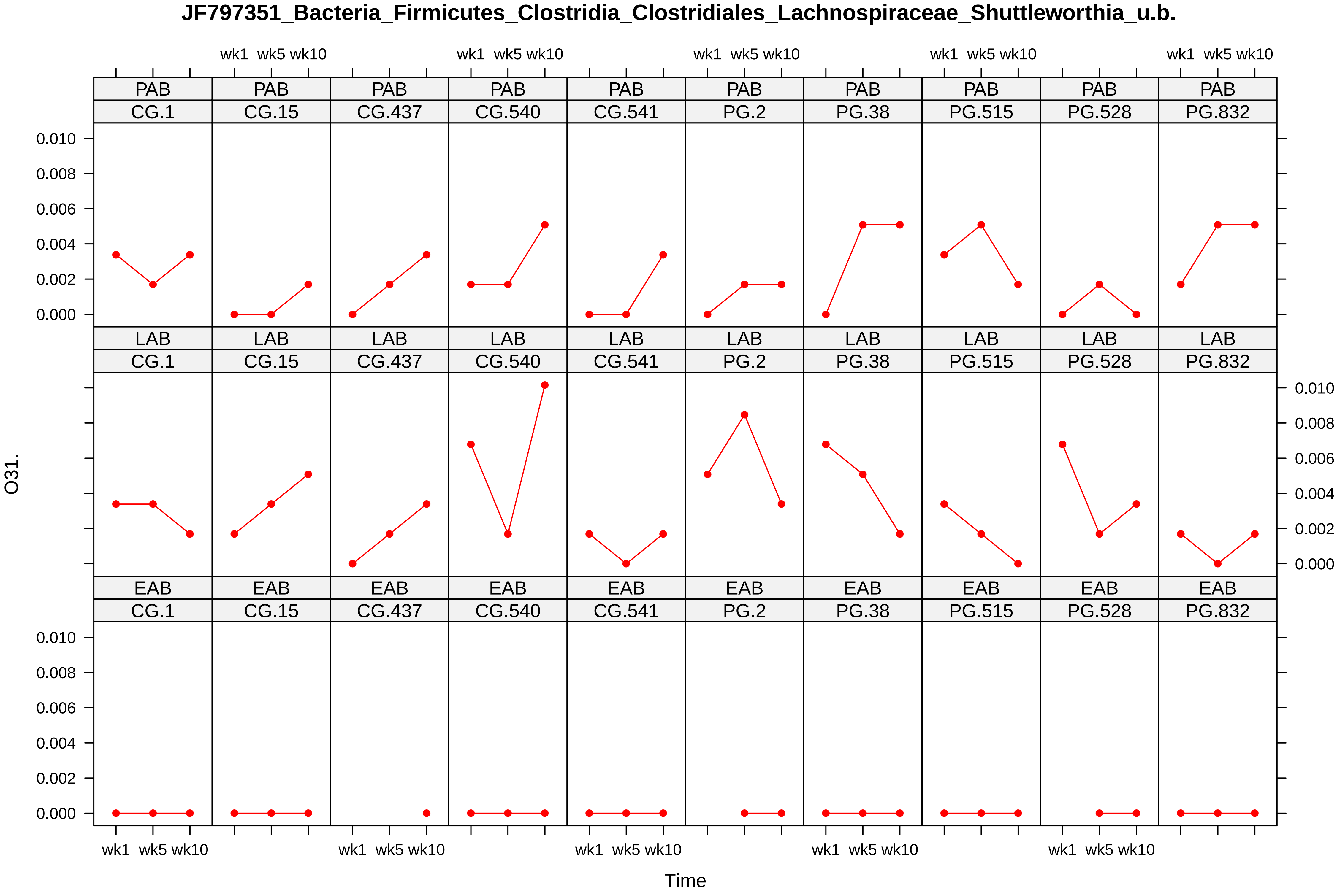

JF797351_Bacteria_Firmicutes_Clostridia_Clostridiales_Lachnospiraceae_Shuttleworthia_u.b.
PAB
LAB
EAB
CG.1
CG.15
CG.437
CG.540
CG.541
PG.2
PG.38
PG.515
PG.528
PG.832
0.000
0.002
0.004
0.006
0.008
0.010
wk1 wk5 wk10
O31.
Time

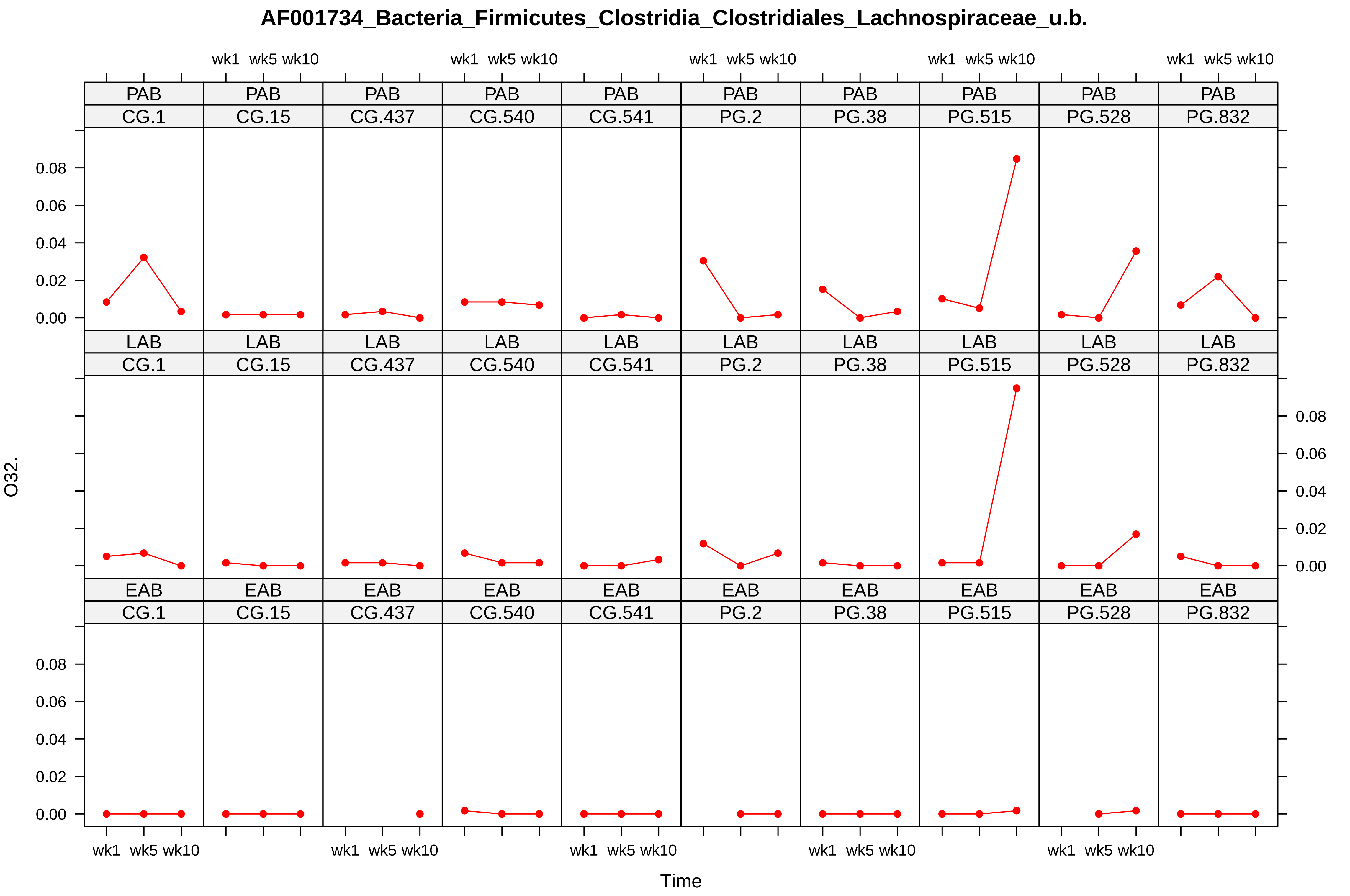

AF001734_Bacteria_Firmicutes_Clostridia_Clostridiales_Lachnospiraceae_u.b.
PAB CG.1
PAB CG.15
PAB CG.437
PAB CG.540
PAB CG.541
PAB PG.2
PAB PG.38
PAB PG.515
PAB PG.528
PAB PG.832
LAB CG.1
LAB CG.15
LAB CG.437
LAB CG.540
LAB CG.541
LAB PG.2
LAB PG.38
LAB PG.515
LAB PG.528
LAB PG.832
EAB CG.1
EAB CG.15
EAB CG.437
EAB CG.540
EAB CG.541
EAB PG.2
EAB PG.38
EAB PG.515
EAB PG.528
EAB PG.832
0.00
0.02
0.04
0.06
0.08
wk1 wk5 wk10
O32.
Time

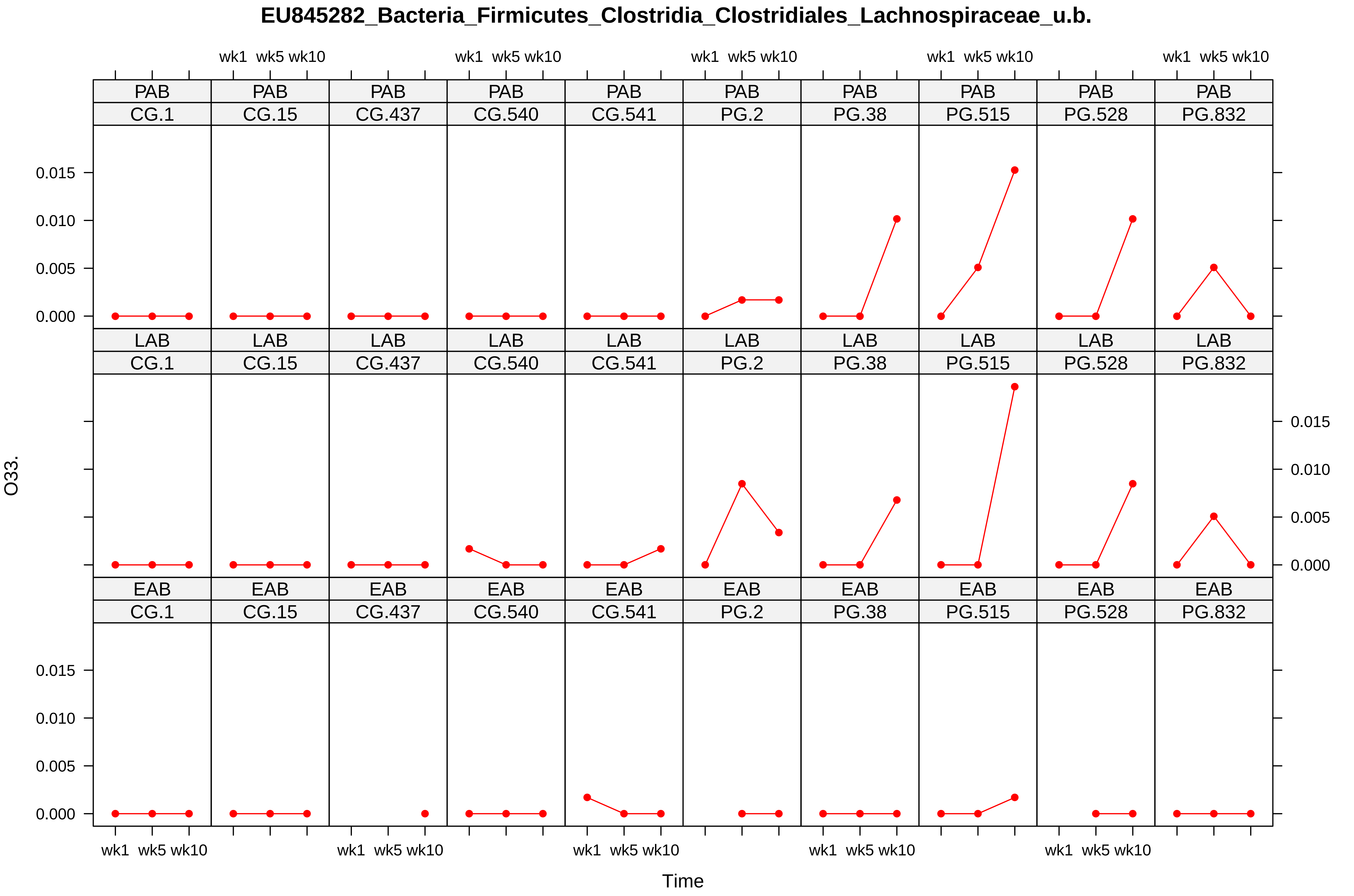
EU845282_Bacteria_Firmicutes_Clostridia_Clostridiales_Lachnospiraceae_u.b.
PAB
LAB
EAB
CG.1
CG.15
CG.437
CG.540
CG.541
PG.2
PG.38
PG.515
PG.528
PG.832
0.000
0.005
0.010
0.015
wk1 wk5 wk10
O33.
Time

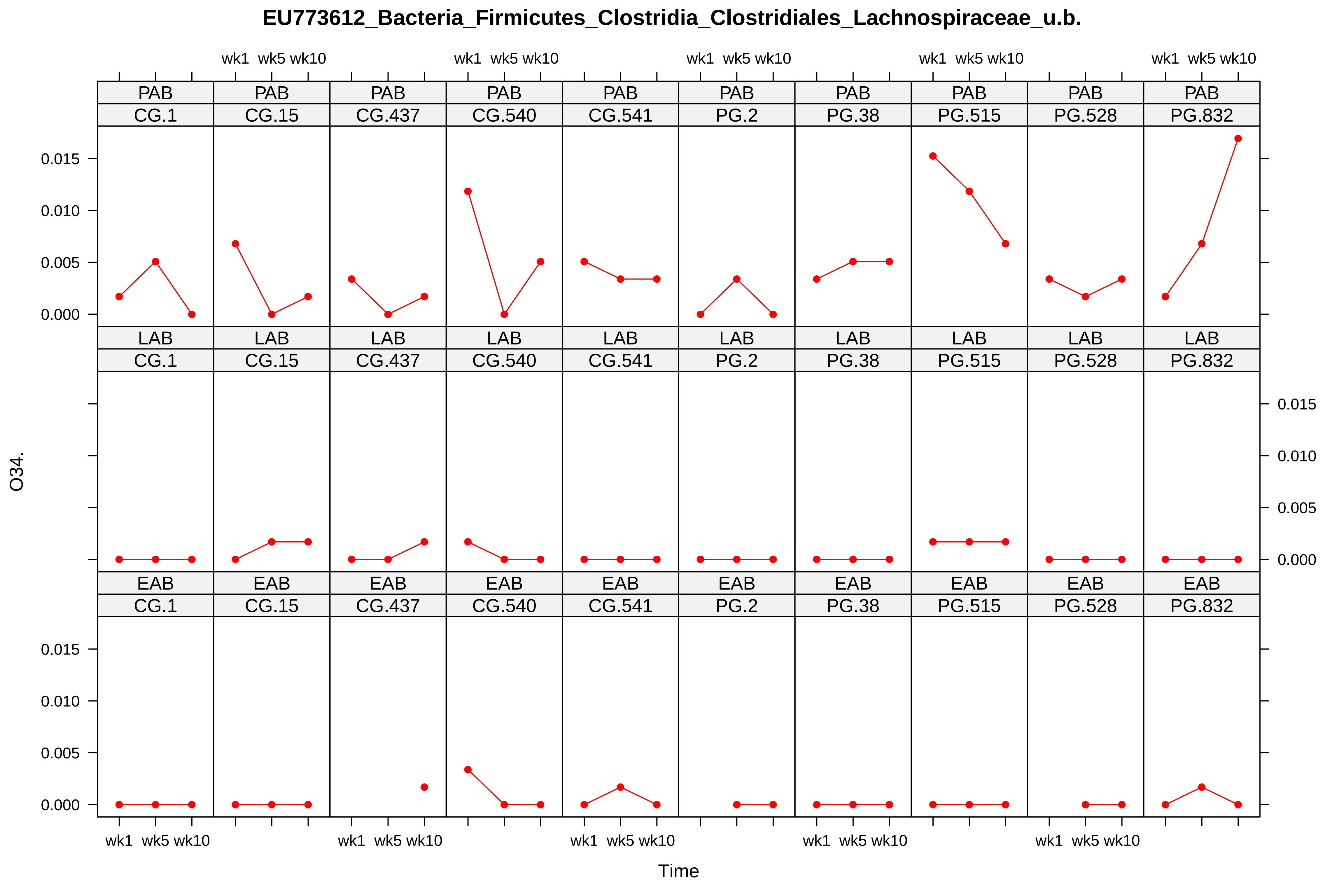

EU773612_Bacteria_Firmicutes_Clostridia_Clostridiales_Lachnospiraceae_u.b.
PAB
LAB
EAB
CG.1
CG.15
CG.437
CG.540
CG.541
PG.2
PG.38
PG.515
PG.528
PG.832
wk1 wk5 wk10
0.000
0.005
0.010
0.015
O34.
Time

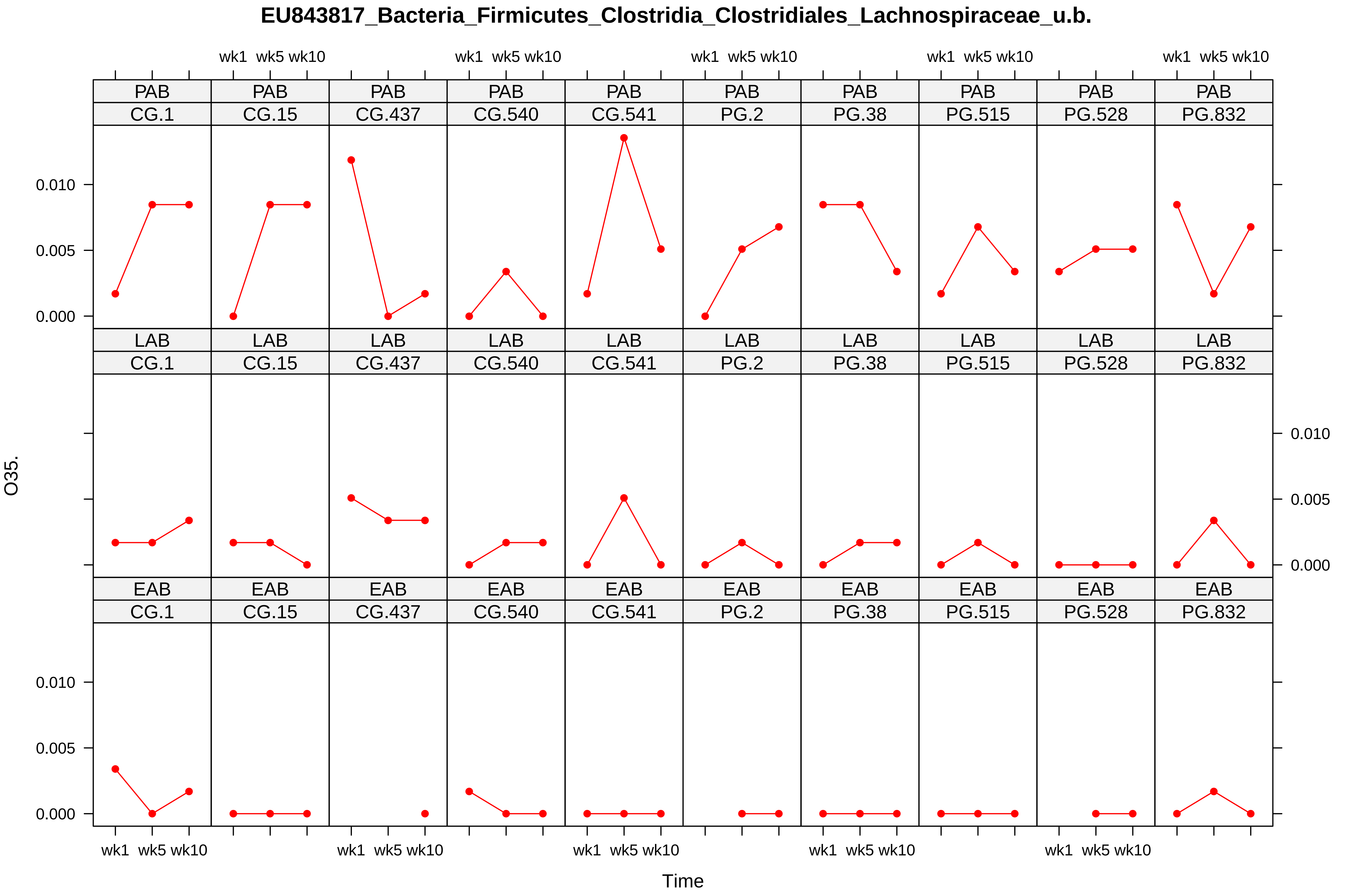
EU843817_Bacteria_Firmicutes_Clostridia_Clostridiales_Lachnospiraceae_u.b.
PAB
LAB
EAB
CG.1
CG.15
CG.437
CG.540
CG.541
PG.2
PG.38
PG.515
PG.528
PG.832
wk1 wk5 wk10
0.000
0.005
0.010
O35.
Time

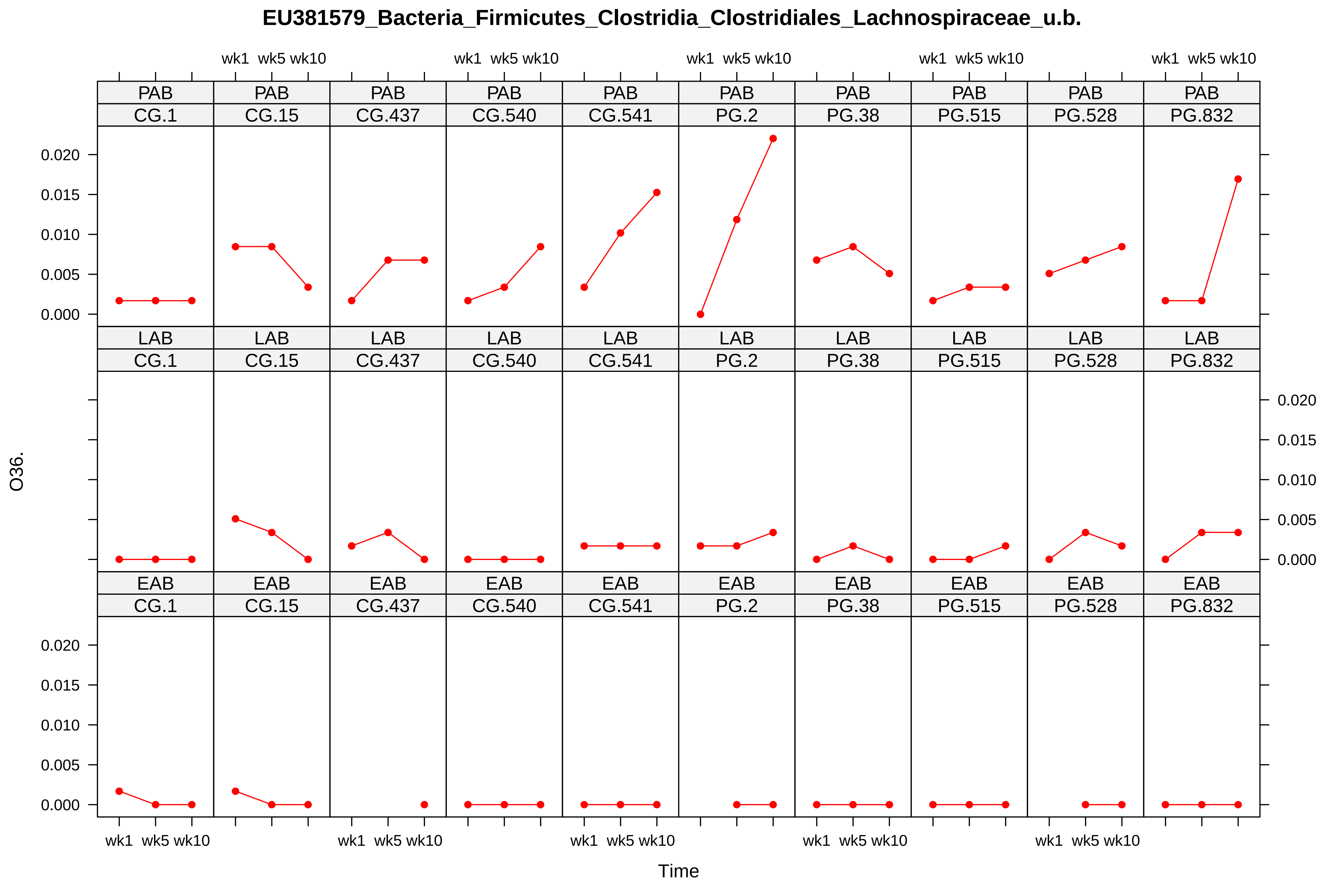
EU381579_Bacteria_Firmicutes_Clostridia_Clostridiales_Lachnospiraceae_u.b.
PAB
LAB
EAB
CG.1
CG.15
CG.437
CG.540
CG.541
PG.2
PG.38
PG.515
PG.528
PG.832
0.000
0.005
0.010
0.015
0.020
wk1
wk5
wk10
O36.
Time

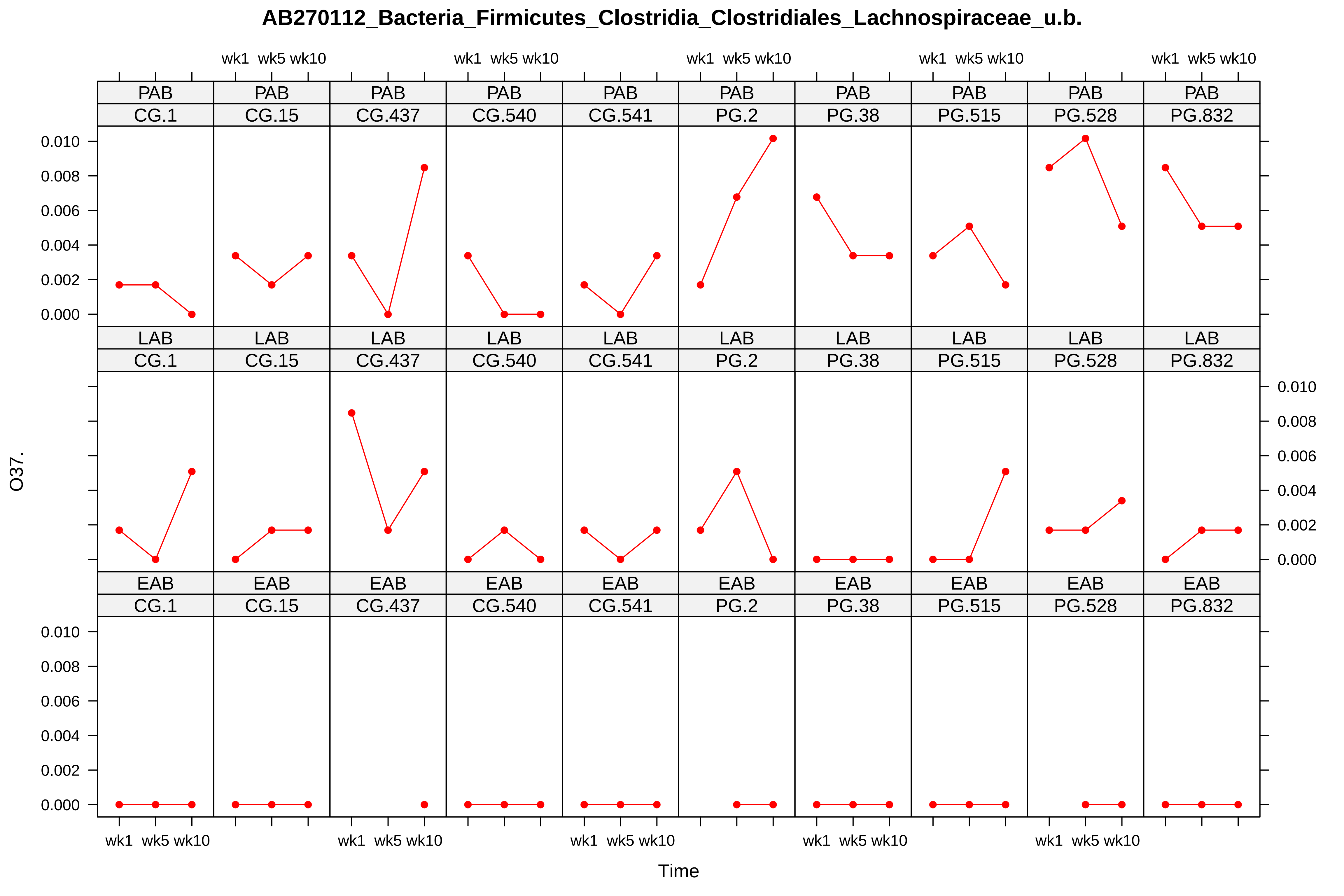
AB270112_Bacteria_Firmicutes_Clostridia_Clostridiales_Lachnospiraceae_u.b.
PAB
LAB
EAB
CG.1
CG.15
CG.437
CG.540
CG.541
PG.2
PG.38
PG.515
PG.528
PG.832
O37.
Time
wk1 wk5 wk10
0.000
0.002
0.004
0.006
0.008
0.010

AB494866_Bacteria_Firmicutes_Clostridia_Clostridiales_Lachnospiraceae_u.b.

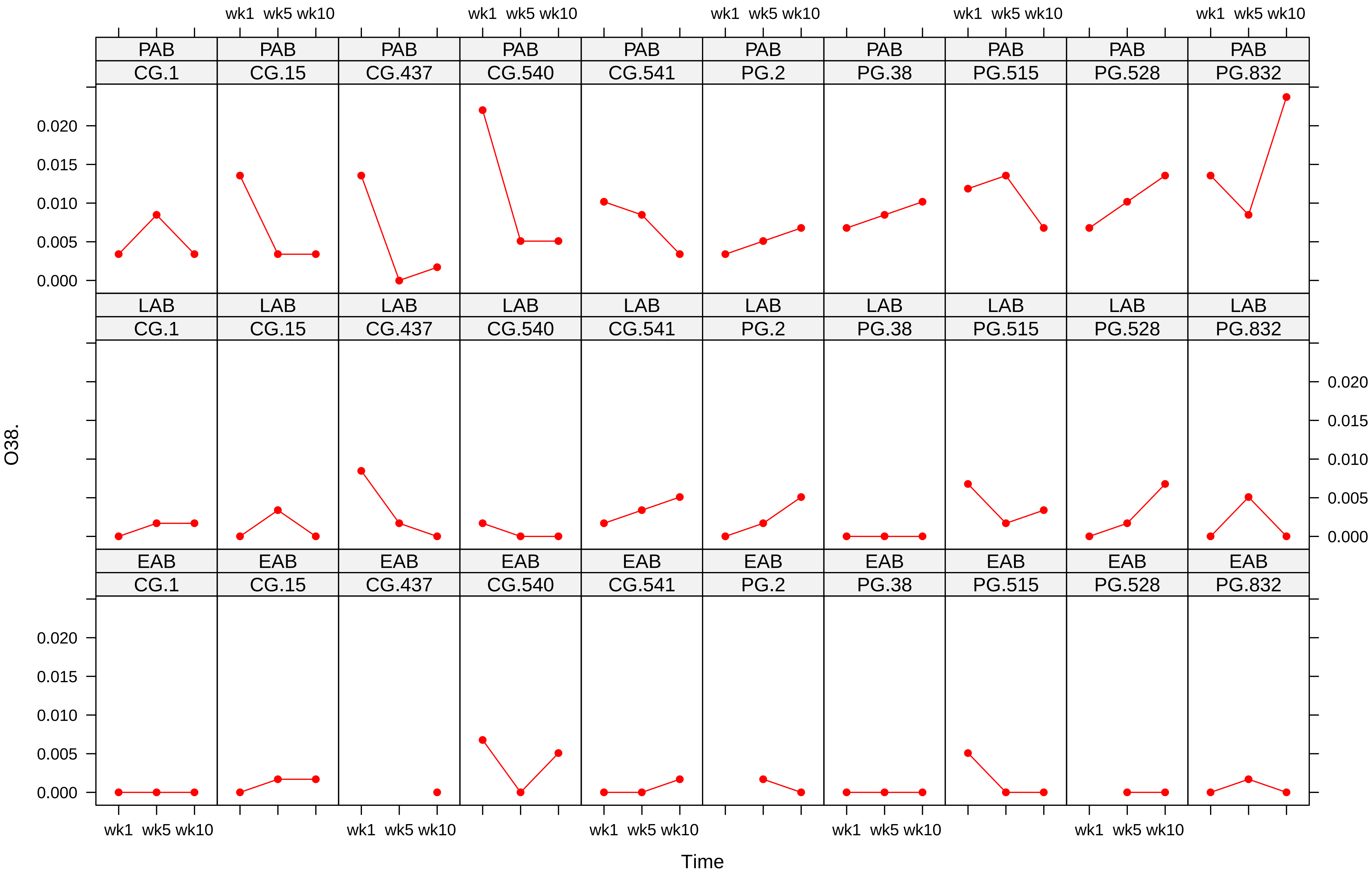

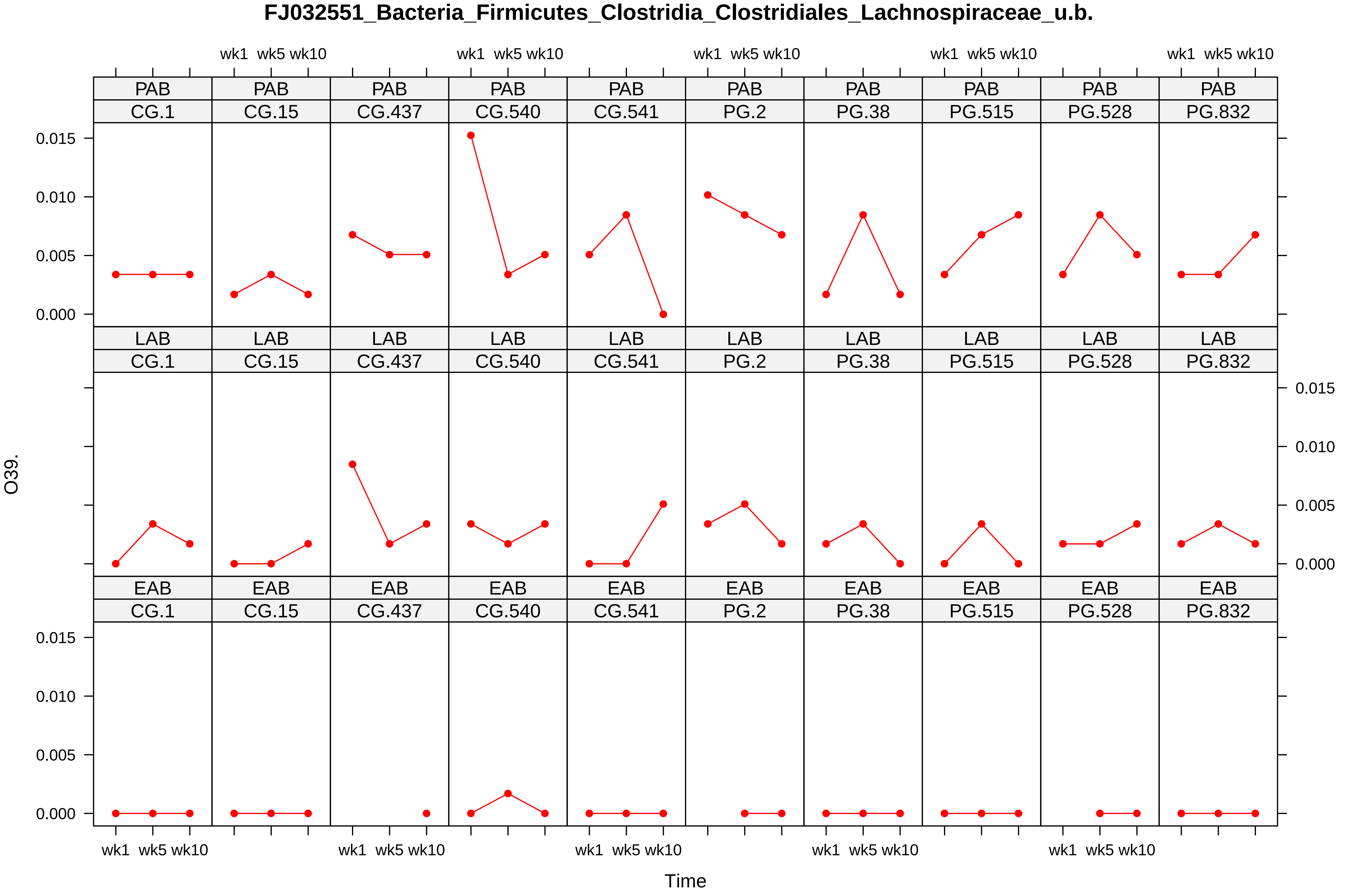

FJ032551_Bacteria_Firmicutes_Clostridia_Clostridiales_Lachnospiraceae_u.b.
PAB
LAB
EAB
CG.1
CG.15
CG.437
CG.540
CG.541
PG.2
PG.38
PG.515
PG.528
PG.832
0.000
0.005
0.010
0.015
wk1 wk5 wk10
O39.
Time

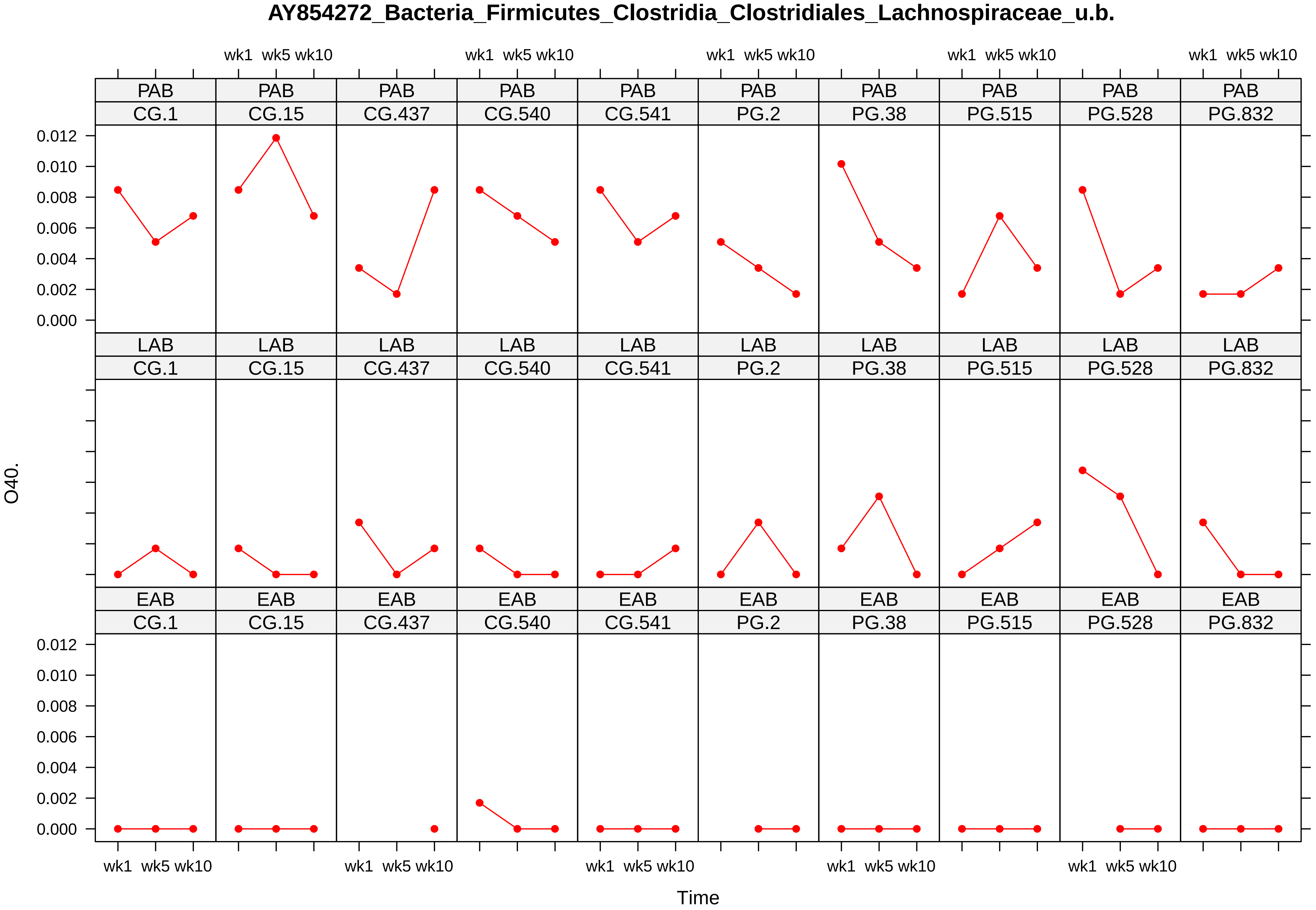

AY854272_Bacteria_Firmicutes_Clostridia_Clostridiales_Lachnospiraceae_u.b.
PAB
LAB
EAB
CG.1
CG.15
CG.437
CG.540
CG.541
PG.2
PG.38
PG.515
PG.528
PG.832
wk1 wk5 wk10
0.000
0.002
0.004
0.006
0.008
0.010
0.012
O40.
Time

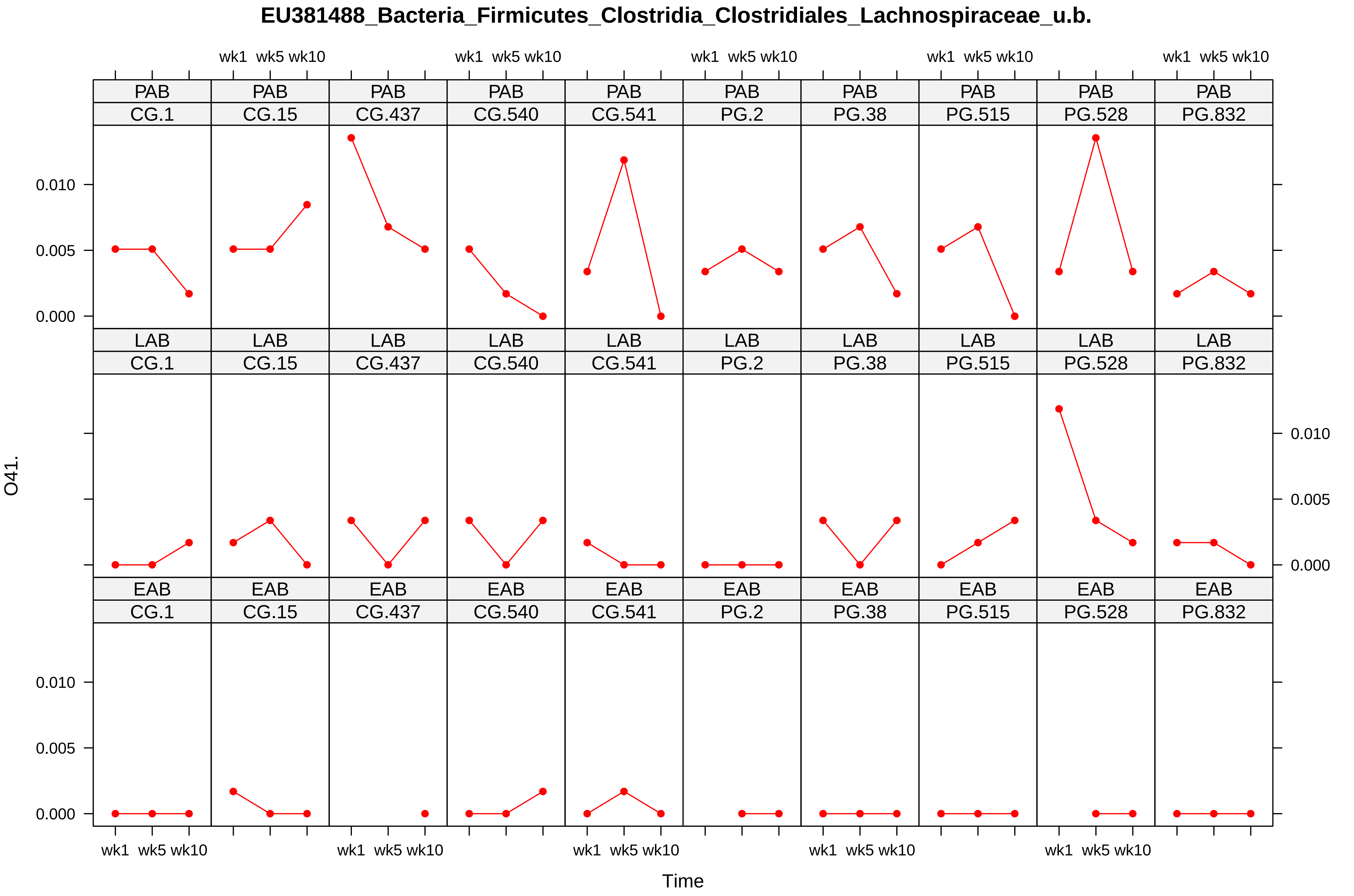
EU381488_Bacteria_Firmicutes_Clostridia_Clostridiales_Lachnospiraceae_u.b.
PAB
LAB
EAB
CG.1
CG.15
CG.437
CG.540
CG.541
PG.2
PG.38
PG.515
PG.528
PG.832
0.000
0.005
0.010
wk1
wk5
wk10
O41.
Time

AF001717_Bacteria_Firmicutes_Clostridia_Clostridiales_Lachnospiraceae_u.b.

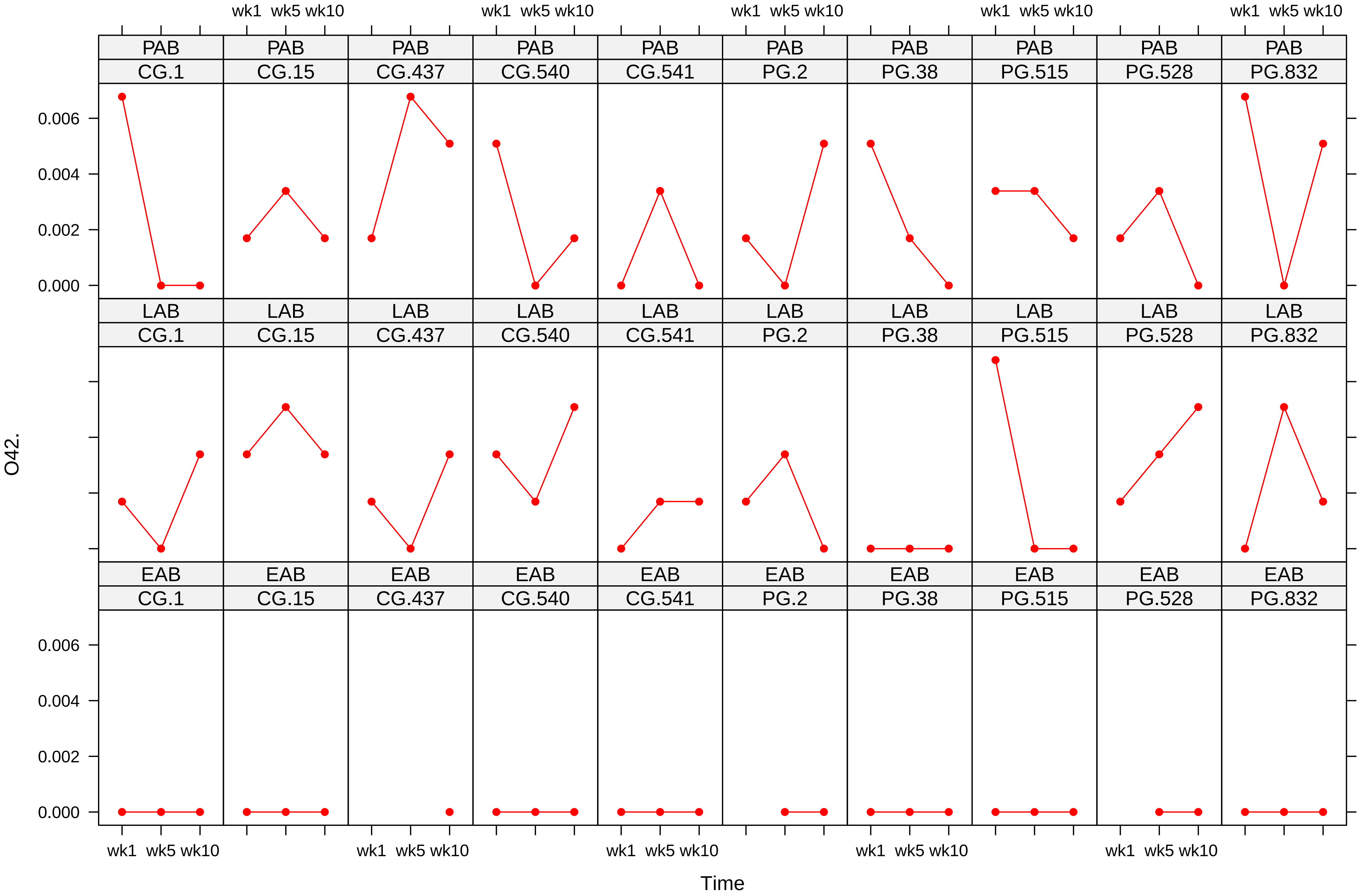

EU719231_Bacteria_Firmicutes_Clostridia_Clostridiales_Lachnospiraceae_u.b.

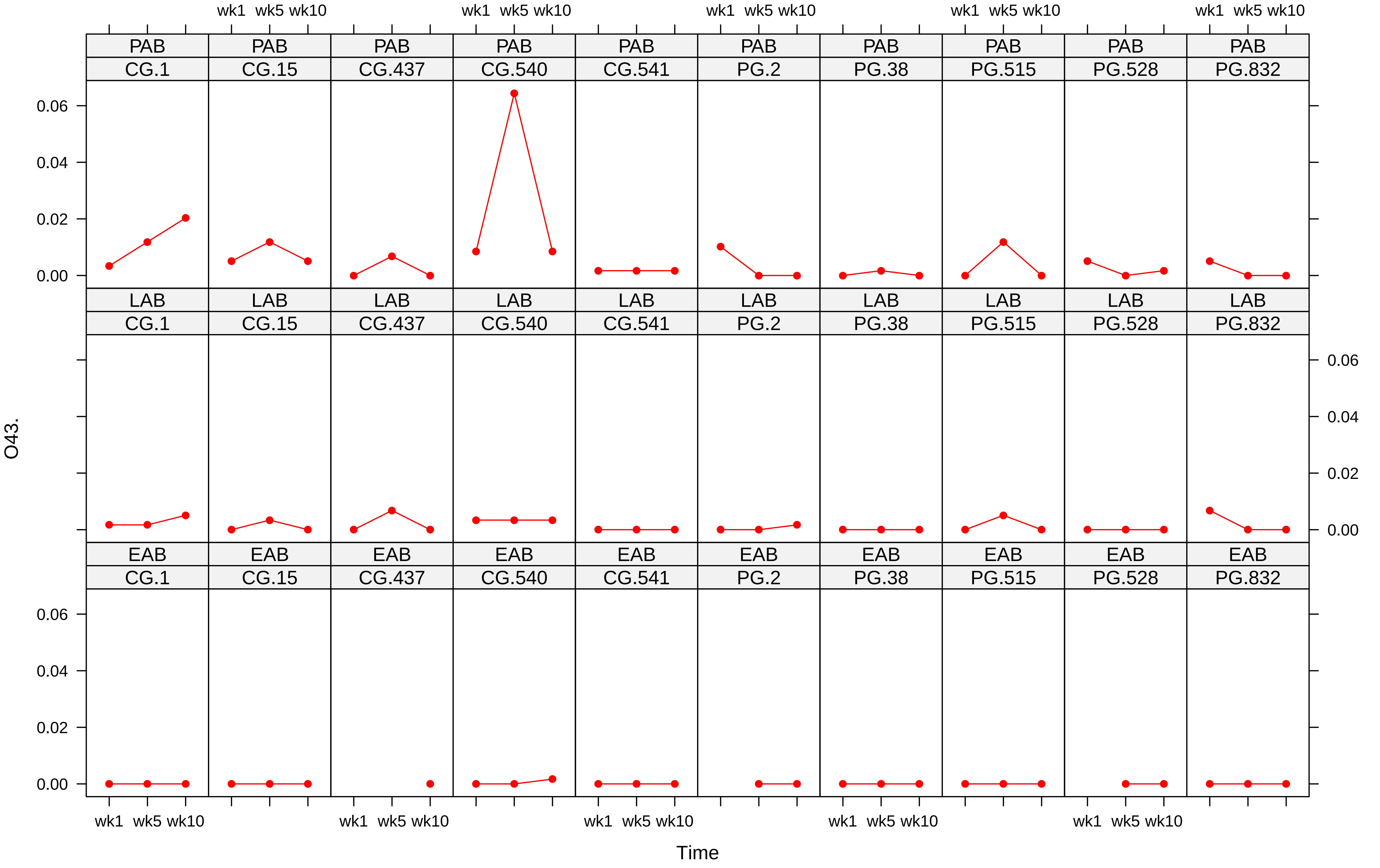

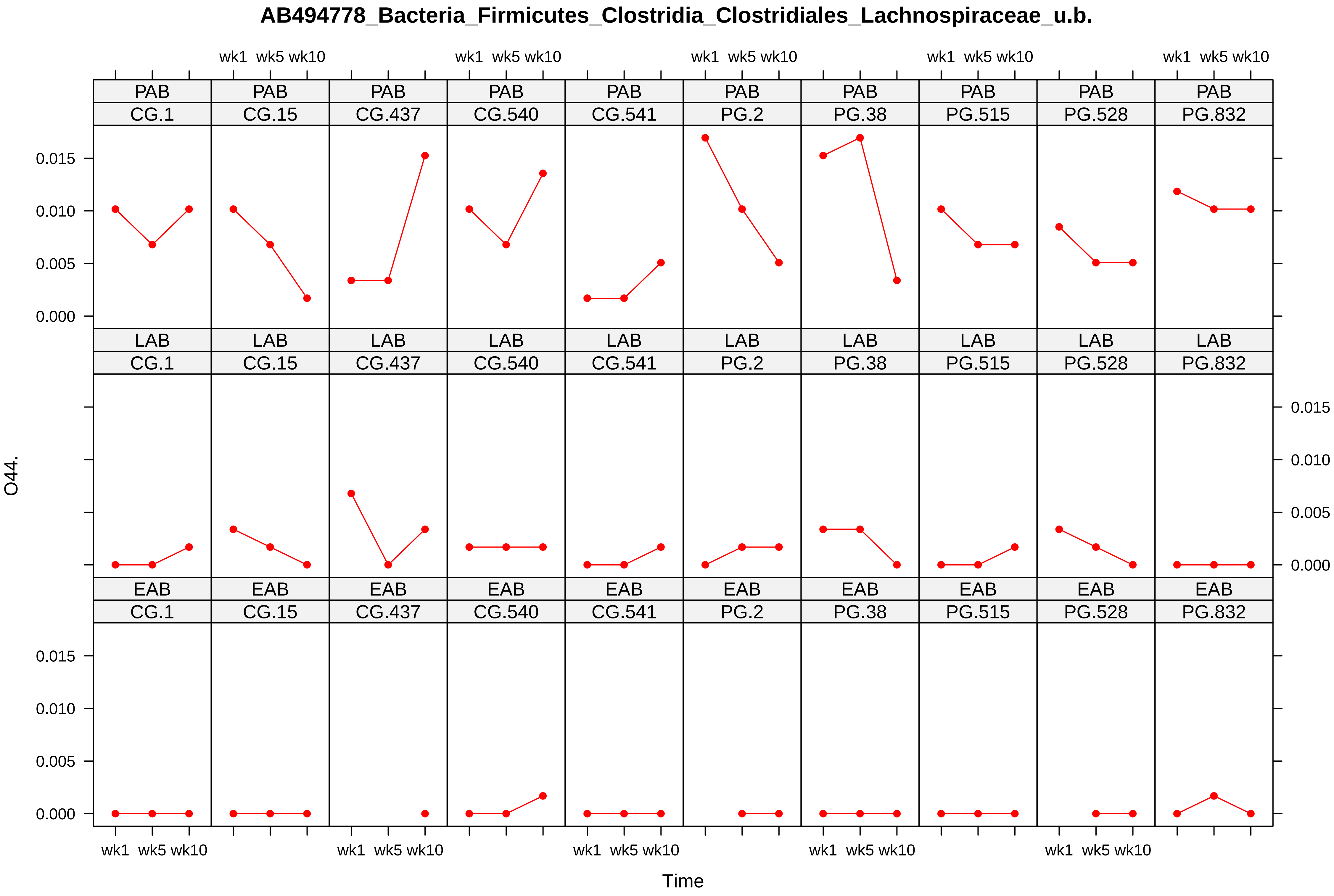

AB494778_Bacteria_Firmicutes_Clostridia_Clostridiales_Lachnospiraceae_u.b.
PAB
LAB
EAB
CG.1
CG.15
CG.437
CG.540
CG.541
PG.2
PG.38
PG.515
PG.528
PG.832
wk1 wk5 wk10
0.000
0.005
0.010
0.015
O44.
Time

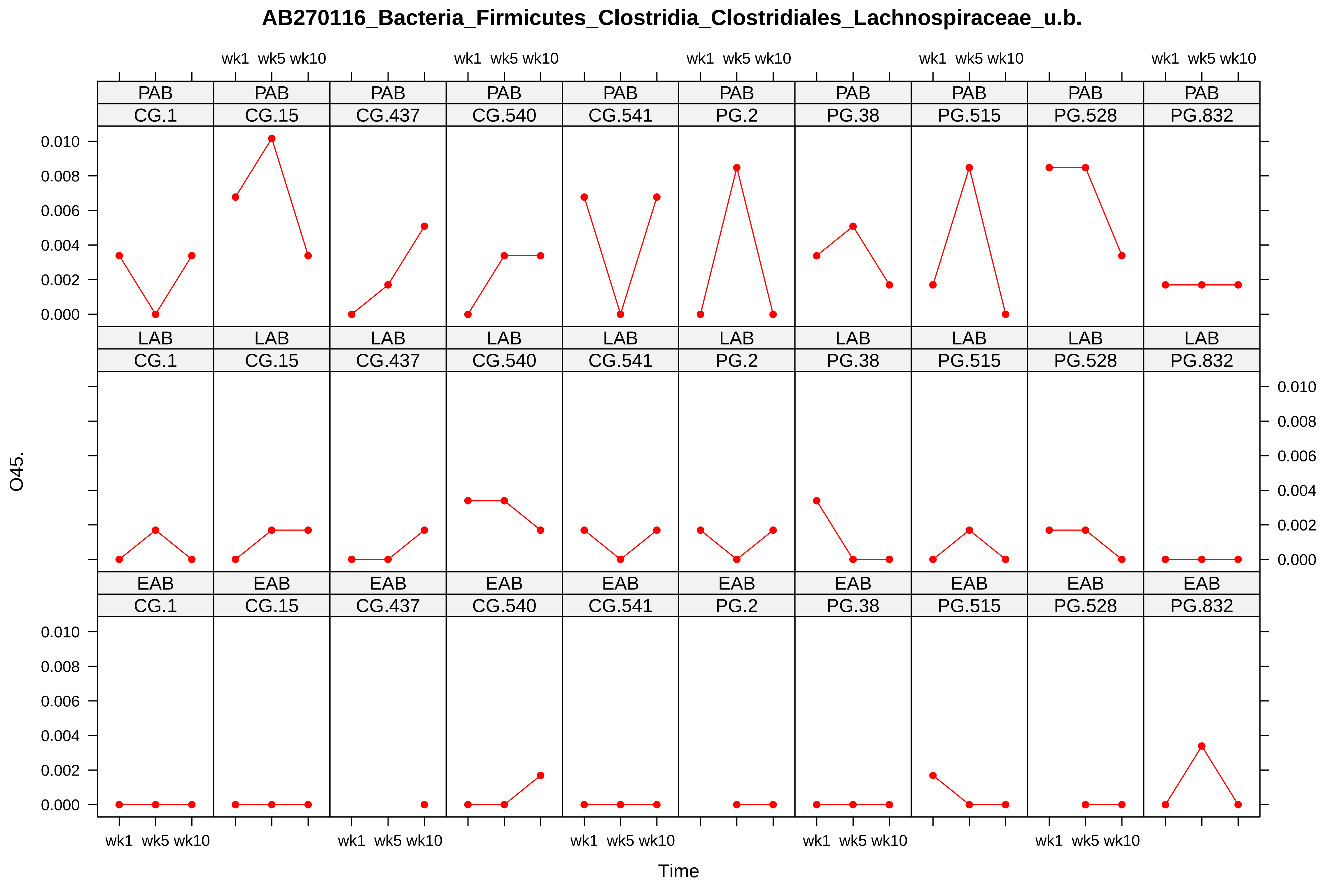
AB270116_Bacteria_Firmicutes_Clostridia_Clostridiales_Lachnospiraceae_u.b.
PAB
LAB
EAB
CG.1
CG.15
CG.437
CG.540
CG.541
PG.2
PG.38
PG.515
PG.528
PG.832
O45.
Time
wk1 wk5 wk10
0.000
0.002
0.004
0.006
0.008
0.010

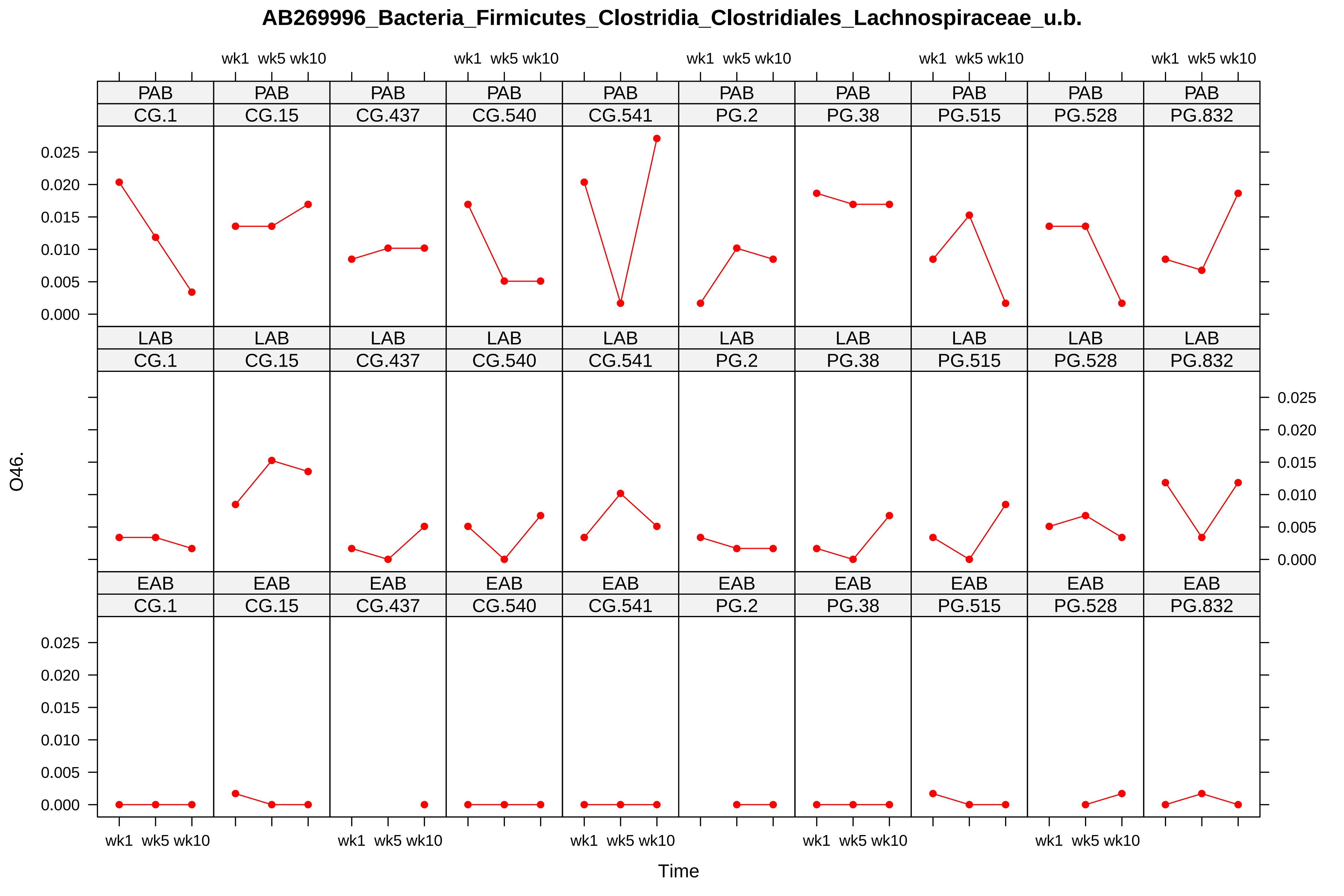
AB269996_Bacteria_Firmicutes_Clostridia_Clostridiales_Lachnospiraceae_u.b.
PAB
LAB
EAB
CG.1
CG.15
CG.437
CG.540
CG.541
PG.2
PG.38
PG.515
PG.528
PG.832
O46.
Time
wk1 wk5 wk10
0.000
0.005
0.010
0.015
0.020
0.025

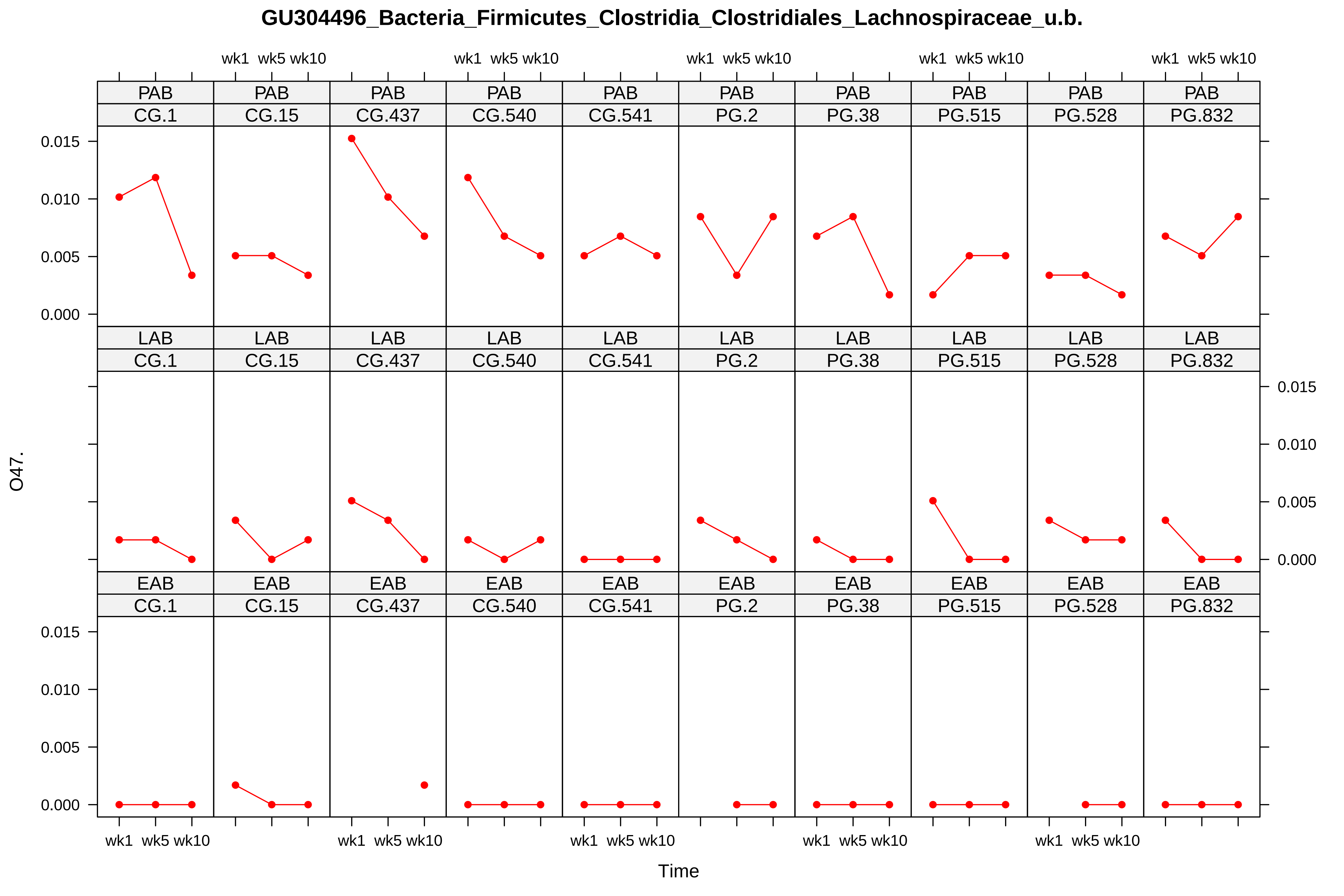

GU304496_Bacteria_Firmicutes_Clostridia_Clostridiales_Lachnospiraceae_u.b.
PAB
LAB
EAB
CG.1
CG.15
CG.437
CG.540
CG.541
PG.2
PG.38
PG.515
PG.528
PG.832
0.000
0.005
0.010
0.015
wk1 wk5 wk10
O47.
Time

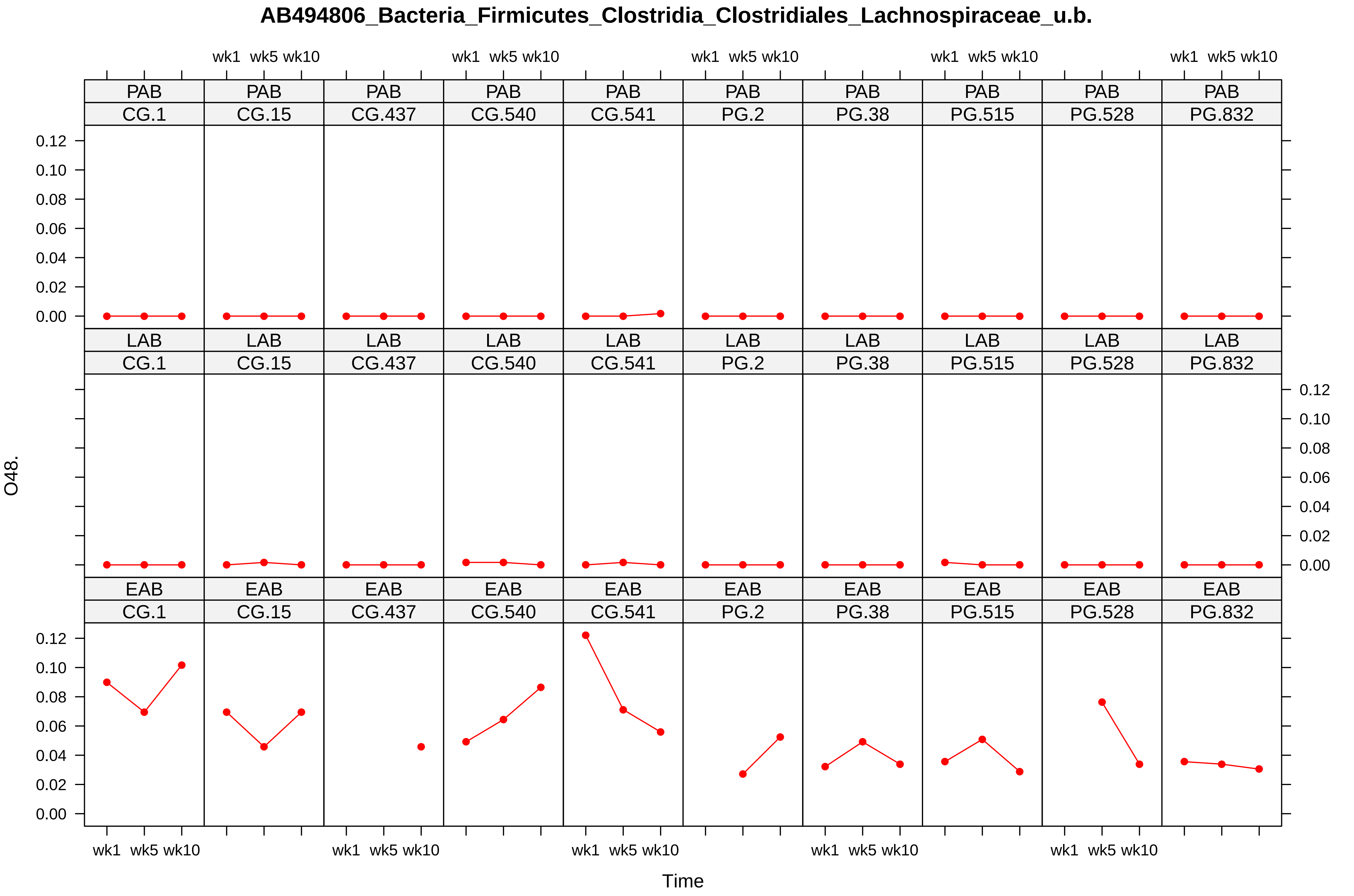

AB494806_Bacteria_Firmicutes_Clostridia_Clostridiales_Lachnospiraceae_u.b.
PAB
LAB
EAB
CG.1
CG.15
CG.437
CG.540
CG.541
PG.2
PG.38
PG.515
PG.528
PG.832
0.00
0.02
0.04
0.06
0.08
0.10
0.12
wk1 wk5 wk10
O48.
Time

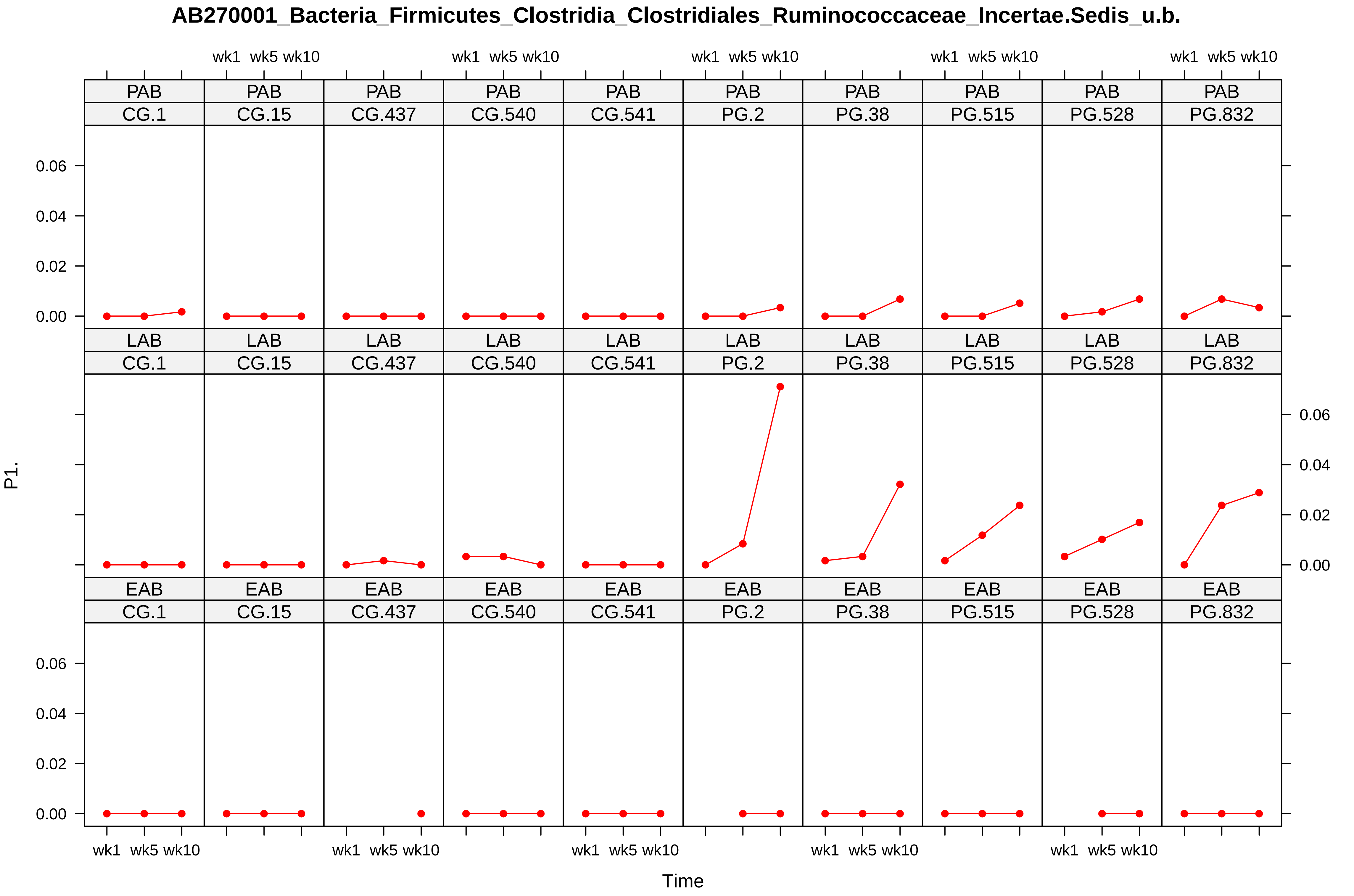
AB270001_Bacteria_Firmicutes_Clostridia_Clostridiales_Ruminococcaceae_Incertae.Sedis_u.b.
PAB
LAB
EAB
CG.1
CG.15
CG.437
CG.540
CG.541
PG.2
PG.38
PG.515
PG.528
PG.832
0.00
0.02
0.04
0.06
wk1 wk5 wk10
P1.
Time

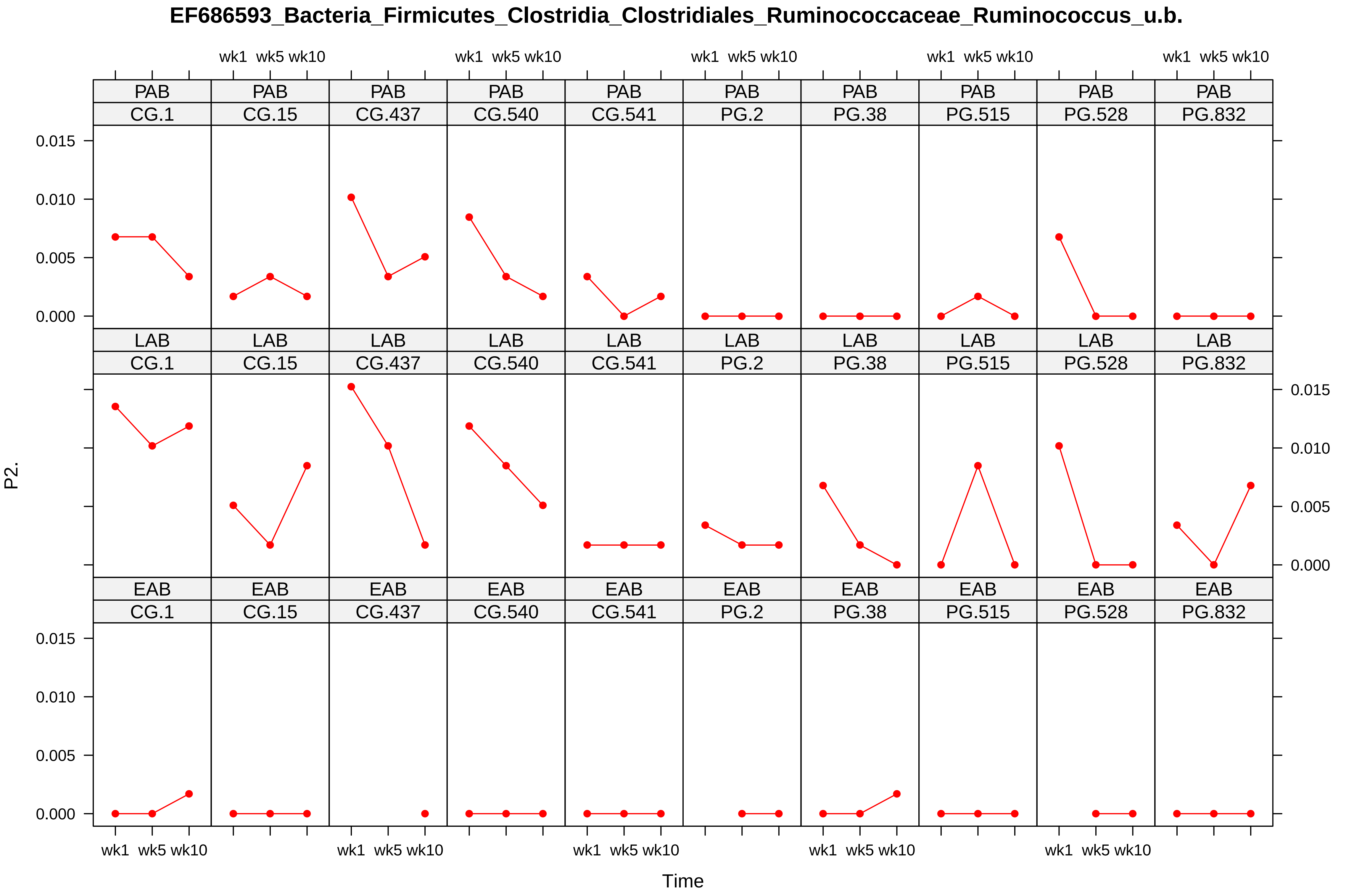

EF686593_Bacteria_Firmicutes_Clostridia_Clostridiales_Ruminococcaceae_Ruminococcus_u.b.
PAB
LAB
EAB
CG.1
CG.15
CG.437
CG.540
CG.541
PG.2
PG.38
PG.515
PG.528
PG.832
0.000
0.005
0.010
0.015
wk1 wk5 wk10
P2.
Time

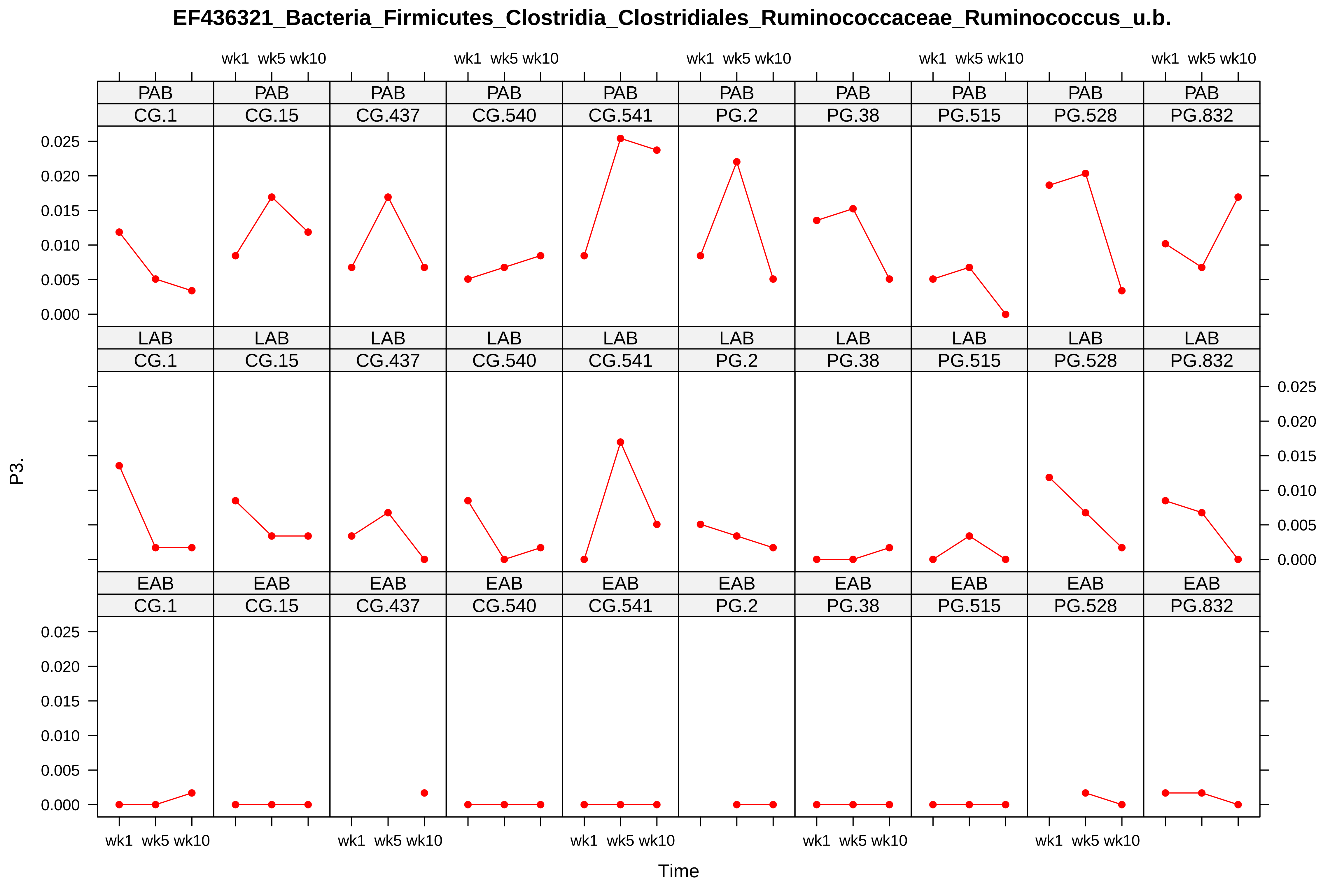
EF436321_Bacteria_Firmicutes_Clostridia_Clostridiales_Ruminococcaceae_Ruminococcus_u.b.
PAB
LAB
EAB
CG.1
CG.15
CG.437
CG.540
CG.541
PG.2
PG.38
PG.515
PG.528
PG.832
0.000
0.005
0.010
0.015
0.020
0.025
wk1 wk5 wk10
P3.
Time

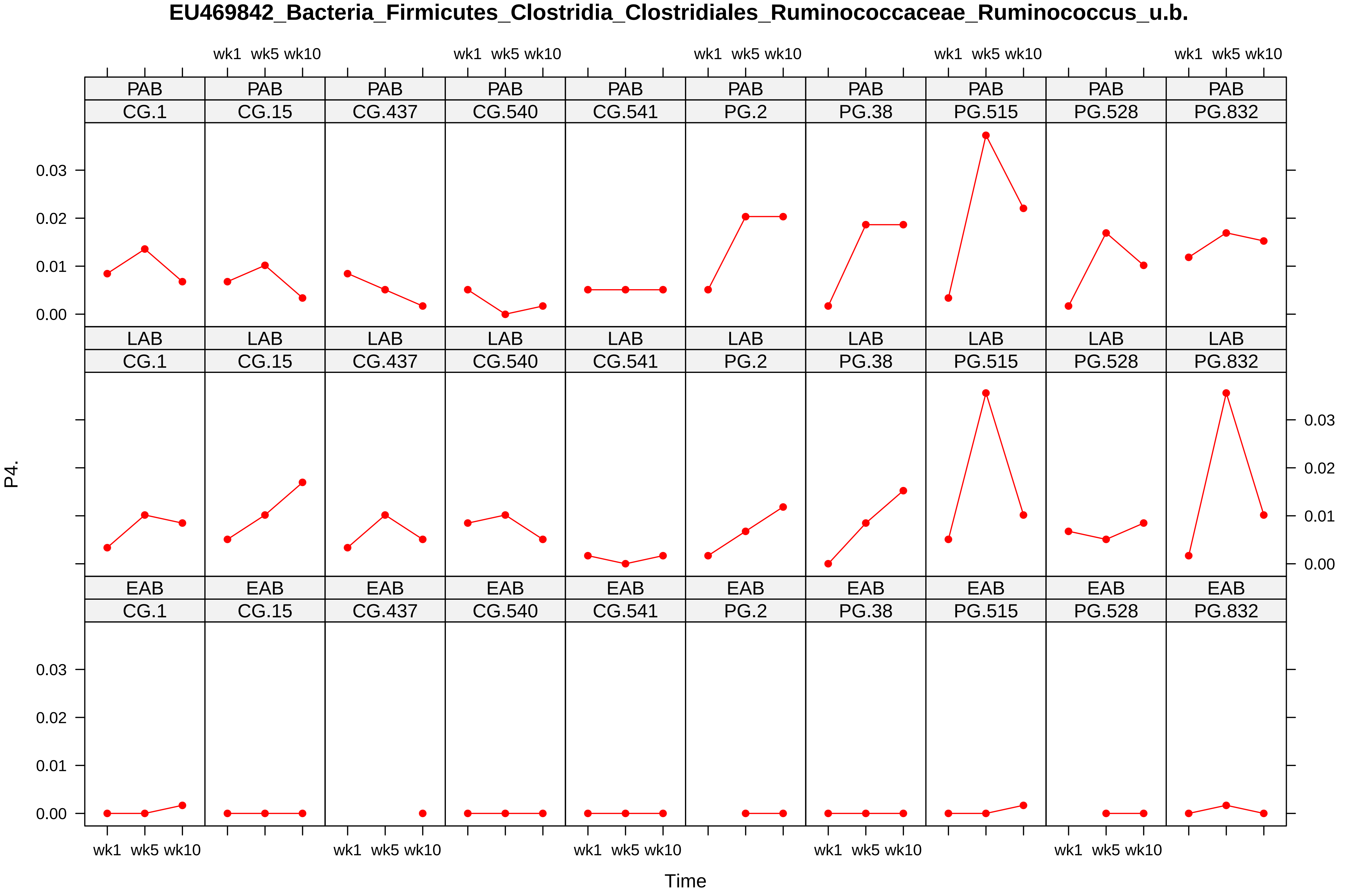

EU469842_Bacteria_Firmicutes_Clostridia_Clostridiales_Ruminococcaceae_Ruminococcus_u.b.
PAB CG.1
PAB CG.15
PAB CG.437
PAB CG.540
PAB CG.541
PAB PG.2
PAB PG.38
PAB PG.515
PAB PG.528
PAB PG.832
LAB CG.1
LAB CG.15
LAB CG.437
LAB CG.540
LAB CG.541
LAB PG.2
LAB PG.38
LAB PG.515
LAB PG.528
LAB PG.832
EAB CG.1
EAB CG.15
EAB CG.437
EAB CG.540
EAB CG.541
EAB PG.2
EAB PG.38
EAB PG.515
EAB PG.528
EAB PG.832
0.00
0.01
0.02
0.03
wk1 wk5 wk10
P4.
Time

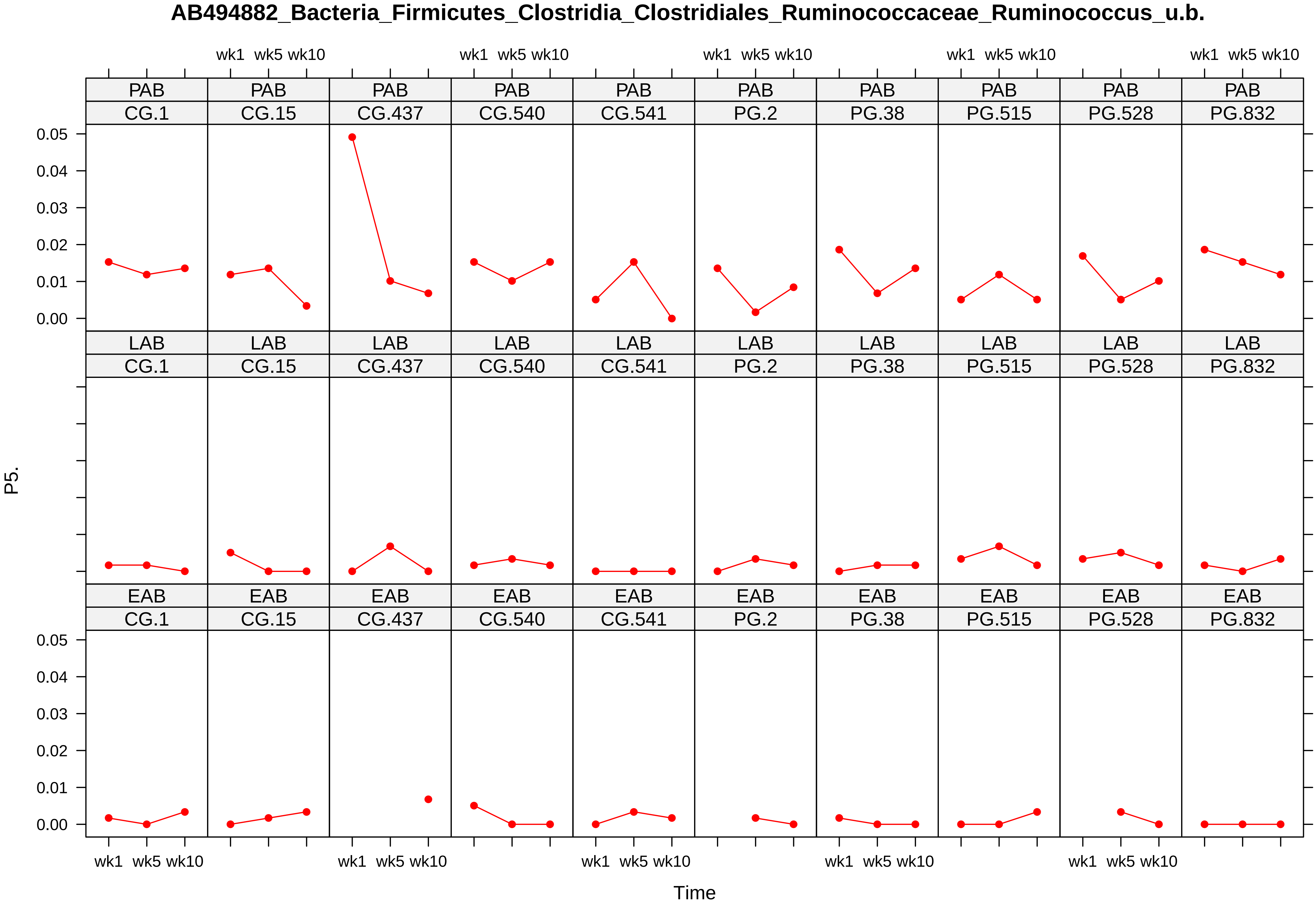
AB494882_Bacteria_Firmicutes_Clostridia_Clostridiales_Ruminococcaceae_Ruminococcus_u.b.
PAB
LAB
EAB
CG.1
CG.15
CG.437
CG.540
CG.541
PG.2
PG.38
PG.515
PG.528
PG.832
wk1
wk5
wk10
0.00
0.01
0.02
0.03
0.04
0.05
P5.
Time

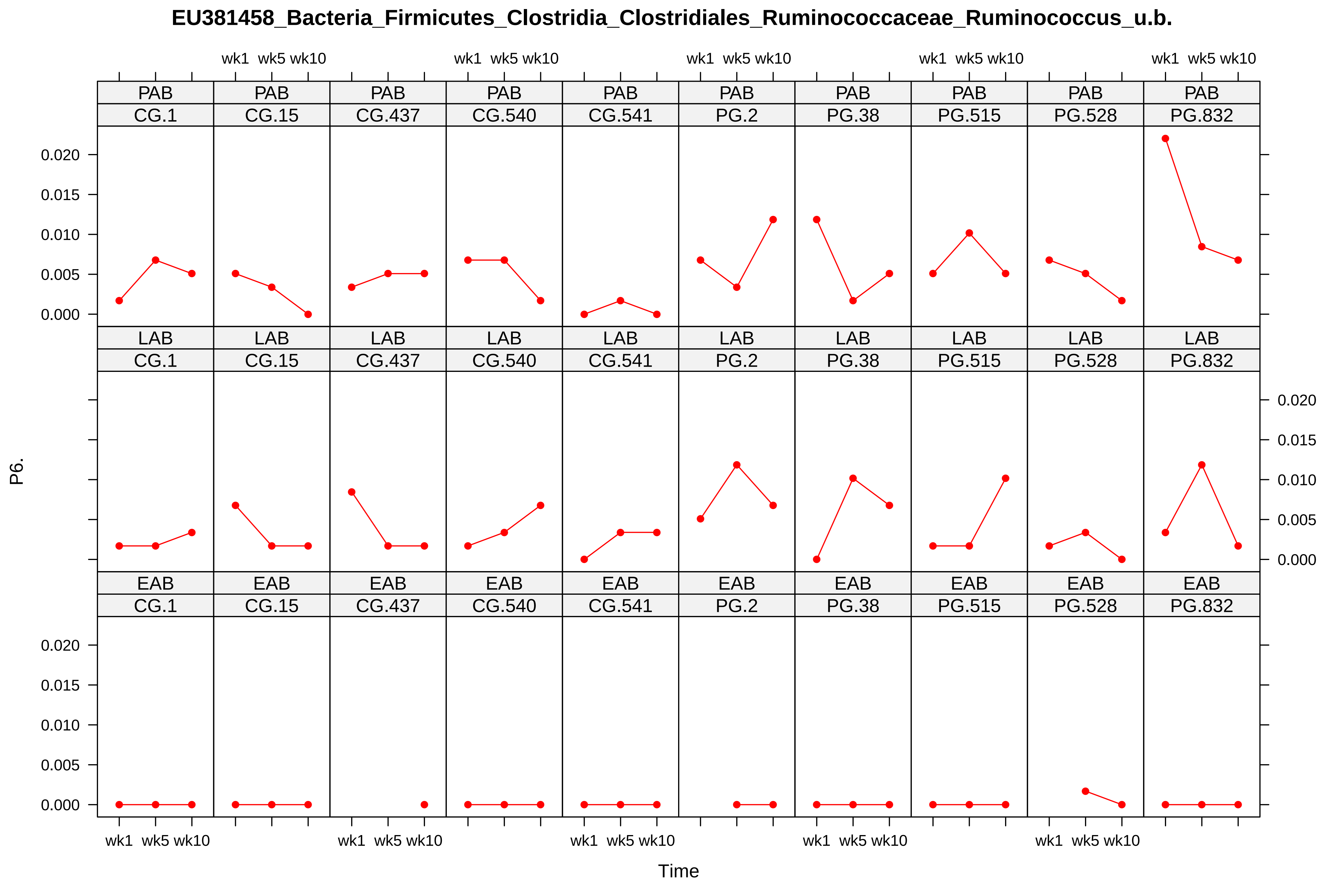

EU381458_Bacteria_Firmicutes_Clostridia_Clostridiales_Ruminococcaceae_Ruminococcus_u.b.
PAB
LAB
EAB
CG.1
CG.15
CG.437
CG.540
CG.541
PG.2
PG.38
PG.515
PG.528
PG.832
0.000
0.005
0.010
0.015
0.020
wk1 wk5 wk10
P6.
Time

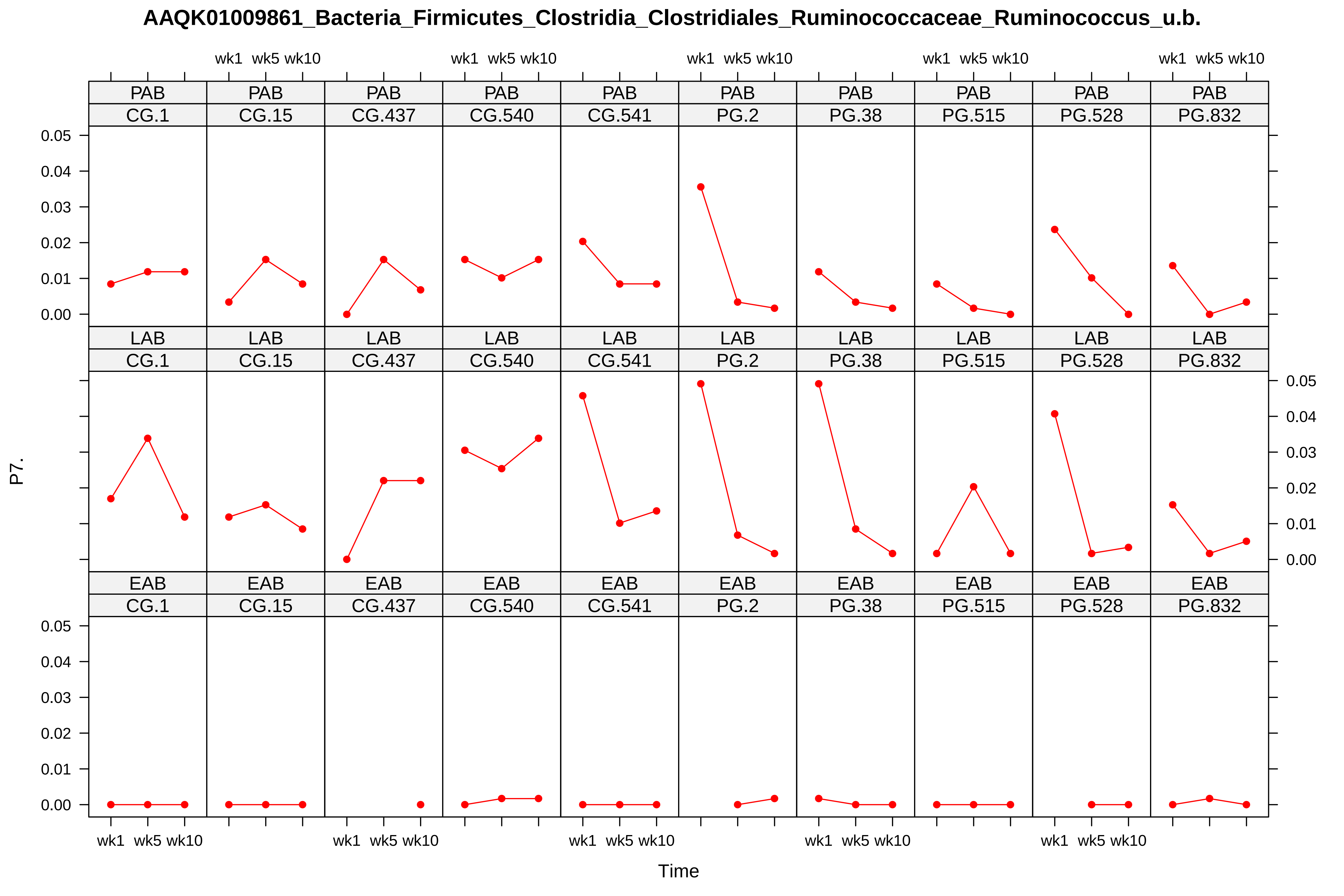
AAQK01009861_Bacteria_Firmicutes_Clostridia_Clostridiales_Ruminococcaceae_Ruminococcus_u.b.
PAB
LAB
EAB
CG.1
CG.15
CG.437
CG.540
CG.541
PG.2
PG.38
PG.515
PG.528
PG.832
wk1 wk5 wk10
0.00
0.01
0.02
0.03
0.04
0.05
P7.
Time

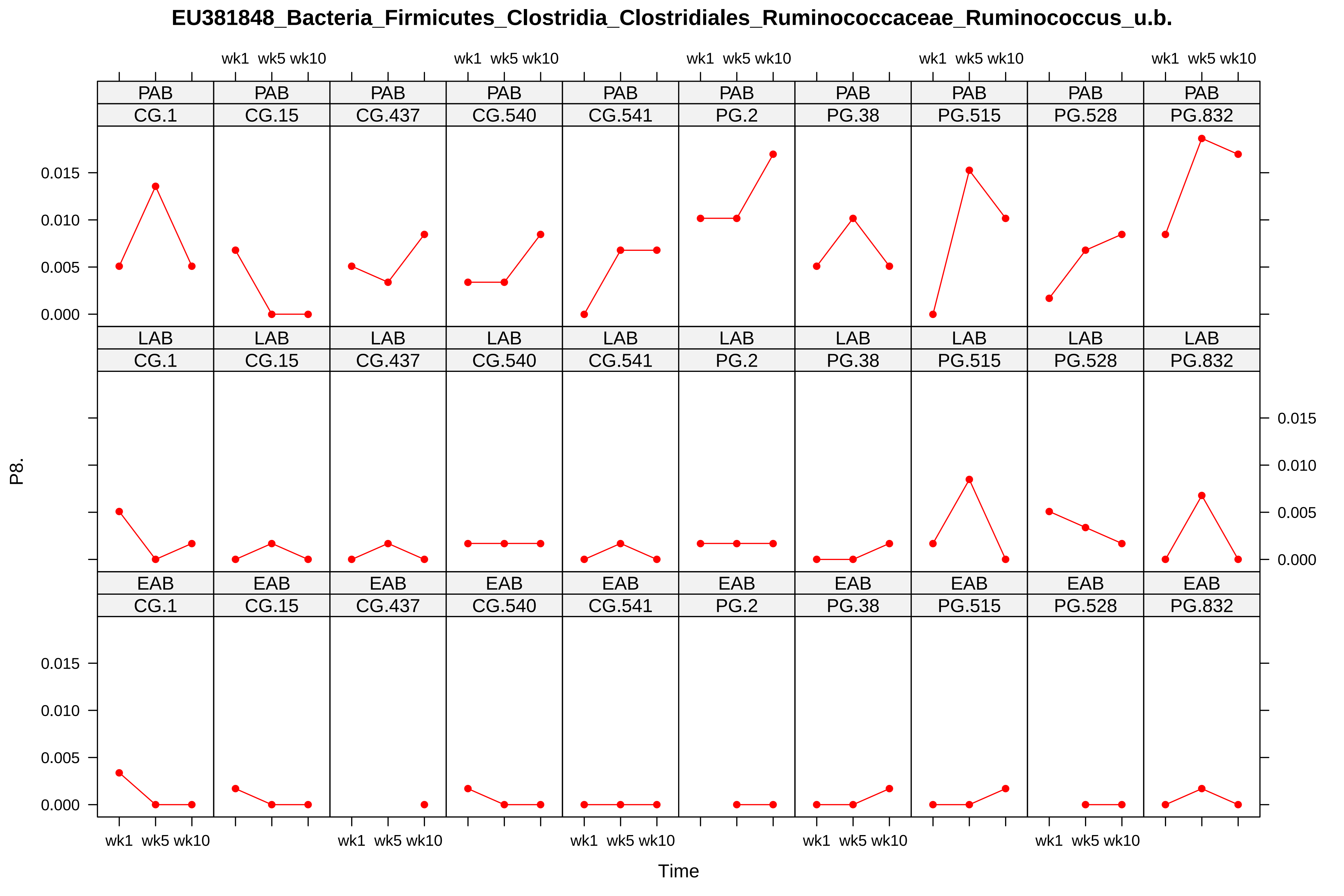
EU381848_Bacteria_Firmicutes_Clostridia_Clostridiales_Ruminococcaceae_Ruminococcus_u.b.
PAB
LAB
EAB
CG.1
CG.15
CG.437
CG.540
CG.541
PG.2
PG.38
PG.515
PG.528
PG.832
0.000
0.005
0.010
0.015
wk1 wk5 wk10
P8.
Time

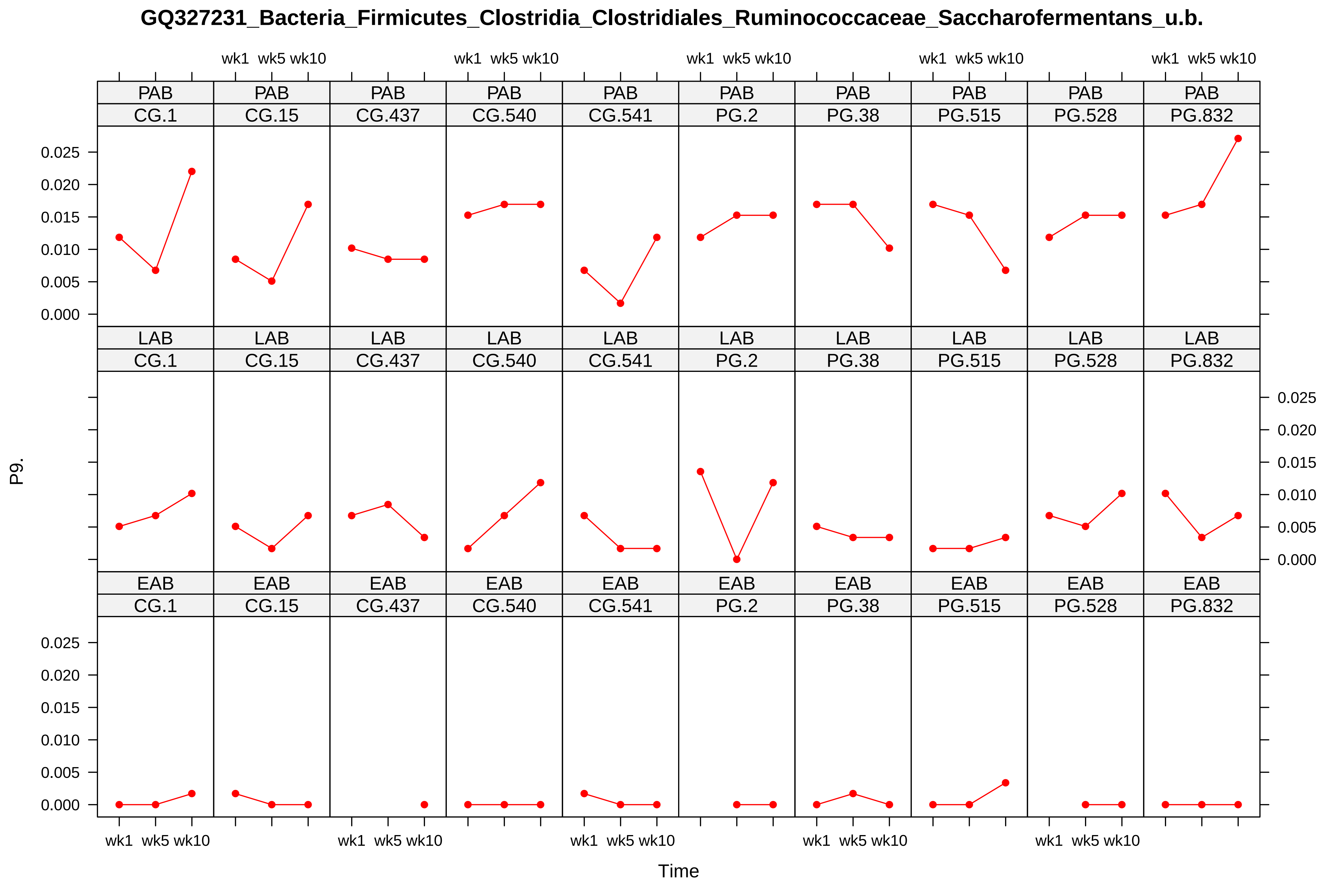
GQ327231_Bacteria_Firmicutes_Clostridia_Clostridiales_Ruminococcaceae_Saccharofermentans_u.b.
PAB
LAB
EAB
CG.1
CG.15
CG.437
CG.540
CG.541
PG.2
PG.38
PG.515
PG.528
PG.832
0.000
0.005
0.010
0.015
0.020
0.025
wk1 wk5 wk10
P9.
Time

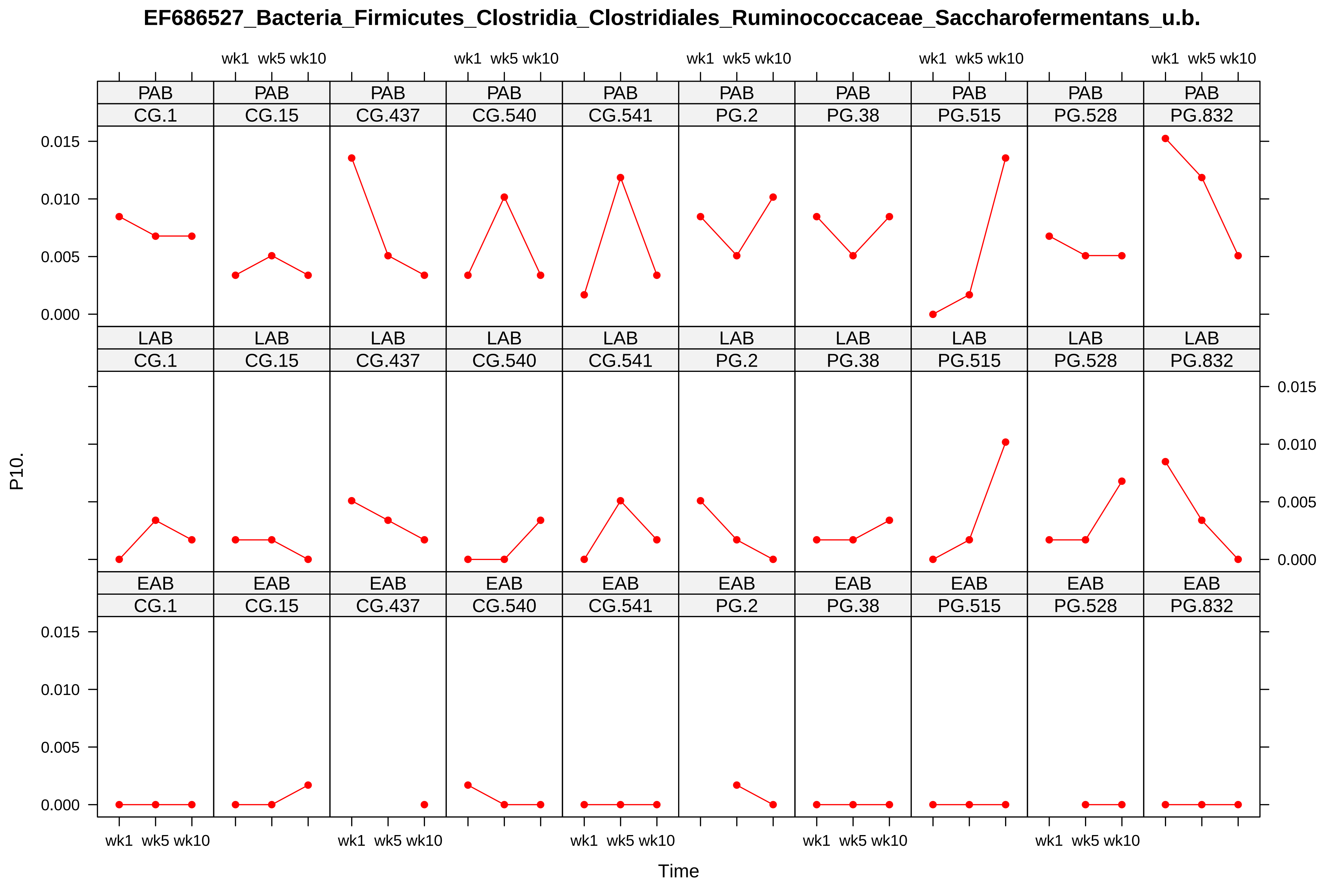
EF686527_Bacteria_Firmicutes_Clostridia_Clostridiales_Ruminococcaceae_Saccharofermentans_u.b.
PAB
LAB
EAB
CG.1
CG.15
CG.437
CG.540
CG.541
PG.2
PG.38
PG.515
PG.528
PG.832
wk1
wk5
wk10
0.000
0.005
0.010
0.015
P10.
Time

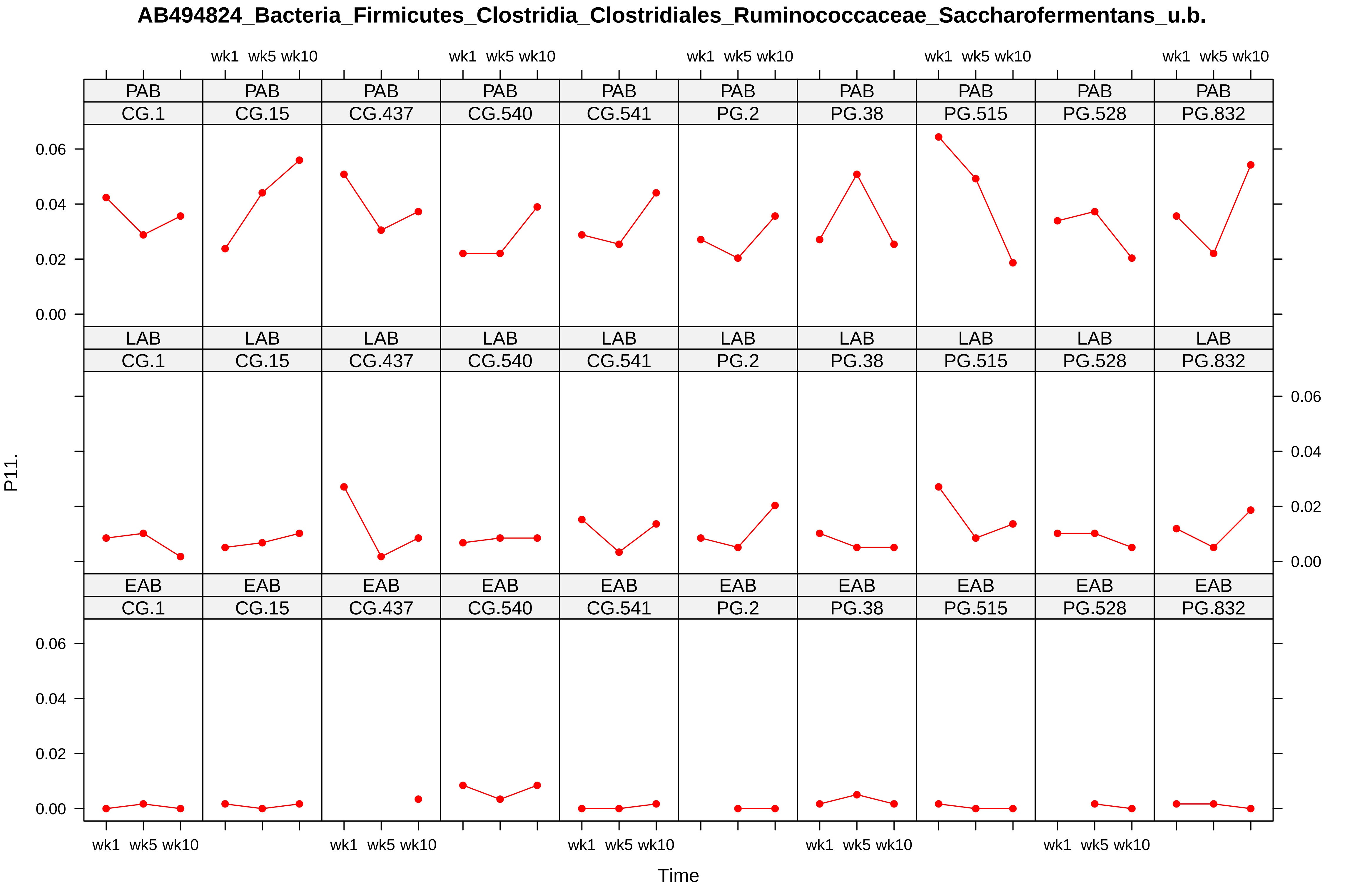

AB494824_Bacteria_Firmicutes_Clostridia_Clostridiales_Ruminococcaceae_Saccharofermentans_u.b.
PAB
LAB
EAB
CG.1
CG.15
CG.437
CG.540
CG.541
PG.2
PG.38
PG.515
PG.528
PG.832
0.00
0.02
0.04
0.06
wk1
wk5
wk10
P11.
Time

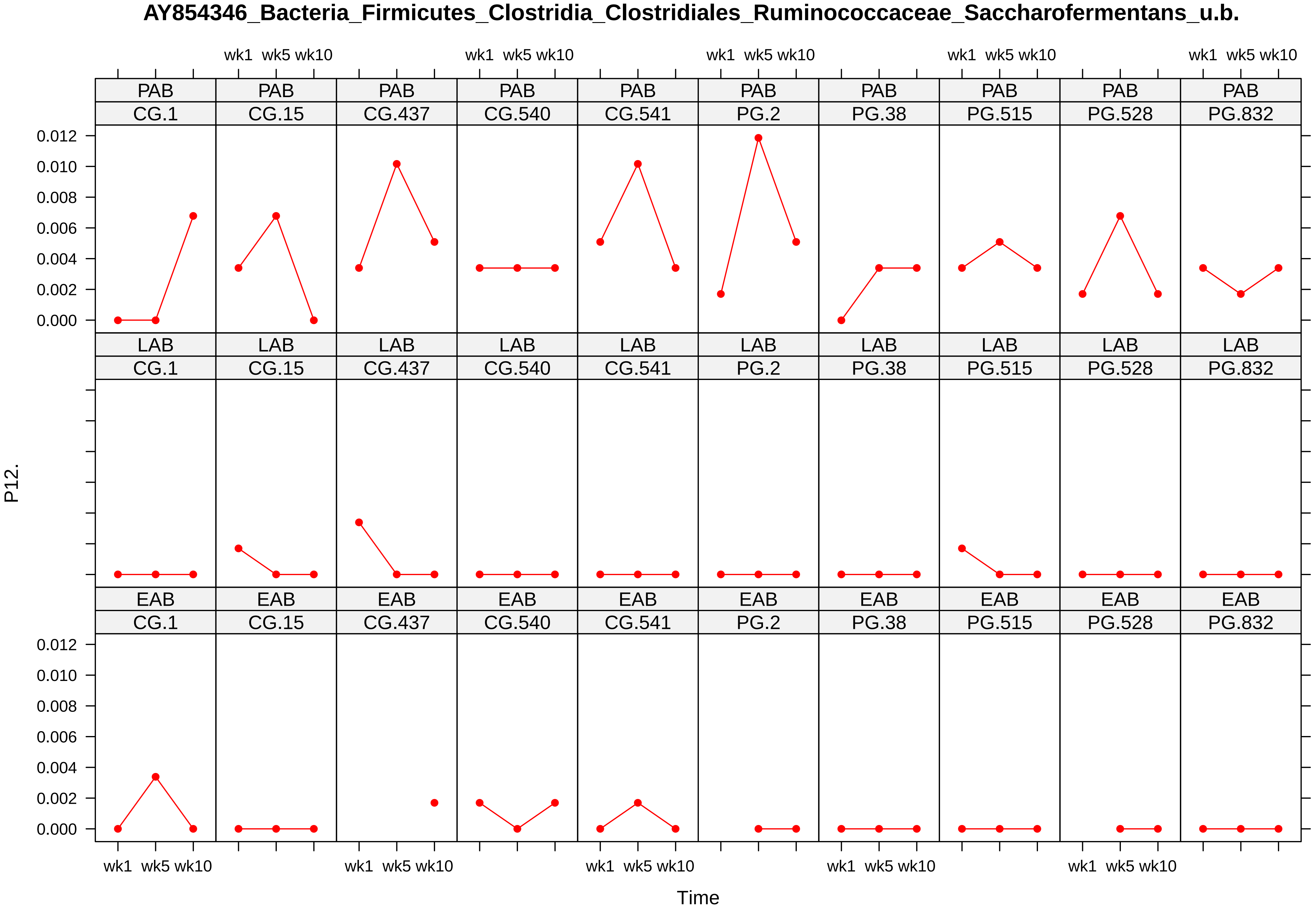
AY854346_Bacteria_Firmicutes_Clostridia_Clostridiales_Ruminococcaceae_Saccharofermentans_u.b.
PAB
CG.1
CG.15
CG.437
CG.540
CG.541
PG.2
PG.38
PG.515
PG.528
PG.832
LAB
EAB
wk1 wk5 wk10
0.000
0.002
0.004
0.006
0.008
0.010
0.012
P12.
Time

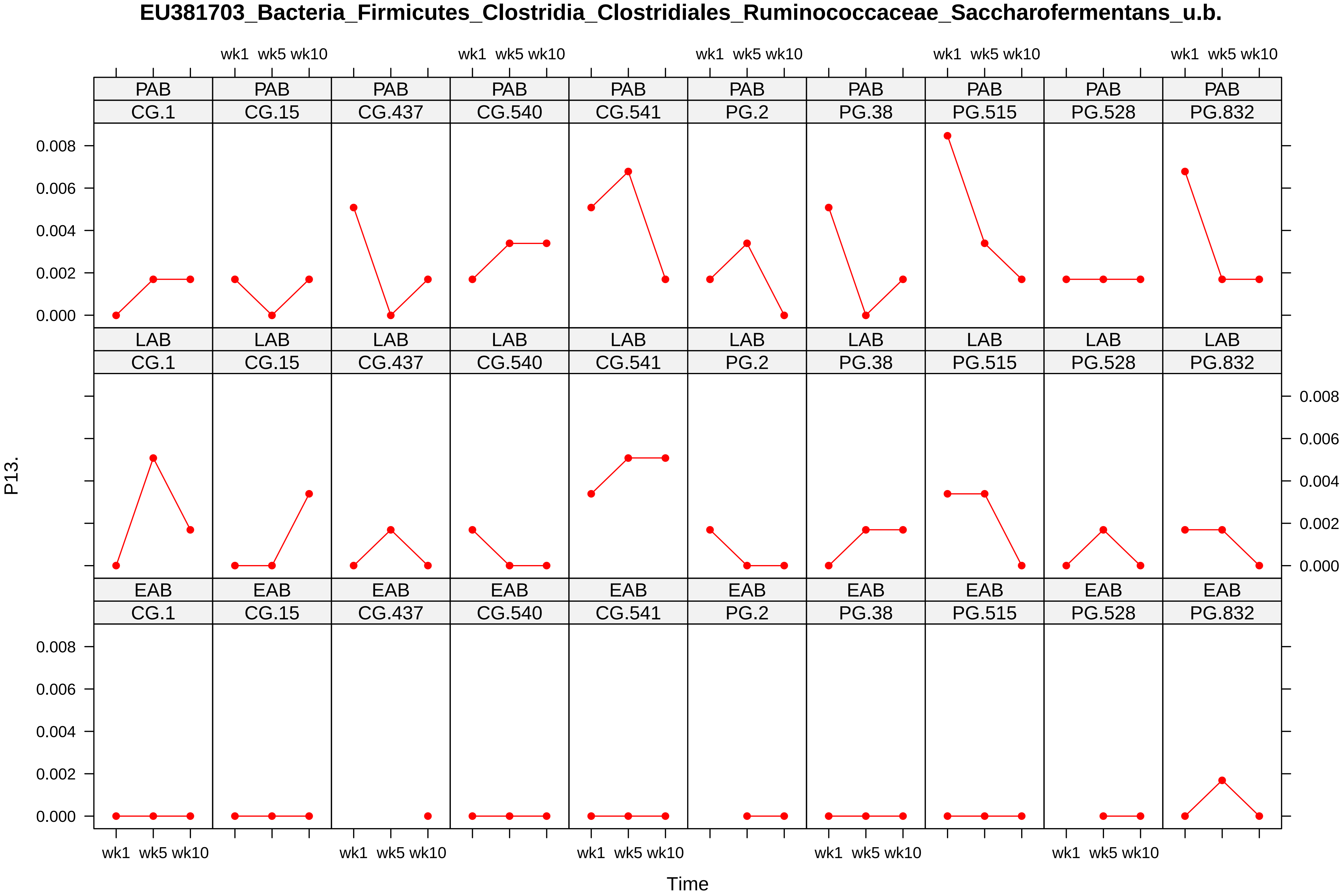
EU381703_Bacteria_Firmicutes_Clostridia_Clostridiales_Ruminococcaceae_Saccharofermentans_u.b.
PAB
CG.1
CG.15
CG.437
CG.540
CG.541
PG.2
PG.38
PG.515
PG.528
PG.832
LAB
EAB
P13.
0.000
0.002
0.004
0.006
0.008
wk1
wk5
wk10
Time

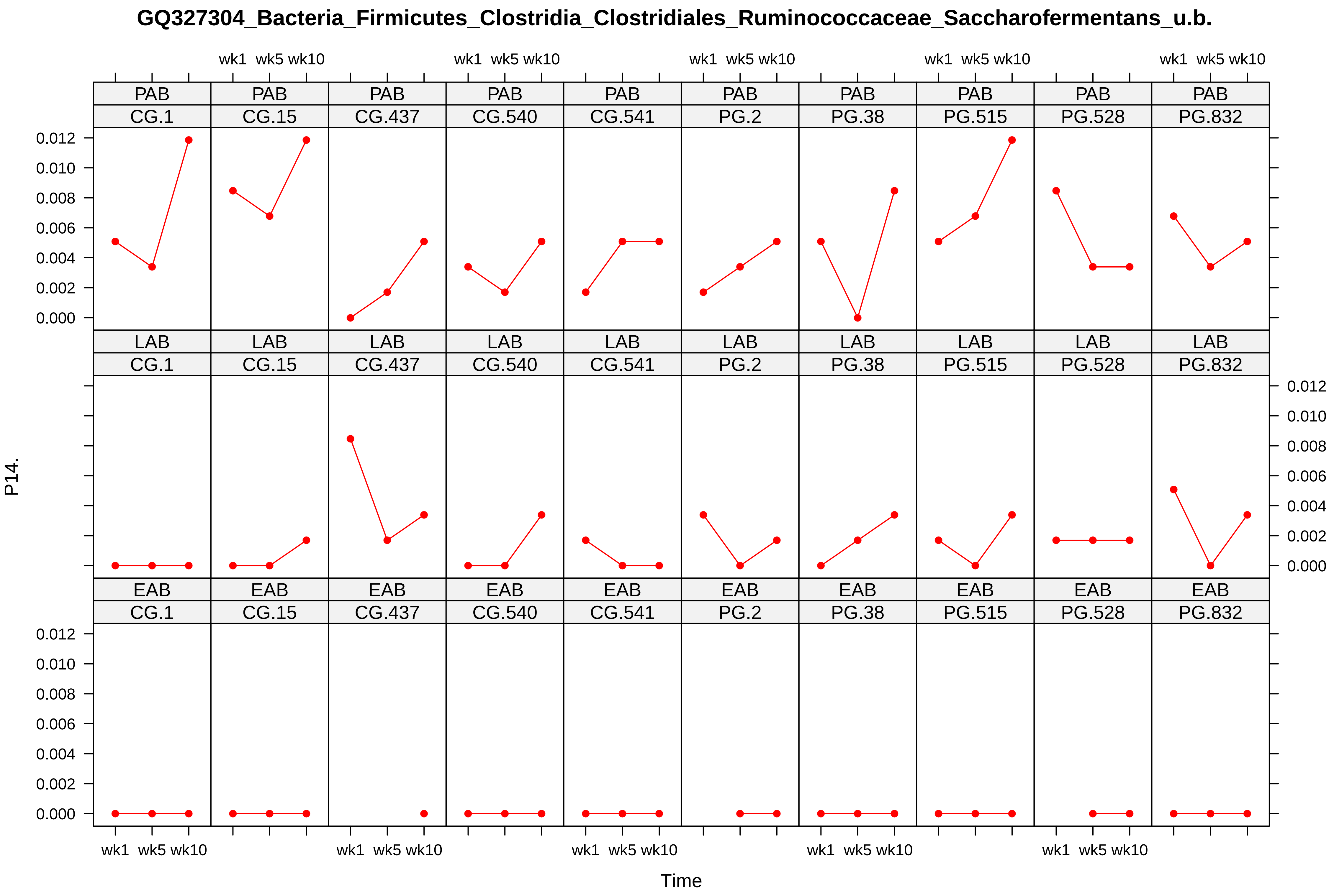
GQ327304_Bacteria_Firmicutes_Clostridia_Clostridiales_Ruminococcaceae_Saccharofermentans_u.b.
PAB
LAB
EAB
CG.1
CG.15
CG.437
CG.540
CG.541
PG.2
PG.38
PG.515
PG.528
PG.832
wk1 wk5 wk10
0.000
0.002
0.004
0.006
0.008
0.010
0.012
P14.
Time

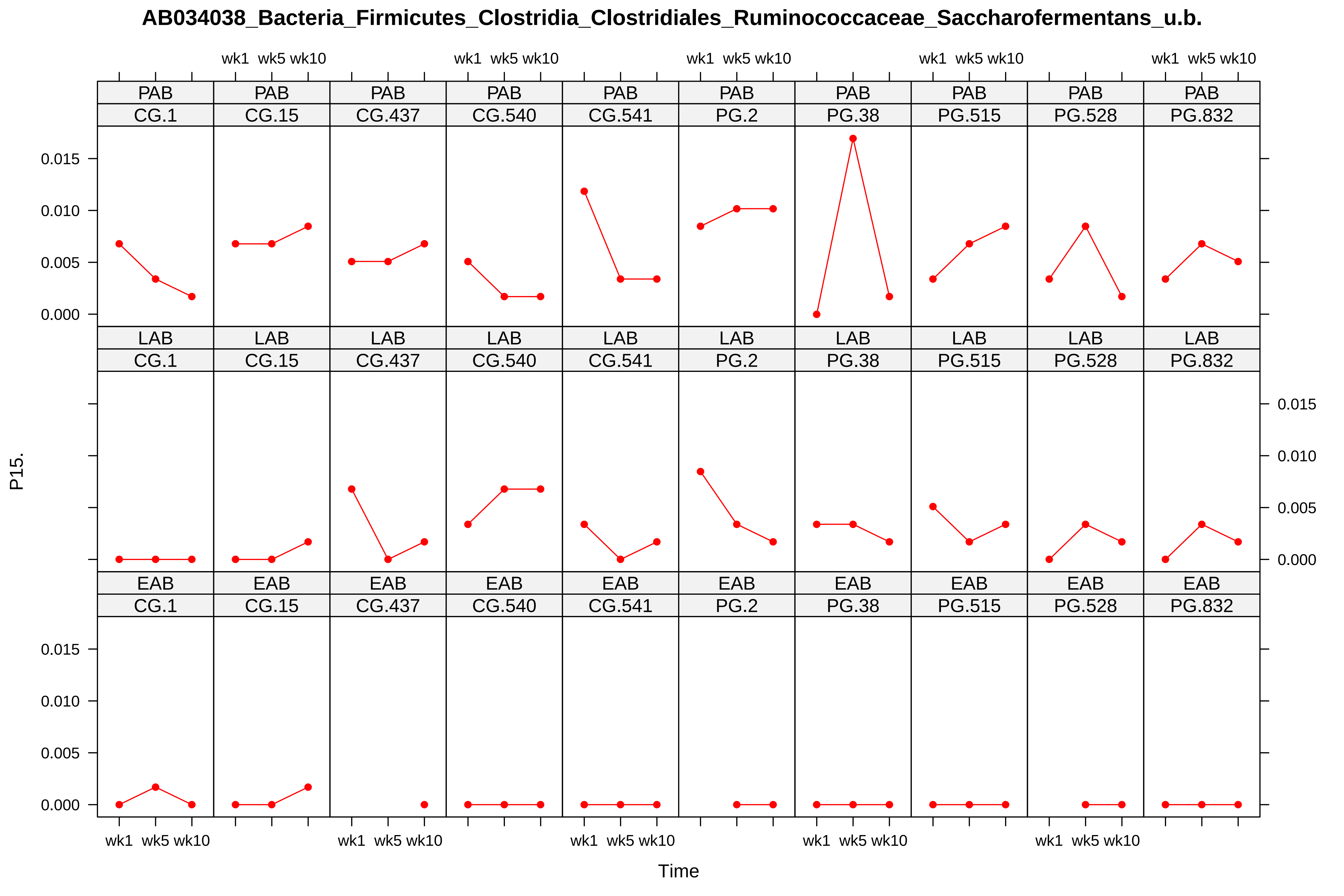
AB034038_Bacteria_Firmicutes_Clostridia_Clostridiales_Ruminococcaceae_Saccharofermentans_u.b.
PAB
LAB
EAB
CG.1
CG.15
CG.437
CG.540
CG.541
PG.2
PG.38
PG.515
PG.528
PG.832
0.000
0.005
0.010
0.015
wk1 wk5 wk10
P15.
Time

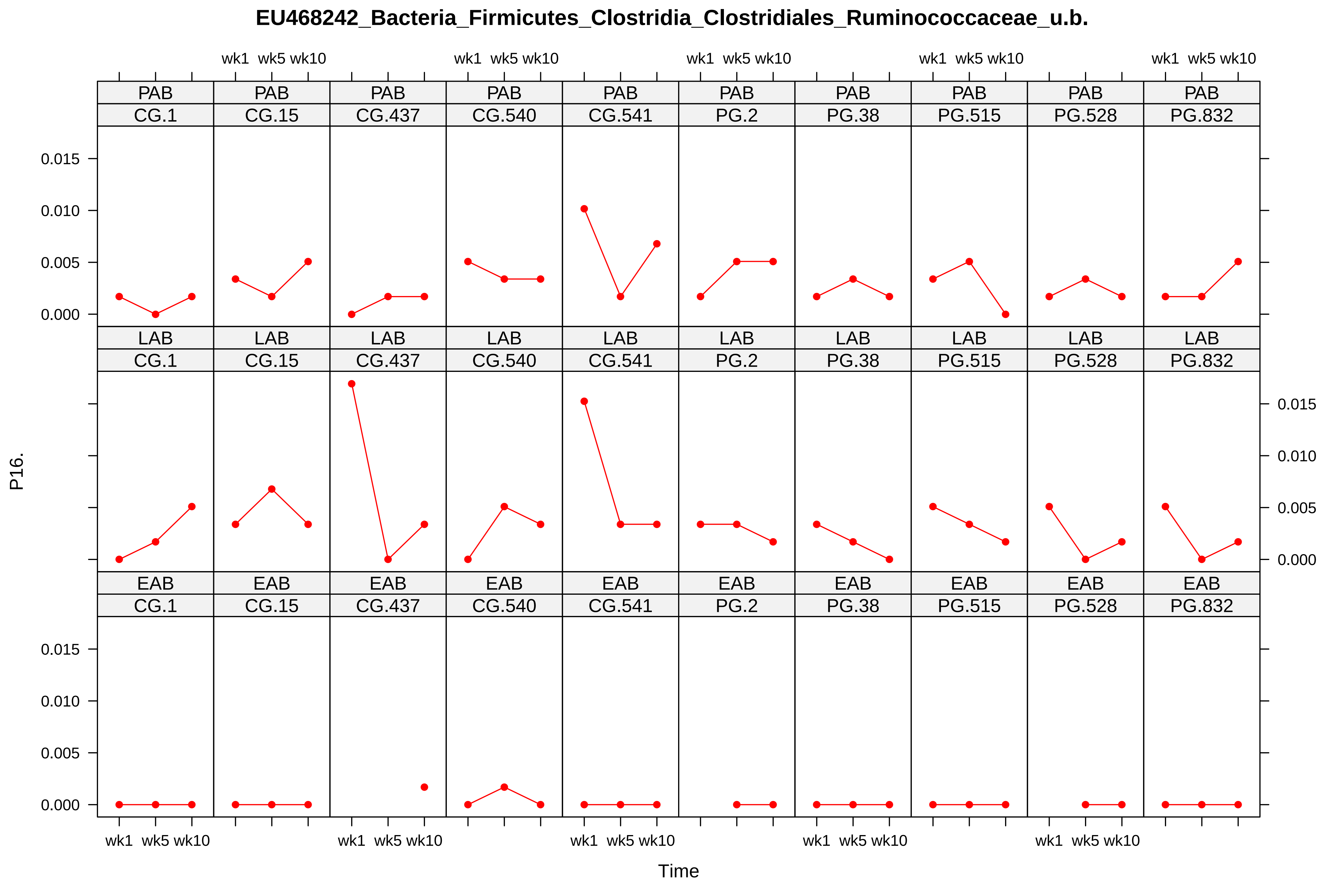
EU468242_Bacteria_Firmicutes_Clostridia_Clostridiales_Ruminococcaceae_u.b.
PAB
LAB
EAB
CG.1
CG.15
CG.437
CG.540
CG.541
PG.2
PG.38
PG.515
PG.528
PG.832
0.000
0.005
0.010
0.015
wk1 wk5 wk10
P16.
Time

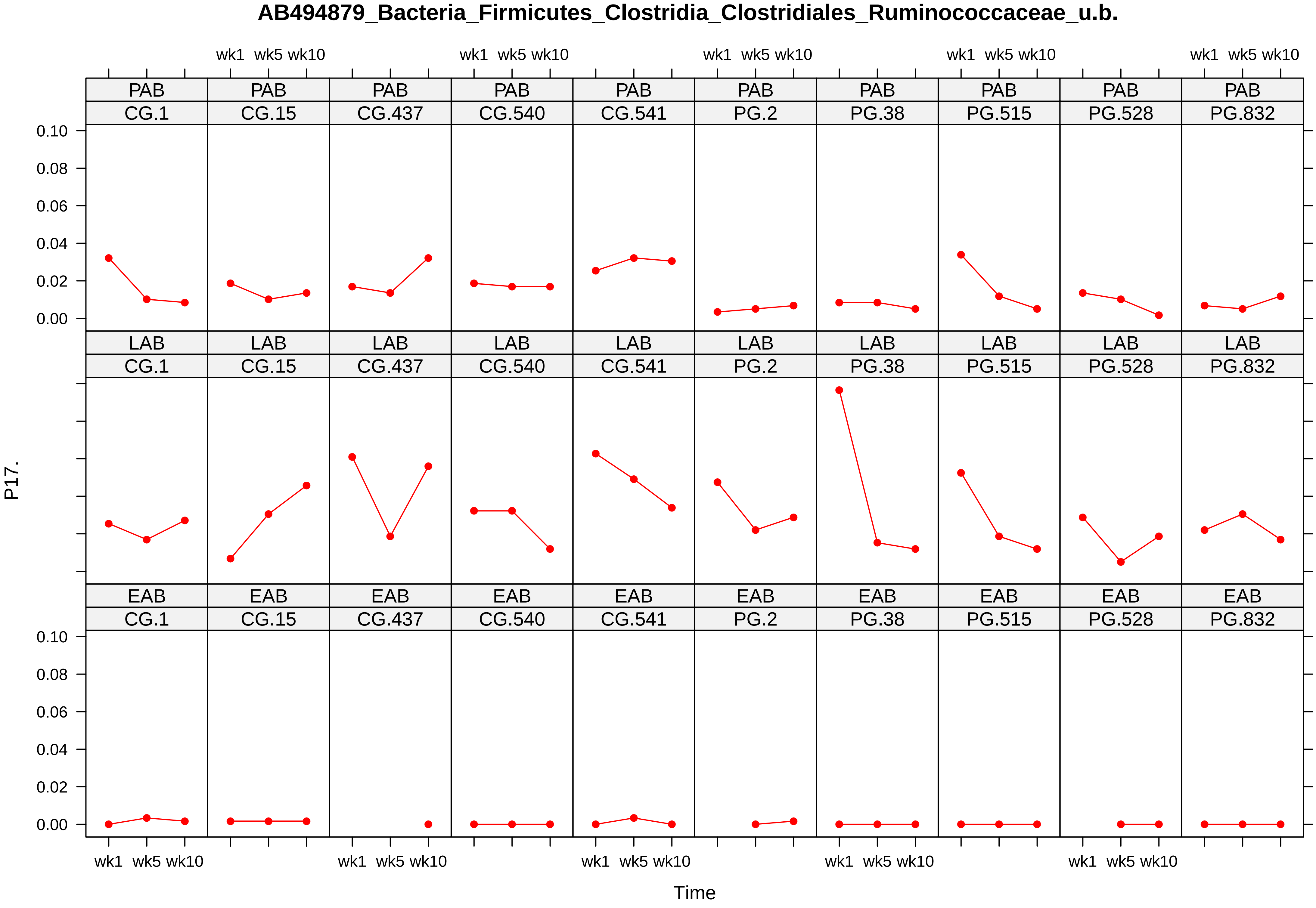
AB494879_Bacteria_Firmicutes_Clostridia_Clostridiales_Ruminococcaceae_u.b.
PAB
LAB
EAB
CG.1
CG.15
CG.437
CG.540
CG.541
PG.2
PG.38
PG.515
PG.528
PG.832
0.00
0.02
0.04
0.06
0.08
0.10
wk1
wk5
wk10
P17.
Time

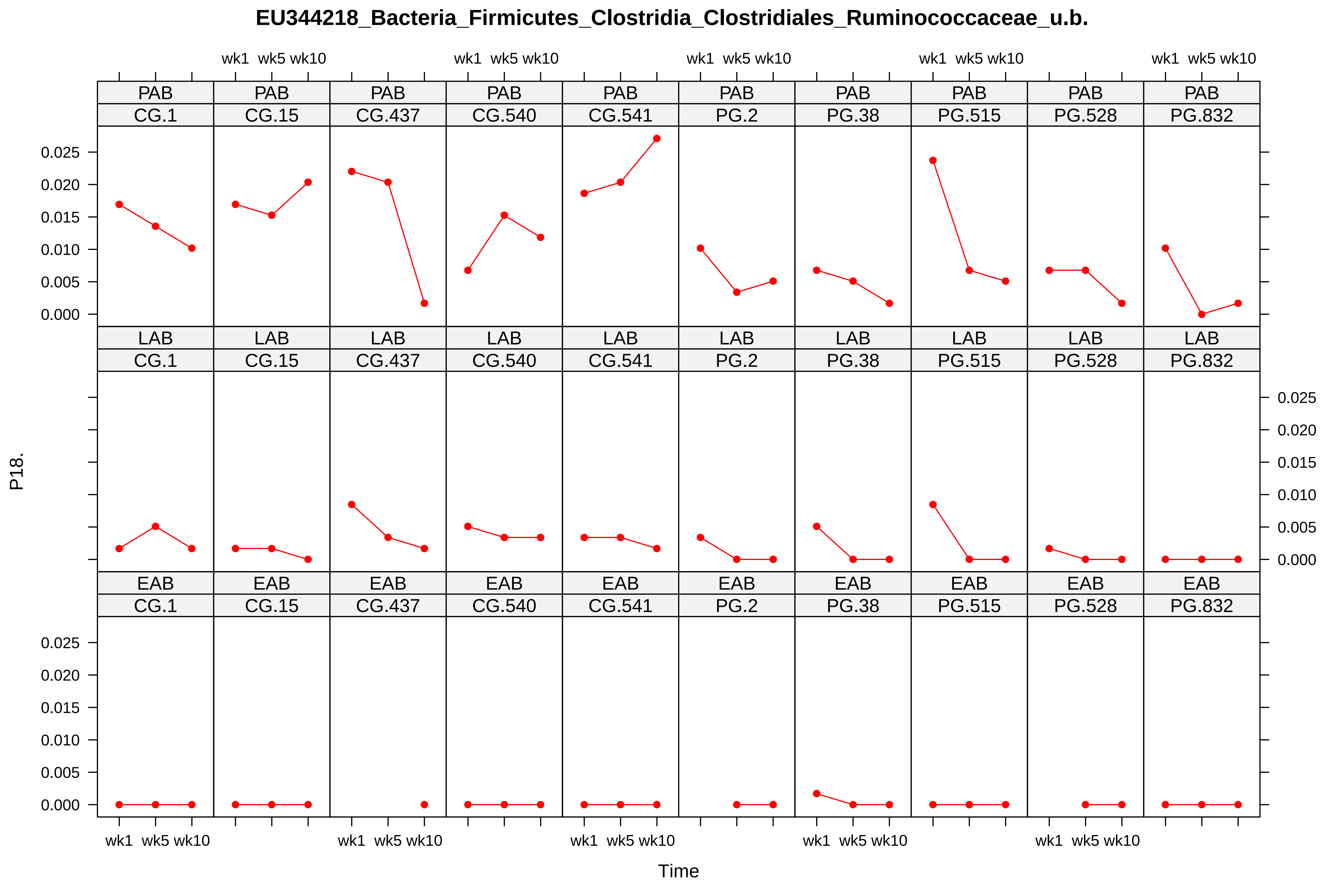
EU344218_Bacteria_Firmicutes_Clostridia_Clostridiales_Ruminococcaceae_u.b.
PAB
LAB
EAB
CG.1
CG.15
CG.437
CG.540
CG.541
PG.2
PG.38
PG.515
PG.528
PG.832
0.000
0.005
0.010
0.015
0.020
0.025
wk1 wk5 wk10
P18.
Time

EU381706_Bacteria_Firmicutes_Clostridia_Clostridiales_Ruminococcaceae_u.b.

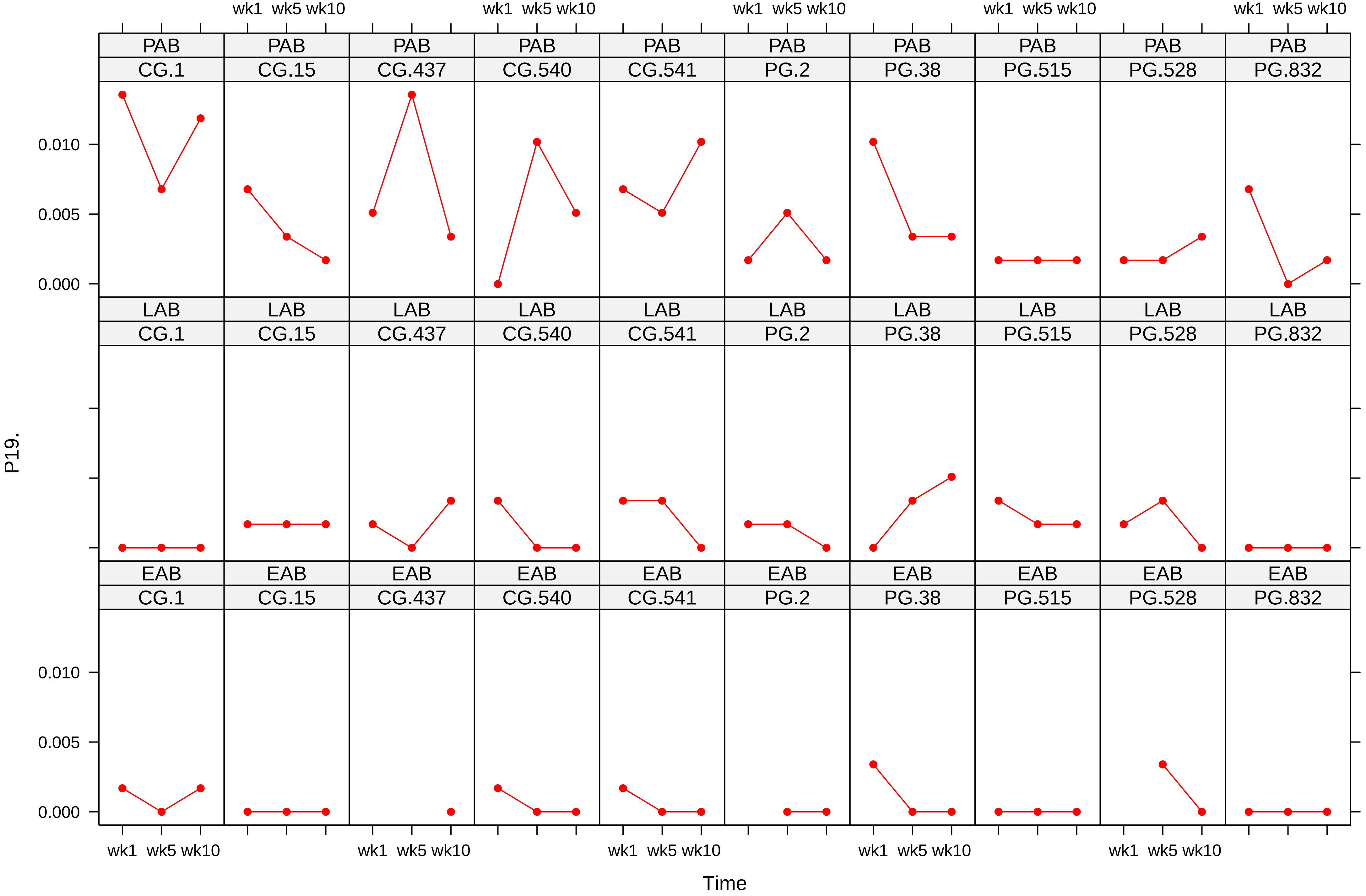

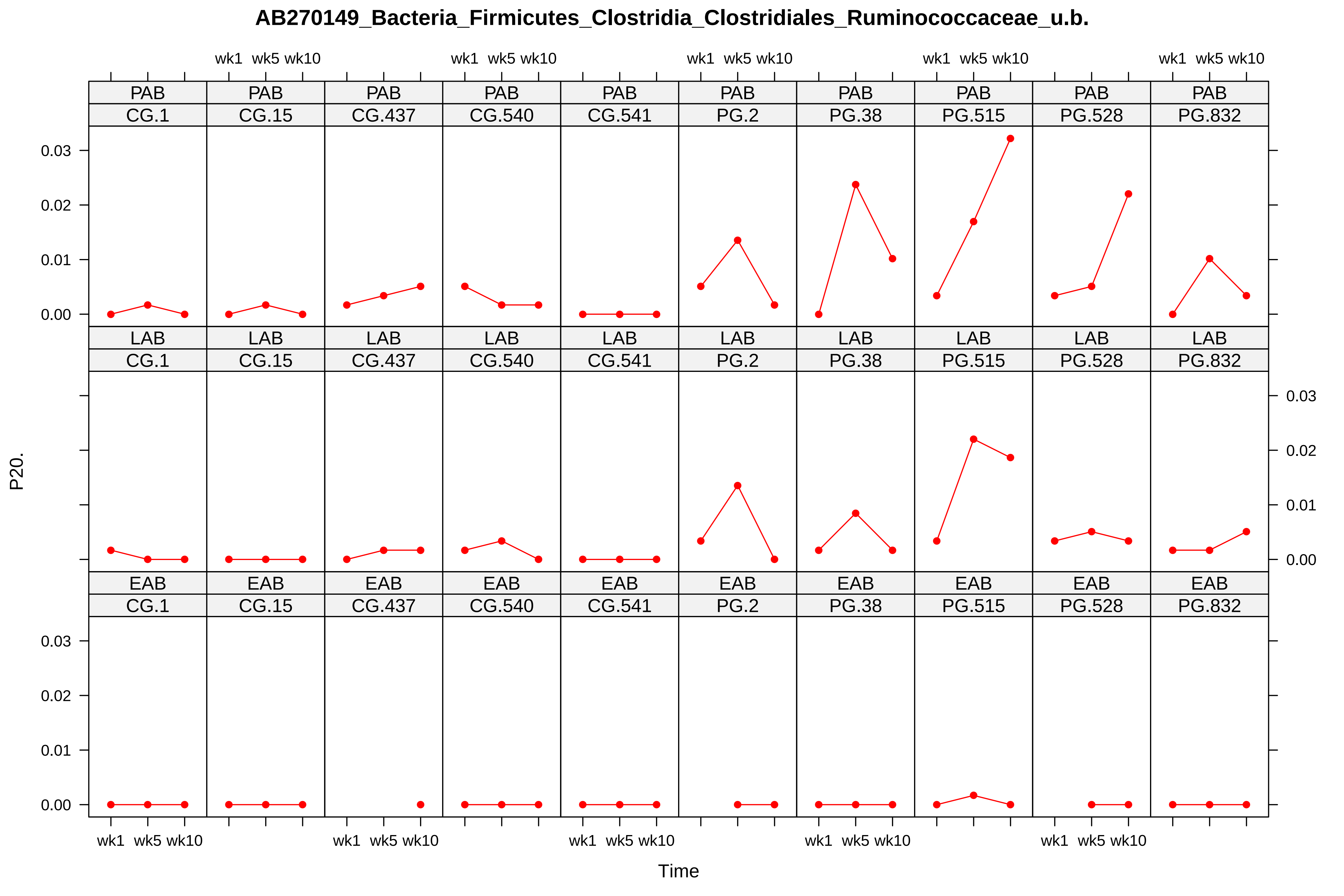

AB270149_Bacteria_Firmicutes_Clostridia_Clostridiales_Ruminococcaceae_u.b.
PAB
LAB
EAB
CG.1
CG.15
CG.437
CG.540
CG.541
PG.2
PG.38
PG.515
PG.528
PG.832
0.00
0.01
0.02
0.03
wk1
wk5
wk10
P20.
Time

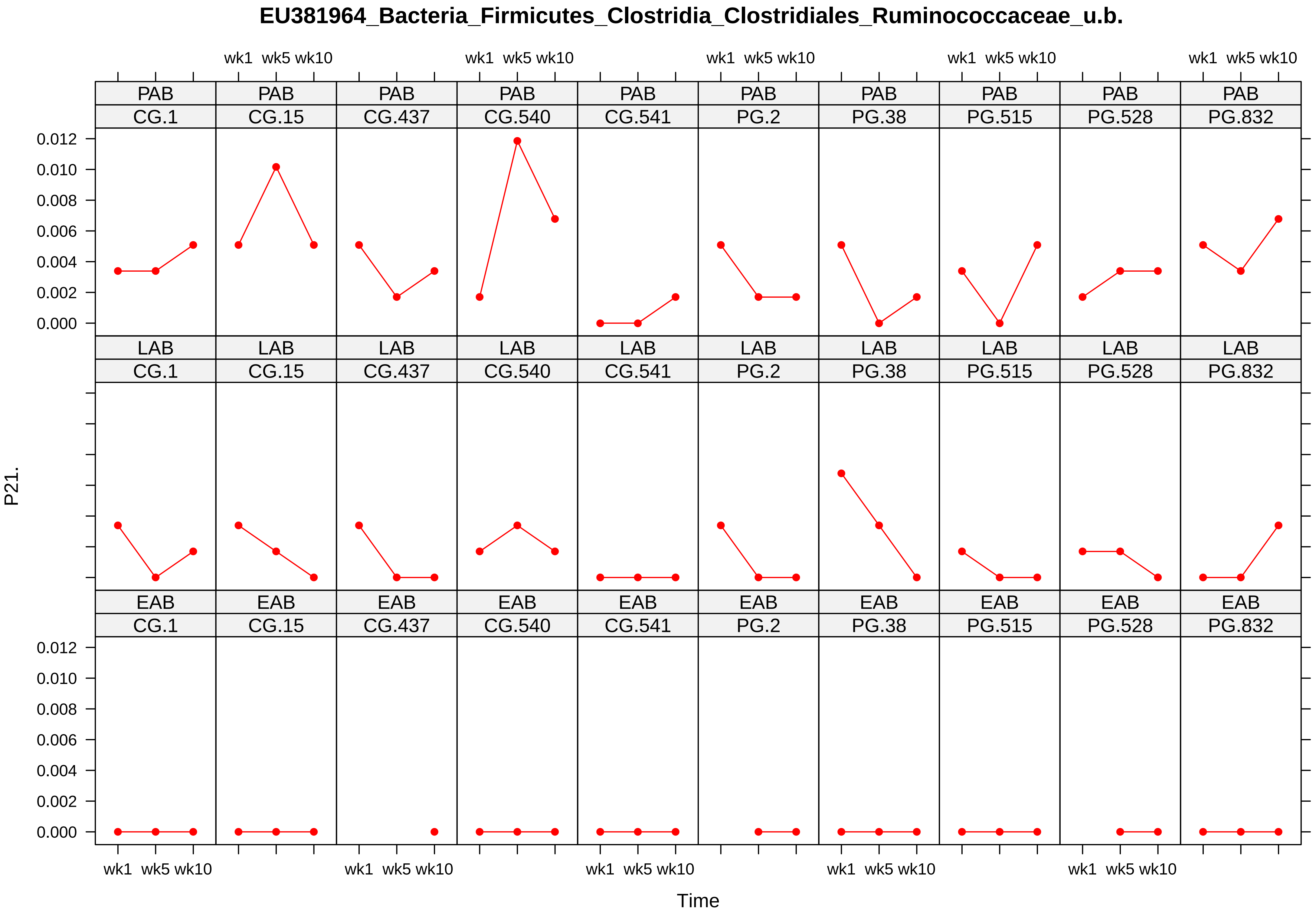

EU381964_Bacteria_Firmicutes_Clostridia_Clostridiales_Ruminococcaceae_u.b.
PAB
LAB
EAB
CG.1
CG.15
CG.437
CG.540
CG.541
PG.2
PG.38
PG.515
PG.528
PG.832
wk1 wk5 wk10
0.000
0.002
0.004
0.006
0.008
0.010
0.012
P21.
Time

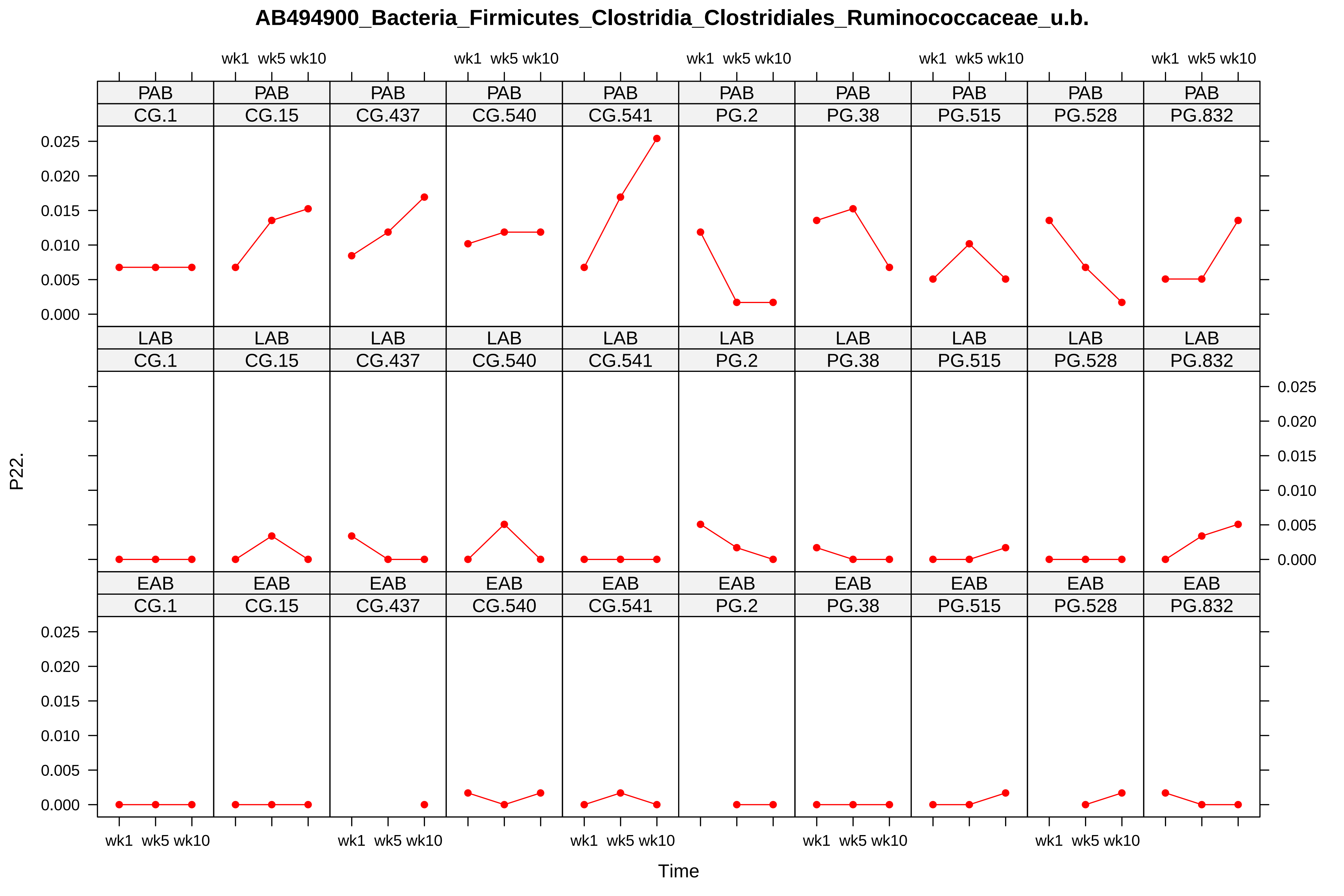

AB494900_Bacteria_Firmicutes_Clostridia_Clostridiales_Ruminococcaceae_u.b.
PAB
LAB
EAB
CG.1
CG.15
CG.437
CG.540
CG.541
PG.2
PG.38
PG.515
PG.528
PG.832
P22.
0.000
0.005
0.010
0.015
0.020
0.025
wk1
wk5
wk10
Time

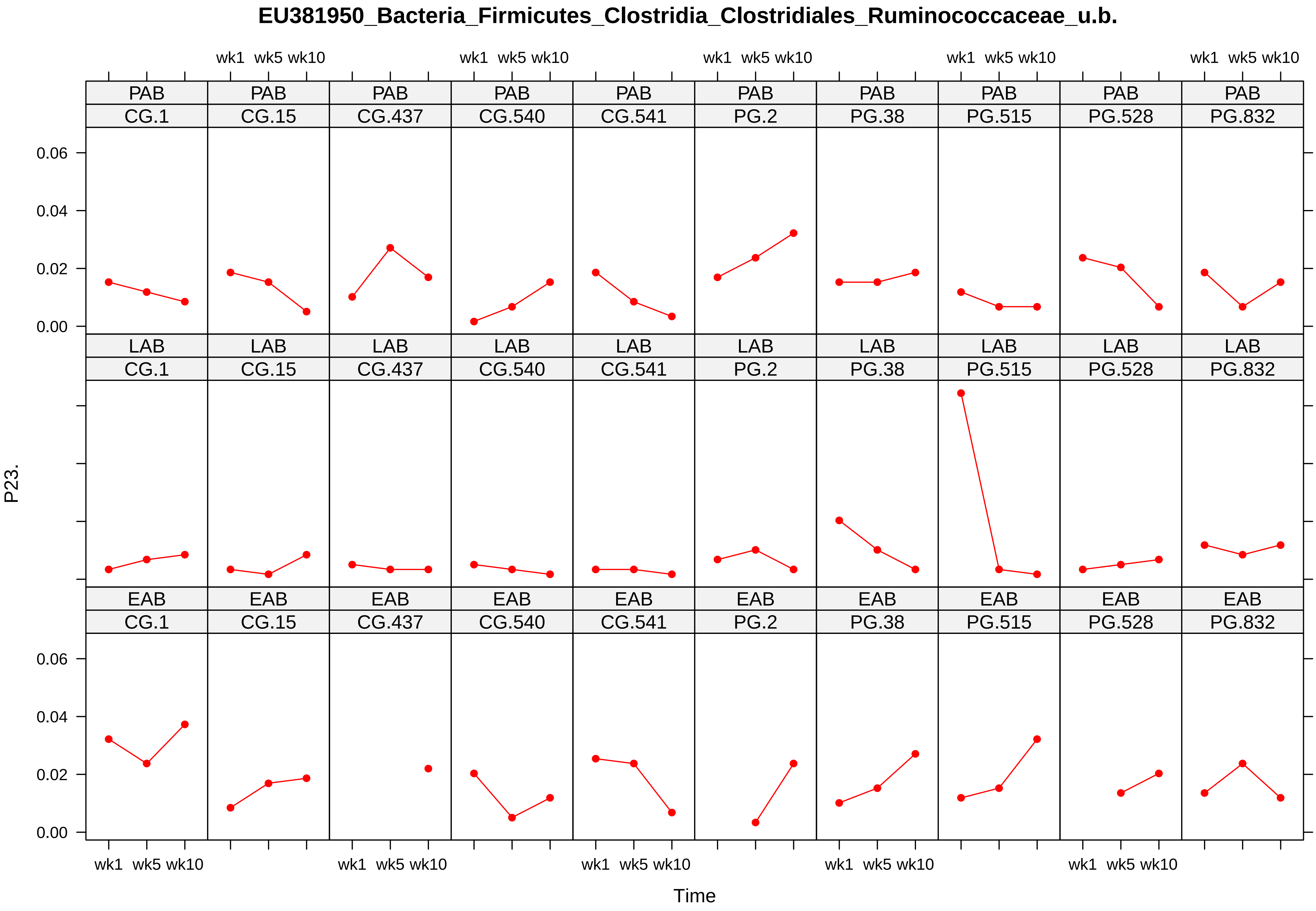

EU381950_Bacteria_Firmicutes_Clostridia_Clostridiales_Ruminococcaceae_u.b.
PAB
LAB
EAB
CG.1
CG.15
CG.437
CG.540
CG.541
PG.2
PG.38
PG.515
PG.528
PG.832
0.00
0.02
0.04
0.06
wk1
wk5
wk10
P23.
Time

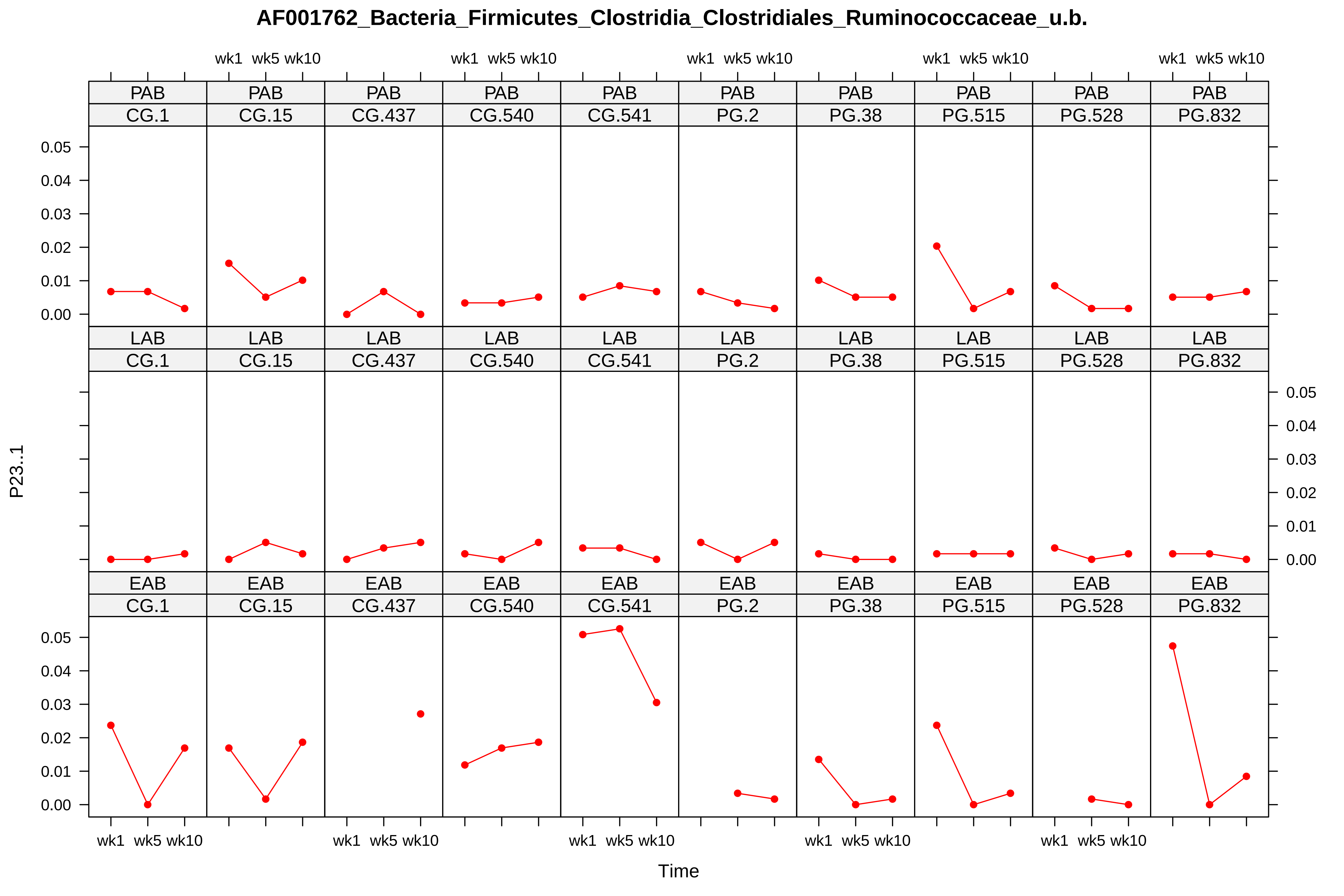
AF001762_Bacteria_Firmicutes_Clostridia_Clostridiales_Ruminococcaceae_u.b.
PAB
CG.1
CG.15
CG.437
CG.540
CG.541
PG.2
PG.38
PG.515
PG.528
PG.832
LAB
EAB
wk1
wk5
wk10
0.00
0.01
0.02
0.03
0.04
0.05
P23..1
Time

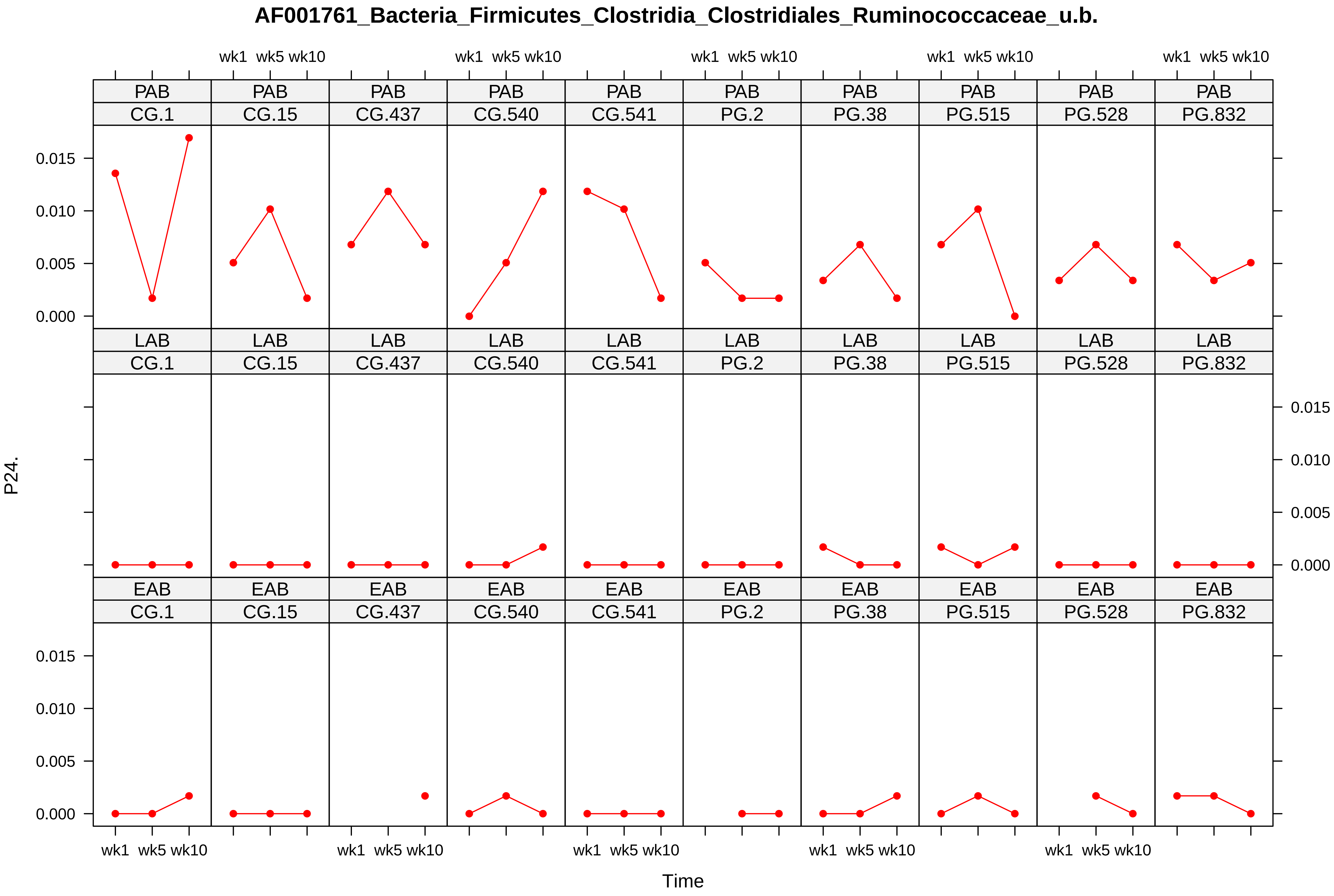
AF001761_Bacteria_Firmicutes_Clostridia_Clostridiales_Ruminococcaceae_u.b.
PAB
LAB
EAB
CG.1
CG.15
CG.437
CG.540
CG.541
PG.2
PG.38
PG.515
PG.528
PG.832
wk1 wk5 wk10
0.000
0.005
0.010
0.015
P24.
Time

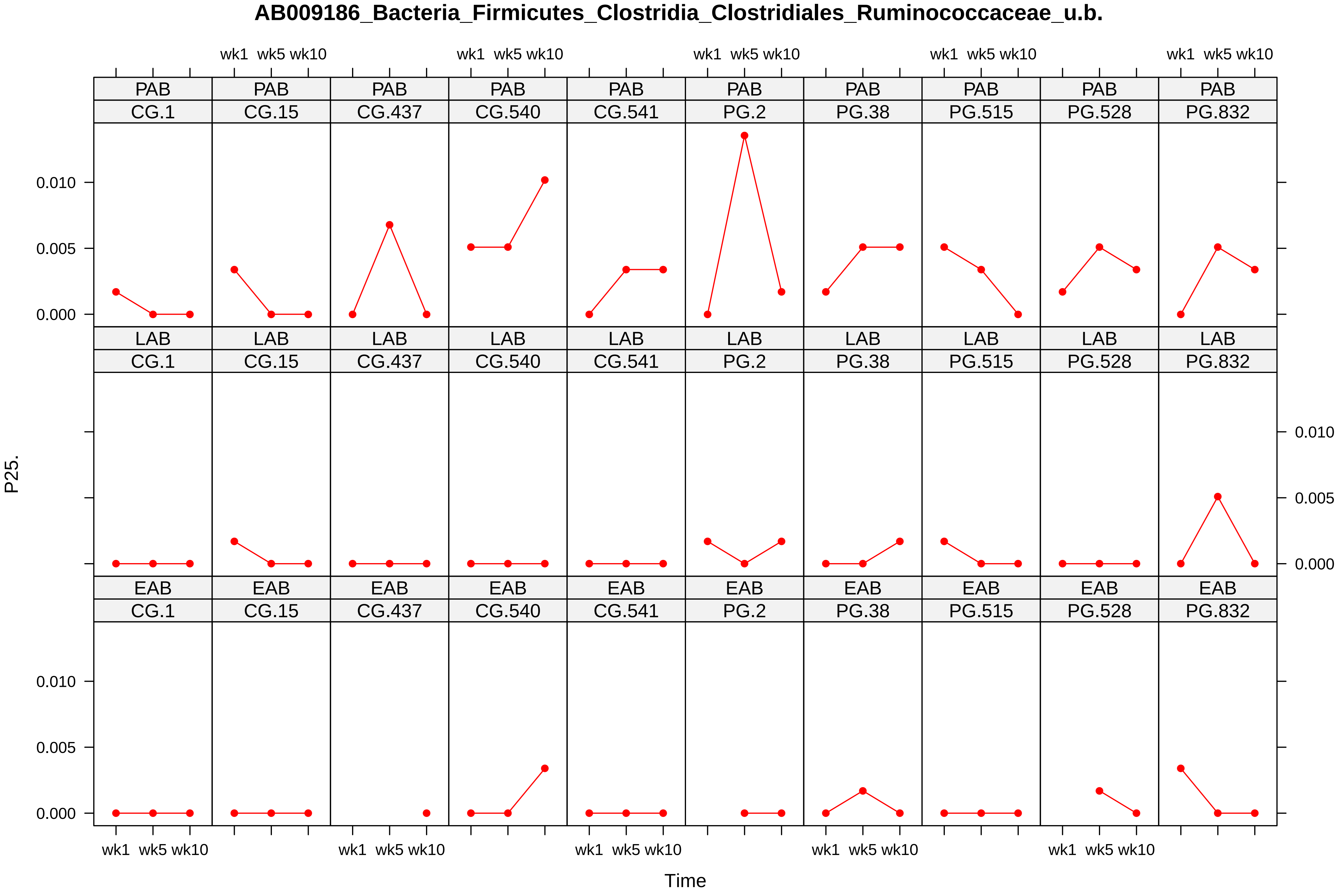
AB009186_Bacteria_Firmicutes_Clostridia_Clostridiales_Ruminococcaceae_u.b.
PAB
LAB
EAB
CG.1
CG.15
CG.437
CG.540
CG.541
PG.2
PG.38
PG.515
PG.528
PG.832
wk1 wk5 wk10
0.000
0.005
0.010
P25.
Time

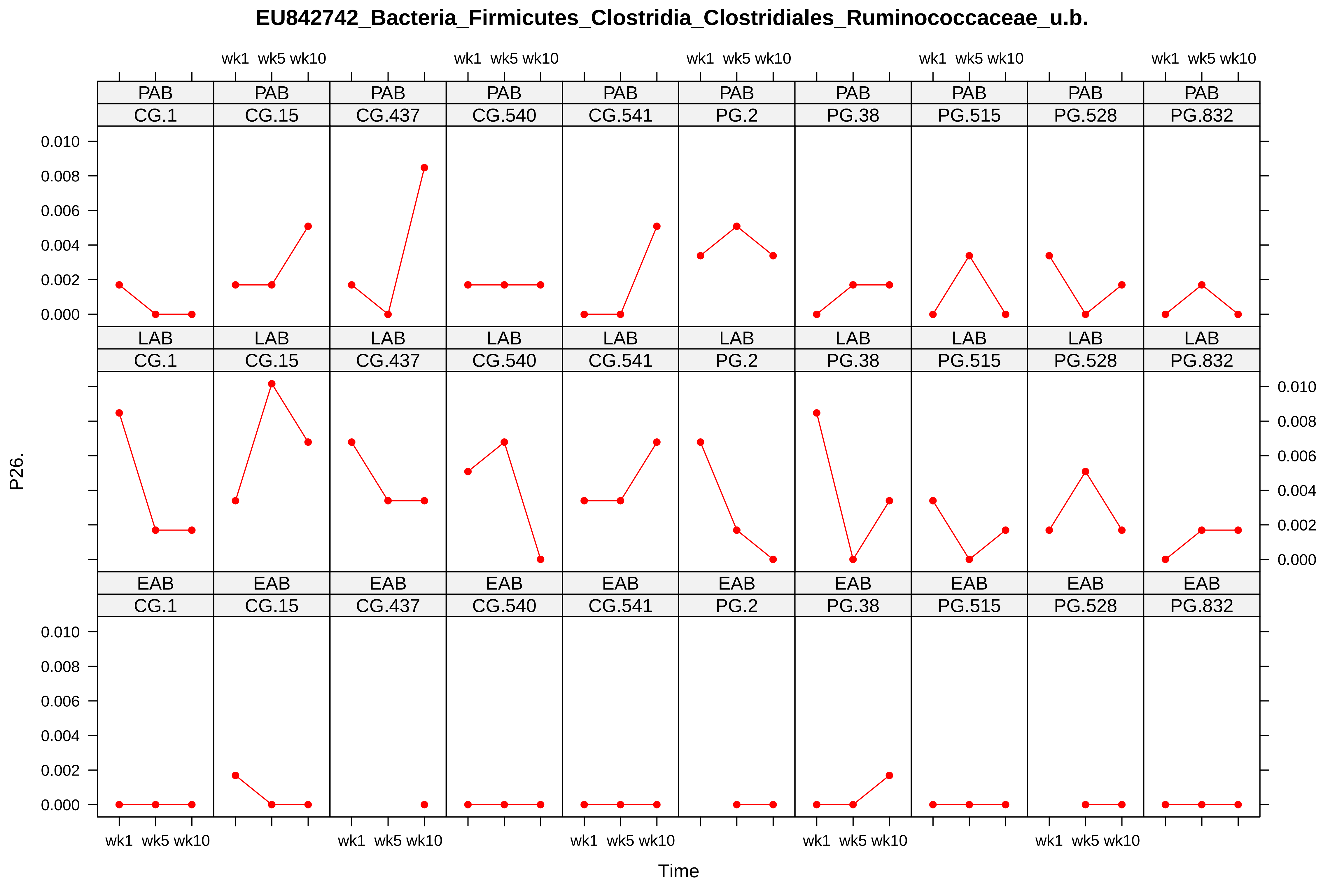
EU842742_Bacteria_Firmicutes_Clostridia_Clostridiales_Ruminococcaceae_u.b.
PAB
LAB
EAB
CG.1
CG.15
CG.437
CG.540
CG.541
PG.2
PG.38
PG.515
PG.528
PG.832
0.000
0.002
0.004
0.006
0.008
0.010
wk1 wk5 wk10
P26.
Time

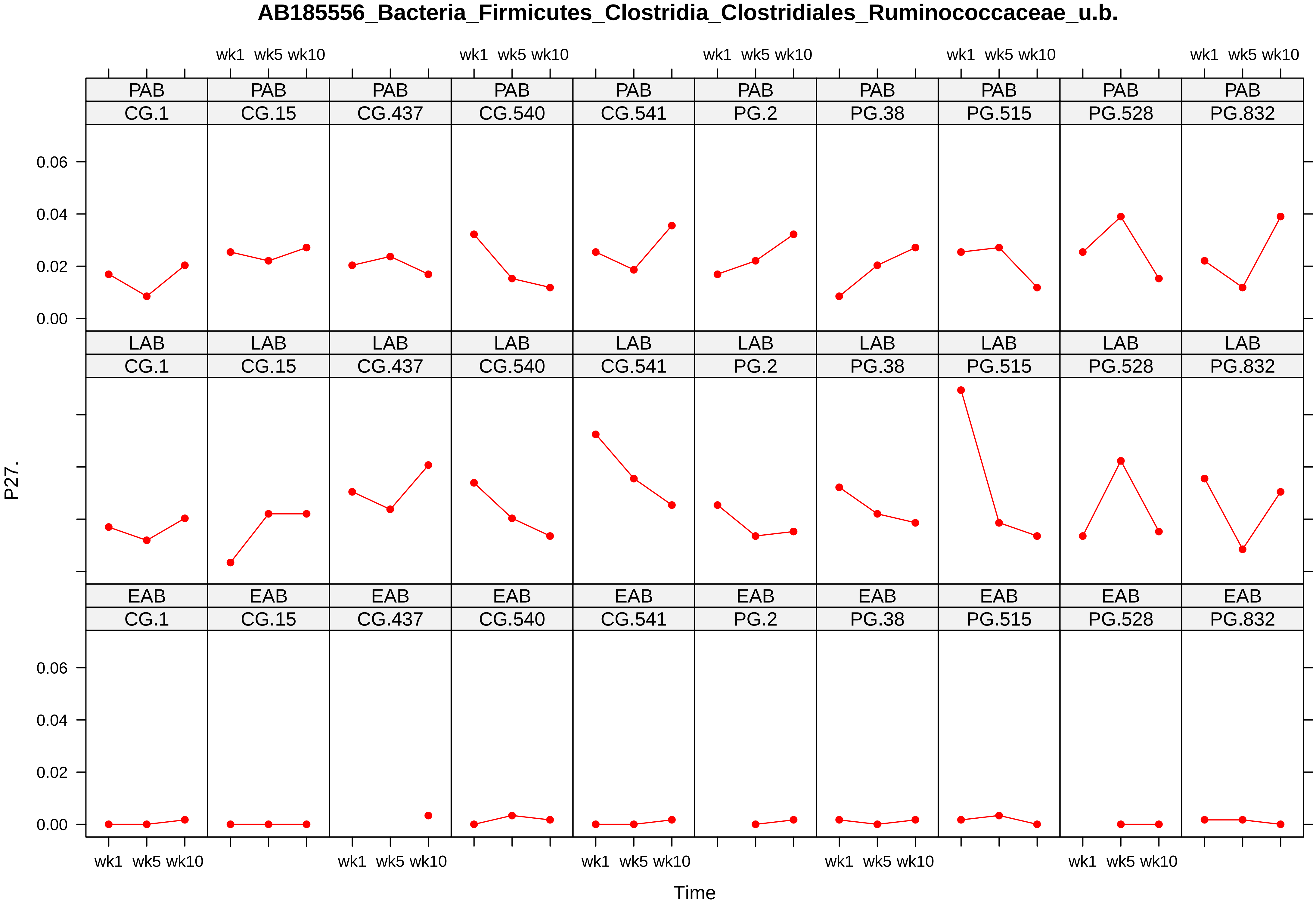
AB185556_Bacteria_Firmicutes_Clostridia_Clostridiales_Ruminococcaceae_u.b.
PAB
LAB
EAB
CG.1
CG.15
CG.437
CG.540
CG.541
PG.2
PG.38
PG.515
PG.528
PG.832
wk1
wk5
wk10
0.00
0.02
0.04
0.06
P27.
Time

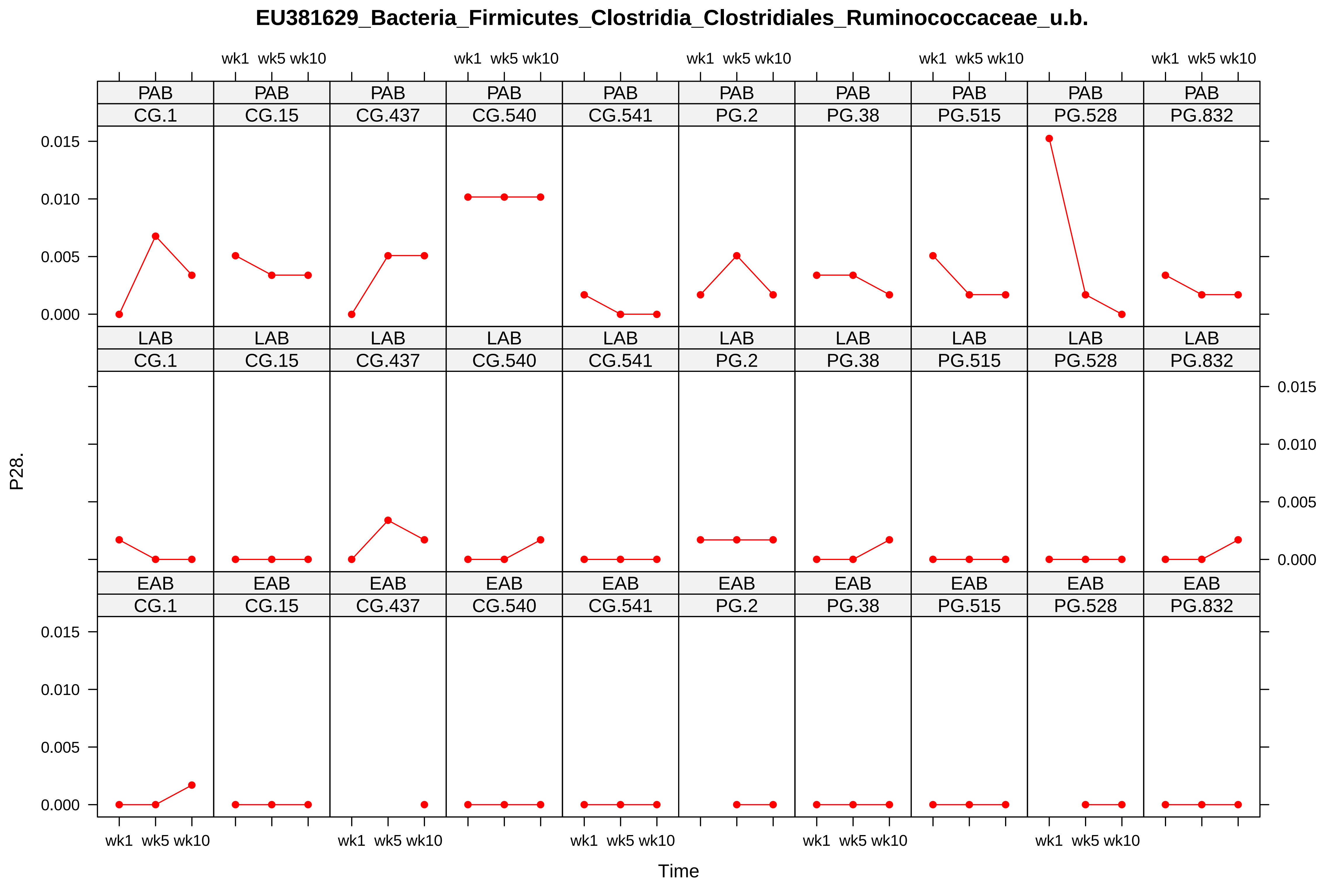

EU381629_Bacteria_Firmicutes_Clostridia_Clostridiales_Ruminococcaceae_u.b.
PAB
LAB
EAB
CG.1
CG.15
CG.437
CG.540
CG.541
PG.2
PG.38
PG.515
PG.528
PG.832
0.000
0.005
0.010
0.015
wk1 wk5 wk10
P28.
Time

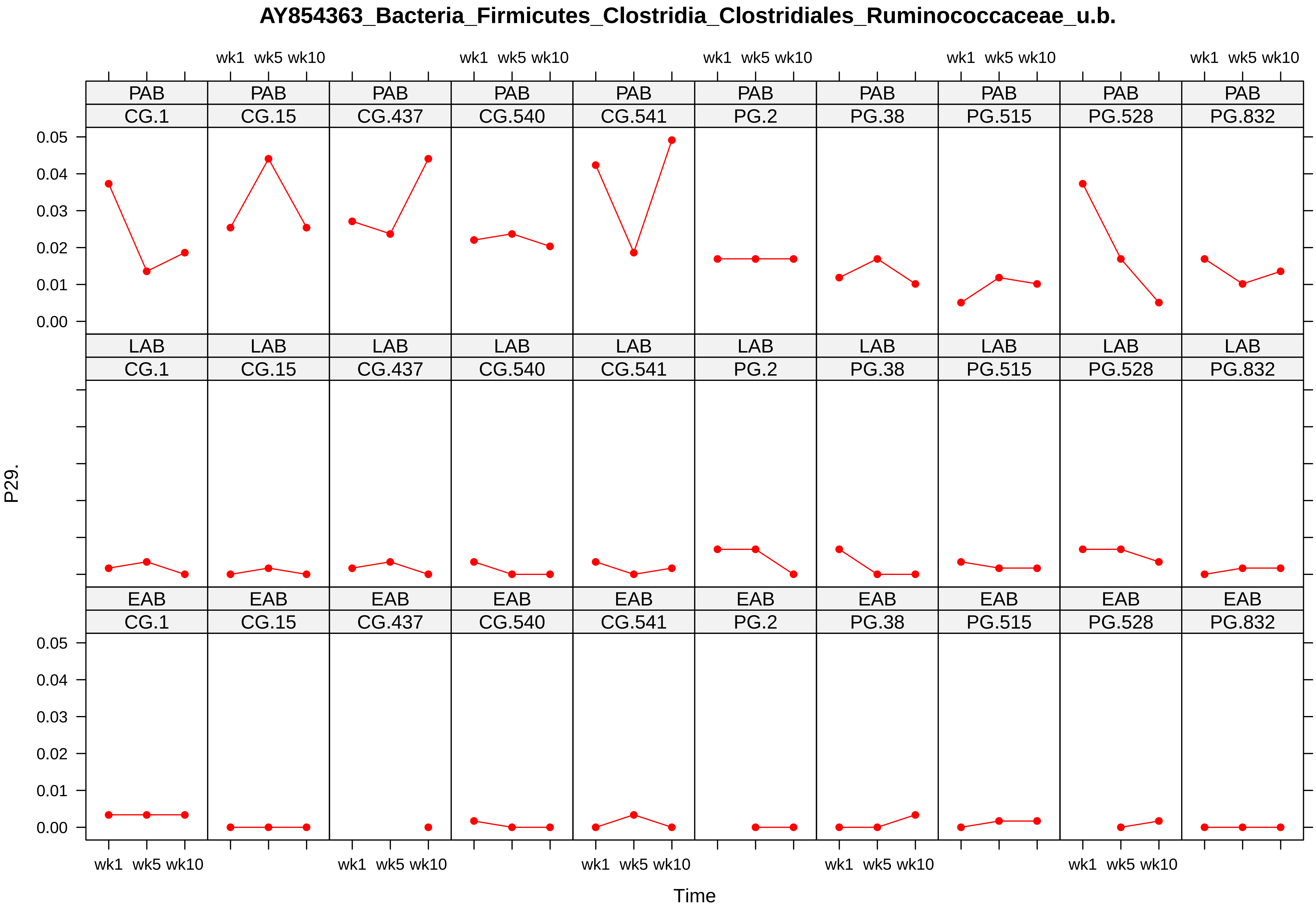

AY854363_Bacteria_Firmicutes_Clostridia_Clostridiales_Ruminococcaceae_u.b.
PAB
LAB
EAB
CG.1
CG.15
CG.437
CG.540
CG.541
PG.2
PG.38
PG.515
PG.528
PG.832
0.00
0.01
0.02
0.03
0.04
0.05
wk1 wk5 wk10
P29.
Time

AB185810_Bacteria_Firmicutes_Clostridia_Clostridiales_Ruminococcaceae_u.b.

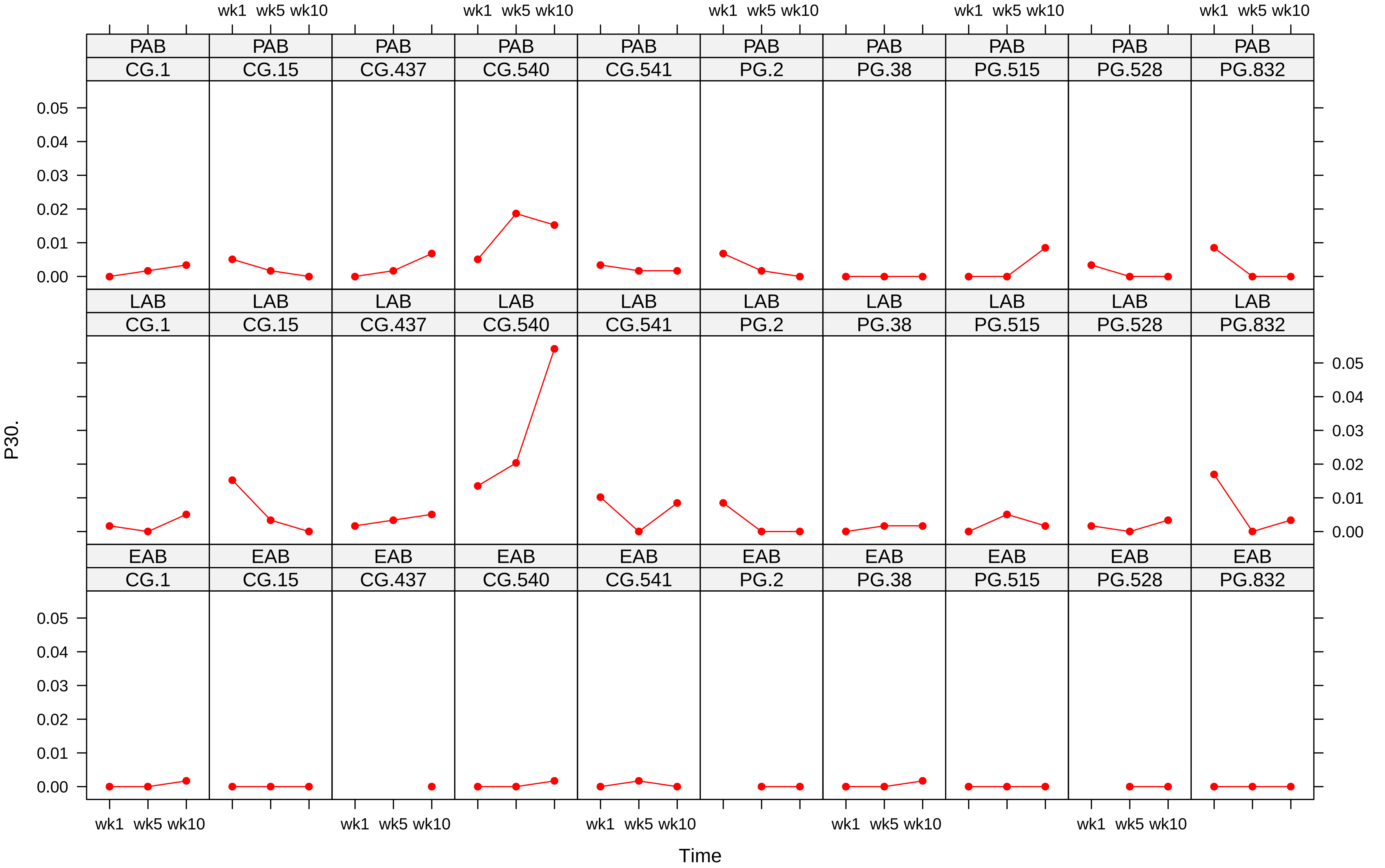

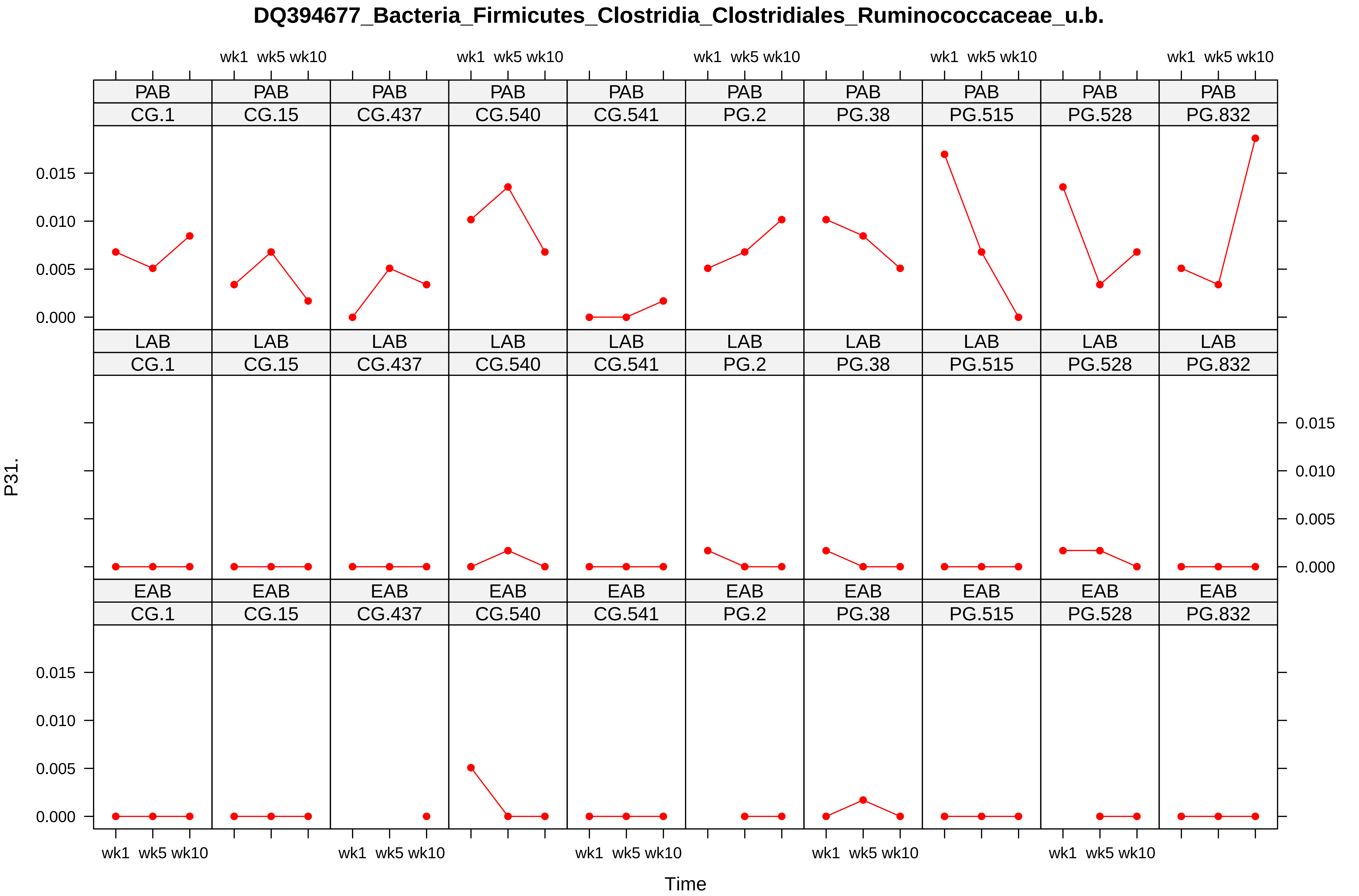
DQ394677_Bacteria_Firmicutes_Clostridia_Clostridiales_Ruminococcaceae_u.b.
PAB
LAB
EAB
CG.1
CG.15
CG.437
CG.540
CG.541
PG.2
PG.38
PG.515
PG.528
PG.832
wk1 wk5 wk10
0.000
0.005
0.010
0.015
P31.
Time

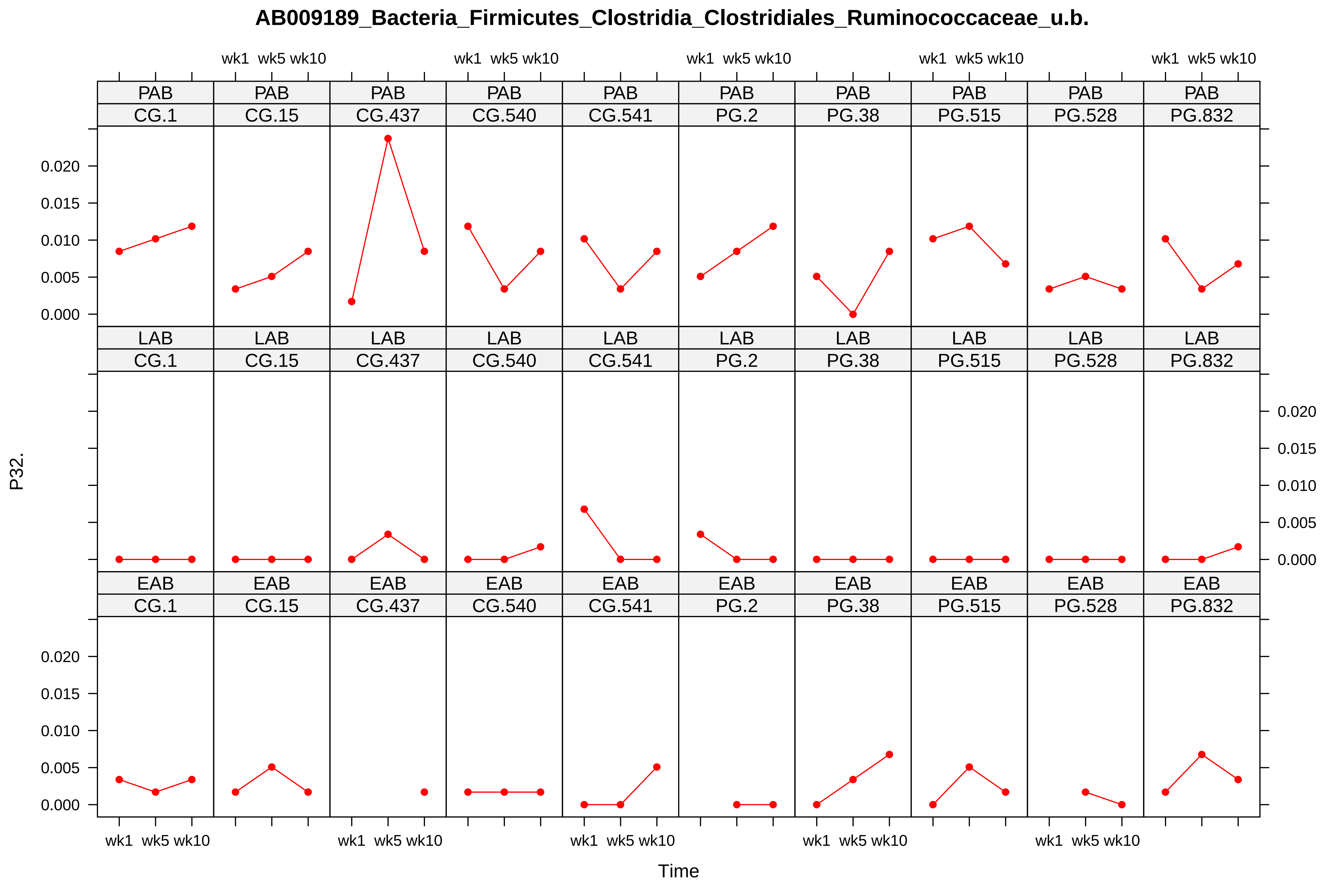
AB009189_Bacteria_Firmicutes_Clostridia_Clostridiales_Ruminococcaceae_u.b.
PAB
LAB
EAB
CG.1
CG.15
CG.437
CG.540
CG.541
PG.2
PG.38
PG.515
PG.528
PG.832
0.000
0.005
0.010
0.015
0.020
wk1 wk5 wk10
P32.
Time

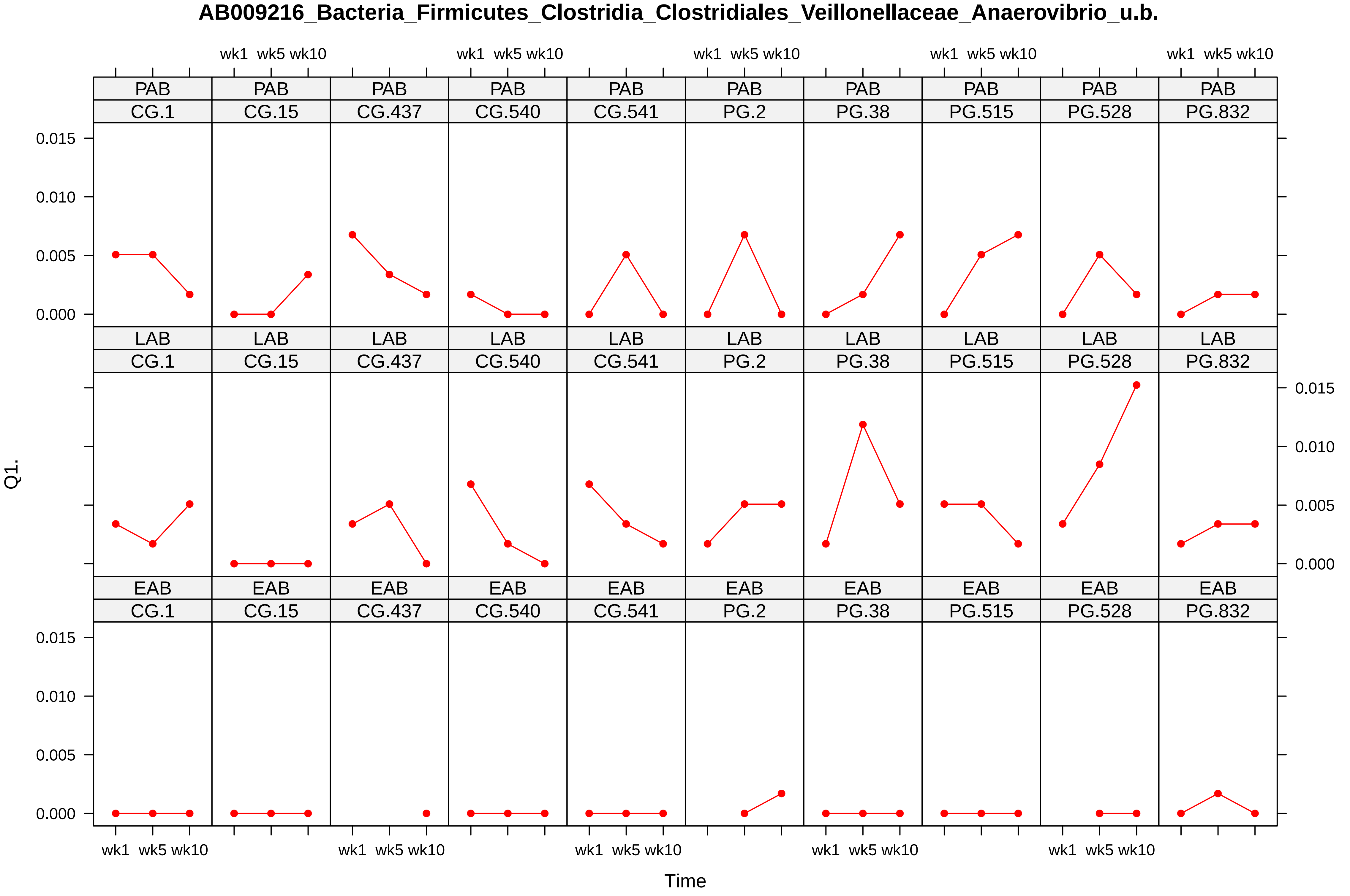
AB009216_Bacteria_Firmicutes_Clostridia_Clostridiales_Veillonellaceae_Anaerovibrio_u.b.
PAB
LAB
EAB
CG.1
CG.15
CG.437
CG.540
CG.541
PG.2
PG.38
PG.515
PG.528
PG.832
0.000
0.005
0.010
0.015
wk1 wk5 wk10
Q1.
Time

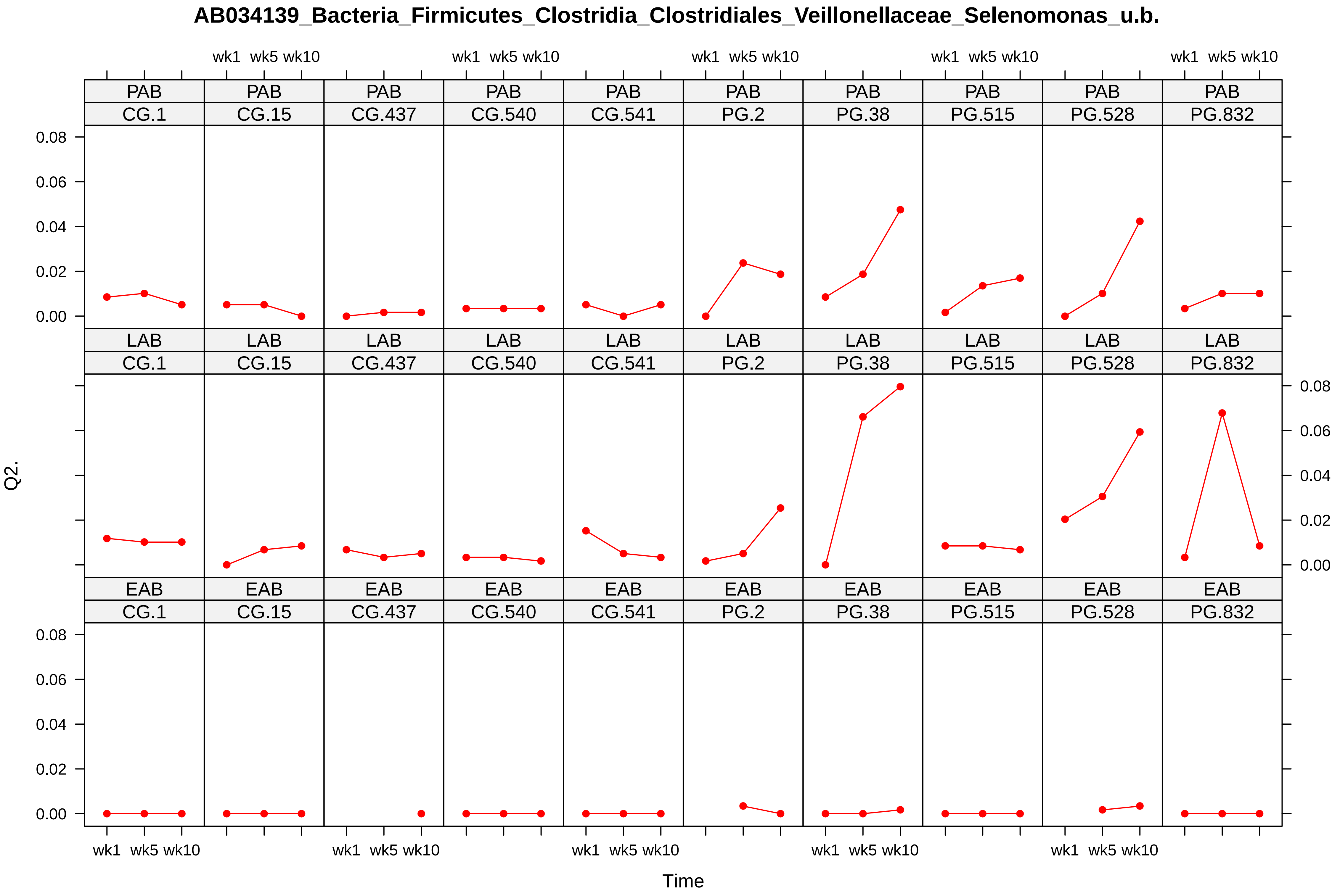
AB034139_Bacteria_Firmicutes_Clostridia_Clostridiales_Veillonellaceae_Selenomonas_u.b.
PAB
LAB
EAB
CG.1
CG.15
CG.437
CG.540
CG.541
PG.2
PG.38
PG.515
PG.528
PG.832
wk1 wk5 wk10
0.00
0.02
0.04
0.06
0.08
Q2.
Time

GQ327079_Bacteria_Firmicutes_Clostridia_Clostridiales_Veillonellaceae_Selenomonas_u.b.

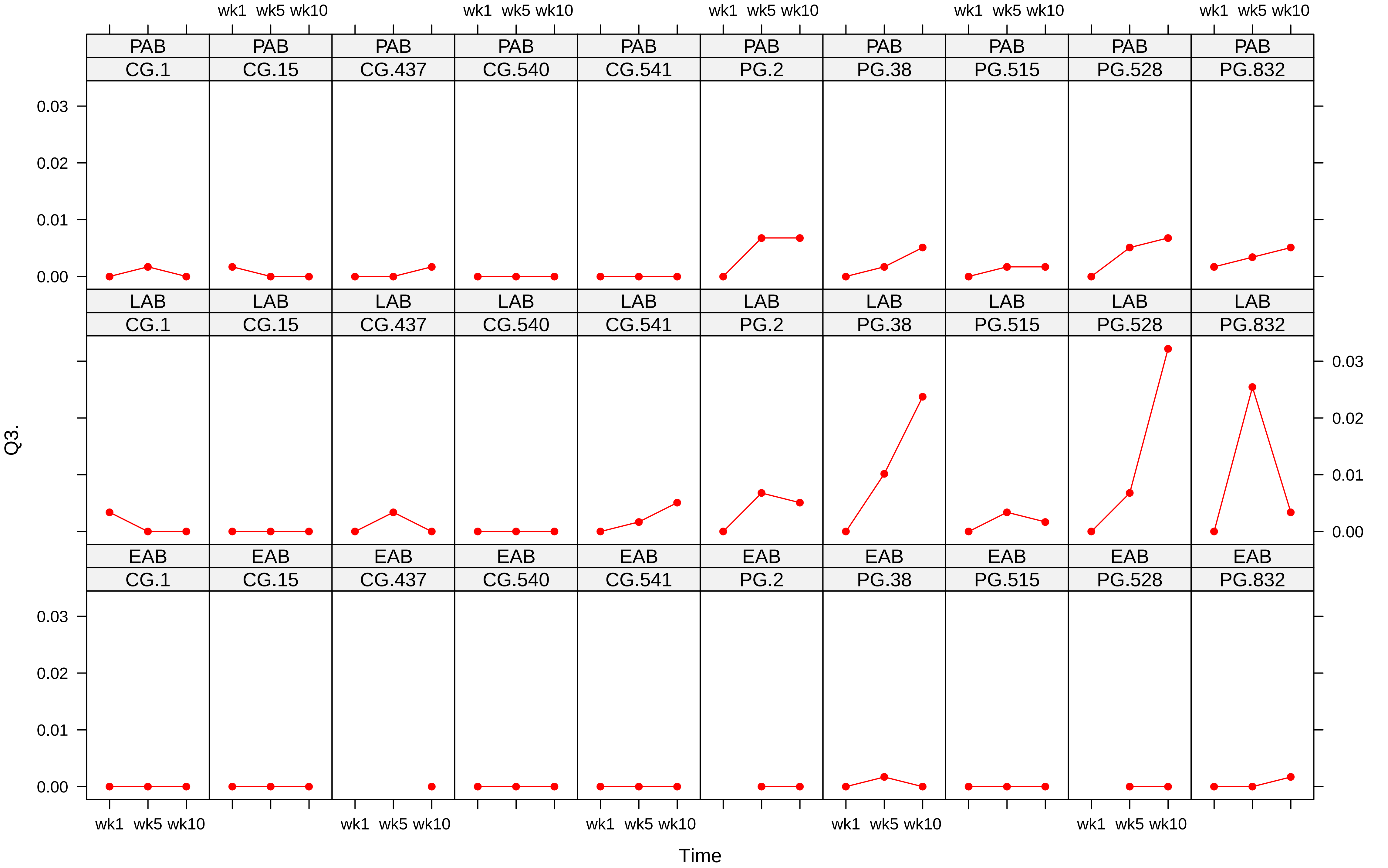

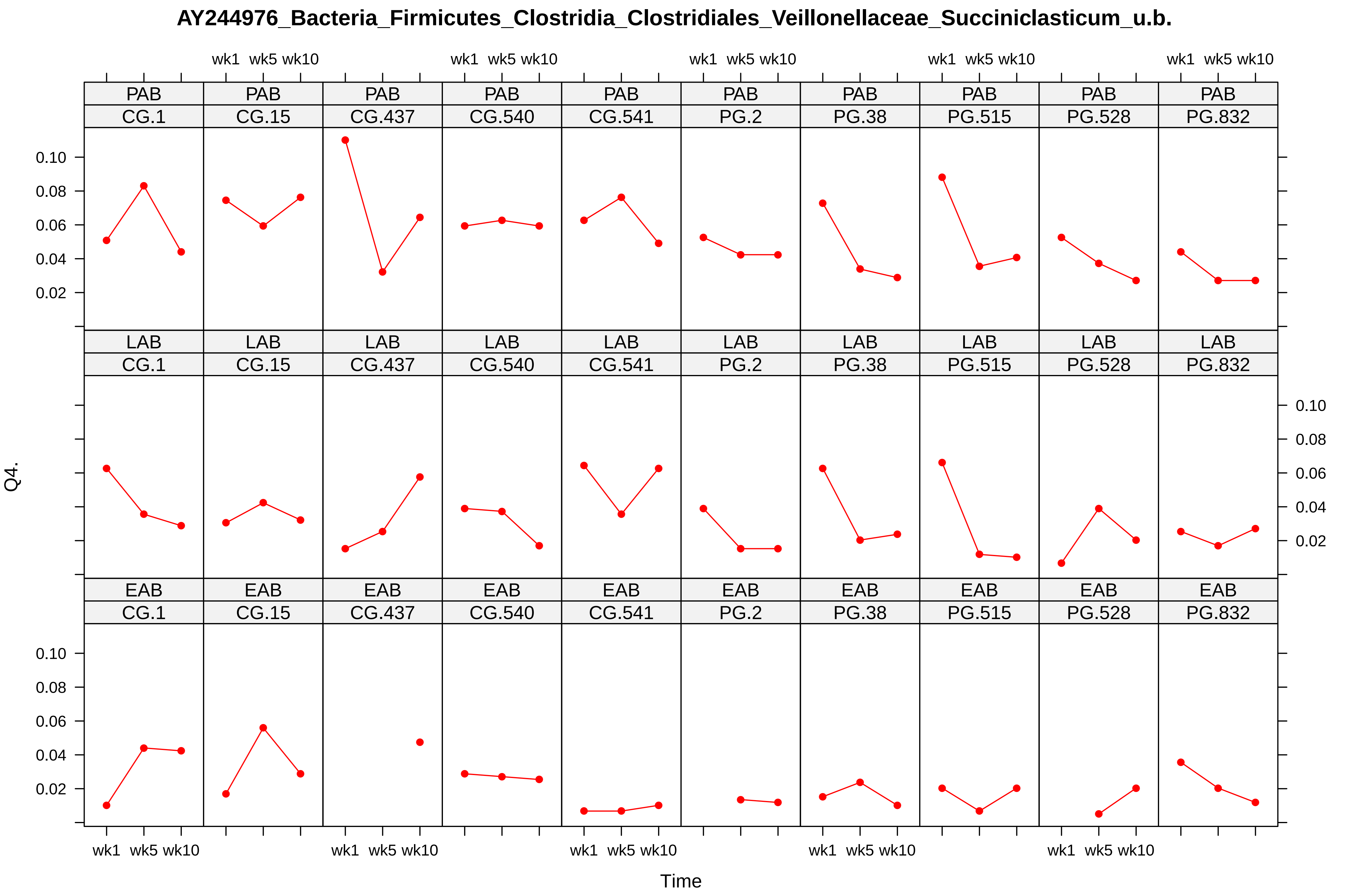

AY244976_Bacteria_Firmicutes_Clostridia_Clostridiales_Veillonellaceae_Succiniclasticum_u.b.
PAB
LAB
EAB
CG.1
CG.15
CG.437
CG.540
CG.541
PG.2
PG.38
PG.515
PG.528
PG.832
wk1
wk5
wk10
0.02
0.04
0.06
0.08
0.10
Q4.
Time

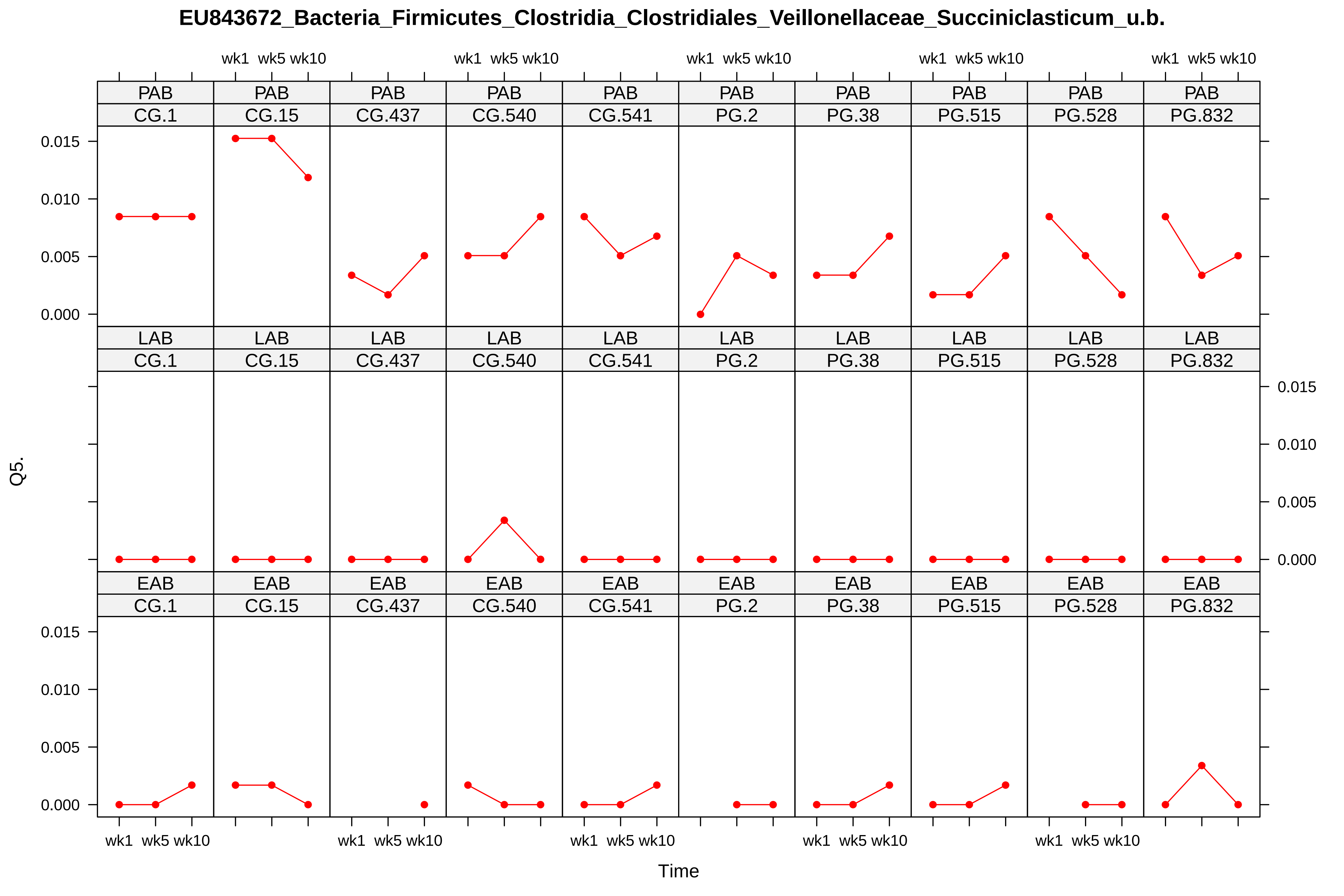

EU843672_Bacteria_Firmicutes_Clostridia_Clostridiales_Veillonellaceae_Succiniclasticum_u.b.
PAB
LAB
EAB
CG.1
CG.15
CG.437
CG.540
CG.541
PG.2
PG.38
PG.515
PG.528
PG.832
0.000
0.005
0.010
0.015
wk1 wk5 wk10
Q5.
Time

AB210825_Bacteria_Firmicutes_Erysipelotrichi_Erysipelotrichales_Erysipelotrichaceae_Catenibacterium_u.b.

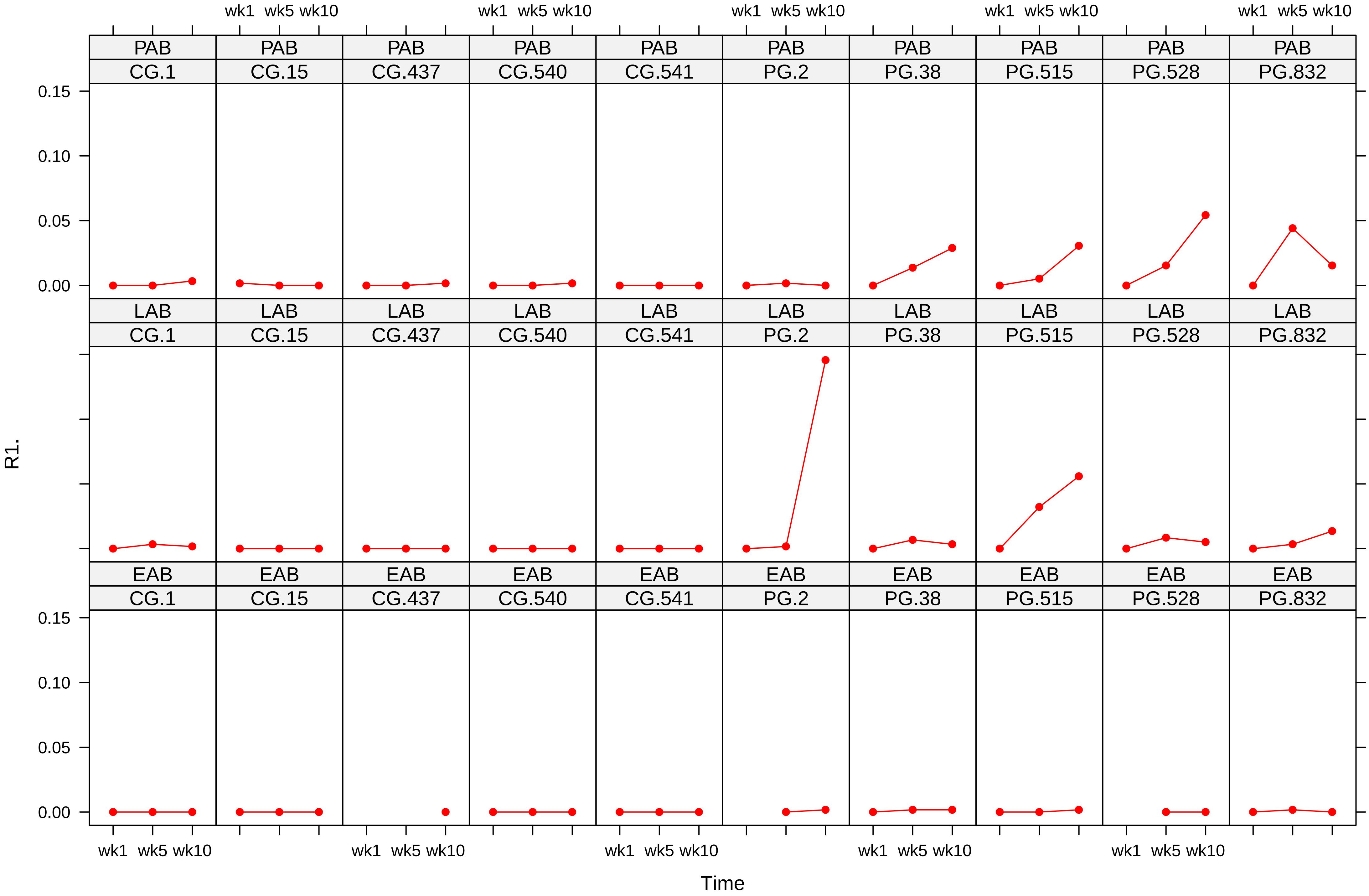

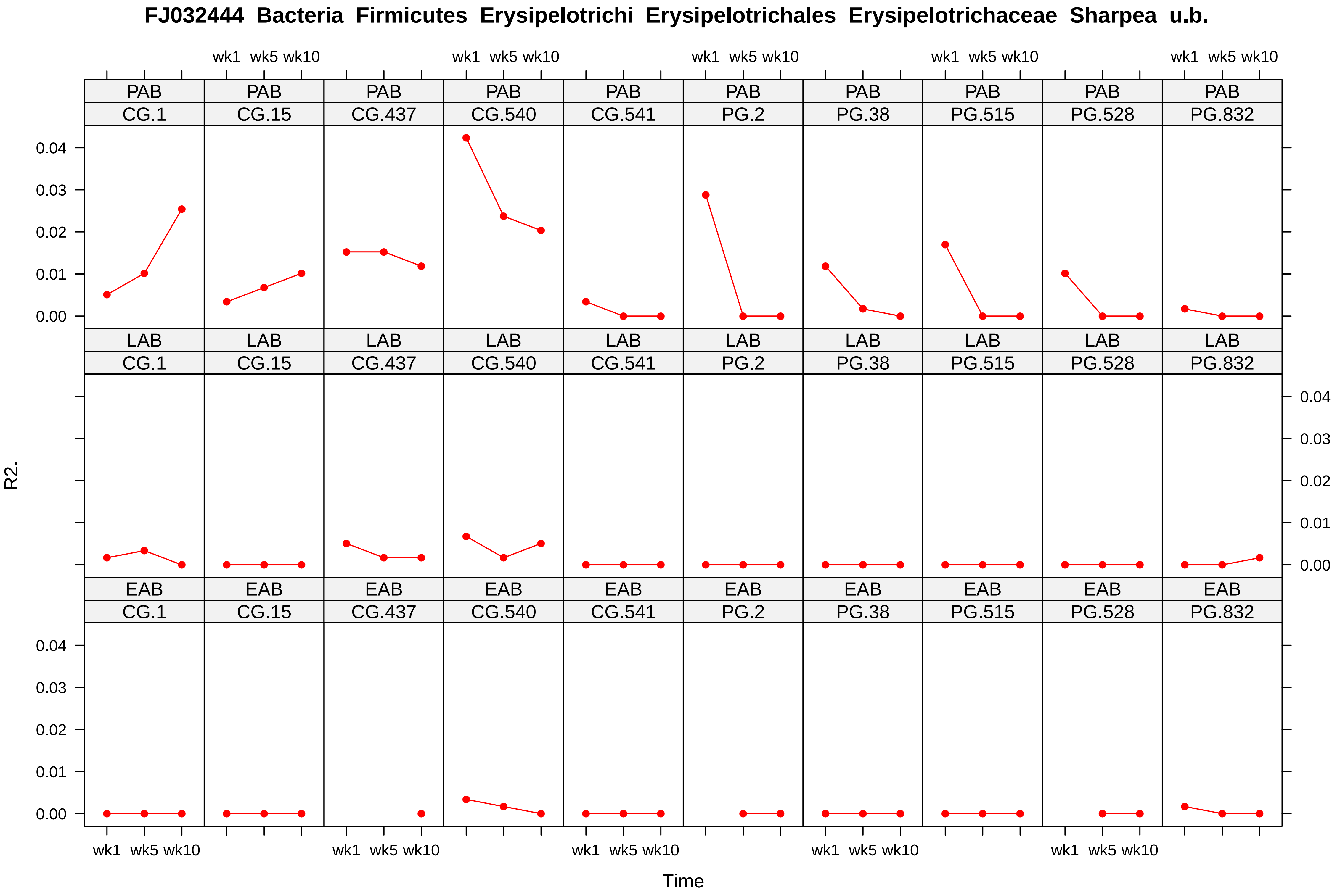

FJ032444_Bacteria_Firmicutes_Erysipelotrichi_Erysipelotrichales_Erysipelotrichaceae_Sharpea_u.b.
PAB
LAB
EAB
CG.1
CG.15
CG.437
CG.540
CG.541
PG.2
PG.38
PG.515
PG.528
PG.832
wk1 wk5 wk10
0.00
0.01
0.02
0.03
0.04
R2.
Time

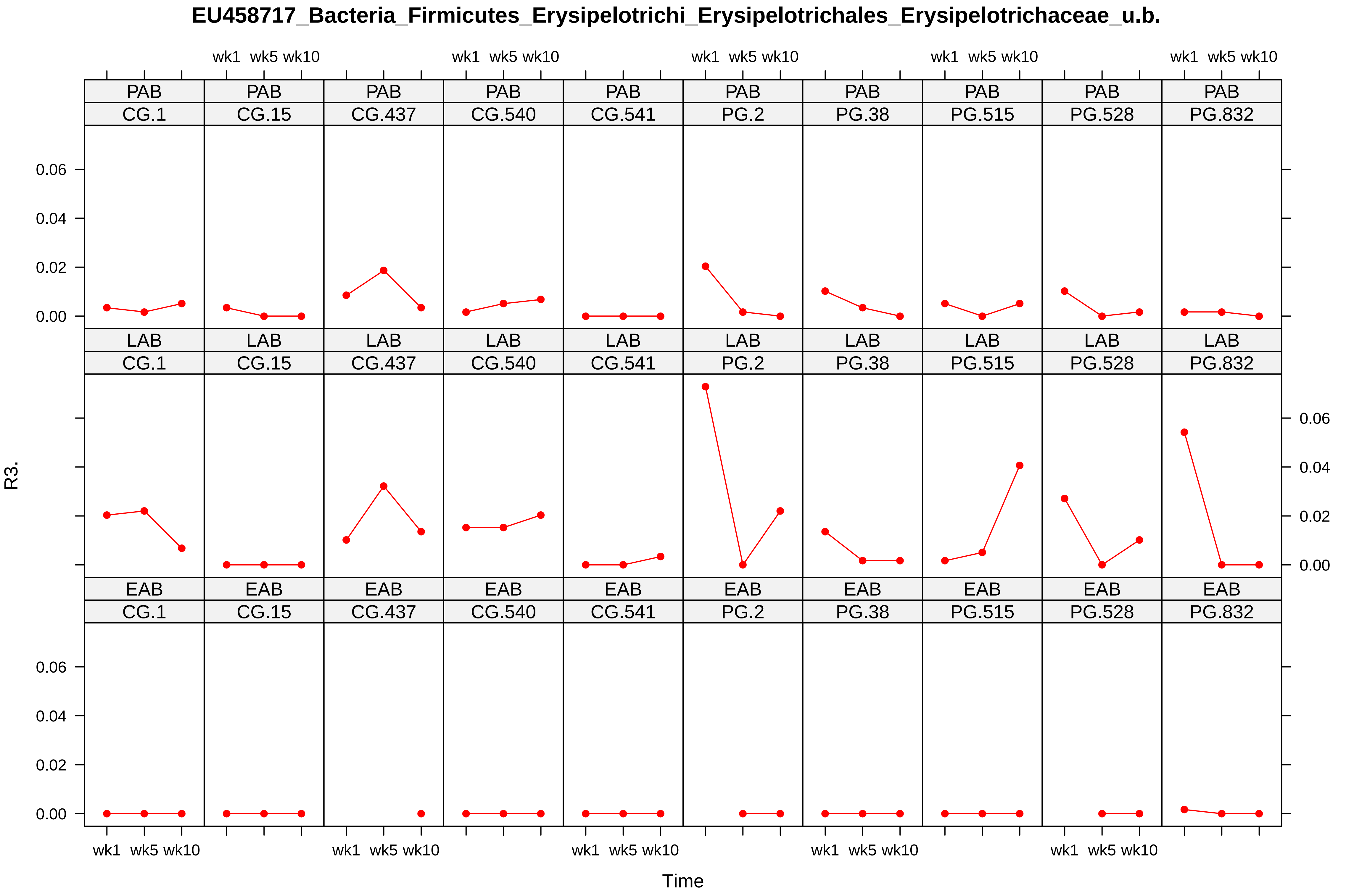

EU458717_Bacteria_Firmicutes_Erysipelotrichi_Erysipelotrichales_Erysipelotrichaceae_u.b.
PAB
LAB
EAB
CG.1
CG.15
CG.437
CG.540
CG.541
PG.2
PG.38
PG.515
PG.528
PG.832
wk1 wk5 wk10
0.00
0.02
0.04
0.06
R3.
Time

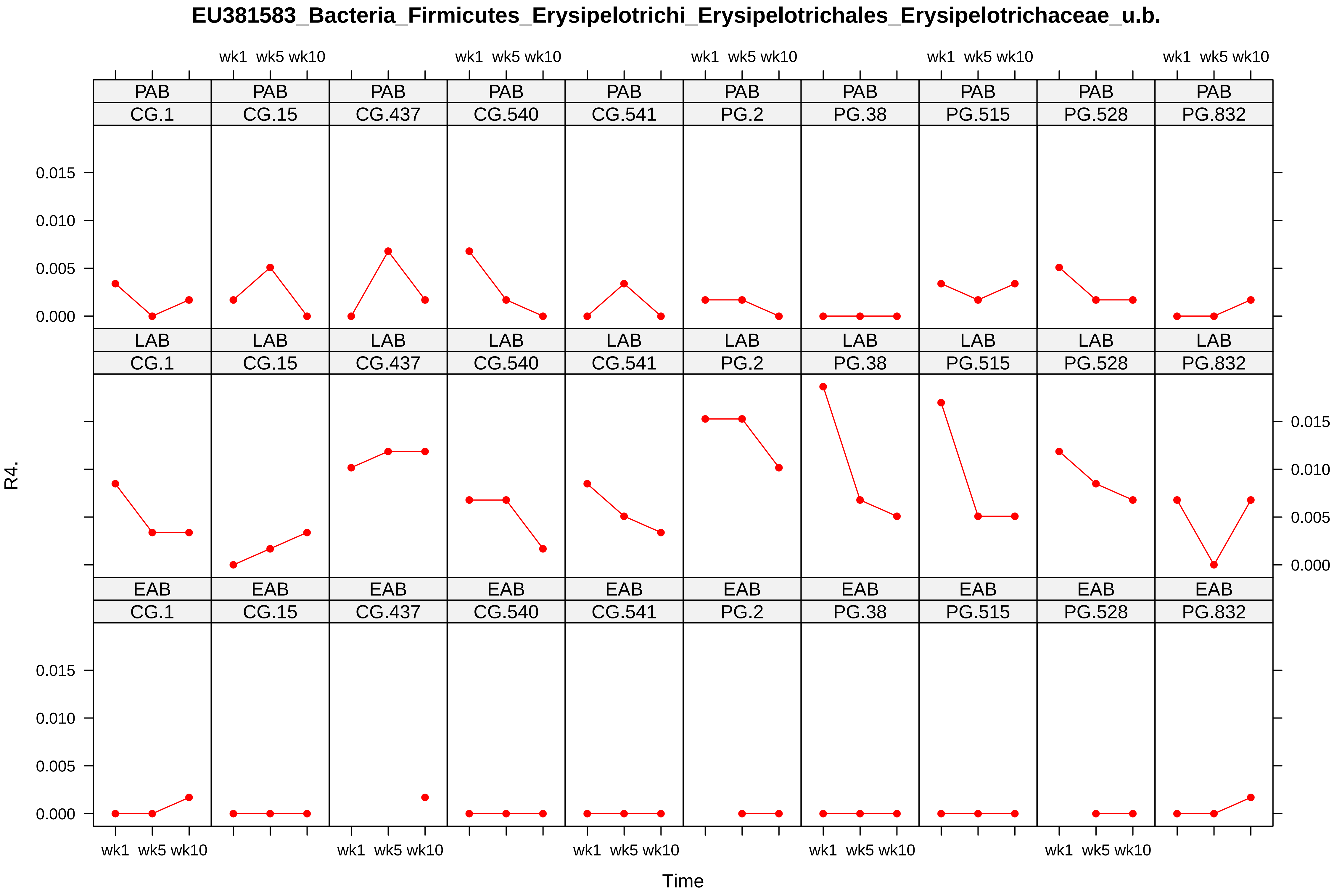
EU381583_Bacteria_Firmicutes_Erysipelotrichi_Erysipelotrichales_Erysipelotrichaceae_u.b.
PAB
LAB
EAB
CG.1
CG.15
CG.437
CG.540
CG.541
PG.2
PG.38
PG.515
PG.528
PG.832
wk1 wk5 wk10
0.000
0.005
0.010
0.015
R4.
Time

EU381506_Bacteria_Firmicutes_Erysipelotrichi_Erysipelotrichales_Erysipelotrichaceae_u.b.

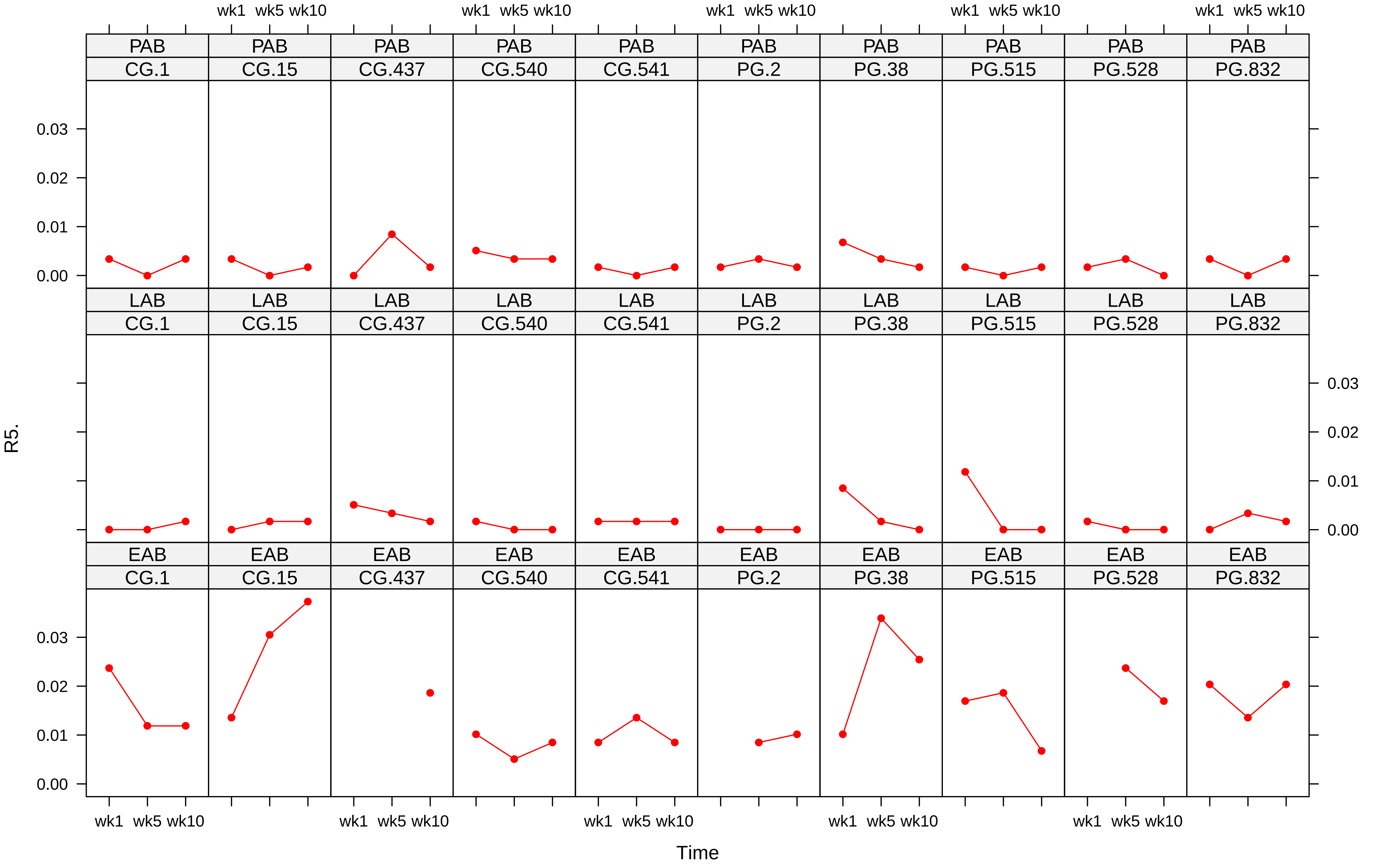

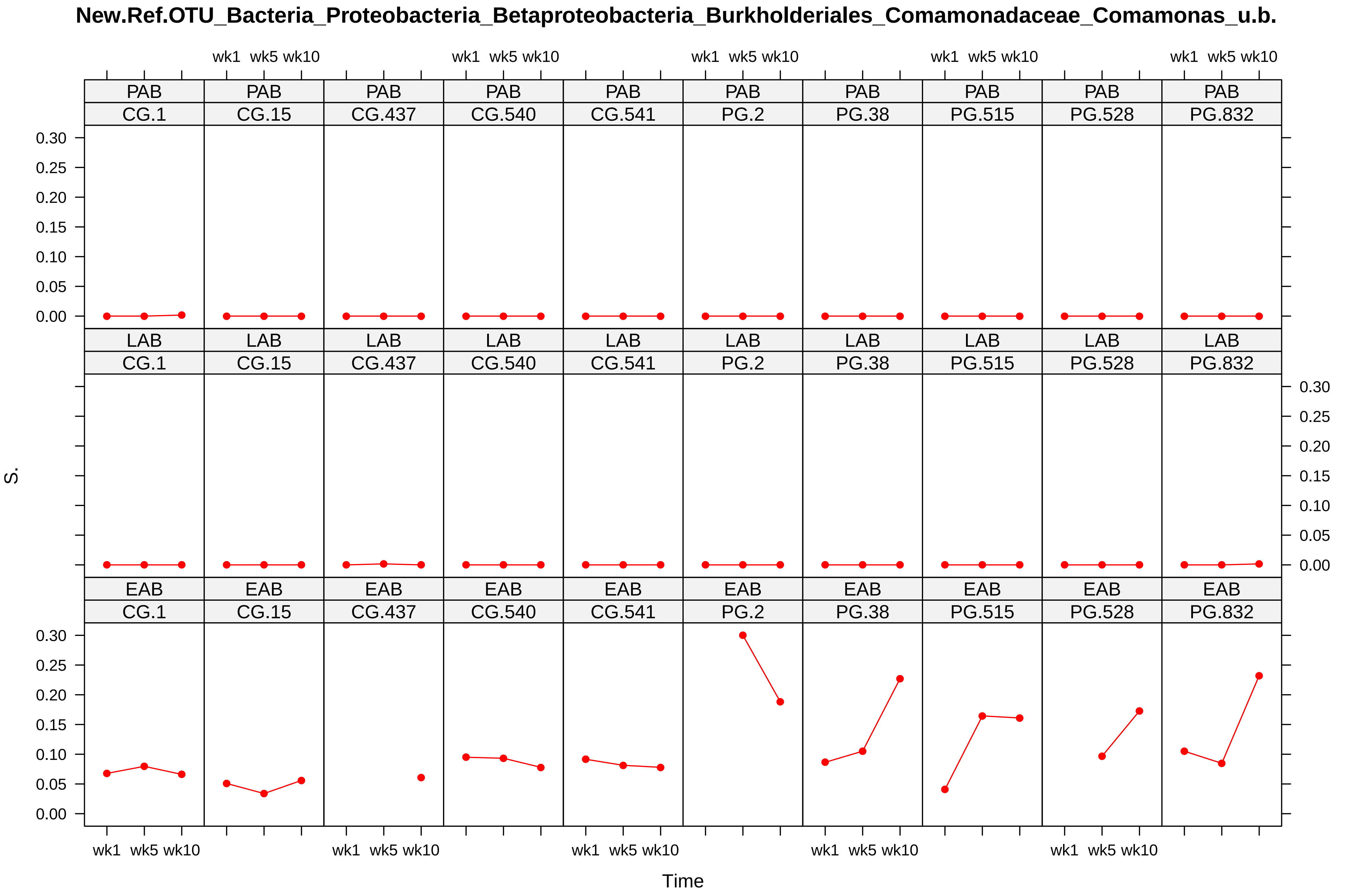

New.Ref.OTU_Bacteria_Proteobacteria_Betaproteobacteria_Burkholderiales_Comamonadaceae_Comamonas_u.b.
PAB CG.1
PAB CG.15
PAB CG.437
PAB CG.540
PAB CG.541
PAB PG.2
PAB PG.38
PAB PG.515
PAB PG.528
PAB PG.832
LAB CG.1
LAB CG.15
LAB CG.437
LAB CG.540
LAB CG.541
LAB PG.2
LAB PG.38
LAB PG.515
LAB PG.528
LAB PG.832
EAB CG.1
EAB CG.15
EAB CG.437
EAB CG.540
EAB CG.541
EAB PG.2
EAB PG.38
EAB PG.515
EAB PG.528
EAB PG.832
0.30
0.25
0.20
0.15
0.10
0.05
0.00
wk1 wk5 wk10
S.
Time

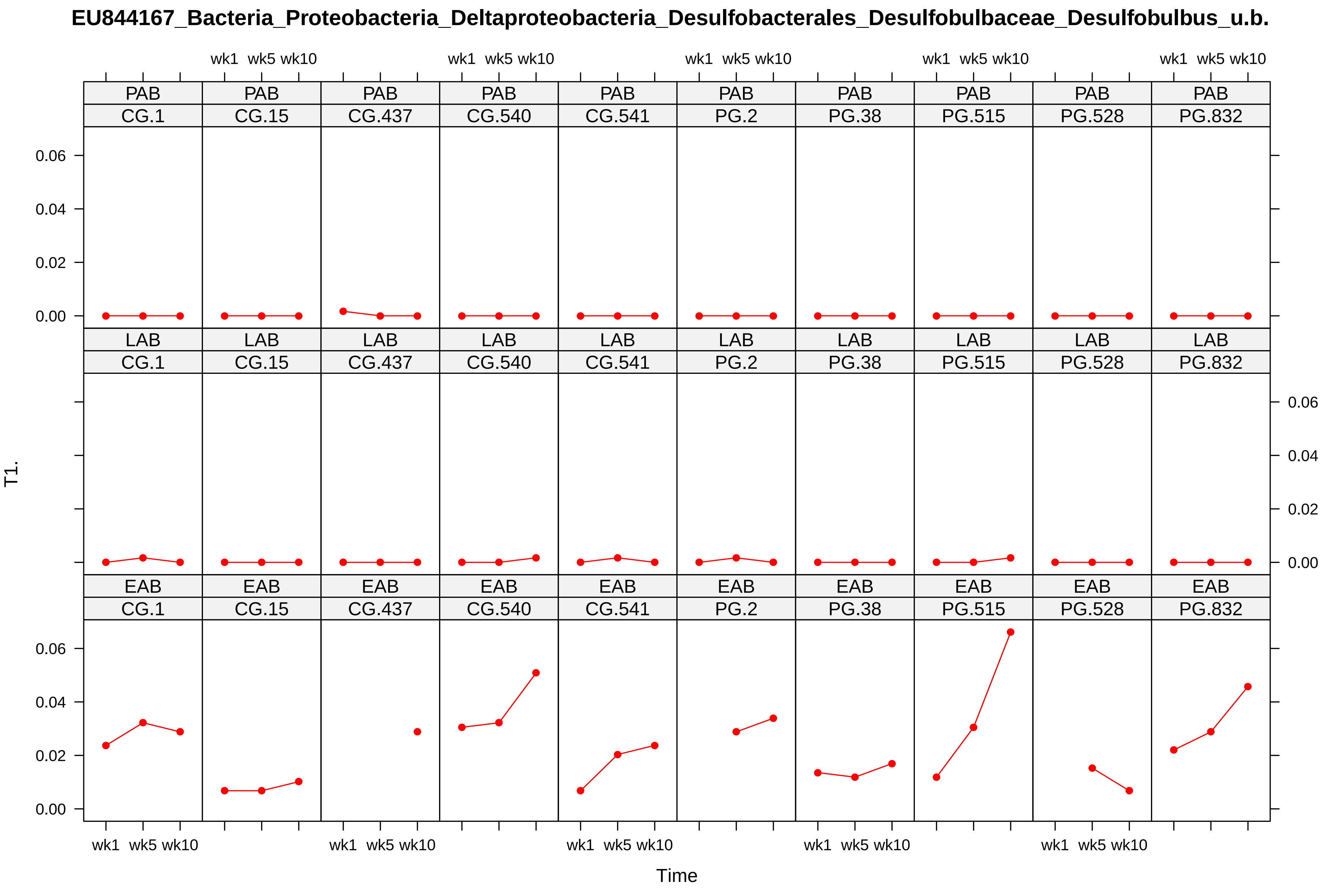
EU844167_Bacteria_Proteobacteria_Deltaproteobacteria_Desulfobacterales_Desulfobulbaceae_Desulfobulbus_u.b.
PAB
LAB
EAB
CG.1
CG.15
CG.437
CG.540
CG.541
PG.2
PG.38
PG.515
PG.528
PG.832
wk1 wk5 wk10
0.00
0.02
0.04
0.06
T1.
Time

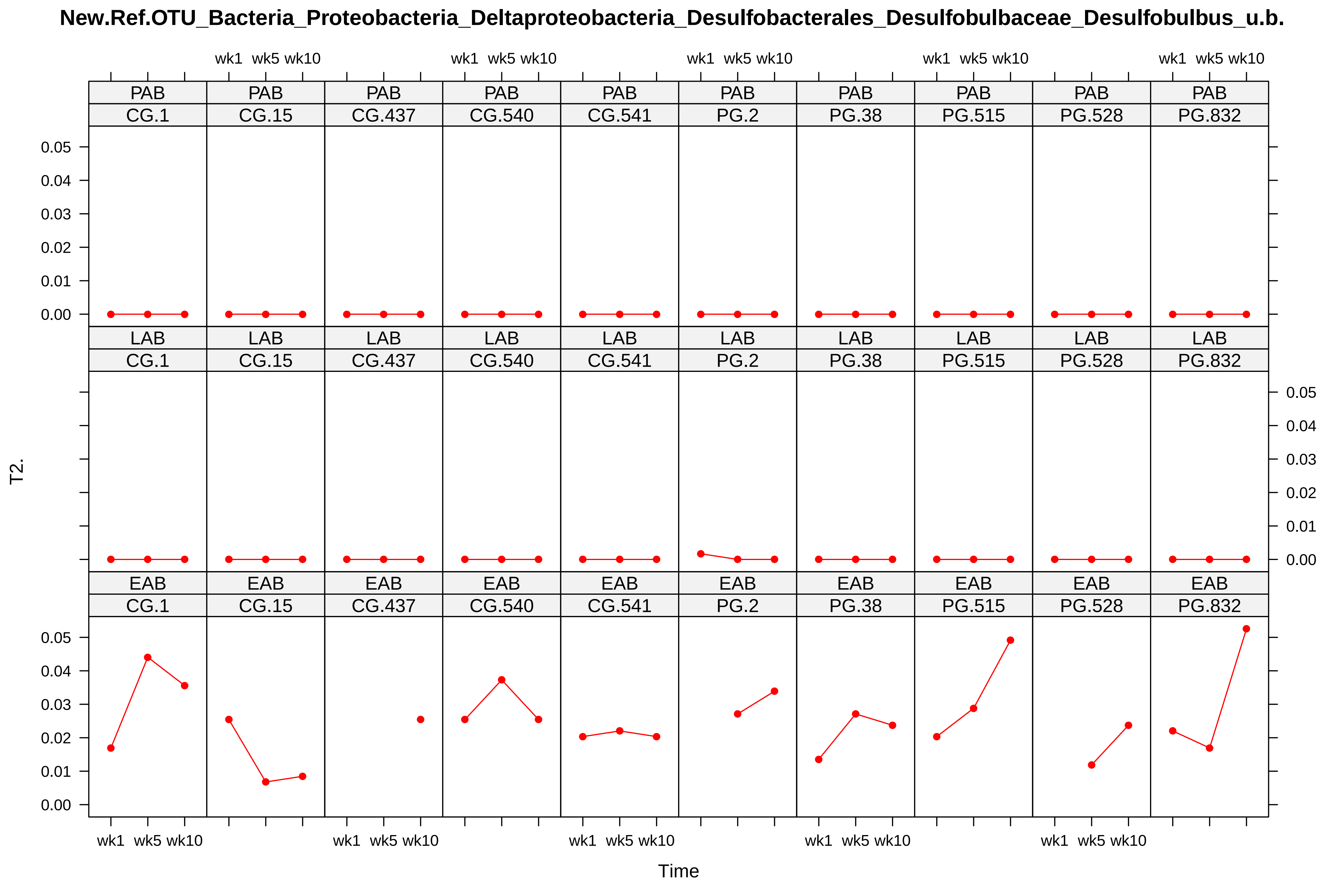
New.Ref.OTU_Bacteria_Proteobacteria_Deltaproteobacteria_Desulfobacterales_Desulfobulbaceae_Desulfobulbus_u.b.
PAB
LAB
EAB
CG.1
CG.15
CG.437
CG.540
CG.541
PG.2
PG.38
PG.515
PG.528
PG.832
wk1 wk5 wk10
0.00
0.01
0.02
0.03
0.04
0.05
T2.
Time

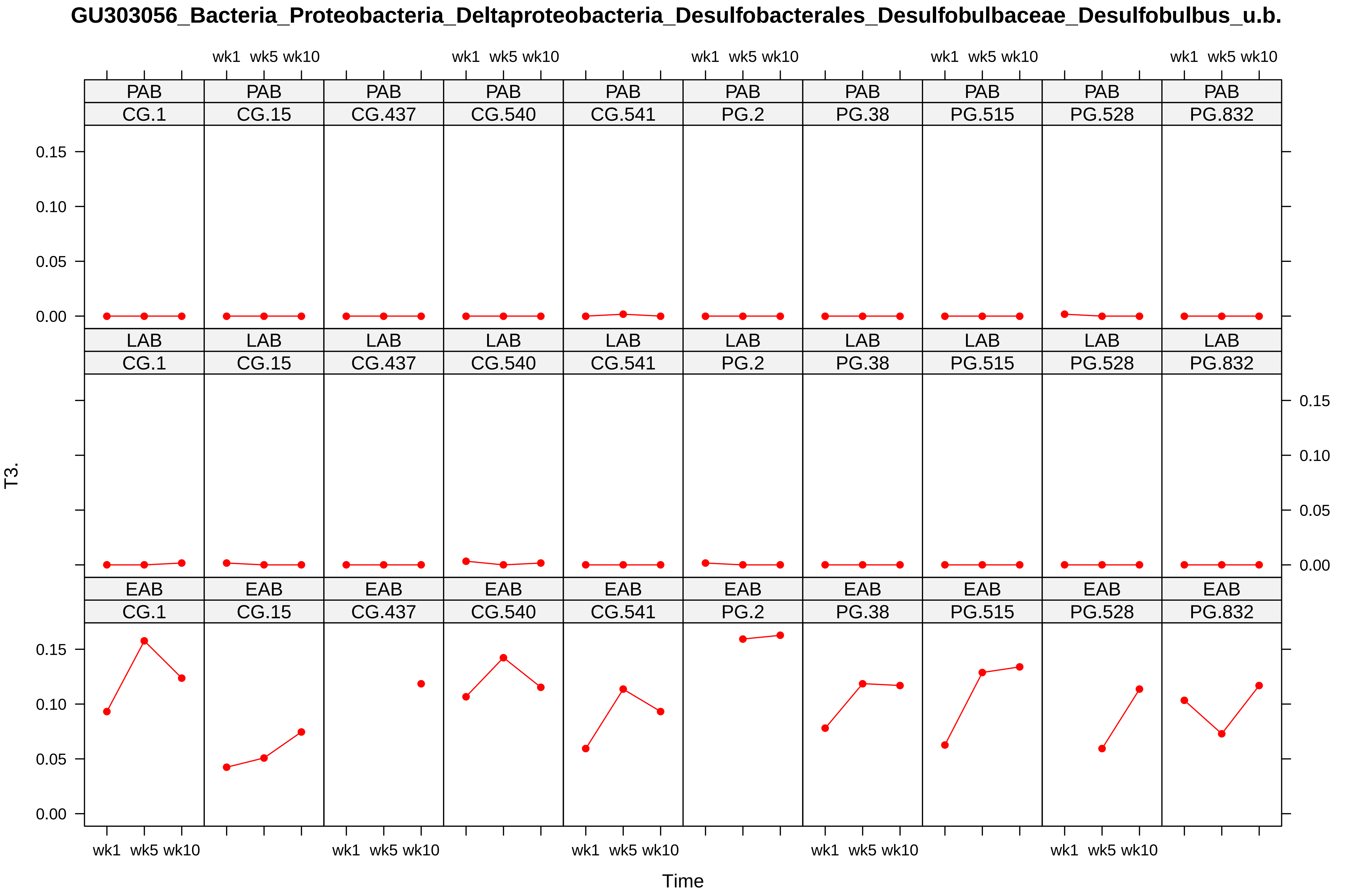

GU303056_Bacteria_Proteobacteria_Deltaproteobacteria_Desulfobacterales_Desulfobulbaceae_Desulfobulbus_u.b.
PAB CG.1
PAB CG.15
PAB CG.437
PAB CG.540
PAB CG.541
PAB PG.2
PAB PG.38
PAB PG.515
PAB PG.528
PAB PG.832
LAB CG.1
LAB CG.15
LAB CG.437
LAB CG.540
LAB CG.541
LAB PG.2
LAB PG.38
LAB PG.515
LAB PG.528
LAB PG.832
EAB CG.1
EAB CG.15
EAB CG.437
EAB CG.540
EAB CG.541
EAB PG.2
EAB PG.38
EAB PG.515
EAB PG.528
EAB PG.832
0.00
0.05
0.10
0.15
wk1 wk5 wk10
T3.
Time

DQ174169_Bacteria_Proteobacteria_Epsilonproteobacteria_Campylobacterales_Campylobacteraceae_Campylobacter_u.b.

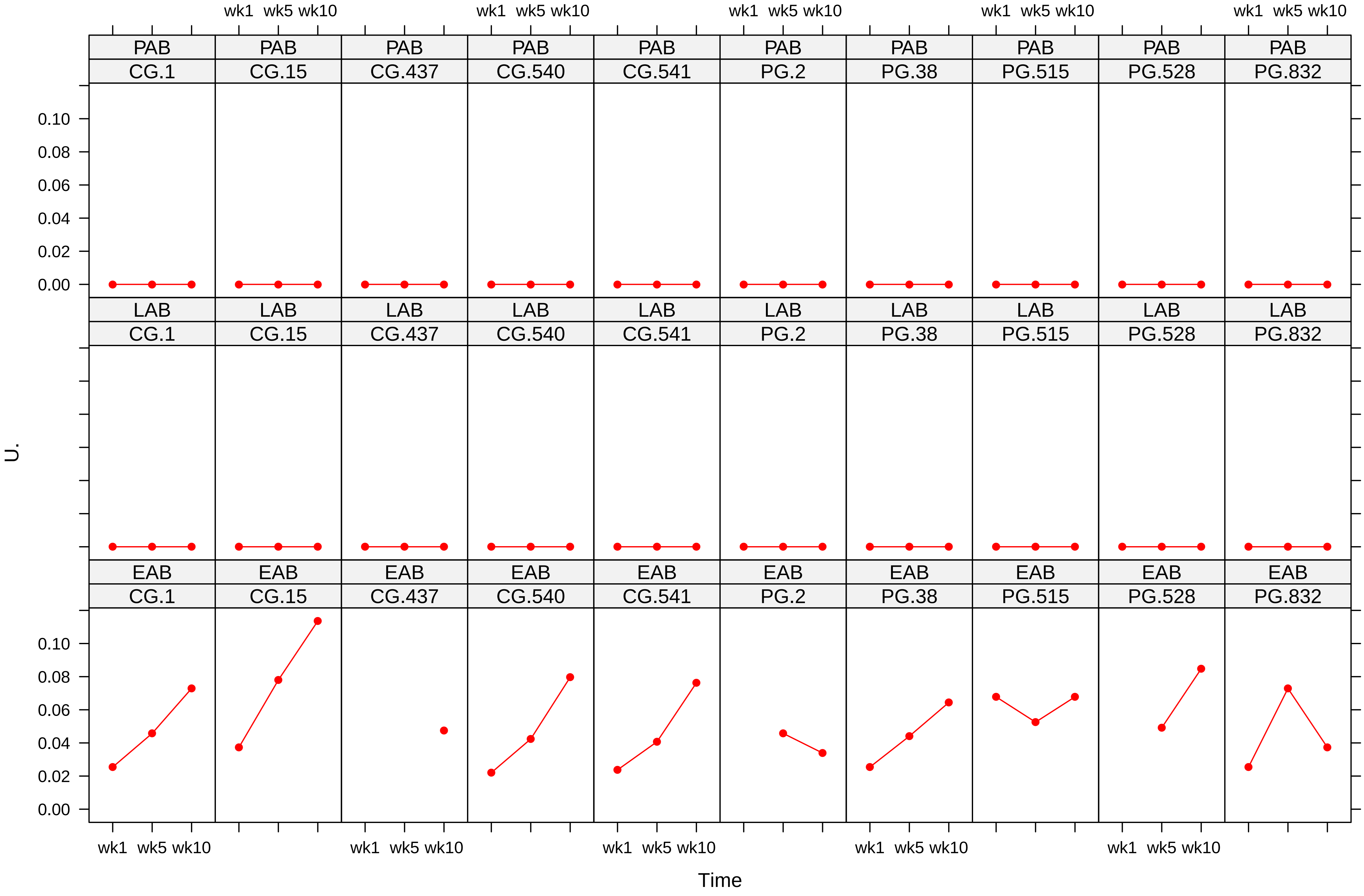

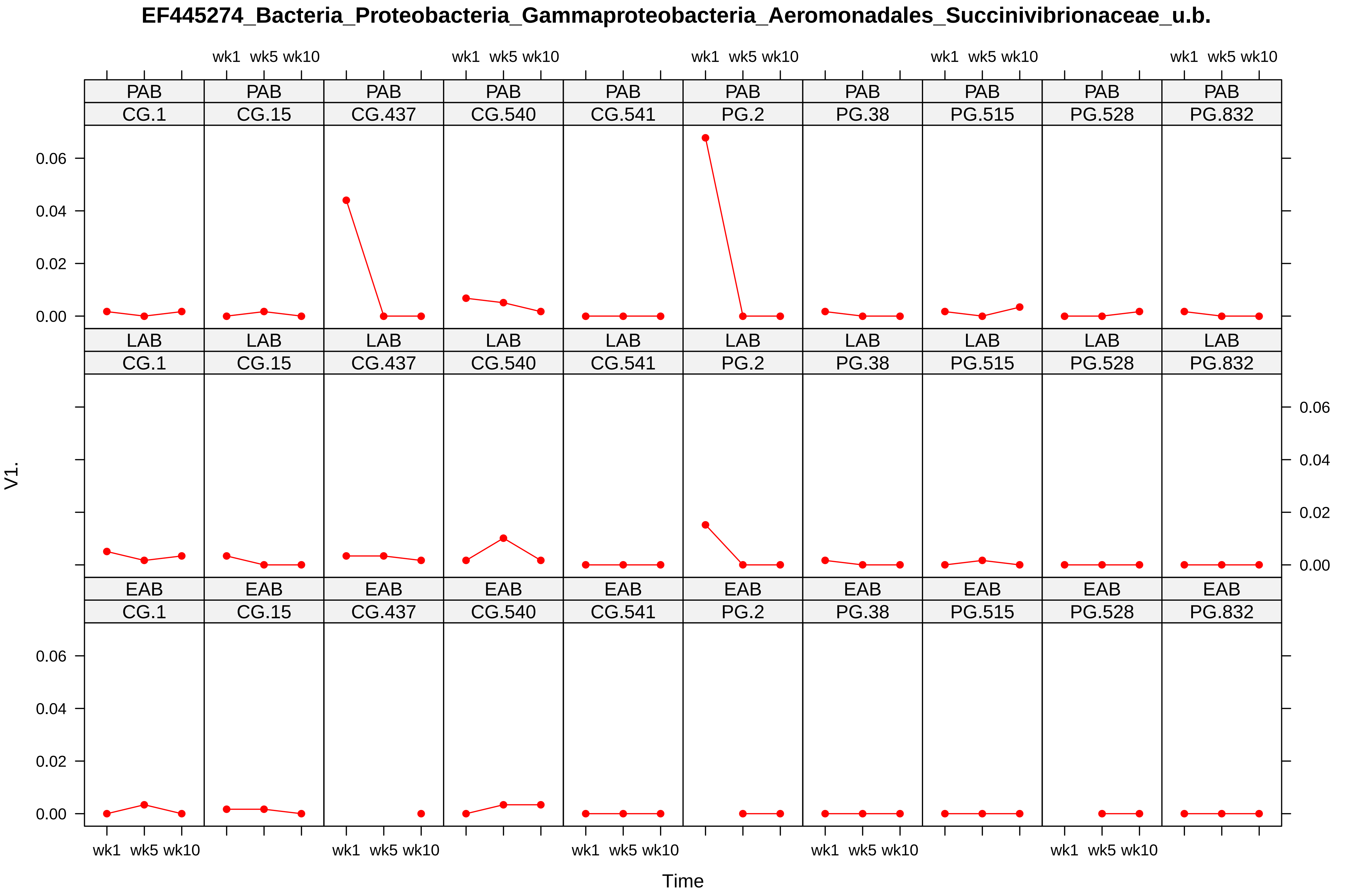

EF445274_Bacteria_Proteobacteria_Gammaproteobacteria_Aeromonadales_Succinivibrionaceae_u.b.
PAB
LAB
EAB
CG.1
CG.15
CG.437
CG.540
CG.541
PG.2
PG.38
PG.515
PG.528
PG.832
0.00
0.02
0.04
0.06
wk1
wk5
wk10
V1.
Time

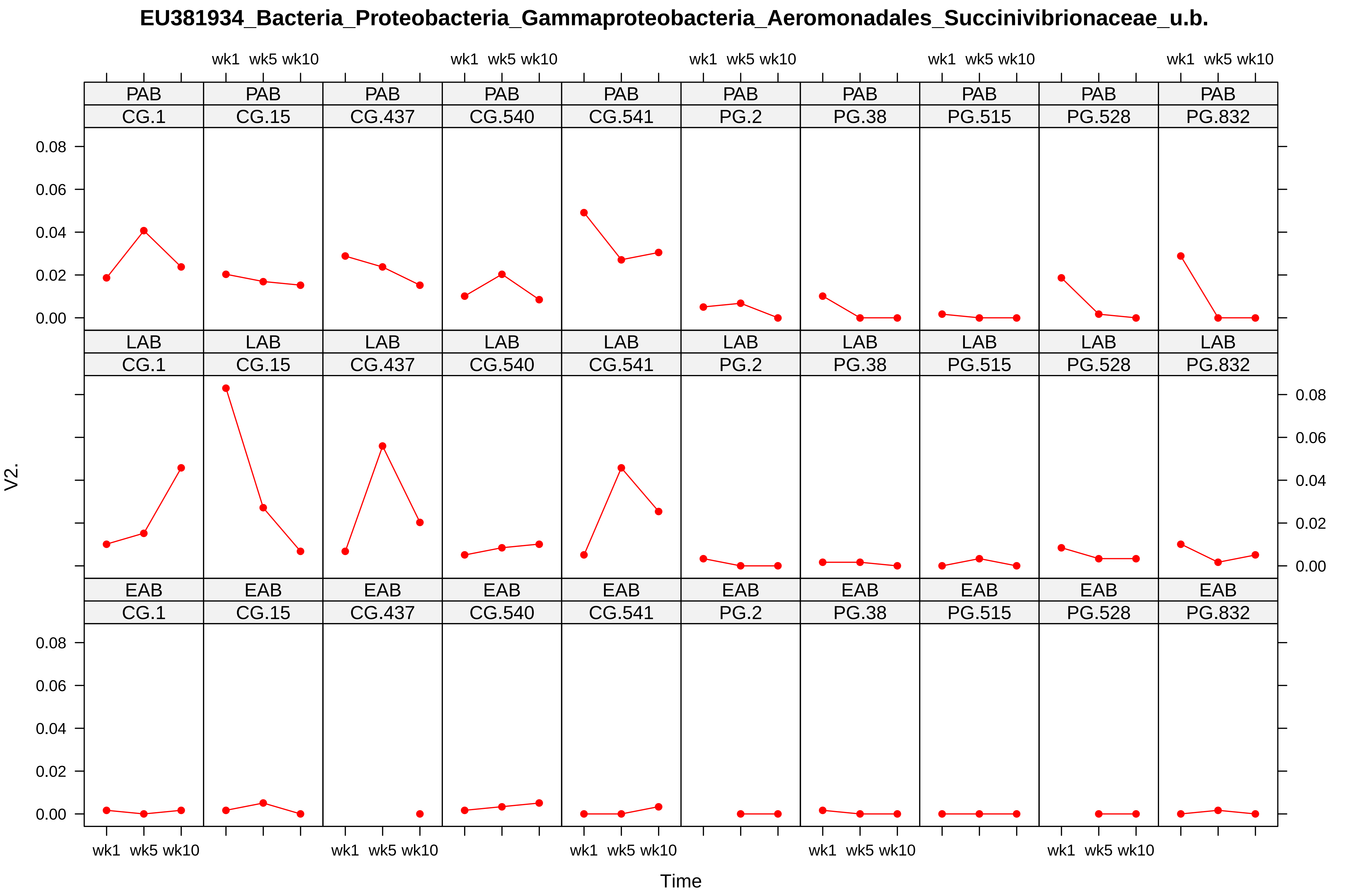
EU381934_Bacteria_Proteobacteria_Gammaproteobacteria_Aeromonadales_Succinivibrionaceae_u.b.
PAB
LAB
EAB
CG.1
CG.15
CG.437
CG.540
CG.541
PG.2
PG.38
PG.515
PG.528
PG.832
0.00
0.02
0.04
0.06
0.08
wk1 wk5 wk10
V2.
Time

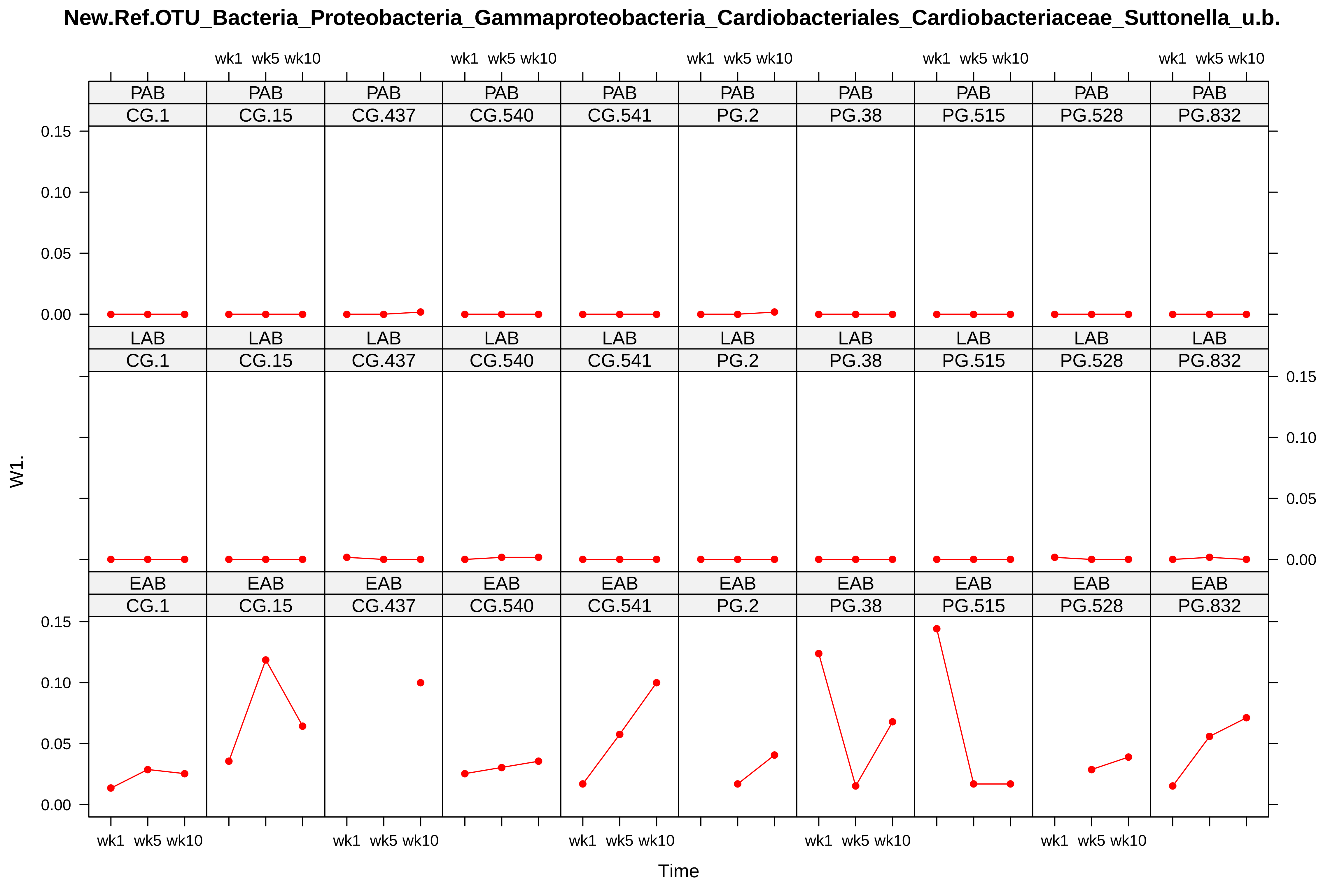

New.Ref.OTU_Bacteria_Proteobacteria_Gammaproteobacteria_Cardiobacteriales_Cardiobacteriaceae_Suttonella_u.b.
PAB CG.1
PAB CG.15
PAB CG.437
PAB CG.540
PAB CG.541
PAB PG.2
PAB PG.38
PAB PG.515
PAB PG.528
PAB PG.832
LAB CG.1
LAB CG.15
LAB CG.437
LAB CG.540
LAB CG.541
LAB PG.2
LAB PG.38
LAB PG.515
LAB PG.528
LAB PG.832
EAB CG.1
EAB CG.15
EAB CG.437
EAB CG.540
EAB CG.541
EAB PG.2
EAB PG.38
EAB PG.515
EAB PG.528
EAB PG.832
0.00
0.05
0.10
0.15
wk1 wk5 wk10
W1.
Time

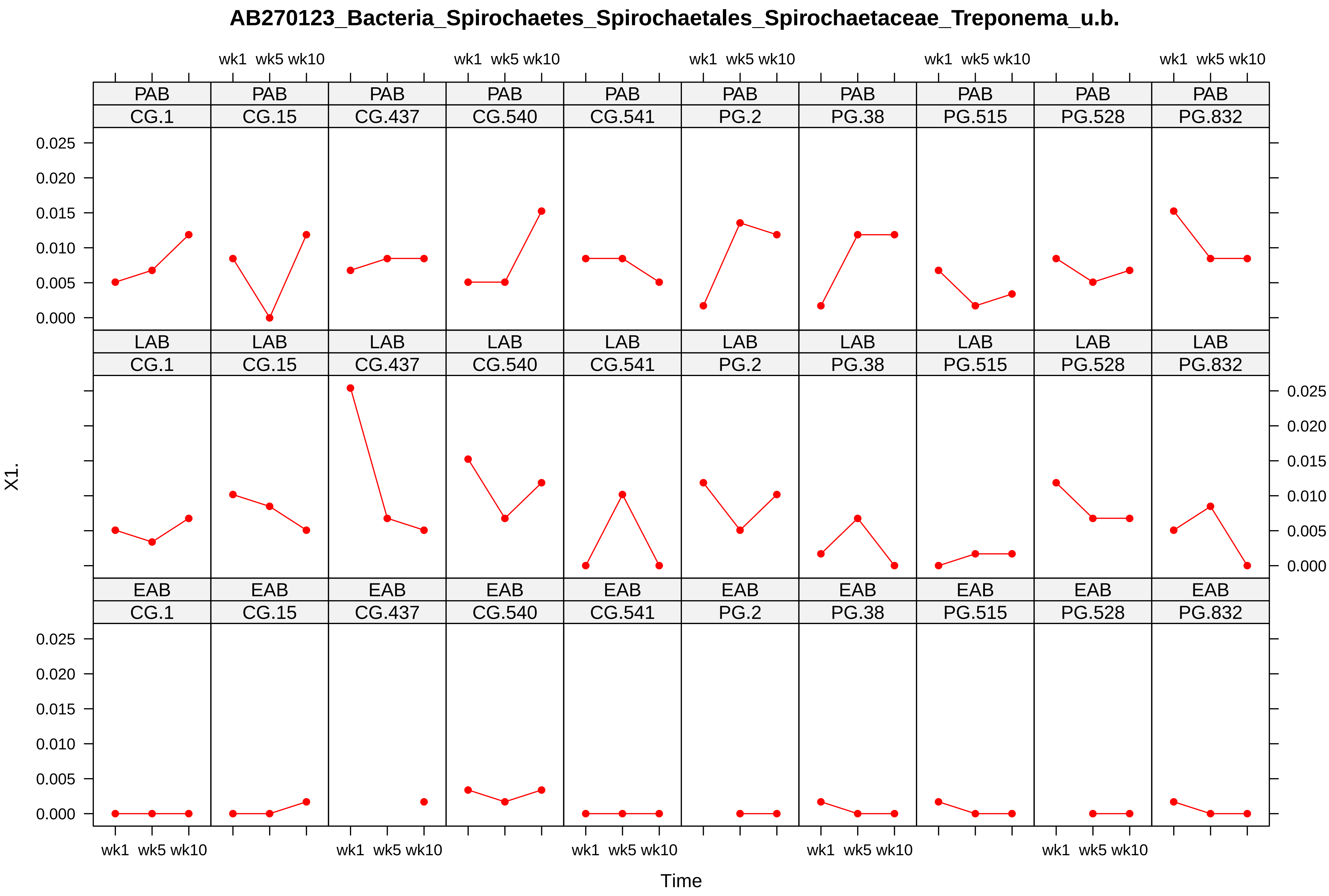

AB270123_Bacteria_Spirochaetes_Spirochaetales_Spirochaetaceae_Treponema_u.b.
PAB
LAB
EAB
CG.1
CG.15
CG.437
CG.540
CG.541
PG.2
PG.38
PG.515
PG.528
PG.832
0.000
0.005
0.010
0.015
0.020
0.025
wk1 wk5 wk10
X1.
Time

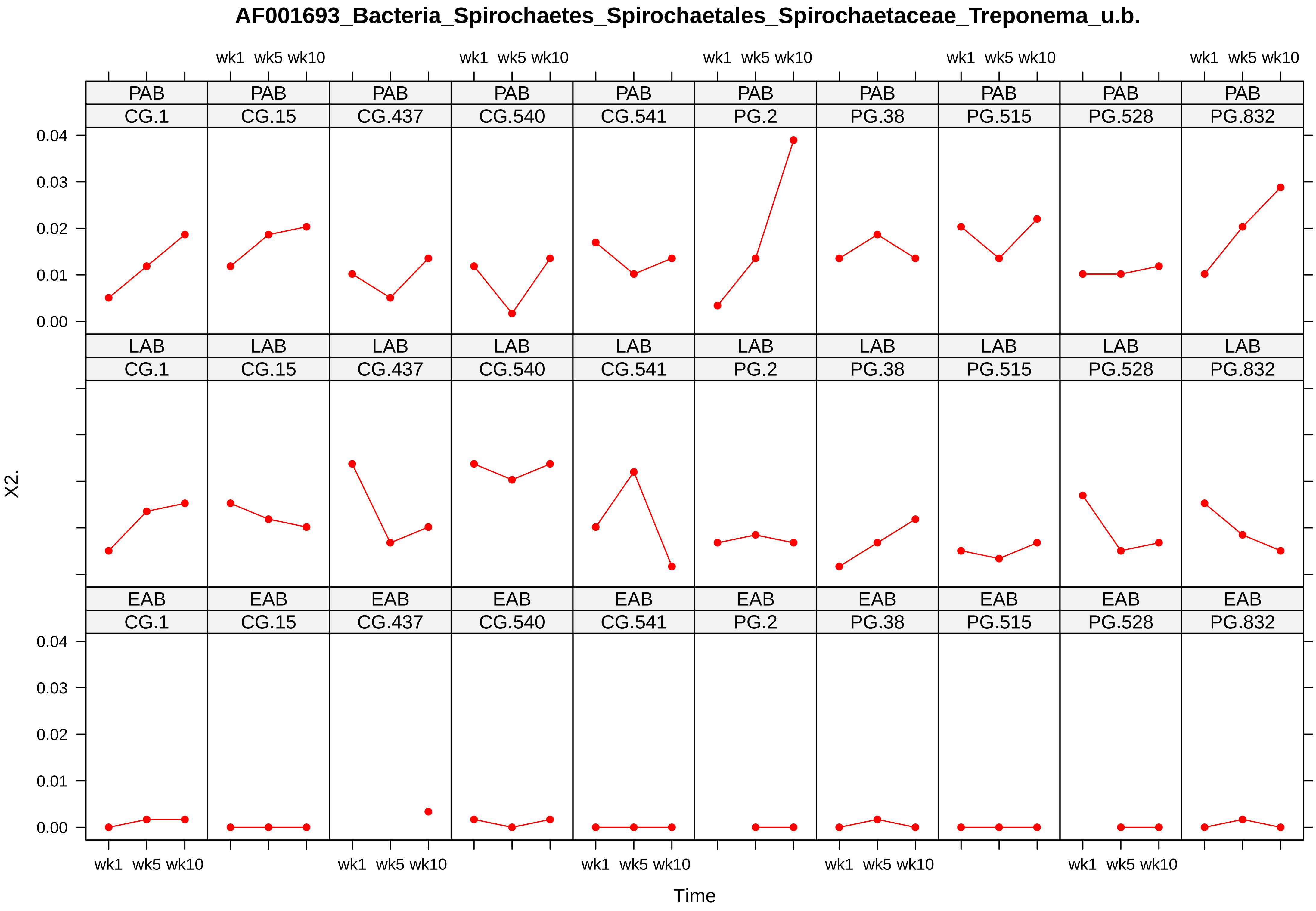
AF001693_Bacteria_Spirochaetes_Spirochaetales_Spirochaetaceae_Treponema_u.b.
PAB
LAB
EAB
CG.1
CG.15
CG.437
CG.540
CG.541
PG.2
PG.38
PG.515
PG.528
PG.832
X2.
Time
wk1 wk5 wk10
0.00
0.01
0.02
0.03
0.04

EF445251_Bacteria_Tenericutes_Mollicutes_RF9_u.b.

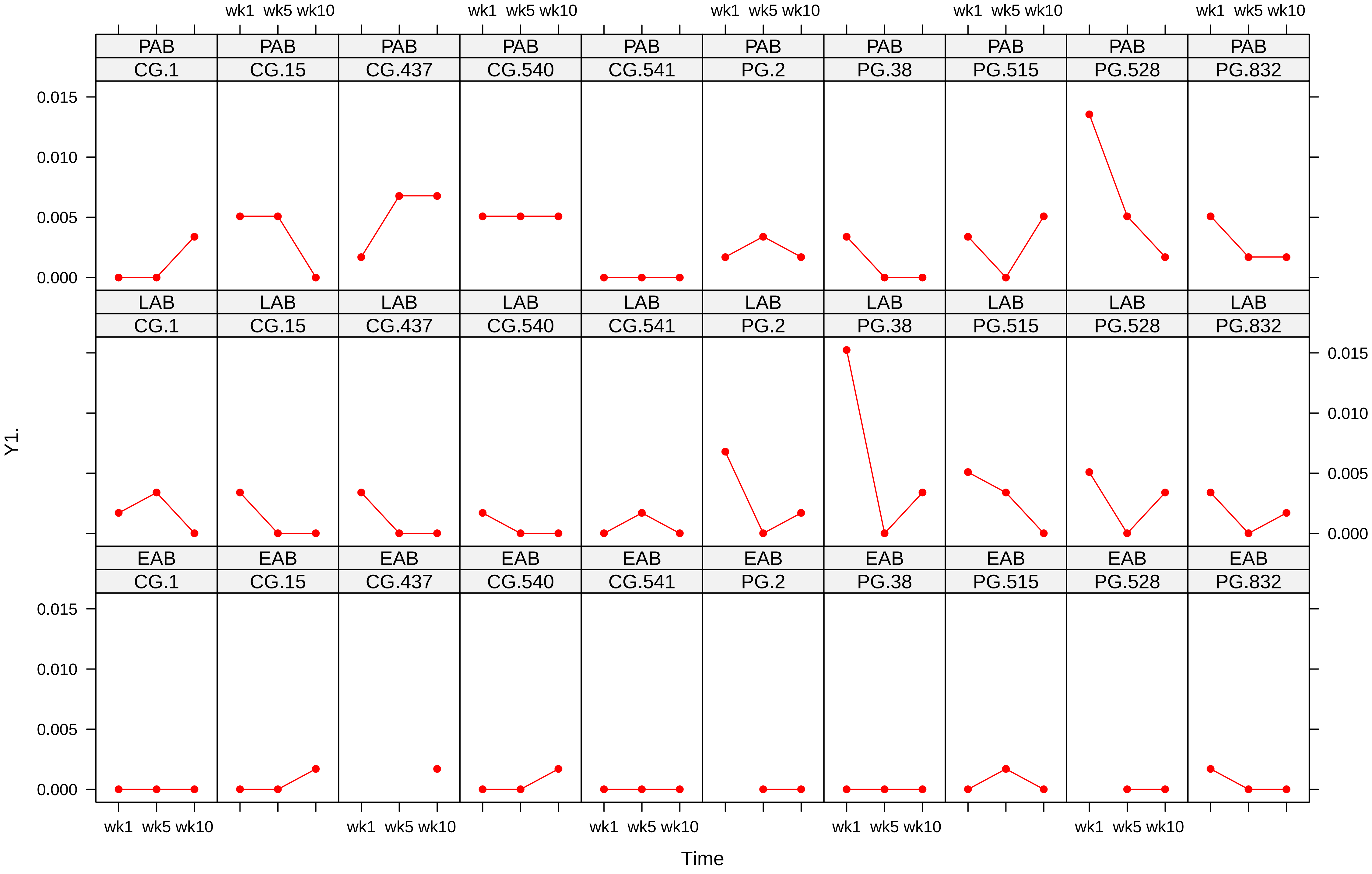

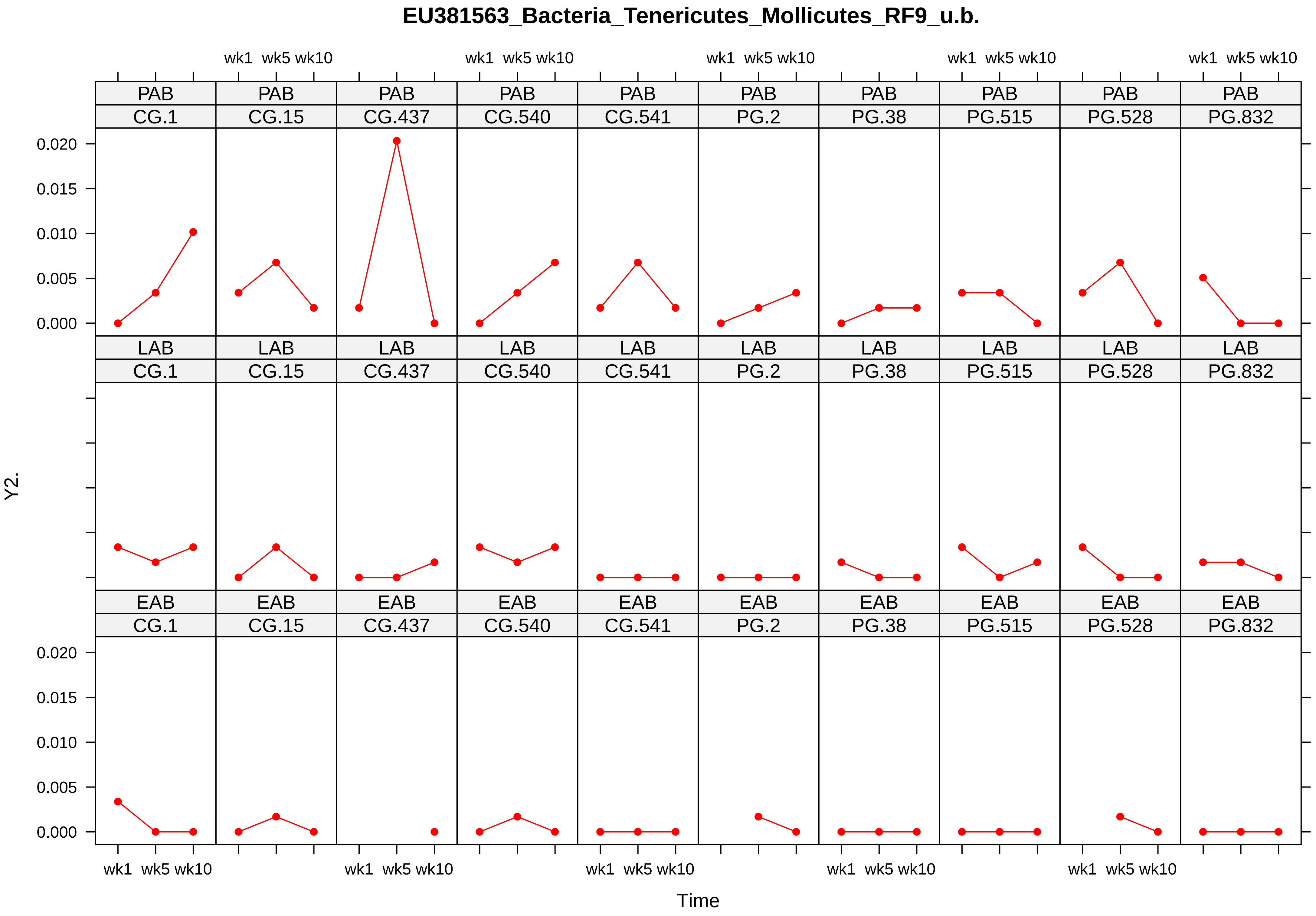

EU381563_Bacteria_Tenericutes_Mollicutes_RF9_u.b.
PAB
LAB
EAB
CG.1
CG.15
CG.437
CG.540
CG.541
PG.2
PG.38
PG.515
PG.528
PG.832
wk1
wk5
wk10
0.000
0.005
0.010
0.015
0.020
Y2.
Time

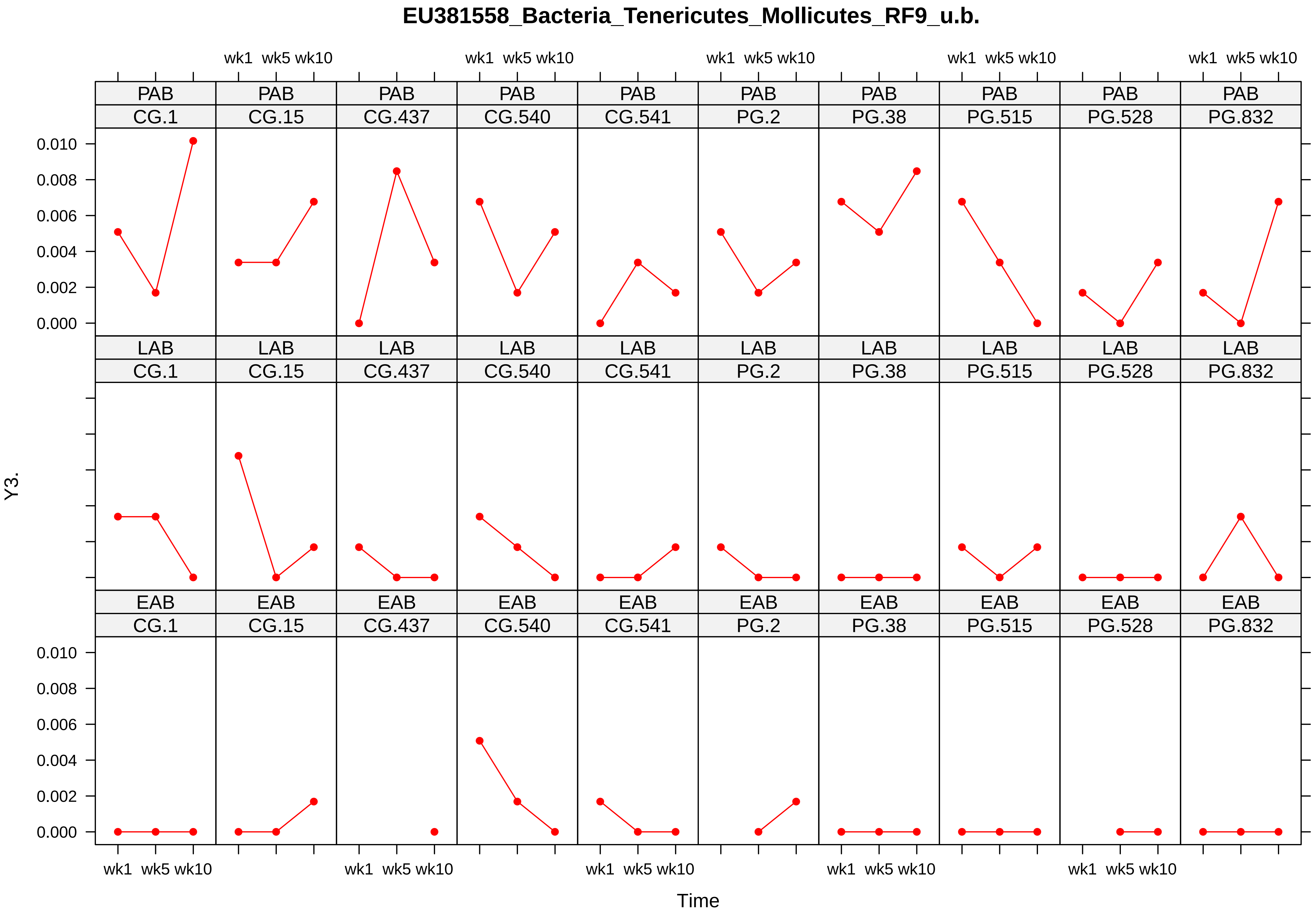

EU381558_Bacteria_Tenericutes_Mollicutes_RF9_u.b.
PAB
LAB
EAB
CG.1
CG.15
CG.437
CG.540
CG.541
PG.2
PG.38
PG.515
PG.528
PG.832
0.000
0.002
0.004
0.006
0.008
0.010
wk1 wk5 wk10
Y3.
Time

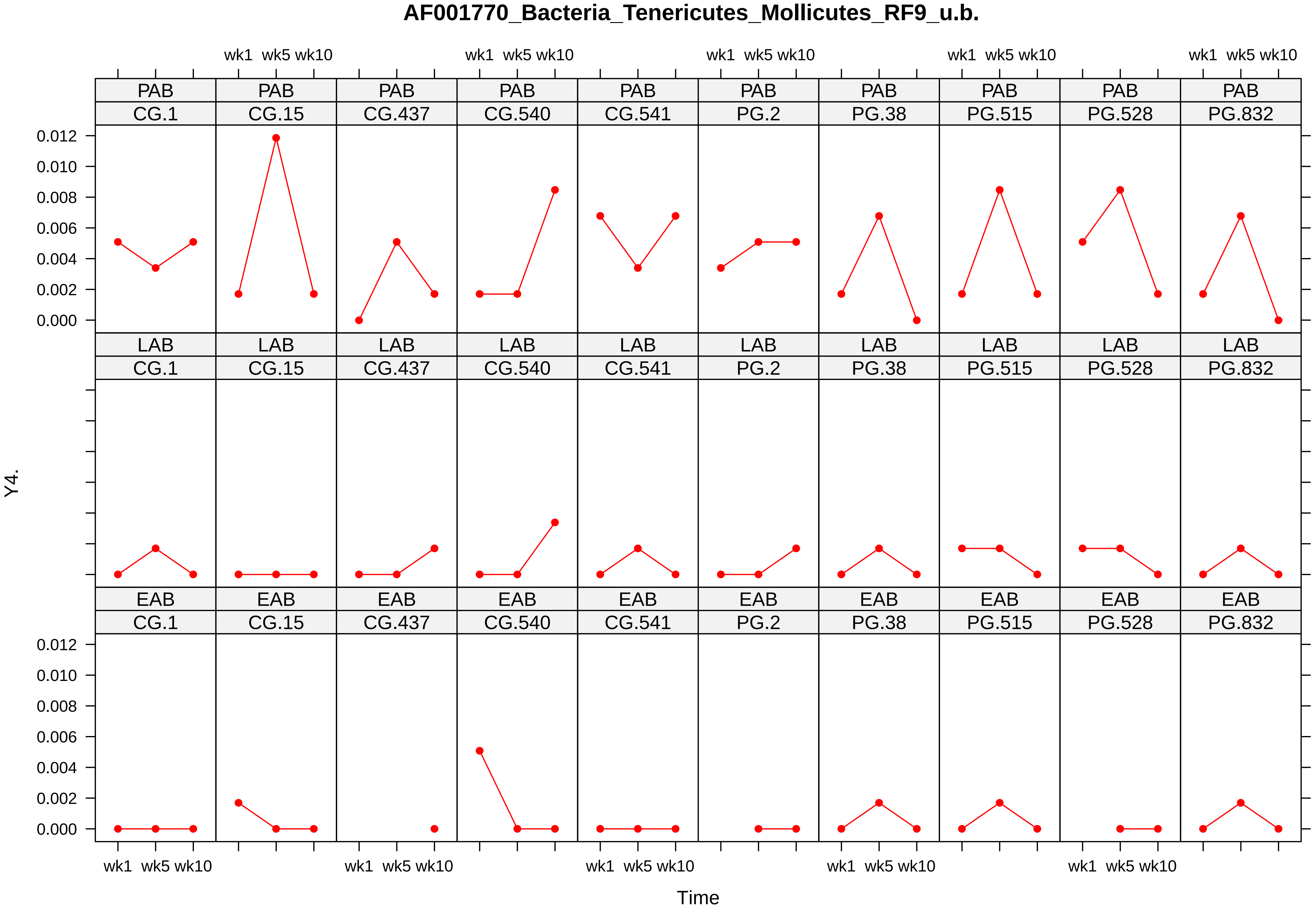
AF001770_Bacteria_Tenericutes_Mollicutes_RF9_u.b.
PAB
LAB
EAB
CG.1
CG.15
CG.437
CG.540
CG.541
PG.2
PG.38
PG.515
PG.528
PG.832
0.012
0.010
0.008
0.006
0.004
0.002
0.000
Y4.
wk1 wk5 wk10
Time
